# ﻿Segregation of the genus *Parahypoxylon* (Hypoxylaceae, Xylariales) from *Hypoxylon* by a polyphasic taxonomic approach

**DOI:** 10.3897/mycokeys.95.98125

**Published:** 2023-02-20

**Authors:** Marjorie Cedeño-Sanchez, Esteban Charria-Girón, Christopher Lambert, J. Jennifer Luangsa-ard, Cony Decock, Raimo Franke, Mark Brönstrup, Marc Stadler

**Affiliations:** 1 Dept. Microbial Drugs, Helmholtz Centre for Infectiion Research, Inhoffenstrasse 7, 38124 Braunschweig, Germany; 2 Institute of Microbiology, Technische Universität Braunschweig, Spielmannstraße 7, 38106 Braunschweig, Germany; 3 Division of Molecular Cell Biology, Zoological Institute, Technische Universität Braunschweig, Spielmannstrasse 7, 38106 Braunschweig, Germany; 4 National Center for Genetic Engineering and Biotechnology (BIOTEC), 113 Thailand Science Park, Phaholyothin Road, Klong Luang, Pathumthani 12120, Thailand; 5 Mycothéque de l’ Universite catholique de Louvain (BCCM/MUCL), Place Croix du Sud 3, B-1348 Louvain-la-Neuve, Belgium; 6 Department of Chemical Biology, Helmholtz Centre for Infection Research, Partner site Hannover/Braunschweig, Inhoffenstrasse 7, 38124 Braunschweig, Germany; 7 German Center for Infection Research (DZIF), Site Hannover-Braunschweig, 38124 Braunschweig, Germany

**Keywords:** Ascomycota, metabolite annotation, one new genus, one new species, phylogeny, polythetic taxonomy, Xylariales

## Abstract

During a mycological survey of the Democratic Republic of the Congo, a fungal specimen that morphologically resembled the American species *Hypoxylonpapillatum* was encountered. A polyphasic approach including morphological and chemotaxonomic together with a multigene phylogenetic study (ITS, LSU, *tub2*, and *rpb2*) of *Hypoxylon* spp. and representatives of related genera revealed that this strain represents a new species of the Hypoxylaceae. However, the multi-locus phylogenetic inference indicated that the new fungus clustered with *H.papillatum* in a separate clade from the other species of *Hypoxylon*. Studies by ultrahigh performance liquid chromatography coupled to diode array detection and ion mobility tandem mass spectrometry (UHPLC-DAD-IM-MS/MS) were carried out on the stromatal extracts. In particular, the MS/MS spectra of the major stromatal metabolites of these species indicated the production of hitherto unreported azaphilone pigments with a similar core scaffold to the cohaerin-type metabolites, which are exclusively found in the Hypoxylaceae. Based on these results, the new genus *Parahypoxylon* is introduced herein. Aside from *P.papillatum*, the genus also includes *P.ruwenzoriense***sp. nov.**, which clustered together with the type species within a basal clade of the Hypoxylaceae together with its sister genus *Durotheca*.

## ﻿Introduction

The genus *Hypoxylon* Bull. 1791 remains one of the largest in the Xylariales, even after a turbulent taxonomic history, during which its generic concept has changed drastically. Its early taxonomic history has been reviewed in great detail by Ju and Rogers (1996). Therefore, we largely refer to this monograph for the taxonomic treatments that occurred in the 19^th^ and early 20^th^ century.

The first world monograph of *Hypoxylon* by Miller (1961) was mainly based on stromatal morphology and ascal micromorphology. He recognized four sections (*Hypoxylon*, *Annulata*, *Applanata* and *Papillata*, the latter of which was further subdivided into two subsections, *Papillata* and *Primocinerea*). Ju and Rogers (1996) then restricted *Hypoxylon* to sections *Hypoxylon* and *Annulata*, and included several species of section *Papillata* in their emended section Hypoxylon. The main criteria for this taxonomic change were the presence of stromatal pigments and a nodulisporium-like anamorph. Many of the species in sections *Applanata* and *Papillata* sensu Miller (1961) do not show the latter mentioned features and were accommodated in other genera (e.g., *Biscogniauxia*, *Nemania*, *Whalleya*), which were later transferred to different families (Wendt et al. 2018). For their current classification, we refer to Hyde et al. (2020).

With the advent of molecular phylogenetic studies, and chemotaxonomy as an additional tool, the taxonomic concepts of *Hypoxylon* and other stromatic genera of the Xylariales have been further refined. The holomorphic concepts developed by Ju and Rogers, as well as other mycologists who put more emphasis on the anamorphic characters than on stromatal and ascospore morphology, have largely been confirmed. Hsieh et al. (2005) used protein-coding genes of a large number of representative taxa to resolve the phylogeny of *Hypoxylon* s. lat., which resulted in the recognition of the genus *Annulohypoxylon*. The composition of the latter genus was then equivalent to that of sect. Annulata sensu Ju and Rogers (1996). Notably, a parallel approach to establish a phylogeny based on ITS nrDNA sequences resulted in a very low resolution of the hypoxyloid taxa (Triebel et al. 2005). Later studies revealed that a multi locus phylogeny involving both protein-coding genes and rDNA are suitable to achieve a sufficient phylogenetic resolution within *Hypoxylon* and its allies (Kuhnert et al. 2014b, 2015, 2017a; Sir et al. 2015) in scope of a polythetic concept. Concurrent chemotaxonomic studies have aided in establishing correlations between the genotypes and the phenotypes of these pyrenomycetes. Their stromatal pigments, as well as certain secondary metabolites of their mycelial cultures, turned out to be informative for taxonomic segregation at the species or even genus level (cf. Helaly et al. 2018; Becker and Stadler 2021).

**Figure 1.**
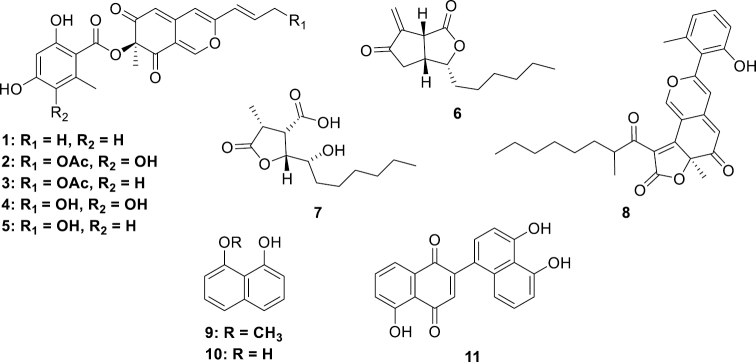
Characteristic stromatal pigments and other secondary metabolites of *Hypoxylon* species. (+)-mitorubrin (**1**); (+)-6˝-hydroxymitorubrinol acetate (**2**); (+)-mitorubrinol acetate (**3**); (+)-6˝-hydroxymitorubrinol (**4**); (+)-mitorubrinol (**5**); sporothriolide (**6**); dihydroisosporothric acid (**7**); cohaerin E (**8**); 8-methoxy naphthol (**9**); 1,8-naphthol (**10**); hypoxylone (**11**).

Based on the above accomplishments, Wendt et al. (2018) proposed a rearrangement of the families of the stromatic Xylariales, as well as the further segregation of genera from the mainstream of *Hypoxylon*. The Hypoxylaceae were resurrected to accommodate *Hypoxylon* and its closely related allies, and the Xylariaceae were restricted to the genera with geniculosporium-like anamorphs, which had already been recognized as phylogenetically distinct in earlier studies (e.g., Hsieh et al. 2010). *Annulohypoxylon* was further subdivided and largely restricted to those species that have ostiolar rings and do not produce cohaerin-type azaphilones. The genus *Jackrogersella* was erected to accommodate those species of *Annulohypoxylon* sensu Hsieh et al. (2005) that produce the aforementioned compounds and have papillate ostioles without rings. In addition, the genus *Pyrenopolyporus* was erected for species of *Hypoxylon* sensu Ju and Rogers (1996) that have massive stromata, long tubular perithecia, contain naphthopyrones in their stromata and (where this is known) produce a characteristic virgariella-like anamorph. A follow-up study by Lambert et al. (2019) provided evidence that the species of the *H.monticulosum* complex differ from *Hypoxylon* by the production of antifungal sporothriolides in culture. In addition, these fungi also lack the typical stromatal pigments of *Hypoxylon* (Fig. 1) and appear in a basal clade in the molecular phylogeny. The genus *Hypomontagnella* was therefore introduced to accommodate them.

The genus *Hypoxylon* in the current sense still appears heterogeneous and paraphyletic in the recently established phylogenies, also because its type species, *H.fragiforme* clustered in a relatively small clade comprising only a few species such as *H.howeanum*, *H.ticinense* and *H.rickii* (Wendt et al. 2018; Lambert et al. 2021). The latter species have in common that their stromatal pigments are of the mitorubrin type.

Another species that was retained in *Hypoxylon*, even though the DNA sequences of the only available strain formed an aberrant clade in the phylogeny by Wendt et al. (2018) is *H.papillatum* Ellis & Everh. This species is characterized by effused-pulvinate stromata featuring long tubular perithecia. Therefore, its stromata somewhat resemble those of *Pyrenopolyporus* and certain *Daldinia* species such as *D.placentiformis* that do not have internal concentric zones. Ju and Rogers (1996) have studied the type material and concluded that the syntypes they studied from BPI and NY (i.e., the specimens listed in the protologue by Ellis and Everhart (see Smith 1893) did not all correspond to the same taxon. They identified some of the specimens as *Hypoxylonplacentiforme* (now: *Daldiniaplacentiformis*), which was confirmed by Stadler et al. (2014) in the *Daldinia* world monograph, and selected a lectotype from Ohio (Commons No. 2160) which showed a characteristic morphology and could easily be distinguished from the former taxon. They also listed several other specimens from North America and Trinidad that showed the same characteristics.

Rogers (1985) cultured this fungus and provided a detailed description of its nodulisporium-like anamorph and culture. The corresponding specimen was collected by him in West Virginia, USA, and could have served as epitype. The culture is deposited in ATCC and showed the typical characteristics of *H.papillatum* sensu Rogers (1985) and was included in the phylogeny by Wendt et al. (2018) as a representative of this taxon. However, it showed an aberrant phylogenetic position in a clade that appeared basal to the others in which the DNA sequences of *Hypoxylon* species were located. We have come across a very similar fungus that was collected in Central Africa and have studied it, along with several extant type and authentic specimens for comparison. The results of this study, which also relies on state-of-the art metabolomics, are reported herein.

## ﻿Materials and methods

### ﻿Sample sources

All scientific names of fungi are given without authorities or publication details, according to Index Fungorum (http://www.indexfungorum.org). Type and reference specimens were provided by Washington State University herbarium (**WSP**), U.S. National Fungus Collections (**BPI**) and the New York Botanical Garden (**NY**), USA. Fungal cultures were provided from the Belgian Coordinated Collections of Microorganisms (**MUCL**), Belgium and the Westerdijk Fungal Biodiversity Institute (**CBS**), The Netherlands.

### ﻿Morphological characterization

The microscopic characteristics of the teleomorph were carried out as described by Pourmoghaddam et al. (2020). To observe the macro-morphology of the cultures, the strains were grown on Difco Oatmeal Agar (**OA**), 2% Malt Extract Agar (**MEA**) and Yeast Malt agar (**YM** agar; malt extract 10 g/L, yeast extract 4 g/L, D-glucose 4 g/L, agar 20 g/L, pH 6.3 before autoclaving) and the cultures checked at 15 days after inoculation. Pigment colors were determined following the color-codes by Rayner (1970).

### ﻿DNA extraction, PCR and sequencing

The DNA was extracted from pure cultures grown on plates with YM agar. Small amounts of mycelia were harvested after five days of growth and transferred to a 1.5 ml homogenization tube filled with six to eight Precellys Ceramic beads (1.4 mm, Bertin Technologies, Montigny-le-Bretonneux, France).

DNA extraction was performed using the commercially available Fungal gDNA Miniprep Kit EZ-10 spin column (NBS Biologicals, Cambridgeshire, UK) following the manufacturer’s instructions. The *tub2* (partial β-tubulin) gene region was amplified using the primers T1 and T22 (O’Donnell and Cigelnik 1997); ITS (nuc rDNA internal transcribed spacer) region using the primers ITS4 and ITS5 (White et al. 1990); LSU (Large subunit nuc 28S rDNA) using LR0R and LR7 (Vilgalys and Hester 1990) and *rpb2* (partial second largest subunit of the DNA-directed RNA polymerase II) using fRPB2-5F and fRPB2-7cR (Liu et al. 1999).

PCR reactions were performed by mixing template gDNA (2–3 µL), 12.5 µL JumpStart Taq Ready Mix (Sigma Aldrich, Deisenhofen, Germany), 0.5 µL of both forward and reverse primers (10 mM) and 8.5 to 9.5 μl of sterile filtered and sterilized water to a final volume of 25 µL. Amplification was achieved using a Mastercycler nexus Gradient (Eppendorf, Hamburg, Germany). Thermocycling for ITS commenced with an initial denaturation at 94 °C for 5 min followed by 34 cycles of denaturation (30 s at 94 °C), annealing (30 s at 52 °C), and elongation (1 min at 72 °C). The program concluded with a 10 min lasting elongation at 72 °C and reaction tubes were stored at 4 °C until further use. In the case of the other loci, the following steps were modified: LSU denaturation (1 min at 94 °C), annealing (1 min at 52 °C), and elongation (2 min at 72 °C); For *tub2* the cycle repetitions were raised to 38, annealing (30 s at 47 °C) and elongation (2 min 30 s at 72 °C); for *rpb2*, the cycle repetitions were raised to 38, annealing (1 min at 54 °C) and elongation (1 min 30 s at 72 °C).

### ﻿Molecular phylogenetic analyses

Sequences were analyzed and processed in Geneious 7.1.9 (Kearse et al. 2012). The generated sequence data were complemented by available sequence data from GenBank and the data sets for each genetic marker were aligned using MAFFT online (http://mafft.cbrc.jp/alignment/server/, Katoh et al. 2019), and manually curated in MEGA 11 (Tamura et al. 2021). A maximum-likelihood phylogenetic tree was constructed using IQ-TREE v. 2.1.3 [-b 1000 -abayes -m MFP -nt AUTO] (Minh et al. 2020), The selection of the appropriate nucleotide exchange model was selected by ModelFinder (Chernomor et al. 2016; Kalyaanamoorthy et al. 2017) based on Bayesian inference criterion. Branch support was calculated with non-parametric bootstrap (Felsenstein 1985 and approximate Bayes test (Anisimova et al. 2011). The total 1000 bootstrap replicates were mapped onto the ML tree with the best (highest) ML score. Single locus trees were calculated following the identical methodology and checked for congruence with the multigene phylogenetic tree.

A second phylogenetic inference was carried out following a Bayesian approach using MrBayes 3.2.7a (Ronquist et al. 2012) with algorithm options set to the ones reported by Matio Kemkuignou et al. (2022). The data matrix was subjected to PartitionFinder2 (Lanfear et al. 2016) as implemented in the program package phylosuite v. 1.2.2 (Zhang et al. 2020) with settings set to an un-linked determination of the best-fitting nucleotide substitution models following Bayesian information criterion (BIC) for the different partitions, restricted to the ones available in MrBayes. Posterior probabilities (PP) above 95% were regarded as significant. To determine the congruence of the topologies of ML and Bayes, an approximate unbiased (AU) topology test was carried out in IQ-TREE [iqtree -s example.phy -z example.treels -n 0 -zb 10000 -zw -au](Shimodaira 2022). All sequences used for the pyhlogeny are listed in Table 1.

**Table 1.** Strains used in the phylogenetic analyses, including the strain IDs, GenBank accession numbers, and the references where the sequence data have been originally generated. Type specimens are labeled with T (holotype), IT (isotype), PT (paratype) and ET (epitype).

Species	Strain number	GenBank Accession Number				Origin	References
		ITS	LSU	*rpb2*	*tub*2		
*Annulohypoxylonannulatum*	CBS 140775	KY610418	KY610418	KY624263	KX376353	USA (ET)	Kuhnert et al. (2017a; *tub2*), Wendt et al. (2018: ITS, LSU, *rpb2*)
*Annulohypoxylonmichelianum*	CBS 119993	KX376320	KY610423	KY624234	KX271239	Spain	Kuhnert et al. (2014a; ITS, *tub2*), Wendt et al. (2018; LSU, *rpb2*)
*Annulohypoxylontruncatum*	CBS 140778	KY610419	KY610419	KY624277	KX376352	USA (ET)	Kuhnert et al. (2017a; *tub2*), Wendt et al. (2018; ITS, LSU, *rpb2*)
*Daldiniabambusicola*	CBS 122872	KY610385	KY610431	KY624241	AY951688	Thailand (T)	Hsieh et al. (2005; *tub2*), Wendt et al. (2018; ITS, LSU, *rpb2*)
*Daldiniachildiae*	CBS 122881	KU683757	MH874773	KU684290	KU684129	France (T)	U’Ren et al. (2016; ITS, *tub2*, *rpb2*), Vu et al. (2019; LSU)
*Daldiniaconcentrica*	CBS 113277	AY616683	KY610434	KY624243	KC977274	Germany	Triebel et al. (2005; ITS), Kuhnert et al. (2014a; *tub2*), Wendt et al. (2018; LSU, *rpb2*)
*Daldiniadennisii*	CBS 114741	JX658477	KY610435	KY624244	KC977262	Australia (T)	Stadler et al. (2014; ITS), Kuhnert et al. (2014a; *tub2*), Wendt et al. (2018; LSU, *rpb2*)
*Daldiniaeschscholtzii*	MUCL 45435	JX658484	KY610437	KY624246	KC977266	Benin	Stadler et al. (2014a; ITS), Kuhnert et al. (2014a; *tub2*), Wendt et al. (2018; LSU, *rpb2*)
*Daldiniapetriniae*	MUCL 49214	AM749937	KY610439	KY624248	KC977261	Austria (ET)	Bitzer et al. (2008; ITS), Kuhnert et al. (2014a; *tub2*), Wendt et al. (2018; LSU, *rpb2*)
*Daldiniaplacentiformis*	MUCL 47603	AM749921	KY610440	KY624249	KC977278	Mexico	Stadler et al. (2014a; ITS), Kuhnert et al. (2014a; *tub2*), Wendt et al. (2018; LSU, *rpb2*)
*Daldiniavernicosa*	CBS 119316	KY610395	KY610442	KY624252	KC977260	Germany (ET)	Kuhnert et al. (2014a; *tub2*), Wendt et al. (2018; ITS, LSU, *rpb2*)
*Durothecarogersii*	YMJ 92031201	EF026127		JX507794	EF025612	Taiwan	Ju et al. (2007) as *Theissenia*
*Durothecacomedens*	YMJ 90071615	EF026128		JX507793	EF025613	Taiwan (T)	Ju et al. (2003) as *Theissenia*
*Durothecacrateriformis*	GMBC0205	MH645426	MH645425	MH645427	MH049441	China (T)	de Long et al. (2019)
*Durothecaguizhouensis*	GMBC0065	MH645423	MH645421	MH645422	MH049439	China (T)	de Long et al. (2019)
*Durothecarogersii*	GMBC0204	MH645433	MH645434	MH645435	MH049449	China	de Long et al. (2019)
*Graphostromaplatystomum*	CBS 270.87	JX658535	DQ836906	KY624296	HG934108	France (T)	Zhang et al. (2006; LSU), Stadler et al. (2014; ITS), Koukol et al. (2015; *tub2*), Wendt et al. (2018; *rpb2*)
*Hypomontagnellabarbarensis*	STMA 14081	MK131720	MK131718	MK135891	MK135893	Argentina (T)	Lambert et al. (2019)
*Hypomontagnellamonticulosa*	MUCL 54604	KY610404	KY610487	KY624305	KX271273	French Guiana	Wendt et al. (2018)
*Hypomontagnellasubmonticulosa*	CBS 115280	KC968923	KY610457	KY624226	KC977267	France	Kuhnert et al. (2014a; ITS, *tub2*), Wendt et al. (2018; LSU, *rpb2*)
*Hypoxylonaddis*	MUCL 52797	KC968931	ON954141	OP251037	KC977287	Ethiopia (T)	Kuhnert et al. (2014a; ITS, *tub2*), This study
*Hypoxylonaveirense*	MUM 19.40	MN053021	ON954142	OP251028	MN066636	Portugal (T)	Vicente et al. (2021; ITS, *tub2*), This study
*Hypoxylonbaruense*	UCH9545	MN056428	ON954143		MK908142	Panama (T)	Cedeño–Sanchez et al. (2020; ITS, *tub2*); This study
*Hypoxyloncanariense*	MUCL 47224	ON792787	ON954140	OP251029	ON813073	Spain, Canary Islands (PT)	This study. (Species described by Stadler et al. 2008)
*Hypoxyloncarneum*	MUCL 54177	KY610400	KY610480	KY624297	KX271270	France	Wendt et al. (2018)
*Hypoxyloncercidicola*	CBS 119009	KC968908	KY610444	KY624254	KC977263	France	Kuhnert et al. (2014a; ITS, *tub2*), Wendt et al. (2018; LSU, *rpb2*)
*Hypoxylonchionostomum*	STMA 14060	KU604563	ON954144	OP251030	ON813072	Argentina	Sir et al. (2016; ITS); This study
*Hypoxylonchrysalidosporum*	FCATAS2710	OL467294	OL615106	OL584222	OL584229	China (T)	Ma et al. (2022)
*Hypoxyloncrocopeplum*	CBS 119004	KC968907	KY610445	KY624255	KC977268	France	Kuhnert et al. (2014a; ITS, *tub2*), Wendt et al. (2018; LSU, *rpb2*)
*Hypoxyloncyclobalanopsidis*	FCATAS2714	OL467298	OL615108	OL584225	OL584232	China (T)	Ma et al. (2022)
*Hypoxylonerythrostroma*	MUCL 53759	KC968910	ON954154	OP251031	KC977296	Martinique	Kuhnert et al. (2014a; ITS2, TUB), This study
*Hypoxyloneurasiaticum*	MUCL 57720	MW367851		MW373852	MW373861	Iran (T)	Lambert et al. (2021)
*Hypoxylonfendleri*	MUCL 54792	KF234421	KY610481	KY624298	KF300547	French Guiana	Kuhnert et al. (2014a; ITS, *tub2*), Wendt et al. (2018; LSU, *rpb2*)
*Hypoxylonferrugineum*	CBS 141259	KX090079			KX090080	Austria	Friebes and Wendelin (2016)
*Hypoxylonfragiforme*	MUCL 51264	KC477229	KM186295	MK887342	KX271282	Germany (ET)	Stadler et al. (2013; ITS), Daranagama et al. (2015; LSU, *rpb2*), Wendt et al. (2018; *tub2*)
*Hypoxylonfuscoides*	MUCL 52670	ON792789	ON954145	OP251038	ON813076	France (T)	This study. (Species described by Fournier et al. 2010a)
*Hypoxylonfuscum*	CBS 113049	KY610401	KY610482	KY624299	KX271271	Germany (ET)	Wendt et al. (2018)
*Hypoxylongibriacense*	MUCL 52698	KC968930	ON954146	OP251026	ON813074	France (T)	Kuhnert et al. (2014a; ITS). This study
*Hypoxylongriseobrunneum*	CBS 331.73	KY610402	KY610483	KY624300	KC977303	India (T)	Kuhnert et al. (2014a; *tub2*), Wendt et al. (2018; ITS, LSU, *rpb2*)
*Hypoxylonguilanense*	MUCL 57726	MT214997	MT214992	MT212235	MT212239	Iran (T)	Pourmoghaddam et al. (2020)
*Hypoxylonhaematostroma*	MUCL 53301	KC968911	KY610484	KY624301	KC977291	Martinique (ET)	Wendt et al. (2018; LSU, *rpb2*), Kuhnert et al. (2014a; ITS, *tub2*),
*Hypoxylonhainanense*	FCATAS2712	OL467296	OL616132	OL584224	OL584231	China (T)	Ma et al. (2022)
*Hypoxylonhinnuleum*	ATCC 36255, MUCL 3621	MK287537	MK287549	MK287562	MK287575	USA (T)	Sir et al. (2019)
*Hypoxylonhoweanum*	MUCL 47599	AM749928	KY610448	KY624258	KC977277	Germany	Bitzer et al. (2008; ITS), Kuhnert et al. (2014a; *tub2*), Wendt et al. (2018; LSU, *rpb2*)
*Hypoxylonhypomiltum*	MUCL 51845	KY610403	KY610449	KY624302	KX271249	Guadeloupe	Wendt et al. (2018)
*Hypoxyloninvadens*	MUCL 51475	MT809133	MT809132	MT813037	MT813038	France (T)	Becker et al. (2020)
*Hypoxyloninvestiens*	CBS 118183	KC968925	KY610450	KY624259	KC977270	Malaysia	Kuhnert et al. (2014a; ITS, *tub2*), Wendt et al. (2018; LSU, *rpb2*)
*Hypoxylonisabellinum*	MUCL 53308	KC968935	ON954155	OP251032	KC977295	Martinique (T)	Kuhnert et al. (2014a; ITS, *tub2*), This study
*Hypoxylonlaschii*	MUCL 52796	JX658525	ON954147	OP251027	ON813075	France	Stadler et al. (2014; ITS), This study
*Hypoxylonlateripigmentum*	MUCL 53304	KC968933	KY610486	KY624304	KC977290	Martinique (T)	Kuhnert et al. (2014a; ITS, *tub2*), Wendt et al. (2018; LSU, *rpb2*)
*Hypoxylonlechatii*	MUCL 54609	KF923407	ON954148	OP251033	KF923405	French Guiana	Kuhnert et al. (2014b; ITS, *tub2*), This study
*Hypoxylonlenormandii*	CBS 119003	KC968943	KY610452	KY624261	KC977273	Ecuador	Kuhnert et al. (2014a; ITS, *tub2*), Wendt et al. (2018; LSU, *rpb2*)
*Hypoxylonlienhwacheense*	MFLUCC 14-1231	KU604558	MK287550	MK287563	KU159522	Thailand	Sir et al. (2016; ITS, *tub2*), Sir et al. (2019; LSU, *rpb2*)
*Hypoxylonlividipigmentum*	STMA14045	ON792788	ON954149		ON813077	Argentina	This study
*Hypoxylonlividipigmentum*	BCRC 34077	JN979433			AY951735	Mexico (IT)	Hsieh et al. (2005)
*Hypoxylonmacrocarpum*	CBS119012	ON792785	ON954151	OP251034	ON813071	Germany	This study
*Hypoxylonmunkii*	MUCL 53315	KC968912	ON954153	OP251035	KC977294	Martinique	Kuhnert et al. (2014a; ITS, *tub2*), This study
*Hypoxylonmusceum*	MUCL 53765	KC968926	KY610488	KY624306	KC977280	Guadeloupe	Kuhnert et al. (2014a; ITS, *tub2*), Wendt et al. (2018; LSU, *rpb2*)
*Hypoxylonochraceum*	MUCL 54625	KC968937		KY624271	KC977300	Martinique (ET)	Kuhnert et al. (2014a; ITS, *tub2*), Wendt et al. (2018; *rpb2*)
*Hypoxylonolivaceopigmentum*	DSM 107924	MK287530	MK287542	MK287555	MK287568	USA (T)	Sir et al. (2019)
*Hypoxylonperforatum*	CBS115281	KY610391	KY610455	KY624224	KX271250	France	Wendt et al. (2018)
*Hypoxylonpetriniae*	CBS 114746	KY610405	KY610491	KY624279	KX271274	France (T)	Wendt et al. (2018)
*Hypoxylonpilgerianum*	STMA 13455	KY610412	KY610412	KY624308	KY624315	Martinique	Wendt et al. (2018)
*Hypoxylonporphyreum*	CBS 119022	KC968921	KY610456	KY624225	KC977264	France	Kuhnert et al. (2014a; ITS, *tub2*), Wendt et al. (2018; LSU, *rpb2*)
*Hypoxylonpseudofuscum*	DSM112038	MW367857	MW367848	MW373858	MW373867	Germany (T)	Lambert et al. (2021)
*Hypoxylonpulicicidum*	CBS 122622	JX183075	KY610492	KY624280	JX183072	Martinique (T)	Bills et al. (2012; ITS, *tub2*), Wendt et al. (2018; LSU, *rpb2*)
*Hypoxylonrickii*	MUCL 53309	KC968932	KY610416	KY624281	KC977288	Martinique (ET)	Kuhnert et al. (2014a; ITS, *tub2*), Wendt et al. (2018; LSU, *rpb2*)
*Hypoxylonrubiginosum*	MUCL 52887	KC477232	KY610469	KY624266	KY624311	Germany (ET)	Stadler et al. (2013; ITS), Wendt et al. (2018; *tub2*, LSU, *rpb2*)
*Hypoxylonsamuelsii*	MUCL 51843	KC968916	KY610466	KY624269	KC977286	Guadeloupe (ET)	Kuhnert et al. (2014a; ITS, *tub2*), Wendt et al. (2018; LSU, *rpb2*)
*Hypoxylonsporistriatatunicum*		MN056426	ON954150	OP251036	MK908140	Panama (T)	Cedeño-Sanchez et al. (2020; ITS, *tub2*); This study
*Hypoxylonsubticinense*	MUCL 53752	KC968913	ON954152		KC977297	French Guiana	Kuhnert et al. (2014a; ITS, *tub2*), This study
*Hypoxylontexense*	DSM 107933	MK287536	MK287548	MK287561	MK287574	USA (T)	Sir et al. (2019)
*Hypoxylonticinense*	CBS 115271	JQ009317	KY610471	KY624272	AY951757	France	Hsieh et al. (2005; ITS, *tub2*), Wendt et al. (2018; LSU, *rpb2*)
*Hypoxylontrugodes*	MUCL 54794	KF234422	KY610493	KY624282	KF300548	Sri Lanka (ET)	Kuhnert et al. (2014a; ITS, *tub2*), Wendt et al. (2018; LSU, *rpb2*)
*Hypoxylonvogesiacum*	CBS 115273	KC968920	KY610417	KY624283	KX271275	France	Kuhnert et al. (2014a; ITS), Kuhnert et al. (2017a; *tub2*), Wendt et al. (2018; LSU, *rpb2*)
*Hypoxylonwuzhishanense*	FCATAS2708	OL467292	OL615104	OL584220	OL584227	China (T)	Ma et al. (2022)
*Jackrogersellacohaerens*	CBS 119126	KY610396	KY610497	KY624270	KY624314	Germany	Wendt et al. (2018)
*Jackrogersellamultiformis*	CBS 119016	KC477234	KY610473	KY624290	KX271262	Germany (ET)	Kuhnert et al. (2014a ; ITS), Kuhnert et al. (2017a; *tub2*), Wendt et al. (2018; LSU, *rpb2*)
*Natonodosaspeciosa*	CLM-RV86	MF380435	MF380435	MH745150		Mexico (T)	Heredia et al. (2020)
*Parahypoxylonpapillatum* comb. nov.	ATCC 58729	KC968919	KY610454	KY624223	KC977258	USA (T)	Kuhnert et al. (2014a; ITS, *tub2*), Wendt et al. (2018; LSU, *rpb2*)
*Parahypoxylonruwenzoriense* sp. nov.	MUCL51392	ON792786	ON954156	OP251039	ON813078	D. R. Congo (T)	This study
*Pyrenopolyporushunteri*	MUCL 52673	KY610421	KY610472	KY624309	KU159530	Ivory Coast (ET)	Kuhnert et al. (2017a; *tub2*), Wendt et al. (2018; ITS, LSU, *rpb2*)
*Pyrenopolyporuslaminosus*	MUCL 53305	KC968934	KY610485	KY624303	KC977292	Martinique (T)	Kuhnert et al. (2014a; ITS, *tub2*), Wendt et al. (2018; LSU, *rpb2*)
*Pyrenopolyporusnicaraguense*	CBS 117739	AM749922	KY610489	KY624307	KC977272	Burkina_Faso	Bitzer et al. (2008; ITS), Kuhnert et al. (2014a; *tub2*), Wendt et al. (2018; LSU, *rpb2*)
*Rhopalostromaangolense*	CBS 126414	KY610420	KY610459	KY624228	KX271277	Ivory Coast	Wendt et al. (2018)
*Rostrohypoxylonterebratum*	CBS 119137	DQ631943	DQ840069	DQ631954	DQ840097	Thailand (T)	Tang et al. (2007), Fournier et al. (2010b)
*Ruwenzoriapseudoannulata*	MUCL 51394	KY610406	KY610494	KY624286	KX271278	D. R. Congo (T)	Wendt et al. (2018)
*Thamnomycesdendroidea*	CBS 123578	FN428831	KY610467	KY624232	KY624313	French Guiana (T)	Stadler et al. (2010; ITS), Wendt et al. (2018; *tub2*, LSU, *rpb2*)
*Xylariaarbuscula*	CBS 126415	KY610394	KY610463	KY624287	KX271257	Germany	Fournier et al. (2011; ITS), Wendt et al. (2018; *tub2*, LSU, *rpb2*)
*Xylariahypoxylon*	CBS 122620	KY610407	KY610495	KY624231	KX271279	Sweden (ET)	Wendt et al. (2018)

### ﻿UHPLC profiling and dereplication

The secondary metabolites were extracted using a small piece of the stromata (approx. 1 mm^3^). Each piece was placed in 1.5 ml reaction tubes, covered with 1000 µl of methanol and placed for 30 min at 40 °C in an ultrasonic bath. The tubes were centrifuged at 14 000 rpm for 10 min. The methanol extract was separated from the remaining stromata, which was extracted again under the same procedure. Finally, both organic phases were combined and dried under nitrogen. Each sample was analyzed at a concentration of 450 µg/mL on an ultrahigh performance liquid chromatography system (Dionex Ultimate3000RS, Thermo Scientific, Dreieich, Germany), using a C18 column (Kinetex 1.7 µm, 2.1 × 150 mm, 100 Å; Phenomenex, Aschaffenburg, Germany) with a sample injection volume of 2 µL. The mobile phase consisted of A (H_2_O + 0.1% formic acid) and B (ACN + 0.1% formic acid) with a constant flow rate of 0.3 mL/min. The gradient began with 1% B for 0.5 min, increasing to 5% B in 1 min, then to 100% B in 19 min and holding at 100% B for 5 min. The temperature of the column was kept at 40 °C and UV-Vis data were recorded with a DAD at 190–600 nm.

MS spectra were collected using a trapped ion mobility quadrupole time-of-flight mass spectrometer (timsTOF Pro, Bruker Daltonics, Bremen, Germany) with the following parameters: tims ramp time 100 ms, spectra rate 9.52 Hz, PASEF on, cycle time 320 ms, MS/MS scans 2, scan range (*m/z*, 100–1800 Da; 1/k_0_, 0.55–2.0 V∙s/cm^2^). For the stromatal extracts and the standards ESI mass spectra were acquired in positive ion mode. Raw data were pre-processed with MetaboScape 2022 (Bruker Daltonics, Bremen, Germany) in the retention time range of 0.5 to 25 min. The obtained features were dereplicated against our in-house database comprising MS/MS spectra of standards from characteristic secondary metabolites of hypoxylaceous species (e.g. azaphilones, asterriquinones, binaphthalenes, cytochalasins, macrolides and sesquiterpenoids) in MetaboScape. A molecular network was created with the Feature-Based Molecular Networking (FBMN) (Nothias et al. 2020) and the Spec2Vec (Huber et al. 2021) workflows on the GNPS platform (Wang et al. 2016) using the pre-processed feature table from MetaboScape. Fragmentation ions resulting from the MS/MS spectra of cohaerin E, cohaerin H, and minutellin A were assigned using CFM-ID 4.0 web server (Wang et al. 2021) and validated with the SmartFormula 3D tool from MetaboScape. The datasets generated/analyzed for this study are included in Suppl. material 1.

## ﻿Results

### ﻿Phylogenetic analyses

**Figure 2.**
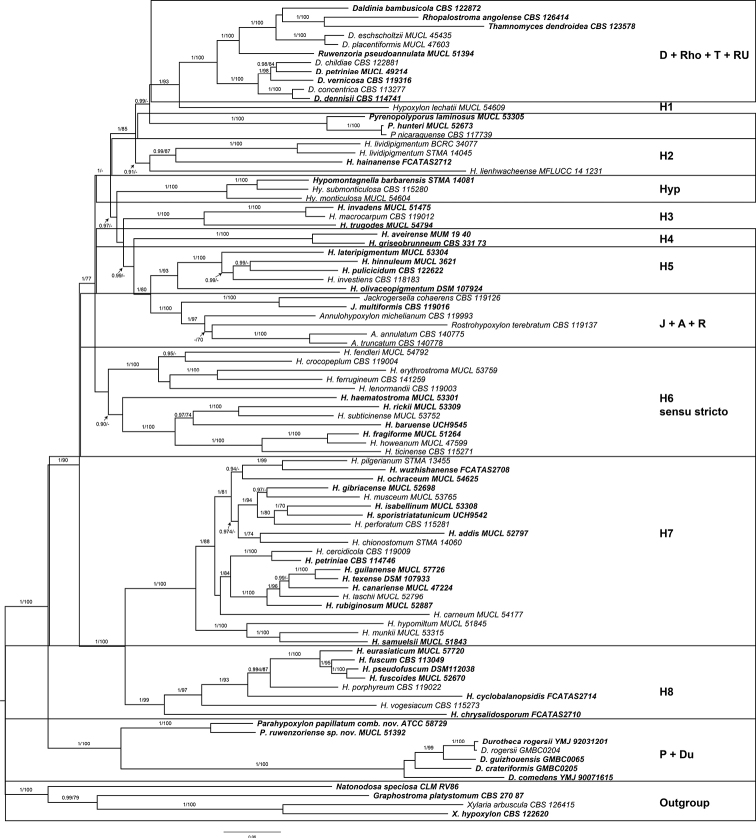
Inferred molecular phylogenetic maximum Likelihood (lLn = -122825.7921) tree of selected Hypoxylaceae, Graphostromataceae and Xylariaceae sequences. The analysis was calculated by using IQ-Tree with posterior probability support calculated from Bayesian inference methodology and support values generated from 1000 bootstrap replicates using a multigene alignment (ITS, LSU, *tub*2 and *rpb*2). The tree was rooted with *Xylariahypoxylon*CBS 122620, *X.arbuscula*CBS 126415 (Xylariaceae) and *Graphostromaplatystomum*CBS 27087 (Graphostromataceae). Type material is highlighted in **bold** letters. Bayesian posterior probability scores ≥ 0.95 / Bootstrap support values ≥ 70 are indicated along branches.

The final data matrix for the molecular phylogenetic analysis (Fig. 2) comprised 345 sequences (44 generated in this study, and complemented by sequences available from GenBank, NCBI) derived from 89 strains and four loci, namely ITS, LSU, *rpb2* and *tub2.* The final MAFFT alignments consisted of 4018 nucleotides for the ITS alignment, 3642 for the LSU alignment, 2238 for the *tub2* alignment and 4023 positions for the *rpb2* alignment. The alignment of each locus is available in the Suppl. material 1: table S3–S6. Sequences of representatives for each molecularly well-established genus of the Hypoxylaceae were included: *Annulohypoxylon* (3 strains), *Daldinia* (8 strains), *Durotheca* (5 strains), *Hypomontagnella* (3 strains), *Hypoxylon* (58 strains), *Jackrogersella* (2 strains), *Natonodosa* (1 strain), *Pyrenopolyporus* (3 strains), as well as *Rhopalostroma*, *Rostrohypoxylon*, *Ruwenzoria*, and *Thamnomyces* (1 strain each). Three members of Xylariaceae and Graphostromataceae (*Xylariahypoxylon*, *X.arbuscula* and *Graphostromaplatystomum*) served as outgroup.

The inference of phylogenetic relationship using a Maximum-Likelihood and Bayesian approach yielded two different, discongruent topologies. An approximate unbiased (AU) topology test implemented in IQTree indicated that the tree resulting from Bayesian inference received a significantly (p < 0.05) lower maximum likelihood score, suggesting its rejection. Hence, we included support values of the approximate Bayes test implemented in IQTree to access posterior probability support values of the inferred phylogenetic tree. The combined rooted phylogenetic tree showed a clade consisting of the core members of the Hypoxylaceae, such as *Hypoxylon*, *Daldinia*, *Pyrenopolyporus*, *Hypomontagnella*, *Jackrogersella*, *Rostrohypoxylon*, *Thamnomyces* and *Ruwenzoria* with medium BS and high PP support (1/90), which was placed in a sister position to a clade consisting of members of *Parahypoxylon* gen. nov., and *Durotheca* (Hypoxylaceae) at the base of the tree with strong support (1/100). The genus *Hypoxylon* could be confirmed as paraphyletic, as has been described already by Wendt et al. (2018), Lambert et al. (2019), and Becker et al. (2020). The sequences assigned to *Parahypoxylonruwenzoriense* formed a highly supported (1/100) cluster with the sequences derived from *Parahypoxylonpapillatum*. The topology of *Durotheca* and the newly described genus *Parahypoxylon* as a basal lineage in the Hypoxylaceae are further reflected upon in the taxonomic part of this study.

### ﻿Taxonomy

#### ﻿Lecto- and epitypification

##### ﻿ Hypomontagnella monticulosa

Taxon classificationFungiXylarialesHypoxylaceae

(Mont.) Sir, L. Wendt & C. Lamb.

712C8D76-2967-5CD1-9F1A-FD8D882E23B6

###### Type.

French Guiana, Cayenne, Leprieur, C. 1176, dead wood (PC, holotype; FH, isotype of *H.monticulosum*).

###### Epitype

**(designated here).** France. French Guyana, Sinnamary, Paracou, Amazonian rain forest, bark of unknown tree, June 2012, leg J. Fournier (LIP, ex-epitype culture MUCL 54604). GenBank acc. nos for DNA sequences: KY610404 and KJ810556 (ITS), KY610487 (LSU), KY624305 (*rpb*2), KX271273 (*tub2*); MT889334 (sporothriolide gene cluster published by Tian et al. 2020).

MBT no: 10010042.

###### Notes.

The strain designated here as epitype was used by Lambert et al. (2019) and the subsequent publications on genome analysis (Stadler et al. 2020; Tian et al. 2020; Kuhnert et al. 2021; Wibberg et al. 2021). The specimen and culture are perfectly suitable, because it was collected from the same geographic area as the holotype.

##### ﻿ Parahypoxylon

Taxon classificationFungiXylarialesHypoxylaceae

M. Cedeño-Sanchez, E. Charria-Girón & M. Stadler gen. nov.

9AC34E1D-9CE2-561D-905A-8A1D79258346

845463

###### Etymology.

Refers to the morphological similarity to *Hypoxylon*, from which the genus is phylogenetically distinct.

###### Diagnosis.

Differs from the genus *Durotheca* by the presence of greenish KOH-extractable pigments and by having an amyloid ascal apical apparatus. Differs from the genus *Hypoxylon* by containing yet unknown cohaerin-type azaphilones and by its basal position in the molecular phylogenetic inference using am ITS, LSU, *rpb2* and *tub2* matrix.

##### ﻿ Parahypoxylon papillatum

Taxon classificationFungiXylarialesHypoxylaceae

(Ellis & Everh) M. Cedeño-Sanchez, E. Charria-Girón & M. Stadler comb. nov.

50AFC28B-1ADC-57E2-A321-8DDDF3C465B9

845462

Figs 3 , 4

Hypoxylon papillatum Ellis & Everh. in Smith, Bull. Lab. Nat. Hist. Iowa State Univ. 2: 408 (1893). Syn.

###### Lectotype.

USA. Ohio, Delaware, 21 Jul 1893, A. Commons 2160, rotten wood of *Carya* (NY [2 pks.], selected by Ju and Rogers (1996).

###### Epitype.

USA. West Virginia, Mason Co., Bruce’s Chapel, 18 Aug 1983, wood of *Acer*, J.D. Rogers (WSP 7557; ex-epitype culture ATCC 58729).

MBT no: 10011515.

###### Teleomorph.

Stromata superficial, effused-pulvinate to plane, with inconspicuous to conspicuous perithecial mounds, up to 12.5 cm long × up to 4 cm broad × 1.8–4.0 mm thick; surface Honey (64) to Isabelline (65), Isabelline (65) to Gray Olivaceous (107), or Isabelline (65) to Olivaceous (48); blackish granules immediately beneath surface and between perithecia, with KOH-extractable pigments Isabelline (65); the tissue below the perithecial layer conspicuous, black, 1.0–2.5 mm thick. Perithecia long-tubular, 0.3–0.4 mm diam × 0.8–1.5 mm high. Ostioles umbilicate. Asci with amyloid, discoid apical apparatus, 1–2 µm high × 3.5 µm wide, stipe up 137–180 µm long × 8–10 µm broad, the spore-bearing parts 93–110 µm long, the stipes 30–80 µm long. Ascospores brown to dark brown, unicellular, ellipsoid, nearly equilateral, with broadly to narrowly rounded ends, 12.0–18.5 × 6.5–9.0 µm, with straight germ slit spore-length; perispore indehiscent in 10% KOH; epispore smooth.

**Figure 3.**
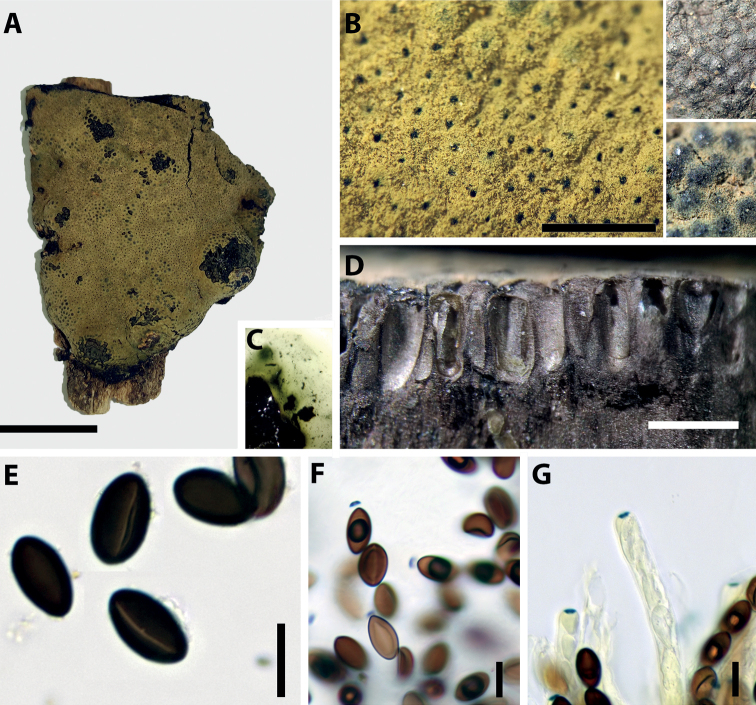
*Parahypoxylonpapillatum* comb. nov. **A** stroma **B** ostioles **C** KOH extractable stromatal pigments **D** perithecia (cross section) **E** ascospores with straight germ slits **F** amyloid apical apparatus in a mature ascus treated with Melzer’s reagent **G** amyloid apical apparatus in an immature ascus treated with Melzer’s reagent. Scale bars: 1 cm (**A**); 10 μm (**E–F**); 10 μm (**G**).

**Figure 4.**
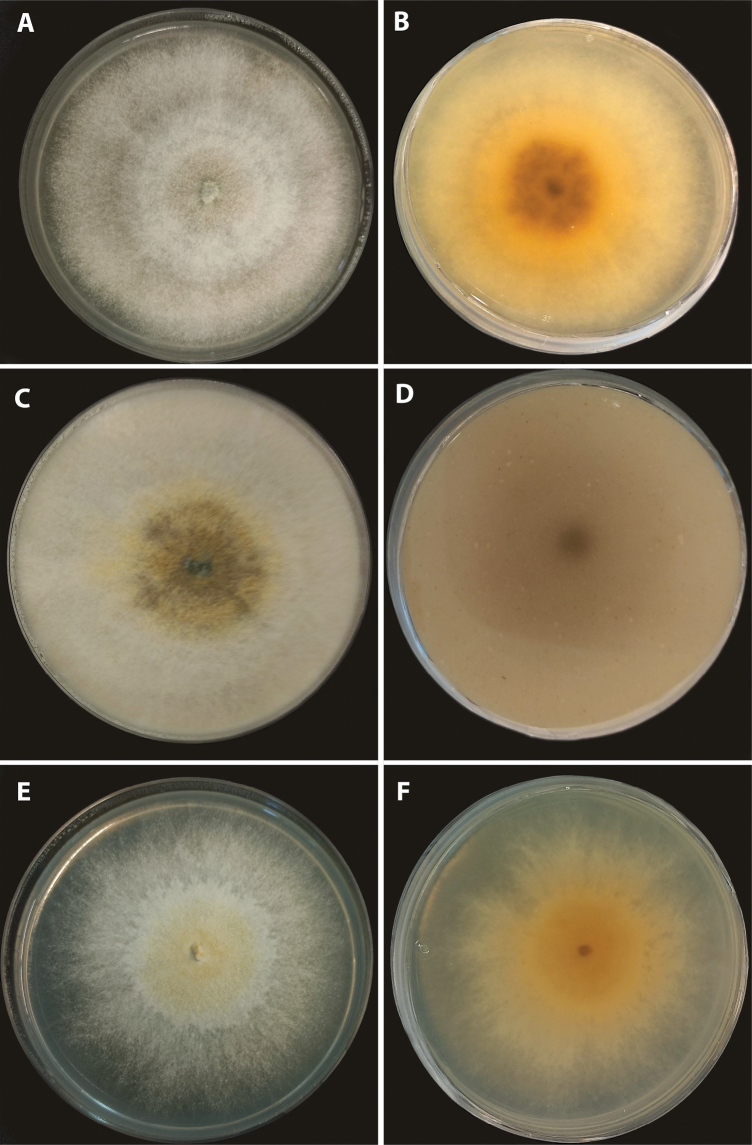
*Parahypoxylonpapillatum* comb. nov. (ATCC 58729) Colonies after 2 weeks (**A, B**) on 2% MEA (**C, D**) on OA (**E, F**) on YM.

###### Cultures and anamorph.

Colonies on MEA, OA, and YM covering a 9 cm Petri plate in 2 weeks, with white, flat, mycelium, margins filamentous. Reverse at first white, becoming yellowish at the center. The anamorph has been described by Rogers (1985), but we were unable to confirm the presence of conidial structures when we studied the strain more than 30 years later.

###### Secondary metabolites.

Stromata contain BNT and cohaerin type azaphilones according to the MS/MS analysis.

###### Notes.

We were not only able to confirm the morphometric results of Ju and Rogers (1996) but even established that this species is characterized by a rather specific metabolite profile. This species has to our knowledge still not been reported from outside America and seems to be most frequently encountered in the Eastern USA.

###### Further specimens examined.

USA. Kansas, on decorticated wood, Feb 1884, F.W. Cragin 257 (NY00830462, syntype of *H.papillatum*); Pennsylvania, Allegheny Co., on dead wood, 14 Aug 1941, Henry, L.K. 4885 (BPI 591033); Pennsylvania, Meadville, old log, 17 Oct 1922, E.C. Smith 353 (BPI 591030); CANADA., on wood, J. Dearness (BPI 591035A, syntype of *H.papillatum*).

##### ﻿ Parahypoxylon ruwenzoriense

Taxon classificationFungiXylarialesHypoxylaceae

M. Cedeño-Sanchez, E. Charria-Girón & M. Stadler sp. nov.

782D5F9A-F76C-5699-8F23-E41D84227369

845457

Figs 5 , 6

###### Holotype.

Democratic Republic Of The Congo. North Kivu: Mt. Ruwenzori, about 00°33.961'N, 29°81.795'E, between 2,138 and 2,400 m alt., 3–5 Feb 2008, tropical mountain forest, C. Decock (MUCL 51392, ex-holotype culture MUCL 51392).

###### Etymology.

Named after the Ruwenzori Mountains, where the species was collected.

###### Teleomorph.

Stromata superficial, incomplete, effused-pulvinate, 60 mm long × 40 mm broad × 3–5 mm thick; surface Fawn (87), with inconspicuous perithecial mounds, with a black, shiny hard crust 100–150 µm thick above perithecia, without visible granules, with KOH-extractable pigments Hazel (88); the pruina hyphae turn violet in KOH; the tissue below the perithecia 2–4 mm thick, vertically fibrose, dark grey. Perithecia tubular, 0.90–1.50 mm high × 0.2–0.3 mm diam (n=18). Ostioles umbilicate, surrounded by a white substance. Asci cylindrical, 8-spored, the spore-bearing parts 82–105 µm long × 5.5–6.0 µm broad, the stipes 38–130 µm long, with amyloid, discoid apical ring 0.7–2.0 µm high × 2.5–3.5 µm (n=21) broad. Ascospores smooth, unicellular, brown to dark brown, narrowly ellipsoid, nearly equilateral with narrowly rounded ends, 10.5–13.8 × 4.0–5.6 µm (n=40), with a faint, straight germ slit; perispore indehiscent in 10% KOH.

**Figure 5.**
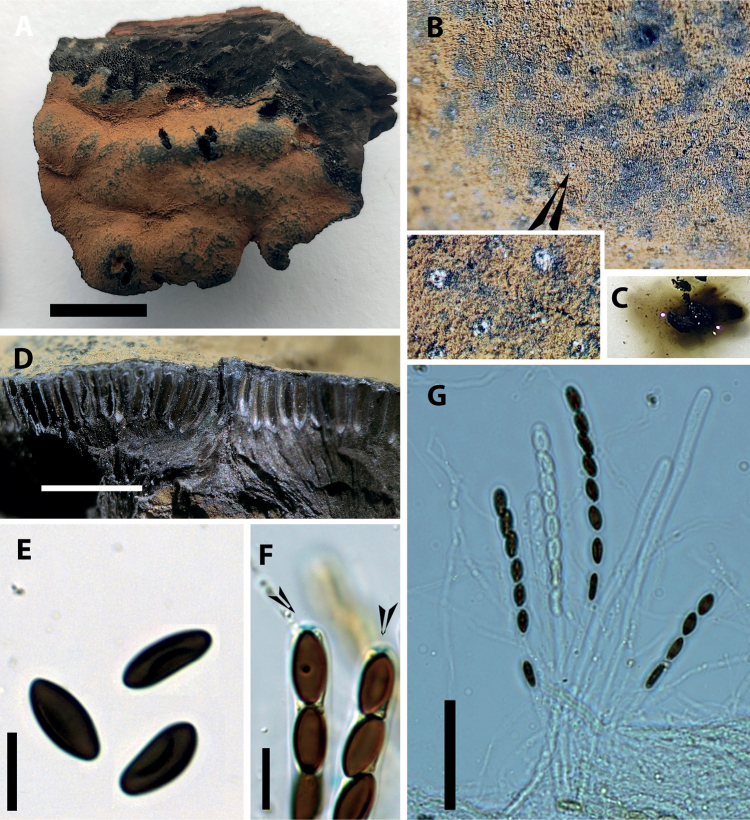
*Parahypoxylonruwenzoriense* sp. nov. (MUCL 51392). **A** stroma **B** ostioles with white ring **C** KOH extractable stromatal pigments **D** perithecia (cross section) **E** ascospores **F** amyloid apical apparatus (blueing in Melzer’s reagent) indicated by arrowheads **G** asci. Scale bars: 1 cm (**A**); 2 mm (**D**); 10 μm (**E, F**); 50 μm (**G**).

**Figure 6.**
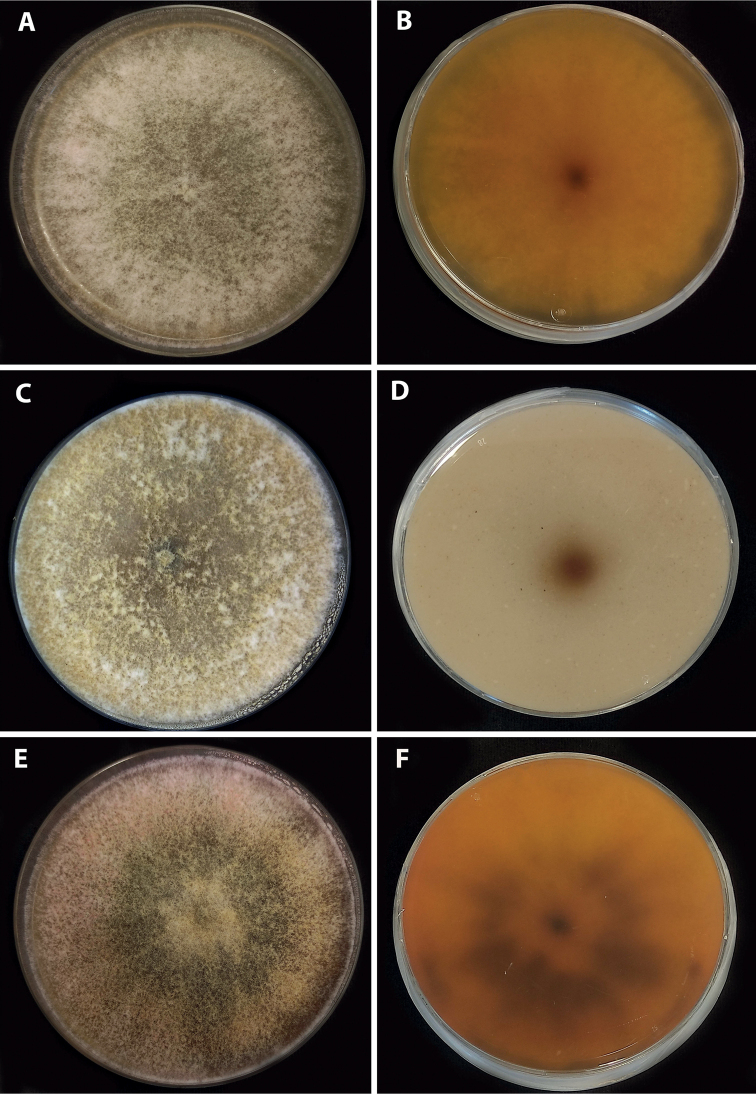
*Parahypoxylonruwenzoriense* sp. nov. (MUCL 51392) Colonies after 2 weeks (**A, B**) on 2% MEA (**C, D**) on OA (**E, F**) on YM.

###### Cultures and anamorph.

Colonies on MEA, OA, and YM covering a 9 cm Petri plate in 2 weeks, with mycelium white at first, flat to raised in some zones, to becoming greenish in the center. Reverse at first yellowish, to become orange with a black spot at the center. Conidiophores not produced.

###### Secondary metabolites.

Stromata contain BNT and cohaerin type azaphilones according to the MS/MS analysis.

###### Notes.

*P.ruwenzoriense* is phylogenetically close to *P.papillatum* but differs by its KOH-extractable pigments Hazel (88) and by smaller ascospores.

### ﻿Metabolomic profiling of stromata

As explained in the Experimental section, stromata of five herbarium specimens assignable to *Parahypoxylon* were extracted and analysed by UHPLC-DAD-IM-MS/MS. The raw data sets were pre-processed and the obtained feature table dereplicated using high resolution *m/z*, MS/MS spectra, retention time, CCS value, and UV/Vis spectra and reference data obtained from our in-house library of common secondary metabolites of the Hypoxylaceae (data not shown).

**Figure 7.**
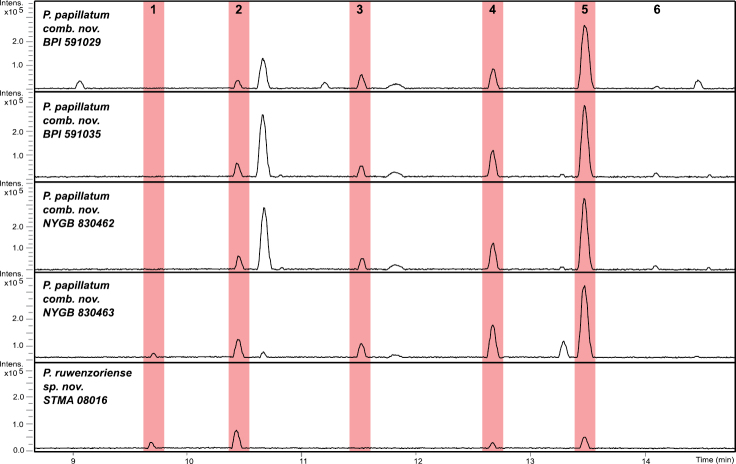
Base peak chromatograms (BPCs) from UHPLC-MS analysis of the stromatal extracts of *P.papillatum* (BPI 591029), *P.papillatum* (BPI 591035), *P.papillatum* (NYGB 830462), *P.papillatum* (NYGB 830463), and *Parahypoxylonruwenzoriense* sp. nov. (STMA 08016). Compounds common between several species (numbered **1**–**6**) are highlighted in red.

**Figure 8.**
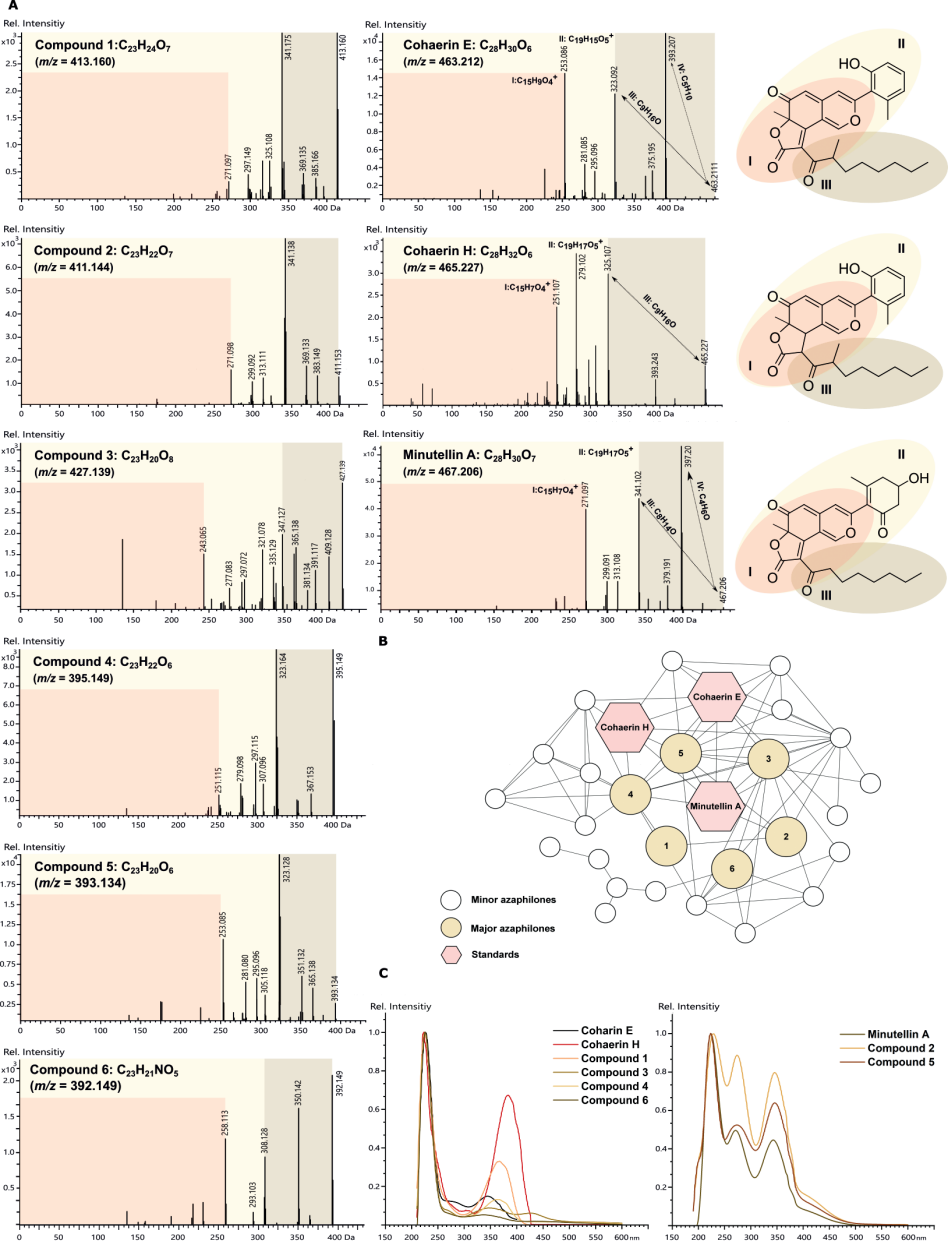
**A** Reference MS/MS spectra of cohaerin E, cohaerin H, and minutellin A standards, and the six major azaphilones identified in the UHPLC-MS chromatograms of stromatal extracts from the *Parahypoxylon* spp. **B** azaphilone cluster in a molecular network created from the *Parahypoxylon* spp. stromatal extracts and MS/MS spectra from selected standards **C** UV/Vis profile comparison from compound **1**–**6**, cohaerin E, cohaerin H, and minutellin A.

From the base peak chromatograms (BPC) of the stromatal extracts of the studied specimens, six major peaks could be distinguished (Fig. 7). An additional MS/MS similarity search without matching the precursor mass against our in-house library in MetaboScape yielded a MS/MS score > 700 for compounds **2** and **5** when compared with cohaerin E, cohaerin H, and minutellin A standards, which were not contained in the stromatal extracts (Suppl. material 1: fig. S2). This tentatively advocated a structural relation to the azaphilone family (Fig. 8a). Molecular formulae for compounds **1**–**6** were predicted as C_23_H_24_O_7_, C_23_H_22_O_7_, C_23_H_20_O_8_, C_23_H_22_O_6_, C_23_H_20_O_6_, and C_23_H_21_NO_5_ (Suppl. material 1: table S7), with a lower number of carbons than cohaerin E (C_28_H_30_O_6_), cohaerin H (C_28_H_32_O_6_), and minutellin A (C_28_H_30_O_7_). To further validate the presence of cohaerin E-like azaphilones in the stromatal extracts of the *Parahypoxylon* spp. a molecular networking (MN) approach was pursued. The above mentioned tool can be employed to organize in an automatic basis MS/MS spectra into groups based on similarities in their fragmentation patterns and the hypothesis that structurally related molecules will yield similar MS/MS spectra (Duncan et al. 2015). For this analysis, we compared the MS/MS spectra of cohaerin E, cohaerin H, and minutellin A (Suppl. material 1: table S7, fig. S2) with all MS/MS spectra obtained from the *Parahypoxylon* gen. nov. stromatal extracts by means of the unsupervised machine learning approach Spec2Vec. As a result, the molecular cluster containing the cohaerin standards consisted of 29 consensus spectra (nodes), which included compounds **1**–**6** (Fig. 8b). In addition, cohaerin E and H have UV/Vis absorptions at λ_max_ 226–223 and 344–380 nm, which are resembling UV/Vis absorptions from compounds **1**, **3**, **4**, and **6**. Minutellin A displayed UV/Vis absorptions at λ_max_ 224, 271, and 343 nm, a pattern identified also for compounds **2** and **5** (Fig. 8c).

**Figure 9.**
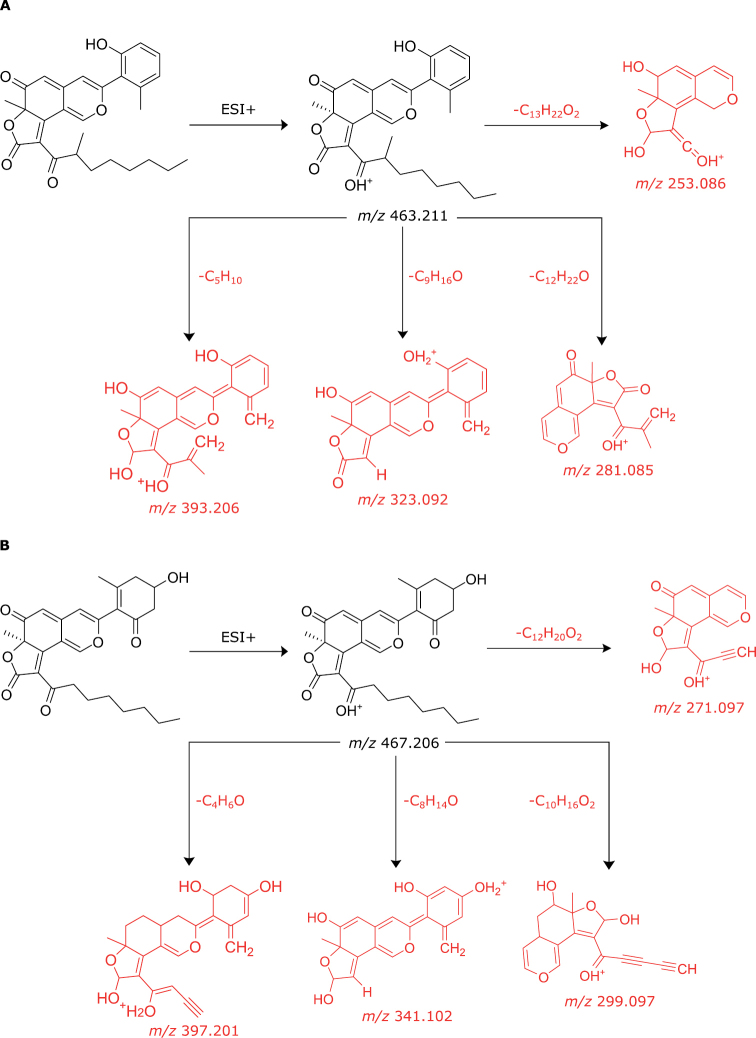
**A** most abundant fragment ions observed in MS/MS spectra for cohaerin E and associated structures as predicted by CFM-ID 4.0 **B** most abundant fragment ions observed in MS/MS spectrum for minutellin A and associated structures as predicted by CFM-ID 4.0.

Cohaerin-type azaphilones present as well a distinct MS fragmentation pattern. In MS/MS experiments, cohaerin E generated fragment ions at 393.207 Da, 323.092 Da, 281.085 Da, and 253.086 Da, while minutellin A generated fragment ions at 397.201 Da, 341.102 Da, 299.091 Da, and 271.097 Da. The most abundant fragments were annotated using the CFM-ID 4.0 peak assignment module. In both cases, the most abundant fragments were traced down to the azaphilone backbone (Fig. 9). For instance, the mass difference of 18 Da between 323.092 Da and 341.102 Da could be interpreted as H_2_O, reflecting the different substitution of the 3-methylphenol moiety. Fragment ions at 281.085 Da and 253.086 Da for cohaerin E represent the tricyclic portion of the molecule, while fragment ions at 299.091 Da and 271.097 Da represent the same part of the molecule in minutellin A. Analogously, MS fragmentation patterns for cohaerin H (Fig. 8) resembles the generated fragments for cohaerin E. As some typical cohaerin-type azaphilones fragmentation patterns were conserved, we assume that the changes found for the stromatal metabolites of **1**–**6** occur in the side chain of the molecules. In summary, the UHPLC-DAD-IM-MS/MS and UV/Vis data, combined with a comparison of molecular networking analyses, indicated the presence of novel azaphilones related to the cohaerin family in the stromatal extracts from the *Parahypoxylon* spp., in contrast to the absence of other common secondary metabolites of the Hypoxylaceae.

## ﻿Discussion

The genus *Hypoxylon* in the current taxonomic concept has frequently been shown to be paraphyletic (Wendt et al. 2018; Lambert et al. 2019), which has once more been confirmed in this study, foreshadowing again future rearrangements for a thorough revision of its systematics. This is especially apparent because the type species *H.fragiforme* forms a relatively small clade clustering with a small subset of closely related taxa. Therefore, further segregation will eventually be unavoidable once more data to safely delineate the different lineages becomes available. Here, we gathered chemotaxonomic, morphological and sequence data to enable a polyphasic characterization of a basal clade formerly phylogenetically resolved inside *Hypoxylon*, containing specimen closely related to *H.papillatum*, for which we propose the erection of the new genus *Parahypoxylon*, sharing many salient features with *Hypoxylon* in the “traditional” definition.

The investigation of the stromatal metabolite extracts by HPLC has proven to be a valuable resource to achieve a more natural classification of hypoxylaceous taxa (Kuhnert et al. 2015; Wendt et al. 2018; Lambert et al. 2019). Recent advances in the analytics for in-depth characterization of natural products, mainly driven by metabolomics-based strategies, have enabled a better understanding of complex natural systems (Van der Hooft et al. 2020). The current MS-based techniques can help as a predictor for the discovery of new carbon skeletons to help and prioritize their isolation and description instead of the isolation of new derivatives of already known metabolite scaffolds. Nevertheless, relying mainly on MS/MS fragmentation spectra could lead to an underestimation of chemical diversity. The complex chemical space produced by a single BGC may result in completely different fragmentation patterns only by the addition of small structural changes (McCaughey et al. 2022). Still, a general methodology for characterizing and classifying structural analogs with a common biosynthetic origin is absent particularly in the field of fungal natural products (Almeida et al. 2020).

However, in many occasions and applications, the isolation and structure elucidation of yet unidentified compounds is not possible, such as in the example of isolating pigments from natural sources, as is the case in the genus *Hypoxylon*. Even very old specimens have been reported to harbor intact secondary metabolites, as has been described for fossilized stromata assigned to *Hypoxylonfragiforme* in a study of archeological samples by Surup et al. (2018). Here, fortunately the original species could be recollected in German woods, but for rarer specimens, or specimens only producing scarce amounts of stromata, this is not a practicable option. Instability of the contained metabolites during e.g. purification further complicates the issue (Stadler et al. 2008; Kuhnert et al. 2014b; Sir et al. 2019). In this study, we demonstrated the value of integrating metabolomics-based tools to characterize the secondary metabolite profile of the type and authentic specimens of *P.papillatum* and the new species from the D.R. Congo.

An MS/MS analysis of the major metabolites suggested the presence of six unknown compounds assignable to the azaphilones related to the cohaerin family, which have been predicted to harbor a smaller carbon skeleton than the known cohaerins, and which still conserve some of the distinctive fragmentation patterns of these secondary metabolites (Suppl. material 1: fig. S3). This phenomenon has been exemplified within the Hypoxylaceae, which present a highly diverse group of PKS-derived pigments, among which the different subfamilies present different attached side chains at the C-8 oxygen (Kuhnert et al. 2021). The above findings suggest that the type of azaphilone produced by the studied species belong to a different type of azaphilones with a shorter side chain, but with a shared backbone in comparison to the cohaerins and minutellins. Additionally, the number of nodes found in the MN analysis suggests that the chemical diversity of the azaphilones produced by the strains belonging to *Parahypoxylon* gen. nov. is much higher than thought. In general, following a similar approach, the MolNetEnhancer workflow allowed the characterization of triterpenoid metabolites with several distinct phenolic acid modifications (e.g., vanillate, protocatechuate) in a different taxonomic background in the plant family Rhamnaceae (Ernst et al. 2019). The same methodology enabled the annotation of molecular families with known chemical motifs previously unreported for *Salinispora*, *Streptomyces*, and *Xenorhabdus* bacterial extracts (Ernst et al. 2019). Even though the ideal scenario would remain to isolate and elucidate the structures of the secondary metabolites, these tools are a powerful resource to classify chemical structural annotation and enhance our understanding of chemodiversity by adding biological and chemical insights of complex metabolic mixtures. It is worth noting that the stromatal material could eventually become available in the future from forthcoming collection campaigns, and therefore the aforementioned hypothesis might be confirmed through isolation and chemical characterization of the major metabolites.

In this context, the stromatal metabolite profile of the specimens of *P.papillatum* and the new species *P.ruwenzoriense* are rather unique, even though it exhibits related chemotaxonomic features more likely to be found in the Hypoxylaceae. The cohaerin type azaphilones (which include also the multiformins and minutellins) have first been reported by Quang et al. (2005a, b, 2006), Surup et al. (2013) and Kuhnert et al. (2017b) and were recently found to possess interesting antiviral effects (Jansen-Olliges et al. 2023). Their producers are now all classified in *Jackrogersella* (Wendt et al. 2018) and were formerly placed in Hypoxylonsect.Annulata or (Ju and Rogers 1996) *Annulohypoxylon* (Hsieh et al. 2005), respectively. Kuhnert et al. (2017a) already reported that the species of *Annulohypoxylon* are divided into two chemotypes, one of which is characterized by stromata with papillate ostioles and cohaerin type azaphilones. The other chemotype is devoid of these compounds and produces binaphthalenes as prevailing stromatal metabolites. It includes *A.truncatum*, the type species of *Annulohypoxylon*, and many other species that feature ostiolar discs. Since this coincided with the molecular phylogeny by Wendt et al. (2018), the new genus *Jackrogersella* was erected for the cohaerin-containing species with papillate, diskless ostioles. There is only one species in *Annulohypoxylon* (i.e., *A.michelianum*) that has such ostiolar rings and also produces cohaerins. It was left at interim in *Annulohypoxylon*, even though its DNA sequence occupied a separate clade in the phylogeny by Wendt et al. (2018). The reason is that the strain studied did not constitute type material, and we felt that the erection of a separate genus should only be carried out by including fresh material from the geographic area and host (*Laurus* in South Europe) from which the holotype specimen was reported. Aside from the above-mentioned fungi, metabolites with cohaerin-like characteristics (i.e. characteristic mass and diode array spectra) have even been detected in species of *Hypoxylon*, such as *H.pulicicidum* (Bills et al. 2012). A recent study based on the analysis of full genomes based on 3^rd^ generation sequencing techniques, such as PacBio and Oxford nanopore (Wibberg et al. 2021), has even revealed the corresponding biosynthetic gene clusters encoding for these azaphilone pigments to be present in the studied *Jackrogersella* species and *H.pulicicidum* (Kuhnert et al. 2021). For instance, the identified BGC in *H.pulicicidum* carries the core set of conserved genes for this family of azaphilones, but the additional presence of additional tailoring enzymes indicates that the produced metabolites might have different structural features compared to the known cohaerins (Kuhnert et al. 2021).

In the future, it will become easier to tell if the genetic information for the successful biosynthesis of such secondary metabolites is present in the genomes of the respective organisms even if the products cannot be detected. Chemotaxonomic evidence can also be used to segregate the new genus from the species that are located in neighboring basal clades in the current phylogeny (i.e., *Hypoxylonaeruginosum* and *Durotheca* spp.). Interestingly, these species neither contain azaphilones nor binaphthalenes, with *H.aeruginosum* and the related genus *Chlorostroma* reported to have lepraric acid derivatives as major stromatal metabolites (Læssøe et al. 2010), which are otherwise unique and only occur in some lichenized ascomycetes. *Durotheca*, on the other hand, appears to be poor in stromatal metabolites, and Læssøe et al. (2013) only found traces of lepraric acids in one of the species they studied. The current study has further confirmed the results by de Long et al. (2019), who found that *Durotheca* is a hypoxylaceous genus, even though its species have a distinctive ascospore morphology and other secondary metabolites.

The integration of state-of-the-art metabolomic-based tools in chemotaxonomic surveys will further accelerate and assist the systematic study of paraphyletic taxa within the concept of polyphasic taxonomy as herein demonstrated for the introduction of *Parahypoxylon*.

## Supplementary Material

XML Treatment for Hypomontagnella monticulosa

XML Treatment for Parahypoxylon

XML Treatment for Parahypoxylon papillatum

XML Treatment for Parahypoxylon ruwenzoriense

## Supplementary materials

Supplementary material 1 Supplementary information Data type Alignments and MS raw data (PDF file) https://binary.pensoft.net/file/813203This dataset is made available under the Open Database License (http://opendatacommons.org/licenses/odbl/1.0/). The Open Database License (ODbL) is a license agreement intended to allow users to freely share, modify, and use this Dataset while maintaining this same freedom for others, provided that the original source and author(s) are credited.Marjorie Cedeño-Sanchez, Esteban Charria-Girón, Christopher Lambert, J. Jennifer Luangsa-ard, Cony Decock, Raimo Franke, Mark Brönstrup, Marc Stadler
