# Supplementary material for: ﻿Segregation of the genus Parahypoxylon (Hypoxylaceae, Xylariales) from Hypoxylon by a polyphasic taxonomic approach
Source: MycoKeys. 2023 Feb 20;95:131–62. doi: 10.3897/mycokeys.95.98125 (PMC10210297; doi:10.3897/mycokeys.95.98125)
Supplement: Supplementary material 1 — Supplementary information [file mycokeys-95-131-s001.pdf]

Supplementary Information for

Segregation of the genus *Parahypoxylon* (Hypoxylaceae) from *Hypoxylon* by a polyphasic taxonomic approach

Marjorie Cedeño-Sanchez[1,2,†], Esteban Charria-Girón[1,2,†], Christopher Lambert[1,2,3], J. Jennifer Luangsa-ard[4], Cony Decock[5], Raimo Franke[6], Mark Brönstrup[6], and Marc Stadler[1,2,*]

[1]Helmholtz-Zentrum für Infektionsforschung GmbH, Dept. Microbial Drugs, Inhoffenstrasse 7, 38124 Braunschweig, Germany

[2]Institute of Microbiology, Technische Universität Braunschweig, Spielmannstraße 7, 38106 Braunschweig, Germany

[3]Division of Molecular Cell Biology, Zoological Institute, Technische Universität Braunschweig, Spielmannstrasse 7, 38106 Braunschweig, Germany

[4]National Center for Genetic Engineering and Biotechnology (BIOTEC), 113 Thailand Science Park, Phaholyothin Road, Klong Luang, Pathumthani 12120, Thailand

[5]Mycothéque de l' Universite catholique de Louvain (BCCM/MUCL), Place Croix du Sud 3, B-1348 Louvain-la-Neuve, Belgium

[6]Department Chemical Biology, Helmholtz Centre for Infection Research GmbH (HZI) Partner site Hannover/Braunschweig, Inhoffenstrasse 7, 38124 Braunschweig, Germany

*Corresponding author: Marc Stadler, e-mail: marc.stadler@helmholtz-hzi.de, phone: +49 531 6181-4240, fax: 49 531 6181-9499

† Author contributed equally

Content

Table S1. Selected edge-linked proportional partition substitution models subjected to IQTree2 calculated with ModelFinder using Bayesian information criterion (BIC). 3

Table S2. Characteristics of the MAFFT alignments following the phylogenetic analysis using IQTree2 for phylogenetic inference. 3

Figure S1. Inferred molecular phylogenetic tree of *Hypoxylon* calculated by using MrBayes from a multigene alignment (ITS, LSU, tub2 and rpb2). The tree was rooted with *Xylaria hypoxylon* CBS 122620, *X. arbuscula* CBS 126415 (Xylariaceae) and *Graphostroma platystomum* CBS 27087 (Graphostromataceae). Type material is highlighted in bold letters. Bayesian posterior probability scores ≥ 0.95.. 5

Table S3. Alignment of the ITS sequences used in the phylogenetic study. 5

Table S4. Alignment of the LSU sequences used in the phylogenetic study. 104

Table S5. Alignment of the rpb2 sequences used in the phylogenetic study................................ 201

Table S6. Alignment of the tub2 sequences used in the phylogenetic study................................ 282

Figure S2. Extracted ion chromatogram of the stromatal metabolites **1**–**6** from the *Parahypoxylon* spp. and standards of minutellin A and cohaerin E. 347

Table S7. Dereplicated metabolites from the stromatal extracts of the *Parahypoxylon* spp and in-house standards. 348

Figure S3. Observed different carbon skeletons for the known cohaerin type azaphilones in the Hypoxylaceae. 349

Table S1. Selected edge-linked proportional partition substitution models subjected to IQTree2 calculated with ModelFinder using Bayesian information criterion (BIC).

DNA Locus	Model	Speed	Parameters
ITS	TIM2e+R5	1.0022	TIM2e{1.50442,2.74232,3.80361}+FQ+R5{0.416992,0.0250716,0.169307,0.36117,0.159166,1.01994,0.178643,2.40544,0.0758909,4.4319}
LSU	TIM2e+R5	1.0060	TIM2e{1.45777,3.71794,6.6591}+FQ+R5{0.577914,0.0119461,0.147175,0.17454,0.0732767,0.690252,0.123724,1.74119,0.0779107,9.00263}
RPB2	TIM3+F+R5	0.9273	TIM3{1.36981,5.43247,9.51658}+F{0.253438,0.261415,0.260778,0.22437}+R5{0.429023,0.00866679,0.139458,0.248022,0.126929,0.966577,0.195877,2.00835,0.108713,4.09903}
tub2	GTR+F+R5	1.1170	GTR{1.00706,4.17322,1.38695,0.759934,5.0497}+F{0.220539,0.305779,0.231102,0.24258}+R5{0.39862,0.00681493,0.12775,0.284271,0.189545,0.997931,0.206761,2.15047,0.0773247,4.23127}

Table S2. Characteristics of the MAFFT alignments following the phylogenetic analysis using IQTree2 for phylogenetic inference.

DNA Locus	Sequences	Sites	Unique	Informative	Invariant	Constant
ITS	90	4018	972	496	3226	3226
LSU	84	3642	1312	434	2551	2551
RPB2	85	4023	947	665	2649	2649
TUB2	89	2238	1412	891	1023	1023

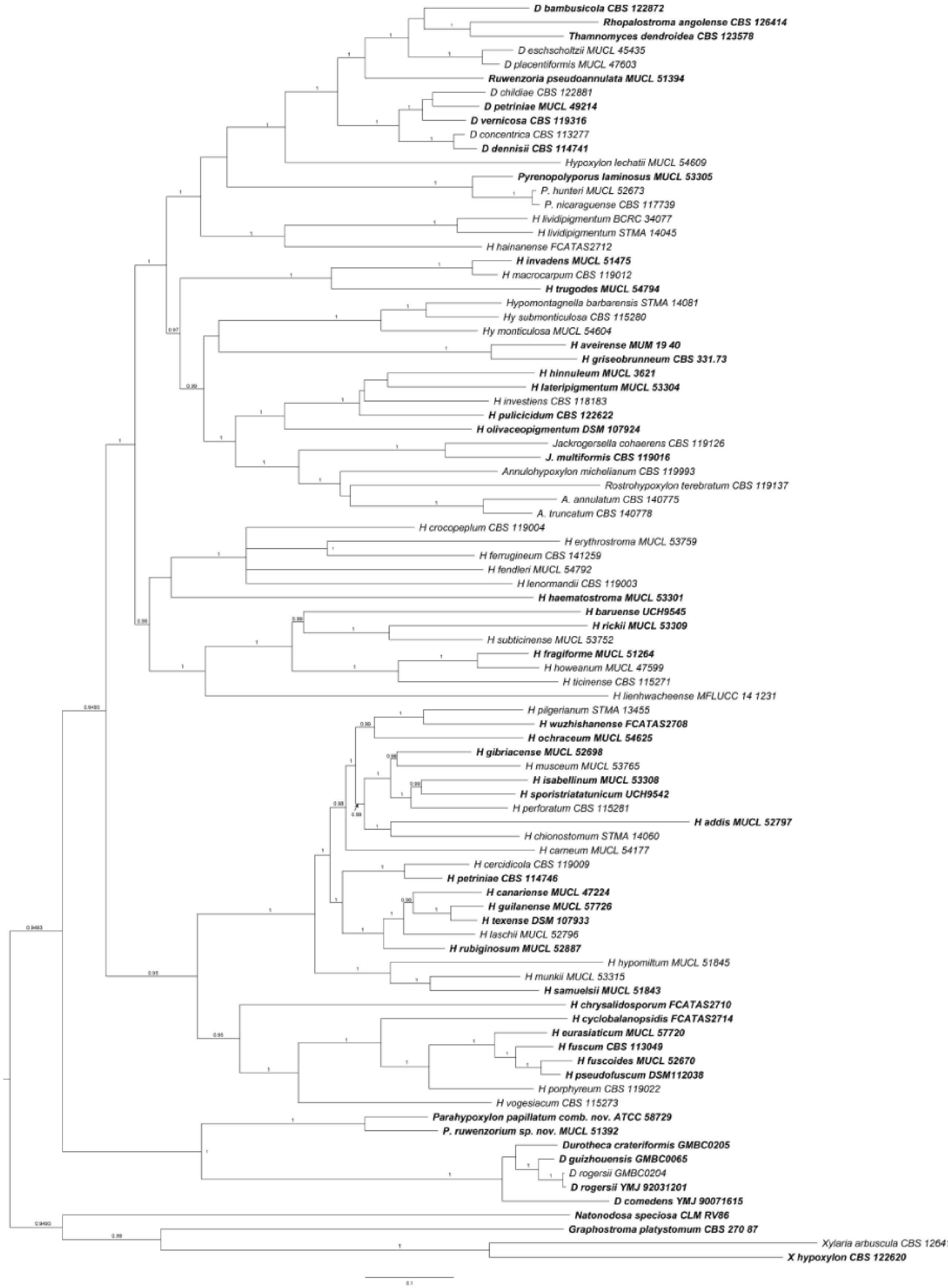

D bambusicola CBS 122872
Rhopalostroma angolense CBS 126414
Thamnomyces dendroidea CBS 123578
D eschscholtzii MUCL 45435
D placentiformis MUCL 47603
Ruwenzoria pseudoannulata MUCL 51394
D childiae CBS 122881
D petriniae MUCL 49214
D vernicosa CBS 119316
D concentrica CBS 113277
D dennisii CBS 114741
Hypoxylon lechatii MUCL 54609
Pyrenopolyporus laminosus MUCL 53305
P. hunteri MUCL 52673
P. nicaraguense CBS 117739
H lividipigmentum BCRC 34077
H lividipigmentum STMA 14045
H hainanense FCATAS2712
H invadens MUCL 51475
H macrocarpum CBS 119012
H trugodes MUCL 54794
Hypomontagnella barbarensis STMA 14081
Hy submonticulosa CBS 115280
Hy monticulosa MUCL 54604
H aveirense MUM 19 40
H griseobrunneum CBS 331.73
H hinnuleum MUCL 3621
H lateripigmentum MUCL 53304
H investiens CBS 118183
H pulicicidum CBS 122622
H olivaceopigmentum DSM 107924
Jackrogersella cohaerens CBS 119126
J. multiformis CBS 119016
Annulohypoxylon michelianum CBS 119993
Rostrohypoxylon terebratum CBS 119137
A. annulatum CBS 140775
A. truncatum CBS 140778
H crocopeplum CBS 119004
H erythrostroma MUCL 53759
H ferrugineum CBS 141259
H fendleri MUCL 54792
H lenormandii CBS 119003
H haematostroma MUCL 53301
H baruense UCH9545
H rickii MUCL 53309
H subticinense MUCL 53752
H fragiforme MUCL 51264
H howeanum MUCL 47599
H ticinense CBS 115271
H lienhwacheense MFLUCC 14 1231
H pilgerianum STMA 13455
H wuzhishanense FCATAS2708
H ochraceum MUCL 54625
H gibriacense MUCL 52698
H musceum MUCL 53765
H isabellinum MUCL 53308
H sporistriatatunicum UCH9542
H perforatum CBS 115281
H addis MUCL 52797
H chionostomum STMA 14060
H carneum MUCL 54177
H cercidicola CBS 119009
H petriniae CBS 114746
H canariense MUCL 47224
H guilanense MUCL 57726
H texense DSM 107933
H laschii MUCL 52796
H rubiginosum MUCL 52887
H hypomiltum MUCL 51845
H munkii MUCL 53315
H samuelsii MUCL 51843
H chrysalidosporum FCATAS2710
H cyclobalanopsidis FCATAS2714
H eurasiaticum MUCL 57720
H fuscum CBS 113049
H fuscoides MUCL 52670
H pseudofuscum DSM112038
H porphyreum CBS 119022
H vogesiacum CBS 115273
Parahypoxylon papillatum comb. nov. ATCC 58729
P. ruwenzorium sp. nov. MUCL 51392
Durotheca crateriformis GMBC0205
D guizhouensis GMBC0065
D rogersii GMBC0204
D rogersii YMJ 92031201
D comedens YMJ 90071615
Natonodosa speciosa CLM RV86
Graphostroma platystomum CBS 270 87
Xylaria arbuscula CBS 126415
X hypoxylon CBS 122620

Figure S1. Inferred molecular phylogenetic tree of Hypoxylon calculated by using MrBayes from a multigene alignment (ITS, LSU, *tub*2 and *rpb*2). The tree was rooted with *Xylaria hypoxylon* CBS 122620, *X. arbuscula* CBS 126415 (Xylariaceae) and *Graphostroma platystomum* CBS 27087 (Graphostromataceae). Type material is highlighted in bold letters. Bayesian posterior probability scores ≥ 0.95.

Table S3. Alignment of the ITS sequences used in the phylogenetic study.

>A_annulatum_CBS_140775

----------------------------------ACCC-TTT-GTGAACC-TTAC-CGT--
CGTTTCCTCGGCGCACTGCT----G---TG-----------
GGA------------------G----------------------GCT----------------ACCCTG-TAGC-----------------
----

--GGTTGTTTACCCTA--------------------------------
---CAGGACGCACCCT------------------
---GCA------------------------------
----GCGGCGCCGAAAGGACT--AC------------CAAA---------ACTCT-----T--TTATC-CA-
-AGTTACCTC-GAACAATTT-ACT--ATA------
CAATAGTTTAAAACTTTCAACAACGGATCTCTTGGTTCTGGCATCGATGAAGAA
CGCAGCGAAATGCGATAAGTAATGTGAATTGCAGAATTCAGTGAATCATCGAA
TCTTTGAACGCACATT-
GCGCCCATTAGTATTCTAGTGGGCATGCCTATTCGAGCGTCATT-

TCAACCCCTTAAG--CCCTGTT-GCTTAGCG-TTGGGAAT-CTGCTAG------------------
------CCTCGG--------CGCAGTTCCTTAAATTCA-TTGGCGGAGCTGT-GG---------
CACACTCTAGGCGTAGTAGTT-T-A-----ACACCTCGCCTCT-AGAG---TG--------------
-GCCG-CGG----------------------------TTACTGGCCGTAAAA----------------CCCCTA-------
-------TATTTCT-----------
AGTGGTTGACCTCGGATTAGGTAGGAATACCCGCTGAACTTAAGCATATCAAT
AAGCGGAGGAAAAGAAACCAACAGGGATTGCCCTAGTAACGGCGAGTGAAGC
GGCAACAGCTCAAATTTGAAATCTGGCCCTCGCGGTCCGAGTTGTAATTTGCAG
AGGATGCTTTTGGTGCGGTGCCTTCCGAGTTCCCTGGAACGGGACGCCAGAGA
GGGTGAGAGCCCCGTACGGTTGGCCGCCTACCCTATATATAGCTCCTTCGACGA
GTCGAGTAGTTTGGGAATGCTGCTCTAAATGGGAGGTAAATTTCTTCTAAAGCT
AAATACCGGCCAGAGACCGATAGCGCACAAGTAGAGTGATCGAAAGATGAAA
AGCACTTTGAAAAGAGGGTTAAATAGCACGTGAAATTGTTGAAAGGGAAGCGT
TTGCGACCAGACTTTTTCCAGGCGGATCATCCGGTGTTCTCACCGGTGCACTTC
GCCTGGTTTAGGCCAGCATCGGTTTCTCTAGGGGGATAAAGGCCTGGGGAACG
TAGCTCTTTAGGGAGTGTTATAGCCCCTTGCGTAATACCCCTCGGGGGACCGAG
GACC----

>A_truncatum_CBS_140778

----------------------------------ACCC-TTT-GTGAACC-TTAC-CGT--
CGTTTCCTCGGCGCACTGCT----G---
CGACCGCCC----CGC------------------A-----------------------GGC----------------GGCCCT-
CGCGGCCCCGCGG---

GCAGCTACTTACCCT-------GT--
-------------------ACGGATCTACCCT---
--------------------------GCA-GCAGCTGAA------------------
GGCGCAGCGGCGCCGAAAGGACC--GC------------TACA--------AACTCT-----T--
TTATC-CA--AGCTACGTCTGAACAATTT-ACTATGCA------
AAATAGTTTAAAACTTTCAACAACGGATCTCTTGGTTCTGGCATCGATGAAGAA
CGCAGCGAAATGCGATAAGTAATGTGAATTGCAGAATTCAGTGAATCATCGAA
TCTTTGAACGCACATT-
GCGCCCATTAGTATTCTAGTGGGCATGCCTATTCGAGCGTCATT-TCGA-
CCCTTAAG--CCCTGTT-GCTTAGCG-TTGGGAGT-CTGCGGC-----------------------
TTCGGC--------CGCAGTTCCTTAAAGTCA-GTGGCGGAGCTGT-GG---------
CACACTCTAGGCGTAGTAGTT-TTA-----CCGCCTCGCCTCC-AGAG---TG------------
--GCCC-CGG---------------------------CTGCCTGCCGTAAAA---------------CCCCTA------
--------ATTTTCT-----------
AGTGGTTGACCTCGGATTAGGTAGGAATACCCGCTGAACTTAAGCATATCAAT
AAGCGGAGGAAAAGAAACCAACAGGGATTGCCCTAGTAACGGCGAGTGAAGC
GGCAACAGCTCAAATTTGAAATCTGGCCCTCGCGGTCCGAGTTGTAATTTGCAG
AGGATGCTTTTGGTGCGGTGCCTTCCGAGTTCCCTGGAACGGGACGCCAGAGA
GGGTGAGAGCCCCGTACGGTTGGACACCTACCCTATATATAGCTCCTTCGACGA
GTCGAGTAGTTTGGGAATGCTGCTCTAAATGGGAGGTAAATTTCTTCTAAAGCT
AAATACCGGCCAGAGACCGATAGCGCACAAGTAGAGTGATCGAAAGATGAAA
AGCACTTTGAAAAGAGGGTTAAATAGCACGTGAAATTGTTGAAAGGGAAGCGT
TTGCGACCAGACTTTTTCCAGGCGGATCATCCGGTGTTCTCACCGGTGCACTTC
GCCTGGTTTAGGCCAGCATCGGTTTCCTTAGGGGGATAAAGGCCTGGGGAACG
TAGCTCTCTAGGGAGTGTTATAGCCCCTCGCGTAATACCCCTCGGGGGACCGAG
GACCGCGC

>D_bambusicola_CBS_122872

TAACAAGGTCTCCGTTGGTGAACCAGCGGAGGGATCATTACCGAGTTATCT------
---------AA---------ACTCC------AACCC-TAT-GTGAAC--CTAC-CGC--
CGTTGCCTCGGCG---GGCC----G--CGCTTAC-------

---------------------------------------CTGCCCG---
----------------------------------GTA--GCGCGCT------------------------ACGGGCCCGCCG-
GTGGACT--GC------------TAAA--------CTCTGT-----T--ATTTA-TA---
AGTATCTCTGAATGCTTC-A----ACT------
TAATAAGTTAAAACTTTCAACAACGGATCTCTTGGTTCTGGCATCGATGAAGAA
CGCAGCGAAATGCGATAAGTAATGTGAATTGCAGAATTCAGTGAATCATCGAA
TCTTTGAACGCACATT-
GCGCCCATTAGTATTCTAGTGGGCATGCCTGTTCGAGCGTCATT-TCAA-
CCCTTAAG--CCCTGCT-GCTTAGTG-TTGGGAGT-CTAGGTC-----------------------
CCAGGG--------CCTAGTTCCTCAAAGTTA-TTGGCGGAGTTGG-GG---------
TATACTCTGAGCGTAGTAGTG-T-------TCTTCTCGCTTTT-GCAG---TA--------------
GCCC-TGG-----------------------------CGGCTTGCCGTAAAA----------------CCCCTA--------
------TATC-TT-----------
AGTGGTTGACCTCGAATCAGGTAGGAATACCCGCTGAACTTAAGCATAT---------

>D_eschscholtzii_MUCL_45435

GTTATCT---------------AA---------ACTCC------AACCC-TAT-GTGAAC--TTAC-CGC--
CGTTGCCTCGGCG---GGCC----G--CGTTCGCCC---
-TGT-----------------A----------------------GTT---

---TACTACCTG--
--GCG--GCGCGCT----------------------
ACAGGCCCGCCG-GTGGACT--GC------------TAAA--------CTCTGT-----T--ATATA-
TA---CGTATCTCTGAATGCTTC-A----ACT------
TAATAAGTTAAAACTTTCAACAACGGATCTCTTGGTTCTGGCATCGATGAAGAA
CGCAGCGAAATGCGATAAGTAATGTGAATTCGAGAATTCAGTGAATCATCGAA
TCTTTGAACGCACATT-
GCGCCCATTAGCATTCTAGTGGGCATGCCTGTTCGAGCGTCATT-TCAA-
CCCTTAAG-CCCCTGTT-GCTTAGCG-TTGGGAAT-CTAGGTC----------------------
TCCAGGG--------CCTAGTTCCCCAAAGTCA-TCGGCGGAGTCGG-AG---------
CGTACTCTCAGCGTAGTAATA-C-------CATTCTCGCTTTT-GCAG---TA--------------
GCCC-CGG--------------------------CGGCTTGCCGTAAAA----------------CCCCTA--------
------TATCTTT-----------AGTGGTTGACCTCGAATCAGGTAGGAT---------------------
----

>D_placentiformis_MUCL_47603

ATTACTGAGTTATCT---------------AA---------ACTCC------AACCC-TAT-GTGAAC--
TTAC-CGC--CGTTGCCTCGGCG---GGCC----G--
CGTTCGCCC----TGT------------------A----------------------GTT--------------------------------
----

---TACTACCTG--------------------------
--GCG----GCGCT----------------------
ACAGGCCCGCCG-GTGGACT--GC------------TAAA--------CTCTGT-----T--ATATA-
TAT-ACGTATCTCTGAATGCTTC-A----ACT------
TAATAAGTTAAAACTTTCAACAACGGATCTCTTGGTTCTGGCATCGATGAAGAA
CGCAGCGAAATGCGATAAGTAATGTGAATTGCAGAA-
TCAGTGAATCATCGAATCTTTGAACGCACATT-
GCGCCCATTAGTATTCTAGTGGGCATGCCTG-TCGAGCGTCATT-TCAA-
CCCTTAAG-CCCCTGTT-GCTTAGCGCTTGGGAAT-CTAGGTC----------------------
TCCAGGG--------CCCTAGTCCCCAAAGTCA-ACGGCGGAGCCGG-AC---------
CGTACTCTCAGCGTAGTAATA-C-------CAATCTCGCTTTT-GTAG---TA--------------
GCCC-CGG---------------------------CGGCTTGCCGTAAAA----------------CCCCTA--------
------TATCTTT-----------AGTGGTTGACCTCGAATCAGGTAGGAATA-------------------
----

>Ruwenzoria_pseudoannulata_MUCL_51394

AGAGGAAGTAAAAGTCGTAACAAGGTCTCCGTTGGTGAACCAGCGGAGGGATC
ATTACCGAGTTATCT--------------AAA---------ACTCC------AACCC-TAT-GTGAAC--
TTAC-CGT--CGTTGCCTCGGCG---GGCT----G---
CGCTTGCCC----GGT------------------A-----------------------GCT----------------GCCCTG-----
----

---CGGCGGCTACCCG---------------
---GTGTAGCGCGCG--------
-------------ACAAGGCCCGCCG-GTGGACC--ACA-----------TAAA--------CTCTGT-----
TATATGTA-TA--CTGTATCTCTGAATGCTTCAA----ACT------
TAATAAGTTAAAACTTTCAACAACGGATCTCTTGGTTCTGGCATCGATGAAGAA
CGCAGCGAAATGCGATAAGTAATGTGAATTGCAGAATTCAGTGAATCATCGAA
TCTTTGAACGCACATT-
GCGCCCATTAGTATTCTAATGGGCATGCCTATTCGAGCGTCATT-
TCAACCCCTTAAG-CCCCTGTT-GCTTAGTG-TTGGGAAT-CTGCGTC-----------------
-------TTGGGG--------CGCAGTTCCTCAAAGTAA-TCGGCGGAGTTGG-GG---------
CTTACTCTGAGCGTAGTAGTA-T-------CTTTCTCGCTTCT-GTGG---TA---------------
GCCC-TGG----------------------------CGGCCGGCCGTAAAA----------------CCCCTA-------
------TTATCTTT-----------
AGTGGTTGACCTCGAATTAGGTAGGAATACCCGCTGAACTTAAGCATATCAAT
A---

>D_childiae_CBS_122881

GATCATTACTGAGTTATCT---------------AA---------ACTCC------AACCC-TAT-
GTGAACC-TTAC-CGT--CGTTGCCTCGGCG---GGCT----G----------------------------------
-----------CGTCTACCC----TGT------------------A-----------------------GCT----------------
ACCCTG---

---TAGCTACCCG--
--GTA-
GGCGTGCT-----------------------ACAAGCCCGCCG-GTGGACC--AC------------TAAA----
----CTCTGT-----T--ATAAA-TA--CTGTATCTCTGAATGCTTC-A----ACT------
TAATAAGTTAAAACTTTCAACAACGGATCTCTTGGTTCTGGCATCGATGAAGAA
CGCAGCGAAATGCGATAAGTAATGTGAATTGCAGAATTCAGTGAATCATCGAA
TCTTTGAACGCACATT-
GCGCCCATTAGTATTCTAGTGGGCATGCCTATTCGAGCGTCATT-TCAA-
CCCTTAAG-CCTAAGTT-GCTTAGCG-TTGGGAAT-CTGCCCTGTA---------------------
TTACGG--------GGCAGTTCCCTAAAGTTA-TCGGCGGAGTTAG-GG---------
CATACTCTAAGCGTAGTACTA-T-------TATTCTCGCTTCT-GCAG---TT---------------
GTCC-CGA----------------------------CGGCTTGCCGCTAAA----------------CCCCTA--------
-----TATTTTCT-----------
AGTGGTTGACCTCGGATTAGGTAGGAATACCCGCTGAACTTAAGCATATCAAT
AAGCGGAGGAAAAGAAACCAACAGGGATTGCCCTAGTAACGGCGAGTGAAGC
GGCAACAGCTCAAATTTGAAATCTGGCCCTAGCGGTCCGAGTTGTAATTTGTAG
AGGATGCTTTTGGTTAGGTGCCTTCCGAGTTCCCTGGAACGGGACGCCAGAGA
GGGTGAGAGCCCCGTACGGTTGGACACCGAGCCTCTATATAGCTCCTTCGACG
AGTCGAGTAGTTTGGGAATGCTGCTCTAAATGGAGGTAAATTTCTTCTAAAGC
TAAATACCGGCCAGAGACCGATAGCGCACAAGTAGAGTGATCGAAAGATGAA

AAGTACTTTGAAAAGAGGGTTAAATAGCACGTGAAATTGTTGAAAGGGAAGCG
TTTGCGACCAGACTTTTTCCAGGCGGATCATCCGGTGTTCTCACCGGTGCACTT
CGCCTGGTTTAGGCCAGCATCGGTTCCCTTAGGGGGATAAAGGCCTGGGGAAC
GTAGCTCCTTCGGGAGTGTTATAGCCCCTAGCGTAATACC-----------------------

>D_concentrica_CBS_113277

CCGAGTTATCT---------------AA---------ACTCC------AACCC-TTT-GTGAAAC-TTAC-
CGT--CGTTGCCTCGGCG---GGCT----G---
CGCTTACCC----TGT------------------A-----------------------GCT----------------ACCCTG-----
----

---TAGCTACCCG-------------------
--GTA-GGCGCGCT--------------
---------CCAAGCCCGCCG-GTGGACC--AC------------TAAA--------CTCTGT-----T---
TTAA-TA--CCGAATCTCTGAATGCTTC-A----ACT------
TAGTAAGTTAAAACTTTCAACAACGGATCTCTTGGTTCTGGCATCGATGAAGAA
CGCAGCGAAATGCGATAAGTAATGTGAATTGCAGAATTCAGTGAATCATCGAA
TCTTTGAACGCACATT-
GCGCCCATTAGTATTCTAGTGGGCATGCCTATTCGAGCGTCATT-TCAA-
CCCTTAAG-CCTTAGTT-GCTTAGCG-TTGGGAGT-CTGCGCTGTAC----------------
TTGTTACGG--------CGCAGTTCCTCAAAGTGA-TTGGCGGAGTTAG-GG---------
CATACTCTAAGCGTAGTAATATT-------TCTTCTCGCTTCT-GTAG---TT---------------
GTCC-TGG----------------------------CGGCTTGCCGTTAAA----------------CCCCT-----------

----

>D_dennisii_CBS_114741

ATTACCGAGTTATCT------------------AA------------ACTCC-------AACCC-TTT-GTGAACC-
TTAC-CGT--CGTTGCCTCGGCG---GGCT----G---
CGCTTACCC----TGT---------------------A---------------------------GCT-------------------ACCCTG------
----

--TAGCTACCCG----------------------
---GTA-GGCGCGCT------------------
---------CTAAGCCCGCCG-GTGGACC--AC--------------TAAA-----------CTCTGT------T---
TTAA-TA--CTGAATCTCTGAATGCTTC-A----ACT-------
TAATAAGTTAAAACTTTCAACAACGGATCTCTTGGTTCTGGCATCGATGAAGAA
CGCAGCGAAATGCGATAAGTAATGTGAATTGCAGAATTCAGTGAATCATCGAA
TCTTTGAACGCACATT-
GCGCCCATTAGTATTCTAGTGGGCATGCCTATTCGAGCGTATAT-TCAA-
CCCTTAAG-CCTCGGTT-GCTTAGCG-TTGGGAAT-CTGCGCTGTAC-----------------------
TTGCTACGG--------CGAAGTTCCTTAAAGTGA-TTGGCGGAGTCAG-AG----------

CATACTCTAAGCGTAGTAATATT-------TCTTCTAGCTTCT-GTAG---TT--------------
GT-C-TGG-----------------------------CGGCTTG-CGTTAAA----------------CCCCTA----------
---TATTTTCT-----------AGTGGTTGACCTCGGATTAGGTAGAATA-----------------------
----

>D_vernicosa_CBS_119316

TAACAAGGTCTCCGTTGGTGAACCAGCGGAGGGATCATTACTGAGTTATCT------
--------AAA---------CTCCC------AACCC-TAT-GTGAACC-TTAC-CGT--
CGTTGCCTCGGCG---GGCT----G--TGCTTACCC----
GGT------------------A----------------------GCT----------------ACCCTG---------------------------
----

---TAGCTACCCG--
--GTA-GGTACGCT------------------------
GCAAGCCCGCCG-GTGGACC--AC------------TAAA--------CTCTGT--------TTAAT-TA-
-CTGTATCTCTGAATGCTTC-A----ACT------
TAATAAGTTAAAACTTTCAACAACGGATCTCTTGGTTCTGGCATCGATGAAGAA
CGCAGCGAAATGCGATAAGTAATGTGAATTGCAGAATTCAGTGAATCATCGAA
TCTTTGAACGCACATT-

GCGCCCGCTAGTATTCTAGCGGGCATGCCTGTCCGAGCGTCATT-TCAA-
CCCTTAAG--CCTAGCT-GCTTAGTG-TTGGGAAT-CTGCCCTGCA-------------------
TCTGCAG--------CGCAGTTCCTTAAAGTAA-TCGGCGGAGTTAG-GG---------
CATACTCTAAGCGTAGTAATATT-------TCTTCTCGCTTCT-GTAG---TT--------------
GTCC-TGG---------------------------CGGCTTGCCGTTAAA---------------CCCCTA--------
------TATTTCT-----------
AGTGGTTGACCTCGGATCAGGTAGGAGTACCCGCTGAACTTAAGCATATC-------

>D_petriniae_MUCL_49214

ATCATTACTGAGTTATCT---------------AA---------ACTCC----CAAACCC-TAT-
GTGAACC-TTAC-CGT--CGTTGCCTCGGCG---GGCC----G--------------------------------
-----------CGCTTACCC----GGT-----------------A----------------------GCT---------------
ACCCTG---

--TAGCTACCCG--
---GTA-
GGTGCGCT----------------------ATAGGCCCGCCG-GTGGACT--AC------------TCAA----
----CTCTGT-----T--TTTAA-TA--CTGTATCTCTGAATGCTTC-A----ACT------

TAATAAGTTAAAACTTTCAACAACGGATCTCTTGGTTCTGGCATCGATGAAGAA
CGCAGCGAAATGCGATAACTAATGTGAATTGCAGAATTCAGTGAATCATCGAA
TCTTTGAACGCACATT-
GCGCCCATTAGTATTCTAGTGGGCATGCCTATTCGAGCGTCATT-TCAA-
CCCTTAAG--CCTAGCT-GCTTAGTG-TTGGGAAC-CTGCCCCGTA--------------------
GCTACGG--------GGCAGCTCCCTAAAGTCA-TCGGCGGAGTTAG-GG---------
CATACTCTAAGCGTAGTACTA-T-------TCTTCTCGCTTCT-GTAG---TT---------------
GTCC-TGG-----------------------------CGGCTTGCCGTTAAA----------------CCCCCTATA--
-------TTTTTTTCT-----------AGTGGTTGACCTCGGATTAGGTAGGAATA----------------
----

>H_pilgerianum_STMA_13455

AGGAAGTAAAAGTCGTAACAAGGTCTCCGTTGGTGAACCAGCGGAGGGATCAT
TACTGAGTTCTTA-------------CAAA---------CTCCC------AACCC-TTT-GTGAATC-
ATAT-CAC--TGTTGCCTCGGCGCCGAGCG----G--
CAGCTACCC----GGG------------------A-----------------------GCT---------------ACCCTG-
TAGAAGC---

GAGAGC---------------------ATCTACC--

CTGTAGCTACCCT--
-------GTA-GTTGCACT---------------------CAACGCTCCGCCG-GCGGACC--AT----------
--TCAA--------CTCTGT-----T----TTA-CA--GTGTATCTCTGAGTACTTA-A----ACT-----
-
AAATAAGTTAAAACTTTCAACAACGGATCTCTTGGTTCTGGCATCGATGAAGA
ACGCAGCGAAATGCGATACGTAATGTGAATTGCAGAATTCAGTGAATCATCGA
ATCTTTGAACGCATATT-
GCGCCCATTAGTATTCTAGTGGGCATGCCTATTCGAGCGTCATT-TCAA-
CCCTTAAG--CCCTGTA-GCTTAGTG-TTGGGACT-CTACTCT-----------------------
TTAGTG--------AGCAGTTCCCTAAAACCA-GTGGCGGTGCTA--GG---------
TACACTCATAGCGTAGTAATT---------CTTTCTCGCTTCT-GCGG---TG-------------
GACG-TAG--------------------------CTACCTGCCGTAAAA---------------CCCCCT--------
------ATTTTCT-----------
AATGGTTGACCTCGGATTAGGTAGGAATACCCGCTGAACTTAAGCATATCAAT
AAGCGGAGGAAAAGAAACCAACAGGGATTGCCCTAGTAACGGCGAGTGAAGC
GGCAACAGCTCAAATTTGAAATCTGGCCCTAGCGGTCCGAGTTGTAATTTGTAG
AGGATGCTTTTGGTGCGGTGCCTTCCGAGTTCCCTGGAACGGGACGCCAGAGA
GGGTGAGAGCCCCGTACGGTTGGACACCTACCCTATATATAGCTCCTTCGACGA
GTCGAGTAGTTTGGGAATGCTGCTCTAAATGGGAGGTAAATTTCTTCTAAAGCT
AAATACCGGCCAGAGACCGATAGCGCACAAGTAGAGTGATCGAAAGATGAAA
AGCACTTTGAAAAGAGGGTTAAATAGCACGTGAAATTGTTGAAAGGGAAGCGT
TTGCGACCAGACTTTTTCAGGGAGGATCATCCGGTGTTCTCACCGGTGCACTTC
ACCCTGTTTAGGCCAGCATCGGTTTTTGCAGGGGGATAAAAACTTGGGGAATG
TGGCTCCTTCGGGAGTGTTATAGCCCCTTGTATAATACCCCTGCGGAG------------
-

>H_wuzhishanense_FCATAS2708

GTTCTTT---------TGGTCTCA---------ACTCC------CACCC-TTT-GTGATC--TTAC-
CAC--TGTTGCCTCGGCGCCGAGCG----G--
CAGCTACCC----GGG------------------A--------------------------GCT----------------ACCCTG-
GAGA--

--CACCTACCCT-------
GTAGAT-----------------------GGCTACC--
CTGGAGCTACCCT---
-------GTA-GTTGCACT-------------------------CAACGCTCCGCCG-GCGGACC--AT----------
--TAAA--------CTCTGT-----T----TTA-CA--TTGTATCTCTGAGTATATA-A----CCA-----
-
AAATACGTTAAAACTTTCAACAACGGATCTCTTGGTTCTGGCATCGATGAAGAA
CGCAGCGAAATGCGATACGTAATGTGAATTGCAGAATTCAGTGAATCATCGAA
TCTTTGAACGCATATT-
GCGCCCATTAGTATTCTAGTGGGCATGCCTATTCGAGCGTCATT-TCAA-
CCCTTAAG-CCCCTGTT-GCTTAGTG-TTGGGAAC-CTACTCC---------------------------
TCAGGG--------TGTAGCTCCCTAAAACCA-GTGGCGGTGCTA--GG---------
TACACTCGTAGCGTAGTAAAT-C-------TTTTCTCGCTCCT-GTAG---TG-----------------
TTCG-TAG--------------------------------TTACCGGCCGTAAAA------------------CCCTTA-------
------TATTTCT-----------
AGTGGTTGACCTCGGATTAGGTAGGAATACCCGCTGAACTTAAGCATATCAAA
AAC--

>H_gibriacense_MUCL_52698

TAGAGGAAGTAAAAGTCGTAACAAGGTCTCCGTTGGTGAACCAGCGGAGGGAT
CATTACCGAGTTCTAT-----------ACAAAA---------CTCCC------AACCC-TTT-
GTGAACC-TTAT-TCA--TGTTGCCTCGGCGCCGAGCG----G------------------------------
--------------CATCTACCC----TGC-------------------A-----------------------GCT----------------
ACTCTG--

--GAGCTACCCT--
---GTA-
GTTGCGCC---------------------AAAACGCTCCGCCG-GTGGACC--AT------------TCAA--
------CTCTGT-----C----TTA-TG--GTGTATCTCTGAACTATAT-A----ACA------
AAATTCGTTAAAACTTTCAACAACGGATCTCTTGGTTCTGGCATCGATGAAGAA
CGCAGCGAAATGCGATAAGTAATGTGAATTGCAGAATTCAGTGAATCATCGAA
TCTTTGAACGCATATT-
GCGCCCATTAGTATTCTAGTGGGCATGCCTATTCGAGCGTCATT-TCAA-
CCCTTAAG--CCCTGTA-GCTTAGCG-TTGGGACC-CTACTCC--------------------
TCCGGGT--------AGTAGCTCCCTAAAACCA-GTGGCGGTGTTC--GG---------
TACACTCATAGCGTAGTATATTT-------TATTTTCGCTTCT-GCAG---TG-------------
GACC-GGA----------------------------CGGCTCGCCGTAAAA----------------CCCCTTA-----
------TATTTTTCT-----------
AGTGGTTGACCTCGGATTAGGTAGGAATACCCGCTGAACTTAAGC------------------
----

>H_addis_MUCL_52797

GGAAGTAAAAGTCGTAACAAGGTCTCCGTTGGTGAACCAGCGGAGGGATCATT
ACTGAGTTCTAC------------AAAAA---------CTCCC------AACCC-TTT-GTGAACC-
ATAC-TTC--AGTTGCCTCGGCGCTGAGCG----G---
TAGCTACCC----GGG-------------------------A----------------------AGAGCT-----------------ACCCTG-
GAGAAATCCGG--

GGCGACCTACCCT--
-------GTA-GTTGCACC------------------------TAACGCTCCGCCG-GTGGACC--AT-----------
--TACA--------CTCTGT-----T-TTTAAC-CA--CTGTATCTCTGAAATACTT-A----ACG--
----
AAATACGTTAAAACTTTCAACAACGGATCTCTTGGTTCTGGCATCGATGAAGAA
CGCAGCGAAATGCGATAAGTAATGTGAATTGCAGAATTCAGTGAATCATCGAA
TCTTTGAACGCATATT-
GCGCCCATTAGTATTCTAGTGGGCATGCCTATTCGAGCGTCATT-TCAA-
CCCTTAAG--CCCTGTT-GCTTAGTG-TTAGGAGC-CTGCTTT------------------------
CAGTAGAG--------GGCAGCTCCCTAAAGATA-GTGGCAGTGTTC--GG---------
TACACTCGTAGCGTAGTAATT-T-------CTATCTCGCTTCT-GTAG---TG-----------------
GCCC-GAA-------------------------------TTTCTCGCCGTAAAA-----------------CCCCCT--------
------ATTTTCT-----------
CAAGGTTGACCTCGGATTAGGTAGGAATACCCGCTGAACTTAAGCAT-------------

>H_chionostomum_STMA_14060

CGGAGGGATCATTACTGAGTTCTAC---------------AAA---------ACTCC------CACCC-
TTT-GTGAATT-ATAC-TCC--TGTTGCCTCGGCGCCGAGCG----G-------------------------
----------------------CAGCTACCC----TGG------------------A------------------------GCT-----------
-----ACCCGG--

TAGCTACCCG---
-GTA-GTTGCGCC---------------------------CACGCTCCGCCG-GTGGACC--AT------------
TACA--------CTCTTG--------TTATA-CA--CTGTATCTCTGAGACATTA------ACT------
AAATACGTTAAAACTTTCAACAACGGATCTCTTGGTTCTGGCATCGATGAAGAA
CGCAGCGAAATGCGATAAGTAATGTGAATTGCAGAATTCAGTGAATCATCGAA
TCTTTGAACGCATATT-
GCGCCCATTAGTATTCTAGTGGGCATGCCTATTCGAGCGTCATT-TCAA-
CCCTTACG--CCCTGTT-GCGTAGTG-TTGGGAGT-CTACCCT---------------------
GTAGAAGG--------GGTAGTTCCCGAAAACCA-GTGGCGGTGTTC--GG---------
TACACACATAGCGTAGTAATT-T-------ATTTCTCGCTTCT-GTCG---TG--------------
GCCT-GAG-----------------------------CTACCCGCCGTAAAA----------------CCCCCT--------
-----ATACTTCTAA--

>H_musceum_MUCL_53765

TAAAAGTCGTAACAAGGTCTCCGTTGGTGAACCAGCGGAGGGATCATTACTGA
GTTCTAC------------AAAAA---------CTCCC------AACCC-TTT-GTGAATT-CTAC-TCT-
-TGTTGCCTCGGCGCCGAGCA----G--
CAGCTACCC----GGG------------------A----------------------GCT----------------ACCCGG-
GAGT--

---CAGCTACCCT-------
GTAGTT---------------------GGCTACC---
CGGTAGCTACCCT--
-------GTA-GATGCGCC-----------------------TACGCTCCGCCG-GTGGACC--AT-----------
-GCAA--------CTCTTG-----T--TTTTA-TA--GTGTATCTCTGAACCATAT-A----ACT----
--
AAATTCGTTAAAACTTTCAACAACGGATCTCTTGGTTCTGGCATCGATGAAGAA
CGCAGCGAAATGCGATAAGTAATGTGAATTGCAGAATTCAGTGAATCATCGAA
TCTTTGAACGCATATT-
GCGCCCATTAGTATTCTAGTGGGCATGCCTATTCGAGCGTCATT-TCAA-
CCCTTAAG--CCCTGTT-GCTTAGTG-TTGGGAAT-CTACCCTGTACCTACCC---------
--TGTAGGGGA--------GGTAGTTCCCGAAAATCA-GTGGCGGTGTTC--GG---------
TACACACATAGCGTAGTAAAT-CCA-----TTATCTCGCTTCT-GTAG---TG-------------
--GCCT-GAA----------------------------TATCCCGCCGTGAAA----------------CCCCCT------
--------AATTTTC-----------
CATGGTTGACCTCGGATTAGGTAGGAATACCCGCTGAACTTAAGC------------------
----

>H_cercidicola_CBS_119009

ATCATTACTGAGTTCTAC------------AAAAA---------CTCCC------AACCC-TTT-
GTGAACC-CTAC-CGT--CGTTGCCTCGGCGCTGAGCG----G--------------------------------
--------------CGGCTACCC----GGG------------------A--------------------GAAGCT---------------
-ACCCTG-GAGA--

CACCTACCCT-------GTAGGT--------------------GGCTACC-------------------------------------
-----------------------CTGGAGTTACCCT--
-------------------------------GTA-GTTGCACT----------------------TTACGTTCCGCCG-
GAGGACC--ACA-----------CAAA--------CTCTTC-----T-GTATTC-TG--
GTGTATCTCTGAGTACGTA------ACA------
AAATAAGTTAAAACTTTCAACAACGGATCTCTTGGTTCTGGCATCGATGAAGA
ACGCAGCGAAATGCGATAAGTAATGTGAATTGCAGAATTCAGTGAATCATCGA
ATCTTTGAACGCATATT-
GCGCCCATTAGTATTCTAGTGGGCATGCCTATTCGAGCGTCATT-TCAA-
CCCTTAAG--CCCTGTT-GCTTAGCG-TTGGGACC-CTACCCT-----------------------
GTAGCAGG--------CGTAGTTCCCTAAAGGTA-GTGGCAGTGTTA--GG---------
TACACTCGTAGCGTAGTAATC---------TTTTCTCGCTCCT-GTGG---TG-------------
GCCC-TGA-----------------------------CGACTCGCCGTAAAA-----------------CCCCCT--------

----ACAACTTCT-----------AGTGG--

>H_petriniae_CBS_114746

GAAGTAAAAGTCGTAACAAGGTCTCCGTTGGTGAACCAGCGGAGGGATCATTA
CTGAGTTCTAC-------------AAAA---------CTCCC------AACCC-TTT-GTGAACC-
TTAC-CGT--CGTTGCCTCGGCGCTGAGCG----G--
TAGCTACCT----GGG------------------A--------------------GAAGCT----------------ACCCTG-
GAGA--

---CACCTACCCT-------
GTAGGT---------------------GGCTACC--
CTGGAGCTACCCT--
-------GTA-GTTGCACT----------------------TTACGCTCCGCCG-GAGGACC--ACA--------
---CAAA--------CTCTTC-----T-GTTTTA-TG--GTGTATCTCTGAATCGTTA------ACT---
---
AAATACGTTAAAACTTTCAACAACGGATCTCTTGGTTCTGGCATCGATGAAGAA
CGCAGCGAAATGCGATAAGTAATGTGAATTGCAGAATTCAGTGAATCATCGAA
TCTTTGAACGCATATT-
GCGCCCATTAGTATTCTAGTGGGCATGCCTATTCGAGCGTCATT-TCAA-

CCCTTAAG-CCCCTGTT-GCTTAGCG-TTGGGACT-CTACCCT----------------------
GCTGCAGG--------TGTAGTTCCCTAAAGGTA-GTGGCAGTGTTA--GG---------
TACACTCGTAGCGTAGTAATT---------CTTTCTCGCTTCG-GTGG---TG---------------
GCCC-TAG-----------------------------CTACTCGCCGTGAAA----------------CCCCCCT------
-----ATAACTTCT-----------
AGTGGTTGACCTCGGATTAGGTAGGAATACCCGCTGAACTTAAGCATAT----------

>H_guilanense_MUCL_57726

GAGGAAGTAAAAGTCGTAACAAGGTCTCCGTTGGTGAACCAGCGGAGGGATCA
TTACTGAGTTCTAC-----------AAAAAA---------CTCCC------AACCC-TTT-GTGAACC-
TTAC-CGT--CGTTGCCTCGGCGCCGAGCG---G--
CGGCTACCC----TGG------------------A--------------------GAAGCT----------------
ACCCGG-GAGC--

CACCTACCCT-------GTAGGT---------------------GGCTACC------------------------------------
----------------------CTGGAGCTACCCT---
------------------------------GTA-GTTGCATT----------------------CTACGCTCCGCCG-
GCGGACC-TTC------------TACA--------CTCTGT-----T--TTGTA-TA--

GTGTATCTCTGAAACCTAT-A----ACG------
TAATACGTTAAAACTTTCAACAACGGATCTCTTGGTTCTGGCATCGATGAAGAA
CGCAGCGAAATGCGATACGTAATGTGAATTGCAGAATTCAGTGAATCATCGAA
TCTTTGAACGCATATT-
GCGCCCATTAGTATTCTAGTGGGCATGCCTATTCGAGCGTCATT-TCAA-
CCCTTACG-CCCCTGTT-GCGTAGTG-TTGGGAAC-CTACAGG------------------------
CCTGTAAAGAGGACCTGTAGCTCCCTAAAGGTA-GTGGCGGTGTTA--GG---------
TACACTCGTAGCGTAGTAACATC-------TTTTCTCGCTCCT-GCAG---TG----------------
TACC-TAA-------------------------------GGCCTGCCGTGAAAA-------------ACCCCCT-------
-----ATAACTTCT-----------
AGTGGTTGACCTCGGATTAGGTAGGAATACCCGCTGAACTTAAGCATATCA-----

>H_texense_DSM_107933

GGAAGTAAAAGTCGTAACAAGGTCTCCGTTGGTGAACCAGCGGAGGGATCATT
ACTGAGTTCTCC------------CAAAA---------CTCCC------AACCC-TTT-GTGAACC-
TTAC-CGT--CGTTGCCTCGGCGCCGAGCG----G--
CGGCTACCC----TGG------------------A--------------------GAAGCT----------------
ACCCGG-GAGAG--

CCACCTACCCC-------GTAGGT-------------------GGCTACC------------------------------
----------------------CTGGAGCTACCCTGT---
-----------------------------AGTA-GTTGCTCT--------------------TTACGCTCCGCCG-
GTGGACC--TC-----------TACA--------CTCTGT-----T--TTGTA-TA--
GTGTATCTCTGAAACCTAT-A----ACT------
TAATACGTTAAAACTTTCAACAACGGATCTCTTGGTTCTGGCATCGATGAAGAA
CGCAGCGAAATGCGATAAGTAATGTGAATTGCAGAATTCAGTGAATCATCGAA
TCTTTGAACGCATATT-
GCGCCCATTAGTATTCTAGTGGGCATGCCTATTCGAGCGTCATT-TCAA-
CCCTTACG--CCCTGCT-GCGTAGTG-TTGGGAAC-CTACAAG---------------------
CCCGTGAAAAGGCCCTGTAGCTCCCTAAAGGTA-GTGGCGGTGTTA--GG--------
TACACTCGTAGCGTAGTACGT-C-------TTTTCTCGCTTCT-GTAG---TG--------------
CACC-TAG--------------------------GGCCTGCCGTGAAA--------------ACCCCTT-------
----ATACCTTCT----------
AGTGGTTGACCTCGGATTAGGTAGGAATACCCGCTGAACTTAAGCATAT---------

>H_rubiginosum_MUCL_52887

TCATTACTGAGTTCTAC-----------AAAAA--------CTCCC-----AACCC-TTT-

GTGAACC-TTAC-CGT--CGTTGCCTCGGCGCCGAGCG----G----------------------------------
--------------CGGCTACCC----GGG------------------A-----------------------GCT----------------
ACCCTG-TAGAC---

CACCTACCCT-------GTAGGT----------------------GCCTACC--------------------------------------
----------------------CTGTAGCTACCCT--
-----------------------------GTA-GTTGCACT-----------------------TTACGCTCCGCCG-
GTGGACT--AC------------CCAA--------CTCTGT-----T--TTTAA-TG--
GTGTATCTCTGAAAACAAA-A----ACA------
AAATAAGTTAAAACTTTCAACAACGGATCTCTTGGTTCTGGCATCGATGAAGA
ACGCAGCGAAATGCGATAAGTAATGTGAATTGCAGAATTCAGTGAATCATCGA
ATCTTTGAACGCATATT-
GCGCCCACTAGTATTCTGGTGGGCATGCCTATTCGAGCGTCATT-TCAA-
CCCTTAAG--CCCTGTT-GCTTAGTG-TTAGGAGC-CTACTAC------------------------
CTTACGG--------CGTAGCTCCCTAAAGGCA-GTGGCGGTGTTA--GG---------
TACACTCGTAGCGTAGTAATTCT-------TTTTCTCGCTCCT-GCTG---TG---------------
TACC-TAG-----------------------------GGCCTGCCGTAAAA----------------CCCCTA---------
-----TACTTCT-----------AGTG---

>H_laschii_MUCL_52796

--TT-GTGAACC-TTAC-CGT--
CGTTGCCTCGGCGCCGAGCG----G--
CGGCTACCC----TGG----------------A-----------------------GCT----------------ACCCTG-
TACCTGCT--
CTGTAA--

CTTGAAGTTA-------CTAAGT---------------------AGCTACC-----------------------------------
----------------------TTGTAGCTACCCT--
-----------------------------GGA-GTTGCACT----------------------CTACGCTCCGCCG-
GAGGACC--AC------------TACA--------CTCTGT--------TTTTC-TA--
GTGTATCTCTGAAATCTTA------ACT------
TAATACGTTAAAACTTTCAACAACGGATCTCTTGGTTCTGGCATCGATGAAGAA
CGCAGCGAAATGCGATAAGTAATGTGAATTGCAGAATTCAGTGAATCATCGAA
TCTTTGAACGCATATT-
GCGCCCATTAGTATTCTAGTGGGCATGCCTATTCGAGCGTCATT-TCAA-
CCCTTAAG--CCCTGTA-GCTTAGCG-TTGGGAAC-TACGGCC-----------------------
CTAGGGG--------CGTAGCTCCCTAAAGGTA-GTGGCGGTGTTA--GG---------
TACACTCGTAGCGTAGTAGCT-T-------TCTTCTCG--

>H_barbarensis_STMA_14081

GGAAGTAAAAGTCGTAACAAGGTCTCCGTTGGTGAACCAGCGGAGGGATCATT
ACTGAGTTATCC-------------AAAA---------CTCCC------AACCC-TTT-GTGAAC--
TTAC-CAC--TGTTGCCTCGGCG---AGCT----G---TG-
CTACCC----TAT-----------------A----------------------GCT---------------ACC----------------
----

---CTGTAGCTACCCG------------------------
--GGA--GCACGCT---------------------
--ATAAGCTCGCCA-GAGGACC--AC------------TAAA--------CTCTGT--------TTTTA-
TT--CTGTATCTCTGAATCGTAT-A----ACT------
AAATCAGTTAAAACTTTCAACAACGGATCTCTTGGTTCTGGCATCGATGAAGAA
CGCAGCGAAATGCGATAAGTAATGTGAATTGCAGAATTCAGTGAATCATCGAA
TCTTTGAACGCACATT-
GCGCCCATTAGTATTCTAGTGGGCATGCCTATTCGAGCGTCATT-TCAA-
CCCTTAAG-CCTCGGTT-GCTTAGCG-TTGGGATT-CTACGGG-----------------------
TTACAG--------CGTAGTTCCCCAAAAGTA-GTGGCGGAGTTGG-TG---------
CTCACTCTCAGCGTAGTAATC---------TCTTCTCGCTTTT-GTAG---TG--------------
GCGC-TGG----------------------------CGGCCGGCCGTAAAA----------------CCCCCT-------
------ATATTTCT-----------
AGTGGTTGACCTCGGATTAGGTAGGAATACCCGCTGAACTTAAGCATAT----------

>H_submonticulosa_CBS_115280

GAGGAAGTAAAAGTCGTAACAAGGTCTCCGTTGGTGAACCAGCGGAGGGATCA
TTACTGAGTTATCC------------AAAAA---------CTCCC------AACCC-TAT-GTGAAC--
TTAC-CAC--TGTTGCCTCGGCG---AGCT----G--
CG-CTACCC----TGT------------------A----------------------GCT----------------ACTT----------
----

---TTGTAGCGACCTG-------------------
--GGA--GCGCGTT--------------
--------ATAGGCTCGCCA-GAGGACC--AC------------TAAA--------CTCTGT---------
TTTA-TT--TTGTATCTCTGAATCTTAC-A----ACT-----
GAAATCAGTTAAAACTTTCAACAACGGATCTCTTGGTTCTGGCATCGATGAAGA
ACGCAGCGAAATGCGATAAGTAATGTGAATTGCAGAATTCAGTGAATCATCGA
ATCTTTGAACGCACATT-
GCGCCCATTAGTATTCTAGTGGGCATGCCTATTCGAGCGTCATT-TCAA-
CCCTTAAG-CCTCAGTA-GCTTAGCG-TTGGGAAT-CTACAGG-----------------------
TTACAG--------CGTAGTTCCTTAAAGGTA-GTGGCGGAGTTAG-AG---------
CACACTCTAAGCGTAGTACTT---------GTTTCTCGCTTCTGGTAG---TG--------------
GCTT-TGG---------------------------CGGCCGGCCGTGAAA---------------CCCCCT--------
-----ATATTTCT-----------
AATGGTTGACCTCGGATTAGGTAGGAATACCCGCTGAACTTAAGCATAT----------

>H_monticulosa_MUCL_54604

TAGAGGAAGTAAAAGTCGTAACAAGGTCTCCGTTGGTGAACCAGCGGAGGGAT
CATTACTGAGTTATCC-------------AAAA---------CTCCC------AACCC-TTT-GTGAAC-
-TTAC-CAC--TGTTGCCTCGGCG---AGTT----G---
TG-CTACCC----TAT------------------A------------------------GCT-----------------ACC------------
----

---CTGTAGCTACCCG---------------------
--GGA--ACACATT-----------------
-------CCAAGCTCGCCA-GAGGACC--TA------------CCAA--------CTCTGT---------
TTTA-TA--CTGTATCTCTGAACTTTAT-A----ACT------
AAATAAGTTAAAACTTTCAACAACGGATCTCTTGGTTCTGGCATCGATGAAGA
ACGCAGCGAAATGCGATAAGTAATGTGAATTGCAGAATTCAGTGAATCATCGA
ATCTTTGAACGCACATT-
GCGCCCATTAGTATTCTAGTGGGCATGCCTATTCGAGCGTCATT-TCAA-
CCCTTAAG-CCTCAGTT-GCTTAGTA-TTGGGACT-CTACGAC---------------------
CTATTATAG--------CGTAGTTCCTTAAAGGTA-GTGGCGGAGTTAT-AG---------
CACACTCTAAGCGTAGTAATT---------CTCTCTCGCTTCTTGTAG---TG---------------
GTTA-TAG-----------------------------TTGCTAGCCATAAAA----------------CACCCC--------
------CTATTTT-----------
AATGGTTGACCTCGGATTAGGTAGGAATACCCGCTGAACTTAAGCATATCA-----

>H_fendleri_MUCL_54792

GGAAGTAAAAGTCGTAACAAGGTCTCCGTTGGTGAACCAGCGGAGGGATCATT
ACAGAGTTATAC------------TAAA---------CTCCA------AACCC-TAT-GTGAACC--
TAC-CAC--TGTTGCCTTGGCG-TGTGCC----G--CG-

---GGCTACCCT---
--GTA--GTGCGCA---------------------
TACGGCCCGCCG-AAGGACC--AC-----------TAAA--------CTCTTT----GT--TTTTA-
CT--GTGAATATCTGAATGCTTCAA----ACT------
TAATAAGTTAAAACTTTCAACAACGGATCTCTTGGTTCTGGCATCGATGAAGAA
CGCAGCGAAATGCGATAAGTAATGTGAATTGCAGAATTCAGTGAATCATCGAA
TCTTTGAACGCACATT-
GCGCCCATTAGTATTCTAGTGGGCATGCCTATTCGAGCGTCATT-TCAA-
CCCTTAAG--CCCTGTT-GCTTAGCG-TTGGGAAT-CAGCGTC----------------------
TTTCGG--------CGCTGTTCCTTAAATTTA-GTGGCGGAGTTAT-AG---------
CACACTCTAAGCGTAGTAAAT----------CTTCTCGCTTCT-GAAG---TT--------------
GCCC-TAA-------------------------TTCTTAGCCGTAAAA---------------CCCCCT---------
-----ATTTTCT-----------
AATGGTTGACCTCGGATTAGGTAGGAATACCCGCTGAACTTAAGCATATCATA-

>H_invadens_MUCL_51475

TAGAGGAAGTAAAAGTCGTAACAAGGTCTCCGTTGGTGAACCAGCGGAGGGAT
CATTACAGAGTTACTT-----------AAAAAA---------CTCCC------AACCC-TTT-
GTGAAC--TTAC-CAC--TGTTGCCTCGGCG-CTGGCT----G---------------------------------
------------CGGCTACCC----TAT-----------------A----------------------GCT---------------
ACCCTG-TAGGA--

-------------------------AACTACC---
CTGTACCTATCTT---
------GTA--GTACGCT----------------------TAGGCCCGTCG-GAGGACC--AT-----------
TGAA--------CTCTTG-----T--TTTTA-CT--GTGAATATCTGAATGCTTC-A----ACT-----
GAAATAAGTTAAAACTTTCAACAACGGATCTCTTGGTTCTGGCATCGATGAAG
AACGCAGCGAAATGCGATAAGTAATGTGAATTGCAGAATTCAGTGAATCATCG
AATCTTTGAACGCACATT-
GCGCCCATTAGTATTCTAGTGGGCATGCCTATTCGAGCGTCATT-TCAA-
CCCTTAAG-CCCCTGTT-GCTTAGCG-TTAGGAGA-CTGAGCC--------------------
CCCCCGGGG--------CCCAGCTCCCTAAAACTA-GTGGCGATGTTAT-GG---------
CACATCCTAAGCGTAGTAATT-T-------TCTTCTCGCTTCT-GTGA---TT--------------
GTCG-AAC----------------------------CTATCTGCCGTAAAA---------------CCCCCT--------
------ATATTCT-----------
AGTGGTTGACCTCGGATTAGGTAGGAATACCCGCTGAACTTAAGCATATCA-----

>H_macrocarpum_CBS_119012

GTAACAAGGTCTCCGTTGGTGAACCAGCGGAGGGATCATTACAGAGTTACTT---
---------AAAAA---------CTCCC------AACCC-TTT-GTGAAC--TTAC-CAC--
TGTTGCCTCGGCG-TTGGCT----G---CGGCTACCC--
--TAT------------------A-----------------------GTT----------------ACCCTG-TAGGC--------------
----

AACTACC---CTGTACCTATCTC-------------
--GTA--GTTCGCT----------
--------------TAGGCCCGTCG-GAGGACC--AC------------TGAA--------CTCTTG-----T--
TTTTA-CT--GTGAATATCTGAATGCTTC-A----ACT-----
GAAATAAGTTAAAACTTTCAACAACGGATCTCTTGGTTCTGGCATCGATGAAG
AACGCAGCGAAATGCGATAAGTAATGTGAATTGCAGAATTCAGTGAATCATCG
AATCTTTGAACGCACATT-
GCGCCCATTAGTATTCTAGTGGGCATGCCTATTCGAGCGTCATT-TCAA-
CCCTTAAG-CCCCTGTT-GCTTAGCG-TTGGGAGA-CTGAGCCCCCC-----------------
CCCCGGGGG--------CCCAGCTCCTTAAAAGTA-GTGGCGATGTCGT-GG---------
CACATCCTAAGCGTAGTAATT-T-------TCTTCTCGCTTCT-GTGA---TT--------------
GTCG-CGG----------------------------CTATCTGCCGTAAAA----------------CCCCCT--------

-----ATATTTCT-----------
AGTGGTTGACCTCGGATTAGGTAGGAATACCCGCTGAACTTAAGCATATCA-----

>H_aveirense_MUM_19_40

GCGGAGGGACATTACTGAGTTCT-----------------AA---------ACTCC------AACCC-
TAT-GTGAAC--TTAC-CAC--TGTTGCCTCGGCG-TGTGCT------------------------------
----

--CGCGAC---------------------
AGCAGCCCGCCA-GTGGGCC--TT------------TAAA--------CTCTGA-----T--ATTAC-
CA--CTGTATCTCTGAATTTCTT-A----ACT-----
GAAATACGTTAAAACTTTCAACAACGGATCTCTTGGTTCTGGCATCGATGAAGA
ACGCAGCGAAATGCGATAAGTAATGTGAATTGCAGAATTCAGTGAATCATCGA
ATCTTTGAACGCACATT-
GCGCCCATTAGTATTCTAGTGGGCATGCCTATTCGAGCGTCATT-TCAA-
CCCTTGCG-CCCCTGTT-GCGTAGCG-TTGGGAAT-CTGCG-------------------------

TACAG--------CGCAGTTCCTCAAAGTGATCTGGCGGAGCTAG-TG---------
CATACTCTAAGCGTAGTAAACAC-------CATTCTCGCTTCT-GTAG---TA-------------
-GGCC-TGG----------------------------CGGCTAGCCGTTAAA----------------CCCCCT-------
------ATACTTCT-----------
AGTGGTTGACCTCGGATTAGGTAGGAATACCCGCTGAACTTAA---------------------
----

>H_griseobrunneum_CBS_331_73

GGAAGTAAAAGTCGTAACAAGGTCTCCGTTGGTGAACCAGCGGAGGGATCATT
ACTGAGTTCT-----------------AA---------ACTCC------AACCC-TAT-GTGAAC--TTAC-
CAC--TGTTGCCTCGGCGCTGTGCC---

--------------------------------TGCGAG---------------------AGCAGGCCCGCCG-
GTGGACC--AC------------TAAA--------CTCTGC-----T--ATACC-TA--
CTGTATCTCTGAATTTATA------ACT-----
GAAATACGTTAAAACTTTCAACAACGGATCTCTTGGTTCTGGCATCGATGAAGA

ACGCAGCGAAATGCGATAAGTAATGTGAATTGCAGAATTCAGTGAATCATCGA
ATCTTTGAACGCACATT-
GCGCCCATTAGTATTCTAGTGGGCATGCCTATTCGAGCGTCATT-TCAA-
CCCTTAAG-CCCCTGTT-GCTTAGTG-TTGGGAAT-CTGCG------------------------
TTACGG--------CGCAGTTCCTTAAAGTGATTTGGCGGAGCTAG-TG---------
CATACTCTAGGCGTAGTAAATAC-------CATTCTCGCTTTT-GTAG---TA--------------
GGCC-TGG--------------------------CGGCTTGCCGTAAAA---------------CCCCTA--------
------TATTTCT-----------
AGTGGTTGACCTCGGATTAGGTAGGAATACCCGCTGAACTTAAGCATAT----------

>A_michelianum_CBS_119993

CGAACAAGGTCTCCGTTGGTGAACCAGCGGAGGGATCATTAACGAGTTACCA---
----------AAAC---------TCCAA------AACCC-TTT-GTGAACC-TTAC--CT--
AGTTGCCTCGGCG-TGAGCT----G--CGGTTACCC-
---TGG-------------------A----------------------GCT---------------ACCCTG-GAGCGTT--------
--ACCCTGTAGC-----------------

--GCTACCGCTTACCCC-------
GTAAGT------------------GGTCCAAGG---

AAGAAGCTACCCT---
--------GGA--ACCGGCC------------------------TACGGCCCGCCG-GAGGACC--GC---------
---TAAA--------CTCTGT-----C-TTATAC-CA--CTGTATCTCTGAATTCGTA------ACT--
---
AAAATAAGTTAAAACTTTCAACAACGGATCTCTTGGTTCTGGCATCGATGAAG
AACGCAGCGAAATGCGATAAGTAATGTGAATTGCAGAATTCAGTGAATCATCG
AATCTTTGAACGCACATT-
GCGCCCATTAGTATTCTAGTGGGCATGCCTATTCGAGCGTCATT-TCGA-
CCCTTAAG--CCTTGCT-GCTTAGCG-TTGGGAGT-CTACGGC-------------------------
TTTGG--------CGTAGCTCCTTAAAGTCA-GTGGCGGAGTTAT-GG---------
CACACTCTAAGCGTAGTAATC-TTT-----ACCTCTCGCTTCT-GTAG---TT-------------
-GCCT-TAA----------------------------CTGCCTGCCGTAAAA-----------------CCCCCT-------
-----ATATTTTCT-----------
AATGGTTGACCTCGGATTAGGTAGGAATACCCGCTGAACTTAAGCATATCA-----

>J_cohaerens_CBS_119126

TCTTGGTCATTTAGAGGAAGTAAAAGTCGTAACAAGGTCTCCGTTGGTGAACC
AGCGGAGGGATCATTACAGAGTTAAAC--------------AAA---------CTCCA------
AACCC-TTT-GTGAACC-TTAC-CATA-AGTTGCCTCGGCG-TGCGCT----G-------------

---------------------------------CGGCTACCC----GGT------------------T------------------------TCC-
----------------GGCCC--

------------------------------------CCCAGAA---
GGGTGGTTACCCT--
-------GTA--GCCGGCC------------------------AACAGCCCGCCG-AAGGACC--CC-----------
-TAAA--------CTCTGT-----T--TAAAA-TG--GTGTATTCTGAATTACTTA-A----ACT----
-
AAAATAAGTTAAAACTTTCAACAACGGATCTCTTGGTTCTGGCATCGATGAAG
AACGCAGCGAAATGCGATAAGTAATGTGAATTGCAGAATTCAGTGAATCATCG
AATCTTTGAACGCACATT-
GCGCCCATTAGTACTCTAGTGGGCATGCCTATTCGAGCGTCATT-TCGA-
CCATTAAG--CCCTGCA-GCTTAGCG-TTGGGAGC-CTACGCC-----------------------
CTTGCGG--------CGTAGCTCCTCAAAGTCA-GTGGCGGAGTTGG-AG---------
CACACTCTAAGCGTAGTAAAC---------TTATCTCGCTTCT-GTAG---TT---------------
GCCC-CGG-----------------------------CTGCCTGCCGTAAAA----------------CCCCCT--------
-----ATATTTCT-----------
AGTGGTTGACCTCGGATTAGGTAGGAATACCCGCTGAACTTAAGCATATCAAT
AAGCGGAGGAA---

>J_multiformis_CBS_119016

--------------------CTCCA------AACCC-TTT-GTGAACC-TTAC-CTCC-
AGTTGCCTCGGCG-CTCGCT----G---CGGCTACCC-
---CGT-----------------A---------------------GTC--------------CGCCCTG-TAGAAC---------
----

--GGGCGGCTACCCT------------------------
---GTA--GCCGGCT---------------------
CACGGCCCGCCG-AAGGACC--CC------------TAAA--------CTCTGT-----T--TAAAA-
TA--GTGTATTTCTGAATGCTCCAA----ACA-----
AAAATAAGTTAAAACTTTCAACAACGGATCTCTTGGTTCTGGCATCGATGAAG
AACGCAGCGAAATGCGATAAGTAATGTGAATTGCAGAATTCAGTGAATCATCG
AATCTTTGAACGCACATT-
GCGCCCATTAGTATTCTAGTGGGCATGCCTATTCGAGCGTCATT-TCGA-
CCATTAAG--CCCTGTA-GCTTAGCG-TTGGGAGC-CTACGGT------------------------
TGTAG--------CGTAGCTCCTCAAAGTCA-GTGGCGGAGTTAT-GG---------
CGTACTCTGAGCGTAGTAAAT-C-------TTTTCTCGCTTCT-GTAG---TC--------------
GCCC-TAG--------------------------CTGCCTGC--

>H_papillatum_ATCC_58729

GTAACAAGGTCTCCGTTGGTGAACCAGCGGAGGGATCATTAGCGAGTTTACA---
-----------AAA---------CTCCC----AAAACCC-TTT-GTGAACC-TTAC-CAT--
TGTTGCCTCGGCG-TGAGCC----G--CGGCTACCT-
---GGT------------------A-----------------------GCT----------------ACCCTG-TAATT-------------
----

---GTTACCCT-------GTAAT-------------------
---GATTACC---CGGGAGCTACCCT---------
--GTA--GTACGCG-----
-----------------TGAAGGCCCGCCG-GTGGACC--AC------------TAAA--------CTCTGT----
-T--TATCA-TT--GTGGAATTCTGAATACTTA------ACT-----
GAAATACGTTAAAACTTTCAACAACGGATCTCTTGGTTCTGGCATCGATGAAGA
ACGCAGCGAAATGCGATAAGTAATGTGAATTGCAGAATTCAGTGAATCATCGA
ATCTTTGAACGCACATT-
GCGCCCATTAGTATTCTAGTGGGCATGCCTATTCGAGCGTCATTAACAA-
CCCTTAAG-CCCTTGTT-GCTTAGTG-TTGGGACT-CTACGGC----------------------
CTTAGGG--------CGTAGTTCCTTAAAGTTA-GTGGCGGAGTTAG-GG---------
TACACTCTCAGCGTAGTAGTT-A-------TTTTCTCGCTTTT-GTAG---TG--------------
GTCC-TAA---------------------------CGGCTAGCCGTAAAA----------------CCCTTA--------
-------TATTCT-----------
AGTGGTTGACCTCGGATTAGGTAGGAATACCCGCTGAACTTAAGCATATCATA-

>H_sp_MUCL_51392

GTAACAAGGTCTCCGTTGGTGAACCAGCGGAGGGATCATTAGCGAGTTTACA---
-----------AAA---------CTCCC----AAAACCC-TTT-GTGAACC-TTAC-CAC--
TGTTGCCTCGGCG-TGAGCT----G---CGTCTACCT--
--GGT-------------------A------------------------GTT-----------------ACCCTG-TATTTATT--------
--ACCCTGTA------------------------

--TTTATTACCCT-------GTAAT-------
---------------GATTACC---
CGGGAGCTACCCT--
--------GTA--ATTCGCG-----------------------TGAAGGCCCGCCG-GTGGACC--AC---------
---TAAA--------CTCTGT-----T--TATCA-CT--GTGGAATTCTGAATACCTA-A----ACT--
---
GAAATACGTTAAAACTTTCAACAACGGATCTCTTGGTTCTGGCATCGATGAAGA
ACGCAGCGAAATGCGATAAGTAATGTGAATTGCAGAATTCAGTGAATCATCGA
ATCTTTGAACGCACATT-
GCGCCCATTAGTATTCTAGTGGGCATGCCTATTCGAGCGTCATT-ACAA-
CCCTTAAG-CCCTTGTT-GCTTAGCG-TTGGGACT-CTACGGC------------------------
CTTAGGG--------CGTAGTTCCTTAAAGTTA-GTGGCGGAGTTAG-GG---------
TACACTCTAAGCGTAGTAGTT-A-------TCTTCTCGCTTTT-GCAG---TG---------------
GCCT-TAG-----------------------------CTGCTAGCCGTAAAA-----------------CCCCTA--------
-------TATTCT-----------
AGTGGTTGACCTCGGATTAGGTAGGAATACCCGCTGAACTTAAGCATATC--------

>H_crocopeplum_CBS_119004

CAGAGTTATTC-------------TAAA---------CTCCA------AACCC-TTT-GTGAACC-
TTAC-CAC--TGTTGCCTCGGCG-TAAGCT----G--
CAGTTACCC----TAT------------------A-----------------------GCT----------------ACCCTG-
TAACTATCTGGGA---

--GATCTACCCT---
----GT-----------------------AGCTACC---
CTGGAGTTACCCTGTAG--CTAT------------------------
------------AATGTA--ACTTGCA----------------------AAATAGCCCGCCG-AAGGACC--
AC------------TAAA--------CTCTTG-----TTTTACTA-CA--CTGTATCTCTGAATGCTTC-
A----ACT-------
AAATAATTAAAACTTTCAACAACGGATCTCTTGGTTCTGGCATCGATGAAGAAC
GCAGCGAAATGCGATAAGTAATGTGAATTGCAGAATTCAGTGAATCATCGAAT
CTTTGAACGCACATT-
GCGCCCATTAGTATTCTAGTGGGCATGCCTATTCGAGCGTCATT-TCAA-
CCCTTAAG-CCCTTGTT-GCTTAGCG-TTGGGAAT-CTACGAT------------------------
TTACAG--------CGTAGTTCCTTAAAGTTA-GTGGCGGAGTTAG-GG---------
CATACTCTAAGCGTAGTAATTTT-------CTATCTCGCTTTT-GTAG---TA---------------
GCCT-TGG---------------------------CTTCTTGCCGTAAAG----------------CCCCCTA-------
----

>H_haematostroma_MUCL_53301

ATCATTACAGAGTTATCC----------------AA----------CTCCC------AACCC-TTT-
GTGAACC-TTAC-CGC--AGTTGCCTCGGCG-CGAGCC----G----------------------------------
---------------CGGCTACCC----TGC-------------------A-------------------------GCT-----------------
ACCCTG---

--TAGCTGCCGC--
--GTA--
GCACGCA-------------------------CATGGCCCGCCG-GTGGACA--GC-------------TAAA------
--CTCTTG-----T-ATGTAC-AC--AAGTATGTCTGATTGCTTA-A----ATA------
AAATAAGTCAAAACTTTCAACAACGGATCTCTTGGTTCTGGCATCGATGAAGA
ACGCAGCGAAATGCGATAAGTAATGTGAATTGCAGAATTCAGTGAATCATCGA
ATCTTTGAACGCACATT-
GCGCCCATTAGTATTCTAGTGGGCATGCCTATTCGAGCGTCATT-TCAA-
CCCTTAAG-CCTCTGTC-GCTTAGCG-TTGGGAGC-CTACGTC---------------------------
TACAG--------CGTATCTCCTCAAAGTCA-GTGGCGGTGCTGG-AG---------
CACACTCTCAGCGTAGTAGTT-T-------TCTTCTCGCTTCT-GTAG---TG----------------
GCCC-CTG------------------------------CAGCCTGCCGTAA--

>H_lateripigmentum_MUCL_53304

------------------------------------AAACC-TTT-GTGAACT-TAAC-CGT--
CGTTGCCTCGGCG-TGAGCT----G--CGGCTACCC-
---GGT--------------------A-------------------------GCT------------------ACCCTG--------------------------
----

---TAGCTACCCT--
--GTA--GCCGGTT------------------------
CACGGCCCGCCG-AAGGACA--GC------------TAAA--------CTCTTG-----TTAATTAC-
CA--CTGTATCTCTGAATTGTCA------ACT------
AAATAAGTTAAAACTTTCAACAACGGATCTCTTGGTTCTGGCATCGATGAAGA
ACGCAGCGAAATGCGATAAGTAATGTGAATTGCAGAATTCAGTGAATCATCGA
ATCTTTGAACGCACATT-
GCGCCCATTAGTATTCTAGTGGGCATGCCTATTCGAGCGTCATT-TCAA-
CCCTTAAG-CCAATGCT-GCTTAGTG-TTGGGAGC-ATACCCT------------------------
CCCCGGG--------GGTATCTCCTTAAAGTTA-GTGGCGGAGTTAG-GG---------
CACACTCTCAGCGTAGTAATT----------TCTCTCGCTCGG------------------------------------
----

>H_hinnuleum_MUCL_3621

TAGAGGAAGTAAAAGTCGTAACAAGGTCTCCGTTGGTGAACCAGCGGAGGGAT
CATTACAGAGTTACCT-------------AAAC---------TCCCA------AACCC-TTT-
GTGAACC-TTAC-CAC--CGTTTCCTCGGCG-CGAGCT----G------------------------------
-------------CGGCTACCC----GGG-----------------A---------------------GTT---------------
ACCCTG-CAGC---

---TACCCT---
----GTA---
GGGGACCTACCCT---
--------GTA--GCCGGCC---------------------CACGGCCCGCCG-AAGGACC--GC---------
--TAAA--------CTCTTG-----T-TTGAAC-CA--CTGTATCTCTGAACACCTA------ACT---
--
GAAATAAGTTAAAACTTTCAACAACGGATCTCTTGGTTCTGGCATCGATGAAG
AACGCAGCGAAATGCGATAAGTAATGTGAATTGCAGAATTCAGTGAATCATCG
AATCTTTGAACGCACATT-
GCGCCCGCTAGTATTCTGGCGGGCATGCCTATTCGAGCGTCATT-
TCGACCCCTTAAG-CCCCTGTT-GCTTAGCG-TTGGGACT-CTGCGCC----------------
-------TCAGGG--------CGCAGTTCCCGAAAGTTA-GTGGCGGAGTTAG-GG---------
TACACTCTCAGCGTAGTAATC---------TCTTCTCGCTCGT-GTGG---TG--------------
GCCC-TGG---------------------------CTGCTGGCCGTTAAA---------------CACCCC--------
------
CCCGCCCCCGCGCGGACAAGTGGTTGACCTCGGATTAGGTAGGAATACCCGCT
GAACTTAAGCATATC---

>H_trugodes_MUCL_54794

TCTTGGTCATTTAGAGGAAGTAAAAGTCGTAACAAGGTCTCCGTTGGTGAACC
AGCGGAGGGATCATTACTGAGTTATCA--------------AAA---------CTCCC------
AACCC-TTT-GTGAACC-TTAC-CAC--TGTTGCCTCGGCG-TCAGCT----G--------------
-------------------------------CGGCTACCC----TGG------------------G---------------------
GTGCT----------------ACCCTG-GAGTG---

---------CACCTACCTG-------GTAGG----------------------TGCTACC---------------------------
------------------------------TGGTAGTTACCCT---
-------------------------------------GTA--GCCCGCG-----------------------TATAGCGCGCCG-
GTGGACC--AA------------TAAA--------CTCTGT-----T--TTTAC-CT--
GAGAATCTCTGAATGCTTC-A----ACT------
AAATTAGTTAAAACTTTCAACAACGGATCTCTTGGTTCTGGCATCGATGAAGAA
CGCAGCGAAATGCGATAAGTAATGTGAATTGCAGAATTCAGTGAATCATCGAA
TCTTTGAACGCACATT-
GCGCCCATTAGTATTCTAGTGGGCATGCCTATTCGAGCGTCATT-TCAA-
CCCTTAAG-CCTTAGTT-GCTTAGCG-TTGGGACT-CTGAGCC-----------------------

TTTACGG--------CCTAGTTCCTTAAAGTTA-GTGGCGGAGTTAT-AG---------
CACACTCTCAGCGTAGTAATT-T-------CTATCTCGCTTTT-GTGG---TG----------------
GCTG-TGG----------------------------CGACTTGCCGTAAAA-----------------CCCCTAAT----
----

>H_investiens_CBS_118183

GTAACAAGGTCTCCGTTGGTGAACCAGCGGAGGGATCATTACTGAGTTATCA---
------------AAA---------CTCCC------AACCC-TTT-GTGAACC--TAC-CGC--
CGTTGCCTCGGCG-TGAGCT----G---CGGCTACCC-
---TGG--------------------A-------------------------GCT-----------------ACCCTG-
GAGCTACCCTATA--
GCTACCCTG---

CACCTACCCT-------ATAGTTACCC--------------TATAGCTACCC-----------------------------
TGCAGCTACCCTA--------TAGTCGCTCCGGGGCTACCCT------------------------------------
---GTA--GCCGGCT-------------------------
TACGGCCCGCCG-AAGGACC--GC------------CAAA--------CTCTTG-----T-TTTTTG-
CA--CTGCATGTCTGAATTTTAA------ACT-------
AAATAATTAAAACTTTCAACAACGGATCTCTTGGTTCTGGCATCGATGAAGAAC

GCAGCGAAATGCGATAAGTAATGTGAATTGCAGAATTCAGTGAATCATCGAAT
CTTTGAACGCACATT-
GCGCCCATTAGTATTCTAGTGGGCATGCCTATTCGAGCGTCATT-ACGA-
CCCCTAAG-CCCCTGTT-GCTTAGCG-TTGGGAAT-CTAC------------------------------
GG--------CGTAGTTCCTTAAAATCA-GTGGCGGAGTTAG-GG---------
TACACTCTCAGCGTAGTAATT----------TCTCTCGCTCGT-GTGG---TG--------------
GCCT-TGG---------------------------CTGCTAGCCGTTAAA----------------CCCCCC--------
-----TATATCTT-----------
AGTGGTTGACCTCGAATTAGGTAGGAATACCCGCTGAACTTAAGCATA-----------

>H_eurasiaticum_MUCL_57720

GGAAGTAAAAGTCGTAACAAGGTCTCCGTTGGTGAACCAGCGGAGGGATCATT
ACTGAGTTCTTA--------------CAA---------ACTCC------AACCC-TTT-GTGAACC-
ATAC-CAA--TGTTGCCTCGGCG-CGAGCT----G--
CGGCTGCTT----GGT------------------A----------------------GGTT----------------ACCCCG-
TAGTC---

------------ACCTACC--

CGGTACCTACCCT--
-------GTAACGTCTGCG----------------------TACAAGCCCGCCG-AAGGACC--AC--------
----TAAA--------CTCTGT-----T----TGA-CA--GTGTAT-TCTGAATGCTTC-A----ACT----
---
AAATAGTTAAAACTTTCAACAACGGATCTCTTGGTTCTGGCATCGATGAAGAAC
GCAGCGAAATGCGATAAGTAATGTGAATTGCAGAATTCAGTGAATCATCGAAT
CTTTGAACGCACATT-
GCGCCCATTAGTATTCTAGTGGGCATGCCTATTCGAGCGTCATT-TCGA-
CCCTGAAG-CCCTAGTT-GCTTCGCG-TTGGGACT-CTACTGG-----------------------
CTACCC--------TGTAGTTCCCTAAAGACA-GTGGCGGAGTTCA-GG---------
TGTACTCTCAGCGTAGTAATT---------TCTTCTCGCTTTT-GCAG---TA--------------
GCC--TGG---------------------------TCACCAGCCGTTAAA----------------CCCCCT---------
-----ATTATCT-----------
AGTGGTTGACCTCGGATTAGGTAGGAATACCCGCTGAACTTAAGCATAT----------

>H_fuscum_CBS_113049

GGAAGTAAAAGTCGTAACAAGGTCTCCGTTGGTGAACCAGCGGAGGGATCATT
ACTGAGTTCTTA--------------CAA---------ACTCC------AACCC-TTT-GTGAACC-
ATAC-CAAC-TGTTGCCTCGGCG-TGAGCT----G--

CGGCTGCGT----GGT------------------A------------------------GCT-----------------ACCCGG-
TAGTC--

-------------ACCTACC--
CGGTACCTACCCT---
-------GTAGCGTCTGCG-----------------------TATAAGCCCGCCG-AAGGACC--AC--------
----TAAA--------CTCTGT-----T----TGA-CA--GTGTAT-TCTGAATGCTTC-A----ACT----
---
AAATAGTTAAAACTTTCAACAACGGATCTCTTGGTTCTGGCATCGATGAAGAAC
GCAGCGAAATGCGATAAGTAATGTGAATTGCAGAATTCAGTGAATCATCGAAT
CTTTGAACGCACATT-
GCGCCCATTAGTATTCTAATGGGCATGCCTATTCGAGCGTCATT-TCGA-
CCCTGAAG-CCCTGGTT-GCTTCGCG-TTGGGACT-CTACTGG-------------------------
CTACCC--------TGTAGTTCCCTAATGACA-GTGGCGGAGTTCA-GG---------
TGTACTCTCAGCGTAGTAATT---------TCTTCTCGCTTTT-GCAG---TA----------------
GCC--TGG------------------------------TCACCAGCCGTAAAA-----------------CCC---------------
---TTTTCT--------------
AGTGGTTGACCTCGGATTAGGTAGGAATACCCGCTGAACTTAAGCATAT----------

>H_pseudofuscum_DSM112038

GTCGTAACAAGGTCTCCGTTGGTGAACCAGCGGAGGGATCATTACTGAGTTCTT
A--------------CAA---------ACTCC------AACCC-TTT-GTGAACC-ATAC-CAAC-
TGTTGCCTCGGCG-CGAGCT----G---CGGCTGCTT-
---GGT------------------A-----------------------GCT----------------ACCCGG-TAGTC------------
----

ACCTACC---CGGGAGCTACCCG------------
---GTAGCGTCTGCG-------
--------------TACAGGCCCGCCG-AAGNNCC--AC------------CAAA--------CTCTGT-----
T----TGA-CA--GTGTAT-TCTGAATGCTTC-A----ACT-------
AAATAGTTAAAACTTTCAACAACGGATCTCTTGGTTCTGGCATCGATGAAGAAC
GCAGCGAAATGCGATAAGTAATGTGAATTGCAGAATTCAGTGAATCATCGAAT
CTTTGAACGCACATT-
GCGCCCATTAGTATTCTAGTGGGCATGCCTATTCGAGCGTCATT-TCGA-
CCCTGAAG-CCCTAGTT-GCTTCGCG-TTGGGACT-CTACTGG------------------------
CTACCC--------TGTAGTTCCCTAATGACA-GTGGCGGAGTTCA-GG---------
TGTACTCTCAGCGTAGTAATT---------TCATCTCGCTTTT-GCAG---TA---------------
GCC--TGG----------------------------TCGCCGGCCGTAAAA----------------CCCCCT---------
-----ATTTTCT-----------AGTGG--

>H_fuscoides_MUCL_52670

AAAGTCGTAACAAGGTCTCCGTTGGTGAACCAGCGGAGGGATCATTACTGAGT
TCTTA-------------CAA--------ACTCC------AACCC-TTT-GTGAACC-ATAC-CAAC-
TGTTGCCTCGGCG-TGAGCT----G---CGGCTGCCT-
---GGT-----------------A---------------------GCT---------------ACCCGG-TAGTC-----------
----

GCCTACC--CGGTAGCTACCCT------------
---GTAGCGTCTGCG------
-------------TACAGGCCTGCCG-AAGGACC--AC-----------CAAA--------CTCTGT-----
T----TGA-CA--GTGTAT-TCTGAATGCTTC-A----ACT-----
AAATAGTTAAAACTTTCAACAACGGATCTCTTGGTTCTGGCATCGATGAAGAAC
GCAGCGAAATGCGATAAGTAATGTGAATTGCAGAATTCAGTGAATCATCGAAT
CTTTGAACGCACATT-
GCGCCCATTAGTATTCTAGTGGGCATGCCTATTCGAGCGTCATT-TCGA-
CCCTGAAG-CCCTAGTT-GCTTCGCG-TTGGGACT-CTACTGG----------------------
CTACCC---------TGTAGTTCCCTAATGACA-GTGGCGGAGTTCA-GG--------
TGTACTCTCAGCGTAGTAATT---------TCTTCTCGCTTTT-GCAG---TA---------
GCC--TGG-------------------------TCGCCGGCCGTAAAA--------------CCCCCT--------
-----ATTTTCT--------
AGTGGTTGACCTCGGATTAGGTAGGAATACCCGCTGAACTTAAGCATATCA-----

>H_porphyreum_CBS_119022

GGAAGTAAAAGTCGTAACAAGGTCTCCGTTGGTGAACCAGCGGAGGGATCATT
ACTGAGTTCTTA-------------CAAA---------CTCCC------AACCC-TTT-GTGAACC-
ATAC-TGT--CGTTGCCTCGGCG-CGAGCT----G---
CGGCTACCC----TGC-----------------A----------------------GCT---------------ACCCTG-
TAGTC---

-------------GTCTACC--
CGGTAGCTACCCT---
-------GTAGTCTGCGTA----------------------CAAGGCCCGCCG-GAGGACC--AT---------
---TCAA--------CTCTGT-----T-TTGTTA-CG--GTGTAT-TCTGAATGCTTCAA----ACT-
-----
ATAATAGTTAAAACTTTCAACAACGGATCTCTTGGTTCTGGCATCGATGAAGAA
CGCAGCGAAATGCGATAAGTAATGTGAATTGCAGAATTCAGTGAATCATCGAA
TCTTTGAACGCACATT-
GCGCCCATTAGTATTCTAGTGGGCATGCCTATTCGAGCGTCATT-TCAA-
CCCTGAAG-CCCTGGCT-GCTTCGCG-TTGGGACC-CTACAGG--------------------
TACCTACCC--------TGTAGTTCCCTAAAGGCA-GTGGCGGAGTTCG-GG---------
TATACTCTCAGCGTAGTAGTT---------TCTTCTCGCTGTG-GCAG---TA--------------
GCCC-TAG---------------------------CTATCAGCCGTAAA--------------------------------------
----

>P_laminosus_MUCL_53305

GGAAGTAAAAGTCGTAACAAGGTCTCCGTTGGTGAACCAGCGGAGGATCATT
ACTGAGTTGTCA-------------AAAA---------CTCCA------AACCC-TTT-GCGAACC--
TAT-AAT--CGTTGCCTCGGCG-TGAGCC----G--
CGCGTGCCC----CGC-----------------A----------------------GCT---------------GCTGCG-
GGGT--

---ACGCGCA------------------
--AGACGGCCCGCCG-GAGGACC--AATT---------CAAAA--------CTCTTG----
CTCCAAAAG-CT--TTGCTTCTCTGAATTATTGCT----TCA------
AAATCAGTTAAAACTTTCAACAACGGATCTCTTGGTTCTGGCATCGATGAAGAA
CGCAGCGAAATGCGATAAGTAATGTGAATTGCAGAATTCAGTGAATCATCGAA
TCTTTGAACGCACATT-
GCGCCCATTAGTATTCTAGTGGGCATGCCTATTCGAGCGTCATT-TCGA-
CCCTTAAG-CCCCAGTT-GCTTAGCG-TTGGGAGT-CTGCGTC---------------------
CTTCGGG--------CGCAGTTCCTCAAAGTCA-GTGGCGGAGTTAG-GT---------
CACACTCTAAGCGTAGTAAAT---------CGTTCTCGCTTCT-GTGG---TG--------------
TACC-TAG--------------------------CTGCCTGCCGTAAAA---------------CCCCTA--------
------TACTTCT-----------
AGTGGTTGACCTCGAATTAGGTAGGAATACCCGCTGAACTTAAG------------------
----

>H_hypomiltum_MUCL_51845

TAGAGGAAGTAAAAGTCGTAACAAGGTCTCCGTTGGTGAACCAGCGGAGGGAT
CATTACAGAGTTACTA------------TAAAA---------CTCCC------AACCC-TTT-
GTGAACC-TTAC-CGT--CGTTGCCTCGGTG-GAAGGTG---G-------------------------------
--------------TGTGCGGTG----GGA------------------A-----------------------GCT----------------
ACCCTG-GAGC---

TACCTACCCT-------GTAGAT---------------------AGCTACC------------------------------------
---------------------CTGGAGCTACCCT---
----------------------------GAA-AATACGCC--------------------CCCCGCCAGCCGCCG-
AAGGACC--AC------------TAAA--------CTCTTG-----T--TTTTA-CT--
GTGTCTCTCTGAATAATGA-A----ACA-----
AAAATTCGTTAAAACTTTCAACAACGGATCTCTTGGTTCTGGCATCGATGAAGA
ACGCAGCGAAATGCGATAAGTAATGTGAATTGCAGAATTCAGTGAATCATCGA
ATCTTTGAACGCATATT-
GCGCCCATTAGTATTCTAGTGGGCATGCCTATTCGAGCGTCATT-TCAA-
CCCTTAAGCACCTATCT-GCTTAGCG-TTGGGAGT-CTGCGCC--------------------------
TCGG--------CGCAGTTCCCTAAAGGTA-GTGGCAGTGTTA--GG---------
TACACTCGTAGCGTAGTAATT-T-------TCTTCTCGCGTCT-GTAG---TG---------------
GTCC-TGAA---------------------------CGTCTAGCCGTTAAA----------------CCCCCC-------
-------TATTTCT-----------
CAAGGTTGACCTCGGATTAGGTAGGAATACCCGCTGAACTTAAGCATATCA-----

>H_isabellinum_MUCL_53308

TTCTAC------------AAAAA---------CTCCC------AACCC-TTT-GTGAACC-TTAC-CTA--
TGTTGCCTCGGCGCCGAGCA----G---
CAGCTACCC----TGGAG----------------A-----------------------GCT----------------ACTTTG-
AAGCTACCT--
ACCCTGTAGAAACATA---

----CATCTACCCT-------GTAGTG---------------------AGCTACC---------------------------------
--------------------------CTGGAGCTACCCC--
---------------------------------GGA-GTTGCATT-----------------------TACGCTCCGCCG-
ATGGACC--AG------------TAAA--------CTCTGT-----TTTTTTCC-CA--
GTATATCTCTGAATTCTTT-A----ACA------
AAAATAGTTAAAACTTTCAACAACGGATCTCTTGGTTCTGGCATCGATGAAGA
ACGCAGCGAAATGCGATAAGTAATGTGAATTGCAGAATTCAGTGAATCATCGA
ATCTTTGAACGCATATT-
GCGCCCAGTAGTATTCTACTGGGCATGCCTATTCGAGCGTCATT-TCAA-
CCCTTATG--CCCTGTA-GCATAGTG-TTGGGGCT-CTAC---------------------------
TGAAA--------GGTAGTCCCCGAAAACCA-GTGGCGGTGTTC--GG---------
TACACTCATAGCGTAGTAATT----------TTTCTCGCTTCT-GACG---TG--------------
GCCT-GAA-----------------------------TCTTCGG--

>H_perforatum_CBS_115281

GTAACAAGGTCTCCGTTGGTGAACCAGCGGAGGGATCATTACTGAGTTCTAC---
---------AAAAA---------CTCCC------AACCC-TTT-GTGAATT-ATAC-CTT--
AGTTGCCTCGGCGTCGAGCGA---G---------------------------------TC----------
AACCTACCC----GGG------------------A-----------------------GCT----------------ACCCTG-
GAGTCACCT---
ACCCTGTAGA-ACCTA---

CCCTGTAG--GACCTACCCT-------GTAGAC---------------------GGCTACC-----------------
--CTGGAGCTACCCT---
--GTA-GTTGCACT-----------------------
TTCGCGTCGCCG-GTGGACT--AC------------CAAA--------CTCTTA-----T--ATGTA-
TA--GTGTATCTCTGAATTCTTA------ACA------
AAATTAGTTAAAACTTTCAACAACGGATCTCTTGGTTCTGGCATCGATGAAGAA
CGCAGCGAAATGCGATAAGTAATGTGAATTGCAGAATTCAGTGAATCATCGAA
TCTTTGAACGCATATT-
GCGCCCAGTAGTATTCTACTGGGCATGCCTATTCGAGCGTCATT-TCAA-
CCCTTACG--CCCTGTA-GCGTAGTG-TTAGGACT-CTACTCT------------------------

TTAGAG--------AGCAGTTCCCTAAAACCA-GTGGCAGTGTTC--GG---------
TACACTCATAGCGTAGTAATT----------CTTCTCGCTTCT-GCAG---TG--------------
GCCC-GTA----------------------------CTACTCGCCGTAAAA----------------CCCCTA--------
------ATTTTCT-----------
AATGGTTGACCTCGGATTAGGTAGGAATACCCGCTGAACTTAAGCAT-------------

>H_sporistriatatunicum_UCH9542

TCTCCGTTGGTGAACCAGCGGAGGGATCATTACTGAGTTCTAC------------CAAA-
--------CTCCC------AACCC-TTT-GTGAATC-GTAT-TCC--
TGTTGCCTCGGCGGCCAGCA----G---
TACCTATTC----GAT-----------------A----------------------GCT-------------ACCCTG-
GAGTCACCT---
ACCCTGGAGGCGTCTA--

CCCTGTAGATGACCTACCCT-------GGAGGT--------------------GGCTACC---------------
--CTGGAGCTACCCT---
---GTA-GTTGCACG----------------------
AATGCCCCGCCG-GTGGACC--AC------------CCAA--------CTCTGA--------TTTTA-TA-
-GTGTATCTCTGAATTCTTT-A----AC-------

AAATTAGTTAAAACTTTCAACAACGGATCTCTTGGTTCTGGCATCGATGAAGAA
CGCAGCGAAATGCGATAAGTAATGTGAATTGCAGAATTCAGTGAATCATCGAA
TCTTTGAACGCATATT-
GCGCCCAGTAGTATTCTGCTGGGCATGCCTATTCGAGCGTCATT-TCAA-
CCCTTACG--CCCTGCA-GCGTAGCG-TTGGGACT-CTACTCC-----------------------
TCCGGG--------GGCAGTTCCCTAAAACCA-GTGGCGGTGTTC--GG---------
TACACTCATAGCGTAGTAAAT---------CATCCTCGCTTCT-GTAG---TA--------------
TACC-GTC----------------------------CTATCGGCCGTAAAA----------------CCCCCT--------
------ATATTCT-----------
AATGGTTGACCTCGGATTAGGTAGGAATACCCGCTGAACTTAAGCATATCAAT
AAGCGGAGGAA--

>X_arbuscula_CBS_126415

AACAAGGTCTCCGTTGGTGAACCAGCGGAGGGATCATTAAAGAGTTAAAC------
---------AA---------CTCCT------AAACC-CAT-GTGAACC--TAC-CTT--
TGTTGCCTCGGCA--GGTCT----G--CAACTTACCC--
--GGA------------------G-------------------GGGACCT----------------ACCCTG-TA----------------
----

---GGGACCTTACCCG----------------------------
---GTA-GTTGCCGG------------------------
CATAACCTGCCG-GTGGTCT--AC------------TAAA--------CTCTGT-----T-----TA-CT--
ATGTTATTCTGAATAATAT-A----ACT------
AAATAAGTTAAAACTTTCAACAACGGATCTCTTGGTTCTGGCATCGATGAAGA
ACGCAGCGAAATGCGATAAGTAATGTGAATTGCAGAATTCAGTGAATCATCGA
ATCTTTGAACGCACATT-
GCGCCCATTAGTATTCTAGTGGGCATGCCTGTTCGAGCGTCATT-TCAA-
CCCTTAAG--CCCTGTT-GCTTAGCG-TTGGGAGC-CTACAGA----------------------
TACCCTC--------TGTAGTTCCTTAAAGTTA-GTGGCGGAGTCGGTTT---------
CACACTCTAGACGTAGTAAAT-T-------TTATCTCGCCT---ATAG---AT--------------
GAGC-CGG---------------------------TCCCTTGCCGTAAAA----------------CCCCCT--------
------AATTTCT-----------
AAAGGTTGACCTCGGATCAGGTAGGAATACCCGCTGAACTTAAGCATATC-------

>X_hypoxylon_CBS_122620

AGGAAGTAAAAGTCGTAACAAGGTCTCCGTTGGTGAACCAGCGGAGGGATCAT
TAAAGAGTTAATT-------------ACAA---------CTCCC------AAACC-CAT-GTGAACT-

TACC-TTC--TGTTGCCTCGGCA---GGTC----G--
TGTTTACCC----TGT------------------G-------------------AGGTCCT----------------
ACCCTGT---

AGGACCCTACCTG---
--------GTA-GACACG------------------------GGTACGCCTGCCG-GTGGCCC--AT----------
--GAAA--------CTCTGT--------TAATT-CT--ATGTTATTCTGAATCTATA------ACT-----
-
AAATAAGTTAAAACTTTCAACAACGGATCTCTTGGTTCTGGCATCGATGAAGA
ACGCAGCGAAATGCGATAAGTAATGTGAATTGCAGAATTCAGTGAATCATCGA
ATCTTTGAACGCACATT-
GCGCCCATTAGTATTCTAGTGGGCATGCCTGTTCGAGCGTCATT-TCAA-
CCCTTAAG-CCCCTGTT-GCTTAGCG-TTGGGAGC-CTACAGCCTTC------------------
---------------TGTAGCTCCCCAAAGTTA-GTGGCGGAGTCGG-TT---------
CACACTCTAGACGTAGTAGAT-T-------CTATCTCGTCT---GTAG---TG--------------
AGGC-CGG----------------------------TCCCCTGCCGTAAAA----------------CCCCCC--------
-----TAATTTTT-----------
AAAGGTTGACCTCGGATCAGGTAGGAATACCCGCTGAACTTAAGCATAT---------

>H_erythrostroma_MUCL_53759

TTACAGAGTTACCA---------------AAC--------TCCAA------AACCC-TTT-GTGAACC-
TTAC-CAC--TGTTTCCTCGGCG-AGGTATT--
CAGGCCCTC----AGC------------------G------------------------GCC---------------------------------
----

GAGAACCCGCCG-A-AGACC--AC------------TAAA--------CTCGTG-----T--TTGTT-
TA--GCGTATCTGAATGCTTCTA-A----AAC------
AAATAAGTTAAAACTTTCAACAACGGATCTCTTGGTTCTGGCATCGATGAAGA
ACGCAGCGAAATGCGATAAGTAATGTGAATTGCAGAATTCAGTGAATCATCGA
ATCTTTGAACGCACATT-
GCGCCCATTAGTATTCTAGTGGGCATGCCTATTCGAGCGTCATT-TCAA-
CCCTTAAG--CCCTGTT-GCTTAGCG-TTGGGANT-CTGCGTC-----------------------
TTAGGG--------CGCAGCTCCCTAAAGTTA-GTGGCGGAGTCGG-AG---------
CACACTCTGAGCGTAGTAATT-C-------TGTTCTCGCTTCT-GTGG---TG---------------
GCTT-TGG-----------------------------CGACCTGCCGTAAAA-------------------------------------
----

>H_canariense_MUCL_47224

AACAAGGTCTCCGTTGGTGAACCAGCGGAGGGATCATTACTGAGTTCTAT--------
-----AAAA---------ACTCC------TACCC-TTT-GTGAACC-TTAC-CGT--
CGTTGCCTCGGCGCCGAGCG----GCTTCT----------CAA-GAGATCTC------T------
GGGGGAACCTACCC----TGGAGAAG----TCTGGGAGAA---------------------GCT------
----------ACCCTG-TAGAACTTT---

AAGAGCTCCTACCCT-------GGAGAACTTT-----------GGGAGCTCCTACC-------------
---CTGGAGCTACCCT---
--GTA-GTTGCAT-----------------------
CACGCTCCGCCG-GTGGACC--TC------------TACA--------CTCTGT-----T--TTGTA-TA-
-GTGTATCTCGGAAAACCAT-A----ACT------
TAATACGTTAAAACTTTCAACAACGGATCTCTTGGTTCTGGCATCGATGAAGAA
CGCAGCGAAATGCGATAAGTAATGTGAATTGCAGAATTCAGTGAATCATCGAA
TCTTTGAACGCATATT-
GCGCCCATTAGTATTCTAGTGGGCATGCCATTTCGAGCGTCATT-TCGA-
CCCTTAAG--CCCTGCT-GCTTAGCG-TTGGGAAC-CTACGGGGCC------------------
TACGGGCCC--------TGTAGCTCCCTAAAGGTA-GTGGCGGTGTTA--GG---------
TACACTCGTAGCGTAGTAAATCT-------TTTTCTCGCTCCT-GCGG---TG------------
TACC-TGA----------------------------GGCCTGCCGTGAAAA---------------CCCCTTA------
-----TAAATTTAC-----------
ATGGTTTGACCTCGGATTAGGTAGGAATACCCGCTGAACTTAAGCATATCA------

>H_lenormandii_CBS_119003

TCATTACAGAGTTACTA---------------AA---------CTCCC------AACCC-TAT-
GTGAACC--TAC-CAC--TATTGCTTCGGCG---GGCT----G----------------------------------
----------CTGCTACCC----TGT-----------------A----------------------GCC---------------
ACCCTG-TA---

---------------------------ATTCAGG---
GTGGGCTTACCCG---
-------GTA--GTTTGCG----------------------TCAAGGCCCGTCA-GAGGACC--AC----------
--TAAA--------CTCTTG-----T-TACCCT-GT--ACGTATTTCTGAATGCTTC-A----ACT--
----
TAATAAGTTAAAACTTTCAACAACGGATCTCTTGGTTCTGGCATCGATGAAGAA
CGCAGCGAAATGCGATAAGTAATGTGAATTGCAGAATTCAGTGAATCATCGAA
TCTTTGAACGCACATT-
GCGCCCATTAGTATTCTAGTGGGCATGCCTATTCGAGCGTCATT-TCAA-
CCCTTAAG--CCCTGTT-GCTTAGCG-TTGGGAA--CTGCGCC-----------------------
ATACGG--------CG-AGCTCCTTAAAGTTA-GTGGCGGAGTTAG-TG---------
TATACTCTTAGCGTAGTAAAT----------ATCCTCGCTCAC-GTAG---TA----------------
---CGC---

>H_rickii_MUCL_53309

GGAAGTAAAAGTCGTAACAAGGTCTCCGTTGGTGAACCAGCGGAGGGATCATT
AAAGGGTCGCGTGCATTGCTCGCGGCTTA---------CTGCC-----AAACCC-TAT-
GTGAAC--TTAC-CAC--TGTTGCCTCGGCG--GCGCC----G----------------------------------
-----------CGGCTACCC----TGGAG----------------A-----------------------GCT----------------
ACCCTG-TAGC--

CATTTACCCT-------GTAGAT---------------------GGCTACC------------------------------------
---------------------CTGTAGCTACCCT--
----------------------------GTAGCTCGCACG----------------------TAAAGGCCCGTCA-
AAGGACC---------------CTAAA--------CTCTAT--------TTTGA-CT--
TGGTATCTCTGAGTAAATC-A----ACT------
TAATAAGTTAAAACTTTCAACAACGGATCTCTTGGTTCTGGCATCGATGAAGAA
CGCAGCGAAATGCGATAAGTAATGTGAATTGCAGAATTCAGTGAATCATCGAA
TCTTTGAACGCACATT-
GCGCCCATTAGTATTCTAGTGGGCATGCCTATTCGAGCGTCATT-
TCAACCCCTTAAG--CCCTGTT-GCTTAGCG-TTGGGGGC-CTGCGCC------------------
------TGGCGG--------CGTAGCCCCTGAAAGATA-GTGGCGGAGTCAG-TG---------
AGCACTCTGAGCGTAGTAACT----------TTTCTCGCTCCG-GTAG-CTTG---------------
CCCC-TGG----------------------------CTGCTGGCCGTTAAA----------------CCCCCC--------
---CATATCTTCT-----------
AGTGGTTGACCTCGGATTAGGTAGGAATACCCGCTGAACTTAAGCATAT----------

>H_lividipigmentum_BCRC_34077

TCCGTAGGTGAACCTGCGGAGGGATCATTACTGAGTTGCTT------------TAAAA----
-----CTCCA-----AAACCCTTAT-GTGAAC--ATAC-CAT--AGTTGCCTCGGCG-
TAAGCC----G--CACTGCCCC----GGC------------------
A-----------------------GCC----------------GCCCCG---

------------------------CGCGGCCGCCTG---
-------------------------------GCA--GCGCATT-----------------------CAAGGCCCGCCG-
AAGGACC--GC------------CAAAA-------CTCTTG----TTTAATTTA-AA--
ACGTTTCTCTGAATGCTTC-A----ACT-----
GAAATAAGTTAAAACTTTCAACAACGGATCTCTTGGTTCTGGCATCGATGAAG
AACGCAGCGAAATGCGATAAGTAATGTGAATTGCAGAATTCAGTGAATCATCG
AATCTTTGAACGCACATT-
GCGCCCATTAGTATTCTAGTGGGCATGCCTATTCGAGCGTCATT-
TCAACCCCTTAAG--CCCTGTT-GCTTAGCG-TTGGGTAT-CTACGGG-------------------
----CTTAGGCG--------CGTAGTTCCTGAAAATTA-GTGGCGGAGCCG--AG---------
CGTGCCGCGAGCGTAGTAATT-C-------ATATCTCGCCCGT-GTGG---TG--------------
CCCT-CGG-----------------------------TTGCCGGCCGTAAAA----------------CCCCTT--------
----ATATTTCTT-----------
AGTGGTTGACCTCGGATTAGGTAGGAATACCCGCTGAACTTAAGCATATCAAT
AAGCGGAGGA---

--

>H_lividipigmentum_STMA_14045

GAACAAGGTCTCCGTTGGTGAACCAGCGGAGGGATCATTACCGAGTTCT--------
-------AAA---------CTCCC-----AAACCC-TAT-GTGAAC--GTAC-CGC--
AGTTGCCTCGGCG-TGAGCC----G---CACTGCCCT-
---GGT-----------------G----------------------GCC---------------GCCCTC-----------------------
----

---GCGCGGCCGCCTG-------------------------------
--GCG--GCGCACT---------------------
CAAGGCCCGCCG-AAGGACC--AC------------TAAA--------CTCTGT-----TTTAAATA-
TA--ACGTTTCTCTGAATGCTTCAA----ACT-----
AAAATAAGTTAAAACTTTCAACAACGGATCTCTTGGTTCTGGCATCGATGAAG
AACGCAGCGAAATGCGATAAGTAATGTGAATTGCAGAATTCAGTGAATCATCG
AATCTTTGAACGCACATT-
GCGCCCATTAGTATTCTAGTGGGCATGCCTATTCGAGCGTCATT-
TCAACCCCTTAAG--CCCTGTT-GCTTAGCG-TTGGGAAT-CTACCGG-----------------
----GTACCCCG--------CGTAGTTCCCTAAAACCA-GTGGCGGAGCCC--AG---------
GGTGCCGCGAGCGTAGTAATC-T-------TTATCTCGCTTTT-GCAG---TT--------------
CCCT-GTG---------------------------TTGCCGGCCGTAAAA----------------CCCCCC--------

------CATATTT-----------
TCTGGTTGACCTCGGATTAGGTAGGAATACCCGCTGAACTTAAGCATATCT------

>H_ferrugineum_CBS_141259

TCCGTAGGTGAACCTGCGGAGGGATCATTACAGAGTTACCT--------------AAAC---
-----TCCAA------AACCC-TAT-GTGAACC-TTAC-TATC-AGTTTCCTCGGCG-
TCGCCG----C--GGGCTACCC----TGT-------------------
A------------------------GCT----------------ACCCTG-TAGA--

------------------------------GACCTCC--------------------------------------ATCTACC----------------
--CGGCAGCTACCCT--
---GTAGCCCGCGCT-----------------------
GAAGGCCCGCCG-CTGGACC--AC------------TAAA--------CTCTTG-----T-TTTTA-
CA--GTGTATCTCTGAATGCTTCTAA---ACA-----
AAAATAAATTAAAACTTTCAACAACGGATCTCTTGGTTCTGGCATCGATGAAG
AACGCAGCGAAATGCGATAAGTAATGTGAATTGCAGAATTCAGTGAATCATCG
AATCTTTGAACGCACATT-
GCGCCCACTAGTATTCTGGTGGGCATGCCTATTCGAGCGTCATT-TCAA-

CCCTTAAG-CCCCTGTT-GCTTAGCG-TTGGGAAT-CTACGTC----------------------
CTTCAGGG--------CGTAGTTCCTTAAAATTA-GTGGCGGAGTCGG-TG---------
CATACTCTAGGCGTAGTAACTAAATCTTTTTTTACCAGCCTCT-GTAG--TTG-------
--------TACC-TGG----------------------------CTTCCGGCCGTTAAA----------------
GCCCCTATA---------AATTTTTTC-----------
AATGGTTGACCTCGGATTAGGTAGGAATACCCGCTGAACTTAAGCATATCAAT
AAGCGGAGGA--

--

>H_baruense_UCH9545

CCGTTGGTGAACCAGCGGAGGGATCATTACAGAGTATTAC---------------AA--------
-CTCAT------AACCC-TAT-GTGAACC-TTAC-CAC--CGTTTCCTCGGCG-CGTGCC---
-G---CGGCTGCGT----TGC------------------G-------------
-----GGAGGGCG--------------GCGCTCCG-TCG--

------------------CCCCTCCCGCCCC---
-------------------------GGAGCTCGCGCC----------------------TCAAGGCCCGCCG-
GCGGACC--CC------------CAAA--------CTCTTG--------TCATG-CA--

GTGGAATTCTGAATGCTTA-A----CTA------
AAATAAGTTAAAACTTTCAACAACGGATCTCTTGGTTCTGGCATCGATGAAGA
ACGCAGCGAAATGCGATAAGTAATGTGAATTGCAGAATTCAGTGAATCATCGA
ATCTTTGAACGCACATT-
GCGCCCATTAGCATTCTAGTGGGCATGCCTATTCGAGCGTCATT-
TCAACCCCTTAAG--CCTCGTC-GCTTAGCG-TTGGGGGC-CTGCCGC------------------
-----GGAAGCG--------CGCAGCCCCTGAAAACCA-GTGGCGGAGTCGG-TG---------
AGCACTCTGAGCGTAGTAACT-T---------CTCTCGTTCCT-GTAGTCTTG---------------
CTCC-CGG-----------------------------CGGCCTGCCGTGAAA-----------------CCCCCA--------
----TATACTTCT-----------
AATGGTTGACCTCGGATTAGGTAGGAATACCCGCTGAACTTAAGCATATCAAT
AAGCGGAGGAA---

>H_subticinense_MUCL_53752

TATAGAGTTCTAA-------------ACAGA---------CTCCC----AAAACCC-TGT-GTGAACC-
-TAC-CGCA-TGTTGCCTCGGCG--GCGCC----G---
CGGCTACCC----TGG--------------------A--------------------------GCT-----------------ACCCTG-
GAGCTA---
CCCTGTAGA--

CGCCTACCCT-------GTAGGC-------------------GCCTGCC----------------------------
CTGTAGA-------------CCTCCCGGCGCAGCCACCCG--
--GGAGCTCGCGCT----------------------
AAAGGCCCGCCA-AAGGACC--CC------------CTAA--------CTCTGT-----T--TTATT---
T-TGGCTATTCTGAACGTATC-A----ACTT---
TAAAATGAATCAAAACTTTCAACAACGGATCTCTTGGTTCTGGCATCGATGAAG
AACGCAGCGAAATGCGATAAGTAATGTGAATTGCAGAATTCAGTGAATCATCG
AATCTTTGAACGCACATT-
GCGCCCATTAGTATTCTAGTGGGCATGCCTATTCGAGCGTCATT-
TCAACCCCTTAAG--CCCTGTT-GCTTAGCG-TTGGGAGC-CTGCGGC-----------------
------CTGCGC--------CGCAGCTCCTTAAATTCA-GTGGCGGACCGG--TG---------
AGCACCCTGAGCGTAGTAATC------------TCTCGCTTCT-GGAG---GC--------------
TCCC-CGG---------------------------CGGCCGGCCGTG---

>Rhopalostroma_angolense_CBS_126414

------------A---------CTC-C------CACCC-TTT-GCGAACC-TTAC-CAC--
TGTTGCCTCGGCG-GGACGC----G--

--C--

------------------------GGAGAACGCGCT-------------------------ACAGGCCCGCCG-
GCGGACT--GC------------TATA--------CATTCT----GT-CACGTG-TA--
ACGTAACTCTGAATGCTTC-A----AAC------
AAATAAGTTAAAACTTTCAACAACGGATCTCTTGGTTCTGGCATCGATGAAGA
ACGCAGCGAAATGCGATAAGTAATGTGAATTGCAGAATTCAGTGAATCATCGA
ATCTTTGAACGCACATT-
GCGCCCGTTAGTATTCTAGCGGGCATGCCTGTTCGAGCGTCATT-ACGA-
CCCTTAAG--CCCTGTA-GCTTAGCG-TTGGGAAC-
CTAGGTTTCTCCCCCCCCCCCTCGGGGGGTGTGTGTG--------
CCCAGCTCCCTAAAGGTA-GTGGCGGAGTCGGGGT---------
CCTGCTCTGAGCGTAGTAGTA-T-------TCTTCTCGCTTCG-GTAG---TA--------------
GTCC-CGG-------------------------------CGGCCTGCCGTAAAA-----------------CAACCC-------
---CCTA---

>H_cyclobalanopsidis_FCATAS2714

TGGAAGTAAAAAATCGTAACAAGGTCTCCGTTGGTGAACCAGCGGAGGGATCA
TTACTGAGTTATCA-------------TAA--------ACTCC------AACCC-TAT-GTGAACC-
ATAC-TCT--TGTTGCCTTGGCG-TGCGCC----G--------------------------------------
GAAGGCTGTCG----GGT----------------A--------------------GCT--------------
ATCCTG-GAGGT---

CCTACCCG-------GTAGG--------------------ACCTACCC------------------------GG------
------------------CACTAGCTACCCG---
------------------------GGA--GCCTGCC--------------------ATATGGCCCGCCA-
GAGGACC--AAC----------CAAA-------CTCTGC-------TTCTA-CC--
GTGGGACTCTGAATTAGCA------ACT------
GAAATCGTTAAAACTTTCAACAACGGATCTCTTGGTTCTGGCATCGATGAAGAA
CGCAGCGAAATGCGATAAGTAATGTGAATTGCAGAATTCAGTGAATCATCGAA
TCTTTGAACGCACATT-
GCGCCCATTAGTATTCTAGTGGGCATGCCTATTCGAGCGTCATT-TCAA-
CCCTGAAG-CCCTCGTC-GCTTCGCG-TTGGGGAT-CTACAGG--------------------
ACCTACCC--------TGTAGGCCCCGAAAAGGA-GTGGCAGAGTTCG-GG---------
CATACTCTCAGCGTAGTAAAT---------CTTCCTCGCTCTT-GCAG---TA---------------
TCC-CGT-------------------------CCCCTCGCCGTAAAA---------------CCCCCC----------
--CTATTCTT----------CTAAGGTGACCTCGGATTAGGTAGAATGCCCCAAT---------
----

>H_hainanense_FCATAS2712

TGGAAGTAAAAGTCGTAACAAGGTCTCCGTTGGTGAACCAGCGGAGGGATCAT
TACCGAGTTGTCTAAA---------CGAAA---------CTCCC-----AAACCC-ATT-
GTGAACC-TTAC-CG---CGTTGCCTCGGCGGTGAGCT----G--------------------------------
-------------CGGCGCACC----TGGGA---------------GA----------------------GCTT--------------
-ACCCTG-TAGAA--

CTACCCT-------GAAGCTACCC--------------GGGAGCTACC----------------------------------
---------------------CTGGAGAGCTACCCT--
---------------------------------GGA-GCTGC-------------------------CGCAAGCCCGCCG-
GAGGACC--AC-----------CTAAA--------CTCTTG-----A-TTACGC-TG--
CAGAATTTCTGAATCATAA-A----ACC-----
AAAATACGTTAAAACTTTCAACAACGGATCTCTTGGTTCTGGCATCGATGAAGA
ACGCAGCGAAATGCGATAAGTAATGTGAATTGCAGAATTCAGTGAATCATCGA
ATCTTTGAACGCACATT-
GCGCCCATTAGTATTCTAGTGGGCATGCCTATTCGAGCGTCATT-TCGA-
CCCTTAAG-CCCCTGTT-GCTTAGCG-TTGGGAGT-CTACGGC------------------------
TACGG--------CGTAGTTCCCGAAAATGA-GTGGCGGAGTTAG-GG---------
CGTACTCTAAGCGTAGTAAAC-TAT-----ACCTCTCGCTTCT-GCAG---TA-------------
--TCCC-TAG----------------------------CTACCTGCCGTAAAA----------------CCCCTA------
--------TCTCTTT-----------AGTGG-TGACCTCGGATAGAAGGAAAAACCCCAT--------
----

--

>H_carneum_MUCL_54177

GAGGAAGTAAAAGTCGTAACAAGGTCTCCGTTGGTGAACCAGCGGAGGGATCA
TTACCGAGTTCTGC-------------GAGA---------CTCCC------AACCC-TGT-
GTGAACCGTTAC-CGC--CGTTGCCTCGGCG-----------G-------------------------------------
--C-------------------GCC-------------------------------------
----

CCGCGCGCCGCCG-GAGGACC--CAAG---------CAACACT----CTCTCTGC-----C--
TAGCG-GC--GTGCATCTCTGAACGCGTA------ACC------
GAATCAGTTAAAACTTTCAACAACGGATCTCTTGGTTCTGGCATCGATGAAGAA
CGCAGCGAAATGCGATACGTAATGCGAATTGCAGAATTTAGTGAGCCATCGAA
TCTTTGAACGCACATT-
GCGCCCGCCAGCATTCTGGCGGGCATGCCTATCCGAGCGTCATT-TCAA-
CCCTTAAG-CCCCTGCC-GCTTAGCG-TTGGGACC-CTGCGGC-----------------------
GCGCGC--------CGCAGCTCCCCAAAGGTA-GTGGCGGTGCCG--GG---------
TGCACTCGTAGCGCAGTAAGC-CA------CACTCTCGCTCAC-GAGG---CG------------
--CCCC-CGG---------------------------CCACCCGCCGTAAAA---------------CGCCCC------
-------GTGCCTCT-----------
CGCGGTTGACCTCGGATTAGGTAGGGATACCCGCTGAACTTAAGCATATCAAT
A--

>H_ochraceum_MUCL_54625

GGAAGTAAAGTCGTAACAAGGTCTCCGTTGGTGAACCAGCGGAGGATCATT
ACCGAGTTATTA-------------AAAA---------ACTCC------CACCC-TTT-GTGAAT--
TTAC-TCT--TGTTGCCTCGGCGTTGGGAA----G---
CACCTACCC----TGGAG---------------GA-----------------------GCT----------------ACCCTG-
GAAGA---

---GACCTACCCT-------
GGAGAAGATTACCCCTCAGCTGAGAGGACCTACC---------------------------
CTGGAGAAGTT--------TTTTTCCACAGCACCTACCCT-----------------------------------
--GGA-GATGCACT--------------------
TTACACCACGCCG-GTGGACT--TT------------ATAA--------CTCTGT-----T--TTTTC-
CA--CTGAATCTCTGAAAAAATA------ACT------
AAATTAGTTAAAACTTTCAACAACGGATCTCTTGGTTCTGGCATCGATGAAGAA
CGCAGCGAAATGCGATAAGTAATGTGAATTGCAGAATTCAGTGAATCATCGAA
TCTTTGAACGCATATT-
GCGCCCATTAGTATTCTAGTGGGCATGCCTATTCGAGCGTCATT-TCAA-
CCCTTAAG--CCCTGTT-GCTTAGTG-TTGGGTAT-CTACCCT---------------------
GTAAAAG--------AGTAGTTCCCTAAATGTA-GTGGCGGTGTTT--AG---------
GGCACTGGTAGCGTAGTAAATTT-------ATTTCTCGCCTCT-AAAG---TG-------------
--CCC-TAG---------------------------AAGCCTGCCGTAAAA---------------
TTATAAATATCTTATATTTATATATAT-----------
CAAGGTTGACCTCGGATTAGGTAGGAATACCCGCTGAACTTAAGCATAT---------

>H_munkii_MUCL_53315

TGTTACCGCGT---GAACCC-TTT-GTGAACC-TTAC-
CGT--CGTTGCCTCGGCGGCGAGCCTCGTG---
TCGAAATCC----GAAGGGT---GCT------------------ACCCTG-
GAGC---

---CACTTACCCT--------
GTAGGT------------------------GGCTACC---
CTGGAGCTACCCC--
-------GGA-GCTACGTCGTA---------------GTGGACATCGGCCCCGTCG-AAGGACC--
CG---------------TTAAGA----AATTCTT---------------GCA--TCACGTCTGAACAATATTA-
A----AAA-----
AAAAATCGTTAAAACTTTCAGCAACGGATCTCTTGGTTCTGGCATCGATGAAGA
ACGCAGCGAAATGCGATAAGTAATGTGAATTGCAGAATTCAGTGAATCATCGA
ATCTTTGAACGCATATT-
GCGCCCATTAGTATTCTAGTGGGCATGCCTATTCGAGCGTCATTTTCAACCCCT
TAAG-CCCCTGTT-GCTTAGCG-TTAGGAAT-CTGCGTC------------------------------GCGG-
--------CGCAGTTCCCCAAAGGTA-GTGGCAGTGTAG--GG----------
CACACTCGTAGCGTAGTAGAT---------TTATCTCGCTTCC-GACG---TGT-------------
GGTCCC-CGAA-------------------------------CGACTCGC--

>H_samuelsii_MUCL_51843

TCATTACAGAGTTACTA------------TAAAA---------CTCCC------AACCC-TTT-
GTGAACC-TTAC-CGT--CGTTTCCTCGGCGGCGAGCCGCGCG---------------------------
------------------TCGGAATTC----AGAAG---------------GA----------------------GCT-----------
-----ACCCTG-GAGC---

CACCTACCCT-------GTAGGT---------------------GGCTACC------------------------------------
----------------------CTGGAGCTACCGT---
-----------------------------ATGGAGTGGACG---------------------CGCCGGCCCCGTCG-
ATGGACC--AGAC----------CAAA--------CTCTTT----GTCTATATA-CT--
ACGTGTCTCTGAACAACCG-A----AACA---
AAAAATTCGTTAAAACTTTCAACAACGGATCTCTTGGTTCTGGCATCGATGAAG
AACGCAGCGAAATGCGATAAGTAATGTGAATTGCAGAATTCAGTGAATCATCG
AATCTTTGAACGCATATT-
GCGCCCATTAGTATTCTAGTGGGCATGCCTATTCGAGCGTCATT-TCAA-
CCCTTAAG-CCTCTGTT-GCTTAGCG-TTAGGAAT-CTGCGTC------------------------
GAGAGA--------CGCAGCTCCCTAAAGGTA-GTGGCGGTGTCGG-GT---------
ACACTTCGTAGCGCAGTAACTTTTT-----TTTTCTCGCTTCT-GCAG---TG--------------
-TGCTGGAA----------------------------AGACTTACCGTAAAAA-AAAATA-------G---------
---------TGACTATTT---

>H_lechatii_MUCL_54609

TAGAGGAAGTAAAAGTCGTAACAAGGTCTCCGTTGGTGAACCAGCGGAGGGAT
CATTACCGAGTTCTAT---------------AA---------CTTTGT---AAAACCC-TAT-
GTGAAAT-GTATTCAT--GTATGCCTCGGCA-GGAGCT----GCTA------------------------
-----------------CGCTTTCTCG-AGGGTGAGGGCT----------------------------------GCT------
----------ACCCTG-TAGCAACC---

CCCCTTTCCCGGAC---
-------------GTGGCGCGGCCCT---
---------------------GTA-------------------------ATTGGACCCGTTCCTGCCA-GCAGGCT--
TC------------TAAAA-------TTCTAT-------TTACCA-----
CTATATATTCAGATTTTATTA----TGT-----
ATAATAAGTTAAAACTTTCAACAACGGATCTCTTGGTTCTGGCATCGATGAAGA
ACGCAGCGAAATGCGATAAGTAATGTGAATTGCAGAATTCAGTGAATCATCGA
ATCTTTGAACGCACATT-
GCGCCCATTAGTATTCTAGTGGGCATGCCTATTCGAGCGTCATT-TCAA-
CCCTTAAG-CCTCAGTTAGCTTAGCG-TTGGGAGA-CTGAGCCC-------------------

CCCCAGGGG--------CCCAGCTCCTCAAAGTCA-GCGGCGGAATTAG-GG---------
CGTATCCTAAGCGTAGTAGTC-T-------ATATCTCGCTTCT-GCGG---TA---------------
CCCC-TGA----------------------------TCTCTAGCCGTTAAA----------------CCCCCC---------
----TATAATCT-----------
AGTGGTTGACCTCGGATTAGGTAGGAATACCCGCTGAACTTAAGCATATCATA-

>Graphostroma_platystomum_CBS_270_87

TCATTAGCGAGTTAAAC---------------AA---------CTCCA------AAACC-CAT-
GTGAACA-TACC-TAT--CGTTGCCTCGGCAGGCTGCGCTTCG---------------------------
-------------GGCGGCTGTTACCC----TGTAGAAG------------GA-----------------------GCT---
-------------ACCCTG---

TAGCTACCCT-------ATAGCT--
--------------GGTGAGCTACCCT--
--------------------CGTAAGCTGCTGC----------------------CTAAAGCCTGCCG-
GCGGACC--CC------------TAAA--------CTCTGA---ATT--------TA--
CTGTATCTCTGAGTGTAAA------ACA-----
AAAATAAGTTAAAACTTTCAACAACGGATCTCTTGGTTCTGGCATCGATGAAG

AACGCAGCGAAATGCGATAAGTAATGTGAATTGCAGAATTCAGTGAATCATCG
AATCTTTGAACGCACATT-
GCGCCTAATAGTATTCTGTTAGGCATGCCTGTTCGAGCGTCATT-TCGA-
CCATTAAG-CCCTGTTT-GCTTAGCG-TTGGGAAC-TTACGCC-------------------------
TGC--------CGTAATTCCTTAAATTCA-GTGGCGGAGCTAG-GT---------
CATGCTCTAAGCGTAGTAATT-A-------TTTCCTCGCTTCT-GTAG---CT--------------
GGCC-TAT--ATCCTG----------------------

>Natonodosa_speciosa_CLM_RV86

CAGTTATCGTTTATTTGATAGTACCTTACTACTTGGATAACCGTGGTAATTCTAG
AGCTAATACATGCTAAAAAACCCGACTCACGGAGGGTTGTATTTATTAGATTA
AAAACCAATGCCCTTCGGGGTTTACTGGTGATTCATAATAACTTCTCGAATCGC
ATGGCCTTGTGCCGGCGATGGTTCATTCAAATTTCTGCCCTATCAACTTTCGAT
GGCAGGGTCTTGGCCTGCCATGGTTACAACGGGTAACGGAGGGTTAGGGCTCG
ACCCCGGAGAAGGAGCCTGAGAAACGGCTACTACATCCAAGGAAGGCAGCAG
GCGCGCAAATTACCCAATCCCGACTCGGGGAGGTAGTGACAATAAATACTGAT
ACAGGGCTCTTTTGGGTCTTGTAATTGGAATGAGTACAATTTAAATCCCTTAAC
GAGGAACAATTGGAGGGCAAGTCTGGTGCCAGCAGCCGCGGTAATTCCAGCTC
CAATAGCGTATATTAAAGTTGTTGCAGTTAAAAAGCTCGTAGTTGAACCTTGGG
TCTGGCTGGCCGGTCCGCCTCACCGCGTGCACTGGTTCGGCCGGACCTTTCCCT
CTGGGGAGCCCCATGCCCTTCACTGGGTGTGGTGGGGAACCAGGACTTTTACTG
TGAAAAAATTAGAGTGTTCAAAGCAGGCCTATGCTCGAATACATCAGCATGGA
ATAATAGAATAGGACGTGTGGTTCTATTTTGTTGGTTTCTAGGACCGCCGTAAT
GATTAATAGGGACAGTCGGGGGCATCAGTATTCAATTGTCAGAGGTGAAATTC
TTGGATTTATTGAAGACTAACTACTGCGAAAGCATTTGCCAAGGATGTTTTCAT
TAATCAGGAACGAAAGTTAGGGGATCGAAGACGATCAGATACCGTCGTAGTCT
TAACCATAAACTATGCCGACTAGGGATCGGACGATGTTATTTTTTGACTCGTTC
GGCACCTTACGAGAAATCAAAGTCTTTGGGTTCTGGGGGGAGTATGGTCGCAA
GGCTGAAACTTAAAGAAATTGACGGAAGGGCACCACCAGGAGTGGAGCCTGC
GGCTTAATTTGACTCAACACGGGGAAACTCACCAGGTCCAGACACAATGAGGA
TTGACAGATTGAGAGCTCTTTCTTGATTTTGTGGGTGGTGGTGCATGGCCGTTC
TTAGTTGGTGGAGTGATTTGTCTGCTTAATTGCGATAACGAACGAGACCTTAAC
CTGCTAAATAGCCCGTATTGCTTTGGCAGTACGCTGGCTTCTTAGAGGGACTAT
CCGCTCAAGCGGATGGAAGTTTGAGGCAATAACAGGTTGAATTCACAGGCCTG
TAATAGTGGACCTCTTTAAATATTCTGCTAGTCTAGTGCTTAATCTCTTCGAGG
GAAGCCCCTCGCTATCGGGAAGAGAGGCAGCGAAAGCTGCTTTGCACAGGCGA
CACTACCTGGTACAGGGAACGCTAAATCCTACTTACAGTGGGATAAGCCGATC

CTGTGGCGAGTTCGGGTCGCGCCGAACCGTCGCAACGCGCGGAAAGGAGTGGA
CTGCTACTTAGTAGCGGTTTAAGGTACGTGCTAATCCCTCGGGAAACCGAGTCC
TTGCAACTAGAGCCGATAACTCGAAGTGCAGGGAGTACCGGTGCGGTTAAACG
CACCGGTATGGCGTCTAGAATCCCGATTCGTCGCGGTTCTAGAAAATGCTGTGA
TGCCCTTAGATGTTCTGGGCCGCACGCGCGTTACACTGACAGAGGCAGCGAGT
ACTTCCTTGGTAGAAATACCCGGGTAATCTTGTTAAACTCTGTCGTGCTGGGGA
TAGAGCATTGCAATTATTGCTCTTCAACGAGGAATTCCTAGTAAGCGCAAGTCA
TCAACTTGCGTTGATTACGTCCCTGCCCTTTGTACACACCGCCCGTCGCTACTAC
CGATTGAATGGCTCAGTGAGGCTTTCGGACTGGCCCAGAGGAGTCGGCAACGA
CACCTCAGGGCCGGAAAGTTATCCAAACTCGGTCATTTAGAGGAAGTAAAAGT
CGTAACAAGGTCTCCGTTGGTGAACCAGCGGAGGGATCATTACTGAGTTGCAA-
--------------AA---------CTCCC------AAACC-CAT-GTGAACT-TACTGTTT--
TATTGCCTCGGCGGGTTGGCC--TG---
CAGCGGCCTCTCCTAGAGTTT--------GGGCGA----------------------GCTT---------------
ACCCTG-AAAATG--

GGCGCGCTTACCCTA--
----------ATTTCTGGGGGGCCTACCCT--
-------------------------------GCA-GCGCCGCT----------------------TACGGCCCGCCG-
GCGGTCT--AC------------TATA--------CTCTGT--------TTCTT-TA--
GTGAATCTCTGAGTGATTA-------------
TACAAATAATCAAAACTTTCAACAACGGATCTCTTGGTTCTGGCATCGATGAAG
AACGCAGCGAAATGCGATAAGTAATGTGAATTGCAGAATTCAGTGAATCATCG
AATCTTTGAACGCACATT-
GCGCCCATTAGTATTCTAGTGGGCATGCCTGTTCGAGCGTCATT-TCGA-
CCATTACG--CCCTGTT-GCGTAGCA-TTGGGACT-CTACCTT-----------------------------
-------CGGGTAGTTCCTCAAAGTTA-GTGGCAGAGTCGG-AT---------
CAGTCTCTAAGCGTAGTAATT-A-------TTATCTCGCTTCT-GTAG---CT---------------
GGCC-CG-----------------------------TCCCTCGCCGTAAAA----------------CCCCCA---------
-----ATTTTTC-----------AATGG--

>H_pulicicidum_CBS_122622

CATTAATGAGTTACTA---------------AA---------CTCCA-----AAACCC-TTT-
GTGAACC-TTAC-CGT--CGTTTCCTCGGCG-CGTGCT----
GCTGCTACCTGGAGCTACC-------------------CTGGAAGGCACCTACCC----
TGTAGTGGTTG-TCTGCCAAGA-----------------------GCT----------------
ATCCTGTCAGGCACCTACCTCTGT-----------------AGTGGTTGTCTG---------------------
----------CCAGGAGTTATCTTGGCAGGCACCTAC---
------------------------CCTGTAGTAGTTGTCTGCTAAG--
--AGTTATCCTAGCAGGCACCTACCCTGTA----
GTAGTT---------------------GTCTACC---
CTGGAGCTACCCT--
-------GTA-GCCGCG------------------------TGAAGGCCCGCCG-AAGGACC--GT-----------
-TAAA--------CTCTTG-----C-TTCTAC-AA--CTGTATCTCTGAACACGTA------ACT----
-
GAAATAAGTTAAAACTTTCAACAACGGATCTCTTGGTTCTGGCATCGATGAAG
AACGCAGCGAAATGCGATAAGTAATGTGAATTGCAGAATTCAGTGAATCATCG
AATCTTTGAACGCACATT-
GCGCCCATTAGTATTCTAGTGGGCATGCCTATTCGAGCGTCATT-TCAA-
CCCTTAAG--CCCTGTT-GCTTAGTG-TTGGGAGT-CTACGGC-------------------------
TTCGG--------CGTAGTTCCTGAAAGTCA-GTGGCGGAGTTAG-GG---------
TACACTCTCAGCGTAGTAATC----------TCTCTCGCTCGT-GTGG---TG---------------
GCCC-TGG----------------------------CTGCTGGCCGTAAAA----------------CCCCCT--------
------ATTTCT-----------AGT---

>H_fragiforme_MUCL_51264

TCATTAGAGGAATACCC-------------AAAA---------CCTCC------CAACC-
CCTAGTGAA-C-TTAC-CAC--TGTTTCCTCGGCG-TGCGC-----------------------------------
---ACCTGG-
TTGCCCGC---

GGGGGATCCGAGCCTACCCT---------------------------------GTAGCTACC--------------------
---------------------------------------CTGTAGCTACCCTGT----------------------------------
AGACCCGGGTCCTCAGC--------------------------------------ACACG-GCGCCGCT----------
------------CAAGGTCCCGCCG-AAGTACC--C-------------TGAA--------CTCTGT-----T----
----TAACGTGGAATTCTGAATGCTTC-A----ACT------
AAATAAGTTAAAACTTTCAACAACGGATCTCTTGGTTCTGGCATCGATGAAGA
ACGCAGCGAAATGCGATAAGTAATGTGAATTGCAGAATTCAGTGAATCATCGA
ATCTTTGAACGCACATT-
GCGCCCATTAGTATTCTAGTGGGCATGCCTATTCGAGCGTCATT-TCAA-
CCCTTAAG-CCTCTGTT-GCTTAGCG-TTGGGAGT-CTACGGG-------------------------
TTACCC--------TGTAGTTCCTGAAAACCA-TTGGCGGAGTCAG-GG---------
AGCACTCTAAGCGTATTACACAGTCTTT--TTGTCTCGCTTTG-GATA---TT----------
------CCC-CGC-----------------------------CTCCACGCCGTAAAA----------------CCCCCC----
--------ATAT----C-----------AAATGTTGACCTCG---

>H_howeanum_MUCL_47599

ATTAGAGGAATACCC-------------AAAA---------CCTCC------CAACC-CCT-GTGAA-C-
TTAC-CAC--TGTTTCCTCGGCG-TGCGC--
--ACCTGG-ATGCCCGC--------

--GGGAGATGCGGACTTACCCT--------------------
-----------GTAGCTACC--
CTGTAGCTACCCTGT----------------------------------AAGGCCGGATCTTCAGC------------
--------------------------ACACG-GCGCCGCT-----------------------CAAGGTCCCGCCG-
AAGTACC--C-------------TGAA--------CTCTGT-----T--------
TTACGTGGAATTCTGAATGCTTC-A----ACT------
AAATAAGTTAAAACTTTCAACAACGGATCTCTTGGTTCTGGCATCGATGAAGA
ACGCAGCGAAATGCGATAAGTAATGTGAATTGCAGAATTCAGTGAATCATCGA
ATCTTTGAACGCACATT-
GCGCCCATTAGTATTCTAGTGGGCATGCCTATTCGAGCGTCATT-TCAA-
CCCTTAAG-CCCCTGTT-GCTTAGCG-TTGGGAGT-CCACAGG-------------------------
TTACCC--------TGTAGCTCCTGAAAACCA-TTGGCGGAGTCTG-GG---------
AGCACTCTAAGCGTATTATACA---TTT--TTGTCTCGCTTTG-GATC---TT--------------
-GCCC-CGC-----------------------------CTCCACGCCGTAAAA----------------CACCCC-------
-----ATAT----C-----------AAATGTTGACCTCGA-TTAGGTAGGAATA-----------------------

----

>H_ticinense_CBS_115271

TCCGTAGGTGAACCTGCGGAGGGATCATTAGAGGAATATCC-------------AAAA------
-----CCTCC------TAACC-TGT-GTGAACC-TTAT-CTC--TG-TGTTTCGGCG-
TGTGCCCG--GCCTGCGACAACTGCC------------------------------CCCCTACCCT---------
-------------GGA-----------------------GCT----------------ACCCTG-AAGAAACT----------------
----

--GCGGGAGAGAAATCCCCCCT-------GCAGCTACCG-
-------------GGGGGCTACCC------------------------------
GGGAGGAAGATACCCTGGAAGCTGCAAAGTAGCTGCCCT-----------------------------
--GGG-GCCGCGGTTC-----
AAACATATGGCGCCACACGGGCCCGCCG-AAAGAAC---C------------CAAA--------
TTCTAT-----T--------CTGTGTGGAATTCGAAAAGCTTC-A----ACTA--
AAAAAATAAGTTAAAACTTTCAACAACGGATCTCTTGGTTCTGGCATCGATGA
AGAACGCAGCGAAATGCGATAAGTAATGTGAATTGCAGAATTCAGTGAATCAT
CGAATCTTTGAACGCACATT-
GCGCCCATTAGTATTCTAGCGGGCATGCCTATTCGAGCGTCATT-TCAA-
CCCTTAAG-CCTCTGTT-GCTTAGTG-TTGGGAGC-CTGCGTG-----------------------

TTACAG--------CGCAGTTCCTGAAAATTA-TCGGCGGAGTTAG-GG---------
AGCATTCCAAGCAAAATACGT-C-------TGTTTTTACACCT-GCAT---
CAAGCTTTGGAACCAGTGCCC-CGC---------------------------TTCCTTGCCGTAAAC--
--------------CCCCCC------------TAATATTC--------------
ATGTTGACCTCGGATTAGGTAGGAATACCCGCTGAACTTAAGCATATCAATAA
GCGGAGGA---

>Rostrohypoxylon_terebratum_CBS_119137

GGGATCATTACTGAGTTATCA-------------AAAA---------CTCCC------AACCC-ACT-
GTGAACC-TACC-TCT--GTTTCCTCCGGCGCTCCGGC----GCCG------------------------
-----------------AGAGGACCCT-TCTAA--ACT-TTA------
----GCACCTAG-TGCGT---

CCAGCCCC-------------------------------GCGTCCTAGCG---------------------------GG---------
-----------------GTCTGAATGATTTC---
-------------------------ACA------------ATG----------------GCGAAGTCGCCCC-CTGGAAA-
-CT------------TCGT--------TTCTAG-------GTGGTG-----ATGGAACCGCAGGTTTTAA-
A----ACC-----
AAAATCAGTTAAAACTTTCAACAACGGATCTCTTGGTTCTGGCATCGATGAAGA

ACGCAGCGAAATGCGATAAGTAATGTGAATTGCAGAATTCAGTGAATCATCGA
ATCTTTGAACGCACATT-
GCGCCCGTTAGCATTCTAGCGGGCATGCCTATTCGAGCGTCATT-ACAA-
CCCTTAAG--CCCTGTT-GCTTAGCG-TTGGGAGT-CTGCGCC----------------------
TTAGGG--------CGCAGTTCCTTAAATGTA-GTGGCGGAGTTAC-AG---------
CACACCCTGAGCGTAGTAGTG-TA------TCAGCTCGCTCCC-GGGG---AA------------
--TCTG-TGG-------------------------CTGCTTGCCGTAAAA-------------------------------
----

>H_olivaceopigmentum_DSM_107924

GTAAAAGTCGTAACAAGGTCTCCGTTGGTGAACCAGCGGAGGGATCATTAACG
AGTTAAAC-------------AAAA---------CTCCC-----AAACCC-TTT-GTGAACC-TTAC-
CAC--AGTTTCCTCGGCG-CAAGCGC---
TGGCTACCC----CGTAGTCGTAG---------------------------------
GCCTCTACCCTAGATTCCTACCCTG-TAGGAATC-------------------------
GATGATGGGTTGAAATCTATCTACCCAGTAGGAATCTACCCTAGAGATCTACCC
TGTAGAAATCTAT---

GACGGATTGAGATCTACCCT-------GTAGGA------------------

ATCTACCCTAGATCTTTACGCGTAGACGCTGTCTACGCTGTAGAGGT------
CGATGATGGGTTGAGATCTACCCTGTAGAGATCTATCAAAATATCTATTTCTAT
CTACCATATATTTGCCATCTACCCTATAGCAATCTATTGAAATATTTATTTTGTA
TCTACTGCCATCTACGCTGTAAAACAACAGCTGTAGCTAGCCAGCGCCCGCCG-
GTGGACC--GC------------TAAA--------CTCTTG-----T-CATACT-GA--
TGTGGAATCTGAATTATCA------ACT-----
GAAATAAGTTAAAACTTTCAACAACGGATCTCTTGGTTCTGGCATCGATGAAG
AACGCAGCGAAATGCGATAAGTAATGTGAATTGCAGAATTCAGTGAATCATCG
AATCTTTGAACGCATATT-
GCGCCCACTAGCATTCTGGTGGGCATGCCTATTCGAGCGTCATT-TCGA-
CCCCTAAG-CCCCTGTT-GCTTAGCG-TTGAGGAT-CTACGGC---------------------
TTCTGG--------CGTAGTCCTCTAAAGTTA-GTGGCGGAGCCGC-GG---------
CGTACTCTGAGCGTAGTAATTAA-------TCTCCTCGCTTTT-GCTG---TA-------------
CCCG-TAG-------------------------CTGTCGGCCGTAAAA---------------CCCCTTA------
-----AACCTTTCA----------
AGTGGTTGACCTCGGATTAGGTAGGAATACCCGCTGAACTTAAGC----------------
----

>P_hunteri_MUCL_52673

-----------------------------AACCC-TTT-GTGAACC-TTAC-CGT--CGTTGCCTCGGCG-

TGAGCCG--CGCGTGC-CCTG-------------------------------CAGCTACCCT-
GCGGCGGG------------CGACCCGG-----------GGGAAGGCT---------------ACCCCG-
CAGTAGACCTACCCCGCGGCGCCCGCCCTTCAGGGGCAGCTGTGCGGCGCGCG
CTATACGGCCCGCCGGAGGACCGTTCAAACTCTTGCTCCAAAAAAAGCTCGAA
AGCTTC-
AAAAAATTTCCGCTCCAAAGCCTGTTCGAAAAAAAGCTCCAAAGCTCGAAAAA
GAATCCTGCTCCAAAGCTTGCTCGAAAAATCCTGCTCCAAAGCTCGAAAATCCT
GCTCCAAAGCTCGAAGATCCTGCTCCAAAGCTCGCTCGAAAAAAAGCTCCAAA
GCTCGAAAAAAAGTTCCAAAGCTTTTGCTCGA----AAAAAGGA-------------------
TTCTGCT--CCAAAGCTTGCTC------------
--GAAATAAATGCT-------
--------------CCAAAGCTCGAAA-TGAAGCT--CC-----------AAAA--------GCTTAT----
TT-----------TTTTTTTTCTGAATTACTTCA----ATT-----
AAAATCAGTTAAAACTTTCAACAACGGATCTCTTGGTTCTGGCATCGATGAAGA
ACGCAGCGAAATGCGATAAGTAATGTGAATTGCAGAATTCAGTGAATCATCGA
ATCTTTGAACGCACATT-
GCGCCCATTAGTATTCTAGTGGGCATGCCTATTCGAGCGTCATT-TCGA-
CCCTTAAG-CCCTCGTT-GCTTAGCG-TTGGGAGC-CTGCGTC-------------------
CCCTAGGGG--------CGCAGTTCCTCAAAGTTA-GTGGCGGAGCTAG-GG---------
CACACTCTAAGCGTAGTAAGC---------TATTCTCGCTTCT-GTGG---TG--------------
TACC-TGG--------------------------CTTCCTGCCGTAAAA---------------CCCC------------
----

>P_nicaraguense_CBS_117739

ATTACTGAGTTGTCA--------------AAA---------CTCCA------AACCC-TTT-GTGAACC-
TTAC-CGT--CGTTGCCTCGGCG-TGANNG----GCGTGC-CCTA-----------------------------
-------CAGCTACCCT-GCGGCGGG-------------CGACCCGG------------NGGAAGGCT----
------------ACCCCG-CAGTAGACCTACCCCGCGGCGCCCG-
CCTTCAGGGGCAGCTGTGCGGCGCGCGCTATATGGCCCGCCGGAGGACCGTTC
AAACTCTTGCTCCAAAAAAAGCTCGAAAGCTTCAAAAAAATTTCCGCTCCAAA
GCCTGTTCGAAAAAAAGCTCCAAAGCTCGAAAAAGAATCCTGCTCCAAAGCTC
GCTCGAAAAATCCTGCTCCAAAGCTCGAAAATCCTGCTCCAAAGCTCGAAGAT
CCT-----------------------
GCTCCAAAGCTCGAAAAAAAGTTCCAAAGCTTTTGCTCTA----AAAAAGGA-------
--------------TTCTGCT--
CCAAAGCTTGCTC--
-------GAAATAAATGCT-----------------------CCAAACTCGAAA-TGAAGCT--CC---------
---AAAA--------GCTTAT-----T------------TTTTTTTTCTGAATTACTTCA----ATN-----
AAAATCAGTTAANACTTTCAACAACGGATCTCTTGGTTCTGGCATCGATGAAGA
ACGCAGCGAAATGCGATAAGTAATGTGAATTGCAGAATTCAGTGAATCATCGA
ATCTTTGAACGCACATTGGCGCCCATTAGTATTCTAGTGGGCATGCCTATTCGA
GCGTCATT-TCGA-CCCTTAAG-CCCTCGTT-GCTTAGCG-TTGGGAGC-CTGCGTC-
--------------------CCCTAGGGG--------CGCAGTTCCTCAAAGTTA-
GTGGCGGAGCTAG-GG---------CACACTCTAAGCGTAGTAAGC---------
TATTCTCGCTTCT-GTGG---TG---------------TACC-TGG-----------------------------
CTTCCTGCCGTAAAA----------------CCCCTA--------------TACATCT-----------
AGTGGTTGACCTCGAATTAGGTAGGAATA--

>H_vogesiacum_CBS_115273

AAGTAAAAGTCGTAACAAGGTCTCCGTTGGTGAACCAGCGGAGGGATCATTAC
TGAGTTCTAA-------------CAAA---------CTCCC-----AAACCC-TTT-GTCAACT-GTAC-
C----TGTTGCTTCGGCG-CGAGCT----
GTAGGTGGCTAACCCTTGGTGGGCTACCCTGTAGGCGTCTGCTTGGCGGCTACC
C----TGTAA----------------A----------------------GCT----------------ACCCTG-
TAAAGCTACCCT--
AGAGCTACCTTAGAGCCACTCCCGCAGCTATTT---

-------------------------ACCCCATATCCATGTATCTACCTG-------CTCGAT-----------------
---GACTACC---CGGGAGCTACCCTAG-----
-----------------TGCTACCCTGCAGATGCCTACC--
CTGTAGCCCATCCCGC----------------TACCCTGGAGCCCGCCG-AAAGACC--
GAAC----------CAAA--------CTCGTT-----TAGAATAT-TA--
TTGAATCTCTGAATCCTTC-A----ACT-----
AAAATTAGTTAAAACTTTCAACAACGGATCTCTTGGTTCTGGCATCGATGAAGA
ACGCAGCGAAATGCGATAAGTAATGTGAATTGCAGAATTCAGTGAATCATCGA
ATCTTTGAACGCACATT-
GCGCCCATTAGTATTCTAGTGGGCATGCCTATTCGAGCGTCATT-TCGA-
CCCTGAAG-CCCTGGCT-GCTTCGCG-TTGGGACTTCTACATC---------------------
TCTATATAG--------AGTAGTTCCTTAAAGTAA-TTGGCAGAGTTGG-GG---------
TATGCCCTAAGCGTAGTATTA-T-------ATTTCTCGCTTGAGGGTG---TG--------------
TCCC-TGG----------------------------CTACCAGCCGTAAAG----------------CTGTTT---------
---------TTAT-----------AGTGGTTGACCTCGGATTAGGTAGGAATACCCGCTGAACT-
----

>H_chrysalidosporum_FCATAS2710

TGGAAGTAAAAAAACGTAACAAGGTCTCCGTTGGTGAACCAGCGGAGGGATCA
TTACTGAGTTCTTA------------CCAAA---------CTCCC-----AAACCC-TAT-GTGAACA-
TTAC-CGTATCGTTGCCTCGGCG-----------G--
---GCAT--------------GCCCCT------------------------

--TCAAAAACGGCTCCGCCG-
GTGGATC--TACC----------TACAA-------CTCTTA-------GTCATA-CT--
GTGAATATCTGAATGCTTC-A----ACT-----
ATAAATAGTTAAAACTTTCAACAACGGATCTCTTGGTTCTGGCATCGATGAAGA
ACGCAGCGAAATGCGATAAGTAATGTGAATTGCAGAATTCAGTGAATCATCGA
ATCTTTGAACGCATATT-
GCGCCCATTAGTATTCTAGTGGGCATGCCTATTCGAGCGTCATT-TCAA-
CCCTTACG-CCCTCGTC-GCGTAGCG-TTGGGACT-CTACCGC--------------------
AGCTCCTGC--------GGTAGTTCCTGAAATGTA-GCGGGCGGACCTG-GAG-------
CCGTACCCCAAGCGTAGTAACT-T-------TTCTCTCGCTTCTGGCGG---TA------------
---CCTC-CGGG------------------------ACTTTCTAGCCCTAAAA----------------
CCCCCTA-----------ATATTAATC-----------AAGTGGTGACCTCGAA-
TAGGTAGAATCCCCAGTT--T-------------------

>Thamnomyces_dendroidea_CBS_123578

TCATTAGCGAGTGGTAA-----------TAATAA---------CTCGT------AGCCT-CGT-
GCGAACC------TACCGCGTAGCCTCGGCG-----------G---------------------------------------
---GTCGTG---------------

CCGCC---CGGTGTTTAGCCGG--------------
--GTGGCGCG-------------
---------CTATGGCCCGTCG-GTGGACG--TT-----------TTAAC-------CTATGC------------
--CC--TACGTATTCTGAATGGACT-A------------
GTAAACTATTACAACTTTCAACGACGGATCTCTTGGTTCTGGCATCGATGAAGA
ACGCAGCGAAATGCGATACGTAATGCGAATTGCAGAATTCAGTGAGTCATCGA
ATCTTTGAACGCACATT-
GCGCCCGCTAGCATTCTAGCGGGCATGCCTGCTCGAGCGTCATT-ACA--
CCCCTAAG-CCT------AGCTTGCG-TTGGGAAT-CTAGTCA---------------------
GCCTCGCCG--------GCTAGTTCCTCAAAATCA-GTGGCGGAGTCAG-GA---------
TAGACCGTACGCGTAGTAATC-----------ATCTCGCCTGC-GTAG---------------------
TCC-TGG----------------------------CGGCTTGCCGTAAAA----------------CATCCT----------
---T-------------------
CCACAGT--

>D_rogersii_YMJ_92031201

TCCGTAGGTGAACCTGCGGAGGGATCATTAGCGAGTTGTAA-------------AAAC---
GCTCC-GCTTC------TACCC-GAT-GTGAACGTTAACTTGT--
CGTTGCCTCGGCGTAGTGCT----G-------------------------------T--------------
AGGCTGCCT----GGTAGAATCTT--------TAA-----------------------GCTCT--------------
CTCTC--

--TCCCTGCTTT----
---TTA----------------------GGGTTGGCCTCCTAT-------------------
AAGATTAGGGGGGG--------ATTAGAGTAAAGAGAGCTACCCT-------------------------
--GTA--GTCTGCG----------------------
CGACGGCCCGCCG-TAGGACCAAAC------------TAAA--------CTCTGT-----T-
ATTATC-GA--GAGCGTCTCTGAATTTTTTAGAATGAAA-----
AAAACACGTTAAAACTTTCAACAACGGATCTCTTGGCTCTGGCATCGATGAAG
AACGCAGCGAAATGCGATAAGTAGTGTGAATTGCAGAATTCAGTGAATCATCG
AATCTTTGAACGCACATT-
GCGCCCACTAGCATTCTGGTGGGCATGCCTATTCGAGCGTCATT-ACGA-
CCTTTAAG-CCTCTGCA-GCTTAGCG-TTGGGTGC-GTGCGAGCTCC--------------
TACAGTAAGGGC--------CGCAGCTCCTTAAATACA-GTGGCGGCGTCGG-AG----

GGGTCTCCGTCCCGAGCGTAGTAGTT-TTC----GTCTCCTCGCTCTG-GTGG---CG--
-------------GTCC-
CTTTTCCTCAGGTTCCGAAAGGAAACTAAGAAAGCTGGCCGTAAAATAAAG-----
------CAACTT------------TACTTTTCT-----------
AGTGGTTGACCTCGGATTAGGTAGGGTTACCCGCTGAACTTAAGCATATCAATA
AGCGGAGGA--

>D_rogersii_GMBC0204

--CTTGT--CGTTGCCTCGGCGTAGTGCT----
G------------------------------T--------------AGGCTGCCT----GGTAGAATCTT--------TAC---
--------------------GCTCT--------------CTCCTC---

--------------------T-CCTGCTTT-------TTA----------------------GGGTTGGCCTCCTAT-------
------------AAGATTAGGGGGGG--------ATTAGAGTAGGGAGAGCTACCCT-------------
--GTA--GTCTGCG----------
------------CGACGGCCCGCCG-TAGGACC-AAC------------TAAA--------CTCTGT-----T-
ATTATC-GA--GAGCGTCTCTGAATTTTTAGA--ATGAA-----
AAAACACGTTAAAACTTTCAACAACGGATCTCTTGGCTCTGGCATCGATGAAG
AACGCAGCGAAATGCGATAAGTAGTGTGAATTGCAGAATTCAGTGAATCATCG

AATCTTTGAACGCACATT-
GCGCCCACTAGCATTCTGGTGGGCATGCCTATTCGAGCGTCATT-ACGA-
CCTTTAAG-CCTCTGCA-GCTTAGCG-TTGGGTGC-GTGCGAGCTCT-------------
TATAGTAAGGGC--------CGCAGCTCCTTAAATACA-GTGGCGGCGTCGG-AG----
GGGTCTCCGTCCCGAGCGTAGTAGTT-TTC----GTCTCCTCGCTCTG-GTGG---CG--
------------GTCC-CTTTTCCTCAGGTTCCGAAAGGAAACTAAGAAAG-----------------
----

>D_guizhouensis_GMBC0065

---AACGTTAACTTCT--
TGTCGCCTCGGCGTAGTGCT----G----------------------------C-------------
AGGCTGCCT----GGTAGAATTTT--------AGA---------------------GCTCT-------------
CTCTTC--

--TCGCTTCT-------
TAA--------------------GGCCCTAGCGCCTCTA-----------------TAGGTAGAGAGGGA--
------TAGAGCGGAAGAAAGACTGCCCT--
---------------------------------GCG--GCCCGCG--------------------CGACGGCCCGTCG-
TAGGACT-AAC-----------TAAA--------CTCTGG-----T-ATTATC-

GAGAGAGCGTCTCTGAATTTTTATA--AAGGA-----
AAACATGTTAAAAACTTTCAACAACGGATCTCTTGGCTCTGGCATCGATGAAG
AACGCAGCGAAATGCGATAAGTAGTGTGAATTGCAGAATTCAGTGAATCATCG
AATCTTTGAACGCACATT-
GCGCCCACTAGCATTCTGGTGGGCATGCCTATTCGAGCGTCATT-ACGA-
CCCTTAAG-CCTCTACG-GCTTAGCG-TTGGGTGC-GTGCGAGCCCCCT-----------
GTGGCGGGGGGAC--------CGCAGCTTCTTAAATATA-GTGGCGGCGTCGG-
AGGGGGGGGTCTCTGTCCCGAGCGTAGTAGTA--
---GTTC---

>D_comedens_YMJ_90071615

TCCGTAGGTGAACCTGCGGAGGGATCATTAGCGAGTCGTTG------------
AAAAAAACGCTCCGTCTTC------CACCC-ACC-GCGAACGTTAAACTCC--
CGTTGCCTCGGCGCAGTGCC----G----------------------------C-------------
GGGCTGCCT----GGCGGCGCCGG--------AGG----------------------ACCCC-------------
GCCCCC-TCGAAGCCTC---

---GGGGG--------
GGCGGGGGCCCGGAGCCGCCCT--
---------------------------------GCG--GTCCGCG-----------------------CGACGGCCCGCCG-
CAGGACC-TAT------------CAAA--------CTCTGT-----C-ATGACC----
GAGAGGCTTCTGAGCTGTAT-A----
GCAAGCAAGTTAAATGTTAAAACTTTCAACAACGGATCTCTTGGCTCTGGCATC
GATGAAGAACGCAGCGAAATGCGATAAGTAGTGTGAATTGCAGAATTCAGTGA
ATCATCGAATCTTTGAACGCACATT-
GCGCCCGCCAGCATTCTGGCGGGCATGCCTGTTCGAGCGTCATT-TCGA-
CCCTTAAG-CCCCCGCG-GCTTAGCG-TTGGGAGC-ATACGAGCCCC-------------
GCGACGGGGAGG--------CGTAGCTCCTCAAATACA-GTGGCGGCGTCGG-AG----
GGGGTTCCGTCCCGAGCGTAGTAGTT-C-------TCTCCTCGCTCCG-GCGG---CG-----
----------GACC-TTCTCCGCGGAG-------------------AAGCCGGCCGTAAAA---------------
ACGCGAGGA--------AACTCGACCT-----------
AATGGTTGACCTCGGATCAGGTAGGGCTACCCGCTGAACTTAAGCATATCAAT
AAGCGGAGGA--

--

>D_crateriformis_GMBC0205

---------------------------TTC------TACCC-ATT-GCGAACGTTAAACTCT--

TATTGCCTCGGCGTAGTGCT----G------------------------------------C-----------------
GGGCTGCCT----GGTAGAAAAGA--------AAA---------------------------CTTTT----------------
TTTTTC--

---TTTTTTTT--------
ATAGCA---------------------AAGTTCTCTCGCCTTTTA------
AGAGGGAGAATAAAGCTAAGAGAAA--------AGAAGCTAAAAGATAGCTACCCT-
--GCA--
GTCCGTG--------------------------CGACGGCCCGCCG-TAGGACC-AAT------------CAAA----
----CTCTGT-----T-ATTATC-GA--GAGAATTTCTGAACTGTTA-----TACG-----
AAAATATATTAAAACTTTCAACAACGGATCTCTTGGCTCTGGCATCGATGAAAA
ACGCAGCGAAATGCGATAAGTAGTGTGAATTGCAGAATTCAGTGAATCATCGA
ATCTTTGAACGCACATT-
GCGCCCACTAGTATTCTGGGGGGCATGCCTATTCGAGCGTCATT-ACGA-
CCCTTAAGCCCTCTATA-GCTTATAG-CTGGGAGA-GTGCGGGCCCCC-------------
GCAGAGGGGGAC--------CGCACCTTCTTAAAAACA-CTGGGGGCGTCTC-AG-------
----

>H_lienhwacheense_MFLUCC_14_1231

AAGTAAAAGTCGTAACAAGGTCTCCGTTGGTGAACCAGCGGAGGGATCATTAT
CGAGTTGTCT------------AAAAA---------CTCCCAGTAAAAACCCTTTT-GTGAACC-
TTAC-CGT--TGTTGCCTCGGCG-CGTAAG----
GGGAGAGGCCTCTTCTTGCTTATATC--------------------CAAGCACTCC-
CGCGTTGATGTTAATTTTTATTAATCCGGCGTTAGGTGGTATAAGTAGCTGCG---
----------GCCCCGGTGGTCCCCCTCCTCT--
------------------TGGTGGGGTACCTCC--

--------CGGGGGGGCATGCTAACCCC----------------------------------ACCTACC------------
----------------------------------TGGCAGCGCGGGTGCGGCCGCCCC--------------------------
---GCG-------------------------
TCAATGGAGCAGCCCACCATTTGAACT--
CTTGCATATCTTTTTAGACAGGGAGCCTCTGTTAGGGCGCTCCCGGTC--
TTAGTCTTAGGAAATATAT------ATA-----
TAATCATATAAAAACTTTCAACAACGGATCTCTTGGTTCTGGCATCGATGAAGA
ACGCAGCGAAATGCGATAAGTAATGTGAATTGCAGAATTCAGTGAATCATCGA
ATCTTTGAACGCATATT-
GCGCCCATTAGTATTCTAGTGGGCATGCCTATCCGAGCGTCATT-
TCGACCCCTTAAG-CCCCTGTT-GCTTAGCG-TTGGGAGC-CTGCGGT-----------------
----CCTCCGGGG--------CGCATCTCCTCAAAATGA-GTGGCGGAGTCGC-GG---------
CGTGCTCTGAGCGTAGTAGAT-G-------TCTTCTCGCTTTG-GTAG---CG---------------
CCCG-CGG----------------------------
CAGCTAGCCGTTAACACACGATAGGGGCTTACCCCCC-------------TATCTCTT------
-----AGTGGTTGACCTCGGATTAGGTAGGAATACCCGCTGAACTTAA-----------------
----

Table S4. Alignment of the LSU sequences used in the phylogenetic study.

>A_annulatum_CBS_140775

ACCCTTTGTGAACCTTACCGTCGTTTCCTCGGCGCACTGCTGTGGGAGGCT-------
-----------ACCCTGTAGCGGT-----TGTTTACCCTACA-GGACGCACCCT---------------
GCAGCG-GCGCCGAAAGGACTACCA----------AAACTCTTTTATCCAAGTTACCTC-
GAACAATTTACTATAC---
AATAGTTTAAAACTTTCAACAACGGATCTCTTGGTTCTGGCATCGATGAAGAAC
GCAGCGAAATGCGATAAGTAATGTGAATTGCAGAATTCAGTGAATCATCGAAT
CTTTGAACGCACATTGCGCCCATTAGTATTCTAGTGGGCATGCCTATTCGAGCG
TCATTTCAACCCCTTAAGCCCTGTTGCTTAGCGTTGGGAATCT----
GCTAGCCTCGGCGCAGTTCCTTAAATTCATTGGCGGAGCTGTGGCACACTCTAG

GCGTAGTAG---TTTAACACCTCGCCTCTAGAG--
TGGCCGCGGTTACTGGCCGTAAAA--------------
CCCCTATATTTCTAGTGGTTGACCTCGGATTAGGTAGGAATACCCGCTGAACTT
AAGCATATCAATAAGCGGAGGAAAAGAAACCAACAGGGATTGCCCTAGTAAC
GGCGAG--TGAAGCGGCAACAGCTCAAA-TTTGAAATCTGGCCC---------
TCGCGGTCCGAGTTGTAATTTGCAGAGGATGCTTTTGGTGCGGT-
GCCTTCCGAGTTCCCTGGAACGGGACGCCAGAGAGGGTGAGAGCCCCGTACGG
TTGGC-
CGCCTACCCTATATATAGCTCCTTCGACGAGTCGAGTAGTTTGGGAATGCTGCT
CTAAATGGGAGGTAAATTTCTTCTAAAGCTAAATACCGGCCAGAGACC-
GATAGCGCACAAGTAGAGTGATCGAAAGATGAAAAGCACTTTGAAAAGAGGG
TTAAATAGCACGTGAAATTGTTGAAAGGGAAGCG-
TTTGCGACCAGACTTTTTCCAGGCGGATCATCCGGTG-TTC-
TCACCGGTGCACTTCGCCTGG--TTTAGGCCAGCATCGGTTTC---
TCTAGGGGGATAAAGGCCTGGGGAACGTAGCTCT----TTA--GGGAGTGTT-
ATAGCCCCTTGCGTAATACCCCT-CGGGGGACCGAGGACC--------------------------
----

>A_truncatum_CBS_140778

ACCCTTTGTGAACCTTACCGTCGTTTCCTCGGCGCACTGCTGCGACCGCCCCGC
AGGCGGCCCTCGCGGCCCCGCGGGCAGC-----
TACTTACCCTGTACGGATCTACCCTGCAGCAGCTGAAGGCGCAGCG-
GCGCCGAAAGGACCGCTA---------
CAAACTCTTTATCCAAGCTACGTCTGAACAATTTACTATGCA-

AAATAGTTTAAAACTTTCAACAACGGATCTCTTGGTTCTGGCATCGATGAAGAA
CGCAGCGAAATGCGATAAGTAATGTGAATTGCAGAATTCAGTGAATCATCGAA
TCTTTGAACGCACATTGCGCCCATTAGTATTCTAGTGGGCATGCCTATTCGAGC
GTCATTTCGA-CCCTTAAGCCCTGTTGCTTAGCGTTGGGAGTCT----
GCGGCTTCGGCCGCAGTTCCTTAAAGTCAGTGGCGGAGCTGTGGCACACTCTA
GGCGTAGTAGT--TTTACCGCCTCGCCTCCAGAG--
TGGCCCCGGCTGCCTGCCGTAAAA-------------
CCCCTAATTTTCTAGTGGTTGACCTCGGATTAGGTAGGAATACCCGCTGAACTT
AAGCATATCAATAAGCGGAGGAAAAGAAACCAACAGGGATTGCCCTAGTAAC
GGCGAG--TGAAGCGGCAACAGCTCAAA-TTTGAAATCTGGCCC---------
TCGCGGTCCGAGTTGTAATTTGCAGAGGATGCTTTTGGTGCGGT-
GCCTTCCGAGTTCCCTGGAACGGGACGCCAGAGAGGGTGAGAGCCCCGTACGG
TTGGA-
CACCTACCCTATATATAGCTCCTTCGACGAGTCGAGTAGTTTGGGAATGCTGCT
CTAAATGGGAGGTAAATTTCTTCTAAAGCTAAATACCGGCCAGAGACC-
GATAGCGCACAAGTAGAGTGATCGAAAGATGAAAAGCACTTTGAAAAGAGGG
TTAAATAGCACGTGAAATTGTTGAAAGGGAAGCG-
TTTGCGACCAGACTTTTTCCAGGCGGATCATCCGGTG-TTC-
TCACCGGTGCACTTCGCCTGG--TTTAGGCCAGCATCGGTTTC---
CTTAGGGGGATAAAGGCCTGGGGAACGTAGCTCT----CTA--GGGAGTGTT-
ATAGCCCCTCGCGTAATACCCCT-CGGGGGACCGAGGACCGCGC------------------
----

>H_pilgerianum_STMA_13455

AGGAAGTAAAAGTCGTAACAAGGTCTCCGTTGGTGAACCAGCGGAGGGATCAT
TACTGAGTTCTTACAAACTCCCAACCCTTTGTGAATCATATCACTGTTGCCTCG
GCGCCGAGCGGCAGCTACCCGGGAGCT-----------
ACCCTGTAGAAGCGAGAGCATCTACCCTGTA----GCTACCCT--------------
GTAGTT-
GCACTCAACGCTCCGCCGGCGGACCATTCAACTCTGTTTTACAGTGTATCTCTG
AGTACT----TAAACT-
AAATAAGTTAAAACTTTCAACAACGGATCTCTTGGTTCTGGCATCGATGAAGA
ACGCAGCGAAATGCGATACGTAATGTGAATTGCAGAATTCAGTGAATCATCGA
ATCTTTGAACGCATATTGCGCCCATTAGTATTCTAGTGGGCATGCCTATTCGAG
CGTCATTTCAA-CCCTTAAGCCCTGTAGCTTAGTGTTGGGACTCT----
ACTCTTTAGTGAGCAGTTCCCTAAAACCAGTGGCGGTGCTA-
GGTACACTCATAGCGTAGTAA-----TTCTTTCTCGCTTCTGCGG--
TGGACGTAGCTACCTGCCGTAAAA-------------
CCCCCTATTTTCTAATGGTTGACCTCGGATTAGGTAGGAATACCCGCTGAACTT
AAGCATATCAATAAGCGGAGGAAAAGAAACCAACAGGGATTGCCCTAGTAAC
GGCGAG--TGAAGCGGCAACAGCTCAAA-TTTGAAATCTGGCCC---------
TAGCGGTCCGAGTTGTAATTTGTAGAGGATGCTTTTGGTGCGGT-
GCCTTCCGAGTTCCCTGGAACGGGACGCCAGAGAGGGTGAGAGCCCCGTACGG
TTGGA-
CACCTACCCTATATATAGCTCCTTCGACGAGTCGAGTAGTTTGGGAATGCTGCT
CTAAATGGGAGGTAAATTTCTTCTAAAGCTAAATACCGGCCAGAGACC-
GATAGCGCACAAGTAGAGTGATCGAAAGATGAAAAGCACTTTGAAAAGAGGG
TTAAATAGCACGTGAAATTGTTGAAAGGGAAGCG-
TTTGCGACCAGACTTTTTCAGGGAGGATCATCCGGTG-TTC-
TCACCGGTGCACTTCACCCTG--TTTAGGCCAGCATCGGTTTT---
TGCAGGGGGATAAAAACTTGGGGAATGTGGCTCC----TTC--GGGAGTGTT-
ATAGCCCCTTGTATAATACCCCT-GCGGAG--

>A_michelianum_CBS_119993

---ATTGCCCTAGTAACGGCGAG--
TGAAGCGGCAACAGCTCAAA-TTTGAAATCTGGCCC---------
TCGCGGTCCGAGTTGTAATTTGTAGAGGATGCTTTTGGTACGGT-
GCCTTCCGAGTTCCCTGGAACGGGACGCCAGAGAGGGTGAGAGCCCCGTACGG
TGGGA-
CACCTATCCTATATATAGCTCCTTCGACGAGTCGAGTAGTTTGGGAATGCTGCT
CTAAATGGGAGGTAAATTTCTTCTAAAGCTAAATACCGGCCAGAGACC-
GATAGCGCACAAGTAGAGTGATCGAAAGATGAAAAGCACTTTGAAAAGAGGG
TTAAATAGCACGTGAAATTGTTGAAAGGGAAGCG-
TTTGCGACCAGACCTTTTCCAGGCGGATCATCCGGTG-TTC-
TCACCGGTGCACTTCGCCTGG--TTTAGGCCAGCATCGGTTTC---
CTTAGGGGGATAAAGGCCTGGGGAACGTAGCTCT----TTA--GGGAGTGTT-
ATAGCCCCTCGCGTAATACCCCT-CAGGGGACCGAGGACCGCGC-TCT----
GCAAGGATGCTGGCGTAATGGTCGTCAACGACCCGTCTTGAAACACGGACCAA
GGAGTCGAACATTTGTGCGAGTGTTTGGGTG--
TTAAACCCTCACGCGTAATGAAAGTGAACGGAGGTGAGAGCCCTT-------
AGGGGTGCATCATCGACCGATCCTGATG-
TCTTCGGATGGATTTGAGTAAGAGCATAACTGTTCGGACCCGAAAGATGGTGA
ACTATGCGTGGATAGGGTGAAGCCAGAGGAAACTCTGGTGGAGGCTCGCAGCG
GTTCTGACGTGCAAATCGATCGTCAAATCTGCGCATGGGGGCGAAA--------------

GACTTATCGAACCATTAA---AAACCAGCGGTAAA--TT-
TGAATTACTAGGTGGTTAAGAGGCCCCTC---

>H_invadens_MUCL_51475

--A-
TTTGAAATCTGGCCC---------
TCGTGGTCCGAGTTGTAATTTGTAGAGGATGCTTTTGGTGCGGT-
GCCTTCCGAGTTCCCTGGAACGGGACGCCATAGAGGGTGAGAGCCCCGTACGG
TTGGA-
CGCCTACCCTATATATAGCTCCTTCGACGAGTCGAGTAGTTTGGGAATGCTGCT
CTAAATGGGAGGTAAATTTCTTCTAAAGCTAAATACCGGCCAGAGACC-
GATAGCGCACAAGTAGAGTGATCGAAAGATGAAAAGCACTTTGAAAAGAGGG
TTAAATAGCACGTGAAATTGTTGAAAGGGAAGCG-
TTTGCGACCAGACTTTTTCCAGGCGGATCATCCGGTG-TTC-
TCACCGGTGCACTTCGCCTGG--TTTAGGCCAGCATCGGTTTC---
CTTAGGGGGATAAAGGCCTGGGGAACGTAGCTCT----CTA--GGGAGTGTT-
ATAGCCCCTCGCGTAATACCCCT-CGGGGGACCGAGGATCGCGC-TCT----
GCAAGGATGCTGGCGTAATGGTCGTCAACGACCCGTCTTGAAACACGGACCAA
GGAGTCGAACATTTGTGCGAGTGTTTGGGTG--
TTAAACCCTCACGCGTAATGAAAGTGAACGGAGGTGAGAGCCCTT-------
ACGGGTGCATCATCGACCGATCCTGATG-
TCTTCGGATGGATTTGAGTAAGAGCATAACTGTTCGGACCCGAAAGATGGTGA
ACTATGCGTGGATAGGGTGAAGCCAGAGGAAACTCTGGTGGAGGCTCGCAGCG
GTTCTGACGTGCAAATCGATCGTCAAATCTGCGCATGGGGGCGAAA---------------
----
GACTTATCGAACCATT-----ACACCAGCGGTAAA--TT-
TGATTTACTAGGTGGTTAAGAGGGACCCCGTGAGGGGATGTTACCCTAGGTCA
CCGGGTCGTCCTACCCTGTA----GCTACCCTGTAG------------------------
CTGTCTTGGAACGACTGGCCCG---
---------------------------------------GCCGACACCGCTAAATTGCGGGGACGTCCTAAT-
ACAGGTCGCAGGCTACCGCCGGGCGCTGAAAAGCGTGTTTCCGGCACCAAGAA
TAGCGCTCTTGGGTATGGTAAAAACGCCTGCGATAGCGGATGACCTGCAGCCA
ACCCCG-----
CCTTAGGGGAGAGTTCACTGACTAAACAGCGGTGGGTCAGCGCGCGTC-----------
GCTGGCCTAAGACATAGTCGATCCAGGCCCTGAAAAGGTGCCTTACC----------
CTATAGGTG-----------------CTACCCTGTAAGTG---------CATACCCTATAG-----
GTATGCAGCGGAGCTAGTAGCTGGTTACCGCCGAAGTTTCCCTCAGGATAGCA

GTGTTG--
TATTCAGTTTTATGAGGTAAAGCGAATGATTAGGGACTCGGGGGCGCTATTTAG
CCTTCATCCATTCTCAAACTTTAAATATGTAAGAAGCCCTTGTTGCTTAGTTGA
ACGTGGGCATTCGAATGTACCAACACTAGTGGGCCATTTTTGGTAAGCAGAAC
TGGCGATGCGGGATGAACCGAACGCGGGGTTAAGGTGCCAGAGTGGACGCTCA
TCAGACACCACAAAAGGTGTTAGTACATCTTGACAGC-------------------------------
----

AGGACGGTGGCCATGGAAGTCGGAATCCGCTAAGGACTGTGTAACAACTCACC
TGCCGAATGTACTAGCCCTGAAAATGGATGGCGCTCAAGCGTCT-
CACCCATACCTCGCCC---

>H_macrocarpum_CBS_119012

---GCCCTAGTAACGGCGAG--
TGAAGCGGCAACAGCTCAAA-TTTGAAATCTGGCCC---------
TCGTGGTCCGAGTTGTAATTTGTAGAGGATGCTTTTGGTGCGGT-
GCCTTCCGAGTTCCCTGGAACGGGACGCCATAGAGGGTGAGAGCCCCGTACGG
TTGGA-
CGCCTACCCTATATATAGCTCCTTCGACGAGTCGAGTAGTTTGGGAATGCTGCT
CTAAATGGGAGGTAAATTTCTTCTAAAGCTAAATACCGGCCAGAGACC-
GATAGCGCACAAGTAGAGTGATCGAAAGATGAAAAGTACTTTGAAAAGAGGG
TTAAATAGCACGTGAAATTGTTGAAAGGGAAGCG-
TTTGCGACCAGACTTTTTCCAGGCGGATCATCCGGTG-TTC-
TCACCGGTGCACTTCGCCTGG--CTTAGGCCAGCATCGGTTTC---
CTTAGGGGGATAAAGGCCTGGGGAACGTAGCTCT----CTA--GGGAGTGTT-
ATAGCCCCTCGCGTAATACCCCT-CGGGGGACCGAGGATCGCGC-TCT----
GCAAGGATGCTGGCGTAATGGTCGTCAACGACCCGTCTTGAAACACGGACCAA
GGAGTCGAACATTTGTGCAAGTGTTTGGGTG--
TTAAACCCTCACGCGTAATGAAAGTGAACGGAGGTGAGAGCCCTT-------
ACGGGTGCATCATCGACCGATCCTGATG-
TCTTCGGATGGATTTGAGTAAGAGCATAACTGTTCGGACCCGAAAGATGGTGA
ACTATGCGTGGATAGGGTGAAGCCAGAGGAAACTCTGGTGGAGGCTCGCAGCG
GTTCTGACGTGCAAATCGATCGTCAAATCTGCGCATGGGGGCGAAA-------------

GACTTATCGAACCATT-----ACACCAGCGGTAAA--TC-
TGATTTACTAGGTGGTTAAGAGGGACCCCGCGAGGGGGTGTTACCCTAGGTCA
CCGGGCCGTCCTACCCTGGA----GCTACCCTGTAG-----------------------
TTACCCTGGAGCGATTGGTCCG--
--GCCGACACCGCTAAATTGCGGGGACGTCCTATT-
GAAGGTCGCAGGCTACCGCCGGGCGCTGAAAAGCGTGTTGCCGGCACCAAGAA
TAGCGCTCTTGGGTATGGTAAGAACGCCTGCGATAGCGGATGACCTGCAGCCA
ACCCCG-----
CCGTAGGGGAGAGTTCACTGACTAAACAGCGGTGGGTCAGCGCGCGTC----------
GCTGGCCTAAGACATAGTCGATCCAGGCCCTGAAAAGGTGCCTTACC----------
CTGTAGGCG----------------TCTACCCTGTAGACG---------GTTACCCTATAG-----
GTACGCAGCGGAGCTAGTAGCTGGTTACCGCCGAAGTTTCCCTCAGGATAGCA
GTGTTG--
TATTCAGTTTTATGAGGTAAAGCGAATGATTAGGGACTCGGGGGCGCTATTTAG
CCTTCATCCATTCTCAAACTTTAAATATGTAAGAAGCCCTTGTTGCTTAATTGA
ACGTGGGCATTCGAATGTACCAACACTAGTGGGCCATTTTTGGTAAGCAGAAC
TGGCGATGCGGGATGAACCGAACGCGGGGTTAAGGTGCCAGAGTGGACGCTCA
TCAGACACCACAAAAGGTGTTAGTACATCTTGACAGC-----------------------------------
----

AGGACGGTGGCCATGGAAGTCGGAATCCGCTAAGGACTGTGTAACAACTCACC
TGCCGAATGTACTAGCCCTGAAAATGGATGGCGCTCAAGCGTCT-
CACCCATACCTCGCCCTTAGGGTAGAAACGATGCCCTAAGG-
AGTAGGCGGCCGTGG--GGG-TCAGTGACGAAGCCTAGGCG-----------------------------
----

>D_childiae_CBS_122881

GCATATCAATAAGCGGAGGAAAAGAAACCAACAGGGATTGCCCTAGTAACGG
CGAG--TGAAGCGGCAACAGCTCAAA-TTTGAAATCTGGCCC---------
TAGCGGTCCGAGTTGTAATTTGTAGAGGATGCTTTTGGTTAGGT-
GCCTTCCGAGTTCCCTGGAACGGGACGCCAGAGAGGGTGAGAGCCCCGTACGG
TTGGA-
CACCGAGCCTCTATATAGCTCCTTCGACGAGTCGAGTAGTTTGGGAATGCTGCT
CTAAATGGGAGGTAAATTTCTTCTAAAGCTAAATACCGGCCAGAGACC-

GATAGCGCACAAGTAGAGTGATCGAAAGATGAAAAGTACTTTGAAAAGAGGG
TTAAATAGCACGTGAAATTGTTGAAAGGGAAGCG-
TTTGCGACCAGACTTTTTCCAGGCGGATCATCCGGTG-TTC-
TCACCGGTGCACTTCGCCTGG--TTTAGGCCAGCATCGGTTCC---
CTTAGGGGGATAAAGGCCTGGGGAACGTAGCTCC----TTC--GGGAGTGTT-
ATAGCCCCTAGCGTAATACCCTT-CGGGGGACCGAGGAACGCGCATCT----
GCAAGGATGCTGGCGTAATGGTCGTCAACGACCCGTCTTGAAACACGGACCAA
GGAGTCGAACATTTGTGCGAGTGTTTGGGTG--
TTAAACCCTCACGCGTAATGAAAGTGAACGGAGGTGAGAGCCCTT-------
CGGGGTGCATCATCGACCGATCCTGATG-
TCTTCGGATGGATTTGAGTAAGAGCATAACTGTTCGGACCCGAAAGATGGTGA
ACTATGCGTGGATAGGGTGAAGCCAGAGGAAACTCTGGTGGAGGCTCGCAGCG
GTTCTGACGTGCAAATCGATCGTCAAATCTGCGCATGGGGGCGAAA---------------
----
GACTTATCGAACCATC---

TAGTAGCTGGTTACCGC--

>H_fragiforme_MUCL_51264

--AATCT-
GGCC---------TCGTGGTCCGAGTTGTAATTTGTAGAGGATGCTTTTGGTGCGGT-
GCCTTCCGAGTTCCCTGGAACGGGACGCCAGAGAGGGTGAGAGCCCCGTACGG
TTGGA-
CACCTACCCTATATATAGCTCCTTCGACGAGTCGAGTAGTTTGGGAATGCTGCT

CTAAATGGGAGGTAAATTTCTTCTAAAGCTAAATACCGGCCAGAGACC-
GATAGCGCACAAGTAGAGTGATCGAAAGATGAAAAGCACTTTGAAAAGAGGG
TTAAATAGCACGTGAAATTGTTGAAAGGGAAGCG-
TTTGCGACCAGACTTTTTCCAGGGGGATCATCCGGTG-TTC-
TCACCGGTGCACTCCGCCTGG--TTTAGGCCAGCATCGGTTCT---
CTTAGGGGGATAAAGGCTTGGGGAACGTAGCTCC----TTC--GGGAGTGTT-
ATAGCCCCTTGCGTAATA-CCTT-CGGGGGACCGAGGATCGCGC-TCT----
GCAAGGATGCTGGCGTAATGGTCGTCAACGACCCGTCTTGAAACACGGACCAA
GGAGTCGAACATTTGTGCGAGTGTTTGGGTG--
TTAAACCCTCACGCGTAATGAAAGTGAACGGAGGTGAGAGCCCTT-------
ACGGGTGCATCATCGACCGATCCTGATG-
TCTTCGGATGGATTTGAGTAAGAGCATAACTGTTCGGACCCGAAAGATGGTGA
ACTATGCGTGGATAGGGTGAAGCCAGAGGAAACTCTGGTGGAGGCTCGCAGCG
GTTCTGACGTGCAAATCGATCGTCAAATCTGCGC---

>J_cohaerens_CBS_119126

TACGGGTTACCCTGTAGTTCCTGAAAACCATTGGCGGAGTCAGGGAGCACTCT
AAGCGTATTACACAATCTTTTTGTCTCGCTTTGGATATTGCCCCGCCTCCACGCC
GTAAAA----------------
CCCCCCATATCAAATGTTGACCTCGGATTAGGTAGGAATACCCGCTGAACTTAA
GCATATCAATAAGCGGAGGAAAAGAAACCAACAGGGATTGCCCTAGTAACGG
CGAG--TGAAGCGGCAACAGCTCAAA-TTTGAAATCTGGCCC---------

TCGTGGTCCGAGTTGTAATTTGTAGAGGATGCTTTTGGTGCGGT-
GCCTTCCGAGTTCCCTGGAACGGGACGCCAGAGAGGGTGAGAGCCCCGTACGG
TTGGA-
CACCTACCCTATATATAGCTCCTTCGACGAGTCGAGTAGTTTGGGAATGCTGCT
CTAAATGGGAGGTAAATTTCTTCTAAAGCTAAATACCGGCCAGAGACC-
GATAGCGCACAAGTAGAGTGATCGAAAGATGAAAAGCACTTTGAAAAGAGGG
TTAAATAGCACGTGAAATTGTTGAAAGGGAAGCG-
TTTGCGACCAGACTTTTTCCAGGGGGATCATCCGGTG-TTC-
TCACCGGTGCACTCCGCCTGG--TTTAGGCCAGCATCGGTTCT---
CTTAGGGGGATAAAGGCTTGGGGAACGTAGCTCC----TTC--GGGAGTGTT-
ATAGCCCCTTGCGTAATACCCTT-CGGGGGACCGAGGATCGCGC-TCT----
GCAAGGATGCTGGCGTAATGGTCGTCAACGACCCGTCTTGAAACACGGACCAA
GGAGTCGAACATTTGTGCGAGTGTTTGGGTG--
TTAAACCCTCACGCGTAATGAAAGTGAACGGAGGTGAGAGCCCTT-------
ACGGGTGCATCATCGACCGATCCTGATG-
TCTTCGGATGGATTTGAGTAAGAGCATAACTGTTCGGACCCGAAAGATGGTGA
ACTATGCGTGGATAGGGTGAAGCCAGAGGAAACTCTGGTGGAGGCTCGCAGCG
GTTCTGACGTGCAAATCGATCGTCAAATCTGCGCATGGGGGCGAAA---------------
----
GACTTATCGAACCATC--

TAGTAGCTGGTTACCGCCNAAGTTTCCCTCAGGATAGCAGTGTTG--
TTTTCAGTTTTATGAGGTAAAGCGAATGATTAGGGACTCGGGGGCGCTATTTAG
CCTTCATCCATTCTCAAACTTTAAATANNTAAGAAGCCCTTNTTACTTAATTGA
ACGTGGGCATTCGAATGTATCANCACTAGTGGGCCATTTTTGGTAAGCAGAACT
GGCGATGCGGGATGAACCGAACGCGGGGTTAAGGTGCCAGAGTGGACGCTCAT
CAGNCACCACAAAAGGTGTTAGTACATCTTGACAGC------------------------------------
----

AGGACGGTGGCCATGGAAGTCGGAATCCGCTAAGGAC---------------------------------
----

>H_trugodes_MUCL_54794

--CCTAGTAACGGCGAG--
TGAAGCGGCAACAGCTCAAA-TTTGAAATCTGGCCC---------
TTGCGGTCCGAATTGTAATTTGTAGAGGATGCTTTTGGTGCGGT-
GCCTTCTGAGTTCCCTGGAACGGGACGCCAGAGAGGGTGAGAGCCCCGTACGG
TTGGA-
CACCTAGCCTATATATAGCTCCTTCGACGAGTCGAGTAGTTTGGGAATGCTGCT
CTAAATGGGAGGTAAATTTCTTCTAAAGCTAAATACCGGCCAGAGACC-
GATAGCGCACAAGTAGAGTGATCGAAAGATGAAAAGCACTTTGAAAAGAGGG
TTAAATAGCACGTGAAATTGTTGAAAGGGAAGCG-
TTTGCGACCAGACTTTTTCCAGGCGGATCATCCGGTG-TTC-
TCACCGGTGCACTTCGTCTGG--TTTAGGCCAGCATCGGTTTT---
CTTAGGGGGATAAAGGCTTGGGGAACGTAGCTCT----TTC--GGGAGTGTT-
ATAGCCCCTTGCGTAATACCCCT-CGGGGGACCGAGGAACGCGC-TCT----
GCAAGGATGCTGGCGTAATGGTCGTCAACGACCCGTCTTGAAACACGGACCAA
GGAGTCGAACATTTGTGCGAGTGTTTGGGTG--
TTAAACCCTCACGCGTAATGAAAGTGAACGGAGGTGAGAGCCTT---------
AGGGTGCATCATCGACCGATCCTGATG-
TCTTCGGATGGATTTGAGTAAGAGCATAACTGTTCGGACCCGAAAGATGGTGA
ACTATGCGTGGATAGGGTGAAGCCAGAGGAAACTCTGGTGGAGGCTCGCAGCG
GTTCTGACGTGCAAATCGATCGTCAAATCTGCGCATGGGGGCGAAA--------------

GACTTATCGAACCATTAA---AAACCAGCGGTAAA--TT-
TGATTTACTAGGTGGTTAAGAGGGGCCCCGCGAGGGG-TACTACCCTAGAGT-
CTGGGAGGACCTACCCTATA----GCTACCCTGTAG-----------------------
CCTTAGGGTAACCCCCTCCCGA--

CCGACACCGCTAAATTGCGGGGACGTCCTATTAGAAGGTCGCAAGCTACCGCC
GGGCGCTGAAAAGCGTG--
CCCGGCACCAAGAATAGCGCTCTTGGGTATGGTAAAAACGCTTGCGATAGCGG
ATGACCTGCAGCCAACCCCG-----
TCGTAGGGGAGAGTTCACTGACTAAACAGCGGTGGGTCGGCGTTCGTT----
ACGTGACGTCGGCCTAAGACATAGTCGATCCAGGCCCTGAAAAGGTGCCTACC
CTATACCTACCCTGTAGGTGGCTCGACCCGAGCGCCGCTACCCTGTAGCGGCCC
GGACGAGCTACCTTATAGGAGGTGCTTTTAGCGGAGCTAGTAGCTGGTTACCG
CCGAAGTTTCCCTCAGGATAGCAGTGTTG--
TCTTCAGTTTTATGAGGTAAAGCGAATGATTAGGGACTCGGGGGCGCTATTTAG
CCTTCATCCATTCTCAAACTTTAAATATGTAAGAAGCCCTTGTTACTTAATTGA
ACGTGGGCATTCGAATGTACCAACACTAGTGGGCCATTTTTGGTAAGCAGAAC
TGGCGATGCGGGATGAACCGAACGCGGGGTTAAGGTGCCAGAGTGGACGCTCA
TCAGACACCACAAAAGGTGTTAGTACATCTTGACAGC---------------------------------
----

AGGACGGTGGCCATGGAAGTCGGAATCCGCTAAGGACTGTGTAACAACTCACC
TGCCGAATGTACTAGCCCTGAAAATGGATGGCGCTCAAGCGTCT-
CACCCATACCTCGCCCTTAGGGTAGAAACGATGCCCTAAGG-
AGTAGGCGGCCG---

>H_sp_MUCL_51392

--AACAGGGATTGCCCTAGTAACGGCGAG--
TGAAGCGGCAACAGCTCAAA-TTTGAAATCTGGCCC---------
TCGTGGTCCGAGTTGTAATTTGTAGAGGATGCTTTTGGCGCGGT-
GCCTTCTGAGTTCCCTGGAACGGGACGCCAGAGAGGGTGAGAGCCCCGTACGG
TTGGA-
CACCTAGCCTTTGTATAGCTCCTTCGACGAGTCGAGTAGTTTGGGAATGCTGCT
CTAAATGGGAGGTAAATTTCTTCTAAAGCTAAATACCGGCCAGAGACC-
GATAGCGCACAAGTAGAGTGATCGAAAGATGAAAAGCACTTTGAAAAGAGGG
TTAAATAGCACGTGAAATTGTTGAAAGGGAAGCG-
TTTGCGACCAGACCTTTTCCAGGCGGATCATCCGGTG-TTC-
TCACCGGTGCACTTCGCCTGG--TTTAGGCCAGCATCGGTTTT---
CTTAGGGGGATAAAGGCTTAGGGAACGTAGCTCC----TCA--GGGAGTGTT-
ATAGCCCTTTGCGTAATACCCCT-CGGGGGACCGAGGACCGCGC-TCT----
GCAAGGATGCTGGCGTAATGGTCGTCAACGACCCGTCTTGAAACACGGACCAA
GGAGTCGAACATTTGTGCGAGTGTTTGGGTG--
TTAAACCCTCACGCGTAATGAAAGTGAACGGAGGTGAGAGCCCTT-------
ACGGGTGCATCATCGACCGATCCTGATG-
TCTTCGGATGGATTTGAGTAAGAGCATAACTGTTCGGACCCGAAAGATGGTGA
ACTATGCGTGGATAGGGTGAAGCCAGAGGAAACTCTGGTGGAGGCTCGCAGCG
GTTCTGACGTGCAAATCGATCGTCAAATCTGCGCATGGGGGCGAAA--------------

GACTTATCGAACCATTAAAGGAAACCAGCGGTAAA-GAT-
TTATTTACTAGGTGGTTAAGAGGCCCCCCGCGAGGGGGCG----CCTAGGTT--
GGGGACCGCTCACCTTGTA----GCTACCCTGTAG-----------------------
CTACCCGGTAGAGCATACCCGGTAGG--TTCTACCCTGTAG---------------------
AACCTACCCGGGAGCTACCCTGCAAAG----------------------GAGTTTGCTCCC-
GAGCCGACACCGCTAAATTGCGGGGACATCCTATC--
AAGGCCGCAGGCTACCGCCGGGCGCTGAAAAGCGCT--
GCCGGCACCAAGAGTAGCGCTCTTGGGTGTGGTAAGAACGCCTGCGGTAACGG

ACGACCTGCAGCCAACCCCGCA-
AACTACAGGGGAGAGTTCACAGACTAAACAGCGGTGGGTTAGCGCTCGT---------
----GCTAGCCTAAGACATAGTCGATCCGGGCCCTGAGAAGGTGCCTAATCTACG-

AAATTTAGCGGAGCTAGTAGCTGGTTACCGCCGAAGTTTCCCTCAGGATAGCA
GTGTTG--
TCTTCAGTTTTATGAGGTAAAGCGAATGATTAGGGACTCGGGGGCGCTATTTAG
CCTTCATCCATTCTCAAACTTTAAATATGTAAGAAGCCCTTGTTACTTAATTGA
ACGTGGGCATTCGAATGTACCAACACTAGTGGGCCATTTTTGGTAAGCAGAAC
TGGCGATGCGGGATGAACCGAACGCGGGGTTAAGGTGCCAGAGTGGACGCTCA
TCAGACACCACAAAAGGTGTTAGTACATCTTGACAGC-------------------------------
----

AGGACGGTGGCCATGGAAGTCGGAATCCGCTAAGGACTGTGTAACAACTCACC
TGCCGAATGTACTAGCCCTGAAAATGGATGGCGCTCAAGCGTCT-
CACCCATACCTCGCCCTCAGGGTAGAAACGATGCCCTGAGG-
AGTAGGCGGCCGTGG--AGG-TTAGTGACGAAGCCTAGGGCGGAGCC--------------
----

>Rostrohypoxylon_terebratum_CBS_119137

---GCCCTAGTAACGGCGAG--
TGAAGCGGCAAAAGCTCAAA-TTTGAAATCTGGCCC---------
TAGCGGTCCGAGTTGTAATTTGCAGAGGATGCTTTCGGTGCGGT-
GCCTTCCGAGTTCCCTGGAACGGGACGCCATAGAGGGTGAGAGCCCCGTACGG
TTGGA-
CGCCTAGCCTCTATAAAGCTCCTTCGACGAGTCGAGTAGTTTGGGAATGCTGCT
CTAAATGGGAGGTAAATTTCTTCTAAAGCTAAATACCGGCCAGAGACC-
GATAGCGCACAAGTAGAGTGATCGAAAGATGAAAAGCACTTTGAAAAGAGGG
TTAAATAGCACGTGAAATTGTTGAAAGGGAAGCG-
TTTGCGACCAGACCTTCTCCGGGGGGATCACCCGCTG-TTC-
TCAGCGGTGCACTTCCCTCGG--TTTAGGCCAGCATCGGTTCT---
CTTAGGGGGATAAAGGCCTGGGGCACGTAGCTCT----TTA--GGGAGTGTT-
ATAGCCCCTGGCGTAATACCTTT-CAGGGGACCGAGGACCGCGC-TTC---
GGCAAGGATGCTGGCGTAATGGTCGTCAACGACCCGTCTTGAAACACGGACCA
AGGAGTCGAACATTTGTGCGAGTGTTTGGGTG--

TTAAACCCTCACGCGTAATGAAAGTGAACGGAGGTGAGAGCCTT---------
AGGGTGCATCATCGACCGATCCTGATG-
TCTTCGGATGGATTTGAGTAAGAGCATAACTGTTCGGACCCGAAAGATGGTGA
ACTATGCGTGGATAGGGTGAAGCCAGAGGAAACTCTGGTGGAGGCTCGCAGCG
GTTCTGACGTGCAAATCGATCGTCAAATCTGCGCATGGGGGCGAAA--------------
---GACTTATCGA--------

>H_papillatum_ATCC_58729

GAAGCGGCAACAGCTCAAA-TTTGAAATCTGGCCC---------
TCGTGGTCCGAGTTGTAATTTGTAGAGGATGCTTTTGGCGCGGT-
GCCTTCTGAGTTCCCTGGAACGGGACGCCAGAGAGGGTGAGAGCCCCGTACGG
TTGGA-
CACCTAGCCTCTGTATAGCTCCTTCGACGAGTCGAGTAGTTTGGGAATGCTGCT
CTAAATGGGAGGTAAATTTCTTCTAAAGCTAAATACCGGCCAGAGACC-
GATAGCGCACAAGTAGAGTGATCGAAAGATGAAAAGCACTTTGAAAAGAGGG
TTAAATAGCACGTGAAATTGTTGAAAGGGAAGCG-
TTTGCGACCAGACCTTTTCCTGGCGGATCATCCGGTG-TTC-
TCACCGGTGCACTTCGCTTGG--TTTAGGCCAGCATCGGTTTT---
CTTAGGGGGATAAAGGCTTAGGGAACGTAGCTCC----TCC--GGGAGTGTT-
ATAGCCCTCTGCGTAATACCCTT-CAGGGGACCGAGGACCGCGC-TCT----
GCAAGGATGCTGGCGTAATGGTCGTCAACGACCCGTCTTGAAACACGGACCAA

GGAGTCGAACATTTGTGCGAGTGTTTGGGTG--
TTAAACCCTCACGCGTAATGAAAGTGAACGGAGGTGAGAGCCCTT-------
ACGGGTGCATCATCGACCGATCCTGATG-
TCTTCGGATGGATTTGAGTAAGAGCATAACTGTTCGGACCCGAAAGATGGTGA
ACTATGCGTGGATAGGGTGAAGCCAGAGGAAACTCTGGTGGAGGCTCGCAGCG
GTTCTGACGTGCAAATCGATCGTCAAATCTGCGCATGGGGGCGAAA--------------

GACTTATCGAACCATTGA---AAACCAGCGGTAAAAAAT-
ACATTTACTAGGTGGTTAAGAGGTCCCCCGCGAGGGGGTT---ACCTAGGCT--
CGGGCAGGCTTACCCTGTA----GTTACCCTGTAA-----------------------
CTACCCTGTAGACCTTACCCGG--
GAGCTACCCTGTACGG-----------------------
GAGTTTGCTCCTAAAGCCGACACCGCTAAATTGCGGGGACATCCTATT--
AAGGCCGCAGGCTACCGCCGGGCGCTG-AAAGCGCT--
GTCGGCACCAAGAGTAGCGCTCTTGGGTATGGTAAAAACGCCTGCGGTAACGG
ACGACCTGCAGCCAACCCCGTATATTTACAGGGGAGAGTTCACAGACTAAACA
GCGGTGGGTTAGCGCATAT------------
GCTAGCCTAAGACATAGTCGATCCAGGCCCTGAGAAGGTGCCTAACTTATG-----

AAATATAGCGGAGCTAGTAGCTGGTTACCGCCGAAGTTTCCCTCAGGATAGCA
GTGTTG--
TCTTCAGTTTTATGAGGTAAAGCGAATGATTAGGGACTCGGGGGCGCTATTTAG
CCTTCATCCATTCTCAAACTTTAAATATGTAAGAAGCCCTTGTTACTTAATTGA
ACGTGGGCATTCGAATGTACCAACACTAGTGGGCCATTTTTGGTAAGCAGAAC
TGGCGATGCGGGATGAACCGAACGCGGGGTTAAGGTGCCAGAGTGGACGCTCA
TCAGACACCACAAAAGGTGTTAGTACATCTTGACAGC----------------------------------
----

AGGACGGTGGCCATGGAAGTCGGAATCCGCTAAGGACTGTGTAACAACTCACC
TGCCGAATGTACTAGCCCTGAAAATGGATGGCGCTCAAGCGTCT-
CACCCATACCTCGCCCTCAGGGTAGAAACGATGCC--------------------------------------
----

>D_bambusicola_CBS_122872

--ATTGCCCTAGTAACGGCGAG--
TGAAGCGGCAACAGCTCAAA-TTTGAAATCTGGCCC---------
TAGCGGTCCGAGTTGTAATTTGTAGAGGATGCTTTTGGTGAGGT-
GCCTTCCGAGTTCCCTGGAACGGGACGCCGGAGAGGGTGAGAGCCCCGTACGG
TTGGA-
CGCCAAACCTCTGTATAGCTCCTTCGACGAGTCGAGTAGTTTGGGAATGCTGCT
CTAAATGGGAGGTAAATTTCTTCTAAAGCTAAATACCGGCCAGAGACC-
GATAGCGCACAAGTAGAGTGATCGAAAGATGAAAAGCACTTTGAAAAGAGGG
TTAAATAGCACGTGAAATTGTTGAAAGGGAAGCG-
TTTGCGACCAGACCTTTTCCAGGCGGATCATCCGGCG-TTC-
TCGCCGGTGCACTCCGCCTGG--TTGAGGCCAGCATCGGTTTC---
CTTAGGGGGATAAAGGCCTGGGGAACGTAGCTCC----TTC--GGGAGTGTT-
ATAGCCCCTGGCGCAATACCCCT-CGGGGGACCGAGGACCGCGC-TTC---
GGCAAGGATGCTGGCGTAATGGTCGTCAACGACCCGTCTTGAAACACGGACCA
AGGAGTCGAACATTTGTGCGAGTGTTTGGGTG--
TCAAACCCTCACGCGTAATGAAAGTGAACGGAGGTGAGAGCCCTT-------
ACGGGTGCATCATCGACCGATCCTGATG-
TCTTCGGATGGATTTGAGTAAGAGCATAACTGTTCGGACCCGAAAGATGGTGA
ACTATGCGTGGATAGGGTGAAGCCAGAGGAAACTCTGGTGGAGGCTCGCAGCG
GTTCTGACGTGCAAATCGATCGTCAAATCTGCGCATGGGGGCGAAA--------------

GACTTATCGAACCATC---

TAGTAGCTGGTTACCGCCGAAGTTTCCCTCAGGATAGCAGTGTTG--
TCTTCAGTTTTATGAGGTAAAGCGAATGATTAGGGACTCGGGGGCGCTATATTG
CCTTCATCCATTCTCAAACTTTAAATATGTAAGAAGCCCTTGTTACTTAGTTGA
ACGTGGGCATTCGAATGTACCAACACTAGTGGGCCATTTTTGGTAAGCAGAAC
TGGCGATGCGGGATGAACCGAACGCGGGGTTAAGGTGCCAGAGTGGACGCTCA
TCAGACACCACAAAAGGTGTTAGTACATCTTGACAGC---------------------------------
----

AGGACGGTGGCCATGGAAGTCGGAATCCGCTAAGGACTGTGTAACAACTCACC
TGCCGAATGTACTAGCCCTGAAAATGGATGGCGCTCAAGCGTCTACACCCATA
CCTCGCCCTTAGGGTAGAAACGATGCCCTAAGG-AGTAGGCGGCCGTGGG-
AGG-TTAGTGACGAAGCCTA--

>D_eschscholtzii_MUCL_45435

---ATTGCCCTAGTAACGGCGAG--
TGAAGCGGCAACAGCTCAAA-TTTGAAATCTGGCCC---------
TAGCGGTCCGAGTTGTAATTTGTAGAGGAGGCTTTGGTGAGGT-
GCCTTCCGAGTTCCCTGGAACGGGACGCCGGAGAGGGTGAGAGCCCCGTACGG
TTGGA-
CGCCAAACCTCTGTATAGCTCCTTCGACGAGTCGAGTAGTTTGGGAATGCTGCT
CTAAATGGGAGGTAAATTTCTTCTAAAGCTAAATACCGGCCAGAGACC-
GATAGCGCACAAGTAGAGTGATCGAAAGATGAAAAGCACTTTGAAAAGAGGG
TTAAATAGCACGTGAAATTGTTGAAAGGGAAGCG-
TTTGCGACCAGACTTTTTCCAGGCGGATCATCCGGCG-TTC-
TCGCCGGTGCACTCCGCCTGG--TTGAGGCCAGCATCGGTTTC---
CTTAGGGGGATAAAGGCCTGGGGAACGTAGCTCC----TTC--GGGAGTGTT-
ATAGCCCCTGGCGTAATACCCCT-CGGGGGACCGAGGAACGCGC-TCT----
GCAAGGATGCTGGCGTAATGGTCGTCAACGACCCGTCTTGAAACACGGACCAA
GGAGTCGAACATTTGTGCGAGTGTTTGGGTG--
TCAAACCCTCACGCGTAATGAAAGTGAACGGAGGTGAGAGCCCTT-------
ACGGGTGCATCATCGACCGATCCTGATG-
TCTTCGGATGGATTTGAGTAAGAGCATAACTGTTCGGACCCGAAAGATGGTGA
ACTATGCGTGGATAGGGTGAAGCCAGAGGAAACTCTGGTGGAGGCTCGCAGCG
GTTCTGACGTGCAAATCGATCGTCAAATCTGCGCATGGGGGCGAAA---------------
----
GACTTATCGAACCATC---

TAGTAGCTGGTTACCGCCGAAGTTTCCCTCAGGATAGCAGTGTTG--
TCTTCAGTTTTATGAGGTAAAGCGAATGATTAGGGACTCGGGGGCGCTATATTG
CCTTCATCCATTCTCAAACTTTAAATATGTAAGAAGCCCTTGTTACTTAGTTGA
ACGTGGGCATTCGAATGTACCAACACTAGTGGGCCATTTTTGGTAAGCAGAAC
TGGCGATGCGGGATGAACCGAACGCGGGGTTAAGGTGCCAGAGTGGACGCTCA
TCAGACACCACAAAAGGTGTTAGTACATCTTGACAGC-----------------------------------
----

AGGACGGTGGCCATGGAAGTCGGAATCCGCTAAGGACTGTGTAACAACTCACC
TGCCGAATGTACTAGCCCTGAAAATGGATGGCGCTCAAGCGTCT-
CACCCATACCTCGCCCTTAGGGTAGAAACGATGCCCTAAGGNAGTAGGCGGCC
GTGGGAAGG-TTAGTGACGAAGCCTA--

>D_placentiformis_MUCL_47603

--ATTGCCCTAGTAACGGCGAG--
TGAAGCGGCAACAGCTCAAA-TTTGAAATCTGGCCC---------
TAGCGGTCCGAGTTGTAATTTGTAGAGGATGCTTTTGGTGAGGC-
GCCTTCCGAGTTCCCTGGAACGGGACGCCGGAGAGGGTGAGAGCCCCGTACGG
TTGGA-
CGCCAAACCTCTGTATAGCTCCTTCGACGAGTCGAGTAGTTTGGGAATGCTGCT
CTAAATGGGAGGTAAATTTCTTCTAAAGCTAAATACCGGCCAGAGACC-
GATAGCGCACAAGTAGAGTGATCGAAAGATGAAAAGCACTTTGAAAAGAGGG
TTAAATAGCACGTGAAATTGTTGAAAGGGAAGCG-
TTTGCGACCAGACTTTTTCCAGGCGGATCATCCGGCG-TTC-
TCGCCGGTGCACTCCGCCTGG--TTGAGGCCAGCATCGGTTTC---
CTTAGGGGGATAAAGGCCTGGGGAACGTAGCTCC----TTC--GGGAGTGTT-
ATAGCCCCTGGCGTAATACCCCT-CGGGGGACCGAGGAACGCGC-TCT----
GCAAGGATGCTGGCGTAATGGTCGTCAACGACCCGTCTTGAAACACGGACCAA
GGAGTCGAACATTTGTGCGAGTGTTTGGGTG--
TCAAACCCTCACGCGTAATGAAAGTGAACGGAGGTGAGAGCCCTT-------
ACGGGTGCATCATCGACCGATCCTGATG-
TCTTCGGATGGATTTGAGTAAGAGCATAACTGTTCGGACCCGAAAGATGGTGA
ACTATGCGTGGATAGGGTGAAGCCAGAGGAAACTCTGGTGGAGGCTCGCAGCG
GTTCTGACGTGCAAATCGATCGTCAAATCTGCGCATGGGGGCGAAA-------------

GACTTATCGAACCATC--

TAGTAGCTGGTTACCGCCGAAGTTTCCCTCAGGATAGCAGTGTTG--
TCTTCAGTTTTATGAGGTAAAGCGAATGATTAGGGACTCGGGGGCGCTATATTG
CCTTCATCCATTCTCAAACTTTAAATATGTAAGAAGCCCTTGTTACTTAGTTGA
ACGTGGGCATTCGAATGTACCAACACTAGTGGGCCATTTTTGGTAAGCAGAAC
TGGCGATGCGGGATGAACCGAACGCGGGGTTAAGGTGCCAGAGTGGACGCTCA
TCAGACACCACAAAAGGTGTTAGTACATCTTGACAGC------------------------------
----

AGGACGGTGGCCATGGAAGTCGGAATCCGCTAAGGACTGTGTAACAACTCACC
TGCCGAATGTACTAGCCCTGAAAATGGATGGCGCTCAAGCGTCT-
CACCCATACCTCGCCCTTAGGGTAGAAACGATGCCCTAAGG-
AGTAGGCGGCCGTGGG-AGG-TTAGTGACGAAGCCTA------------------------------------
----

>D_concentrica_CBS_113277

GCCCTAGTTAACGGCGAAGTTGAAGCGGCAACAGCTCAAATTTTGAAATCTGG
CCC---------TAGCGGTCCGAGTTGTAATTTGTAGAGGATGCTTTTGGTTAGGT-
GCCTTCTGAGTTCCCTGGAACGGGACGCCAGAGAGGGTGAGAGCCCCGTACGG
TTGGA-
CACCGAGCCTCTATATAGCTCCTTCGACGAGTCGAGTAGTTTGGGAATGCTGCT
CTAAATGGGAGGTAAATTTCTTCTAAAGCTAAATACCGGCCAGAGACC-
GATAGCGCACAAGTAGAGTGATCGAAAGATGAAAAGTACTTTGAAAAGAGGG
TTAAATAGCACGTGAAATTGTTGAAAGGGAAGCG-
TTTGCGACCAGACTTTTTCCAGGCGGATCATCCGGTG-TTC-
TCACCGGTGCACTTCGCCTGG--TTTAGGCCAGCATCGGTTCT---
CTTAGGGGGATAAAGGCCTGGGGAACGTAGCTCC----TTC--GGGAGTGTT-
ATAGCCCCTTGCGTAATACCCTT-CGGGGGACCGAGGAACGCGC-ATC---
TGCAAGGATGCTGGCGTAATGGTCGTCAACGACCCGTCTTGAAACACGGACCA
AGGAGTCGAACATTTGTGCGAGTGTTTGGGTG--
TTAAACCCTCACGCGTAATGAAAGTGAACGGAGGTGAGAGCCCTT-------
CGGGGTGCATCATCGACCGATCCTGATG-
TCTTCGGATGGATTTGAGTAAGAGCATAACTGTTCGGACCCGAAAGATGGTGA
ACTATGCGTGGATAGGGTGAAGCCAGAGGAAACTCTGGTGGAGGCTCGCAGCG
GTTCTGACGTGCAAATCGATCGTCAAATCTGCGCATGGGGGCGAAA---------------
----
GACTTATCGAACCATC---

TAGTAGCTGGTTACCGCCGAAGTTTCCCTCAGGATAGCAGTGTTG--
TCTTCAGTTTTATGAGGTAAAGCGAATGATTAGGGACTCGGGGGCGCTATATTG
CCTTCATCCATTCTCAAACTTTAAATATGTAAGAAGCCCTTGTTACTTAATTGA
ACGTGGGCATTCGAATGTACCAACACTAGTGGGCCATTTTTGGTAAGCAGAAC
TGGCGATGCGGGATGAACCGAACGCGGGGTTAAGGTGCCAGAGTGGACGCTCA
TCAGACACCACAAAAGGTGTTAGTACATCTTGACAGC--------------------------------------
----

AGGACGGTGGCCATGGAAGTCGGAATCCGCTAAGGACTGTGTAACAACTCACC
TGCCGAATGTACTAGCCCTGAAAATGGATGGCGCTCAAGCGTCT-
CACCCATACCTCGCCCTTAGGGTAGAAACGATGCCCTAAGG-
AGTAGGCGGCCGTGG--AGG-TTAGTGACGAAGCCTAGG-------------------------------------
----

>D_petriniae_MUCL_49214

---CAGCTCAAA-
TTTGAAATCTGGCCC---------
TAGCGGTCCGAGTTGTAATTTGTAGAGGATGCTTTTGGTTAGGT-
GCCTTCCGAGTTCCCTGGAACGGGACGCCAGAGAGGGTGAGAGCCCCGTACGG
TTGGA-
CGCCGAGCCTCTGTATAGCTCCTTCGACGAGTCGAGTAGTTTGGGAATGCTGCT
CTAAATGGGAGGTAAATTTCTTCTAAAGCTAAATACCGGCCAGAGACC-
GATAGCGCACAAGTAGAGTGATCGAAAGATGAAAAGTACTTTGAAAAGAGGG
TTAAATAGCACGTGAAATTGTTGAAAGGGAAGCG-
TTTGCGACCAGACTTTTTCTAGGCGGATCATCCGGTG-TTC-
TCACCGGTGCACTTCGCCTGG--TTTAGGCCAGCATCGGCTCT---
CTTAGGGGGATAAAGGCCAGGGGAACGTAGCTCC----TTC--GGGAGTGTT-
ATAGCCCCTAGCGTAATACCCTT-CGGGGGGCCGAGGAACGCGC-ATC---
TGCAAGGATGCTGGCGTAATGGTCGTCAACGACCCGTCTTGAAACACGGACCA
AGGAGTCGAACATTTGTGCGAGTGTTTGGGTG--
TCAAACCCTCACGCGTAATGAAAGTGAACGGAGGTGAGAGCCCTT-------
CGGGGTGCATCATCGACCGATCCTGATG-
TCTTCGGATGGATTTGAGTAAGAGCATAACTGTTCGGACCCGAAAGATGGTGA
ACTATGCGTGGATAGGGTGAAGCCAGAGGAAACTCTGGTGGAGGCTCGCAGCG
GTTCTGACGTGCAAATCGATCGTCAAATCTGCGCATGGGGGCGAAA--------------

GACTTATCGAACCATC--

TAGTAGCTGGTTACCGCCGAAGTTTCCCTCAGGATAGCAGTGTTG--
TCTTCAGTTTTATGAGGTAAAGCGAATGATTAGGGACTCGGGGGCGCTATATTG
CCTTCATCCATTCTCAAACTTTAAATATGTAAGAAGCCCTTGTTACTTAATTGA
ACGTGGGCATTCGAATGTACCAACACTAGTGGGCCATTTTTGGTAAGCAGAAC
TGGCGATGCGGGATGAACCGAACGCGGGGTTAAGGTGCCAGAGTGGACGCTCA
TCAGACACCACAAAAGGTGTTAGTACATCTTGACAGC---------------------------------
----

AGGACGGTGGCCATGGAAGTCGGAATCCGCTAAGGACTGTGTAACAACTCACC
TGCCGAATGTACTAGCCCTGAAAATGGATGGCGCTCAAGCGTCT-
CACCCATACCTCGCCCTTAGGGTAGAAACGATGCCCTAAGG-
AGTAGGCGGCCGTGG--AGG-TTAGTGACGAAGCCTAG---------------------------------
----

>D_vernicosa_CBS_119316

---------------GAGTTGTAATTTGTAGAGGATGCTTTTGGTTAGGT-
ACCTTCCGAGTTCCCTGGAACGGGACGCCAGAGAGGGTGAGAGCCCCGTACGG
TTGGA-
TACCGAGCCTCTATATAGCTCCTTCAACGAGTCGAGTAGTTTGGGAATGCTGCT
CTAAATGGGAGGTAAATTTCTTCTAAAGCTAAATACCGGCCAGAGACC-
GATAGCGCACAAGTAGAGTGATCGAAAGATGAAAAGTACTTTGAAAAGAGGG
TTAAATAGCACGTGAAATTGTTGAAAGGGAAGCG-
TTTGCGACCAGACTTTTTCCGGGCGGATCATCCGGTG-TTC-
TCACCGGTGCACTTCGCCCGG--TTTAGGCCAGCATCGGTTCT---
CCTAGGGGGATAAAGGCGGGGGGAACGTGGCTCC----TTC--GGGAGTGTT-
ATAGCCCCCCGCGTAATACCCCT-CGGGGGACCGAGGAACGCGC-ATC---
TGCAAGGATGCTGGCGTAATGGTCGCCAACGACCCGTCTTGAAACACGGACCA

AGGAGTCGAACATTTGTGCGAGTGTTTGGGTG--
TCAAACCCTCACGCGTAATGAAAGTGAACGGAGGTGAGAGCCCCT-------
CGGGGTGCATCATCGACCGATCCTGATG-
TCTTCGGATGGATTTGAGTAAGAGCATAAATGTTCGGACCCGAAAGATGGTGA
ACTATGCGTGGATAGGGTGAAGCCAGAGGAAACTCTGGTGGAGGCTCGCAGCG
GTTCTGACGTGCAAATCGATCGTCAAATCTGCGCATGGGGGCGAAA--------------

GACTTATCGAACCATC--

TAGTAGCTGGTTACCGCCGAAGTTTCCCTCAGGATAGCAGTGTTG--
TCTTCAGTTTTATGAGGTAAAGCGAATGATTAGGGACTCGGGGGCGCTATATTG
CCTTCATCCATTCTCAAACTTTAAATATGTAAGAAGCCCTTGTTACTTAATTGA
ACGTGGGCATTCGAATGTACCAACACTAGTGGGCCATTTTTGGTAAGCAGAAC
TGGCGATGCGGGATGAACCGAACGCGGGGTTAAGGTGCCAGAGTGGACGCTCA
TCAGACACCACAAAAGGTGTTAGTACATCTTGACAGC---------------------------------
----

AGGACGGTGGCCATGGAAGTCGGAATCCGCTAAGGACTGTGTAACAACTCACC
TGCCGAATGTACTAGCCCTGAAAATGGATGGCGCTCAAGCGTCT-
CACCCATACCTCGCCCTTAGGGTAGAAACGATGCCCTAAGG-
AGTAGGCGGCCGTGG--AGG-TTAGTGACGAAGCCTA-----------------------------------
----

>D_dennisii_CBS_114741

--ATTGCCCTAGTAACGGCGAG--
TGAAGCGGCAACAGCTCAAA-TTTGAAATCTGGCCC---------
TAGCGGTCCGAGTTGTAATTTGTAGAGGATGCTTTTGGTTAGGT-
GCCTTCTGAGTTCCCTGGAACGGGACGCCAGAGAGGGTGAGAGCCCCGTACGG
TTGGA-
CACCGAGCCTCTATATAGCTCCTTCGACGAGTCGAGTAGTTTGGGAATGCTGCT
CTAAATGGGAGGTAAATTTCTTCTAAAGCTAAATACCGGCCAGAGACC-

GATAGCGCACAAGTAGAGTGATCGAAAGATGAAAAGTACTTTGAAAAGAGGG
TTAAATAGCACGTGAAATTGTTGAAAGGGAAGCG-
TTTGCGACCAGACTTTTTCCAGGCGGATCATCCGGTG-TTC-
TCACCGGTGCACTTCGCCTGG--TTTAGGCCAGCATCGGTTCT---
CTTAGGGGGATAAAGGCTTGGGGAACGTAGCTCC----CTC--GGGAGTGTT-
ATAGCCCCTCGCGTAATACCCTT-CGGGGGACCGAGGAACGCGC-ATC---
TGCAAGGATGCTGGCGTAATGGTCGTCAACGACCCGTCTTGAAACACGGACCA
AGGAGTCGAACATTTGTGCGAGTGTTTGGGTG--
TTAAACCCTCACGCGTAATGAAAGTGAACGGAGGTGAGAGCCCTT-------
CGGGGTGCATCATCGACCGATCCTGATG-
TCTTCGGATGGATTTGAGTAAGAGCATAACTGTTCGGACCCGAAAGATGGTGA
ACTATGCGTGGATAGGGTGAAGCCAGAGGAAACTCTGGTGGAGGCTCGCAGCG
GTTCTGACGTGCAAATCGATCGTCAAATCTGCGCATGGGGGCGAAA--------------

GACTTATCGAACCATC--

TAGTAGCTGGTTACCGCCGAAGTTTCCCTCAGGATAGCAGTGTTG--
TCTTCAGTTTTATGAGGTAAAGCGAATGATTAGGGACTCGGGGGCGCTATATTG
CCTTCATCCATTCTCAAACTTTAAATATGTAAGAAGCCCTTGTTACTTAATTGA
ACGTGGGCATTCGAATGTACCAACACTAGTGGGCCATTTTTGGTAAGCAGAAC
TGGCGATGCGGGATGAACCGAACGCGGGGTTAAGGTGCCAGAGTGGACGCTCA
TCAGACACCACAAAAGGTGTTAGTACATCTTGACAGC--------------------------------
----

AGGACGGTGGCCATGGAAGTCGGAATCCGCTAAGGACTGTGTAACAACTCACC
TGCCGAATGTACTAGCCCTGAAAATGGATGGCGCTCAAGCGTCT-
CACCCATACCTCGCCCTTAGGGTAGAAACGATGCCCTAAGG-
AGTAGGCGGCCGTGG--AGG---

>Ruwenzoria_pseudoannulata_MUCL_51394

--GCTCAAA-
TTTGAAATCTGGCCC---------
TAGCGGTCCGAGTTGTAATTTGTAGAGGATGCTTTTGGCGAGGT-
GCCTTCCGAGTTCCCTGGAACGGGACGCCGGAGAGGGTGAGAGCCCCGTACGG
TTGGA-
CGCCGAGCCTCTATATAGCTCCTTCGACGAGTCGAGTAGTTTGGGAATGCTGCT
CTAAATGGGAGGTAAATTTCTTCTAAAGCTAAATACCGGCCAGAGACC-
GATAGCGCACAAGTAGAGTGATCGAAAGATGAAAAGCACTTTGAAAAGAGGG
TTAAATAGCACGTGAAATTGTTGAAAGGGAAGCG-
TTTGCGACCAGACTTTTTCCGGGCGGATCATCCGGGG-TTT-
TCCCCGGTGCACTTCGCCCGG--TTTAGGCCAGCATCGGTTTC---
CTTAGGGGGATAAAGGCTTGGGGAACGTAGCTCC----CTC--GGGAGTGTT-
ATAGCCCCTTGCGTAATACCCTT-CGGGGGACCGAGGAACGCGC-TTC---
GGCAAGGATGCTGGCGTAATGGTCGTCAACGACCCGTCTTGAAACACGGACCA
AGGAGTCGAACATTTGTGCGAGTGTTTGGGTG--
TCAAACCCTCACGCGTAATGAAAGTGAACGGAGGTGAGAGCCCTT-------
ACGGGTGCATCATCGACCGATCCTGATG-
TCTTCGGATGGATTTGAGTAAGAGCATAACTGTTCGGACCCGAAAGATGGTGA
ACTATGCGTGGATAGGGTGAAGCCAGAGGAAACTCTGGTGGAGGCTCGCAGCG
GTTCTGACGTGCAAATCGATCGTCAAATCTGCGCATGGGGCGAAA--------------

GACTTATCGAACCATC--

TAGTAGCTGGTTACCGCCGAAGTTTCCCTCAGGATAGCAGTGTTG--
TCTTCAGTTTTATGAGGTAAAGCGAATGATTAGGGACTCGGGGGCGCTATATTG
CCTTCATCCATTCTCAAACTTTAAATATGTAAGAAGCCCTTGTTACTTAATTGA
ACGTGGGCATTCGAATGTACCAACACTAGTGGGCCATTTTTGGTAAGCAGAAC
TGGCGATGCGGGATGAACCGAACGCGGGGTTAAGGTGCCAGAGTGGACGCTCA
TCAGACACCACAAAAGGTGTTAGTACATCTTGACAGC---------------------------------
----

AGGACGGTGGCCATGGAAGTCGGAATCCGCTAAGGACTGTGTAACAACTCACC
TGCCGAATGTACTAGCCCTGAAAATGGATGGCGCTCAAGCGTCT-
CACCCATACCTCGCCCTTAGGG--

>J_multiformis_CBS_119016

---CC-
--------TAGCGGTCCGAGTTGTAATTTGCAGAGGATGCTTTTGGTGCGGT-
GCCTTCCGAGTTCCCTGGAACGGGACGCCGGAGAGGGTGAGAGCCCCGTACGG
TTGGA-
CACCTACCCTATACATAGCTCCTTCGACGAGTCGAGTAGTTTGGGAATGCTGCT
CTAAATGGGAGGTAAATTTCTTCTAAAGCTAAATACCGGCCAGAGACC-
GATAGCGCACAAGTAGAGTGATCGAAAGATGAAAAGCACTTTGAAAAGAGGG
TTAAATAGCACGTGAAATTGTTGAAAGGGAAGCG-
TTTGCGACCAGACCTTTTCCAGGCGGATCATCCGGCG-TTC-
TCGCCGGTGCACTCCGCCTGG--TCTAGGCCAGCATCGGTTTC---
CTTAGGGGGATAAAGGCCTGGGGAACGTAGCTCT----TCA--GGGAGTGTT-
ATAGCCCCTAGCGTAATACCCTT-CAGGGGACCGAGGACCGCGC-TTC---
GGCAAGGATGCTGGCGTAATGGTCGTCAACGACCCGTCTTGAAACACGGACCA
AGGAGTCGAACATTTGTGCGAGTGTTTGGGTG--
TCAAACCCTCACGCGTAATGAAAGTGAACGGAGGTGAGAGCCCTT-------
ACGGGTGCATCATCGACCGATCCTGAAG-
TCTTCGGATGGATTTGAGTAAGAGCATAACTGTTCGGACCCGAAAGATGGTGA
ACTATGCGTGGATAGGGTGAAGCCAGAGGAAACTCTGGTGGAGGCTCGCAGCG
GTTCTGACGTGCAAATCGATCGTCAAATCTGCGCATGGGGGCGAAA--------------

GACTTATCGAACCATC---

TAGTAGCTGGTTACCGCCGAAGTTTCCCTCAGGATAGCAGTGTTG--
TCTTCAGTTTTATGAGGTAAAGCGAATGATTAGGGACTCGGGGGCGCTATTTTG
CCTTCATCCATTCTCAAACTTTAAATATGTAAGAAGCCCTTGTTGCTTAATTGA
ACGTGGGCATTCGAATGTATCAACACTAGTGGGCCATTTTTGGTAAGCAGAACT
GGCGATGCGGGATGAACCGAACGCGGGGTTAAGGTGCCAGAGTGGACGCTCAT
CAGACACCACAAAAGGTGTTAGTACATCTTGACAGC------------------------------------
----

AGGACGGTGGCCATGGAAGTCGGAATCCGCTAAGGACTGTGTAACAACTCACC
TGCCGAATGTACTAGCCCTGAAAATGGATGGCGCTCAAGCGTCT-
CACCCATACCTCGCCCTTAGGGTAGAAACGATGCCCTAAGG-
AGTAGGCGGCCG---

>H_griseobrunneum_CBS_331_73

---A-
TTTGAAATCTGGCCC---------
TAGTGGTCCGAGTTGTAATTTGTAGAGGATGCTTTGGGTGCGGT-
GCCTTCTGAGTTCCCTGGAACGGGACGCCAGAGAGGGTGAGAGCCCCGTACGG
TTGGA-
CACCTAGCCTATATATAGCTCCTTCGACGAGTCGAGTAGTTTGGGAATGCTGCT
CTAAATGGGAGGTATATTTCTTCTAAAGCTAAATACCGGCCAGAGACC-
GATAGCGCACAAGTAGAGTGATCGAAAGATGAAAAGCACTTTGAAAAGAGGG
TTAAATAGCACGTGAAATTGTTGAAAGGGAAGCG-
TTTGCGACCAGACCTTTTCCGGGCGGATCATCCGGCG-TTC-
TCGCCGGTGCACTTCGCCCGG--TTTAGGCCAGCATCGGTTTT---
CTTAGGGGGATAAAGGCCAGGGGAACGTAGCTCT----TTC--GGGAGTGTT-
ATAGCCCCTGGTGTAATACCCTT-CAGGGGACCGAGGACCGCGC-TTT---
TGCAAGGATGCTGGCGTAATGGTCGTCAACGACCCGTCTTGAAACACGGACCA
AGGAGTCGAACATTGGTGCGAGTGTTTGGGTG--
TTAAACCCTCACGCGTAATGAAAGTGAACGGAGGTGAGAGCCCTT-------
ACGGGTGCATCATCGACCGATCCTGATG-
TCTTCGGATGGATTTGAGTAAGAGCATCACTGTTCGGACCCGAAAGATGGTGA
ACTATGCGTGGATAGGGTGAAGCCAGAGGAAACTCTGGTGGAGGCTCGCAGCG
GTTCTGACGTGCAAATCGATCGTCAAATCTGCGCATGGGGGCGAAA--------------

GACTTATCGAACCATC--

TAGTAGCTGGTTACCGCCGAAGTTTCCCTCAGGATAGCAGTGTTG--
TCTTCAGTTTTATGAGGTAAAGCGAATGATTAGGGACTCGGGGGCGCTATATTG
CCTTCATCCATTCTCAAACTTTAAATATGTAAGAAGCCCTTGTTACTTAATTGA
ACGTGGGCATTCGAATGTACCAACACTAGTGGGCCATTTTTGGTAAGCAGAAC
TGGCGATGCGGGATGAACCGAACGCGGGGTTAAGGTGCCAGAGTGGACGCTCA
TCAGACACCACAAAAGGTGTTAGTACATCTTGACAGC---------------------------------
----

AGGACGGTGGCCATGGAAGTCGGAATCCGCTAAGGACTGTGTAACAACTCACC
TGCCGAATGTACTAGCCCTGAAAATGGATGGCGCTCAAGCGTCT-
CACCCATACCTCGCCCTCAGGGTAGAAACGATGCCCTGAGG-
AGTAGGCGGCCGTGG--AGG-TTA--

>H_crocopeplum_CBS_119004

--ATTGCCCTAGTAACGGCGAG--
TGAAGCGGCAACAGCTCAAA-TTTGAAATCTGGCCC---------
TCGTGGTCCGAGTTGTAATTTGCAGAGGATGCTTTTGGTGAGGT-
GCCTTCCGAGTTCCCTGGAACGGGACGCCAGAGAGGGTGAGAGCCCCGTACGG
TTGGA-
CGCCTAGCCTCTTTATAGCTCCTTCGACGAGTCGAGTAGTTTGGGAATGCTGCT
CTAAATGGGAGGTAAATTTCTTCTAAAGCTAAATACCGGCCAGAGACC-
GATAGCGCACAAGTAGAGTGATCGAAAGATGAAAAGCACTTTGAAAAGAGGG
TTAAATAGCACGTGAAATTGTTGAAAGGGAAGCG-
TTTGCGACCAGACTTTTTCCAGGCGGATCATCCGGTG-TTC-
TCACCGGTGCACTTCGTCTGG--TTTAGGCCAGCATCGGTTTT---
CTTAGGGGGATAAAGGCTTAGGGAACGTAGCTCT----TTA--GGGAGTGTT-
ATAGCCCTTTGCGTAATACCTTT-CAGGGGACCGAGGATCGCGC-TCT----
GCAAGGATGCTGGCGTAATGGTCGTCAACGACCCGTCTTGAAACACGGACCAA
GGAGTCGAACATTTGTGCGAGTGTTTGGGTG--
TTAAACCCTCACGCGTAATGAAAGTGAACGGAGGTGAGAGCCCTT-------
ACGGGTGCATCATCGACCGATCCTGATG-
TCTTCGGATGGATTTGAGTAAGAGCATAACTGTTCGGACCCGAAAGATAGTGA
ACTATGCGTGGATAGGGTGAAGCCAGAGGAAACTCTGGTGGAGGCTCGCAGCG
GTTCTGACGTGCAAATCGATCGTCAAATCTGCGCATGGGGGCGAAA--------------

GACTTATCGAACCATC--

TAGTAGCTGGTTACCGCCGAAGTTTCCCTCAGGATAGCAGTGTTG--

TCTTCAGTTTTATGAGGTAAAGCGAATGATTAGGGACTCGGGGGCGCTATTTTG
CCTTCATCCATTCTCAAACTTTAAATATGTAAGAAGCCCTTGTTACTTAATTGA
ACGTGGGCATTCGAATGTACCAACACTAGTGGGCCATTTTTGGTAAGCAGAAC
TGGCGATGCGGGATGAACCGAACGCGGGGTTAAGGTGCCAGAGTGGACGCTCA
TCAGACACCACAAAAGGTGTTAGTACATCTTGACAGC------------------------------
----

AGGACGGTGGCCATGGAAGTCGGAATCCGCTAAGGACTGTGTAACAACTCACC
TGCCGAATGTACTAGCCCTGAAAATGGATGGCGCTCAAGCGTCT-
CACCCATACCTCGCCCTTAGGGTAGAAACGATGCCCTAAGG-
AGTAGGCGGCCGTGG--AGG-TCAGTGACGAAGCCTAGG----------------------------
----

>H_perforatum_CBS_115281

---ATTGCCCTAGTAACGGCGAG--
TGAAGCGGCAACAGCTCAAA-TTTGAAATCTGGCCC---------
TCGTGGTCCGAGTTGTAATTTGTAGAGGATGCTTTTGGCGCGGT-
GCCTTCTGAGTTCCCTGGAACGGGACGCCAGAGAGGGTGAGAGCCCCGTACGG
TTGGA-
CACCTAGCCTCTGTATAGCTCCTTCGACGAGTCGAGTAGTTTGGGAATGCTGCT
CTAAATGGGAGGTAAATTTCTTCTAAAGCTAAATACCGGCCAGAGACC-
GATAGCGCACAAGTAGAGTGATCGAAAGATGAAAAGCACTTTGAAAAGAGGG
TTAAATAGCACGTGAAATTGTTGAAAGGGAAGCG-
TTTGCGACCAGACCTTTTCCTGGCGGATCATCCGGTG-TTC-
TCACCGGTGCACTTCGCTTGG--TTTAGGCCAGCATCGGTTTT---
CTTAGGGGGATAAAGGCTTAGGGAACGTAGCTCC----TCC--GGGAGTGTT-
ATAGCCCTCTGCGTAATACCCTT-CAGGGGACCGAGGACCGCGC-TCT----
GCAAGGATGCTGGCGTAATGGTCGTCAACGACCCGTCTTGAAACACGGACCAA
GGAGTCGAACATTTGTGCGAGTGTTTGGGTG--
TTAAACCCTCACGCGTAATGAAAGTGAACGGAGGTGAGAGCCCTT-------
ACGGGTGCATCATCGACCGATCCTGATG-
TCTTCGGATGGATTTGAGTAAGAGCATAACTGTTCGGACCCGAAAGATGGTGA
ACTATGCGTGGATAGGGTGAAGCCAGAGGAAACTCTGGTGGAGGCTCGCAGCG
GTTCTGACGTGCAAATCGATCGTCAAATCTGCGCATGGGGGCGAAA-------------

GACTTATCGAACCATC---

TAGTAGCTGGTTACCGCCGAAGTTTCCCTCAGGATAGCAGTGTTG--
TCTTCAGTTTTATGAGGTAAAGCGAATGATTAGGGACTCGGGGGCGCTATTTAG
CCTTCATCCATTCTCAAACTTTAAATATGTAAGAAGCCCTTGTTACTTAATTGA
ACGTGGGCATTCGAATGTACCAACACTAGTGGGCCATTTTTGGTAAGCAGAAC
TGGCGATGCGGGATGAACCGAACGCGGGGTTAAGGTGCCAGAGTGGACGCTCA
TCAGACACCACAAAAGGTGTTAGTACATCTTGACAGC-------------------------------
----

AGGACGGTGGCCATGGAAGTCGGAATCCGCTAAGGACTGTGTAACAACTCACC
TGCCGAATGTACTAGCCCTGAAAATGGATGGCGCTCAAGCGTCT-
CACCCATACCTCGCCCTCAGGGTAGAAACGATGCCCTGAGG-
AGTAGGCGGCCGTGG--AGG-TTAGTGACGAAGCCTAG-------------------------------
----

>H_howeanum_MUCL_47599

TGGAACGGGACGCCAGAGAGGGTGAGAGCCCCGTACGGTTGGA-
CACCTACCCTATATATAGCTCCTTCGACGAGTCGAGTAGTTTGGGAATGCTGCT
CTAAATGGGAGGTAAATATCTTCTAAAGCTAAATACCGGCCAGAGACC-
GATAGCGCACAAGTAGAGTGATCGAAAGATGAAAAGCACTTTGAAAAGAGGG
TTAAATAGCACGTGAAATTGTTGAAAGGGAAGCG-
TTTGCGACCAGACTTTTTCCAGGCGGATCATCCGGTG-TTC-
TCACCGGTGCACTTCGCCTGG--TTTAGGCCAGCATCGGTTCT---
CTTAGGGGGATAAAGGCTTGGGGAACGTAGCTCC----TTC--GGGAGTGTT-
ATAGCCCCTTGCGTAATACCCTT-CGGGGGACCGAGGATCGCGC-TCT----
GCAAGGATGCTGGCGTAATGGTCGTCAACGACCCGTCTTGAAACACGGACCAA
GGAGTCGAACATTTGTGCGAGTGTTTGGGTG--
TTAAACCCTCACGCGTAATGAAAGTGAACGGAGGTGAGAGCCCTT-------

ACGGGTGCATCATCGACCGATCCTGATG-
TCTTCGGATGGATTTGAGTAAGAGCATAACTGTTCGGACCCGAAAGATGGTGA
ACTATGCGTGGATAGGGTGAAGCCAGAGGAAACTCTGGTGGAGGCTCGCAGCG
GTTCTGACGTGCAAATCGATCGTCAAATCTGCGCATGGGGGCGAAA---------------
----
GACTTATCGAACCATC---

TAGTAGCTGGTTACCGCCGAAGTTTCCCTCAGGATAGCAGTGTTG--
TTTTCAGTTTTATGAGGTAAAGCGAATGATTAGGGACTCGGGGGCGCTATTTAG
CCTTCATCCATTCTCAAACTTTAAATATGTAAGAAGCCCTTGTTACTTAATTGA
ACGTGGGCATTCGAATGTATCAACACTAGTGGGCCATTTTTGGTAAGCAGAACT
GGCGATGCGGGATGAACCGAACGCGGGGTTAAGGTGCCAGAGTGGACGCTCAT
CAGACACCACAAAAGGTGTTAGTACATCTTGACAGC--------------------------------------
----

AGGACGGTGGCCATGGAAGTCGGAATCCGCTAAGGACTGTGTAACAACTCACC
TGCCGAATGTACTAGCCCTGAAAATGGATGGCGCTCAAGCGTCT-
CACCCATACCTCGCCCTTAGGGTAGAAACGATGCCCTAAGG-
AGTAGGCGGCCGTGG--GGG-TCAGTGACGAAGCCTAGG---------------------------------
----

>H_ticinense_CBS_115271

---TGCCCTAGTAACGGCGAG--
TGAAGCGGCAACAGCTCAAA-TTTGAAATCTGGCCC---------
TCGCGGTCCGAGTTGTAATTTGTAGAGGATGCTTTTGGTGCGGT-
GCCTTCCGAGTTCCCTGGAACGGGACGCCAGAGAGGGTGAGAGCCCCGTACGG
TTGGA-
CACCTACCCTATACATAGCTCCTTCGACGAGTCGAGTAGTTTGGGAATGCTGCT
CTAAATGGGAGGTAAATTTCTTCTAAAGCTAAATACCGGCCAGAGACC-
GATAGCGCACAAGTAGAGTGATCGAAAGATGAAAAGCACTTTGAAAAGAGGG
TTAAATAGCACGTGAAATTGTTGAAAGGGAAGCG-

TTTGCGACCAGACTTTTTCCAGGCGGATCATCCGGTG-TTC-
TCACCGGTGCACTTCGCCTGG--TTTAGGCCAGCATCGGTTCT---
CTTAGGGGGATAAAGGCTCGGGGAACGTAGCTCC----TTC--GGGAGTGTT-
ATAGCCCCTTGCGTAATACCCTT-CGGGGGACCGAGGATCGCGC-TCT----
GCAAGGATGCTGGCGTAATGGTCGTCAACGACCCGTCTTGAAACACGGACCAA
GGAGTCGAACATTTGTGCGAGTGTCTGGGTG--
TTAAACCCTCACGCGTAATGAAAGTGAACGGAGGTGAGAGCCCTT-------
GCGGGTGCATCATCGACCGATCCTGATG-
TCTTCGGATGGATTTGAGTAAGAGCATAACTGTTCGGACCCGAAAGATGGTGA
ACTATGCGTGGATAGGGTGAAGCCAGAGGAAACTCTGGTGGAGGCTCGCAGCG
GTTCTGACGTGCAAATCGATCGTCAAATCTGCGCATGGGGGCGAAA--------------

GACTTATCGAACCATC---

TAGTAGCTGGTTACCGCCGAAGTTTCCCTCAGGATAGCAGTGTTG--
TTTTCAGTTTTATGAGGTAAAGCGAATGATTAGGGACTCGGGGGCGCTATTTAG
CCTTCATCCATTCTCAAACTTTAAATATGTAAGAAGCCCTTGTTGCTTAATTGA
ACGTGGGCATTCGAATGTATCAACACTAGTGGGCCATTTTTGGTAAGCAGAACT
GGCGATGCGGGATGAACCGAACGCGGGGTTAAGGTGCCAGAGTGGACGCTCAT
CAGACACCACAAAAGGTGTTAGTACATCTTGACAGC--------------------------------
----

AGGACGGTGGCCATGGAAGTCGGAATCCGCTAAGGACTGTGTAACAACTCACC
TGCCGAATGTACTAGCCCTGAAAATGGATGGCGCTCAAGCGTCT-
CACCCATACCTCGCCCTTAGGGTAGAAACGATGCCCTAAGG-
AGTAGGCGGCCGTGG--GGG-TCAG---

>H_haematostroma_MUCL_53301

---CC-
--------TCGCGGTCCGAGTTGTAATTTGTAGAGGATGCTTTTGGTGCGGC-

GCCTTCCGAGTTCCCTGGAACGGGACGCCGGAGAGGGTGAGAGCCCCGTACGG
TTGGA-
CGCCTAGCCTATATATAGCTCCTTCGACGAGTCGAGTAGTTTGGGAATGCTGCT
CTAAATGGGAGGTAAATTTCTTCTAAAGCTAAATACCGGCCAGAGACC-
GATAGCGCACAAGTAGAGTGATCGAAAGATGAAAAGCACTTTGAAAAGAGGG
TTAAATAGCACGTGAAATTGTTGAAAGGGAAGCG-
TTTGCGACCAGACTTTTTCCAGGGGAATCATCCGGTG-TTC-
TCACCGGTGCACTTCCCCTGG--TTTAGGCCAGCGTCGGTTCT---
CTTAGGGGGATAAAGGCTTGGGGAACGTAGCTCT----TTC--GGGAGTGTT-
ATAGCCCCTCGCGTAATACCCTT-CGGGGGACCGAGGATCGCGC-TCT----
GCAAGGACGCTGGCGTAATGGTCGTCAACGACCCGTCTTGAAACACGGACCAA
GGAGTCGAACATTTGTGCGAGTGTTTGGGTG--
TTAAACCCTCACGCGTAATGAAAGTGAACGGAGGTGAGAGCCCTC-------
ACGGGTGCATCATCGACCGATCCTGATG-
TCTTCGGATGGATTTGAGTAAGAGCATAACTGTTCGGACCCGAAAGATGGTGA
ACTATGCGTGGATAGGGTGAAGCCAGAGGAAACTCTGGTGGAGGCTCGCAGCG
GTTCTGACGTGCAAATCGATCGTCAAATCTGCGCATGGGGGCGAAA---------------
----
GACTTATCGAACCATC--

TAGTAGCTGGTTACCGCCGAAGTTTCCCTCAGGATAGCAGTGTTG--
TCTTCAGTTTTATGAGGTAAAGCGAATGATTAGGGACTCGGGGGCGCTATTTTG
CCTTCATCCATTCTCAAACTTTAAATATGTAAGAAGCCCTTGTTACTTAGTTGA
ACGTGGGCATTCGAATGTACCAACACTAGTGGGCCATTTTTGGTAAGCAGAAC
TGGCGATGCGGGATGAACCGAACGCGGGGTTAAGGTGCCAGAGTGGACGCTCA
TCAGACACCACAAAAGGTGTTAGTACATCTTGACAGC----------------------------------
----

AGGACGGTGGCCATGGAAGTCGGAATCCGCTAAGGACTGTGTAACAACTCACC
TGCCGAATGTACTAGCCCTGAAAATGGATGGCGCTCAAGCGTCT-
CACCCATACCTCGCCCTTAGGGTAGAAACGAAGCCCTAAGG-
AGTAGGCGGCCGTGG--AGG-TCA---

>H_investiens_CBS_118183

---ATTGCCCTAGTAACGGCGAG--
TGAAGCGGCAATAGCTCAAA-TTTGAAATCTGGCCC---------
TCGCGGTCCGAGTTGTAATTTGTAGAGGATGCTTTTGGTGCGGT-
GCCTTCTGAGTTCCCTGGAACGGGACGCCAAAGAGGGTGAGAGCCCCGTACGG
TTGGA-
CACCTAGCCTATATATAGCTCCTTCGACGAGTCGAGTAGTTTGGGAATGCTGCT
CTAAATGGGAGGTAAATTTCTTCTAAAGCTAAATACCGGCCAGAGACC-
GATAGCGCACAAGTAGAGTGATCGAAAGATGAAAAGCACTTTGAAAAGAGGG
TTAAATAGCACGTGAAATTGTTGAAAGGGAAGCG-
TTTGCGACCAGACCTTTTCTAGGGGGATCATCCGGCG-TTC-
TCGCCGGTGCACTTCCCTTAG--TTTAGGCCAGCATCGGTTCC---
CTTAGGGGGATAAAGGCTTGGGGAACGTAGCTCT----CTA--GGGAGTGTT-
ATAGCCCCTTGCGTAATACCCCT-CGGGGGACCGAGGACCGCGC-TCT----
GCAAGGATGCTGGCGTAATGGTCGTCAACGACCCGTCTTGAAACACGGACCAA
GGAGTCGAACATTTGTGCGAGTGTTTGGGTG--
TTAAACCCTCACGCGTAATGAAAGTGAACGGAGGTGAGAGCCCTC-------
GCGGGTGCATCATCGACCGATCCTGATG-
TCTTCGGATGGATTTGAGTAAGAGCATAACTGTTCGGACCCGAAAGATGGTGA
ACTATGCGTGGATAGGGTGAAGCCAGAGGAAACTCTGGTGGAGGCTCGCAGCG
GTTCTGACGTGCAAATCGATCGTCAAATCTGCGCATGGGGGCGAAA--------------

GACTTATCGAACCATC---

TAGTAGCTGGTTACCGCCGAAGTTTCCCTCAGGATAGCAGTGTTG--
TCTTCAGTTTTATGAGGTAAAGCGAATGATTAGGGACTCGGGGGCGCTATTTTG
CCTTCATCCATTCTCAAACTTTAAATATGTAAGAAGCCCTTGTTACTTAGTTGA
ACGTGGGCATTTGAATGTATCAACACTAGTGGGCCATTTTTGGTAAGCAGAACT
GGCGATGCGGGATGAACCGAACGCGGGGTTAAGGTGCCAGAGTGGACGCTCAT
CAGACACCACAAAAGGTGTTAGTACATCCAGACAGC--------------------------------
----

AGGACTGTGGCCATGGAAGTCGGAATCAGCTAAGGACTGTGTAACAACTCACC
TGCCGAATGTACTAGCCCTGAAAATGGATGGCGCTCAAGCGTCT-
CACCCATACCCCGCCCTTAGGGTAGAAACGAAGCCCTAAGG-
AGTAGGCGGCCGTGG--AGG-TCAGTGACGAAGCCTAG-------------------------------
----

>H_pulicicidum_CBS_122622

---GCGAG--
TGAAGCGGCAACAGCTCAAA-TTTGAAATCTGGCCC---------
TAGTGGTCCGAATTGTAATTTGTAGAGGATGCTTTGGTGCGGT-
GCCTTCCGAGTTCCCTGGAACGGGACGCCGGAGAGGGTGAGAGCCCCGTACGG
TTGGA-
CACCTAGCCTCTGTATAGCTCCTTCGACGAGTCGAGTAGTTTGGGAATGCTGCT
CTAAATGGGAGGTAAATTTCTTCTAAAGCTAAATACCGGCCAGAGACC-
GATAGCGCACAAGTAGAGTGATCGAAAGATGAAAAGCACTTTGAAAAGAGGG
TTAAATAGCACGTGAAATTGTTGAAAGGGAAGCG-
TTTGCGACCAGACCTTTTCCGGGCGGATCATCCGGCG-TTC-
TCGCCGGTGCACTTCGTCCGG--TCTAGGCCAGCATCGGTTTC---
CTTAGGGGGATAAAGGCTTGGGGAACGTAGCTCT----TTA--GGGAGTGTT-
ATAGCCCCTTGCGTAATACCCTT-CGGGGGACCGAGGACCGCGC-TCT----
GCAAGGATGCTGGCGTAATGGTCGTCAACGACCCGTCTTGAAACACGGACCAA
GGAGTCGAACATTTGTGCGAGTGTTTGGGTG--
TTAAACCCTTACGCGTAATGAAAGTGAACGGAGGTGAGAGCCCTT-------
ACGGGTGCATCATCGACCGATCCTGATG-
TCTTCGGATGGATTTGAGTAAGAGCATAACTGTTCGGACCCGAAAGATGGTGA
ACTATGCGTGGATAGGGTGAAGCCAGAGGAAACTCTGGTGGAGGCTCGCAGCG
GTTCTGACGTGCAAATCGATCGTCAAATCTGCGCATGGGGGCGAAA--------------

GACTTATCGAACCATC---

TAGTAGCTGGTTACCGCCGAAGTTTCCCTCAGGATAGCAGTGTTG--
TCTTCAGTTTTATGAGGTAAAGCGAATGATTAGGGACTCGGGGGCGCTATATTG
CCTTCATCCATTCTCAAACTTTAAATATGTAAGAAGCCCTTGTTACTTAAGTGA
ACGTGGGCATTCGAATGTACCAACACTAGTGGGCCATTTTTGGTAAGCAGAAC
TGGCGATGCGGGATGAACCGAACGCGGGGTTAAGGTGCCAGAGTGGACGCTCA
TCAGACACCACAAAAGGTGTTAGTACATCTNGACAGC----------------------------------
----

AGGACTGTGGCCATGGAAGTCGGAATCAGCTAAGGACTGTGTAACAACTCACC
TGCCGAATGTACTAGCCCTGAAAATGGATGGCGCTCAAGCGTCT-
CACCCATACCTCGCCCTTAGGGTAGAAACGATGCCCTAAGG-
AGTAGGCGGCCGTGG--GGG-TCAG---

>H_lechatii_MUCL_54609

---GCCCTAGTAACGGCGAG--
TGAAGCGGCAACAGCTCAAA-TTTGAAATCTGGCCC---------
TCGCGGTCCGAGTTGTAATTTGTAGAGGATGCTTTTGGCGCGGT-
GCCTTCCGAGTTCCCTGGAACGGGACGCCATAGAGGGTGAGAGCCCCGTACGG
TTGGA-
CGCCTAGCCTCTGTATAGCTCCTTCGACGAGTCGAGTAGTTTGGGAATGCTGCT
CTAAATGGGAGGTAAATTTCTTCTAAAGCTAAATACCGGCCAGAGACC-
GATAGCGCACAAGTAGAGTGATCGAAAGATGAAAAGCACTTTGAAAAGAGGG
TTAAATAGCACGTGAAATTGTTGAAAGGGAAGCG-
TTTGCGACCAGACCTTTTCCAGGCGGATCATCCGGCG-TTC-
TCGCCGGTGCACTTCGCCTGG--TTTAGGCCAGCATCGGTTTC---
CTTAGGGGGATAAAGGCTTAGGGAACGTATCTCT----CTC--GGGAGTGTT-
ATATCCCTTTGCGTAATACCCTT-CGGGGGACCGAGGACCGCGC-TCT----
GCAAGGATGCTGGCGTAATGGTCGTCAACGACCCGTCTTGAAACACGGACCAA
GGAGTCGAACATTTGTGCGAGTGTTTGGGTG--
TTAAACCCTCACGCGTAATGAAAGTGAACGGAGGTGAGAGCCTTT-------
CGGGGCGCATCATCGACCGATCCTGATG-
TCTTCGGATGGATTTGAGTAAGAGCATAACTGTTCGGACCCGAAAGATGGTGA
ACTATGCGTGGATAGGGTGAAGCCAGAGGAAACTCTGGTGGAGGCTCGCAGCG
GTTCTGACGTGCAAATCGATCGTCAAATCTGCGCATGGGGGCGAAA-------------

GACTTATCGAACCATC--

TAGTAGCTGGTTACCGCCGAAGTTTCCCTCAGGATAGCAGTGTTG--
TCTTCAGTTTTATGAGGTAAAGCGAATGATTAGGGACTCGGGGGCGCTATTTG

CCTTCATCCATTCTCAAACTTTAAATATGTAAGAAGCCCTTGTTACTTAGTTGA
ACGTGGGCATTCGAATGTACCAACACTAGTGGGCCATTTTTGGTAAGCAGAAC
TGGCGATGCGGGATGAACCGAACGCGGGGTTAAGGTGCCAGAGTGGACGCTCA
TCAGACACCACAAAAGGTGTTAGTACATCTTGACAGC-------------------------------
----

AGGACGGTGGCCATGGAAGTCGGAATCCGCTAAGGACTGTGTAACAACTCACC
TGCCGAATGTACTAGCCCTGAAAATGGATGGCGCTCAAGCGTCT-
CACCCATACCTCGCCCTTAAGGTAGAAACGATGCCTTAAGG-
AGTAGGCGGCCGTGG--AGG-TCAGTGACGAAGCC-------------------------------------
----

>H_lateripigmentum_MUCL_53304

--A-
TTTGAAATCTGGCCC---------
TCGTGGTCCGAGTTGTAATTTGTAGAGGATGCTTTTGGTGCGGT-
GCCTTCCGAGTTCCCTGGAACGGGACGCCAGAGAGGGTGAGAGCCCCGTACGG
TTGGA-
CGCCTAGCCTACATATAGCTCCTTCGACGAGTCGAGTAGTTTGGGAATGCTGCT
CTAAATGGGAGGTAAATTTCTTCTAAAGCTAAATACCGGCCAGAGACC-
GATAGCGCACAAGTAGAGTGATCGAAAGATGAAAAGCACTTTGAAAAGAGGG
TTAAATAGCACGTGAAATTGTTGAAAGGGAAGCG-
TTTACGACCAGACCTTCTCCGGGGGGATCATCAGGTG-TTC-
TCACCTGTGCACTTCCCCCGG--TTTAGGCCAGCATCGGTTTC---
CTTAGGGGGATAAAGGCTCGGGGAACGTAGCTCC----TTA--GGGAGTGTT-
ATAGCCCCTTGCGTAATACCCCT-CGGGGGACCGAGGACCGCGC-TCT----
GCAAGGATGCTGGCGTAATGGTCGTCAACGACCCGTCTTGAAACACGGACCAA
GGAGTCGAACATTTGTGCGAGTGTTTGGGTG--
TTAAACCCTCACGCGTAATGAAAGTGAACGGAGGTGAGAGCCCTT-------
ACGGGTGCATCATCGACCGATCCTGATG-
TCTTCGGATGGATTTGAGTAAGAGCATAACTGTTCGGACCCGAAAGATGGTGA
ACTATGCGTGGATAGGGTGAAGCCAGAGGAAACTCTGGTGGAGGCTCGCAGCG
GTTCTGACGTGCAAATCGATCGTCAAATCTGCGCATGGGGGCGAAA--------------

GACTTATCGAACCATC--

TAGTAGCTGGTTACCGCCGAAGTTTCCCTCAGGATAGCAGTGTTG--
TCTTCAGTTTTATGAGGTAAAGCGAATGATTAGGGACTCGGGGGCGCTATTTTG
CCTTCATCCATTCTCAAACTTTAAATATGTAAGAAGCCCTTGTTACTTAGTTGA
ACGTGGGCATTCGAATGTACCAACACTAGTGGGCCATTTTTGGTAAGCAGAAC
TGGCGATGCGGGATGAACCGAACGCGGGGTTAAGGTGCCAGAGTGGACGCTCA
TCAGACACCACAAAAGGTGTTAGTACATCCAGACAGC-----------------------------------
----

AGGACGGTGGCCATGGAAGTCGGAATCCGCTAAGGACTGTGTAACAACTCACC
TGCCGAATGTACTAGCCCTGAAAATGGATGGCGCTCAAGCGTCT-
CACCCATACCTCGCCCTTAGGGTAGGATCGATGCCCTAAGG-
AGTAGGCGGCCGTGG--GGG-TCA---

>H_lividipigmentum_STMA_14045

---GCCCTAGTAACGGCGAG--
TGAAGCGGCAACAGCTCAAA-TTTGAAATCTGGCCC---------
TCGCGGTCCGAGTTGTAATTTGTAGAGGATGCTTTTGGTGCGGC-
GCCTTCCGAGTTCCCTGGAACGGGACGCCTTAGAGGGTGAGAGCCCCGTACGG
TTGGA-
CGCCAATCCTATATATAGCTCCTTCAACGAGTCGAGTAGTTTGGGAATGCTGCT
CTAAATGGGAGGTAAATTTCTTCTAAAGCTAAATACCGGCCAGAGACC-
GATAGCGCACAAGTAGAGTGATCGAAAGATGAAAAGCACTTTGAAAAGAGGG
TTAAATAGCACGTGAAATTGTTGAAAGGGAAGCG-
TTTGCGACCAGACCTTCTCCAGGCGGATCAACCAACG-TTC-
TCGATGGTGCACTTCGCCTGG--TTTAGGCCAGCATCGGTTTT---
CCTAGGGGGATAAAGGCTTGGGGAACGTGGCTCC----CTC--GGGAGTGTT-
ATAGCCCCTCGCGTAATACCCTT-AGGGGGACCGAGGACCGCGC-TTC---
GGCAAGGATGCTGGCGTAATGGTCGTCAACGACCCGTCTTGAAACACGGACCA
AGGAGTCGAACATTTGTGCGAGTGTTTGGGTG--

TCAAACCCTCACGCGTAATGAAAGTGAACGGAGGTGAGAGCCCTT-------
ACGGGTGCATCATCGACCGATCCTGATG-
TCTTCGGATGGATTTGAGTAAGAGCATAACTGTTCGGACCCGAAAGATGGTGA
ACTATGCGTGGATAGGGTGAAGCCAGAGGAAACTCTGGTGGAGGCTCGCAGCG
GTTCTGACGTGCAAATCGATCGTCAAATCTGCGCATGGGGGCGAAA--------------

GACTTATCGAACCATC---

TAGTAGCTGGTTACCGCCGAAGTTTCCCTCAGGATAGCAGTGTTG--
TCTTCAGTTTTATGAGGTAAAGCGAATGATTAGGGACTCGGGGGCGCTATTTTG
CCTTCATCCATTCTCAAACTTTAAATATGTAAGAAGCCCTTGTTACTTAGTTGA
ACGTGGGCATTCGAATGTACCAACACTAGTGGGCCATTTTTGGTAAGCAGAAC
TGGCGATGCGGGATGAACCGAACGCGGGGTTAAGGTGCCAGAGTGGACGCTCA
TCAGACACCACAAAAGGTGTTAGTACATCTTGACAGC------------------------------
----

AGGACGGTGGCCATGGAAGTCGGAATCCGCTAAGGACTGTGTAACAACTCACC
TGCCGAATGTACTAGCCCTGAAAATGGATGGCGCTCAAGCGTCT-
CACCCATACCTCGCCCTTAGGGTAGAAACGATGCCCTAAGG-
AGTAGGCGGCCGTGG--AGG-TCAGTGACGAAGCCTAGGC---------------------------
----

>Rhopalostroma_angolense_CBS_126414

GAAGCGGCAACAGCTCAAA-TTTGAAATCTGGCCC---------
TAGCGGTCCGAGTTGTACTTTGTAGAGGATGCTTTTGGCGAGGT-
GCCTTCCGAGTTCCCTGGAACGGGACGCCGGAGAGGGTGAGAGCCCCGTACGG
TTGGA-
CGCCGAGCCTCTGTATAGCTCCTTCGACGAGTCGAGTAGTTTGGGAATGCTGCT
CTAAATGGGAGGTAAATTTCTTCTAAAGCTAAATACCGGCCAGAGACC-
GATAGCGCACAAGTAGAGTGATCGAAAGATGAAAAGCACTTTGAAAAGAGGG

TTAAATAGCACGTGAAATTGTTGAAAGGGAAGCG-
TTTGCGACCAGACTTTTTCCGGGGGGATCATCCGGCG-TTC-
TCGCCGGTGCACTTCCCCCGG--TCGAGGCCAGCATCGGTTTC---
CTTAGGGGGATAAAGGCCCGGGGAACGTGGCTCC----TTC--GGGAGTGTT-
ATAGCCCCGGGCGTAATACCCCT-CGGGGGACCGAGGAACGCGC-TCT----
GCAAGGATGCTGGCGTAATGGTCGTCAACGACCCGTCTTGAAACACGGACCAA
GGAGTCGAACATTTGTGCGAGTGTTTGGGTG--
TCAAACCCTCACGCGTAATGAAAGTGAACGGAGGTGAGAGCCCTC-------
GCGGGTGCATCATCGACCGATCCTGATG-
TCTTCGGATGGATTTGAGTAAGAGCATAACTGTTCGGACCCGAAAGATGGTGA
ACTATGCGTGGATAGGGTGAAGCCAGAGGAAACTCTGGTGGAGGCTCGCAGCG
GTTCTGACGTGCAAATCGATCGTCAAATCTGCGCATGGGGGCGAAA--------------

GACTTATCGAACCATC---

TAGTAGCTGGTTACCGCCGAAGTTTCCCTCAGGATAGCAGTGTTGT-
TCTTCAGTTTTATGAGGTAAAGCGAATGATTAGGGACTCGGGGGCGCTATATTG
CCTTCATCCATTCTCAAACTTTAAATATGTAAGAAGCCCTTGTTACTTAGTTGA
ACGTGGGCATTCGAATGTACCAACACTAGTGGGCCATTTTTGGTAAGCAGAAC
TGGCGATGCGGGATGAACCGAACGCGGGGTTAAGGTGCCAGAGTGGACGCTCA
TCAGACACCACAAAAGGTGTTAGTACATCTTGACAGC-------------------------------------
----

AGGACGGTGGCCATGGAAGTCGGAATCCGCTAAGGACTGTGTAACAACTCACC
TGCCGAATGTACTAGCCCTGAAAATGGATGGCGCTCAAGCGTCT-
CACCCATACCTCGCCCTTAGGGTAGAAACGATGCCCTAAGG-
AGTAGGCGGCCGTGG--AGG-TTAGTGACGAAGCCTAGGGC------------------------------
----

>H_hinnuleum_MUCL_3621

---TGCCCTAGTAACGGCGAG--

TGAAGCGGCAACAGCTCAAA-TTTGAAATCTGGCCC---------
TCGCGGTCCGAGTTGTAATTTGTAGAGGATGCTTTGGGCGCGGC-
GCCTTCCGAGTTCCCTGGAACGGGACGCCAGAGAGGGTGAGGGCCCCGTACGG
TTGGA-
CGCCTAGCCTATGTATAGCTCCTTCGACGAGTCGAGTAGTTTGGGAATGCTGCT
CTAAATGGGAGGTAAATTTCTTCTAAAGCTAAATACCGGCCAGAGACC-
GATAGCGCACAAGTAGAGTGATCGAAAGATGAAAAGCACTTTGAAAAGAGGG
TTAAATAGCACGTGAAATTGTTGAAAGGGAAGCG-
TTTGCGACCAGACCTTCTCCGGGGGGATCATCCGGTG-TTC-
TCACCGGTGCACTCCCCTCGG--TCTAGGCCAGCATCGGTTTC---
CTTAGGGGGATAAAGGCCTGGGGAACGTAGCTCC----TTC--GGGAGTGTT-
ATAGCCCCTTGCGTAATACCCCT-CGGGGGACCGAGGACCGCGC-TTC---
GGCAAGGATGCTGGCGTAATGGTCGTCAACGACCCGTCTTGAAACACGGACCA
AGGAGTCGAACATTTGTGCGAGTGTTTGGGTG--
TTAAACCCTCACGCGTAATGAAAGTGAACGGAGGTGAGAGCCCTC-------
GCGGGTGCATCATCGACCGATCCTGATG-
TCTTCGGATGGATTTGAGTAAGAGCATAACTGTTCGGACCCGAAAGATAGTGA
ACTATGCGTGGATAGGGTGAAGCCAGAGGAAACTCTGGTGGAGGCTCGCAGCG
GTTCTGACGTGCAAATCGATCGTCAAATCTGCGCATGGGGGCGAAA---------------
----
GACTAATCGAACTATC---

TAGTAGCTGGTTACCGCCGAAGTTTCCCTCAGGATAGCAGTGTTG--
TCTTCAGTTTTATGAGGTAAAGCGAATGATTAGGGACTCGGGGGCGCTATATTG
CCTTCATCCATTCTCAAACTTTAAATATGTAAGAAGCCCTTGTTACTTAGTTGA
ACGTGGGCATTCGAATGTACCAACACTAGTGGGCCATTTTTGGTAAGCAGAAC
TGGCGATGCGGGATGAACCGAACGCGGGGTTAAGGTGCCAGAGTGGACGCTCA
TCAGACACCACAAAAGGTGTTAGTACATCCTGACAGC---------------------------------
----

AGGACTGTGGCCATGGAAGTCGGAATCAGCTAAGGACTGTGTAACAACTCACC
TGCCGAATGTACTAGCCCTGAAAATGGATGGCGCTCAAGCGTCT-
CACCCATACCTCGCCCTTAGGGTAGAATCGATGCCCTAAGG-
AGTAGGCGGCCGTGG--AGG-TCAGTGACGAAGCC--

>P_hunteri_MUCL_52673

------------------------------------GCTTTGGGCGCGGC-
GCCTTCCAAGTTCCCTAGAACGGGACGCCTTAGAGGGTGAGAGCCCCGTACGG
TTGGA-
CGCCTAGCCTATGTATAGCTCCTTCGACGAGTCGAGTAGTTTGGGAATGCTGCT
CTAAATGGGAGGTAAATTTCTTCTAAAGCTAAATACCGGCCAGAGACC-
GATAGCGCACAAGTAGAGTGATCGAAAGATGAAAAGTACTTTGAAAAGAGGG
TTAAATAGCACGTGAAATTGTTGAAAGGGAAGCG-
TTTGCGACCAGACCTTTTCCAGGCGGATCATCCGGCG-TTC-
TCGCCGGTGCACTTCGCCTGG--TTTAGGCCAGCATCGGTTTT---
CCCAGGGGGATAAAGGCGGTGGGAACGTAGCTCT----TTC--GGGAGTGTT-
ATAGCCCGCCGCGTAATACCCTT-GGGGGGACCGAGGACCGCGC-TTC---
GGCAAGGATGCTGGCGTAATGGTCGTCAACGACCCGTCTTGAAACACGGACCA
AGGAGTCGAACATTTGTGCGAGTGTTTGGGTG--
TCAAACCCTCACGCGTAATGAAAGTGAACGGAGGTGAGAGCCCCTC------
GCGGGTGCATCATCGACCGATCCTGATG-
TCTTCGGATGGATTTGAGTAAGAGCATAACTGTTCGGACCCGAAAGATGGTGA
ACTATGCGTGGATAGGGTGAAGCCAGAGGAAACTCTGGTGGAGGCTCGCAGCG
GTTCTGACGTGCAAATCGATCGTCAAATCTGCGCATGGGGGCGAAA--------------

GACTTATCGAACCATC--

TAGTAGCTGGTTACCGCCGAAGTTTCCCTCAGGATAGCAGTGTTG--
TCTTCAGTTTTATGAGGTAAAGCGAATGATTAGGGACTCGGGGGCGCTATTTTG
CCTTCATCCATTCTCAAACTTTAAATATGTAAGAAGCCCTTGTTACTTAGTTGA
ACGTGGGCATTCGAATGTACCAACACTAGTGGGCCATTTTTGGTAAGCAGAAC
TGGCGATGCGGGATGAACCGAACGCGGGGTTAAGGTGCCAGAGTGGACGCTCA
TCAGACACCACAAAAGGTGTTAGTACATCTTGACAGC--------------------------------
----

AGGACGGTGGCCATGGAAGTCGGAATCCGCTAAGGACTGTGTAACAACTCACC
TGCCGAATGTACTAGCCCTGAAAATGGATGGCGCTCAAGCGTCT-
CACCCATACCTCGCC--

>P_nicaraguense_CBS_117739

--A-
TTTGAAATCTGGCCC---------
TCGCGGTCCGAGTTGTAATTTGTAGAGGATGCTTTGGGCGCGGC-
GCCTTCCAAGTTCCCTAGAACGGGACGCCTTAGAGGGTGAGAGCCCCGTACGG
TTGGA-
CGCCTAGCCTATGTATAGCTCCTTCGACGAGTCGAGTAGTTTGGGAATGCTGCT
CTAAATGGGAGGTAAATTTCTTCTAAAGCTAAATACCGGCCAGAGACC-
GATAGCGCACAAGTAGAGTGATCGAAAGATGAAAAGTACTTTGAAAAGAGGG
TTAAATAGCACGTGAAATTGTTGAAAGGGAAGCG-
TTTGCGACCAGACCTTTTCCAGGCGGATCATCCGGCG-TTC-
TCGCCGGTGCACTTCGCCTGG--TTTAGGCCAGCATCGGTTTT---
CCCAGGGGGATAAAGGCGGTGGGAACGTAGCTCT----TTC--GGGAGTGTT-
ATAGCCCGCCGCGTAATACCCTT-GGGGGGACCGAGGACCGCGC-TTC---
GGCAAGGATGCTGGCGTAATGGTCGTCAACGACCCGTCTTGAAACACGGACCA
AGGAGTCGAACATTTGTGCGAGTGTTTGGGTG--
TCAAACCCTCACGCGTAATGAAAGTGAACGGAGGTGAGAGCCCCTC------
GCGGGTGCATCATCGACCGATCCTGATG-
TCTTCGGATGGATTTGAGTAAGAGCATAACTGTTCGGACCCGAAAGATGGTGA
ACTATGCGTGGATAGGGTGAAGCCAGAGGAAACTCTGGTGGAGGCTCGCAGCG
GTTCTGACGTGCAAATCGATCGTCAAATCTGCGCATGGGGGCGAAA-------------

GACTTATCGAACCATC---

TAGTAGCTGGTTACCGCCGAAGTTTCCCTCAGGATAGCAGTGTTG--
TCTTCAGTTTTATGAGGTAAAGCGAATGATTAGGGACTCGGGGGCGCTATTTTG
CCTTCATCCATTCTCAAACTTTAAATATGTAAGAAGCCCTTGTTACTTAGTTGA
ACGTGGGCATTCGAATGTACCAACACTAGTGGGCCATTTTTGGTAAGCAGAAC
TGGCGATGCGGGATGAACCGAACGCGGGGTTAAGGTGCCAGAGTGGACGCTCA
TCAGACACCACAAAAGGTGTTAGTACATCTTGACAGC-------------------------------
----

AGGACGGTGGCCATGGAAGTCGGAATCCGCTAAGGACTGTGTAACAACTCACC
TGCCGAATGTACTAGCCCTGAAAATGGATGGCGCTCAAGCGTCT-
CACCCATACCTCGCCCTTAGGGTAGAAACGATGCCCTAAGG-AGTAGGCGGC----

>P_laminosus_MUCL_53305

---GGCGAG--
TGAAGCGGCAACAGCTCAAA-TTTGAAATCTGGCCC---------
TCGCGGTCCGAGTTGTAATTTGCAGAGGATGCTTTGGGTGCGGC-
GCCTTCCAAGTTCCCTAGAACGGGACGCCTTAGAGGGTGAGAGCCCCGTACGG
TTGGA-
CGCCTAGCCTATGTATAGCTCCTTCGACGAGTCGAGTAGTTTGGGAATGCTGCT
CTAAATGGGAGGTAAATTTCTTCTAAAGCTAAATACCGGCCAGAGACC-
GATAGCGCACAAGTAGAGTGATCGAAAGATGAAAAGTACTTTGAAAAGAGGG
TTAAATAGCACGTGAAATTGTTGAAAGGGAAGCG-
TTTGCGACCAGACCTTTTCCAGGCGGATCATCCGGCG-TTC-
TCGCCGGTGCACTTCGCCTGG--TTTAGGCCAGCATCGGTTTT---
CCCAGGGGGATAAAGGCGGCGGGAACGTGGCTCT----TTC--GGGAGTGTT-
ATAGCCCGCCGCGCAATACCCTT-GGGGGGACCGAGGACCGCGC-TTC---
GGCAAGGATGCTGGCGTAATGGTCGTCAACGACCCGTCTTGAAACACGGACCA
AGGAGTCGAACATTTGTGCGAGTGTTTGGGTG--
TCAAACCCTCACGCGTAATGAAAGTGAACGGAGGTGAGAGCCCCTC------
GCGGGTGCATCATCGACCGATCCTGATG-
TCTTCGGATGGATTTGAGTAAGAGCATAACTGTTCGGACCCGAAAGATGGTGA
ACTATGCGTGGATAGGGTGAAGCCAGAGGAAACTCTGGTGGAGGCTCGCAGCG
GTTCTGACGTGCAAATCGATCGTCAAATCTGCGCATGGGGGCGAAA--------------

GACTTATCGAACCATC---

TAGTAGCTGGTTACCGCCGAAGTTTCCCTCAGGATAGCAGTGTTG--
TCTTCAGTTTTATGAGGTAAAGCGAATGATTAGGGACTCGGGGGCGCTATTTTG

CCTTCATCCATTCTCAAACTTTAAATATGTAAGAAGCCCTTGTTACTTAATTGA
ACGTGGGCATTCGAATGTACCAACACTAGTGGGCCATTTTTGGTAAGCAGAAC
TGGCGATGCGGGATGAACCGAACGCGGGGTTAAGGTGCCAGAGTGGACGCTCA
TCAGACACCACAAAAGGTGTTAGTACATCTTGACAGC-------------------------------
----

AGGACGGTGGCCATGGAAGTCGGAATCCGCTAAGGACTGTGTAACAACTCACC
TGCCGAATGTACTAGCCCTGAAAATGGATGGCGCTCAAGCGTCT-
CACCCATACCTCGCCCTTAGGGTAGAAACGATGCCCTAAGG-
AGTAGGCGGCCG--

>H_hypomiltum_MUCL_51845

---ATTGCCCTAGTAACGGCGAG--
TGAAGCGGCAACAGCTCAAA-TTTGAAATCTGGCCC---------
TCGTGGTCCGAATTGTAATTTGTAGAGGATGCTTTTGGTGTGGT-
GCCTTCTGAGTTCCCTGGAACGGGACGCCAGAGAGGGTGAGAGCCCCGTACGG
TTGGA-
CACCTATCCTATATATAGCTCCTTCGACGAGTCGAGTAGTTTGGGAATGCTGCT
CTAAATGGGAGGTAAATTTCTTCTAAAGCTAAATACCGGCCAGAGACC-
GATAGCGCACAAGTAGAGTGATCGAAAGATGAAAAGCACTTTGAAAAGAGGG
TTAAATAGCACGTGAAATTGTTGAAAGGGAAGCG-
TTTGCGACCAGACCTTTTCCTGGCGGATCATCCGGTG-TTC-
TCACCGGTGCACTTCGCTTGG--TTTAGGCCAGCATCGGTTTT---
CTTAGGGGGATAAAGGCCTGGGGCACGTAGCTCT----TCC--GGGAGTGTT-
ATAGCCCCTAGCGTAATGCCCTT-ACGGGGACCGAGGACCGCGC-TTC---
GGCAAGGATGCTGGCGTAATGGTCGTCAACGACCCGTCTTGAAACACGGACCA
AGGAGTCGAACATTTGTGCGAGTGTTTGGGTG--
TTAAACCCTCACGCGTAATGAAAGTGAACGGAGGTGAGAGCCCTT-------
CGGGGTGCATCATCGACCGATCCTGATG-
TCTTCGGATGGATTTGAGTAAGAGCATAACTGTTCGGACCCGAAAGATGGTGA
ACTATGCGTGGATAGGGTGAAGCCAGAGGAAACTCTGGTGGAGGCTCGCAGCG
GTTCTGACGTGCAAATCGATCGTCAAATCTGCGCATGGGGGCGAAA--------------

GACTTATCGAACCATC--

TAGTAGCTGGTTACCGCCGAAGTTTCCCTCAGGATAGCAGTGTTG--
TTTTCAGTTTTATGAGGTAAAGCGAATGATTAGGGACTCGGGGGCTCTTTTTG
CCTTCATCCATTCTCAAACTTTAAATATGTAAGAAGCCCTTGTTACTTAATTGA
ACGTGGGCATTCGAATGTATCAACACTAGTGGGCCATTTTTGGTAAGCAGAACT
GGCGATGCGGGATGAACCGAACGCGGGGTTAAGGTGCCAGAGTGGACGCTCAT
CAGACACCACAAAAGGTGTTAGTACATCCAGACAGC------------------------------
----

AGGACGGTGGCCATGGAAGTCGGAATCCGCTAAGGACTGTGTAACAACTCACC
TGCCGAATGTACTAGCCCTGAAAATGGATGGCGCTCAAGCGTCT-
CACCCATACCTCGCCCTTAAGGTAGAAACGATGCCTTAAGG-
AGTAGGCGGCCGTGG--AGG-TTAGTGACGAAGCCTAGG----------------------------
----

>H_texense_DSM_107933

---------------AATTGTAATTTGTAGAGGATGCTTTTGGTGCGGC-
GCCTTCCGAGTTCCCTGGAACGGGACGCCAGAGAGGGTGAGAGCCCCGTACGG
TTGGA-
TACCTAGCCTCTATATAGCTCCTTCGACGAGTCGGGTAGTTTGGGAATGCTGCT
CTAAACGGGAGGTAAATTTCTTCTAAAGCTAAATACCGGCCAGAGACC-
GATAGCGCACAAGTAGAGTGATCGAAAGATGAAAAGCACTTTGAAAAGAGGG
TTAAATAGCACGTGAAATTGTTGAAAGGGAAGCG-
TTTGCGACCAGACCTTTTCCGGGCGGATCATCCGGTG-TTC-
TCACCGGTGCACTTCGCCCGG--TTTAGGCCAGCATCGGTTTT---
CGTAGGGGGATAAAGGCCTGGGGAACGTATCTCC----TTC--GGGAGTGTT-
ATAGCCCCTCGCGTAATACCCTT-ACGGGGACCGAGGACCGCGC-TCT----
GCAAGGATGCTGGCGTAATGGTCGTCAACGACCCGTCTTGAAACACGGACCAA
GGAGTCGAACATTTGTGCGAGTGTTTGGGTG--
TTAAACCCTCACGCGTAATGAAAGTGAACGGAGGTGAGAGCCCTT-------

CGGGGTGCATCATCGACCGATCCTGATG-
TCTTCGGATGGATTTGAGTAAGAGCATAACTGTTCGGACCCGAAAGATGGTGA
ACTATGCGTGGATAGGGTGAAGCCAGAGGAAACTCTGGTGGAGGCTCGCAGCG
GTTCTGACGTGCAAATCGATCGTCAAATCTGCGCATGGGGGCGAAA---------------
----
GACTTATCGAACCATC--

TAGTAGCTGGTTACCGCCGAAGTTTCCCTCAGGATAGCAGTGTTG--
TTTTCAGTTTTATGAGGTAAAGCGAATGATTAGGGACTCGGGGGCGCTTTATTG
CC-TCATCCATTCTCAAACTTTAAATATGT---

>H_laschii_MUCL_52796

--GCCTCAGTAACGGCGAG--
TGAAGCGGCAACAGCTCAAA-TTTGAAATCTGGCCC---------
TCGTGGTCCGAGTTGTAATTTGTAGAGGATGCTTTTGGTGCGGT-
ACCTTCCGAGTTCCCTGGAACGGGACGCCAGAGAGGGTGAGAGCCCCGTACGG
TCGGA-
TACCTACCCTATATATAGCTCCTTCGACGAGTCGGGTAGTTTGGGAATGCTGCT
CTAAATGGGAGGTAAATTTCTTCTAAAGCTAAATACCGGCCAGAGACC-
GATAGCGCACAAGTAGAGTGATCGAAAGATGAAAAGCACTTTGAAAAGAGGG
TTAAATAGCACGTGAAATTGTTGAAAGGGAAGCG-
TTTACGACCAGACCTCTTCCAGGCGGATCATCCGGTG-TTC-
TCACCGGTGCACTTCGCCTGG--TTTAGGCCAGCATCGGTTTT---
CGTAGGGGGATAAAGGCCTGGGGAACGTATCTCT----CTA--GGGAGTGTT-
ATAGCCCCTCGTGTAATACCCTT-ACGGGGACCGAGGACCGCGC-TCT----

GCAAGGATGCTGGCGTAATGGTCGTCAACGACCCGTCTTGAAACACGGACCAA
GGAGTCGAACATTTGTGCGAGTGTTTGGGTA--
TAAAACCCTCACGCGTAATGAAAGTGAACGGAGGTGAGAGCCCTT-------
CGGGGTGCATCATCGACCGATCCTGATG-
TCTTCGGATGGATTTGAGTAAGAGCATAACTGTTCGGACCCGAAAGATGGTGA
ACTATGCGTGGATAGGGTGAAGCCAGAGGAAACTCTGGTGGAGGCTCGCAGCG
GTTCTGACGTGCAAATCGATCGTCAAATCTGCGCATGGGGGCGAAA---------------
----
GACTTATCGAACCATC---

TAGTAGCTGGTTACCGCCGAAGTTTCCCTCAGGATAGCAGTGTTG--
TTTTCAGTTTTATGAGGTAAAGCGAATGATTAGGGACTCGGGGGCGCTTTATTG
CCTTCATCCATTCTCAAACTTTAAATATGTAAGAAGCCCTTGTTACTTTATTGAA
CGTGGGCATTCGAATGTATCAACACTAGTGGGCCATTTTTGGTAAGCAGAACTG
GCGATGCGGGATGAACCGAACGCGGGGTTAAGGTGCCAGAGTGGACGCTCATC
TGACACCACAAAAGGTGTTAGTACATCTAGACAGT---------------------------------------
----

TGGACGGTGGCCATGGAAGTCGGAATCCGCTAAGGACTGTGTAACAACTCACC
AACCGAATGTACTAGCCCTGAAAATGGATGGCGCTCAAGCGTCT-
CACCCATACCTCGCCCTTAGGGTAGAAACGATGCCCTAAGG-AGTAGGCGGC----

>H_chionostomum_STMA_14060

---CAGGGATTGCCCTAGTAACGGCGAG--
TGAAGCGGCAACAGCTCAAA-TTTGAAATCTGGCCC---------
TCGCGGTCCGAATTGTAATTTGTAGAGGATGCTTTTGGTGCGGT-
GCCTTCCGAGTTCCCTGGAACGGGACGCCAGAGAGGGTGAGAGCCCCGTACGG
TTGGA-
CACCTACCCTATATATAGCTCCTTCGACGAGTCGAGTAGTTTGGGAATGCTGCT

CTAAATGGGAGGTAAATTTCTTCTAAAGCTAAATACCGGCCAGAGACC-
GATAGCGCACAAGTAGAGTGATCGAAAGATGAAAAGCACTTTGAAAAGAGGG
TTAAATAGCACGTGAAATTGTTGAAAGGGAAGCG-
TTTGCGACCAGACCTTTTCTAGGCGGATCATCCGGTG-TTC-
TCACCGGTGCACTTCGCCTGG--TTTAGGCCAGCATCGGTTTT---
CGTAGGGGGACAAAGGCCTGGGGAACGTATCTCC----CTC--GGGAGTGTT-
ATAGCCCCTAGCGTAATACCCCT-ACGGGGACCGAGGACCGCGC-TTC---
GGCAAGGATGCTGGCATAATGGTCGTCAACGACCCGTCTTGAAACACGGACCA
AGGAGTCGAACATTTGTGCGAGTGTTTGGGTG--
TTAAACCCTCACGCGTAATGAAAGTGAACGGAGGTGAGAGCCCCT-------
CGGGGTGCATCATCGACCGATCCTGAAG-
TCTTCGGATGGATTTGAGTAAGAGCATAACTGTTCGGACCCGAAAGATGGTGA
ACTATGCGTGGATAGGGTGAAGCCAGAGGAAACTCTGGTGGAGGCTCGCAGCG
GTTCTGACGTGCAAATCGATCGTCAAATCTGCGCATGGGGGCGAAA--------------

GACTTATCGAACCATC---

TAGTAGCTGGTTACCGCCGAAGTTTCCCTCAGGATAGCAGTGTTG--
TTTTCAGTTTTATGAGGTAAAGCGAATGATTAGGGACTCGGGGGCGCTATATTG
CCTTCATCCATTCTCAAACTTTAAATATGTAAGAAGCCCTTGTTGCTTAATTGA
ACGTGGGCATTCGAATGTATCAACACTAGTGGGCCATTTTTGGTAAGCAGAACT
GGCGATGCGGGATGAACCGAACGCGGGGTTAAGGTGCCAGAGTGGACGCTCAT
CAGACACCACAAAAGGTGTTAGTACATCTAGACAGT----------------------------------
----

TGGACGGTGGCCATGGAAGTCGGAATCCGCTAAGGACTGTGTAACAACTCACC
AACCGAATGTACTAGCCCTGAAAATGGATGGCGCTCAAGCGTCT-
CACCCATACCTCGCCCTTAGGGTAGAAACGATGCCCTAAGG-
AGTAGGCGGCCGTAG--GGG-TCAGTGACGAAGCCTG----------------------------------
----

>H_canariense_MUCL_47224

---ATTGCCTCAGTAACGGCGAG--
TGAAGCGGCAACAGCTCAAA-TTTGAAATCTGGCCC---------
TCGTGGTCCGAATTGTAATTTGTAGAGGATGCTTTGGGCGCGGT-
GCCTTCCGAGTTCCCTGGAACGGGACGCCAGAGAGGGTGAGAGCCCCGTACGG
TCGGATCACCTAGCCTATCTATAGCTCCTTCGACGAGTCGGGTAGTTTGGGAAT
GCTGCTCTAAATGGGAGGTAAATTTCTTCTAAAGCTAAATACCGGCCAGAGAC
C-
GATAGCGCACAAGTAGAGTGATCGAAAGATGAAAAGCACTTTGAAAAGAGGG
TTAAATAGCACGTGAAATTGTTGAAAGGGAAGCG-
TTTACGACCAGACCTCTTCCAGGCGGATCATCCGGTG-TTC-
TCACCGGTGCACTTCGCCTGG--TTCAGGCCAGCATCGGTTTT---
CGTAGGGGGAGAAAGGCCTGGGGAACGTATCTCC----TTA--GGGAGTGTT-
ATAGCCCCTTGCGTAATACCCTT-ACGGGGACCGAGGACCGCGC-TCC---
GGCAAGGATGCTGGCGTAATGGTCGTCAACGACCCGTCTTGAAACACGGACCA
AGGAGTCGAACATTTGTGCGAGTGTTTGGGTA--
GTAAACCCTCACGCGTAATGAAAGTGAACGGAGGTGAGAGCCCCT-------
CGGGGTGCATCATCGACCGATCCTGATG-
TCTTCGGATGGATTTGAGTAAGAGCATAACTGTTCGGACCCGAAAGATGGTGA
ACTATGCGTGGATAGGGTGAAGCCAGAGGAAACTCTGGTGGAGGCTCGCAGCG
GTTCTGACGTGCAAATCGATCGTCAAATCTGCGCATGGGGGCGAAA---------------
----
GACTTATCGAACCATC--

TAGTAGCTGGTTACCGCCGAAGTTTCCCTCAGGATAGCAGTGTTG--
TTTTCAGTTTTATGAGGTAAAGCGAATGATTAGGGACTCGGGGGCGCTATATTG
CCTTCATCCATTCTCAAACTTTAAATATGTAAGAAGCCCTTGTTACTTTATTGAA
CGTGGGCATTCGAATGTATCAACACTAGTGGGCCATTTTTGGTAAGCAGAACTG
GCGATGCGGGATGAACCGAACGTGGGGTTAAGGTGCCAGAGTGGACGCTCATC
AGACACCACAAAAGGTGTTAGTACATCTAGACAGT--------------------------------------
----

TGGACGGTGGCCATGGAAGTCGGAATCCGCTAAGGACTGTGTAACAACTCACC
AACCGAATGTACTAGCCCTGAAAATGGATGGCGCTCAAGCGTCT-
CACCCATACCTCACCCTTAGGGTAGAAACGATGCCCTAAGG-
AGTAGGCGGCCGTAG--GGG-TCAGTGACGAAGCCTAGGGC----------------------------
----

>H_munkii_MUCL_53315

---TAACGGCGAG--
TGAAGCGGCAACAGCTCAAA-TTTGAAATCTGGCCC---------
TCGCGGTCCGAGTTGTAATTTGTAGAGGATGCTTTTGGTGCGGT-
GCCTTCCGAGTTCCCTGGAACGGGACGCCGGAGAGGGTGAGAGCCCCGTACGG
TTGGA-
CACCTATCCTATATGTAGCTCCTTCGACGAGTCGAGTAGTTTGGGAATGCTGCT
CTAAATGGGAGGTAAATTTCTTCTAAAGCTAAATACCGGCCAGAGACC-
GATAGCGCACAAGTAGAGTGATCGAAAGATGAAAAGCACTTTGAAAAGAGGG
TTAAATAGCACGTGAAATTGCTGAAAGGGAAGCG-
TTTGCGACCAGACCTCCTCCGGGCGGATCATCCGGTG-TTT-
TCACCGGTGCACTTCGCCCGG--TCTAGGCCAGCATCGGTTTT---
CGTAGGGGGATAAAGGCCCGGGGCACGTAGCTCT----TCC--GGGAGTGTT-
ATAGCCCCCCGCGTAATGCCCTT-ACGGGGACCGAGGACCGCGC-TCT---
TGCAAGGATGCTGGCGTAATGGTCGTCAACGACCCGTCTTGAAACACGGACCA
AGGAGTCGAACATTTGTGCGAGTGTTTGGGTG--
TTAAACCCTCACGCGTAATGAAAGTGAACGGAGGTGAGAGCCCTT-------
CGGGGTGCATCATCGACCGATCCTGATG-
TCTTCGGATGGATTTGAGTAAGAGCATAACTGTTCGGACCCGAAAGATGGTGA
ACTATGCGTGGATAGGGTGAAGCCAGAGGAAACTCTGGTGGAGGCTCGCAGCG
GTTCTGACGTGCAAATCGATCGTCAAATCTGCGCATGGGGGCGAAA--------------

GACTTATCGAACCATC---

TAGTAGCTGGTTACCGCCGAAGTTTCCCTCAGGATAGCAGTGTTG--
TTTTCAGTTTTATGAGGTAAAGCGAATGATTAGGGACTCGGGGGCGCTTTTTAG
CCTTCATCCATTCTCAAACTTTAAATATGTAAGAAGCCCTTGTTACTTCGTTGAA
CGTGGGCATTCGAATGTATCAACACTAGTGGGCCATTTTTGGTAAGCAGAACTG
GCGATGCGGGATGAACCGAACGCGGGGTTAAGGTGCCAGAGTGGACGCTCATC
AGACACCACAAAAGGTGTTAGTACATCCAGACAGC------------------------------------
----

AGGACGGTGGCCATGGAAGTCGGAATCCGSTAAGGACTGTGTAACAACTCACC
TGCCGAATGTAYTAGCCCTGAAAATGGATGGCGCTCAAGCGTCT-

CACCCATACCTCGCCCTTAGGGTAGAAACGATGCCCTAAGG-
AGTAGGCGGCCGYGG--AGGTTTAGTGACGAAGCCTAGGGCGGAG-----------------
----

>H_samuelsii_MUCL_51843

---CCTAGTAACGGCGAG--
TGAAGCGGCAACAGCTCAAA-TTTGAAATCTGGCCC---------
TCGCGGTCCGAGTTGTAATTTGTAGAGGATGCTTTTGGTGCGGT-
GCCTTCCGAGTTCCCTGGAACGGGACGCCGGAGAGGGTGAGAGCCCCGTACGG
TTGGA-
CACCTATCCTCTATATAGCTCCTTCAACGAGTCGAGTAGTTTGGGAATGCTGCT
CTAAATGGGAGGTAAATTTCTTCTAAAGCTAAATACCGGCCAGAGACC-
GATAGCGCACAAGTAGAGTGATCGAAAGATGAAAAGCACTTTGAAAAGAGGG
TTAAATAGCACGTGAAATTGTTGAAAGGGAAGCG-
TTTGCGACCAGACCTTCTCCAGGGGGATCATCCGGCG-TTC-
TCGCCGGTGCACTTCGCCTGG--TTTAGGCCAGCATCGGTTTT---
CGTAGGGGGAAAAAGTCTTGGGGCACGTAGCTCT----TCC--GGGAGTGTT-
ATAGCCCCTCGTGTAATACCCTT-CCGTGGACCGAGGACCGCGC-
CTTCTCGGCAAGGATGCTGGCGTAATGGTCGTCAACGACCCGTCTTGAAACAC
GGACCAAGGAGTCGAACATTTGTGCGAGTGTTTGGGTG--
TTAAACCCTCACGCGTAATGAAAGTGAACGGAGGTGAGAGCCTTT-------
CGGGGCGCATCATCGACCGATCCTGATG-
TCTTCGGATGGATTTGAGTAAGAGCATAACTGTTCGGACCCGAAAGATGGTGA
ACTATGCGTGGATAGGGTGAAGCCAGAGGAAACTCTGGTGGAGGCTCGCAGCG
GTTCTGACGTGCAAATCGATCGTCAAATCTGCGCATGGGGGCGAAA---------------
----
GACTTATCGAACCATC---

TAGTAGCTGGTTACCGCCGAAGTTTCCCTCAGGATAGCAGTGTTG--
TTTTCAGTTTTATGAGGTAAAGCGAATGATTAGGGACTCGGGGGCGCTTTTTTG
CCTTCATCCATTCTCAAACTTTAAATATGTAAGAAGCCCTTGTTACTTAGCTGA
ACGTGGGCATTCGAATGTATCAACACTAGTGGGCCATTTTTGGTAAGCAGAACT
GGCGATGCGGGATGAACCGAACGCGGGGTTAAGGTGCCAGAGTGGACGCTCAT

CAGACACCACAAAAGGTGTTAGTACATCCAGACAGC-----------------------------------
----

AGGACGGTGGCCATGGAAGTCGGAATCCGCTAAGGACTGTGTAACAACTCACC
TGCCGAATGTACTAGCCCTGAAAATGGATGGCGCTCAAGCGTCT-
CACCCATACCTCGCC---

>H_erythrostroma_MUCL_53759

---TGCCCTAGTAACGGCGAG--
TGAAGCGGCAACAGCTCAAA-TTTGAAATCTGGCCC---------
TAGCGGTCCGAGTTGTAATTTGTAGAGGATGCTTTTGGCGCGGT-
GCCTTCTGAGTTCCCTGGAACGGGACGCCAGAGAGGGTGAGAGCCCCGTACGG
TTGGC-
CGCCTAGCCTTTATACAGCTCCTTCGACGAGTCGAGTAGTTTGGGAATGCTGCT
CTAAATGGGAGGTAAATTTCTTCTAAAGCTAAATACCGGCCAGAGACC-
GATAGCGCACAAGTAGAGTGATCGAAAGATGAAAAGTACTTTGAAAAGAGGG
TTAAATAGCACGTGAAATTGTTGAAAGGGAAGCG-
TTTGCGACCAGACCTTCTCCTGGCGGATCATCCGGTG-TTC-
TCACCGGTGCACTTCGCCTGG--TTTAGGCCAGCATCGGTTCT---
CTTAGGGGGATAAAGGTCTTGGGCACGTAGCTCT----TTC--GGGAGTGTT-
ATAGCCCCTGGCGTAATACCCTT-CGGGGGACCGAGGACCGCGC-TCT----
GCAAGGATGCTGGCGTAATGGTCGTCAACGACCCGTCTTGAAACACGGACCAA
GGAGTCGAACATTTGTGCGAGTGTTTGGGTG--
TTAAACCCTCACGCGTAATGAAAGTGAACGGAGGTGAGAGCCCCT-------
AGGGGTGCATCATCGACCGATCCTGATG-
TCTTCGGATGGATTTGAGTAAGAGCATAACTGTTCGGACCCGAAAGATGGTGA
ACTATGCGTGGATAGGGTGAAGCCAGAGGAAACTCTGGTGGAGGCTCGCAGCG
GTTCTGACGTGCAAATCGATCGTCAAATCTGCGCATGGGGGCGAAA--------------

GACTTATCGAACCATC---

TAGTAGCTGGTTACCGCCGAAGTTTCCCTCAGGATAGCAGTGTTG--
TTTTCAGTTTTATGAGGTAAAGCGAATGATTAGGGACTCGGGGGCGCTATATTG
CCTTCATCCATTCTCAAACTTTAAATATGTAAGAAGCCCTTGTTGCTTAGTTGA
ACGTGGGCATTCGAATGTATCAACACTAGTGGGCCATTTTTGGTAAGCAGAACT
GGCGATGCGGGATGAACCGAACGCGGGGTTAAGGTGCCAGAGTAAACGCTCAT
CAGACACCACAAAAGGTGTTAATACATCCTGACAGC-----------------------------------
----

AGGACGGTGGCCATGGAAGTCGGAATCCGCTAAGGACTGTGTAACAACTCACC
TGCCGAATGTATTAGCCCTGAAAATGGATGGCGCTCAAGCGTTT-
CACCAATACCTCGCCCTTAGGGTAGAAACGATGCCCTAAGG-
AGTAGGCGGCCGTGG--AGG-TTAGTGACGAAGCC---------------------------------------
----

>H_barbarensis_STMA_14081

---TGCCCTAGTAACGGCGAG--
TGAAGCGGCAACAGCTCAAA-TTTGAAATCTGGCCT---------TCG-
GGTCCGAGTTGTAATTTGCAGAGGATGCTTTGGGTGCGGT-
GCCTTCCGAGTTCCCTGGAACGGGACGCCTTAGAGGGTGAGAGCCCCGTACGG
TTGGA-
CACCTAGCCTATGTATAGCTCCTTCGACGAGTCGAGTAGTTTGGGAATGCTGCT
CTAAATGGGAGGTAAATTTCTTCTAAAGCTAAATACCGGCCAGAGACC-
GATAGCGCACAAGTAGAGTGATCGAAAGATGAAAAGCACTTTGAAAAGAGGG
TTAAATAGCACGTGAAATTGTTGAAAGGGAAGCG-
TTTGCGACCAGACCTTTTCCCGGCGGATCATCCGGTG-TTC-
TCACCGGTGCACTTCGCCGGG--TTTAGGCCAGCATCGGTTCT---
CTTCGGGGGATAAAGGCTCGGGGCACGTAGCTCT----TTC--GGGAGTGTT-
ATAGCCCCTTGCGTAATACCCTG-CGGGGGACCGAGGACCGCGC-ATT----
GCAAGGATGCTGGCGTAATGGTCGTCAACGACCCGTCTTGAAACACGGACCAA
GGAGTCGAACATTTGTGCGAGTGTTTGGGTG--
TCAAACCCTCACGCGTAATGAAAGTGAACGGAGGTGAGAGCCCTT-------
CGGGGCGCATCATCGACCGATCCTGATG-
TCTTCGGATGGATTTGAGTAAGAGCATAACTGTTCGGACCCGAAAGATGGTGA
ACTATGCGTGGATAGGGTGAAGCCAGAGGAAACTCTGGTGGAGGCTCGCAGCG

GTTCTGACGTGCAAATCGATCGTCAAATCTGCGCATGGGGGCGAAA--------------

GACTTATCGAACCATC---

TAGTAGCTGGTTACCGCCGAAGTTTCCCTCAGGATAGCAGTGTTG--
TCTTCAGTTTTATGAGGTAAAGCGAATGATTAGGGACTCGGGGGCGCTATATTG
CCTTCATCCATTCTCAAACTTTAAATATGTAAGAAGCCCTTGTTACTTAATTGA
ACGTGGGCATTCGAATGTACCAACACTAGTGGGCCATTTTTGGTAAGCAGAAC
TGGCGATGCGGGATGAACCGAACGTGGGGTTAAGGTGCCAGAGTGGACGCTCA
TCAGACACCACAAAAGGTGTTAGTACATCTTGACAGC--------------------------------
----

AGGACGGTGGCCATGGAAGTCGGAATCCGCTAAGGACTGTGTAACAACTCACC
TGCCGAATGTACTAGCCCTGAAAATGGATGGCGCTCAAGCGTCT-
CACCCATACCTCACCNNNNGGGTAGAAACGATGCCCTAAGG-
AGTAGGCGGCCGNGG--AGG-TTAGTGACGAAG---

>H_submonticulosa_CBS_115280

--ATTGCCCTAGTAACGGCGAG--
TGAAGCGGCAACAGCTCAAA-TTTGAAATCTGGCCT---------TCG-
GGTCCGAGTTGTAATTTGTAGAGGATGCTTTGGGTGCGGT-
GCCTTCCGAGTTCCCTGGAACGGGACGCCTTAGAGGGTGAGAGCCCCGTACGG
TTGGA-
CACCTAGCCTATGTATAGCTCCTTCGACGAGTCGAGTAGTTTGGGAATGCTGCT
CTAAATGGGAGGTAAATTTCTTCTAAAGCTAAATACCGGCCAGAGACC-
GATAGCGCACAAGTAGAGTGATCGAAAGATGAAAAGCACTTTGAAAAGAGGG
TTAAATAGCACGTGAAATTGTTGAAAGGGAAGCG-
TTTGCGACCAGACCTTTTCCAGGCGGATCATCCGGTG-TTC-
TCACCGGTGCACTTCGTCTGG--TCGAGGCCAGCATCGGTTCT---
CCGCAGGGGATAAAGGCTCGGGGCATGTAGCTCT----TTC--GGGAGTGTT-

ATAGCCCCTTGCGTAATATCCTT-CGGGGGACCGAGGACCGCGC-ATT----
GCAAGGATGCTGGCGTAATGGTCGTCAACGACCCGTCTTGAAACACGGACCAA
GGAGTCGAACATTTGTGCGAGTGTTTGGGTG--
TCAAACCCTCACGCGTAATGAAAGTGAACGGAGGTGAGAGCCCCT-------
CGGGGTGCATCATCGACCGATCCTGATG-
TCTTCGGATGGATTTGAGTAAGAGCATAACTGTTCGGACCCGAAAGATGGTGA
ACTATGCGTGGATAGGGTGAAGCCAGAGGAAACTCTGGTGGAGGCTCGCAGCG
GTTCTGACGTGCAAATCGATCGTCAAATCTGCGCATGGGGGCGAAA--------------

GACTTATCGAACCATC--

TAGTAGCTGGTTACCGCCGAAGTTTCCCTCAGGATAGCAGTGTTG--
TCTTCAGTTTTATGAGGTAAAGCGAATGATTAGGGACTCGGGGGCGCTATATTG
CCTTCATCCATTCTCAAACTTTAAATATGTAAGAAGCCCTTGTTACTTAATTGA
ACGTGGGCATTCGAATGTACCAACACTAGTGGGCCATTTTTGGTAAGCAGAAC
TGGCGATGCGGGATGAACCGAACGTGGGGTTAAGGTGCCAGAGTGGACGCTCA
TCAGACACCACAAAAGGTGTTAGTACATCTTGACAGC-------------------------------
----

AGGACGGTGGCCATGGAAGTCGGAATCCGCTAAGGACTGTGTAACAACTCACC
TGCCGAATGTACTAGCCCTGAAAATGGATGGCGCTCAAGCGTCT-
CACCCATACCCCACCCTTAAGGTAGAAACGATGCCCTAAGG-
AGTAGGCGGCCGTGG--AGG-TTAGTGACGAAGCCTAGG------------------------------
----

>H_monticulosa_MUCL_54604

--CCTAGTAACGGCGAG--
TGAAGCGGCAACAGCTCAAA-TTTGAAATCTGGCCT---------TCG-
GGTCCGAATTGTAATTTGTAGAGGATGCTTTGGGTGCGGT-
ACCTTCCGAGTTCCCTGGAACGGGACGCCGGAGAGGGTGAGAGCCCCGTACGG
TTGGA-

TACCAAGCCTATGTATAGCTCCTTCGACGAGTCGAGTAGTTTGGGAATGCTGCT
CTAAATGGGAGGTAAATTTCTTCTAAAGCTAAATACCGGCCAGAGACC-
GATAGCGCACAAGTAGAGTGATCGAAAGATGAAAAGCACTTTGAAAAGAGGG
TTAAATAGCACGTGAAATTGTTGAAAGGGAAGCG-
TTTGCGACCAGACCTTTTCCAGGCGGATCATCCGGTG-TTC-
TCACCGGTGCACTTCGCCTGG--TTTAGGCCAGCATCGGTTTT---
CTTAGGGGGATAAAGGTTTAGGGCACGTAGCTCC----TTC--GGGAGTGTT-
ATAGCCCTCTACGTAATACCCTT-CGGGGGACCGAGGACCGCGC-ATT----
GCAAGGATGCTGGCGTAATGGTCGTCAACGACCCGTCTTGAAACACGGACCAA
GGAGTCGAACATTTGTGCGAGTGTTTGGGTG--
TTAAACCCTCACGCGTAATGAAAGTGAACGGAGGTGAGAGCCCTT-------
CGGGGTGCATCATCGACCGATCCTGATG-
TCTTCGGATGGATTTGAGTAAGAGCATAACTGTTCGGACCCGAAAGATGGTGA
ACTATGCGTGGATAGGGTGAAGCCAGAGGAAACTCTGGTGGAGGCTCGCAGCG
GTTCTGACGTGCAAATCGATCGTCAAATCTGCGCATGGGGGCGAAA--------------

GACTTATCGAACCATC--

TAGTAGCTGGTTACCGCCGAAGTTTCCCTCAGGATAGCAGTGTTG--
TCTTCAGTTTTATGAGGTAAAGCGAATGATTAGGGACTCGGGGGCGCTATATTG
CCTTCATCCATTCTCAAACTTTAAATATGTAAGAAGCCCTTGTTACTTAGTTGA
ACGTGGGCATTCGAATGTACCAACACTAGTGGGCCATTTTTGGTAAGCAGAAC
TGGCGATGCGGGATGAACCGAACGTGGGGTTAAGGTGCCAGAGTGGACGCTCA
TCAGACACCACAAAAGGTGTTAGTACATCTTGACAGC----------------------------------
----

AGGACGGTGGCCATGGAAGTCGGAATCCGCTAAGGACTGTGTAACAACTCACC
TNCCGAATGTACTAGCCCTGAAAATGGATGGCGCTCAAGCGTCT-
CACCCATACCTCACCCTTAGGGTAGAAACGATGCCCTAAGG-AGTAGGCG--------

>H_lenormandii_CBS_119003

--ATTGCCCTAGTAACGGCGAG--
TGAAGCGGCAACAGCTCAAA-TTTGAAATCTGGCCC---------
TAGCGGTCCGAGTTGTAATTTGTAGAGGATGCTTTTGGTGCGGC-
GCCTTCCGAGTTCCCTGGAACGGGACGCCGGAGAGGGTGAGAGCCCCGTACGG
TTGGA-
CGCCTAGCCTCTATATAGCTCCTTCGACGAGTCGAGTAGTTTGGGAATGCTGCT
CTAAATGGGAGGTAAATTTCTTCTAAAGCTAAATACCGGCCAGAGACC-
GATAGCGCACAAGTAGAGTGATCGAAAGATGAAAAGCACTTTGAAAAGAGGG
TTAAATAGCACGTGAAATTGTTGAAAGGGAAGCG-
TTTGCGACCAGACTTTTTCCGGGCGGATCATCCGGTG-TTT-
AGGCCGGTGCACTCCGTCCGG--TTTAGGCCAGCATCGGTTCT---
CTTAGGGGGATAAAGGCTTAGGGCACGTAGCTCT----TTC--GGGAGTGTT-
ATAGCCCTTCGCGTAATACCCTT-CGGGGGACCGAGGTACGCGC-TCT----
GCAAGGATGCTGGCGTAATGGTCGTCAACGACCCGTCTTGAAACACGGACCAA
GGAGTCGAACATTTGTGCGAGTGTTTGGGTG--
TTAAACCCTCACGCGTAATGAAAGTGAACGGAGGTGAGAGCCCTT-------
CGGGGTGCATCATCGACCGATCCTGATG-
TCTTCGGATGGATTTGAGTAAGAGCATAACTGTTCGGACCCGAAAGATGGTGA
ACTATGCGTGGATAGGGTGAAGCCAGAGGAAACTCTGGTGGAGGCTCGCAGCG
GTTCTGACGTGCAAATCGATCGTCAAATCTGCGCATGGGGGCGAAA---------------
----
GACTTATCGAACCATC--

TAGTAGCTGGTTACCGCCGAAGTTTCCCTCAGGATAGCAGTGTTG--
TCTTCAGTTTTATGAGGTAAAGCGAATGATTAGGGACTCGGGGGCGCTATTTTG
CCTTCATCCATTCTCAAACTTTAAATATGTAAGAAGCCCTTGTTACTTAGTTGA
ACGTGGGCATTCGAATGTACCAACACTAGTGGGCCATTTTTGGTAAGCAGAAC
TGGCGATGCGGGATGAACCGAACGCGGGGTTAAGGTGCCAGAGTGGACGCTCA
TCAGACCCCACAAAAGGTGTTAATACATCCTGACAGC----------------------------------
----

AGGACGGTGGCCATGGAAGTCGGAATCCGCTAAGGACTGTGTAACAACTCCCC
TGCCGAATGTATTAGCCCTGAAAATGGATGGCGCTCTAGCGTCT-
CACCCATACCTCGCCCTTAGGGTAGAAACCATGCCCTAAGG-
AGTAGGCGGCCCTGG--AGG--

>H_guilanense_MUCL_57726

--GCCCTAGTAACGGCGAG--
TGAAGCGGCAACAGCTCAAA-TTTGAAATCTGGCCC---------
TCGTGGTCCGAATTGTAATTTGTAGAGGATGCTTTGGGTGCGGT-
GCCTTCCGAGTTCCCTGGAACGGGACGCCAGAGAGGGTGAGAGCCCCGTACGG
TTGGC-
CACCTAGCCTCTATATAGCTCCTTCGACGAGTCGGGTAGTTTGGGAATGCTGCT
CTAAATGGGAGGTAAATTTCTTCTAAAGCTAAATACCGGCCAGAGACC-
GATAGCGCACAAGTAGAGTGATCGAAAGATGAAAAGCACTTTGAAAAGAGGG
TTAAATAGCACGTGAAATTGTTGAAAGGGAAGCG-
TTTACGACCAGACCTTCTCCAGGCGGATCATCCGGTG-TTC-
TCACCGGTGCACTTCGTCTGG--TGTAGGCCAGCATCGGTTTT---
CGTAAGGGGATAAAGGCCTGGGGAACGTATCTCC----TTC--GGGAGTGTT-
ATAGCCCCTCGCGTAATACCCTT-ACGGGGACCGAGGACCGCGC-TCT--
ACGCAAGGATGCTGGCGTAATGGTCGTCAACGACCCGTCTTGAAACACGGACC
AAGGAGTCGAACATTTGTGCGAGTGTTTGGGTG--
GTAAACCCTCACGCGTAATGAAAGTGAACGGAGGTGAGAGCCCCTC------
CGGGGTGCATCATCGACCGATCCTGATG-
TCTTCGGATGGATTTGAGTAAGAGCATAACTGTTCGGACCCGAAAGATGGTGA
ACTATGCGTGGATAGGGTGAAGCCAGAGGAAACTCTGGTGGAGGCTCGCAGCG
GTTCTGACGTGCAAATCGATCGTCAAATCTGCGCATGGGGGCGAAA-----------------
----
GACTTATCGAACCATC--

TAGTAGCTGGTTACCGCCGAAGTTTCCCTCAGGATAGCAGTGTTG--
TTTTCAGTTTTATGAGGTAAAGCGAATGATTAGGGACTCGGGGGCGCTTTATTG
CCTTCATCCATTCTCAAACTTTAAATATGTAAGAAGCCCTTGTTACTTAACTGA
ACGTGGGCATTCGAATGTATCAACACTAGTGGGCCATTTTTGGTAAGCAGAACT
GGCGATGCGGGATGAACCGAACGTGGGGTTAAGGTGCCAGAGTGGACGCTCAT
CAGACACCACAAAAGGTGTTAGTACATCTAGACAGT---

TGGACGGTGGCCATGGAAGTCGGAATCCGCTAAGGACTGTGTAACAACTCACC
AACCGAATGTACTAGCCCTGAAAATGGATGGCGCTCAAGCGTCT-

CACCCATACCTCACCCTTAGGGTAGAAACGATGCCCTAAGG-AGT-----------------
----

>H_musceum_MUCL_53765

TTGAAATCTGGCCC---------TCG-
GGTCCGAATTGTAATTTGTAGAGGATGCTTTTGGTGCGGT-
GCCTTCCGAGTTCCCTGGAACGGGACGCCATAGAGGGTGAGAGCCCCGTACGG
TTGGA-
AGCCAAGCCTATATATAGCTCCTTCGACGAGTCGAGTAGTTTGGGAATGCTGCT
CTAAATGGGAGGTAAATTTCTTCTAAAGCTAAATACCGGCCAGAGACC-
GATAGCGCACAAGTAGAGTGATCGAAAGATGAAAAGCACTTTGAAAAGAGGG
TTAAATAGCACGTGAAATTGTTGAAAGGGAAGCG-
TTTATGACCAGACCTCTTCCAGGCGGATCATCCGGTG-TTC-
TCACCGGTGCACTTCGTCTGG--TTTAGGCCAGCATCGGTTTC---
CTTAGGGGGATAAAAGCCTGGGGAATGTGGCTCT----TTC--GAGAGTGTT-
ATAGCCCCTAGCATAATACCCTT-CAGGGGACCGAGGACCGCGC-TTC---
GGCATGGATGCTGGCGTAATGGTTATCAACGACCCGTCTTGAAACACGGACCA
AGGAGTCGAACATTTGTGCGAGTGTTTGGGTG--
TTAAACCCTCACGCGTAATGAAAGTGAACGGAGGTGAGAGCCCCT-------
CGGGGTGCATCATCGACCGATCCTGATG-
TCTTCGGATGGATTTGAGTAAGAGCATAACTGTTCGGACCCGAAAGATGGTGA
ACTATGCGTGGATAGGGTGAAGCCAGAGGAAACTCTGGTGGAGGCTCGCAGCG
GTTCTGACGTGCAAATCGATCGTCAAATCTGCGCATGGGGGCGAAA-------------

GACTTATCGAACCATC--

TAGTAGCTGGTTACCGCCGAAGTTTCCCTCAGGATAGCAGTGTTG--
TATTCAGTTTTATGAGGTAAAGCGAATGATTAGGGACTCGGGGGCGCTATATTG
CCTTCATCCATTCTCAAACTTTAAATATGTAAGAAGCCCTTGTTACTTCATTGAA
CGTGGGCATTCGAATGTATCAACACTAGTGGGCCATTTTTGGTAAGCAGAACTG
GCGATGCGGGATGAACCGAACGCGAGGTTAAGGTGCCAGAGTGGACGCTCATC

AGACACCACAAAAGGTGTTAGTACATCTAGACAGT--

TGGACGGTGGCCATGGAAGTCGGAATCCGCTAAGGACTGTGTAACAACTCACC
AACCGAATGTACTAGCCCTGAAAATGGATGGCGCTCAAGCGTCT-
CACCCATACCTCGCCCTTAGGGTAGAAACGATGCCTTAAGG-
AGTAGGCGGCCGTAG--GGG-TCAG---

>H_isabellinum_MUCL_53308

--GGGATTGCCCTAGTAACGGCGAG--
TGAAGCGGCAACAGCTCAAA-TTTGAAATCTGGCCC---------TCG-
GGTCCGAATTGTAATTTGTAGAGGATGCTTTTGGTGCGGT-
GCCTTCTGAGTTCCCTGGAACGGGACGCCAGAGAGGGTGAGAGCCCCGTACGG
TTGGC-
CACCTAGCCTCTATATAGCTCCTTCGACGAGTCGAGTAGTTTGGGAATGCTGCT
CTAAATGGGAGGTAAATTTCTTCTAAAGCTAAATACCGGCCAGAGACC-
GATAGCGCACAAGTAGAGTGATCGAAAGATGAAAAGCACTTTGAAAAGAGGG
TTAAATAGCACGTGAAATTGTTGAAAGGGAAGCG-
TTTGTGACCAGACTTTCTCCAGGCGGATCATCCGGTG-TTC-
TCACCGGTGCACTTCGTCTGG--TGTAGGCCAGCATCGGTTTC---
CGTAGGGGGATAAAAGCTCAGGGAATGTGGCTCC----TCC--GGGAGTGTT-
ATAGCCCTCTGTATAATACCCTT-CCGGGGACCGAGGACCGCGC-TTC---
GGCAAGGATGCTGGCGTAATGGTTATCAACGACCCGTCTTGAAACACGGACCA
AGGAGTCGAACATTTGTGCGAGTGTTTGGGTG--
TTAAACCCTCACGCGTAATGAAAGTGAACGGAGGTGAGAGCCCTT-------
CGGGGTGCATCATCGACCGATCCTGATG-
TCTTCGGATGGATTTGAGTAAGAGCATAACTGTTCGGACCCGAAAGATGGTGA
ACTATGCGTGGATAGGGTGAAGCCAGAGGAAACTCTGGTGGAGGCTCGCAGCG
GTTCTGACGTGCAAATCGATCGTCAAATCTGCGCATGGGGGCGAAA----------------
----
GACTTATCGAACCATC--

TAGTAGCTGGTTACCGCCGAAGTTTCCCTCAGGATAGCAGTGTTG--
TTTTCAGTTTTATGAGGTAAAGCGAATGATTAGGGACTCGGGGGCGCTTTATTG
CCTTCATCCATTCTCAAACTTTAAATATGTAAGAAGCCTTTGTTACTTTATTGAA
CGTAGGCATTCGAATGTATCAACACTAGTGGGCCATTTTTGGTAAGCAGAACTG
GCGATGCGGGATGAACCGAACGCGGGGTTAAGGTGCCAGAGTGGACGCTCATC
AGACACCACAAAAGGTGTTAGTACATCTAGACAGC----------------------------------
----

CGGACGGTGGCCATGGAAGTCGGAATCCGCTAAGGACTGTGTAACAACTCACC
GGCCGAATGTACTAGCCCTGAAAATGGATGGCGCTCAAGCGTCT-
CACCCATACCCCGCCCTTAGGGTAGAAACGATGCCCTAAGG-
AGTAGGCGGCCGTGG--AGG-TTAGTGACGAAGCCTAGGGCG-----------------------
----

>H_fendleri_MUCL_54792

---CTCAAA-
TTTGAAATCTGGCCC---------
TAGCGGTCCGAGTTGTAATTTGTAGAGGATGCTTTTGGTGAGGT-
GCCTTCTGAGTTCCCTGGAACGGGACGCCAGAGAGGGTGAGAGCCCCGTACGG
TCGGC-
CACCGAACCTATATATAGCTCCTTCGACGAGTCGAGTAGTTTGGGAATGCTGCT
CTAAATGGGAGGTAAATTTCTTCTAAAGCTAAATACCGGCCAGAGACC-
GATAGCGCACAAGTAGAGTGATCGAAAGATGAAAAGCACTTTGAAAAGAGGG
TTAAATAGCACGTGAAATTGTTGAAAGGGAAGCG-
TTTGCGACCAGACTTTTTCCTGTCGGATCATCCGGTG-
TTCTTCACCGGTGCACTTCGGCAGG--TTTAGGCCAGCATCGGTTCT---
CTTAGGGGGATAAAGGCCTTGGGAACGTAGCTCT----TTA--GGGAGTGTT-
ATAGCCCCTGGCGCAATACCCTT-CGGGGGACCGAGGATCGCGC-TCT----
GCAAGGATGCTGGCGTAATGGTCGTCAACGACCCGTCTTGAAACACGGACCAA
GGAGTCGAACATTTGTGCGAGTGTTTGGGTG--
TCAAACCCTCACGCGTAATGAAAGTGAACGGAGGTGAGAGCCTT---------
AGGGCGCATCATCGACCGATCCTGATG-
TCTTCGGATGGATTTGAGTAAGAGCATAACTGTTCGGACCCGAAAGATGGTGA

ACTATGCGTGGATAGGGTGAAGCCAGAGGAAACTCTGGTGGAGGCTCGCAGCG
GTTCTGACGTGCAAATCGATCGTCAAATCTGCGCATGGGGGCGAAA--------------

GACTTATCGAACCATC---

TAGTAGCTGGTTACCGCCGAAGTTTCCCTCAGGATAGCAGTGTTG--
TCTTCAGTTTTATGAGGTAAAGCGAATGATTAGGGACTCGGGGGCGCTATATTG
CCTTCATCCATTCTCAAACTTTAAATATGTAAGAAGCCCTTGTTACTTAGTTGA
ACGTGGGCATTCGAATGTACCAACACTAGTGGGCCATTTTTGGTAAGCAGAAC
TGGCGATGCGGGATGAACCGAACGCGGGGTTAAGGTGCCAGAGTGGACGCTCA
TCAGACACCACAAAAGGTGTTAGTACATCTTGACAGC---------------------------------
----

AGGACGGTGGCCATGGAAGTCGGAATCCGCTAAGGACTGTGTAACAACTCACC
TGCCGAATGTACTAGCCCTGAAAATGGATGGCGCTCAAGCGTCT-
CACCCATACCTCGCC---

>H_olivaceopigmentum_DSM_107924

-------------CGAGTTGTAATTTGTAGAGGATGCTTTTGGTGCGGC-
GCCTTCCGAGTTCCCTGGAACGGGACGCCGGAGAGGGTGAGAGCCCCGTACGG
TTGGA-
CGCCTAGCCTATATATAGCTCCTTCGACGAGTCGAGTAGTTTGGGAATGCTGCT
CTAAATGGGAGGTAAATTTCTTCTAAAGCTAAATACCGGCCAGAGACC-
GATAGCGCACAAGTAGAGTGATCGAAAGATGAAAAGCACTTTGAAAAGAGGG
TTAAATAGCACGTGAAATTGTTGAAAGGGAAGCG-
TTTGCGACCAGACCTTTTCCAGGCGGATCATCCGGTA-TTC-
TTACCGGTGCACTTCGCCTGG--TCTAGGCCAGCATCGGTTTC---
CTTAGGGGGATAAAGGCCGGGGGAACGTAGCTCT----TCT---GGAGTGTT-
ATAGCCCCCGGTGTAATACCCCT-CGGGGGACCGAGGACCGCGC-ATC---

TGCAAGGATGCTGGCGTAATGGTCGTCAACGACCCGTCTTGAAACACGGACCA
AGGAGTCGAACATTTGTGCGAGTGTTTGGGTG--
TTAAACCCTCACGCGTAATGAAAGTGAACGGAGGTGAGAGCCCTT-------
ACGGGCGCATCATCGACCGATCCTGATG-
TCTTCGGATGGATTTGAGTAAGAGCATAACTGTTCGGACCCGAAAGATGGTGA
ACTATGCGTGGATAGGGTGAAGCCAGAGGAAACTCTGGTGGAGGCTCGCAGCG
GTTCTGACGTGCAAATCGATCGTCAAATCTGCGCATGGGGGCGAAA---------------
----
GACTAATCGAACCATC---

TAGTAGCTGGTTACCGCCGAAGTTTCCCTCAGGATAGCAGTGTTG--
TCTTCAGTTTTATGAGGTAAAGCGAATGATTAGGGACTC--------------------------------
----

>H_aveirense_MUM_19_40

--ACAGGGATTGCCCTAGTAACGGCGAG--
TGAAGCGGCAACAGCTCAAA-TTTGAAATCTGGCCC---------
TCGTGGTCCGAGTTGTAATTTGTAGAGGATGCTTTGGGTGCGGT-
GCCTTCCGAGTTCCCTGGAACGGGACGCCAGAGAGGGTGAGAGCCCCGTACGG
TTGGA-
CACCTAGCCTATCTATAGCTCCTTCGACGAGTCGAGTAGTTTGGGAATGCTGCT
CTAAATGGGAGGTATATTTCTTCTAAAGCTAAATACCGGCCAGAGACC-
GATAGCGCACAAGTAGAGTGATCGAAAGATGAAAAGCACTTTGAAAAGAGGG
TTAAATAGCACGTGAAATTGTTGAAAGGGAAGCG-
TTTGCGACCAGACCTTCTCCGGGCGGATCATCCGGCG-TTC-

TCGCCGGTGCACTTCGCCCGG--TTTAGGCCAGCATCGGTTCT---
CTTAGGGGGATAAAGGCCAGGGGAACGTAGCTCT----TTC--GGGAGTGTT-
ATAGCCCCTGGCGCAATACCCCT-CGGGGGACCGAGGACCGCGC-TTT---
TGCAAGGATGCTGGCGTAATGGTCGTCAACGACCCGTCTTGAAACACGGACCA
AGGAGTCGAACATTGGTGCGAGTGTTTGGGTG--
TCAAACCCTCACGCGTAATGAAAGTGAACGGAGGTGAGAGCCCGT-------
ACGGGTGCATCATCGACCGATCCTGATG-
TCTTCGGATGGATTTGAGTAAGAGCATCACTGTTCGGACCCGAAAGATGGTGA
ACTATGCGTGGATAGGGTGAAGCCAGAGGAAACTCTGGTGGAGGCTCGCAGCG
GTTCTGACGTGCAAATCGATCGTCAAATCTGCGCATGGGGGCGAA---------------

GACTTATCGAACCATC--

TAGTAGCTGGTTACCGCCGAAGTTTCCCTCAGGATAGCAGTGTTG--
TCTTCAGTTTTATGAGGTAAAGCGAATGATTAGGGACTCGGGGGCGCTATATTG
CCTTCATCCATTCTCAAACTTTAAATATGTAAGAAGCCCTTGTTACTTAGTTGA
ACGTGGGCATTCGAATGTACCAACACTAGTGGGCCATTTTTGGTAAGCAGAAC
TGGCGATGCGGGATGAACCGAACGCGGGGTTAAGGTGCCAGAGTGGACGCTCA
TCAGACACCACAAAAGGTGTTAGTACATCTTGACAGCAGGTATGTTTTAACCTT
CTCTTCACTAT---

TTGCTTTTTACCCCCCCCTCTCCCCCTGCTGGAACAGGTGGCCTTAGTGAAGCA
ATGTGGCATCAGAGGTTCTGATGCTATTGTTACTTCACTGGCCACCTGCTGCCG
GCGTGTTCATGTTATAAGCTTAAG-----------CCATACCTGGCCC-----------
GAGCAGCTA-TATATATGGAATCAATCTGTTACTYG------------------------------------
TTTTTTTGAGATATAATACTAACTGTAATTTAATAGGACGGTGGCCATGGAAGT
CGGAATCCGCTAAGGACTGTGTAACAACTCACCTGCCGAATGTACTAGCCCTG
AAAATGGATGGCGCTCAAGCGTCT-
CACCCATACCTCGCCCTCAGGGTAGAAACGATGCCCTGAGG-
AGTAGGCGGCCGTGG--AGG-TTAGTGACGAAGCCTAGGGCGGAGC----------------
----

>H_cercidicola_CBS_119009

---ATTGCCCTAGTAACGGCGAG--
TGAAGCGGCAACAGCTCAAA-TTTGAAATCTGGCCC---------
TCGTGGTCCGAGTTGTAATTTGTAGAGGATGCTTTTGGTGCGGT-
GCCTTCCGAGTTCCCTGGAACGGGACGCCAGAGAGGGTGAGAGCCCCGTACGG
TTGGA-
CACCTACCCTATATATAGCTCCTTCGACGAGTCGAGTAGTTTGGGAATGCTGCT
CTAAATGGGAGGTAAATTTCTTCTAAAGCTAAATACCGGCCAGAGACC-
GATAGCGCACAAGTAGAGTGATCGAAAGATGAAAAGCACTTTGAAAAGAGGG
TTAAATAGCACGTGAAATTGTTGAAAGGGAAGCG-
TTTGCGACCAGACCTTTTCTAGGCGGATCATCCGGTG-TTC-
TCACCGGTGCACTTCGCCTAG--TCTAGGCCAGCATCGGTTTT---
CGTAGGAGGATAAAGGCCTGGGGAACGTATCTCT----TTC--GGGAGTGTT-
ATAGCCCCTCGTGTAATACTCTT-ACGGGGACCGAGGACCGCGC-TTC---
GGCAAGGATGCTGGCGTAATGGTCGTCAACGACCCGTCTTGAAACACGGACCA
AGGAGTCGAACATTTGTGCGAGTGTTTGGGTA--
TTAAACCCTCACGCGTAATGAAAGTGAACGGAGGTGAGAGCCCTT-------
CGGGGTGCATCATCGACCGATCCTGATG-
TCTTCGGATGGATTTGAGTAAGAGCATAACTGTTCGGACCCGAAAGATGGTGA
ACTATGCGTGGATAGGGTGAAGCCAGAGGAAACTCTGGTGGAGGCTCGCAGCG
GTTCTGACGTGCAAATCGATCGTCAAATCTGCGCATGGGGGCGAAAGTATGTA
TAATCCCTTCCTTCTGTAC-----------------------------------AGGAAGCAG--
AACGCTAACAACAATCTC-TTTAGGACTTATCGAACCATC------------------------------
----

TAGTAGCTGGTTACCGCCGAAGTTTCCCTCAGGATAGCAGTGTTG--
TTTTCAGTTTTATGAGGTAAAGCGAATGATTAGGGACTCGGGGGCGCTTTATTG
CCTTCATCCATTCTCAAACTTTAAATATGTAAGAAGCCCTTGTTGCTTAATTGA
ACGTGGGCATTCGAATGTATCAACACTAGTGGGCCATTTTTGGTAAGCAGAACT
GGCGATGCGGGATGAACCGAACGCGGGGTTAAGGTGCCAGAGTGGACGCTCAT
CAGACACCACAAAAGGTGTTAGTACATCTAGACAGT------------------------------------
----

TGGACGGTGGCCATGGAAGTCGGAATCCGCTAAGGACTGTGTAACAACTCACC
AACCGAATGTACTAGCCCTGAAAATGGATGGCGCTCAAGCGTCT-
CACCCATACCTCGCCCTTAGGGTAGAAACGATGCCCTAAGG--------------------------
----

>H_petriniae_CBS_114746

--CC-
--------TCGTGGTCCGAGTTGTAATTTGTAGAGGATGCTTTTGGTGCGGT-
GCCTTCCGAGTTCCCTGGAACGGGACGCCAGAGAGGGTGAGAGCCCCGTACGG
TTGGA-
CACCTACCCTATATGTAGCTCCTTCGACGAGTCGAGTAGTTTGGGAATGCTGCT
CTAAATGGGAGGTAAATTTCTTCTAAAGCTAAATACCGGCCAGAGACC-
GATAGCGCACAAGTAGAGTGATCGAAAGATGAAAAGCACTTTGAAAAGAGGG
TTAAATAGCACGTGAAATTGTTGAAAGGGAAGCG-
TTTGCGACCAGACCTTTTCTAGGCGGATCATCCGGTG-TTC-
TCACCGGTGCACTTCGCCTGG--TTTAGGCCAGCATCGGTTTT---
CGTAAGAGGATAAAGGCTCGGGGAACGTATCTCT----CTC--GGGAGTGTT-
ATAGCCCCTTGTGTAATACTCTT-ATGGGGACCGAGGACCGCGC-TTC---
GGCAAGGATGCTGGCGTAATGGTCGTCAACGACCCGTCTTGAAACACGGACCA
AGGAGTCGAACATTTGTGCGAGTGTTTGGGTA--
TTAAACCCTCACGCGTAATGAAAGTGAACGGAGGTGAGAGCCCTT-------
CGGGGTGCATCATCGACCGATCCTGATG-
TCTTCGGATGGATTTGAGTAAGAGCATAACTGTTCGGACCCGAAAGATGGTGA
ACTATGCGTGGATAGGGTGAAGCCAGAGGAAACTCTGGTGGAGGCTCGCAGCG
GTTCTGACGTGCAAATCGATCGTCAAATCTGCGCATGGGGGCGAAAGTATGTA
TAATCCCTTCCTTCTATAC--
AGGAAGCAGTAAAGGCTAACAATAATCTCTTTTAGGACTTATCGAACCATC-----

TAGTAGCTGGTTACCGCCGAAGTTTCCCTCAGGATAGCAGTGTTG--
TTTTCAGTTTTATGAGGTAAAGCGAATGATTAGGGACTCGGGGGCGCTTTATTG
CCTTCATCCATTCTCAAACTTTAAATATGTAAGAAGCCCTTGTTGCTTAATTGA
ACGTGGGCATTCGAATGTATCAACACTAGTGGGCCATTTTTGGTAAGCAGAACT
GGCGATGCGGGATGAACCGAACGTGGAGTTAAGGTGCCAGAGTGGACGCTCAT
CAGACACCACAAAAGGTGTTAGTACATCTAGACAGT---

TGGACGGTGGCCATGGAAGTCGGAATCCGCTAAGGACTGTGTAACAACTCACC
AACCGAATGTACTAGCCCTGAAAATGGATGGCGCTCAAGCGTCT-

CACCCATACTTCACCCTTAGGGTAGAAACGATGCCCTAAGG-AGTAGGCG--------

>H_gibriacense_MUCL_52698

---TGCCCCAGTAACGGCGAG--
TGAAGCGGCAACAGCTCAAA-TTTGAAATCTGGCCC---------
TCGTGGTCCGAATTGTAATTTGCAGAGGATGCTTTTGGTGCGGT-
GCCTTCCGAGTTCCCTGGAACGGGACGCCAGAGAGGGTGAGAGCCCCGTACGG
TTGGA-
CACCTACCCTATATATAGCTCCTTCGACGAGTCGAGTAGTTTGGGAATGCTGCT
CTAAATGGGAGGTAAATTTCTTCTAAAGCTAAATACCGGCCAGAGACC-
GATAGCGCACAAGTAGAGTGATCGAAAGATGAAAAGCACTTTGAAAAGAGGG
TTAAATAGCACGTGAAATTGTTGAAAGGGAAGCG-
TTTGCGACCAGACTTTTTCCAGGCGGATCATCCGGTG-TTC-
TCACCGGTGCACTTCGCCTGG--TTTAGGCCAGCATCGGTTTT---
CTTAGGGGGATAAAGGCCTGGGGCACGTAGCTCC----CTC--GGGAGTGTT-
ATAGCCCCTAGCGTAATACCTTT-CAGGGGACCGAGGACCGCGC-TTC---
GGCAAGGATGCTGGCGTAATGGTTGTCAACGACCCGTCTTGAAACACGGACCA
AGGAGTCGAACATTTGTGCGAGTGTTTGGGTA--
TTAAACCCTCACGCGTAATGAAAGTGAACGGAGGTGAGAGCCCTT-------
CGGGGTGCATCATCGACCGATCCTGATG-
TCTTCGGATGGATTTGAGTAAGAGCATAACTGTTCGGACCCGAAAGATGGTGA
ACTATGCGTGGATAGGGTGAAGCCAGAGGAAACTCTGGTGGAGGCTCGCAGCG
GTTCTGACGTGCAAATCGATCGTCAAATCTGCGCATGGGGGCGAAAGTAAGTT
TTACCTACCCGGTAGCTACC------CTGT-----------------------AATAAAATTA--
AATACTAACAAGAATCTC-TTTAGGACTTATCGAACCATC-----------------------------
----

TAGTAGCTGGTTACCGCCGAAGTTTCCCTCAGGATAGCAGTGTTG--
TTTTCAGTTTTATGAGGTAAAGCGAATGATTAGGGACTCGGGGGCGCTTTATTG
CCTTCATCCATTCTCAAACTTTAAATATGTAAGAAGCCCTTGTTGCTTAATTGA
ACGTGGGCATTCGAATGTATCAACACTAGTGGGCCATTTTTGGTAAGCAGAACT

GGCGATGCGGGATGAACCGAACGCGAGGTTAAGGTGCCAGAGTGGACGCTCAT
CAGACACCACAAAAGGTGTTAGTACATCTAGACAGT----------------------------------
----

TGGACGGTGGCCATGGAAGTCGGAATCCGCTAAGGACTGTGTAACAACTCACC
AACCGAATGTACTAGCCCTGAAAATGGATGGCGCTCAAGCGTCT-
CACCCATACCTCGCCCTTAAGGTAGAAACGATGCCCTAAGG-
AGTAGGCGGCCGTAG--AGG-TCAGTGACGAAGCCTAGGGCG-------------------------
----

>H_porphyreum_CBS_119022

---ATTGCCCTAGTAACGGCGAG--
TGAAGCGGCAACAGCTCAAA-TTTGAAATCTGGCCC---------
TCGTGGTCCGAATTGTAATTTGTAGAGGATGCTTTTGGTGCGGT-
GCCTTCTGAGTACCCTGGAACGGGTCGCCAGAGAGGGTGAGAGCCCCGTACGG
TTGGA-
CACCTACCCTATATATAGCTCCTTCGACGAGTCGAGTAGTTTGGGAATGCTGCT
CTAAATGGGAGGTAAATTTCTTCTAAAGCTAAATACCGGCCAGAGACC-
GATAGCGCACAAGTAGAGTGATCGAAAGATGAAAAGCACTTTGAAAAGAGGG
TTAAATAGCACGTGAAATTGTTGAAAGGGAAGCG-
TTTGCGACCAGACCTTCTCCTGGCGGATCATCCGGTG-TTT-
TCACCGGTGCACTTTGCCAGG--TCTAGGCCAGCATCGGTTTC---
TTTAGGGGGATAAAGGCGTTGGGAACGTAGCTCT----TTC--GGGAGTGTT-
ATAGCCCTTCACGTAATACCCTT-CGAGGGACCGAGGACCGCGC-TCT----
GCAAGGATGCTGGCGTAATGGTCGTCAACGACCCGTCTTGAAACACGGACCAA
GGAGTCGAACATTTGTGCGAGTGTTTGGGTA--
TTAAACCCTCACGCGTAATGAAAGTGAACGGAGGTGAGAGCCCTT-------
ACGGGTGCATCATCGACCGATCCTGATG-
TCTTCGGATGGATTTGAGTAAGAGCATAACTGTTCGGACCCGAAAGATGGTGA
ACTATGCGTGGATAGGGTGAAGCCAGAGGAAACTCTGGTGGAGGCTCGCAGCG
GTTCTGACGTGCAAATCGATCGTCAAATCTGCGCATGGGGGCGAAA--------------

GACTTATCGAACCATC---

TAGTAGCTGGTTACCGCCGAAGTTTCCCTCAGGATAGCAGTGTTG--
TATTCAGTTTTATGAGGTAAAGCGAATGATTAGGGACTCGGGGGCGCTTTATTG
CCTTCATCCATTCTCAAACTTTAAATATGTAAGAAGCCCTTGTTACTTAATTGA
ACGTGGGCATTCGAATGTATCAACACTAGTGGGCCATTTTTGGTAAGCAGAACT
GGCGATGCGGGATGAACCGAACGCGGGGTTAAGGTGCCAGAGTGGACGCTCAT
CAGACACCACAAAAGGTGTTAGTACATCTTGACAGCAGGTATGTCTACCTACC
CTATACCCCT---
-----------------------------CCTACCCT--

GTAGGACCTTACCCTGT---
AGCAATCATACTAACCCTTTTTCTCTAGGACGGTGGCCATGGAAGTCGGAATCC
GCTAAGGACTGTGTAACAACTCACCTGCCGAATGTACTAGCCCTGAAAATGGA
TGGCGCTCAAGCGTCT-
CACCCATACCTCGCCCTTAGGGTAGAAACGATGCCCTAAGG-
AGTAGGCGGCCGTGG--AGG-T---

>H_pseudofuscum_DSM112038

--ATTGCCCTAGTAACGGCGAG--
TGAAGCGGCAACAGCTCAAA-TTTGAAATCTGGCCC---------
TCGTGGTCCGAGTTGTAATTTGTAGAGGATGCTTTTGGTGCGGT-
GCCTTCCGAGTTCCCTGGAACGGGACGCCAGAGAGGGTGAGAGCCCCGTACGG
TTGGA-
CACCTACCCTATATATAGCTCCTTCGACGAGTCGAGTAGTTTGGGAATGCTGCT
CTAAATGGGAGGTAAATTTCTTCTAAAGCTAAATACCGGCCAGAGACC-
GATAGCGCACAAGTAGAGTGATCGAAAGATGAAAAGCACTTTGAAAAGAGGG
TTAAATAGCACGTGAAATTGTTGAAAGGGAAGCG-
TTTGCGACCAGACCTTTTCCCGGCGGATCATCTGGTG-TTC-
TCACCGGTGCACTTCGCTTGG--TTTAGGCCAGCATCGGTTTC---
TTTAGGGGGATAAAGGTGTTGGGAACGTAGCTCT----TTC--GGGAGTGTT-
ATAGCCCTTCACGTAATACCCTT-CGAGGGACCGAGGACCGCGC-ATT----
GCAAGGATGCTGGCGTAATGGTCGTCAACGACCCGTCTTGAAACACGGACCAA
GGAGTCGAACATTTGTGCGAGTGTTTGGGTG--
TTAAACCCTCACGCGTAATGAAAGTGAACGGAGGTGAGAGCCCTT-------

ACGGGTGCATCATCGACCGATCCTGATG-
TCTTCGGATGGATTTGAGTAAGAGCATAACTGTTCGGACCCGAAAGATGGTGA
ACTATGCGTGGATAGGGTGAAGCCAGAGGAAACTCTGGTGGAGGCTCGCAGCG
GTTCTGACGTGCAAATCGATCGTCAAATCTGCGCATGGGGGCGAAA---------------
----
GACTTATCGAACCATC--

TAGTAGCTGGTTACCGCCGAAGTTTCCCTCAGGATAGCAGTGTTG--
TATTCAGTTTTATGAGGTAAAGCGAATGATTAGGGACTCGGGGGCGCTTTATTG
CCTTCATCCATTCTCAAACTTTAAATATGTAAGAAGCCCTTGTTGCTTAATTGA
ACGTGGGCATTCGAATGTATCAACACTAGTGGGCCATTTTTGGTAAGCAGAACT
GGCGATGCGGGATGAACCGAACGCGGGGTTAAGGTGCCAGAGTGGACGCTCAT
CAGACACCACAAAAGGTGTTAGTACATCTTGACAGCAGGTATGTCTAACTACC
CTATACCCCC---
------------------TACCCTGTACCTACCCG--

GTACGGGC-TACCCTGT---
AACAATCATACTAAC--
TCGCTTTCTAGGACGGTGGCCATGGAAGTCGGAATCCGCTAAGGACTGTGTAA
CAACTCACCTGCCGAATGTACTAGCCCTGAAAATGGATGGCGCTCAAGCGTCT-
CACCCATACCTCGCCCTTAGGGTAGAAACGATGCCCTAAGG-
AGTAGGCGGCCGTGG--AGG--

>H_fuscoides_MUCL_52670

--AGGGATTGCCCTAGTAACGGCGAG--
TGAAGCGGCAACAGCTCAAA-TTTGAAATCTGGCCC---------
TCGTGGTCCGAGTTGTAATTTGTAGAGGATGCTTTTGGTGCGGT-
GCCTTCCGAGTTCCCTGGAACGGGACGCCAGAGAGGGTGAGAGCCCCGTACGG
TTGGA-
CACCTACCCTATATATAGCTCCTTCGACGAGTCGAGTAGTTTGGGAATGCTGCT
CTAAATGGGAGGTAAATTTCTTCTAAAGCTAAATACCGGCCAGAGACC-
GATAGCGCACAAGTAGAGTGATCGAAAGATGAAAAGCACTTTGAAAAGAGGG

TTAAATAGCACGTGAAATTGTTGAAAGGGAAGCG-
TTTGCGACCAGACCTTTTCCCGGCGGATCATCTGGTG-TTC-
TCACTGGTGCACTTCGCTTGG--TTTAGGCCAGCATCGGTTTC---
TTTAGGGGGACAAAGGCGTTGGGAACGTAGCTCT----TTC--GGGAGTGTT-
ATAGCCCTTCACGTAATACCCTT-CGAGGGACCGAGGACCGCGC-ATT----
GCAAGGATGCTGGCGTAATGGTCGTCAACGACCCGTCTTGAAACACGGACCAA
GGAGTCGAACATTTGTGCGAGTGTTTGGGTG--
TTAAACCCTCACGCGTAATGAAAGTGAACGGAGGTGAGAGCCCTT-------
ACGGGTGCATCATCGACCGATCCTGATG-
TCTTCGGATGGATTTGAGTAAGAGCATAACTGTTCGGACCCGAAAGATGGTGA
ACTATGCGTGGATAGGGTGAAGCCAGAGGAAACTCTGGTGGAGGCTCGCAGCG
GTTCTGACGTGCAAATCGATCGTCAAATCTGCGCATGGGGGCGAAA--------------

GACTTATCGAACCATC--

TAGTAGCTGGTTACCGCCGAAGTTTCCCTCAGGATAGCAGTGTTG--
TATTCAGTTTTATGAGGTAAAGCGAATGATTAGGGACTCGGGGGCGCTTTATTG
CCTTCATCCATTCTCAAACTTTAAATATGTAAGAAGCCCTTGTTGCTTAATTGA
ACGTGGGCATTCGAATGTATCAACACTAGTGGGCCATTTTTGGTAAGCAGAACT
GGCGATGCGGGATGAACCGAACGCGGGGTTAAGGTGCCAGAGTGGACGCTCAT
CAGACACCACAAAAGGTGTTAGTACATCTTGACAGCAGGTATGTCTAACTACC
CTACAACCCCT---
------------------TACCCTGTACCTACCCG--

GTACGGGCTTACCCTAT---
AACAATCATACTAAC--
TCGTTTTCTAGGACGGTGGCCATGGAAGTCGGAATCCGCTAAGGACTGTGTAA
CAACTCACCTGCCGAATGTACTAGCCCTGAAAATGGATGGCGCTCAAGCGTCT-
CACCCATACCTCGCCCTTAGGGTAGAAACGATGCCCTAAGG-
AGTAGGCGGCCGTGG--AGG-TT-GTGACGAAGCCTAGGC-----------------------------
----

>X_arbuscula_CBS_126415

---GGCGAG--
TGAAGCGGCAACAGCTCAAA-TTTGAAATCTGGCCT---------TCG-
GGTCCGAGTTGTAATTTGTAGAGGATGCTTTTGGCGCGGT-
GCCTTCCGAGTTCCCTGGAACGGGACGCCTTAGAGGGTGAGAGCCCCGTACGG
TTGGA-
CACCAAGCCTCTGTAAAGCTCCTTCGACGAGTCGAGTAGTTTGGGAATGCTGCT
CTAAATGGGAGGTAAATTTCTTCTAAAGCTAAATATTGGCCAGAGACC-
GATAGCGCACAAGTAGAGTGATCGAAAGATGAAAAGCACTTTGAAAAGAGGG
TTAAATAGCACGTGAAATTGTTGAAAGGGAAGCG-
TTTGCGACCAGACTTTTCCCTAGCGGATCATCCGGTG-TTC-
TCACCGGTGCACTTCGCTAGG--TTAAGGCCAGCATCGGTTTC---
TGTAGGGGGATAAAAGCTTGGGGAATGTAGCTCC----CTC--GGGAGTGTT-
ATAGCCTCTTGTATAATACCCTT-ACGGGGACCGAGGACCGCGC-TTT---
TGCAAGGATGCTGGCGTAATGGTTGTCAACGACCCGTCTTGAAACACGGACCA
AGGAGTCGAACATTTGTGCGAGTGTTTGGGTG--
TTAAACCCTCACGCGTAATGAAAGTGAACGGAGGTGAGAGCCCTT-------
ACGGGTGCATCATCGACCGATCCTGATG-
TCTTCGGATGGATTTGAGTAAGAGCATAACTGTTCGGACCCGAAAGATGGTGA
ACTATGCGTGGATAGGGTGAAGCCAGAGGAAACTCTGGTGGAGGCTCGCAGCG
GTTCTGACGTGCAAATCGATCGTCAAATCTGCGCATGGGGGCGAAA---------------
----
GACTTATCGAACCATC--

TAGTAGCTGGTTACCGCCGAAGTTTCCCTCAGGATAGCAGTGTTGT-
TCTTCAGTTTTATGAGGTAAAGCGAATGATTAGGGACTCGGGGGCGCTTTTTAG
CCTTCATCCATTCTCAAACTTTAAATATGTAAGAAGCCCTTGTTACTTAATTGA
ACGTGGGCATTCGAATGTACCAACACTAGTGGGCCATTTTTGGTAAGCAGAAC
TGGCGATGCGGGATGAACCGAACGCGGGGTTAAGGTGCCGGAGTGGACGCTCA
TCAGACACCACAAAAGGTGTTAGCACATTTAGACAAT----------------------------------
----

AGGACGGTGGCCATGGAAGTCGGAATCCGCTAAGGACTGTGTAACAACTCACC
TATCGAATGTGCTAGCCCTGAAAATGGATGGCGCTCAAGCGTCC-
CACCCATACCCCGCCCTCAGGGTAGAAACGATGCCCTGAGG-
AGTAGGCGGCCGTGG--AGG-TCAGTGACGAAGCCTAGGGC----------------------------
----

>X_hypoxylon_CBS_122620

---AG--
TGAAGCGGCAACAGCTCAAA-TTTGAAATCTGGCTT---------TCG-
GGTCCGAGTTGTAATTTGTAGAGGATGCTTTTGGCGCGGT-
GCCTTCCGAGTTCCCTGGAACGGGACGCCTTAGAGGGTGAGAGCCCCGTACGG
TTGGA-
CACCAAGCCTCTGTAAAGCTCCTTCGACGAGTCGAGTAGTTTGGGAATGCTGCT
CTAAATGGGAGGTAAATTTCTTCTAAAGCTAAATATTGGCCAGAGACC-
GATAGCGCACAAGTAGAGTGATCGAAAGATGAAAAGCACTTTGAAAAGAGGG
TTAAATAGCACGTGAAATTGTTGAAAGGGAAGCG-
TTTGCGACCAGACCTTTTCTTAGCGGATCATCCGGTG-TTA-
TCACCGGTGCACTTCGCTAAG--TTTAGGCCAGCATCGGTTTC---
TGTAGGGGGATAAAAGCCTTGGGAACGTAGCTCC----TTC--GGGAGTGTT-
ATAGCCCTTTGCATAATACCCTT-CTGGGGACCGAGGACCGCGC-TAT--
ATGCAAGGATGCTGGCATAATGGTCGTCAACGACCCGTCTTGAAACACGGACC
AAGGAGTCGAACATTTATGCGAGTGTTTGGGTG--
TTAAACCCTCACGCGTAATGAAAGTGAACGGAGGTGAGAGCCCTT-------
ACGGGTGCATCATCGACCGATCCTGATG-
TCTTCGGATGGATTTGAGTAAGAGCATAACTGTTCGGACCCGAAAGATGGTGA
ACTATGCGTGGATAGGGTGAAGCCAGAGGAAACTCTGGTGGAGGCTCGCAGCG
GTTCTGACGTGCAAATCGATCGTCAAATCTGCGCATGGGGGCGAAA--------------

GACTTATCGAACCATC---

TAGTAGCTGGTTACCGCCGAAGTTTCCCTCAGGATAGCAGTGTTGT-
TCTTCAGTTTTATGAGGTAAAGCGAATGATTAGGGACTCGGGGGCGCTTTTTG
CCTTCATCCATTCTCAAACTTTAAATATGTAAGAAGCCCTTGTTACTTAATTGA
ACGTGGGCATTCGAATGTACCAACACTAGTGGGCCATTTTTGGTAAGCAGAAC
TGGCGATGCGGGATGAACCGAACGCGGGGTTAAGGTGCCGGAGTGGACGCTCA
TCAGACACCACAAAAGGTGTTAGCACATTTAGACAAT-------------------------------
----

AGGACGGTGGCCATGGAAGTCGGAATCCGCTAAGGACTGTGTAACAACTCACC
TATCGAATGTGCTAGCCCTGAAAATGGATGGCGCTCAAGCGTCC-
CACCCATACCTCGCCCTCAGGGTAGAAACGATGCCCTGAGG-AGTAGGCGGC----

>Natonodosa_speciosa_CLM_RV86

TTGACCTCGGATCAGGTAGGAATACCCGCTGAACTTAAGCATATCAATAAGCG
GAGGAAAAGAAACCAACAGGGATTGCCCTAGTAACGGCGAG--
TGAAGCGGCAACAGCTCAAA-TTTGAAATCTGGCCC---------TCG-
GGTCCGAATTGTAATTTGTAGAGGATGTTTTGGCGCGGT-
GCCTTCCGAGTTCCCTGGAACGGGACGCCTTAGAGGGTGAGAGCCCCGTACGG
TTGGA-
CATCAAGCCTCTGTAAAGCTCCTTCGACGAGTCGAGTAGTTTGGGAATGCTGCT
CTAAATGGGAGGTAAATTTCTTCTAAAGCTAAATACCGGCCAGAGACC-
GATAGCGCACAAGTAGAGTGATCGAAAGATGAAAAGCACTTTGAAAAGAGGG
TTAAATAGCACGTGAAATTGTTGAAAGGGAAGCG-
TTTACGACCAGACCTTCTGTAGGCGGATCATGTGGTG-TTC-
TCACCGCTGCACTTCGCCTGC--TGTAGGCCAGCATCGGTTTC---
CGCCGGGGGATAAAAACTTGGGGAAAGTAGCTCT----CTTCGGGGAGTGTT-
ATAGCCCCTTGTATAATGCCCTG-GTGGGGACCGAGGACCGCGC-TTC---
GGCAAGGATGCTGGCATAATGGTCGTCAACGACCCGTCTTGAAACACGGACCA
AGGAGTCGAACATTTGTGCAAGTGTTTGGGTG--
TTAAACCCTCACGCGTAATGAAAGTGAACGGAGGTGAGAGCCCTT-------
ACGGGTGCATCATCGACCGATCCTGATG-
TCTTCGGATGGATTTGAGTAAGAGCATAACTGTTCGGACCCGAAAGATGGTGA
ACTATGCGTGGGTAGGGTGAAGCCAGAGGAAACTCTGGTGGAGGCTCGCAGCG
GTTCTGACGTGCAAATCGATCGTCAAACCTGCGCATGGGGGCGAAA---------------
----
GACTAATCGAACCATC---

TAGTAGCTGGTTACCGCCGAAGTTTCCCTCAGGATAGCAGTGTTG--
TCTTCAGTTTTATGAGGTAAAGCGAATGATTAGGGACTCGGGGGCGCTATTTAG
CCTTCATCCATTCTCAAACTTTAAATATGTAAGAAGCCCTTGTTACTTAATTGA
ACGTGGGCATTCGAATGTACCAACACTAGTGGGCCATTTTTGGTAAGCAGAAC
TGGCGATGCGGGATGAACCGAACGCGGGGTTAAGGTGCCAGAGTAGACGCTCA
TCAGACACCACAAAAGGTGTTAGTACATCTAGACAGC----------------------------------

----

AGGACGGTGGCCATGGAAGTCGGAATCCGCTAAGGACTGTGTAACAACTCACC
TGCCGAATGTACTAGCCCTGAAAATGGATGGCGCTCAAGCGTCT-
CACCCATACCTCGCCCTCAGGGTAGAAACGATGCCCTGAGG-
AGTAGGCGGCCGTGG--AGG-
TCAGTGACGAAGCCTAGGGCGTGAGCCCGGGTAGAACGGCCTCTAGTGCAGAT
CTTGGTGGTAGTAGCAAATACTTCAATGAGAACTTGAAGGACCGAAGTGGGGA
AAGGTTCCATGTGAACAGCGGTTGGACGTGGGTTAGCCGATCCTAAGCCATAG
GGAAGTTCCGTTTCAAAGTGAGCACTTGTGCTCCGTGTGGCGAAAGGGAAGCC
GGTCAATATTCCGGCGCTTGGATTTGGGTTTTGCGCGGCAACGCAACTGAACGT
GGAGACGACGGCGGGGGCCCTGGGCAGAGTTCTCTTTTCTTCTTAACGGTCTAT
CACCCTGAAATCGGTTTGTCCGGAGCTAGGGTTTAACGGCCGGAAGAGCCCGA
CACCTCTGTCGGGTCTGGTGTGCCCCCGACGTCCCTTGAAAATCCACGGGAGGG
AATAATTCTCAAGCCAAGTCGTACTCATAACCGCAGCAGG

>H_sporistriatatunicum_UCH9542

---AGGGATTGCCCTAGTAACGGCGAG--
TGAAGCGGCAACAGCTCAAA-TTTGAAATCTGGCCT---------TCG-
GGTCCGAATTGTAATTTGCAGAGGATGCTTTTGGTGCGGT-
GCCTTCCGAGTTCCCTGGAACGGGACGCCAGAGAGGGTGAGAGCCCCGTACGG
TTGGA-
CACCTACCCTATATATAGCTCCTTCGACGAGTCGAGTAGTTTGGGAATGCTGCT
CTAAATGGGAGGTAAATTTCTTCTAAAGCTAAATACCGGCCAGAGACC-
GATAGCGCACAAGTAGAGTGATCGAAAGATGAAAAGCACTTTGAAAAGAGGG
TTAAATAGCACGTGAAATTGTTGAAAGGGAAGCG-
TTTGTGACCAGACTTTTTCCAGGCGGATCATCCGGTG-TTC-
TCACCGGTGCACTTCGCCTGG--TTTAGGCCAGCATCGGTTTC---
CGTAGGGGGATAAAAACCTGGGGCATGTGGCTCT----CTC--GAGAGTGTT-
ATAGCCCCTCGTATAATACCCCT-CCGGGGACCGAGGACCGCGC-TTC---
GGCAAGGATGCTGGCGTAATGGTTATCAACGACCCGTCTTGAAACACGGACCA
AGGAGTCGAACATTTGTGCGAGTGTTTGGGTG--
TTAAACCCTCACGCGTAATGAAAGTGAACGGAGGTGAGAGCCCTT-------
CGGGGTGCATCATCGACCGATCCTGATG-
TCTTCGGATGGATTTGAGTAAGAGCATAACTGTTCGGACCCGAAAGATGGTGA
ACTATGCGTGGATAGGGTGAAGCCAGAGGAAACTCTGGTGGAGGCTCGCAGCG
GTTCTGACGTGCAAATCGATCGTCAAATCTGCGCATGGGGGCGAAA---------------
----

GACTTATCGAACCATC---

TAGTAGCTGGTTACCGCCGAAGTTTCCCTCAGGATAGCAGTGTTG--
TTTTCAGTTTTATGAGGTAAAGCGAATGATTAGGGACTCGGGGGCGCTTTATTG
CCTTCATCCATTCTCAAACTTTAAATATGTAAGAAGCCTTTGTTACTTCATTGAA
CGTAGGCATTCGAATGTATCAACACTAGTGGGCCATTTTTGGTAAGCAGAACTG
GCGATGCGGGATGAACCGAACGCGAGGTTAAGGTGCCAGAGTGGACGCTCATC
AGACACCACAAAAGGTGTTAGTACATCTAGACAGTTGGTATGTACCCTTTTTTC
ATTTTCCCC--CGTACA-------------
-----------------TCTACTCGGCAGCTACCCT------------
GGAGTAGCTACCCTGGAGTAGCTACCCTGGAGTAGCTACCCTGGAGTAGCTAC-
---CCTGG--------------
AGTAGCTACCCTGGAGATAGCTACCCTGTAGCT-------------------------
GTATACCCCTCACTACTTTGACCCATTCAACTAACTTTTTTCTTTTTAGGACGGT
GGCCATGGAAGTCGGAATCCGCTAAGGACTGTGTAACAACTCACCAACCGAAT
GTACTAGCCCTGAAAATGGATGGCGCTCAAGCGTCT-
CACCCATACCTCGCCCTTAGGGTAGAAACGATGCCCTAAGG-
AGTAGGCGGCCGTGG--AGG-TTAGTGACGAAGCCTAGGGCG-----------------------
----

>H_cyclobalanopsidis_FCATAS2714

AGCGGAAGAAGACCAACAGGGATTGCCCTAGTAACGGCGAG--
TGAAGCGGCAACAGCTCAAA-TTTGAAATCTGGCCC---------TCG-
GGTCCGAGTTGTAATTTGTAGAGGATGCTTTGGGTGCGGT-
GCCTTCCGAGTTCCCTGGAACGGGACGCCAGAGAGGGTGAGAGCCCCGTACGG
TTGGA-
CACCTACCCTGTAGATAGCTCCTTCGACGAGTCGAGTAGTTTGGGAATGCTGCT
CTAAATGGGAGGTAAATTTCTTCTAAAGCTAAATACCGGCCAGAGACC-
GATAGCGCACAAGTAGAGTGATCGAAAGATGAAAAGCACTTTGAAAAGAGGG
TTAAATAGCACGTGAAATTGTTGAAAGGGAAGCG-
TTTGCGACCAGACCTTCTCCAGGCGGATCATCTGGTG-TTC-
TCACCGGTGCACTTCGCCTGG--TCTAGGCCAGCATCGGTTTC---

TCTAGGGGGTAAAGGCGTGGGGAACGTAGCTCT----TTC--GGGAGTGTT-
ATAGCCCTTCGCGTAATACCTTT-CGAGGGACCGAGGACCGCGC-TCT----
GCAAGGATGCTGGCGTAATGGTCGTCAACGACCCGTCTTGAAACACGGACCAA
GGAGTCGAACATTTGTGCGAGTGTTTGGGTG--
TCAAACCCTCACGCGTAATGAAAGTGAACGGAGGTGAGAGCCCTT-------
ACGGGTGCATCATCGACCGATCCTGATG-
TCTTCGGATGGATTTGAGTAAGAGCATAACTGTTCGGACCCGAAAGATGGTGA
ACTATGCGTGGATAGGGTGAAGCCAGAGGAAACTCTGGTGGAGGCTCGCAGCG
GTTCTGACGTGCAAATCGATCGTCAAATCTGCGCATGGGGGCGAAAG------------

TACGTACCATATTCTC--

--TA-
TACCTATCCTTCTACGACG--ACACATATACTAACAGCTATG--CTTCTAG----------
----

GACTTATCGAACCATGAAACCAGCGGTAATATATAT--------------------------------
----

-----------------------------CTT--

ACTAGGTGGTTAAGA--GG--

>H_wuzhishanense_FCATAS2708

TTCCCCGGGAAATCTAGCATATCAATAAGCGGAGGAAAAGAAACCAACAGGG
ATTGCCCTAGTAACGGCGAG--TGAAGCGGCAACAGCTCAAA-
TTTGAAATCTGGCCC---------
TAGCGGTCCGAGTTGTAATTTGTAGAGGATGCTTTTGGTGCGGT-
GCCTTCCGAGTTCCCTGGAACGGGACGCCAGAGAGGGTGAGAGCCCCGTACGG
TTGGA-
CACCTACCCTATATATAGCTCCTTCGACGAGTCGAGTAGTTTGGGAATGCTGCT
CTAAATGGGAGGTAAATTTCTTCTAAAGCTAAATACCGGCCAGAGACC-

GATAGCGCACAAGTAGAGTGATCGAAAGATGAAAAGCACTTTGAAAAGAGGG
TTAAATAGCACGTGAAATTGTTGAAAGGGAAGCG-
TTTGCGACCAGACCTTTTCCGGGCGGATCATCCGGTG-TTC-
TCACCGGTGCACTTCGTCCGG--TTTAGGCCAGCATCGGTTTT---
TGTAGGGGGATAAAAGCCTGGGGAACGTGGCTCC----TTC--GGGAGTGTT-
ATAGCCCCTAGCATAATACCCCT-ACAGGGACCGAGGACCGCGC-TTC---
GGCAAGGATGCTGGCGTAATGGTCGTCAACGACCCGTCTTGAAACACGGACCA
AGGAGTCGAACATTTGTGCGAGTGTTTGGGTA--
TTAAACCCTCACGCGTAATGAAAGTGAACGGAGGTGAGAGCCCTT-------
CGGGGTGCATCATCGACCGATCCTGATG-
TCTTCGGATGGATTTGAGTAAGAGCATAACTGTTCGGACCCGAAAGATGGTGA
ACTATGCGTGGATAGGGTGAAGCCAGAGGAAACTCTGGTGGAGGCTCGCAGCG
GTTCTGACGTGCAAATCGATCGTCAAATCTGCGCATGGGGGCGAAA--------------
--G-----------TATG-------

TATAATCCTATCACCTACAAACTATGCAGATAGGAAGCAAATCAC--
TAACAACAACTCTTAGGACTATCGAACCATCTAGGTTT-------------------------------
----

>H_rickii_MUCL_53309

-------------------------------TAAAGGGTCGCGTGCATTGCTCGCGGCTTACTGCCAA--
----ACCCTATGTGAA-CTTACCACTGTTGCCTCGGCG--
GCGCCGCGGCTACCCTGGAGAGCT---------ACCCTGTAGC--------
CATTTACCCTGTAGATGGCTACCCTGTAGCTACCCT----
GTAGCTCGCACGTAAAGGCCCGTCAAAGGACCCTAAACTCTATTTTGACTTGGT
ATCTCTGAGTAAA----TCAACT-
TAATAAGTTAAAACTTTCAACAACGGATCTCTTGGTTCTGGCATCGATGAAGAA
CGCAGCGAAATGCGATAAGTAATGTGAATTGCAGAATTCAGTGAATCATCGAA
TCTTTGAACGCACATTGCGCCCATTAGTATTCTAGTGGGCATGCCTATTCGAGC
GTCATTTCAACCCCTTAAGCCCTGTTGCTTAGCGTTGGGGGCCT----

GCGCCTGGCGGCGTAGCCCCTGAAAGATAGTGGCGGAGTCAGTGAGCACTCTG
AGCGTAGTAAC------
TTTTCTCGCTCCGGTAGCTTGCCCCTGGCTGCTGGCCGTTAAA-----------
CCCCCCCATATCTTCTAGTGGTTGACCTCGGATTAGGTAGGAATACCCGCTGAA
CTTAAGCATATCAATAAGCGGAGGAAAAGAAACCAACAGGGATTGCCCTAGTA
ACGGCGAG--TGAAGCGGCAACAGCTCAAA-TTTGAAATCTGGCCC---------TAG-
GGCCCGAATTGTAATTTGTAGAGGATGCTTTGGGCGCGGT-
GCCTTCTGAGTTCCCTGGAACGGGACGCCAAAGAGGGTGAGAGCCCCGTACGG
TTGGC-
CACCAAGCCTGTATATAGCTCCTTCGACGAGTCGAGTAGTTTGGGAATGCTGCT
CTAAATGGGAGGTAAATTTCTTCTAAAGCTAAATACCGGCCAGAGACC-
GATAGCGCACAAGTAGAGTGATCGAAAGATGAAAAGCACTTTGAAAAGAGGG
TTAAATAGCACGTGAAATTGTTGAAAGGGAAGCG-
TTTGCGACCAGACCTTTTCCAGGCGGATCATCCGGGG-TTT-
TCTCCGGTGCACTTCGCCTGG--TTTAGGCCAGCATCGGTTCT---
CTTAGGGGGATAAAGGCCTGGGGAACGTAGCTCC----CTC--GGGAGTGTT-
ATAGCCCCTTGCGTAATACCCTT-CGGGGGACCGAGGAACGCGC-TCT----
GCAAGGATGCTGGCGTAATGGTCGTCAACGACCCGTCTTGAAACACGGACCAA
GGAGTCGAACATTTGTGCGAGTGTTTGGGTG--
TCAAACCCTCACGCGTAATGAAAGTGAACGGAGGTGAGAGCCCTT-------
CGGGGTGCATCATCGACCGATCCTGATG-
TCTTCGGATGGATTTGAGTAAGAGCATAACTGTTCGGACCCGAAAGATGGTGA
ACTATGCGTGGATAGGGTGAAGCCAGAGGAAACTCTGGTGGAGGCTCGCAGCG
GTTCTGACGTGCAAATCGATCGTCAAATCTGCGCATGGGGGCGAAA--------------

GACTTATCGAACCATT-----AAACCAGCGGTAAAAAAC-
ACTTTTACTAGGTGGTTAAGAGGCGCCCCGCGAGGGCGCG----
CCTAGGTCATCCGGCCGGTCTACCCTGGAGTTGGCTACCCTGCAG-----------------
--GGAGCTACCCGGTAGTCACCCACCCTGGAGCTAGCTACCCTGTAGCGATTC----
-----CAGGGAATGTCTACCCTGGAGCCACCCTGGAGAT--------------
AGCCACCCTGGAGTGACCGGCCGGGCCGACACCGCTAAATTGCGGGGACATCC
TACA--AAGGCCACAGGCTACCGCCGGGCGCTGAAAAGCGT---
GCCGGCACCGAGAATAGCGCTCTTGGGTACGGTAACAACGCCTGTGGCATGGG
ACGACCTGCAGCCAACCCCGC--
AGCCGTAGGGGAGAGTTCACTGACTAAACAGCGGTGGGTTGGCAGCGGCCTTC
CAGGCCCTGCCAGCCTAAGACATAGTCGATCCAGGCCCTGAAAAGGTGCCTAC
CCTGTA---
AAACATAGCGGAGCTAGTAGCTGGTTACCGCCGAAGTTTCCCTCAGGATAGCA
GTGTTG--
TCTTCAGTTTTATGAGGTAAAGCGAATGATTAGGGACTCGGGGGCGCTATATTG
CCTTCATCCATTCTCAAACTTTAAATATGTAAGAAGCCCTTGTTACTTAGTTGA
ACGTGGGCATTCGAATGTACCAACACTAGTGGGCCATTTTTGGTAAGCAGAAC
TGGCGATGCGGGATGAACCGAACGCGGGGTTAAGGTGCCAGAGTGGACGCTCA
TCAGACACCACAAAAGGTGTTAATACATTATGACAGC----------------------------------
----

CGGACTGTGGCCATGGAAGTCGGAATCAGCTAAGGACTGTGTAACAACTCACC
GGCCGAATGTATTAGCCCTGAAAATGGATGGCGCTCAAGCGTCT-
CACCCATACCTCGCCCTTAGGGTAGAAACGATGCCCTAAGG-
AGTAGGCGGCCGTGG--AGG-TCAG---

>H_vogesiacum_CBS_115273

--TCTCTGAATCCT----
TCAACTAAAATTAGTTAAAACTTTCAACAACGGATCTCTTGGTTCTGGCATCGA
TGAAGAACGCAGCGAAATGCGATAAGTAATGTGAATTGCAGAATTCAGTGAAT
CATCGAATCTTTGAACGCACATTGCGCCCATTAGTATTCTAGTGGGCATGCCTA
TTCGAGCGTCATTTCGACCCTGAAGCCCTGGCTGCTTCGCGTTGGGACTTCTAC
ATCTCTATATAGAGTAGTTCCTTAAAGTAATTGGCAGAGTTGGGGTATGCCCTA
AGCGTAGTATT----ATATTTCTCGCT-
TGAGGGTGTGTCCCTGGCTACCAGCCGTAAAG-----------------
CTGTTTTTATAGTGGTTGACCTCGGATTAGGTAGGAATACCCGCTGAACTTAAG
CATATCAATAAGCGGAGGAAAAGAAACCAACAGGGATTGCCCTAGTAACGGC
GAG--TGAAGCGGCAACAGCTCAAA-TTTGAAATCTGGCCC---------
TCGTGGTCCGAGTTGTACTTTGCAGAGGATGCTTTTGGTGCGGT-
GCCTTCCGAGTTCCCTGGAACGGGACGCCAGAGAGGGTGAGAGCCCCGTACGG
TCGGA-
CACCTACCCTATATATAGCTCCTTCGACGAGTCGAGTAGTTTGGGAATGCTGCT
CTAAATGGGAGGTAAATTTCTTCTAAAGCTAAATACCGGCCAGAGACC-
GATAGCGCACAAGTAGAGTGATCGAAAGATGAAAAGCACTTTGAAAAGAGGG
TTAAATAGCACGTGAAATTGTTGAAAGGGAAGCG-
TTTGCGACCAGACCTTTTCCTGGCGGATCATCCGGTG-TTC-
TCACCGGTGCACTTCGCCTGG--CTTAGGCCAGCATCGGTTTC---
TTTAGGGGGATAAAGGCTTGGGGAACGTAGCTCT----TTC--GGGAGTGTT-
ATAACCCCTTGTGTAATACCTTT-CGAGGGACCGAGGACCGCGC-TTC---
GGCAAGGATGCTGGCATAATGGTCGTCAACGACCCGTCTTGAAACACGGACCA
AGGAGTCGAACATTTGTGCGAGTGTTTGGGTG--
TTAAACCCTCACGCGTAATGAAAGTGAACGGAGGTGAGAGCCCTT-------
ACGGGTGCATCATCGACCGATCCTGATG-
TCTTCGGATGGATTTGAGTAAGAGCATAACTGTTCGGACCCGAAAGATGGTGA
ACTATGCGTGGATAGGGTGAAGCCAGAGGAAACTCTGGTGGAGGCTCGCAGCG
GTTCTGACGTGCAAATCGATCGTCAAATCTGCGCATGGGGGCGAAA---------------
----
GACTTATCGAACCATT---

AACCCAGCGGTAAATATCTA---

---TT---

ACTAGGTGGTTAAGA--

>H_chrysalidosporum_FCATAS2710

--TTAC----------------------------

CCCGCTGAAACTAAGCATATCAATAAGCGGAGGAAAAGAAACCAACAGGGAT
TGCCCTAGTAACGGCGAG--TGAAGCGGCAACAGCTCAAA-
TTTGAAATCTGGCCC---------
TCGCGGTCCGAGTTGTAATTTGTAGAGGATGCTTTGGGCGCGGT-
ACCTACCGAGTTCCCTGGAACGGGACGCCATAGAGGGTGAGAGCCCCGTACGG
CTGGA-
TACCTAGCCTCTGTATAGCTCCTTCGACGAGTCGAGTAGTTTGGGAATGCTGCT
CTAAACGGGAGGTAAATTTCTTCTAAAGCTAAATACCGGCCAGAGACC-
GATAGCGCACAAGTAGAGTGATCGAAAGATGAAAAGCACTTTGAAAAGAGGG
TTAAATAGCACGTGAAATTGTTGAAAGGGAAGCG-
TTTGCGACCAGACCTGTACCTGGCGGATCATCCGGTG-CTC-
GCACCGGTGCACTTCGCCTGG--CATAGGCCAGCATCGGCTTC---
GCCAGGGGGATAAAGGCTTAGGGAAAGTAGCTCC----CTCGTTGGAGTGTT-
ATAGCCCTTCGCGTAATGCCCTT-GGCGGGACCGAGGACCGCGC-TTC--
GTGCAAGGATGCTGGCATAATGGTCGTCAACGACCCGTCTTGAAACACGGACC
AAGGAGTCGAACATTTGTGCGAGTGTTAGGGCG--
TCAAACCCTTACGCGTAATGAAAGTGAACGGAGGTGAGAGCCTT---------
AGGGCGCATCATCGACCGATCCTGATG-
TCTTCGGATGGATTTGAGTAAGAGCATAACTGTTCGGACCCGAAAGATGGTGA
ACTATGCGTGGATAGGGTGAAGCCAGAGGAAACTCTGGTGGAGGCTCGCAGCG
GTTCTGACGTGCAAATCGATCGTCAAATCTGCGCATGGGGGCGAAA----------------

--GAC-
TATCGAACCATC--

---TTATCGGCG------

>H_subticinense_MUCL_53752

--TGCCCTAGTAACGGCGAG--
TGAAGCGGCAACAGCTCAAA-TTTGAAATCTGGCCC---------
TCGCGGCCCGAGTTGTAATTTGTAGAGGATGCTTTTGGCGCGGT-
GCCTTCTGAGTTCCCTGGAACGGGACGCCAGAGAGGGTGAGAGCCCCGTACGG
TTGGC-
CACCAAGCCTCTATATAGCTCCTTCGACGAGTCGAGTAGTTTGGGAATGCTGCT
CTAAATGGGAGGTAAATTTCTTCTAAAGCTAAATACCGGCCAGAGACC-
GATAGCGCACAAGTAGAGTGATCGAAAGATGAAAAGCACTTTGAAAAGAGGG
TTAAATAGCACGTGAAATTGTTGAAAGGGAAGCG-
TTTGCGACCAGACTTTCTCCAGGCGGATCATCCGGTG-TTC-
TCACCGGTGCACTTCGCCTGG--TTTAGGCCAGCATCGGTTCT---
CCTAGGGGGACAAAGGCCAGGGGAACGTAGCTCC----CTC--GGGAGTGTT-
ATAGCCCCTGGCGCAATACCCCT-CGGGGGACCGAGGAACGCGC-TCT----
GCAAGGATGCTGGCGTAATGGTCGTCAACGACCCGTCTTGAAACACGGACCAA
GGAGTCGAACATTTGTGCGAGTGTTTGGGTG--
TCAAACCCTCACGCGCAATGAAAGTGAACGGAGGTGAGAGCCCCT-------
CGGGGTGCATCATCGACCGATCCTGATG-
TCTTCGGATGGATTTGAGTAAGAGCATAACTGTTCGGACCCGAAAGATGGTGA
ACTATGCGTGGATAGGGTGAAGCCAGAGGAAACTCTGGTGGAGGCTCGCAGCG

GTTCTGACGTGCAAATCGATCGTCAAATCTGCGCATGGGGGCGAAA---------------
----
GACTTATCGAACCATTA----
AAACCAGCGGTAAAAAACGATTTTTACTAGGTGGTTAAGAGGCTTCCCGCGAG
GGAGAG----CCTAGGCCAGTGGGCCGACCTACCCTGGA----
GCTACCCTGTAGTCCTTGGGCGGACCTACCCTGGAGCTACCCTGTAGCGCCTGG
GCAG------
ACCTACCCTGGAGCTACCCTGTAGCGCGCGGGAGACACCTACCCTGGAGCTAC
CCTGTAGCGCCTGGGCGGTGGCTCTCCCCCCGGGGCGGGCCGGCCTGGCCGAC
ACCGCTAAATTGCGGGGACATCCTACA-
GAAGGCCGCAGGCTACCGCCGGGCGCTGAAAAGCGC---
CCCGGCACCAAGAATAGCGCTCTTGGGTATGGTAAGAACGCCTGCGGTACCGG
ACGACCTGCAGCCAACCCCGC--
ACGCCCAGGGGAGAGTTCACTGACTATACAGCGGTGGGTCAGC-------------
TACGGCTGGCCTAAGACATAGTCGATCCGGGCCCTGAAAAGGTGCCCAGCACA
CCC--A-
CAGACTCGTGGCGGAGCTAGTAGCTGGTTACCGCCGAAGTTTCCCTCAGGATA
GCAGTGTTG--
TCTTCAGTTTTATGAGGTAAAGCGAATGATTAGGGACTCGGGGGCGCTATTTTG
CCTTCATCCATTCTCAAACTTTAAATATGTAAGAAGCCCTTGTTACTTAGCTGA
ACGTGGGCATTCGAATGTACCAACACTAGTGGGCCATTTTTGGTAAGCAGAAC
TGGCGATGCGGGATGAACCGAACGCGGGGTTAAGGTGCCAGAGTGGACGCTCA
TCAGACACCACAAAAGGTGTTAATACATTATGACAGC--------------------------------
----

AGGACGGTGGCCATGGAAGTCGGAATCCGCTAAGGACTGTGTAACAACTCACC
TGCCGAATGTATTAGCCCTGAAAATGGATGGCGCTCAAGCGTCT-
CACCCATACCTCGCCCTTAGGGTAGAAACGATGCCCTAAGG-
AGTAGGCGGCCGTGG--AGG-TTAGTGACGAAGCCTAGGGCGGAGC----------------
----

>H_baruense_UCH9545

---------------------------------------AACCAACAGGGATTGCCCTAGTAACGGCGAG--
TGAAGCGGCAACAGCTCAAA-TTTGAAATCTGGCCC---------
CCGCGGTCCGAGTTGTAATTTGTAGAGGAAGCTTTTGGCGCGGT-

GCCTTCTGAGTTCCCTGGAACGGGACGCCAGAGAGGGTGAGAGCCCCGTACGG
TTGGC-
CACCAAGCCTCTATAAAGCTCCTTCGACGAGTCGAGTAGTTTGGGAATGCTGCT
CTAAATGGGAGGTAAATTTCTTCTAAAGCTAAATACCGGCCAGAGACC-
GATAGCGCACAAGTAGAGTGATCGAAAGATGAAAAGCACTTTGAAAAGAGGG
TTAAATAGCACGTGAAATTGTTGAAAGGGAAGCG-
TTTGCGACCAGACCTTCTCCAGGCGGATCATCCGGCG-TTC-
TCGCCGGTGCACTTCGTCTGG--TCTAGGCCAGCATCGGTTTT---
CCCAGGGGGATAAAGGCTTGGGGAACGTAGCTCC----CTC--GGGAGTGTT-
ATAGCCCCTCGCGTAATGCCCTT-GGGGGGACCGAGGACCGCGC-ATT----
GCAAGGATGCTGGCGTAATGGTCGTCAACGACCCGTCTTGAAACACGGACCAA
GGAGTCGAACATTTGTGCGAGTGTTTGGGTG--
TTAAACCCTCACGCGTAATGAAAGTGAACGGAGGTGAGAGCCCTT-------
CGGGGTGCATCATCGACCGATCCTGATG-
TCTTCGGATGGATTTGAGTAAGAGCATAACTGTTCGGACCCGAAAGATGGTGA
ACTATGCGTGGATAGGGTGAAGCCAGAGGAAACTCTGGTGGAGGCTCGCAGCG
GTTCTGACGTGCAAATCGATCGTCAAATCTGCGCATGGGGGCGAAA--------------

GACTTATCGAACCATC---

TAGTAGCTGGTTACCGCCGAAGTTTCCCTCAGGATAGCAGTGTTG--
TACTCAGTTTTATGAGGTAAAGCGAATGATTAGGGACTCGGGGGCGCTATTTA
GCCTTCATCCATTCTCAAACTTTAAATATGTAAGAAGCCCTTGTTACTTAGTTG
AACGTGGGCATTCGAATGTATCAACACTAGTGGGCCATTTTTGGTAAGCAGAA
CTGGCGATGCGGGATGAACCGAACGCGGGGTTAAGGTGCCAGAGTGGACGCTC
ATCAGACACCACAAAAGGTGTTAGTACATCTAGACAGCAGGTAAGCATCTTTT
TTTCTTTATTCAT-----------------------------------
GCAGGCTTCTGGGGGCAGTAGGCAGCCGATGTCT---------------------------------------
----

TATGCCTCTAGGCTCCCCCTGGCCTGAACTTCAGGCTCGGTGGGATAACGCCTA
GCCTTTTTGTTTTTATTATAGCGATAACTAACTCTTTTTCTTTTAGGACGGTGGC
CATGGAAGTCGGAATCCGCTAAGGACTGTGTAACAACTCACCTGCCGAATGTA
CTAGCCCTGAAAATGGATGGCGCTCAAGCGTCT-
CACCCATACCTCGCCCTTAGGGTAGAAACGATGCCCTAAGG-
AGTAGGCGGCCGTGG--AGG-TTAGTGACGAAGCCTAGGGC---------------------------
----

>Graphostroma_platystomum_CBS_270_87

AAGCGGAGGAAAAGAAACCAACAGGGATTGCCCTAGTAACGGCGAG--
TGAAGCGGCAACAGCTCAAA-TTTGAAATCTGGCTC---------TAG-
GGTCCGAATTGTAATTCGTAGAGGATGCTTTTGGCGCGGT-
CACTTCCGAGTTCCCTGGAACGGGACGCCTTAGAGGGTGAGAGCCCCGTACGG
TTGGA-
TANCAAGCCTCTGTAAAGCTCCTTCGACGAGTCGAGTAGTTCGGGAATGCTGCT
CTAAATGGGAGGTAANTTTCTTCTNAAGCTAAATNCCGGCCAGAGNCCNGATA
GCGCACAAGTAGAGTGATCGAAAGATGAAAAGCACTTTGAANAGAGGGTTAA
ATAGCACGTGAAATTGNTGAAAGGGAAGCGNTTTNCGGNCAGACCTTTTCCCA
GCGGNTCATNCAGTGNTTC-TCACTGGTGCACTCTGCTGGG--
TTTAGNCCAGCATCGGCTTC---
TGTAGGGGGATAAAAGCCNNGGGAAAGTAGNTCC----CTC--
GGGANTGTTNATNNNNCTAGGCNTNANNCNNTN-
NCGGGGGCCGAGGNNCGCNC-TCT----
GCAAGGANGCTGGCGTAATGGTCGTCANCGACCCGTCTTGAAACACGGACCAN
GGAGTCGAACATTTGTGCGAGTGTTTGGNTG--
TTAAACCCTCACGCGTAATGAAAGTGAACGGAGGTGAGAGCCCTTTA-----
CGGGGTGCATCATCGACCGATCCTGATG-
TCTTNGGATGGATTTGAGTAGGAGCATTAATGTTCGGACCCGAAAGATGGTGA
NCTATGCGTGGATAGGGTGAAGCCAGAGGAAACTCTGGTGGAGGCTCGCAGCG
GNTCTGACGTGCAAATCGATCGTCAAATCNGCGCATGGGGGCGAAA--------------

GACTTATCGAACCATC--

TAGTAGCTGGTTACCGCCGAAGTTTCCCTCAGGATAGCAGTGTTGT-
TCTTCAGTTTTATGAGGTAAAGCGAATGATTAGGGACTCGGGGGCGCTTATTAG
CCTTCATCCATTCTCAAACTTTAAATATGTAAGAAGCCCTTGTTGCTTAGTTGA
ACGTGGGCATTCGAATGTACCAACACTAGTGGGCCATTTTTGGTAAGCAGAAC
TGNCGATGCGGGATGAACCGAACGCGGGGTTAAGGTGCCGGAGTGGACGCTCA
TCAGACACCACAAAAGGTGTTAATACATCCAGACAGC--------------------------------
----

CGGACGGTGGCCATGGAAGTCGGAATCCGCTAAGGACTGTGTAACAACTCACC
GGCCGAATGTATTAGCCCTGAAAATGGATGNNGCTCAAGCGTCC-

CACCCATACCTCGNCCTCAGGGNAGAAACGATGCCCTGAGG-
AGTAGGCGGCCGTGG--AGG-TTAGTG--

>H_carneum_MUCL_54177

TTTGAAATCTGGCCC---------
TGGCGGCCCGAGTTGTAATTTGCAGAGGATGCTTTGGGCGCGGC-
GCCTTCCGAGTTCCCTGGAACGGGACGCCACAGAGGGTGAGAGCCCCGTACGG
TGGGA-
CGCCTACCCCGCGTATAGCTCCTTCGACGAGTCGAGTAGTTTGGGAATGCTGCT
CTAAACGGGAGGTAAATTTCTTCTAAGGCTAAATACCGGCCAGAGACC-
GATAGCGCACAAGTAGAGTGATCGAAAGATGAAAAGCACTTTGAAAAGAGGG
TTAAACAGCACGTGAAATTGTTGAAAGGGAAGCG-
TCCGCGACCAGACCTTCTCCAGGCGGATCCTACGGCG-TTC-
TCGACGTGGCCCTCCGCCTGG--TCTAGGCCAGCATCGGTCCC---
CGCGGGGGGAGAAAGGCCCGGGGAACGTGGCTCC----CCC--GGGAGTGTT-
ATAGCCCCGGGCGCAATGCCCCCGCGGGGGACCGAGGACCGCGC-TCT----
GCAAGGATGCTGGCGTAATGGTCGTCGACGACCCGTCTTGAAACACGGACCAA
GGAGTCGAACATCTGTGCGAGTGTCTGGGTG--
CCAAACCCTCGCGCGTAATGAAAGTGAACGTAGGTGAGAGCCCCC-------
CGGGGCGCATCATCGACCGATCCGGATG-
TCTTCGGATGGATTTGAGTAGGAGCACAGCTGTTCGGACCCGAAAGATGGTGA
ACTATGCGTGGATAGGGTGAAGCCAGAGGAAACTCTGGTGGAGGCTCGCAGCG
GTTCTGACGTGCAAATCGATCGTCGAATCTGCGCATGGGGGCGAAA--------------

GACTTATCGAACCATC---

TAGTAGCTGGTTACCGCCGAAGTTTCCCTCAGGATAGCAGTGTTGTGTCTTCAG
TTTTATGAGGTAAAGCGAATGATTAGGGACTCGGGGGCGCTCTTTAGCCTTCAT
CCATTCTCAAACTTTAAATATGTAAGAAGCCCTTGTTACTTCGCTGAACGTGGG
CATTCGAATGTATCAACACTAGTGGGCCATTTTTGGTAAGCAGAACTGGCGATG
CGGGATGAACCGAACGCGGGGTTAAGGTGCCGGAGTGGACGCTCATCAGACAC

CACAAAAGGTGTTAGTACATCTAGACAGT---

TGGACGGTGGCCATGGAAGTCGGAATCCGCTAAGGACTGTGTAACAACTCACC
A---

>H_lienhwacheense_MFLUCC_14_1231

CATCTCCTCAAAATGAGTGGCGGAGTCGCGGCGTGCTCTGAGCGTAGTAGA----
TGTCTTCTCGCTTTGGTAG--
CGCCCGCGGCAGCTAGCCGTTAACACACGATAGGGCTTACCCCTATCTCTTAGT
GGTTGACCTCGGATTAGGTAGGAATACCCGCTGAACTTAAGCATATCAATAAG
CGGAGGAAAAGAAACCAACAGGGATTGCCCTAGTAACGGCGAG--
TGAAGCGGCAACAGCTCAAA-TTTGAAATCTGGCCC---------
TCGCGGTCCGAGTTGTAATTTGTAGAGGATGCTTTCGGCGCGGC-
GCCTTCCGAGTTCCCTGGAACGGGACGCCAGAGAGGGTGAGAGCCCCGTACGG
TTGGA-
CGCCTAGCCTACACATAGCTCCTTCGACGAGTCGAGTAGTTTGGGAATGCTGCT
CTAAATGGGAGGTAAATTTCTTCTAAAGCTAAATACCGGCCAGAGACC-
GATAGCGCACAAGTAGAGTGATCGAAAGATGAAAAGCACTTTGAAAAGAGGG
TTAAATAGCACGTGAAATTGTTGAAAGGGAAGCG-
TTTGCGACCAGACCTATGCCCGGCGGATCATCCGGCG-TTT-
TCGCCGGTGCACTTCGCCGGGTCTCTAGGCCAGCATCGGTTCC---
CTTAGGGGGAGAAAGGCTCAGGGAACGTAGCTCCCCGTTTG--GGGAGTGTT-
ATAGCCCTCTGCGTAATACCCCT-CGGGGGACCGAGGAACGCGC-
TGTAATGGCAAGGATGCTGGCGTAATGGTCGTTAACGACCCGTCTTGAAACAC
GGACCAAGGAGTCGAACATTTGTGCGAGTGTTTGGGTG--
TCAAACCCTCACGCGTAATGAAAGTGAACGGAGGTGAGAGCCCCTCG-----
CGGGGCGCATCATCGACCGATCCTGATG-
TCTTCGGATGGATTTGAGTAAGAGCATAACTGTTCGGACCCGAAAGATGGTGA
ACTATGCGTGGATAGGGTGAAGCCAGAGGAAACTCTGGTGGAGGCTCGCAGCG
GTTCTGACGTGCAAATCGATCGTCAAATCTGCGCATGGGGGCGAAA---------------
----
GACTTATCGAACCATC---

TAGTAGCTGGTTACCGCCGAAGTTTCCCTCAGGATAGCAGTGTTGT-
TCTTCAGTTTTATGAGGTAAAGCGAATGATTAGGGACTCGGGGGCGCTATTTTG
CCTTCATCCATTCTCAAACTTTAAATATGTAAGAAGCCCTTGTTGCTTAGTTGA
ACGTGGGCATTCGAATGTACCAACACTAGTGGGCCATTTTTGGTAAGCAGAAC
TGGCGATGCGGGATGAACCGAACGCGGGGTTAAGGTGCCAGAGTGGACGCTCA
TCAGACACCACAAAAGGTGTTAGTACATCTTGACAGC-----------------------------------
----

AGGACGGTGGCCATGGAAGTCGGAATCCGCTAAGGACTGTGTAACAACTCACC
TGCCGAATGTACTAGCCCTGAAAATGGATGGCGCTCAAGCGTCT-
CACCCATACCTCGCCCTTAGGGTAGAACAGAGGCCCTAAGG-
AGTAGGCGGCCGTGG--GGA-TCAGTGACGA---

>Thamnomyces_dendroidea_CBS_123578

---ATTGCCCTAGTAACGGCGAG--
TGAAGCGGCAACAGCTCAAA-TTTGAAATCTGGCCC---------
TAGCGGTCCGAGTTGTACTTTGCAGAGGATGCTATGGGCGAGGC-
GCCTTCCGAGTTCCCTGGAACGGGACGCCAGAGAGGGTGAGAGCCCCGTACGG
TGGGA-
CGCGTCGCCTGCGTATAGCTCCTTCGACGAGTCGAGTAGTTTGGGAATGCTGCT
CTAAACGGGAGGTAAATTTCTTCTAAAGCTAAATACCGGCCAGAGACC-
GATAGCGCACAAGTAGAGTGATCGAAAGATGAAAAGCACTTTGAAAAGAGGG
TTAAACAGCACGTGAAATCGTTGAAAGGGAAGCG-
TTTGCGACCAGACGTCGCCTAGGGGGATCATCCGGCA-
GCCTCTGCCGGTGCACTTCCGCTAG--GCGAGGCCAGCGTCGGCTTC---
CTCAGGGGGATAAAGGCCCGGGACACGTATCTCC----TTC--GGGAGTGTT-
ATAGCCCCGGGCGTACTACCCCT-GAGGGGACCGAGGAACGCGT-TCT----
GCAAGGACGCTGGCGTAATGGTCGTCAATGACCCGTCTTGAAACACGGACCAA
GGAGTCGAACGTTTATGCGAGTGTACGGGTG--
TCAAACCCTGGCGCGTAATGAAGGTGAACGGAGGTGAGAGCCCTT-------

CGGGGTGCATCATCGACCGATCCTGATG-
TCTTCGGATGGATTTGAGTAAGAGCATATGCGTTCGGACCCGAAAGATGGTGA
ACTATGCGCGGATAGGGTGAAGCCAGAGGAAACTCTGGTGGAGGCTCGCAGCG
GTTCTGACGTGCAAATCGATCGTCAAATCTGCGCATGGGGGCGAAA--------------

GACTTATCGAACCATC--

TAGTAGCTGGTTACCGCCGAAGTTTCCCTCAGGATAGCAGTGTTG--
TCTTCAGTTTTATGAGGTAAAGCGAATGATTAGGGACTCGGGGGCGCTATACTG
CCTTCATCCATTCTCAAACTTTCAATATGTAAGAAGCCCCCGTTGCTTAGCTGA
ACGGGGGCCTTCGAATGTTGCAACACTAGTGGGCCATTTTTGGTAAGCAGAAC
TGGCGATGCGGGATGAACCGAACGTGGGGTTAAGGTGCCAGAGTAGACGCTCA
TCAGATACCACAAAAGGTGTTAGTACATCTTGACAGC--------------------------------
----

AGGACGGTGGCCATGGAAGTCGGAATCCGCTAAGGACTGTGTAACAACTCACC
TGCCGAATGTACTAGCCCTGAAAATGGATGGCGCTCAAGCGTCT-
CACCTATACCCCGCCCTTAGGGTAGAAACGAGGCCCTAAGG-
AGTAGGCGGCCGTGG--AGG-TCAGTGACGAAGCCTAGG-------------------------------
----

>H_fuscum_CBS_113049

CCC---------TCGTGGTCCGAGTTGTAATTTGTAGAGGATGCTTTTGGTGCGGT-
GCCTTCCGAGTTCCCTGGAACGGGACGCCAGAGAGGGTGAGAGCCCCGTACGG
TTGGA-
CACCTACCCTATATATAGCTCCTTCGACGAGTCGAGTAGTTTGGGAATGCTGCT
CTAAATGGGAGGTAAATTTCTTCTAAAGCTAAATACCGGCCAGAGACC-
GATAGCGCACAAGTAGAGTGATCGAAAGATGAAAAGCACTTTGAAAAGAGGG
TTAAATAGCACGTGAAATTGTTGAAAGGGAAGCG-
TTTGCGACCAGACCTTTTCCCGGCGGATCATCTGGTG-TTC-

TCACCGGTGCACTTCGCTTGG--TTTAGGCCAGCATCGGTTTC---
TTTAGGGGGATAAAGGCGTTGGGAACGTAGCTCT----TTC--GGGAGTGTT-
ATAGCCCTTCACGTAATACCCTT-CGAGGGACCGAGGACCGCGC-ATT----
GCAAGGATGCTGGCGTAATGGTCGTCAACGACCCGTCTTGAAACACGGACCAA
GGAGTCGAACATTTGTGCGAGTGTTTGGGTG--
TTAAACCCTCACGCGTAATGAAAGTGAACGGAGGTGAGAGCCCTT-------
ACGGGTGCATCATCGACCGATCCTGATG-
TCTTCGGATGGATTTGAGTAAGAGCATAACTGTTCGGACCCGAAAGATGGTGA
ACTATGCGTGGATAGGGTGAAGCCAGAGGAAACTCTGGTGGAGGCTCGCAGCG
GTTCTGACGTGCAAATCGATCGTCAAATCTGCGCATGGGGGCGAAAGTAAGTT
CCATACCTACCCTATATACAGCTACCCTGTAGCTACCCTATATATACATCTATA
ACGAGAATTT--AATGCTAACAAGTTTTCCCTTCAGGACTTATCGAACCATC-------

TAGTAGCTGGTTACCGCCGAAGTTTCCCTCAGGATAGCAGTGTTG--
TATTCAGTTTTATGAGGTAAAGCGAATGATTAGGGACTCGGGGGCGCTTTATTG
CCTTCATCCATTCTCAAACTTTAAATATGTAAGAAGCCCTTGTTGCTTAATTGA
ACGTGGGCATTCGAATGTATCAACACTAGTGGGCCATTTTTGGTAAGCAGAACT
GGCGATGCGGGATGAACCGAACGCGGGGTTAAGGTGCCAGAGTGGACGCTCAT
CAGACACCACAAAAGGTGTTAGTACATCTTGACAGC--------------------------------------
----

AGGACGGTGGCCATGGAAGTCGGAATCCGCTAAGGACTGTGTAACAACTCACC
TGCCGAATGTACTAGCCCTGAAAATGGATGGCGCTCAAGCGTCT-
CACCCATACCTCGCCCTTAGGGTAGAAACGATGCCCTAAGG-AGTAGGC----------

>H_addis_MUCL_52797

--GCCCTAGTAACGGCGAG--
TGAAGCGGCAACAGCTCAAA-TTTGAAATCTGGCCC---------

TMGCGGTCCGAGTTGTAATTTGTAGAGGATGCTTTTGGTGCGGT-
GCCTTCTGAGTTCCCTGGAACGGGACGCCAGAGAGGGTGAGAGCCCCGTACGG
TTGGM-
CACCTASCCTTTGTATAGCTCCTTCGACGAGTCGAGTAGTTTGGGAATGCTGCT
CTAAATGGGAGGTAAATTTCTTCTAAAGCTAAATACCGGCCAGAGACC-
GATAGCGCACAAGTAGAGTGATCGAAAGATGAAAAGCACTTTGAAAAGAGGG
TTAAATAGCACGTGAAATTGTTGAAAGGGAAGCG-
TTTRCGACCAGACCTTTTCCAGGCGGATCATCCGGTG-TTC-
TCACCGGTGCACTTCGTCGGG--TTTAGGCCAGCATCGGTTTT---
CGTAGGGGGATAAAAGCCCTGGGAACGTAGCTCT----TTC--GGGAGTGTT-
ATAGCCCTAAGCATAATACCCTT-ACGGGGACCGAGGACCGCGC-TTC---
GGCAAGGATGCTGGCATAATGGTCGTCAACGACCCGTCTTGAAACACGGACCA
AGGAGTCGAACATTTGTGCGAGTGTTTGGGTA--
TCAAACCCTCACGCGTAATGAAAGTGAACGGAGGTGAGAGCCCCT-------
CGGGGTGCATCATCGACCGATCCTGATG-
TCTTCGGATGGATTTGAGTAAGAGCATAACTGTTCGGACCCGAAAGATGGTGA
ACTATGCGTGGATAGGGTGAAGCCAGAGGAAACTCTGGTGGAGGCTCGCAGCG
GTTCTGACGTGCAAATCGATCGTCAAATCTGCGCATGGGGGCGAAAGTATGTA
TATCCCTT------------------CTATAATTCCCTTCAGGGAACG-
CAGGGAAGAAAATCC--
AATGCTAACAACAATCTCTTTTAGGACTTATCGAACCATC-----------------------------
----

TAGTAGCTGGTTACCGCCGAAGTTTCCCTCAGGATAGCAGTGTTG--
TTTTCAGTTTTATGAGGTAAAGCGAATGATTAGGGACTCGGGGGCGCTATATTG
CCTTCATCCATTCTCAAACTTTAAATATGTAAGAAGCCCTTGTTGCTTAATTGA
ACGTGGGCATTCGAATGTATCAACACTAGTGGGCCATTTTTGGTAAGCAGAACT
GGCGATGCGGGATGAACCGAACGCGGGGTTAAGGTGCCAGAGTGGACGCTCAT
CAGACACCACAAAAGGTGTTAGTACATCTAGACAGTTGGTATGTACTAAATCT
ATCCCCTATAA---

CATCCCCTAACTCGCATGCTA--
ATCTTTTACAGGACGGTGGCCATGGAAGTCGGAATCCGCTAAGGACTGTGTAA
CAACTCACCAACCGAATGTACTAGCCCTGAAAATGGATGGCGCTCAAGCGTCT-
CACCCATACCTCGCCCTTAGGGTAGAAACGATGCCCTAAGG-
AGTAGGCGGCCGTAG--AGG-
TCAGTGACGAAGCCTAGGGCGTGAGCCCGGGTCGAAC-GCCTCTAGTGC---------

>H_hainanense_FCATAS2712

TCCGGCG-TTC-TAGCCGGTGCACTTGACCCGG--GGTAGCCCAGCATCGGTTC----
CTTAGGGGGATAAAGGATCTGGGAACGTGGCTCC----TTC--GGGAGTGTT-ATA-
CCCTCAGCGTAAT-CCCTT-AGGGGGACCGAGGACCGCGC-TTC---
GGCAAGGATGCTGGCATAATGGTCGTCAACGACCCGTCTTGAAACACGGACCA
AGGAGTCGAACATTTGTGCGAGTGTTTGGGTG--
TCAAACCCTCACGCGTAATGAAAGTGAACGGAGGCGAGAGCCCT---------
AGGGCGCATCGTCGACCGATCCTGATG-
TCTTCGGATGGATTTGAGTAAGAGCATAACTGTTCGGACCCGAAAGATGGTGA
ACTATGCGTGGATAGGGTGAAGCCAGAGGAAACTCTGGTGGAGGCTCGCAGCG
GTTCTGACGTGCAAATCGATCGTCAAATCTGCGCATGGGGGCGAAA---------------
--GACTTATCGAA------

>H_rubiginosum_MUCL_52887

---GTAACGGCGAG--
TGAAGCGGCAACAGCTCAAA-TTTGAAATCTGGCCC---------
TAGCGGTCCGAATTGTAATTTGTAGAGGATGCTTTTGGTGCGGT-
GCCTTCCGAGTTCCCTGGAACGGGACGCCAGAGAGGGTGAGAGCCCCGTACGG
TTGGA-
CACCTACCCTATATATAGCTCCTTCGACGAGTCGAGTAGTTTGGGAATGCTGCT
CTAAATGGGAGGTAAATTTCTTCTAAAGCTAAATACCGGCCAGAGACC-
GATAGCGCACAAGTAGAGTGATCGAAAGATGAAAAGCACTTTGAAAAGAGGG
TTAAATAGCACGTGAAATTGTTGAAAGGGAAGCG-
TTTACGACCAGACCTCTTCCAGGCGGATCATCCGGTG-TTC-
TCACCGGTGCACTTCGTCTGG--TTTAGGCCAGCATCGGTTTT---
CGTAGGGGGATAAAGACCTGGGGAACGTATCTCC----TTC--GGGAGTGTT-
ATAGCCCCTCGTGTAATACCTCT-ACGGGGACCGAGGACCGCGC-ACT----
GCAAGGATGCTGGCGTAATGGTCGTCAACGACCCGTCTTGAAACACGGACCAA
GGAGTCGAACATTTGTGCGAGTGTTTGGGTA--
TTAAACCCTCACGCGTAATGAAAGTGAACGGAGGTGAGAGCCCTT-------
CGGGGTGCATCATCGACCGATCCTGATG-
TCTTCGGATGGATTTGAGTAAGAGCATAACTGTTCGGACCCGAAAGATGGTGA
ACTATGCGTGGATAGGGTGAAGCCAGAGGAAACTCTGGTGGANGCTCGCAGCG
GTTCTGACGTGCAAATCGATCGTCAAATCTGCGCATGGGGGCGAAAGTATGTA
TTATCCCG-------GGGTGTATACCCTATAATTCTCCCCTGGAGACA--------
ATAAACAG--TATGCTAACAACAA-CTTCTTTAGGACTTATCGAACCATC-----------
----

TAGTAGCTGGTTACCGCCGAAGTTTCCCTCAGGATAGCAGTGTTG--
TGTTCAGTTTTATGAGGTAAAGCGAATGATTAGGGACTCGGGGGCGCTTTATTG
CCTTCATCCATTCTCAAACTTTAAATATGTAAGAAGCCCTTGTTACTTAATTGA
ACGTGGGCATTCGAATGTATCAACACTAGTGGGCCATTTTTGGTAAGCAGAACT
GGCGATGCGGGATGAACCGAACGTGGGGTTAAGGTGCCAGAGTGGACGCTCAT
CAGACACCACAAAAGGTGTTAGTACATCTAGACAGTTGGTATGTACTAATCTTT
TCTTTTACCCCCTGACGGTCCTGAGGGTTTTGAGGGGATGGTCAGATGCAAACT
CTTAAAGGCAATAGTCGCTATAAGTTTAAAAGCTGGGGGATGATGTGTTGTTAT
CTCTCACTGCGTTTATTACTTGTACTTCTCTGTCTGGCGGTGTTATAAGTTTGAG
GGTTGGTAGCCCTAAGGGGCTACCCTACAAAAGATTCAA-----------------
AACCTTGTGTACACGCAGGGAGCTTTGGCATCACGGACCCCACACTTATAGCTT
GTTGTCGAAGAGTTTGCATCGTGCCTTTAGGCACCTTGTGGCCTGAACTGAAGG
ATCAATGGA------
CCTAAAGGTCTCTGTATTTTAACGAAACAAATCACTAATAACTTAAATAGGACG
GTGGCCATGGAAGTCGGAATCCGCTAAGGACTGTGTAACAACTCACCAACCGA
ATGTACTAGCCCTGAAAATGGATGGCGCTCAAGCGTCT-

CACCCATACCTCACCCTTAGGGTAGAAACGATGCCCTAAGG-
AGTAGGCGGCCGTAG--GGG-TCAGTGACGAAGC--

>D_crateriformis_GMBC0205

---CAACAGCTCAAA-
TTTGAAATCTGGCCCTCT----
TGTCGGGGTCCGAGTTGTAATTTGCAGAGGATGCTTTAGGCGCGGCGGCCTTCC
GAGTTCCCTGGAACGGGACGCCGTAGAGGGTGAGAGCCCCGTACGGTTGGA-
CGCTAAGCTTATATATAGCGCCTTCGACGAGTCGAGTAGTTTGGGAATGCTGCT
CTAAATGGGAGGTAAATTTCTTCTAAAGCTAAATACCGGCCAGAGACC-
GATAGCGCACAAGTAGAGTGATCGAAAGATGAAAAGTACTTTGAAAAGAGGG
TTAAACAGCACGTGAAATTGTTGAAAGGGAAGCG-
TTTGCGACCAGACCTTTTCCGGGCGGATCATCCGGCG-TTC-
TCGCCGGTGCACTCCGCCCGG--
TTTAGGCCAGCATCGGTTCCTGTTTCGGGGGGAGAAAGGCCGGGGGAAAGTAG
CTCC----TTC--GGGAGTGTT-ACAGCCCTCGGCGGAATGCCCTC-
GGGGGGACCGAGGACCGCGC-CTTC-
GGGCAAGGATGCTGGCGTAATGGTCGTCAACGACCCGTCTTGAAACACGGACC
AAGGAGTCGAACAGCTGTGCGAGTGTTCGGGTGGCTAAAGCCCTTGCGCGTAA
TGAAGGTGAACGGAGGTGAGAGCCTCCTGTAACGGGGGGTGCATCATCGACCG
ATCCTGATGTTCTTCGGATGGATTTGAGTAAGAGCATAGCTGTTCGGACCCGAA
AGATGGTGAACTATGCGTGGATAGGGTGAAGCCAGAGGAAACTCTGGTGGAG
GCTCGCAGCGGTTCTGACGTGCAAATCGATCGTCAAAT--------------------------------
----

>D_guizhouensis_GMBC0065

TTTGAAATCTGGCTCCCCCTCTTACCGGGGCCCGAGTTGTAATTTGCAGAGGAT
GCTTTGGGCGAGGCGGCCTTCCGAGTTCCCTGGAACGGGACGCCGTAGAGGGT
GAGAGCCCCGTACGGTTGGT-
CGCCGAGCTCGCGTATAGCGCCTTCGACGAGTCGAGTAGTTTGGGAATGCTGCT
CTAAATGGGAGGTAAATTTCTTCTAAAGCTAAATACCGGCCAGAGACC-
GATAGCGCACAAGTAGAGTGATCGAAAGATGAAAAGTACTTTGAAAAGAGGG
TTAAACAGCACGTGAAATTGTTGAAAGGGAAGCG-
TTTGCGACCAGACCTTTTCCGGGCGGATCATCCGGCG-TTC-
TCGCCGGTGCACTCCGCCCGG--TCTAGGCTAGCATCGGTTCCCG-
TCCGGGGGGAGAAAGGCCGGGGGAAAGTGGCTCC----TCC--GGGAGTGTT-
ACAGCCCTCGGCGGAATGCCCTC-GGGGGAACCGAGGACCGCGC-TTC---
GGCAAGGATGCTGGCGTAATGGTCGTCAACGACCCGTCTTGAAACACGGACCA
AGGAGTCGAACAGCTGTGCGAGTGTTCGGGTGACTAAAGCCCCGGCGCGTAAT
GAAGGTGAACGGAGGTGAGAGCCCCCTGTAGCGGGGGGCGCATCATCGACCG
ATCCTGATGTTCTTCGGACGGATTTGAGTAAGAGCACGGCTGTTCGGACCCGAA
AGATGGTGAACTATGCGTGGATAGGGTGAAGCCAGAGGAAACTCTGGTGGAG
GCTCGCAGCGGTTCTGACGTGCAAATCG---

>D_rogersii_GMBC0204

TGAAGCGGCAACAGCTCAAA-TTTGAAATCTGGCTCTCTTTT---
CGGGGGTCCGAGTTGTAATTTGCAGAGGATGCTTTGGGCGAGGCGGCCTTCCG
AGTTCCCTGGAACGGGACGCCGTAGAGGGTGAGAGCCCCGTACGGTTGGC-
CGCCGAGCTCGCGTATAGCGCCTTCGACGAGTCGAGTAGTTTGGGAATGCTGCT
CTAAATGGGAGGTAAATTTCTTCTAAAGCTAAATACCGGCCAGAGACC-
GATAGCGCACAAGTAGAGTGATCGAAAGATGAAAAGTACTTTGAAAAGAGGG
TTAAACAGCACGTGAAATTGTTGAAAGGGAAGCG-
TTTGCGACCAGACCTTCTCCGGGGGGATCATCCGGCG-TTT-
TCGCCGGTGCACTCCGCCTGG--TTTAGGCCAGCATCGGTTCCCG-
TCCGGGGGGAGAAAGGCTGGGGGAAAGTGGCTCC----TCC--GGGAGTGTT-
ACAGCCCCTAGCGGAATGCCCTC-GGGGGGACCGAGGACCGCGC-TTC---
GGCAAGGATGCTGGCGTAATGGTCGTCAACGACCCGTCTTGAAACACGGACCA
AGGAGTCGAACAGCTGTGCGAGTGTTCGGGTGGCTAAAGCCCCGGCGCGTAAT
GAAGGTGAACGGAGGTGAGAGCCCTCTGTAGCGGGGGGCGCATCATCGACCG
ATCCTGATGTTCTTCGGATGGATTTGAGTAGGAGCACGGCTGTTCGGACCCGAA
AGATGGTGAACTATGCGTGGATAGGGTGAAGCCAGAGGAAACTCTGGTGGAG
GCTCGCAGCGGTTCTGACGTGCAAAT---

Table S5. Alignment of the *rpb*2 sequences used in the phylogenetic study.

>A_annulatum_CBS_140775

ACCTCAAACGCTGCATCGACGGAAACAAGCGTTTCCAAATCGAACTTGCCGCCAAGCCCGCAATTATCACCA
ACGGCTTGAAGTACTCGCTCGCCACGGGTAACTGGGGCGATCAAAAGAAGGCGATGAGTTCGACTGCCGG
TGTATCGCAGGTCTTGAACCGCTACACTTTC-----TCCTCGAC-
GCTTTCTCACTTACGACGAACGAACACGCCCATCGGAAGAGATGGAAAGCTAGCCAAGCCGCGGCAGCTCC
ACAACACTCACTGGGGTCTCGTCTGCCCGGCGGAGACTCCCGAGGGCCAGGCTTGCGGCTTGGTGAAGAA
TTTATCCTTAATGTGTTCTATTAGCGTTGGAACATCGACAGATCCTATCGTGGACTACATGATAACTAGGAAT
ATGGAAGTCCTGGAGGAGTATGAACCGATGAGATATCCCAACGCCACCAAGATCTTCCTCAACGGCTCTTG
GATCGGTGTACACCAGGATCCCAAGACCCTCGTCAGGGACGTTCAGGCGCTTCGTCGAGCCAACCAGATTC
CTGCCGAGGTCTCGCTGGTTCGTGATATCCGAGACCGTGAGTTCAAGATCTTTTCTGACGCCGGTCGTGTGA
TGCGTCCCTTGTTTCGCGTACAGCAGGAAGAC--
ATTGCCGAGCAGGGTATTGAGAAGGGCACATTGGCTCTTACTAAGCAGATGATCAAACGCTTAGAAGCAGA
TGTCGATCTGGACCCGGAGAGCGAAGCATACTTTGGCTGGCAAGGTCTAGTCAACGAGGGCGTTATCGAG
TTCCTCGATGCGGAAGAAGAGGAGACTGCCATGATTTGCATGACTCCCGAAGATTTGGATACTTATCGTAT
GACCAAGCTTGGGTACGAGGTGTCCCAGGACAACG-GG---GACGAGGTG------
AACAAGCGACTCAAGACCAAGGTGAACCCGTCAACGCACATGTATACCCATTGCGAGATCCATCCC---------
----

>A_truncatum_CBS_140778

AAATATTGTTCGCCGACTAGTGCAAGAGATAACTCAGCATCTCAAGCGCTGCATCGACGGGAACAAACGTT
TCCAGATTGAACTTGCCGCCAAGCCCGCCATTATCACCAACGGCTTGAAGTACTCGCTCGCTACGGGTAACT
GGGGCGATCAAAAGAAGGCGATGAGCTCGACTGCCGGTGTATCGCAGGTCTTGAACCGCTACACTTTC-----
TCGTCAAC-
CCTTTCTCATTTACGACGAACGAACACGCCCATCGGAAGAGATGGAAAGCTAGCCAAGCCACGGCAGCTCC
ACAACACACACTGGGGTCTCGTCTGTCCGGCAGAGACTCCCGAAGGCCAGGCTTGTGGCTTGGTGAAGAAT
CTATCCTTGATGTGTTCTATCAGTGTTGGAACATCGACAGATCCTATCGTGGACTACATGATCACTAGGAAT
ATGGAAGTCCTGGAGGAGTATGAACCGATGAGATATCCCAACGCCACTAAGATCTTCCTCAACGGCTCCTG
GATCGGTGTACACCAGGACCCAAAGACCCTCGTCAGGGACGTCCAGGCACTTCGTCGAGCCAACCAGATAC
CTGCTGAGGTCTCGCTAGTTCGTGATATCCGAGACCGTGAATTCAAGATCTTTTCGGATGCCGGTCGTGTGA
TGCGCCCCCTGTTCCGCGTACAACAGGAAGAC--
ATCGCCGAGCAGGGCATCGAGAAGGGCACCTTGGCTCTTACCAAGCAGATGATCAAACGTCTAGAAGCAG
ATGTCGATCTGGACCCGGAGAGCGAGGCGTACTATGGCTGGCAAGGTCTAGTCAACGAGGGAGTTATCGA
GTTCCTCGATGCGGAAGAAGAGGAGACTGCGATGATTTGCATGACGCCGGAAGATTTGGACACCTACCGT
ATGACCAAGCTTGGGTATGAGGTGTCCCAGGACAACG-GA---GATGAGGTG------
AATAAGCGACTCAAGACTAAAATCAACCCGTCAACGCACATGTATACCCATTGCGAGATCCATCCCAGTATG
CTCCTGGGTATCTGCGCAAGCATCATTCCATTCCCAGATCATAACCAGGTA--------------------------TGTGAT----
-------GTGCGTAAAAATGTTGC--
TTTTTCGCTACTAAC---

--CA--

>J_cohaerens_CBS_119126

TTTAGTCACGGAGATTACCCAGCATCTCAAGCGTTGCATCGATGGGAACAAACGTTTCCAAATTGAACTAGC
TGCCAAACCCGCCATCATCACTAATGGCTTGAAATATTCTCTCGCCACAGGTAACTGGGGTGATCAGAAAAA
GGCGATGAGCTCGACGGCTGGTGTATCCCAAGTCTTGAACCGTTACACTTTC-----TCGTCAAC-
TCTTTCTCATTTGAGAAGAACGAACACACCTATCGGGAGAGATGGGAAACTCGCCAAGCCGCGGCAACTTC
ATAACACTCATTGGGGTCTGGTCTGTCCGGCCGAGACACCCGAAGGCCAAGCCTGCGGACTGGTGAAGAA
TCTGTCACTCATGTGTTCTATCAGCGTGGGAACATCGACTGATCCTATTGTGGACTATATGATTACTAGAAAT
ATGGAAGTCCTCGAGGAGTATGAACCGATGAGATATCCCAACGCTACCAAGATCTTTCTAAACGGCTCTTG
GATCGGTGTTCACCAGGATCCCAAGACCCTCGTTAGGGATGTACAAAACCTTCGCCGGAACAATCAAATTCC
TTCCGAAGTCTCGTTGGTTCGCGATATCCGCGATCGTGAGTTCAAGATCTTTTCAGATGCCGGCCGTGTGAT
GCGTCCATTGTTC---

>Rostrohypoxylon_terebratum_CBS_119137

TGTTTAGGAATATAATTCGTCGGCTGGTGGCTGAGACTACACAGCATCTCAAACGCTGCATTGACAGTAACA
AGCGTTTCCAAATCGAGCTTGCCGCTAAGCCAGGCATCATCACCAACGGTTTGAAGTACTCGCTCGCCACGG
GTAATTGGGGCGATCAGAAGAAGGCGATGAACTCGACCGCCGGCGTGTCACAGGTCTTGAACAGGTATAC
TTTC-----GCGTCAAC-
CCTTTCTCACTTGAGACGAACCAACACTCCCATCGGAAGAGACGGAAAGCTAGCTAAACCACGGCAGCTTC
ACAACACTCATTGGGGTCTGGTCTGTCCAGCAGAGACGCCCGAAGGCCAGGCTTGCGGATTGGTGAAGAA
CTTGTCCTTGATGTGTTCCATCAGTGTTGGTACATCGACAGATCCCATTGTCGACTACATGATAACCAGGAA
CATGGAAGTTCTTGAGGAGTATGAACCCATGAGATATCCCAACGCCACCAAGATCTTCCTCAACGGCTCTTG
GATCGGTGTTCATCAAGACCCTCAATCCCTCGTCAGAGATATTCAGACACTTCGCCGGGCCAACCAGATTCC
CGCTGAGGTATCGTTAATTCGTAATATTCGAGACCGTGAATTCAAGATCTTCTCGGATGCTGGCCGTGTGAT
GCGTCCCCTCTTCCGTGTACAACAGGAGACC--
GACGAGGCGCAGGGCATTGAGAAGGGCACATTAGCCCTTACCAAACAGATGATTAAGCGCCTAGAAGCGG
ATGTCGAGTTGGATCCAGAGAGTGAAGAATACTTCGGCTGGCAAGGTCTAGTCAACGAAGGTGTTATCGA
GTACCTCGACGCGGAGGAAGAGGAAACGGCCATGATTTGCATGACGCCTGAAGACTTGGACATTTATCGC
ATGA---

>D_bambusicola_CBS_122872

--TTGACCGATATACTTTC-----
TCTTCAAC-
CCTCTCTCATTTGAGGCGAACGAACACGCCTATCGGAAGAGATGGAAAACTCGCGAAGCCTCGACAGCTTC
ACAACACCCACTGGGGTCTGGTCTGTCCGGCCGAAACGCCCGAAGGCCAGGCTTGCGGGCTGGTAAAAAA
CCTATCGCTTATGTGCTCCATCAGCGTAGGTACGTCGACAGATCCCATCGTAGACTACATGATTACTAGGAA
TATGGAAGTCTTGGAGGAATATGAACCGATGCGGTACCCCAACGCCACCAAGATCTTCCTAAACGGATCCT
GGATCGGTGTTCATCAGGATCCTAAGTCTCTCGTGAGAGATGTGCAGCAGCTTCGTCGGGCCAACCAGATC
CCCTCCGAAGTCTCTCTGGTACGCGATATTCGTGATCGTGAGTTCAAGATCTTTTCAGATGCCGGCCGAGTC
ATGCGGCCCTTATTCGTGGTGCAGCAAGAGGAT--
GATCCCGAGGCTGGTACCACCAAGGGCTCGCTGGCTCTCACTAAGGAGATGATCCAAAGATTGGAGGCCA
GCGTCGATCTAGACCAGGAGAGCGAGGAGTACTTTGGCTGGCAAGGTCTTGTTAACGAAGGTGTTATCGA
GTACCTCGACGCGGAAGAAGAAGAAACGGCCATGATTTGCATGACACCCGAAGACTTGGAAACTTACCGC
ATGTCTAAATTGGGATATGACGTATCTCAGGATAACG-GC---GACGAGATC------
AACAAGCGACTCAAGACCAAGTTGAATCCCACGACGCATATGTATACGCATTGCGAGATCCATCCTAGTATG
CTCCTAGGTATTTGCGCGAGCATCATTCCTTTCCCCGACCATAATCAAGGTATGCATTATTCGC-------
AACGAATAAT-TTATATG------TTCCGTTGTAAAT---
AATTTCGTTGCTAAC--

---T--

>D_eschscholtzii_MUCL_45435

---ACACTTTT-----GCCTCGAC-
TCTTTCTCATTTAAGGCGAACGAATACGCCTATCGGAAGAGACGGAAAGCTTGCGAAACCTCGACAGCTGC
ACAATACCCATTGGGGTCTGGTCTGCCCGGCAGAAACGCCCGAAGGCCAGGCTTGCGGTTTGGTGAAGAA
CCTGTCGCTTATGTGCTCGATAAGTGTGGGCACGTCAACGGATCCCATCGTCGACTATATGATCACGAGAAA
TATGGAAGTGTTGGAGGAATACGAACCCATGCGCTATCCTAACGCCACCAAGATCTTCCTCAACGGATCTTG
GATTGGTGTGCACCAGGATCCCAAGTCTCTCGTGAGAGATGTGCAGCAGCTTCGCCGGGCCAACCAGATCC
CCTCCGAAGTATCTCTGGTTCGCGATATTCGTGATCGCGAGTTCAAGATTTTCTCGGATGCCGGTCGTGTCA
TGCGGCCCTTATTCGTGGTGCAGCAAGAAGAT---
GATCCCGAGGCTGGAACCACGAAGGGCTCGCTAGCTCTTACCAAGGAGATGATTCAGAGATTGGAGGCGA
GCGTCGATCTGGACCCAGAGAGCGAGGAGTACTTTGGTTGGCAAGGCCTAGTTAACGAAGGTGTTATCGA
GTACCTCGACGCGGAAGAAGAGGAGACGGCTATGATTTGCATGACACCCGAAGATTTGGAAACTTATCGG
TTGTCCAAACTCGGATATGACGTATCTCAGGATAACG-GA---GATGAGATT------
AACAAGCGTCTCAAGACTAAGTTGAATCCCACGACGCACATGTATACGCATTGCGAGATTCATCCTAGCATG
CTCCTGGGTATCTGCGCCAGCATCATTCCGTTCCCGGATCACAATCAGGTAT----------------------GTGTAAT---
ACTCG------CACTAATATCAAT--TTCTTGT------------

>D_placentiformis_MUCL_47603

--TTGACAGATACACTTTT-----
GCCTCGAC-
TCTTTCTCATTTAAGGCGAACGAATACGCCTATCGGAAGAGACGGAAAGCTTGCGAAACCTCGACAGCTGC
ACAATACCCATTGGGGTCTGGTCTGCCCGGCAGAAACGCCCGAAGGCCAGGCTTGCGGTTTGGTGAAGAA
CCTGTCGCTCATGTGCTCGATAAGTGTAGGCACGTCAACGGATCCCATCGTCGACTATATGATTACGAGAAA
TATGGAAGTGTTGGAGGAATACGAACCCATGCGCTATCCTAACGCTACCAAGATCTTCCTCAACGGATCTTG
GATCGGTGTGCACCAGGATCCCAAGTCTCTCGTGAGAGATGTGCAGCAGCTTCGCCGGGCCAACCAGATCC
CCTCCGAAGTATCTCTGGTCCGCGATATTCGTGATCGCGAGTTCAAGATTTTCTCGGATGCCGGTCGTGTCA
TGCGGCCCTTGTTCGTGGTGCAGCAAGAAGAC---
GATCCCGAGGCTGGAACCACGAAGGGCTCGCTAGCTCTTACCAAGGAGATGATTCAGAGATTGGAGGCGA
GCGTCGATCTAGACCCGGAGAGCGAGGAGTACTTTGGTTGGCAAGGCCTAGTTAACGAAGGTGTTATCGA
GTACCTCGACGCAGAAGAAGAGGAAACGGCTATGATTTGCATGACACCCGAAGATTTGGAAACTTATCGGA
TGTCCAAACTCGGATATGACGTATCTCAGGATAACG-GA---GATGAGATT------
AACAAGCGTCTCAAGACTAAGTTGAATCCCACGACGCACATGTATACGCATTGCGAGATTCATCCTAGCATG
CTCCTGGGTATCTGCGCCAGCATCATTCCGTTCCCGGATCACAATCAGGTA-------------------------TGTAAT---
ACTCG------CACTAATATTAAT--ATCTC----------------

>Rhopalostroma_angolense_CBS_126414

CATTTAAGGCGAACGAACACGCCTATTGGAAGAGACGGGAAGCTCGCGAAACCTCGACAGCTGCACAATA
CCCACTGGGGTCTGGTCTGTCCGGCCGAAACGCCCGAAGGCCAGGCTTGCGGTCTGGTGAAGAATCTGTCG
CTGATGTGCTCCATCAGCGTGGGTACCTCAACGGATCCCATCGTAGACTATATGATTACTAGGAATATGGAA

GTCTTGGAGGAATATGAGCCGATGCGATACCCTAACGCTACCAAGATCTTCCTCAACGGATCGTGGATCGG
CGTGCACCAGGATCCCAAGTCTCTCGTCCGAGACGTCCAGCAGCTTCGTCGGGCTAACCAAATCCCCTCCGA
AGTGTCCCTCGTTCGCGATATTCGTGATCGCGAGTTCAAGATCTTTTCGGACGCAGGCCGTGTCATGCGGCC
CTTGTTCGTGGTGCAGCAAGAGGAC--
GATCCCGATGCCGGTATCACGAAAGGGTCGCTGGCCCTTACCAAGGAGATGATCCAGAGGCTGGAGGCGA
GTGTCGATCTCGACCCGGAGAGCGAGGAGTACTTTGGTTGGCAAAGTCTTGTTAACGAGGGCGTTATCGAG
TACCTCGACGCGGAGGAGGAAGAAACGGCCATGATTTGTATGACACCCGAAGATTTGGAAACCTATCGGAT
GTCCAAGCTTGGGTACGACGTGTCTCAGGACAACG-GA---GATGAGATC------
AACAAGCGGCTCAAGACCAAGTTGAATCCCACGACGCACATGTACACGCATTGCGAGATCCATCCTAGCAT
GCTCCTGGGTATCTGCGCGAGCATCATCCCCTTCCCCGACCACAACCAGGTA----------------------------
CGTATTATTCGCCG------CGCTACTATCTATC---
GTTACCGTTGCTAAC--

--CCTGTCGCCTA---

--GT--------------------------

>Thamnomyces_dendroidea_CBS_123578

--ACTTTC-----TCGTCGAC-
TCTTTCCCATCTAAGGCGAACCAACACGCCTATCGGAAGAGACGGGAAGCTCGCGAAACCTCGACAGCTGC
ACAATACCCACTGGGGTCTTGTCTGTCCGGCCGAAACGCCCGAAGGCCAGGCCTGCGGTCTGGTGAAGAAC
CTATCGCTTATGTGCTCCATCAGCGTGGGTACCTCGACGGATCCTATCGTAGACTACATGATTACTAGGAAT
ATGGAAGTCTTAGAGGAATACGAGCCGATGCGATACCCTAACGCCACCAAGATCTTCCTCAACGGATCCTG
GATCGGTGTGCATCAGGATCCCAAGTCTCTCGTCAGAGATGTCCAGCAGCTTCGTCGGGCTAACCAAATCCC
CTCCGAAGTATCTCTCGTTCGTGATATCCGTGATCGCGAGTTCAAGATCTTTTCGGACGCCGGTCGTGTCAT
GCGGCCCTTGTTCGTGGTGCAGCAAGAGGAT--

GATCCCGAGGCTGGTATCACGAAGGGCTCGCTGGCTCTTACCAAGGAAATGATCCAGAGGTTGGAGGCGA
GTGTTGATGTCGACGCGGAGAGCGAAGAGTACTTTGGCTGGCAAAGTCTTGTCAACGCGGGTGTTATCGA
GTACCTCGACGCGGAGGAGGAAGAAACGGCCATGATTTGCATGACCCCCGAAGATTTAGAAACCTACCGG
ATGTCTAAACTCGGATATGATGTGTCTCAGGACAACG-GG---GACGAGATT------
AACAAGCGGCTCAAGACCAAGTTGAATCTTACGACGCACATGTACACGCATTGCGAGATCCATCCCAGCAT
GCTCCTGGGTATCTGCGCGAGCATCATCCCTTTCCCCGATCACAATCAGGTA----------------------TGTAAT-
TCATCCG------TACTAATATCAAT--
ATTCCGCTGCTAAC--

---TCTATAC---

>D_childiae_CBS_122881

GATCACAAATCACCTCAGACGTTGTATCGATTCAGGCAGGCGTTTCCAGATTGAGCTCGCCGCCAAGCCTGC
TATTGTAACCAACGGGCTGAAGTACTCTCTCGCCACAGGCAACTGGGGTGACCAGAAGAAGGCGATGAGC
TCGACAGCTGGTGTATCGCAAGTTCTGAACCGATACACGTTC-----GCCTCGAC-
TCTTTCTCATTTAAGGCGCACGAACACGCCTATCGGAAGAGACGGGAAACTCGCGAAGCCTCGACAACTTC
ACAATACCCATTGGGGCCTGGTCTGTCCGGCCGAAACGCCCGAAGGCCAAGCTTGTGGTTTGGTGAAGAAT
TTGTCGCTTATGTGCTCTATCAGCGTGGGTACGTCAACAGATCCTATCGTAGACTATATGATTACTAGGAAT
ATGGAAGTCTTGGAGGAATATGAACCGATGAGATACCCCAATGCCACCAAGATCTTTCTTAACGGATCTTG
GATCGGTGTGCACCAGGATCCCAAGTCTCTAGTCAGAGACGTTCAGCAACTTCGTCGGGCCAACCAGATCC
CCTCTGAAGTATCGCTGGTTCGCGACATCCGTGATCGCGAGTTCAAAATCTTCTCGGATGCTGGTCGTGTCA
TGCGGCCCTTATTTGTTGTGCAGCAAGAGGAT--
AATCCCGAGGCTGGTACTACGAAGGGCTCGCTAGCTATCAATAAGGAGATGATCGGGAGGCTGGAGGCGG
ATGTCGATGTAGACCCTGAAAGCGAGGGGTACTTTGGCTGGCAGGGTCTGGTCAACGAGGGTGTTATCGA

GTACCTCGATGCCGAAGAAGAAGAAACGGCTATGATTTGCATGACCCCTGAAGATTTGGAAACCTACCGGA
TGAGCAAGCTCGGATACGATGTGTCCCAGGATAATG-GA---GATGAGATT------
AATAAGCGACTCAAAACTAAGGTGAATCCTACAACGCACATGTATACGCATTGTGAGATTCATCCTAGCATG
CTCCTGGGTATCTGCGCGAGCATTATTCCCTTCCCAGACC--

>D_concentrica_CBS_113277

--TCTGACCGATACACGTTC-----
GCTTCGAC-
TCTTTCTCATCTAAGGCGGACGAACACGCCTATTGGAAGAGATGGAAAACTTGCGAAACCTCGACAGCTGC
ATAATACCCATTGGGGTCTGGTCTGTCCGGCCGAAACGCCCGAAGGCCAAGCTTGTGGGTTAGTGAAAAAT
CTGTCGCTTATGTGCTCTATCAGCGTGGGTACGTCAACGGATCCTATCGTAGACTATATGATTACTAGGAAT
ATGGAAGTCTTGGAGGAATACGAACCTATGCGATACCCTAATGCTACCAAGATCTTCCTCAACGGGTCTTGG
ATCGGTGTGCACCAGGATCCCAAGTCTCTAGTTAGAGACGTCCAGCAACTTCGTCGGGCTAACCAGATCCCC
TCTGAAGTGTCACTAGTTCGCGATATCCGTGATCGCGAGTTCAAGATCTTCTCAGATGCTGGTCGTGTCATG
CGACCCTTATTTGTTGTGCAGCAAGAGGAT--
AATCCCGAGGCTGGTACTACGAAGGGCTCGTTAGCTCTCAACAAGGAGATGATCCAGAGGCTGGAGGCAG
ATGTCGAGCTAGACCCCGAAAGCGAGGAATACTTTGGTTGGCAAGGCCTCGTTAACGAGGGGGTTATCGA
GTATCTCGACGCCGAAGAAGAAGAGACGGCTATGATTTGCATGACTCCTGAAGATTTAGAAACCTACCGGA
TGAGCAAGCTCGGATACGATGTATCGCAGGATAACG-GC---GATGAGATT------
AACAAGCGTCTAAAGACTAAAGTGAATCCTACGACGCACATGTATACACATTGCGAGATCCATCCCAGCAT
GCTCCTGGGTATCTGCGCAAGCATCATTCCCTTCCCGGACCACAATCAAGTA----------------------CGTAAT-

GCACTCA------CCTTAACCCTTGT--
GTTCTGTCACTAAC--

--C--

>D_dennisii_CBS_114741

---CTGACCGATACACGTTC-----
GCTTCGAC-
TCTTTCTCATCTAAGGCGGACGAACACGCCTATTGGAAGAGATGGAAAACTTGCGAAACCTCGACAGCTGC
ATAATACCCATTGGGGTCTGGTCTGTCCGGCCGAAACGCCCGAAGGCCAAGCTTGTGGGCTAGTGAAAAAT
CTATCGCTTATGTGCTCTATCAGCGTGGGTACGTCAACGGATCCTATCGTAGACTATATGATTACTAGGAAT
ATGGAAGTCTTGGAGGAATACGAACCTATGCGATACCCTAATGCCACCAAGATCTTCCTCAACGGATCTTGG
ATCGGTGTGCACCAGGATCCCAAGTCTCTAGTTAGAGACGTCCAGCAACTTCGTCGGGCTAACCAGATCCCC
TCTGAAGTGTCGCTAGTTCGCGATATCCGTGATCGCGAGTTCAAGATCTTCTCAGATGCTGGTCGTGTCATG
CGACCCTTATTTGTTGTGCAGCAAGAGGAT--
AATCCCGAGGCTGGTACTACGAAGGGCTCGTTGGCTCTCAACAAGGAGATGATCCAGAGGCTGGAGGCAG
ATGTCGAACTAGACCCTGAAAGCGAGGAATACTTTGGTTGGCAAGGCCTCGTTAACGAGGGGGTTATCGA
GTATCTCGACGCCGAAGAAGAAGAGACGGCTATGATTTGCATGACTCCTGAAGATTTAGAAACCTACCGGA
TGAGCAAGCTCGGATACGATGTGTCGCAGGATAACG-GC---GATGAGATT------
AACAAGCGTCTAAAGACTAAAGTGAATCCTACGACGCACATGTATACACATTGCGAGATCCATCCCAGCAT
GCTCCTGGGTATCTGCGCAAGCATCATTCCCTTCCCGGACCACAATCAGGTA-----------------------CGTAAT-
GCACTCA------CCTTAACCCTTGT--
GTTCTGTCGCTAAC--

--CTTCCGCGT---

>D_petriniae_MUCL_49214

TATTGGAAGAGACGGGAAACTCGCGAAGCCTCGACAACTTCACAATACCCATTGGGGTCTGGTCTGTCCGG
CCGAAACGCCCGAAGGCCAAGCTTGNGGTTTGGTGAAGAATTTGTCGCTCATGTGCTCTATCAGCGTGGGT
ACGTCAACGGATCCTATCGTAGACTATATGATTACTAGGAATATGGAAGTCTTGGAGGAATACGAACCCAT
GAGATACCCCAACGCCACCAAGATCTTCCTTAACGGATCTTGGATCGGTGTGCACCAGGATCCCAAGTCTCT
AGTTAGAGACGTCCAGCAACTTCGTCGGGCCAACCAGATCCCCTCCGAAGTGTCGCTGGTTCGCGATATCC
GTGATCGCGAGTTCAAGATCTTCTCGGATGCTGGTCGTGTCATGCGGCCCTTATTTGTTGTGCAGCAAGAGG
AT---
AATCCCGAGGCTGGTACTACGAAGGGCTCGCTAGCTCTCAATAAGGAGATGATCCAGAGGCTGGAGGCGG
ATGTCGACTTGGACCCTGAAAGCGATGAGTACTTTGGCTGGCAGGGCCTGGTCAACGAGGGTGTTATCGA
GTACCTCGATGCCGAGGAAGAAGAAACGGCTATGATCTGCATGACGCCTGAAGATCTAGAAACCTACCGG
ATGAGCAAGCTCGGATACGATGTGTCCCAGGATAATG-GA---GATGAGATT------
AATAAGCGGCTCAAGACTAAAGTGAATCCTACGACGCACATGTATACACATTGTGAGATTCATCCTAGCATG
CTCCTGGGTATCTGCGCAAGCATTATTCCCTTCCCGGACCACAATCAGGTA------------------------CGTAAT-
GCGCTCAT----CCCTTAACCCCTTT--
GTTATTTGTTATAT--

--CG---

>D_vernicosa_CBS_119316

--GACCGATACACGTTC-----
GCCTCGAC-
TCTTTCTCATTTAAGGCGTACGAACACGCCTATTGGAAGAGACGGGAAACTCGCAAAGCCTCGACAACTTCA
CAATACCCATTGGGGTCTGGTCTGTCCGGCCGAAACGCCCGAGGGCCAAGCTTGCGGGCTGGTAAAGAATT
TGTCGCTTATGTGCTCCATCAGCGTGGGTACGTCAACAGATCCTATCGTAGACTATATGATTACTAGGAATA
TGGAAGTCTTGGAGGAATACGAACCGATGAGATACCCTAATGCCACCAAGATCTTCCTTAACGGATCTTGG
ATCGGTGTGCACCAGGATCCCAAGTCTCTAGTTAGAGACGTCCAGCAACTTCGTCGGGCCAACCAGATCCC
CTCTGAAGTGTCGCTGGTTCGCGATATCCGTGATCGCGAATTCAAGATCTTCTCGGATGCTGGTCGTGTCAT
GCGGCCCTTATTTGTTGTGCAGCAAGAGGAT---
AATCCCGAGGCTGGTACTACGAAGGGCTCGCTAGCTCTCAGTAAAGAGATGATCCAGAGGCTAGAAGCAG
ATGTCGAACTAGACCCTGAAAGCGAGGAGTACTTTGGCTGGCAAGGTCTAGTCAACGAGGGTGTTATCGA
GTACCTCGATGCCGAGGAAGAAGAAACGGCCATGATTTGCATGACCCCCGAAGATTTAGAAACCTACCGGA
TGAGCAAGCTCGGATACGATGTGTCTCAGGATAATG-GA---GATGAGATT------
AATAAGCGGCTCAAGACTAAGGTCAATCCTACGACGCACATGTATACGCATTGTGAGATCCATCCCAGTATG
CTCCTGGGTATCTGCGCGAGTATTATTCCCTTCCCGGACCACAATCAGGTA-----------------------CGTGAT-
GCGCTTAT----CCTCTGACCCCTTC--GTTATATC-----

>Ruwenzoria_pseudoannulata_MUCL_51394

CCAGAAGAAAGCGATGAGCTCCACGGCTGGTGTGTCACAGGTTTTGAACCGATACACTTTC-----
GCCTCGAC-
CCTCTCCCATCTAAGGCGTACCAACACGCCTATCGGAAGAGATGGAAAGCTCGCAAAGCCTCGACAGCTGC
ACAACACACATTGGGGTTTGGTCTGTCCGGCCGAAACGCCCGAAGNCCAAGCCTGTGGGCTGGTGAAGAA
CTTGTCGCTGATGTGCTCCATCAGTGTTGGTACCTCAACGGATCCTATCGTAGACTATATGATTACTAGGAAT
ATGGAAGTCTTGGAGGAATACGAACCCATGAGGTACCCTAACGCCACCAAGATCTTCCTTAACGGATCTTG
GATCGGTGTGCACCAGGATCCCAAGTCTCTGGTGAGAGATGTCCAGCAGCTTCGTCGGGCTAACCAGATCC
CCTCGGAAGTGTCGCTGGTTCGCGATATCCGTGATCGTGAGTTCAAGATCTTCTCGGATGCCGGTCGTGTTA
TGCGGCCCTTATTTGTGGTGCAGCAAGAGGAT--
AATATCGAGGCCGGCACTTCAAAGGGCACGCTTGCTCTTAATAAAGAGATGATCCAGAGGCTAGAGGCTGA
TGTCGACTTGGATCCGGAGAGTGAGGAATATTTTGGTTGGCAAGGCCTAGTCAACGAGGGTGTTATTGAGT
ACCTCGATGCGGAGGAAGAAGAAACCGCTATGATTTGCATGACACCCGAAGATTTGGAAACTTATCGGATG
TCCAAACTCGGATATGATGTGTCCCAGGACAATG-GA---GACGAGATT------
AACAAGCGGCTTAAGACTAAGGTGAATCCCACAACGCACATGTATACGCATTGCGAGATTCATCCTAGTAT
GCTCCTGGGTATCTGCGCGAGCATCATTCCCTTCCCAGACCACAACCAGGTA-------------------------TGTAAT-
GCCCG--------CCTTACAATTTTT--A---------------------

>H_hainanense_FCATAS2712

CGGGGTATTGTTCGCCGATTGACGCAGGAGATCGTACAGCACCTCAAACGCTGCATCGACCAGAATCGACG
TTTCCAGATCGAGTTGGCTGCCAAGCCTGCCATCATTACCAACGGCCTCAAGTACTCTCTCGCCACGGGCAA
CTGGGGCGACCAGAAGAAGGCGATGAGCTCGACCGCCGGCGTGTCGCAGGTCCTGAACCGATACACGTTT-
----GCGTCGAC-
TCTCTCCCATCTGAGACGGACGAACACGCCCATCGGAAGGGACGGAAAACTTGCGAAGCCCCGGCAGCTG
CACAATACCCATTGGGGCCTGGTCTGTCCGGCCGAGACGCCCGAAGGTCAGGCTTGTGGGCTGGTGAAGA
ATTTGTCGCTGATGTGTTCCATCAGCGTGGGTACGTCGACGGATCCCATCGTAGACTACATGATCACTAGGA
ATATGGAAGTCTTGGAGGAATACGAACCCATGCGGTACCCCAACGCCACCAAGATCTTTCTCAACGGTTCTT
GGATCGGTGTGCACCAAGATCCCAAATCCCTGGTTAGAGATGTTCAGCAGCTCCGTCGGGCTAACCAGATC
CCCTCTGAGGTGTCCCTGGTTCGCGACATCCGCGATCGCGAGTTCAAGATCTTCTCGGACGCCGGCCGTGTT
ATGCGGCCCTTATTTGTCGTGCATCAGGAGGAT--
AACCCGGACGCCGGCACTACGAAGGGCTCGCTGACACTCAACAAGGAGATGATCCAGAGGTTGGAGGCGG
ACGTCGACCTGGATCCCGAAAGCGACGAATACTTTGGCTGGCAAGGCCTGGTTAACGAGGGCGCTATCGA
ATACCTCGACGCAGAAGAGGAGGAGACGGCCATGATCTGCATGACCCCCGAAGACCTAGAGAACTACCGC
TTAACCAAGCTTGGACAAGATATGCCCCAGGACAACG-GA---GACGAGATT------
AACAAGCGTCTCAAAACGAAGGTCAACCCCACGACGCATATGTACACGCATTGCGAGATCCATCCCAGCAT
GCTCTTAGGCATCTGCGCGAGCATCATCCCCTTCCCGGATCACAATCAGGTA-----------------------TGCGTT--
-----------CACTTAAGTTTCCT--TCCCTC---------------

>H_hinnuleum_MUCL_3621

CTTCCAGATTGAGCTTGCCGCCAAGCCCGCCATCATCACCAATGGGCTGAAGTATTCCCTTGCCACGGGTAA
CTGGGGTGATCAGAAGAAGGCAATGAGCTCGACCGCCGGTGTGTCGCAGGTGTTGAATCGATACACCTTC-
----GCCTCGAC-
CCTCTCTCACTTGAGACGAACGAACACTCCCATTGGAAGAGATGGAAAACTCGCGAAGCCCCGACAGCTTC
ACAATACCCACTGGGGCCTTGTCTGTCCGGCTGAGACGCCTGAAGGTCAGGCCTGTGGGCTTGTGAAGAAC
TTGTCGCTGATGTGTTCCATCAGCGTGGGAACATCGACGGATCCTATCGTGGACTATATGATTACTAGGAAT
ATGGAAGTCTTGGAGGAATATGAACCCATGCGATACCCCAACGCTACTAAGATCTTCCTCAATGGTTCTTGG
ATCGGTGTACATCAGGATCCCAAGACGCTCGTCAGAGATATCCAGGCACTTCGTCGAGCTAATCAGATTCCC
TCTGAAGTTTCCTTGGTCCGCGATATCCGTGATCGAGAGTTCAAGATCTTCTCAGATGCAGGTCGTGTCATG
CGCCCCTTGTTTGTCGTAAATCAAGAGGAT---
GTCCCCGAGCAGGGCATTGCTAAGGGTACACTGGCTCTTACCAAAGACATGATCCAACGACTAGAGGCGGA
CGTTGATCTTGATCCTGAAAGCGAGGAATATTATGGCTGGCAAGGCCTGGTCAACGACGGAGTTATCGAGT
TCCTCGATGCGGAAGAAGAGGAAACGGCCATGATTTGTATGACGCCGGAAGATCTGGAAATATACCGCAT
GACTAAGGCTGGGTTCGACGTGATCCAGGACAACA-AA---GATGAGGTT------
AACAAACGACTCAAGACCAAGGTGAACCCGTCCACGCACATGTATACACATTGCGAAATCCACCCC----------
----

>H_pulicicidum_CBS_122622

CCGACGTTTCCAAATCGAGCTTGCCGCCAAGCCCGCCATCATCACCAACGGTCTGAAGTACTCCCTTGCCAC
AGGTAACTGGGGTGACCAGAAGAAGGCGATGAGCTCTACTGCTGGCGTGTCGCAGGTCTTGAACAGATAT
ACCTTC-----GCGTCAAC-
TCTGTCTCACTTGAGGCGAACCAACACACCCATTGGAAGAGACGGGAAGCTTGCGAAGCCTCGACAGCTTC
ACAATACCCATTGGGGTCTCGTCTGTCCGGCTGAGACACCCGAAGGACAGGCCTGCGGGCTGGTGAAGAA
TTTAGCGCTGATGTGCTCTATCAGTGTGGGTACATCGACGGATCCTATTGTAGACTATATGATTACGAGGAA
TATGGAAGTCTTGGAGGAATACGAACCGATGCGATACCCCAACGCCACCAAGATCTTCCTCAATGGCTCTTG
GATCGGTGTACATCAAGATCCCAAGACTCTTGTTAGAGATATCCAGGCCCTTCGTCGAGCCAATCAGATCCC
CTCTGAAGTTTCTCTGGTCCGCGATATCCGTGATCGTGAGTTCAAGATCTTCTCAGATGCGGGTCGTGTCAT
GCGCCCCTTGTTTGTCGTACAACAAGAGGAT---
ATACCCGACCAGAATGTTACTAAGGGTACATTGGCTCTTACCAAAGAGATGATCCAGCGGCTGGAGGCGGA
TGTTGATCTGGATCCTGAGAGCGAGGAGTACTTCGGCTGGCAAGGTCTGGTTAACGAGGGTGTTATCGAGT
TTCTCGACGCGGAAGAAGAGGAAACGGCTATGATTTGCATGACGCCGGAAGATTTGGAAACTTATCGAAT
GACCAAGGCCGGTCTTGAAGTGGACCAGGACAACG-GA---GATGAAGTT------
AACAAACGGCTCAAGACTAAGGTGAACCCGTCGACACACATGTACACGCATTGCGAAATCCACCCCAGTAT
GCTCCTAGGTATCTGCGCCAGC--

>H_lateripigmentum_MUCL_53304

CACACAGCACCTTAAACGGTGTATCGACTCGAACAGACGTTTCCAGATTGAGCTAGCAGCCAAACCCGCTAT
CATCACAAATGGATTGAAGTACTCTCTTGCCACAGGTAACTGGGGTGATCAGAAGAAGGCGGCGAGCTCG
ACTGCCGGTGTGTCACAGGTCTTGAACCGTTACACTTTC-----GCATCGAC-
TCTATCTCACTTGAGGCGAACAAACACTCCTATCGGGAGAGACGGCAAGCTTGCTAAGCCTCGACAACTTCA
CAACACTCATTGGGGTCTGGTCTGTCCGGCTGAGACGCCTGAAGGCCAGGCTTGTGGATTGGTGAAGAACT
TGTCGCTCATGTGTTCCATCAGCGTGGGTACATCAACGGATCCTATCGTGGACTACATGATTACGAGAAACA
TGGAAGTTTTGGAGGAATATGAACCCATGCGATACCCTAACGCTACCAAGATCTTCCTCAATGGTTCTTGGA
TCGGTGTACATCAGGATCCCAAGACTCTCGTCAGAGATATCCAGATGCTTCGTCGGGCCAACCAAATTCCCT
CTGAAGTTTCTTTGGTCCGCGATATCCGTGATCGTGAGTTCAAGATCTTCTCGGATGCCGGTCGTGTCATGC
GTCCCTTGTTCGTCGTCCACCAAGAGGAT--A---
CTGAGCAAGGCGCTGCTAAGGGAACATTGGCCCTTACCAAAGACATGATCCAGCGACTAGAGGCGGACGT
CGATCTAGATCCCGACAGCGAAGAGTACTTCGGCTGGCAAGGCCTGGTCAACGAGGGTGTAATTGAGTTCC
TCGATGCGGAGGAAGAGGAGACGGCTATGATTTGCATGACACCAGAAGATTTGGAAGTCTACCGCGCGGC
CAAGCTTGGGTATGATGTGGTTCAGGATAACG-GT---GATGAGATT------
AATAAACGACTCAAGACGAAGATAAACCCCACGACGCACATGTACACGCATTGCGAGATTCATCCCAGCAT
GCTTCTCGGCATCTGCGCCAGC--

>H_olivaceopigmentum_DSM_107924

ACGTTGTATTGAGACGAATAGACGCTTCCAGATTGAGCTTGCCGCCAAGCCTGCCATCATCACTAACGGCTT
GAAATACTCCCTTGCCACCGGTAACTGGGGCGACCAGAAGAAGGCAATGAGCTCGACTGCCGGTGTGTCA
CAGGTTCTGAACCGATACACCTTC-----TCGTCGAC-
TCTCTCTCACTTGCGACGGACAAACACCCCCATTGGAAGGGATGGAAAGCTGGCGAAGCCCCGACAGCTTC
ATAATACTCATTGGGGTCTGGTCTGTCCGGCTGAGACGCCTGAAGGCCAGGCCTGCGGGTTGGTGAAGAA
CTTGTCGCTGATGTGCTCCATCAGCGTGGGTACATCGACGGATCCTATCGTGGACTATATGATTACTAGGAA
TATGGAAGTCCTGGAGGAATACGAACCGATGCGATACCCTAACGCTACCAAGATCTTCCTCAATGGCTCTTG
GATTGGTGTGCATCAAGATCCTAAGACTCTCGTCAGAGATATCCAAGCACTTCGACGCGCCAACCAGATCCC
TTCGGAGGTGTCGTTGATTCGAGATATTCGTGACCGAGAGTTTAAGATCTTCTCGGATGCGGGTCGTGTGA
TGCGGCCTTTGTTTGTCGTACAGCAGGAGGAT--
AACCCGGAGAAAGGCGTTACTAAGGGCACATTGGCTTTGACCAAAGAGATGATACAGCGACTAGAGGCTG
ATGTTGATCTGGACCCGGAGAGCGAGGAGTACTTCGGCTGGCAAGGCCTCGTCAACGAGGGAGTTATCGA
ATTCCTTGATGCCGAGGAAGAGGAAACGGCCATGATTTGCATGACCCCCGAAGACTTGGAGACCTTTCGTG
CGACCAAGGCCGGGGAGCCAGTTGCTCAGGACAACG-GG---GATGAAGTC------
AACAAGCGACTTAAGACCAAGGTGAACCCATCGACGCACATGTATACGCATTGCGAAATTCATCCGAGTAT
GCTCCTCGGCATCTGCGCCAGTATC--

>H_invadens_MUCL_51475

TTCGTCGTTTAGTTCAGGAGATTACGAATCACCTCAAGCGCTGTATCGATCAGAATAGACGTTTCCAGATTG

AGCTCGCAGCCAAGCCCTCCATCGTCACCAACGGGCTAAAGTATTCCCTGGCTACAGGTAACTGGGGCGAC
CAGAAAAAGGCGATGAGCTCAACCGCCGGTGTGTCACAGGTGTTGAACCGATATACATTC-----GCATCAAC-
CTTGTCACATTTGAGGCGAACCAACACCCCCATCGGAAGAGACGGGAAACTAGCGAAGCCTCGACAACTTC
ACAACACCCATTGGGGTCTGGTCTGTCCGGCCGAAACGCCCGAGGGTCAGGCCTGTGGGTTGGTGAAGAA
TCTGTCGCTGATGTGTTCTATCAGCGTGGGTACATCGACGGATCCTATCGTGGACTATATGATTACTAGGAA
TATGGAAGTCCTTGAGGAATATGAACCGATGCGATACCCCAACGCCACCAAGATTTTCCTGAATGGTTCTTG
GATTGGTGTACATCAGGACCCCAAGTCTCTGGTTAGAGATGTTCAGCAGCTTCGCCGGGCTAACCAGATCC
CCTCAGAAGTATCGTTGGTCCGCGATATTCGTGATCGCGAGTTCAAGATCTTCTCAGATGCCGGTCGTGTTA
TGCGCCCCTTGTTTGTCGTGCAACAAGATGAC--
AATCCGGAAGCCGGTATCATCAAAGGCACATTGGCTCTCAACAAGGACATGATCCAACGGCTAGAGGCCGA
TGTTGACTTAGATCCCGACAGTGATGAGTACTTTGGCTGGCAGGGTCTGGTTAATGAGGGTGTTATCGAGT
ACCTTGATGCCGAGGAAGAGGAAACGGCTATGATATGCATGACGCCTGAAGACTTGGAAAATTATCGTTTG
ACCAAGGCTGGTGTCGAGGTGCCCCAGGATAATG-GG---GATGAGGTT------
AACAAACGACTGAAGACCAAGGTGAATCCTTCGACACATATGTATACGCATTGCGAGATCCACCCTAGCAT
GCTCCTGGGTATTTGCGCCAGTATCATTCCCTTCCCGGATCACAATCAAGTA------------------------TGTAAT-
CCACGTGAAATTCTCTGAATGTTTAC--
CGCTGAC---

---CTGT--

>H_macrocarpum_CBS_119012

TGTTGTCCCTTT--
TAGCCAAACTGTTTCGAAATATCGTTCGTCGTTTGGTTCAGGAGATTACGAATCACCTTAAGCGCTGCATCG
ATCAGAATAGACGTTTCCAGATTGAACTCGCAGCCAAGCCCTCCATCGTCACCAACGGGCTGAAGTATTCCT
TGGCCACAGGCAACTGGGGCGACCAGAAAAAGGCGATGAGCTCAACCGCCGGTGTATCACAGGTGTTGAA
CCGATATACATTC-----GCATCAAC-

CTTGTCGCATTTAAGGCGAACCAACACCCCCATCGGAAGAGACGGGAAGCTAGCGAAGCCTCGACAACTCC
ACAACACCCATTGGGGTCTGGTCTGCCCGGCCGAAACGCCCGAGGGTCAGGCCTGTGGGTTGGTGAAGAA
TTTGTCGCTGATGTGTTCTATCAGCGTGGGTACATCGACGGATCCTATCGTGGACTATATGATTACTAGGAA
TATGGAAGTCCTTGAGGAATATGAACCGATGCGATACCCCAACGCGACCAAGATTTTCCTGAATGGTTCTTG
GATTGGCGTACATCAGGACCCCAAGTCTCTAGTTAGAGATGTTCAGCAGCTTCGCCGGGCTAACCAAATCCC
CTCGGAAGTATCGTTGGTCCGCGATATTCGTGATCGCGAGTTCAAGATCTTCTCAGATGCCGGTCGTGTTAT
GCGCCCCTTGTTTGTCGTGCAACAAGATGAC---
AATCCGGAAGCCGGTATCATGAAGGGCACATTGGCTCTCAACAAGGACATGATCCAACGGCTAGAGGCCG
ATGTTGACTTAGATCCCGACAGTGATGAGTACTTTGGCTGGCAGGGTCTAGTCAATGAGGGTGTTATCGAG
TATCTTGATGCCGAGGAAGAGGAAACGGCTATGATATGCATGACGCCTGAAGACTTGGAAAATTATCGTTT
GACCAAGGCTGGTGTCGAGGTGCCCCAGGATAATGNGG---GATGAGGTT------
AACAAACGACTGAAGACCAAGGTGAATCCTTCGACACATATGTATACGCATTGCGAGATCCACCCTAGCAT
GCTTCTGGGAATTTGCGCCAGTATCATTCCCTTCCCGGATCACAATCAAGTA------------------------TGTAAT-
CCACGTGGAATCCTCTGAATGTTTAC--
CGCTAAC---

---CTTACTC--TA--

--GTCGCAGAAA-------------------------

>H_lechatii_MUCL_54609

CTATTCCGAAATATTGTTCGTCGATTAGTCCAGGAGATCACTGGTAATCTTAGGCGATGTATCGATCAGAAC
AAGCGCTTCCAAATCGAGCTGGCTGCTAAGCCTAGTATAATCACGAATGGTCTAAAGTACTCTCTTGCTACG
GGTAACTGGGGTGACCAGAAGAAGGCAATGAGTTCGACGGCTGGCGTGTCGCAGGTCCTGAATCGATACA
CGTTT-----GCGTCTAC-
TCTCTCTCACTTGAGACGGACAAATACACCTATTGGCAGAGATGGGAAGCTTGCGAAGCCCCGGCAGCTGC

ATAATACCCACTGGGGTCTGGTATGTCCTGCTGAGACGCCTGAAGGCCAAGCCTGCGGGTTGGTAAAGAAT
TTGTCTCTCATGTGCTCTATTAGCGTGGGTACATCGACGGATCCCATCGTAGACTACATGATTACTAGAAAT
ATGGAAGTCTTGGAGGAATACGAACCGATGCGCTACCCCAACGCTACTAAGATCTTTCTCAATGGTTCTTGG
ATTGGTGTACACCAGGACCCCAAGGCTCTGGTTCGAGACGTGCAGCAACTTCGCCGGGCTAACCAGATCCC
CTTCGAAGTATCGTTAGTTCGCGACATTCGTGATCGTGAATTCAAGATCTTCTCGGATGCTGGCCGCGTTAT
GCGGCCCTTGTTCGTTGTACAGCAAGAGGAT---
AATCCCACGACCGGTGCTCCTAAAGGCTCACTAGCCCTTAACAAGGACATGATCCAGAGACTGGAGGCCGA
TGTCGATCTGGACCCCGAGAGTGAGGAGTACTTCGGCTGGCAGGGCCTAGTTAACGAAGGTGCCATCGAA
TATCTCGATGCGGAAGAAGAGGAAACAGCTATGATTTGCATGACTCCTGAGGACTTGGAAAACTACCGGAT
GACCAAACTTGGAATTGATATGTCTCAAGATAATG-GA---GACGAGATT------
AACAAACGGCTCAAAACCAAGGTTAATCCTACGACGCACATGTATACACATTGCGAGATTCATCCCAGTATG
CTCCTGGGTATCTGCGCGAGTATTATTCCATTTCCCGACCATAATCAGGTA--------------------------AGCAAT-
TCACCCTC-GTCCCTTGAATACTTTGC---
CCGCTAAT---

--CTTGCCCTGCA--

---GTCCCT-------------------------------

>H_fragiforme_MUCL_51264

aTGGCCGATTACGATGAGGGTTACGAGTATGAGGAGGAGTATGCCGATGA---
TTCCATTACGCCGGAAGATTGCTGGACTGTCATTGGTTCTTATTTCAAGAGAAAGGGTTTAGTGTCTCAACA
GATCGACTCTTTCAATGACTTTCAAGAGACCACCATTCAAGAATTAGTCGAAGAGTACTCCCAAATCAGCGT
CGATGAGCACAACCCTCCTTCCCCCGAAGGCCGCACCATCGCCCTTCGTCGATATGATCTCAAACTTGGACA
TGTCACCATTGCTCGGCCCGTCGCTATGGAGAGTGACAATACATCCGGTCCACTTCTACCCTACGAGTGCCG
CGATCGTAACATGACATATGCTGCTCCTATCTATGTTAAGGTCGATAGTAAGGTCACCGCATGTATCGAACA
AGACATCCCCCTGCACGAAATGGACGAAGAACAGCAGGCTGAAGCACGCGCTACCGGCAAGCACCCAGTA
CGCCTAGTTTGGGAAGAAGAAGAGAACGTTCTGGAGTCGCCTCTTACCGAGAAGGGCAAGACAAGTGACC
AAGTTTTCATCGGCAAACTTCCCATTATGATCAAGTCCAAGGCTTGCCATCTCAACGGCGAGAGCGAGGAC
GATCTTTTCATGTTGAACGAGTGCCCATATGATCAAGGTGGATATTTCATCATCAACGGTAGCGAAAAGGTT
CTTATTGCTCAAGAAAGATCGGCTGCGAACATTGTTCAGGTTTTCAAGAAGGCCCAGCCTAGCCCGTTCTCT
TACATCGCTGAGATCCGAAGTGCTCTCGAAAAAGGATCGAGACTCATCTCATCACTAATGTTGAAGCTGTAT
ACCAAAGGCGATGCATCAAGGGGCGGCTATGGCCAAACTATTCATACGACCCTCCCATTCGTGCGAGCTGA
CACTCCTATTGCGATTGTCTTCCGCGCTCTGGGCGTGGTCAGCGATGAAGATATTTTAAATTACATCTGTTAC
GACCGCAACGATAGCCAAATGCTCGAGATGTTACGTCCCTGCATCGAAGAAGCCTTTTGCATCCAGGACCG
TGAGGTTGCTCTAGATTTCATCGGGAAACGTGGAAGGGACTCTCACAATACCACGGCGCGAGAGCGTCGT
GTGCGGGCTGCTAAGGATATCTTGCAGAAAGAAATGCTTCCTCATATATCCCAGTCGGAAGGATGCGAGAC

CAGGAAGGCGTTCTTCCTCGGTTATATGGTTCACAAGCTATTGCAATGCGCCCTGGGCCGCCGCGATGTCG
ATGATCGGGATCACTTCGGAAAAAAGCGTCTCGACCTAGCTGGTCCCTTGCTTGCCAAGTTGTTCAGGAATA
TTGTTCGTCGATTGGTAACGGAGATTTCGCAGCATTTGAGACGTTGTATCGACCAAAACAGACGCTTCCAAA
TCGAGCTTGCCGCAAAGCCTGCGATTATTACAAATGGACTCAAATACTCGCTCGCTACAGGAAACTGGGGT
GATCAGAAGAAGGCCATGAGTTCGACCGCCGGTGTGTCTCAAGTCTTGAACCGATACACGTTC-----
GCATCGAC-
CCTTTCTCATTTGAGAAGAACCAACACTCCTATTGGAAGAGACGGAAAATTGGCGAAGCCTCGGCAGCTTC
ACAACACGCATTGGGGCCTTGTCTGTCCAGCCGAGACGCCCGAAGGCCAAGCCTGTGGACTGGTCAAAAAT
CTGTCGTTGATGTGCTCTATCAGTGTGGGTACTTCGACTGATCCTATCGTAGACTATATGATCACTCGAAGC
ATGGATGTTTTAGAAGAGTACGAGCCCAAGACCAATCCTAATGCCACGAAGATCTTCTTGAACGGCTCTTG
GATTGGCACGCACACAGACCCCAAGGCTCTCGTCAGAGATATTCAGGAATTACGACGAGCCAACCAGATTC
CGTCTGAGGTCTCACTGGTGCGCGACATTCGCGATCGTGAGTTCAAGATCTTCTCTGATGCTGGTCGTGTTA
TGCGCCCACTATTCGTCGTTCAACGAGAGGAT---
GATCCGGAGAAGGGTATTGTCAAGGGCTCCTTGGCTCTTACCAAGGAGATGATACAGAGGCTGGAGGCCG
ACAATGATCAAGATCCTGAGAGTGAGGATTATTTTGGCTGGCAAGGACTAGTTAATGACGGTGCTATTGAA
TACCTCGACGCCGAAGAAGAGGAGACGGCGATGATATGCATGACGCCGGAGGATCTTGAGACATTCCGTC
TGACCAAGGCTGGCTATGAAATGAGCCAGGACAACG-GC---GATGAAATC------
AACAAGAGGTTGAAAACCAAAGTGAATCCCACAACTCACATGTACACACATTGCGAGATCCATCCTAGCAT
GCTCCTAGGAATCTGTGCCAGTATTATTCCTTTCCCAGATCACAATCAGGTA------------------------
TGCACTTTTACTCGCCCCCTTTCCCATCTCGTTACTAATATGTTGTTGTGCAGTCACCCAGAAATACATACCAG
TCGGCTATGGGTAAGCAAGCTATGGGGTTTTTCCTTACAAATTACAACCGACGTATGGACACGATGGCAAA
CATTCTTTACTACCCGCAAAAGCCTCTTGGCACCACACGTTCTATGGAATTTTTGAAGTTCCGCGAGCTTCCG
GCTGGACAAAACGCTATCGTGGCCATCGCTTGTTACTCGGGATACAACCAAGAAGATTCCGTCATCATGAAC
CAGAGTAGCATCGATCGAGGTCTGTTCCGCAGTCTTTTCTTCAGATCCTATAGCGACTGCGAAAAGCGTGTT
GGCATCAACACGGTGGAATCATTCGAGAAGCCGTTCAAGAGCGACACGTTACGGCTCAAACAGGGCACGT
ACGATAAGCTTGACGACGATGGCATTGTTGCCCCCGGAGCCCGCGTCATTGGAGAGGACATCATCATCGGA
AAGACGTCACCCATCAACCCAGACAACGAAGAGATGGGACAGCGAACTAAAAATCACACCAAACGTGACG
CTTCCACGCCTCTGCGCAGCACGGAGAGTGGTATTGTCGACTCTGTCATCGTCACGACTAACCAGGATGGCC
TCCGTTACGTCAAGGTCCGCGTTCGAACTACGAAGATTCCCCAGATCGGCGACAAGTTCGCCTCTCGCCACG
GCCAGAAGGGAACCATTGGTGTTACATACAGACAAGAAGACATGCCCTTTACTTGCGAGGGCATTGTCCCC
GACATAATCATTAACCCGCATGCTATCCCATCTCGTATGACTATTGCTCATTTGATCGAGTGTTTGCTTAGTA
AAGTTTCCACCCTGAAGGGGATGGAGGGAGATGCGACGCCATTTACCGATGTTACGGTAGATTCGGTATCC
GGACTTTTGCGCGAACACGGATACCAGTCGCGCGGTTTCGAGATCATGTACCATGGCCACACCGGGCGCAA
GCTCCGAGCGCAGGTCTTCTTTGGGCCAACGTACTACCAACGTCTTCGACATATGGTGGACGACAAGATTCA
TGCTCGAGCACGTGGCCCGGTACAGATCATGACGCGACAGCCTGTTGAGGGTCGTGCGAGAGATGGAGGT
CTCCGTTTCGGAGAGATGGAACGTGATTGCATGATTGCTCATGGTGCCGCCTCTTTCCTGAAGGAGCGTCTA
TTTGAAGTATCGGATGCTTTCAGAGTACACGTCTGCGAGATCTGTGGACTCATGACGCCCATAGCGTAAGTT
TTCCTCGCCCCCCTTTTCTTTCCTGTCCACCTTTTA-------------------
AGGGTGATTGCTGATGTTCGTATTATAGTATCCTCAGCAAGGGTTCGTTCGAATGTCGTCCCTGCAAGAACA
AGACGAAGATCGCACAGATTTATATTCCATACGCTGCCAAGCTTCTATTCCAGGAGCTTCAGGCTATGGGTA
TCGCAACTCGCATGTTCACCGACCGCTCCGGTGTCAGCATCCGCTAG

>H_barbarensis_STMA_14081

GGCAAGAAGCGACTGGATCTTGCTGGTCCTCTACTGGCTAAGCTGTTCCGGAATATTGTTAGACGCTTAGTG
ATGGAAATCAATAATCACCTCAAGCGCTGCATCGACCAGAACAAACGTTTCCAGATCGAGCTGGCCGCTAA
GCCCGCTATTATCACTAACGGACTCAAGTACTCGCTCGCTACTGGCAACTGGGGTGACCAGAAGAAGGCCA
TGAGCTCGACCGCCGGTGTGTCGCAGGTCTTGAATCGATACACGTTT-----GCCTCGAC-
CTTATCTCACTTAAGGCGAACGAATACCCCCATCGGAAGAGACGGGAAGCTCGCGAAGCCCCGACAGCTTC
ACAACACCCATTGGGGTCTGGTCTGCCCGGCCGAGACGCCCGAAGGCCAAGCTTGTGGTTTGGTGAAGAA
CTTATCCTTGATGTGCTCGATCAGCGTGGGTACATCAACGGATCCTATCGTCGACTACATGATCACTAGGAA
CATGGAAGTGCTGGAGGAGTACGAACCTATGCGATACCCCAACGCCACCAAGATCTTTTTGAACGGTTCTT
GGATCGGGGTGCACCAGGACCCCAAGAACCTCGTCCGAGATGTCCAACACCTGCGCCGAACCAATCAGATC
CCCGCCGAGGTATCGTTGGTCCGCGACATTCGTGACCGTGAGTTCAAGATCTTCTCGGATGCCGGTCGCGTC
ATGCGCCCCTTGTTTGTCGTACAGCAAGAAGAT---
GACGAGGCCAACGGTATCAACAAGGGCTCGTTAGCACTTAGCAAGAGCATGATCCAACGACTAGAAGCGG
ATGCCGACGTCGACCCCAAGAGTGACGAGTACTTCGGGTGGCAAGGACTAGTAGACGAGGGAGCCATTGA
ATTTCTCGATGCCGAGGAAGAAGAGACAGCCATGATATGCATGACACCCGAGGATCTAGAGATCTATCGCC
AGAGCAAGGCCGGAATTGAAGTGTCTCAGGACAACG-GC---GACGAAATT------
AACAAACGACTCAAGACCAAGTTGAACCCGACGACGCATATGTACACGCATTGTGAGATCCATCCCAGTAT
GCTCCTGGGTATCTGCGCGAGCATCATTCCCTTCCCGGATCACAATCAGGTA---------------------TGCAAT-
CTCCTTTC-CGTGTGTCAGTACTAAT---
CACTAAC--

---CTCGCTTT-TA--

---GTCTCCCAGAAACAC--------------

--GTATCAGTCGGCCATGGGCAA--

>H_submonticulosa_CBS_115280

---CGTTT-----GCCTCGAC-
CTTATCTCACTTAAGGCGAACGAACACCCCCATCGGAAGAGATGGGAAGCTCGCGAAACCCCGACAGCTCC
ACAACACCCATTGGGGTCTGGTCTGCCCGGCCGAGACGCCCGAAGGCCAAGCTTGTGGTTTGGTGAAGAAT
TTGTCCTTGATGTGTTCCATCAGCGTGGGTACGTCGACGGATCCTATCGTAGATTACATGATTACTAGGAAC
ATGGAGGTGTTGGAGGAGTACGAACCTATGCGATACCCTAACGCCACCAAGATCTTCTTGAACGGTTCCTG
GATCGGCGTGCATCAGGATCCCAAGACCCTCGTCCGAGATGTTCAACACCTGCGCCGAACTAACCAGATCC
CCTCCGAGGTATCGTTGGTCCGCGACATTCGTGACCGTGAGTTCAAGATTTTCTCGGATGCCGGTCGTGTCA
TGCGTCCGTTGTTTGTCGTACAGCAAGAGGAT---
GATGAGTCCAACGGTATCACCAAGGGCTCGTTGGCGCTTAACAAGAGCATGATCCAACGACTAGAAGCAG
ATGCCGACATAGATCCTAAAAGTGAGGAGTATTTTGGCTGGCAAGGCCTGGTAGACGAAGGAGCTATTGA
ATTCCTCGATGCCGAGGAGGAGGAGACTGCCATGATTTGCATGACGCCCGAGGATCTAGAGATCTATCGCC
AGAGCAAGGCCGGAATTGAAGTGTCTCAGGACAACG-GT---GACGAGATT------
AACAAGCGACTCAAGACGAAGTTGAACCCGACGACACATATGTATACACATTGTGAGATCCATCCCAGTAT
GCTCCTGGGTATCTGCGCGAGCATTATTCCCTTCCCGGACCACAATCAGGCA------------------------TGCGAC-
GCCCTTAC-CTTGTGTTGGTACTTA---

>H_monticulosa_MUCL_54604

AACAGACGTTTCCAGATCGAACTCGCCGCTNAGCCCGCCATCATCACCAACGGGCTCAAGTACTCGCTGGC
CACGGGTAACTGGGGTGACCAGAAGAAGGCCATGAGCTCGACTGCCGGTGTATCACAGGTCTTGAATCGA
TATACGTTT-----GCGTCGAC-
CTTGTCTCACTTGAGGCGAACGAATACACCCATTGGAAGAGATGGGAAGCTCGCGAAGCCTCGACAGCTCC
ACAACACCCATTGGGGTTTGGTCTGCCCGGCCGAGACGCCCGAAGGCCAGGCTTGTGGTTTGGTGAAGAA
CTTGTCCTTGATGTGCTCAATCAGCGTGGGTACATCGACAGATCCTATAGTAGACTACATGATTACGAGGAA
CATGGAAGTCTTGGAGGAGTATGAGCCAATGCGATACCCTAACGCCACCAAGATCTTCTTGAATGGTTCTTG
GATCGGTGTGCACCAGGATCCCAAGAGCCTCGTCCGAGACGTCCAGCAGCTGCGCCGAACGAATCAGATCC
CTTCCGAAGTATCGCTGGTCCGCGATATTCGTGACCGCGAATTCAAGATCTTTTCCGATGCAGGCCGTGTCA
TGCGTCCCTTATTTGTCGTGCAGCAAGAGGAT---
GACGAAGCCAACGGTATCACCAAAGGCTCGTTAGCGCTGAATAAGAATATGATTCAGCGACTAGAGGCGG
ATGCCGATATCGACCCGAAGAGTGAGGAGTATTTCGGCTGGCAAGGCCTTGTCGACGAGGGAGCTATCGA
ATTCCTCGACGCCGAGGAAGAGGAGACCGCCATGATCTGCATGACGCCCGAGGATCTGGAAATCTACCGCC
AGAGCAAAGCCGGCATCGAAGTATCCCAGGATAATG-GT---GACGAAATC------
AACAAGCGACTCAAGACCAAGTTGAACCCAACGACACATATGTATACGCATTGTGAGATCCATCCCAGCAT
GCTTCTAGGTATCTGTGCGAGCATCATTCCCTTCCCGGACCACAATCAGGTA------------------------TGCA------
----

>J_multiformis_CBS_119016

GTTTCCNAATCGAACTAGCTGCCAAGCCCGCCATCATCACTAACGGTTTGAAATATTCTCTCGCTACAGGTA
ACTGGGGTGATCAGAAAAAGGCGATGAGCTCGACGGCTGGTGTATCACAAGTCTTGAACCGCTACACTTTC
-----TCATCAAC-
TCTTTCCCATTTGAGAAGGACAAACACACCTATCGGAAGAGACGGGAAGCTCGCCAAGCCACGACAGCTTC
ATAACACTCACTGGGGCCTGGTGTGTCCGGCCGAGACGCCCGAAGGCCAAGCCTGCGGGCTGGTGAAGAA
TCTGTCGCTCATGTGTTCCATCAGCGTGGGAACCTCGACGGATCCTATTGTCGATTATATGATAACGAGAAA
TATGGAAGTCCTCGAGGAGTATGAGCCGATGAGATATCCCAACGCCACTAAGATCTTTCTGAACGGTTCTTG
GATCGGTGTTCACCAGGACCCCAAGTCCCTCGTTCGAGACGTACAGACGCTCCGCCGTAACAACCAAATTCC
TTCTGAAGTCTCGTTGGTTCGCGATATCCGCGATCGTGAATTCAAGATCTTCTCGGATGCCGGTCGTGTGAT
GCGTCCTCTGTTCCGTGTACAACAGGATGAT--
CTTCCCGAGGAGGGCATAAACAAGGGCACCTTAGCTCTAACCAAGGATATGATCCAGCGTTTAGAGGCCGA
CCTTGATTTGGATCCGGATAGTGAAGAATACTTCGGTTGGCAAGGACTAGTCAACGAGGGTGTTGTTGAAT
TCCTTGATGCCGAGGAAGAGGAGACAGCCATGATTTGCATGACGCCCGAGGATTTAGAAAACTTCCGACTG
GCCAAGGCTGGATACCAGGTAGTTCTAGACAACG-GA---GACGAGGTG------
AACAAGCGATTGAAGACTAAGTTCAACCCGTCAACGCACATGTACACTCATTGCGAAATCCACCCCAGTATG
CTCCTAGGCATCTGCGCAAGCATCATTCCGTTCCC--

>H_trugodes_MUCL_54794

TATTGTTCGTCGTTTGACCCAGGAGATCACGAATACACTCAAACGCTGTATTAGCGACAACAAACGTTTCCA
GGTCGAATTGGCAGCCAAGCCTGCTATCGTCACAAATGGTCTCAAGTATTCCCTCGCGACGGGTAACTGGG

GTGACCAGAAGAAGGCCATGAGCTCAACGGCTGGTGTGTCGCAGGTCTTGAACAGATATACATTC-----
GCATCAAC-
TCTTTCCCACTTGAGGCGAACGAACACGCCTATCGGACGAGACGGAAAATTAGCTAAGCCCCGACAACTTC
ACAACACTCATTGGGGTCTTGTCTGCCCGGCCGAAACGCCCGAAGGCCAAGCTTGTGGGTTGGTCAAGAAT
TTATCGTTGATGTGTTCTATCAGTGTGGGTACCTCCACAGAACCCATCATTGACTATATGATCACGAGAAGT
ATGGAAGTGCTCGAGGAATATGAGCCCATGCGCTACCCCAATGCCACGAAGATCTTCCTCAATGGTTCTTG
GATTGGTGTACACCAAGATCCTAAGTCGCTTGTCAGAGATGTCCAACAGCTTCGCCGAGCCAACCAAATTCC
CTCGGAAGTTTCATTGGTTCGTGACATTCGTGATCGTGAGTTCAAGATATTCTCAGATGCAGGCCGTGTCAT
GCGTCCCTTGTTCGTTGTTCAACAAGAAGAC--
GCCCCTGAAGCTGGCATCGAGAAAGGTACACTGGCTTTGAACAAGGAAATGATCCAACGACTAGAGGCGG
ACGTCGATCTCGATCCGGAAAGCGAAGAGTATTTTGGTTGGCAAGGTCTAGTCAATGAGGGTGTTATTGAG
TATCTTGATGCCGAGGAAGAAGAGACGGCCATGATTTGCATGACTCCCGAAGACCTAGAAAACTACCGAAT
GACGAAGCTCGGCCACGAAGCACCACAGGACAACG-GC---GATGAAGTT------
AACAAACGACTCAAGACCAAGGTGAATCCTTCGACACACATGTATACGCACTGTGAGATCCATCCTAGCATG
CTTCTAGGTATCTGCGCCAGCATCATTCCTTTCCCGGATCACAATCAGGTA-----------------------TGTAAT-
CCACGTAA-ATCACCATA--

>H_haematostroma_MUCL_53301

AACAGGCGCTTCCAGATCGAGCTCGCTGCAAAACCCGCCATTATTACCAACGGGNTGAAGTATTCCCTTGCC
ACTGGTAACTGGGGTGACCAGAANAAGGNNATGAGCTCGACCGCTGGTGTATCGCAGGTCCTGAATAGAT
ACACATTC-----GCGTCGAC-
TCTTTCTCACTTGAGAAGAACCAACACCCCCATTGGGAGAGACGGGAAATTGGCGAAACCCCGTCAGCTTC

ATAACACTCATTGGGGCTTGGTCTGCCCGGCCGAAACGCCCGAAGGCCAGGCCTGTGGACTGGTGAAGAA
CTTGTCATTGATGTGCTCCATCAGCGTTGGCACGTCAACGGACCCTATCGTGGACTACATGATTACCAGGAA
TATGGAGGTCCTAGAGGAATACGAGCCGATGCGATACCCCAACGCCACTAAGATCTTCCTCAATGGCTCTT
GGATCGGTGTTCACCAAGATCCCAAGTCTCTCGTCAGGGATGTCCAGCAGCTACGTCGGGCCAACCAGATC
CCATCAGAGGTGTCATTGGTTCGCGATATTCGAGATCGAGAGTTCAAGATCTTCTCGGACGCTGGGCGTGT
CATGCGCCCCTTGTTTGTCGTGCAGCAAGAGGAT---
ATGCCCGAAAAGAATGTTTCCAAGGGGACATTAGCTCTCACCAAGGAGATGATCCAGAGACTAGAAGCGG
ATGTCGACATAGACCCGGATAGCGACGAGTATTTCGGTTGGCAGGGTCTGGTTAACTCGGGTGTTATCGAG
TATCTGGACGCCGAGGAAGAGGAGACGGCCATGATATGCATGACACCTGAGGACCTGGAGACGTATCGCA
TGGCCAAGGCCGGATATGATGTGACGCAGGATAATG-GA---GACGAGATT------
AACAGACGACTAAAAACCAAGATAAATCCTACCACGCACATGTATACCCATTGCGAGATTCACCCCAGCATG
CTTCTGGGCATCTGCGCCAGCATTATTCCCTTCCCCGACCACAACCAGGTA-----------------------
AGACATCATACCCGG-GCCTTTCTACCTGGTGCT---
TTCTTGCTTGCTAA---

>H_griseobrunneum_CBS_331_73

AGCTTGCGGCGAAGCCCTCCATCATCACCAACGGTCTGAAGTACTCTCTCGCGACGGGCAACTGGGGCGAC
CAGAAGAAGGCGATGAGCTCGACCGCTGGCGTGTCGCAGGTTCTGAACCGATACACTTTC-----GCCTCCAC-
CCTTTCTCATCTGAGAAGAACCAACACGCCTATTGGAAGAGACGGAAAGCTCGCAAAGCCCAGGCAGCTGC
ACAACACGCACTGGGGATTGGTCTGTCCGGCCGAGACGCCCGAAGGCCAGGCTTGTGGGTTGGTGAAGAA
CCTGTCGCTGATGTGCTCCATCAGCGTGGGAACGTCCACAGACCCTATTGTCGATTACATGATCACGAGAAA
CATGGAGGTTTTGGAAGAGTACGAACCCATGCGTTATCCCAACGCCACCAAGATTTTCCTCAACGGTTCTTG

GATTGGTGTGCACCAGGATGCCAAGACTCTGGTCAAGGACGTGCAGGAGCTCCGTCGCTCCAACCAGATTC
CCTCGGAAGTATCGCTGATACGCGACATCCGTGACCGCGAGTTCAAGATCTTCTCGGACGCCGGCCGCGTC
ATGCGCCCGCTCTTCGTGGTGCAGCAGGAGGAC--
ATCCCGGAGCGAGGCATCGCCAAAGGCACCCTAGCTCTCACAAAAGAAATGGTTCAGCGATTAGAGGCGG
ATCAGGAGCTTGACCCTGAGAGCGACGACTACTTCGGCTGGCAAGGCCTGTGTGAGGCGGGTGTCATCGA
GTACCTGGATGCCGAGGAAGAGGAGACTGCCATGATCTGCATGACGCCCGAAGACTTGGAAACTTATCGG
ATGGCCAAGGCCGGGTACGACATCCCTCAAGACAACG-GC---GACGAAGTC------
AACAAGCGACTGAAGACCAAGGTCAACCCCTCGACACACATGTATACTCACTGTGAGATCCATCCCAGCAT
GCTTTTGGGAATTTGCGCGAG--

>H_aveirense_MUM_19_40

CCTCGCGAAGTTGTTCCGTAACATTGTTCGACGTCTCAGTCAGGAGATTAGCAGCCAGCTGAAGAGGTGTA
TCGAATCTAACAAACGATTTTACGTCGAGCTTGCGGCTAAGCCTTCCATCATCACCAACGGTCTGAAGTACT
CTCTTGCGACAGGCAACTGGGGCGACCAGAAGAAGGCGATGAGCTCGACCGCCGGCGTGTCGCAGGTCCT
GAACCGATACACTTTC-----GCGTCCAC-
CCTTTCTCATTTGAGAAGAACCAACACGCCCATCGGAAGAGATGGAAAGCTTGCGAAGCCCAGACAGCTGC
ACAACACGCACTGGGGATTGGTCTGTCCGGCTGAGACGCCCGAAGGCCAGGCTTGTGGACTGGTCAAGAA
TCTGTCGCTGATGTGCTCCATCAGCGTGGGAACGTCTACGGACCCCATCGTCGACTACATGATTACCAGGAA
CATGGAAGTCTTGGAGGAGTACGAACCCATGCGTTATCCCAACGCCACCAAGATTTTCCTCAACGGTTCTTG
GATTGGTGTCCACCAGGATGCCAAGACCCTTGTTAAGGATGTCCAGGAGCTTCGTCGCTCCAACCAAATCCC
CTCAGAAGTATCTCTGATTCGCGACATTCGTGACCGTGAGTTCAAGATCTTCTCGGACGCTGGCCGCGTGAT

GCGCCCGCTGTTCGTGGTACAGCAGGAGGAC---
ATCCCCGAACGAGGCATCGCCAAGGGCACCCTGGCTCTCACCAAAGAAATGGTCCAGCGGCTAGAAGCAG
ATCAGGAGCTTAACCCCGACAGCGATGAGTATTACGGCTGGCAAGGTCTGTGCGAGGCGGGCGCCATCGA
GTACCTCGACGCCGAGGAGGAGGAAACGGCCATGATTTGCATGACGCCCGAAGACTTGGAAACCTATCGA
TTGGCCAAGGCCGGGTACGACATCCCTCAGGACAACG-GC---GACGAGGTC------
AACAAGCGACTGAAGACCAAGGTCAACCCCTCGACGCATATGTACACCCACTGCGAAATCCATCCCAGCAT
GCTTCTAGGAATCTGCGCGAGTATCATTCCCTTCCCCGACCACAATCAGTCGCCCGAA------------------------
----

>A_michelianum_CBS_119993

CGATATACCTTCCGCGTCCGACTCCTCCTCCTCCATTTGAGACGAACGAACACGCCCATCGGAAGAGACGGA
AAGCTAGCCAAGCCGCGGCAGCTTCACAACACTCATTGGGGTCTGGTCTGTCCGGCAGAGACGCCCGAAG
GCCAGGCTTGCGGGCTGGTGAAGAACTTGTCCCTGATGTGTTCCATCAGCGTTGGTACGTCGACAGATCCC
ATTGTAGACTACATGATTACTAGGAATATGGAAGTCCTCGAGGAATATGAGCCGATGAGATACCCAAACGC
CACGAAGATCTTCCTCAACGGCTCTTGGATCGGCGTGCACCAGGACGCCAAGACCCTCGTCAGAGATGTCC
AGGCGCTTCGCCGGGCCAACCAGATTCCCGCCGAGGTATCGCTGGTTCGTGATATCCGCGACCGCGAATTC
AAGATCTTCTCGGATGCCGGTCGCGTGATGCGTCCCTTGTTCCGCGTACAGCAGGAGGAT--------------------
----
ATCCAGGAGCTGAGCATCGAGAAAGGCACGCTGGCTCTTACCAAGCAGATGATCAAGCGCCTAGAGACGG
ACGTCGATTTGGATCCGGAGAGCGAAGGGTACTTCGGCTGGCAAGGCCTGGTCAACGAGGGTGTTATCGA
GTTCCTCGACGCGGAGGAAGAGGAGACCGCCATGATCTGCATGACGCCCGAAGATCTAGACATTTACCGCA

TGACCAAGCTCGGATACGACGTAGCCCAGGACAACG-GT---GACGAGGTT------
AACAAGCGACTGAAGACCAAAGTGAACCCGTCGACGCACATGTATACCCACTGCGAAATCCATCCCAGCAT
GCTCCTAGGCATCTGCGCGAGCATCATCCCGTTCCCGGACCACAACCAGGTA----------------------TGCGAT-
----------ACACGTAACACCTTTTG---
TCATCCGCAGCTAAC--

--CGTGAC---

>H_investiens_CBS_118183

TCATTGGGGTCTTGTCTGTCCAGCTGAGACGCCCGAAGGCCAGGCTTGCGGACTGGTGAAGAACTTATCGC
TGATGTGCTCTATCAGCGTGGGTACATCGACGGATCCTATCGTGGAGTATATGATTACTAGAAGTATGGAA
GTCCTCGAGGAATACGAACCGATGCGATACCCGAATGCCACCAAGATCTTCCTTAACGGGTCTTGGATCGG
TGTACACCAGGATCCCAAGACTCTTGTCAAGGATATCCAGGCCCTTCGTCGGGCCAACCAGATCCCCTCCGA
GGTTTCCTTGGTCCGCGATATCCGTGATCGCGAGTTCAAGATATTCTCAGATGCAGGTCGTGTCATGCGCCC
CTTGTTTGTCGTGCAACAAGAAGAT---
ATCCCCGATCAGGGTATTGCTAAGGGTACATTGGCCCTTACCAAAGAGATGATCCAACGACTAGAGGCCGA
TGTTGATCTTGATCCTGAAAGCGAGGAGTACTTCGGCTGGCAAGGTCTCGTTAACGAGGGTGTTATTGAGT
TTCTCGACGCGGAGGAAGAGGAAACGGCTATGATTTGCATGACACCGGAAGATCTGGAAAACTACCGGAT
GGCCAAGCTCGGCTACGAAGTGGTTCAGGATAACG-GA---GATGAGGTT------
AACAAACGACTCAAAACCAAGGTCAACCCCTCAACGCACATGTATACTCATTGCGAAATCCATCCTAGCATG
CTCTTGGGTATCTGCGCGAGTATCATTCCCTTCCCTGACCACAACCAGTCG-------------------------------------
---CCCAGAAACACGTACCAGT-------------

>H_carneum_MUCL_54177

CTCTCTCATCTGAGAAGAACCAACACGCCTATTGGGCGAGACGGAAAGCTAGCAAAGCCCCGACAGCTTCA
CAATACCCACTGGGGTCTAGTCTGCCCGGCCGAGACGCCCGAGGGTCAGGCCTGCGGTTTAGTGAAAAACC
TATCATTGATGTGTTCAATCAGTGTCGGTACATCCACGGATCCCATCGTGGATTATATGATCACCAGAAATA
TGGAAGTCCTCGAAGAGTATGAACCGCTACGATATCCCGACGCCACCAAGATCTTCCTCAACGGCTCTTGGA
TCGGGGTACACCAGAACCCCAAGGCTCTTGTGAGAGACGTGCAGAACCTGCGCCGAACTAATCAGATTCCG
GCCGAGGTGTCGTTGGTTCGTGACATACGCGATCGCGAATTCAAGATCTTTTCGGATGCCGGTCGGGTCAT
GCGTCCTTTGTTTGTCGTACAACAGGAAGAC--
CTCGAGGCCGGCGCTAAGAAGGGAACGTTGGCCCTCAATAAGGAAATGATCCAGAGGCTCGAGGCGGATG
TCGAGGTAGACCCCGACAGCGAGGAGTACTTTGGCTGGCAAGGCTTGGTCAACGAAGGTGTCATCGATTA
CCTCGACGCCGAGGAGGAAGAGACGGCCATGATCTGCATGACGCCCGAGGACCTTGAGACATACCGCCAG
ACCAAGGCCGGATACGCAGTGTCCCAGGATAACG-GC---GATGAGATC------
AACAAGCGACTGAGGACCAAGGTCAACCCCACCACACACATGTACACCCACTGCGAAATCCACCCTAGCAT
GCTCCTAGGTATCTGTGCGAGCATTATTCCGTTCCCGGACCATAATCAGGTA--------------------------
TGCTACCTTGCGGGCGCATTTCTTTTTTC--
TTTCTTTCTCT---

>H_cercidicola_CBS_119009

---TGTGTCGCAGGTG-TGAACCGATACACATTC--
---TCCTCGAC-
GCTTTCTCATTTGCGACGAACCAACACGCCCATTGGGCGAGACGGAAAGCTTGCGAAGCCTCGTCAGCTTC
ATAATACCCATTGGGGTCTGGTTTGCCCGGCCGAGACGCCTGAGGGTCAGGCTTGCGGCCTGGTGAAGAA
CTTGTCATTGATGTGTTCAATCAGCGTGGGTACTTCGACGGATCCTATCGTGGATTATATGATTACGAGGAA
TATGGAGGTTCTCGAGGAGTACGAGCCCTTGCGATACCCCGACGCTACCAAGATCTTCCTCAACGGCTCTTG
GATCGGAGTACATCAGAACCCCAAGGCTCTGGTGAGAGATGTGCAGAATCTGCGCCGGGCTAATCAGATTC
CGGCCGAAGTGTCGTTGGTCCGCGACATTCGTGACCGTGAATTCAAGATCTTTTCCGATGCCGGTCGGGTC
ATGCGTCCCTTGTTTGTCGTACAACAAGAGGAT---
ACCGAAGCTGGCACTAAGAAGGGGACGCTAGCTCTTACCAAGGAAATGATCCAGAGGCTCGAGGCAGACG
TTGATCTGGACCCCGACGGCGAGGAGTACTTTGGCTGGCAAGGCTTGGTCAATGAAGGTGTCATCGATTAC
CTTGACGCCGAGGAAGAGGAGACGGCTATGATTTGCATGACGCCCGAGGACCTCGAGATATACCGTCAGA
CCAAGGCCGGATTCGTAGTGTCCCAGGATAACG-GG---GATGAGATT------
AACAAGCGACTGCGGACCAAGGTGAACCCGACTACGCACATGTACACTCACTGCGAGATTCATCCCAGCAT
GCTCCTAGGTATCTGCGCGAGCATCATTCCGTTCCCGGATC---

>H_petriniae_CBS_114746

--GTGTTGAACCGATACACATTC-----
GCCTCGAC-
GCTTTCACATTTGCGAAGAACCAACACGCCCATTGGGCGAGATGGAAAGCTCGCGAAGCCTCGGCAGCTTC
ATAATACCCATTGGGGTCTGGTCTGCCCAGCCGAGACACCCGAGGGTCAGGCTTGCGGTCTGGTGAAGAAC
TTATCATTGATGTGTTCAATCAGTGTCGGCACCTCGACGGATCCTATCGTGGATTATATGATTACTAGGAAT
ATGGAGGTCCTCGAGGAGTACGAACCTTTGCGATACCCCGACGCTACCAAGATCTTCCTCAACGGCTCTTGG
ATCGGTGTACATCAGAACCCCAAGGCTCTAGTGAGAGACGTGCAGAATCTGCGCCGGGCTAATCAGATTCC
GGCCGAAGTGTCGTTGGTCCGCGACATTCGTGACCGTGAATTCAAGATCTTTTCCGATGCCGGTCGGGTCAT
GCGCCCCTTGTTTGTCGTACAACAAGAGGAT---
ACCGAGGCTGGCATTAAGAAGGGGACGCTAGCTCTTACTAAGGACATGATCCAGAGGCTCGAGGCAGACG
TCGATCTGGATCCCGACGGCGAGGAGTACTTTGGCTGGCAAGGCTTGGTCAATGAAGGTGTCATCGACTAC
CTTGACGCCGAGGAGGAAGAAACGGCCATGATTTGTATGACGCCCGAAGACCTTGAGACATACCGCCAGA
CCAAGGCGGGATTCGCAGTGTCCCAGGATAACG-GG---GATGAGATT------
AACAAGCGACTGCGGACCAAGGTTAACCCGACTACGCACATGTACACCCACTGCGAGATTCATCCTAGCAT
GCTCCTAGGTATCTGCGCGAGCATT---

>H_guilanense_MUCL_57726

--------------GCTGTTTCGG-
ATATTGTTCGTCGGCTCACTACGGAGATTACGAATCACTTGAAGCGATGCATCGACACCAACAAACATTTCG
AAATCGCCCTAGCCGCCAAGCCGGCTATTATTACGAATGGCCTCAAGTACTCGCTCGCTACAGGAAACTGG
GGCGATCAGAAGAAGGCCATGAGTTCGACAGCTGGTGTCTCGCAGGTCCTGAACCGATATACATTC-----
GCCTCGAC-
TCTCTCTCATTTGAGAAGAACCAATACGCCCATTGGCCGAGACGGAAAGCTGGCGAAGCCCCGTCAGCTTC
ACAATACCCATTGGGGCTTGGTCTGCCCGGCCGAAACGCCCGAGGGTCAGGCTTGCGGTCTCGTGAAGAAC
TTGTCATTGATGTGCTCAATCAGTGTCGGTACGTCTACTGACCCTATCGTCGACTATATGATCACTAGAAATA
TGGAGGTTCTCGAGGAGTACGAACCGCTACGATATCCCGATGCCACCAAGATCTTTCTCAACGGTTCTTGGA
TCGGTGTCCATCAGAACCCCAAGGCTCTGGTGAGAGATGTCCAGAATCTGCGTCGAACCAATCAGATCCCG
GCCGAGGTGTCGTTGGTCCGCGACATCCGCGATCGTGAATTCAAGATCTTTTCGGATGCCGGTCGGGTCAT
GCGCCCCCTGTTTGTAGTCCAGCAAGAGGAT---
ACCGAGGCTGGCATTAAGAAGGGAACGTTAGCTCTTACCAAGGAGATGATCCAGAGGCTCGAGGCGGATG
TCGATATCGATCCCGATAGCGATGAGTATTTTGGCTGGCAGGGTCTGGTCAACGAAGGTGTCATCGATTAT
CTTGACGCCGAGGAAGAAGAGGGGGCGATGATATGTATGACGCCCGAGGACCTCGAGACCTACCGCCAAA
CCAAGGCCGGTTACGCGGTGTCCCAGGATAACG-GG---GATGAGATT------
AACAAGCGACTGAGGACCAAGGTTAACCCGACCACCCACATGTACACGCACTGCGAGATTCATCCTAGTAT
GCTCCTAGGTATCTGCGCCAGTATTATTCCGTTCCCGGATCATAATCAGGTA-----------------------
TGCTATTTTGCTAA--

>H_texense_DSM_107933

CAAGCATTTCGAAATCGCTCTAGCCGCCAAGCCGGCTATTATTACGAATGGCCTCAAGTACTCGCTCGCCAC
AGGAAACTGGGGCGATCAGAAGAAGGCCATGAGTTCGACGGCTGGTGTCTCGCAGGTGTTGAACCGATAT
ACATTC-----GCCTCGAC-
TCTCTCTCATTTGAGAAGAACCAATACGCCCATCGGCCGAGACGGAAAGCTGGCGAAGCCCCGTCAGCTTC
ACAATACCCATTGGGGCTTGGTCTGCCCGGCCGAAACGCCCGAGGGCCAGGCCTGCGGTCTCGTGAAGAA
TTTGTCATTGATGTGCTCAATCAGTGTCGGTACGTCTACTGATCCTATCGTCGACTATATGATCACGAGGAAT
ATGGAGGTTCTTGAGGAGTACGAGCCGCTACGATATCCAGATGCCACCAAGATCTTTCTCAACGGTTCTTGG
ATCGGTGTGCATCAGAACCCCAAGGCTCTGGTAAGAGATGTCCAGAACCTGCGCCGAACCAATCAGATCCC
GGCCGAGGTGTCGTTGGTCCGCGATATTCGCGATCGTGAATTCAAGATCTTTTCGGATGCCGGTCGGGTCA
TGCGCCCCCTGTTTGTGGTACAGCAAGAGGAT--
ACCGAGGCTGGCATCAAGAAAGGAACGTTAGCTCTTACCAAGGAGATGATCCAGAGGCTCGAGGCGGATG
TCGATATCGATCCCGATAGCGACGAGTATTTTGGCTGGCAAGGCCTGGTCAACGAAGGTGTCATCGATTAT
CTTGACGCCGAGGAAGAAGAGACGGCGATGATATGTATGACGCCCGAGGACCTCGAGACCTACCGCCAGA
CCAAGGCCGGTTACGCGGTGTCCCAGGATAACG-GG---GATGAGATT------
AACAAGCGACTGAGGACCAAGGTTAACCCGACCACCCACATGTACACGCACTGCGAAATTCATCCTAGTAT
GCTGCTAGGTATCTGCGCCAGTATTATTCCGTTCCCAGACCACAATCAGGTA----------------------
CGCTATTTTGCTCGTGAATCT---

>H_canariense_MUCL_47224

TGCTGTCTCTCCTAGCGAAGCTATTTCGGAATATTGTTCGTCGGCTCACTACGGAGATTACAAACCACCTCAA
GCGATGCATCGACACCAACAAGCATTTCGAAATCGCCCTAGCCGCCAAGCCGGCCATTATTACGAACGGCC
TCAAGTACTCGCTCGCTACAGGAAACTGGGGCGATCAGAAGAAGGCCATGAGTTCGACGGCAGGTGTATC
GCAGGTCTTGAACCGATATACGTTC-----GCCTCGAC-
GCTTTCTCATTTGAGAAGAACCAACACGCCCATCGGCCGAGACGGAAAGCTAGCGAAGCCCCGTCAGCTTC
ACAATACCCATTGGGGCTTGGTCTGTCCGGCCGAAACGCCCGAGGGTCAGGCCTGCGGTCTCGTGAAGAAC
TTATCATTGATGTGTTCAATCAGTGTCGGTACGTCTACTGACCCTATCGTCGACTATATGATCACTAGGAACA
TGGAAGTTCTTGAGGAGTACGAACCGCTGCGATATCCAGATGCCACCAAGATCTTCCTCAACGGTTCTTGGA
TCGGTGTCCATCAGAACCCCAAGGCTCTAGTGAGAGATGTCCAGAATCTGCGCCGGACTAATCAGATCCCG
GCCGAGGTGTCGTTGGTCCGCGACATTCGCGATCGTGAATTCAAGATCTTTTCGGATGCCGGTCGGGTCAT
GCGCCCCTTGTTTGTAGTACAGCAAGAGGAT---
AGCGAGGCTGGCGTTAAGAAGGGAACGCTAGCTCTTACCAAGGAGATGATCCAGAGGCTCGAGGCGGAT
GTCGATACCGATCCCGATAGCCATGAGTATTTTGGCTGGCAAGGTCTGGTCAACGAAGGTGTCATCGATTA
CCTTGACGCCGAGGAAGAAGAGACGGCGATGATATGTATGACGCCCGAGGACCTCGAGACCTACCGCCAG
ACCAAGGCCGGTTACGTCGTATCCCAGGATAACG-GG---GACGAGATT------
AATAAACGGCTAAGGACCAAGGTGAACCCGACCACGCACATGTACACCCACTGCGAAATTCATCCTAGCAT
GCTCCTAGGTATTTGCGCCAGTATCATTCCTTTCCCAGATCATAATCAGGTA------------------------
TGTTATGTTGCTGATGCATCTTGGTA---
TCTATTGCTAAC---

--CGTGTCGC--

>H_laschii_MUCL_52796

GCGAAGCTGTTTCGGAATATTGTTCGTCGGCTCACTACGGAGATTACGAATCATCTGAAGCGCTGCATCGAC
ACCAACAAGCATTTCGAAATCGCCCTAGCCGCCAAGCCGGCTATCATTACGAACGGCCTCAAGTACTCGCTC
GCTACGGGAAACTGGGGCGATCAGAAGAAGGCTATGAGCTCGACGGCTGGTGTGTCGCAGGTCCTGAACC
GATATACGTTC-----TCCTCGAC-
GCTTTCTCACTTGAGAAGAACCAATACGCCCATCGGGCGAGACGGAAAGTTGGCCAAGCCCCGTCAGCTTC
ACAACACCCATTGGGGCCTGGTATGCCCGGCCGAAACGCCCGAGGGCCAGGCCTGCGGCCTCGTGAAGAA
CTTGTCATTGATGTGTTCAATCAGTGTCGGTACGTCTACTGACCCTATCGTCGACTATATGATCACTAGAAAT
ATGGAGGTTCTCGAGGAGTACGAGCCGTTGCGATATCCCGATGCCACCAAGATCTTCCTCAACGGCTCTTG
GATCGGTGTCCACCAGAACCCCAAAGCTCTGGTGAGAGACGTCCAGAATCTGCGCCGAACCAATCAGATCC
CGGCCGAGGTGTCGTTGGTCCGCGACATTCGCGATCGTGAATTCAAGATCTTTTCGGATGCCGGCCGGGTC
ATGCGCCCCTTGTTTGTAGTACAGCAAGAGGAT--
ACCGAGACTGGTCCTAAGAAGGGAACGTTGGCTCTTACCAAGGAGATGATCCAGAGGCTGGAGGCGGATG
TCGATATAGACCCCGATAGCGATGAGTATTTCGGCTGGCAAGGTCTGGTCAACGAAGGTGTCATCGATTAT
CTCGACGCCGAGGAAGAAGAGACGGCGATGATATGTATGACGCCCGAGGATCTCGAGACCTACCGCCAGG
CCAAGGCCGGTTACGCCGTGTCCCAGGATAACG-GG---GATGAGATT------
AACAAGCGACTGAGGACCAAGGTTAATCCGACCACGCACATGTATACCCACTGCGAGATCCATCCCAGCAT
GCTGCTAGGGATTTGCGCAAGTATTATTCCGTTCCCGGATCACAATCAGGTA------------------------
TGCTACTCTGCTAGTGCATCTTGGTA--
TGTATTGCTAAT---

--CGTGTC---

>H_rubiginosum_MUCL_52887

CGCTGCATCGACACTAACAAGCATTTTGAAATCGCCCTAGCCGCCAAGCCGGCTATTATTACGAACGGCCTC
AAGTACTCGCTCGCTACAGGAAACTGGGGCGATCAGAAGAAGGCCATGAGTTCAACGGCCGGTGTGTCGC
AGGTCCTGAATCGATACACGTTC-----GCCTCGAC-
GCTTTCTCATTTGAGAAGAACCAATACGCCCATCGGGCGAGACGGAAAGCTTGCGAAGCCCCGTCAGCTTC
ACAATACTCATTGGGGCCTGGTCTGCCCGGCCGAAACGCCCGAGGGTCAGGCCTGCGGTCTGGTGAAGAA
CTTATCATTGATGTGTTCAATCAGCGTCGGTACGTCCACGGATCCTATCGTGGATTATATGATTACTAGGAAT
ATGGAGGTTCTCGAGGAGTACGAGCCGCTGCGATATCCCGATGCTACCAAGATCTTCCTGAACGGCTCTTG
GATCGGTGTACATCAGAACCCCAAGGCTCTGGTGAGAGATGTCCAGAATCTGCGCCGAACTAATCAGATCC
CGTCCGAGGTGTCGTTGGTCCGCGACATTCGCGATCGTGAATTCAAGATCTTTTCGGATGCCGGTCGGGTC
ATGCGCCCCTTGTTTGTGGTACAGCAAGAGGAT--
ACCGAAGCTGGCGCCAAGAAGGGAACGTTAGCTCTTACCAAGGACATGATCCAGAGGCTCGAGGCGGATG
TCGAAATAGATCCCGACAGTGATGAGTATTTTGGCTGGCAAGGTTTGGTCAACGAAGGTGTCATCGATTAT
CTCGACGCCGAGGAAGAAGAGGGGGCGATGATATGTATGACGCCCGAGGACCTCGAGACCTACCGCCAGA
CCAAGGCCGGATACGCGGTGTCTCAGGATAACG-GG---GATGAGATT------
AACAAGCGACTGAGGACCAAGGTTAACCCGACCACGCACATGTACACTCACTGCGAGATTCATCCTAGCAT
GCTCTTGGGTATTTGCGCCAGCATCATTCCATTC--

>H_gibriacense_MUCL_52698

---------------AGCTATTCCGG-

ATATTGTTCGTCGGCTCACTAACGAGATTACAAACCACCTTCGACGCTGCATCGATACAAACAAGCATTTCG
AAATTGCCCTGGCTGCCAAGCCTGCTATTATTACGAACGGTCTCAAGTACTCGCTCGCTACAGGAAACTGGG
GCGACCAGAAAAAGGCCATGAGCTCGACGGCTGGCGTGTCGCAAGTCTTGAACCGGTACACGTTC-----
GCCTCGAC-
GCTTTCTCATTTGCGAAGAACAAACACGCCCATCGGGCGTGATGGAAAGCTGGCGAAGCCTCGGCAGCTTC
ACAATACCCATTGGGGTCTAGTCTGCCCGGCCGAAACGCCCGAGGGTCAGGCTTGCGGTCTGGTGAAGAA
CTTGTCATTGATGTGTTCGATCAGCGTCGGTACGTCCACGGATCCTATCGTAGACTATATGATTACTAGAAA
TATGGAGGTTCTCGAGGAGTATGAACCGCTGCGATATCCCGATGCTACCAAGATCTTCCTCAACGGCTCCTG
GATCGGCGTACATCAGAACCCGAAGGCTCTGGTGAGAGACGTTCAGAACTTGCGCCGTACCAATCAAATCC
CGGCCGAGGTTTCGTTGGTACGCGACATACGCGACCGTGAATTCAAAATCTTTTCGGATGCCGGTCGCGTT
ATGCGCCCCATGTTCGTTGTGCAGCAAGAGGAC---
ACCGAGGCTGGCACTAAGAAGGGAACGCTAGCTCTCACCAAGGAGATGATCCAGAGGCTCGAGGCGGATG
TCGAGATAGATCCTGATAGCGAGGAGTACTTTGGCTGGCAAGGCTTGGTCAACGAAGGTGTCATCGATTAT
CTTGACGCCGAGGAGGAAGAAACGGCGATGATCTGCATGACGCCCGAGGACCTCGAGACCTACCGACAGA
CTAAGGCCGGATACGAAGTGTCCCAAGACAACG-GG---GATGAGATT------
AATAAGCGACTGAGGACCAAGGTTAACCCGACCACGCACATGTACACCCACTGCGAGATCCATCCTAGCAT
GCTTCTAGGTATTTGCGCAAGCATTATTCCGTTCCCGGATCACAATCAGGTA-----------------------
TGCTGCTTTGTTATTGCATATCGATGC--
TTTTTTACTAAC--

--GGTGTGGTGT--

>H_sporistriatatunicum_UCH9542

CTCTTAGCGAGCTCTTCCGGAATATTGTTCGTCGGCTCACTAACGAGATTACGAATCACCTTCGACGCTGCAT

CGATCTGAATAAGCATTTTGAAATTGCCCTAGCTGCTAAGCCTACCATCATCTCAAACGGCCTCAAGTACTC
GCTCGCCACAGGAAACTGGGGCGATCAGAAAAAGGCCATGAGCTCGACGGCCGGTGTGTCGCAAGTCTTG
AACCGATACACTTTC-----GCCTCTAC-
GCTTTCTCATTTGAGAAGAACTAACACGCCCATTGGGCGAGATGGAAAGCTGGCGAAGCCTCGACAGCTTC
ACAATACTCATTGGGGTTTGGTTTGCCCGGCTGAGACGCCTGAGGGTCAGGCTTGTGGTCTGGTGAAGAAC
TTGTCGCTGATGTGTTCGATCAGTGTCGGTACGTCCACAGATCCTATCGTAGATTACATGATTACGAGAAAC
ATGGAGGTTCTCGAAGAGTACGAACCGCTGCGATACCCCGACGCCACCAAGATCTTCCTCAACGGCTCTTG
GATCGGCGTGCACCAGAATCCCAAGGCTCTAGTGAGAGATGTTCAGAACCTGCGCCGAACCAATCAAATCC
CGGCTGAGGTGTCGCTAGTCCGCGACATACGCGACCGTGAATTCAAAATCTTCTCAGACGCCGGTCGTGTT
ATGCGCCCTATGTTTGTTGTACAGCAAGAAGAT---
ACCGAGGCTGGCGCTAAGAAGGGAACGTTAGCTCTCACTAAGGAGATGATCCAGAAGCTCGAGGCAGATG
TCGAGATAGATCCTGAGAGCGATGAGTACTATGGCTGGCAAGGCTTGGTCAACGAAGGCGCCATCGATTAT
CTCGACGCCGAGGAGGAAGAAACAGCCATGATCTGCATGACGCCTGAGGACCTTGAGACCTACCGTCAGG
CTAAAGCCGGATTCGAGGTGTCCCAGGATAACG-GA---GACGAGGTT------
AATAAACGACTGAGGACCAAGATTAACCCGACCACGCACATGTACACTCACTGCGAGATCCATCCCAGCAT
GCTTCTAGGTATTTGCGCAAGCATTATTCCGTTCCCGGATCATAATCAGGTA-------------------------
TGTTACTTTGTTAGCACATGTTGGTGCT---
TTCTTTTCACTAAC---

---TGTATGGTG---

>H_pilgerianum_STMA_13455

AGATTACGAACAACCTGAGACGCTGTATCGACACGAACAAGCATTTCGAAATTGCCCTGGCTGCTAAGCCG
GCTATCATCACGAACGGTCTCAAGTACTCACTCGCCACAGGAAACTGGGGTGATCAGAAGAAGGCCATGAG

CTCGACGGCTGGTGTGTCCCAGGTCTTGAACCGATACACCTTC-----TCTTCCAC-
CCTTTCTCATTTAAGAAGAACCAACACACCTATTGGGCGTGACGGCAAGTTGGCGAAGCCCCGACAGCTTC
ACAATACACATTGGGGTCTAGTCTGTCCGGCCGAAACGCCCGAGGGCCAGGCCTGCGGCTTGGTGAAGAA
CTTATCATTGATGTGTTCCATCAGCGTCGGTACCTCGACGGATCCCATCGTGGATTACATGATTACCAGGAA
TATGGAGGTTCTCGAAGAGTACGAGCCGCTACGGTATCCCGATGCCACCAAGATCTTCCTCAACGGTTCCTG
GATAGGTGTTCATCAGAATCCCAAGGCGCTGGTGAGAGATGTTCAGAATCTTCGGCGGACGAATCAGATCC
CGGCCGAGGTGTCGTTAGTCCGCGACATACGCGATCGCGAATTCAAAATCTTTTCGGATGCCGGTCGCGTC
ATGCGCCCCCTGTTTGTTGTACATCAAGAGGAC--
ACCGAGGCCGGCACTAAGAAGGGAACGTTAGCTCTTACTAAAGACATGATCCAGAGGCTCGAGTCGGATG
TCGAAGTAGATCCTAATAGCGAGGAGTACTTTGGCTGGGAAGGCTTGGTCAACGAAGGTGCTATCGATTAT
CTAGACGCCGAGGAGGAAGAAACGGCCATGATATGCATGACGCCCGAGGACTTGGAAACCTATCGGCAGA
CCAAGGCCGGGTTCGAAGTATCCCAGGATAACG-GG---GACGAGATT------
AATAAGCGACTGAGGACCAAGGTGAACCCGACCACGCACATGTACACTCACTGCGAGATCCATCCTAGCAT
GCTTCTAGGTAT---

>H_wuzhishanense_FCATAS2708

CGTTCGACGGCTCACTAACGAGATTACGAATCAGCCTGAGCGCTGCATCGACACCAACAAGCACTTCGAGA
TGGCCCTGGCTGCTAAGCCGGCTATCATTACGAACGGTCTCAAGTACTCGCTCGCCACAGGAAACTGGGGC
GATCAGAAGAAGGCCATGAGCTCGACCGCCGGTGTGTCGCAGGTGTTGAACCGATACACCTTC-----
TCTTCGAC-
ACTTTCTCATTTGAGACGAACCAACACGCCTATTGGGCGTGACGGAAAACTGGCGAAGCCTCGACAACTTC

ACAATACTCATTGGGGTCTGGTCTGCCCGGCCGAAACGCCCGAGGGCCAGGCCTGTGGCTTGGTGAAGAA
CTTATCGCTGATGTGTTCCATCAGCGTCGGCACCTCTACAGATCCCATCGTGGATTATATGATTACTAGAAAT
ATGGAAGTCCTCGAAGAGTACGAGCCGCTGCGGTATCCTGACGCCACCAAGATCTTCCTGAACGGTTCTTG
GATCGGTGTACACCAGAACCCCAAGGCTCTAGTGAGAGATGTTCAGAATCTTCGGCGAACCAACCAGATCC
CGGCCGAGGTGTCGCTAGTCCGCGACATACGCGATCGTGAATTCAAGATCTTTTCGGATGCCGGTCGCGTC
ATGCGACCCCTGTTTGTGGTACAGCAAGAGGAC---
ACCGAGGCTGGCGCTAAGAAGGGAACGCTAGCCCTTACTAAAGAGATGATCCAGAGGCTCGAGGCGGATG
TCGAGGTAGATCCTAATAGCGAGGAGTACTTTGGCTGGGAAGGCTTGGTCAATGAAGGTGCTATCGATTAT
CTCGACGCCGAGGAGGAAGAGACGGCGATGATCTGCATGACGCCCGAGGATCTTGAGACCTATCGCCAGA
CCAAGGCCGGATACGAAGTGTCTCAGGATAACG-GA---GACGAGATT------
AACAAGCGACTGAGGACCAAGGTTAATCCGACCACACACATGTATACTCACTGCGAGATCCATCCGAGTAT
GCTTCTGGGTATCTGCGCGAGCATTATTCCATTCCCGGATCACAATCAGGTA----------------------
TGTCACTTTACTAGCAATTCTAGGGAC---
CTTTTCACTA---

>H_ochraceum_MUCL_54625

TCGACGGCTAACTACCGAGATTACGAATCACCTAAGACGCTGCATCGATACAAACAAACATTTTGAAATCGC
CCTAGCTGCCAAGCCGGCTATCATTACTAACGGTCTTAAATACTCGCTTGCTACAGGAAATTGGGGTGACCA
GAAGAAGGCCATGAGCTCGACRGCYGGCGTCTCGCAGGTCTTGAACCGATACACGTTC-----GCTTCGAC-
ACTTTCTCATCTGAGACGAACCAACACGCCTATTGGGCGGGACGGAAAACTGGCGAAGCCCCGGCAACTTC
ACAATACCCATTGGGGTTTAGTCTGCCCGGCCGAAACACCCGAAGGTCAGGCCTGCGGTTTGGTCAAGAAC
TTATCACTGATGTGTTCAATCAGTGTTGGCACTTCGACGGACCCTATCGTTGATTATATGATTACTAGGAATA
TGGAGGTCCTTGAAGAGTACGAACCACTGCGATACCCCGACGCTACCAAGATCTTCCTGAACGGTTCCTGG

ATCGGCGTGCATCAGAACCCCAAGTCTCTCGTGAGAGATGTTCAGAATTTGCGGCGGACAAACCAGATTCC
GGCCGAGGTGTCATTGGTCCGCGATATACGTGATCGTGAATTCAAGATCTTTTCGGACGCTGGTCGCGTCAT
GCGCCCCTTGTTCGTCGTACAGCAAGAAGAC--
ACCGAGGCTGGTACTAAGAAGGGGACGCTGGCCCTTACAAAAGAAATGATTCAGAGGCTCGAGGCGGATG
TCGACGTAGATCCCAATAGTGAGGAGTACTTTGGCTGGCAAGGCTTGGTCAACGAAGGTGTCATCGATTAT
CTCGACGCCGAGGAGGAAGAGACGGCCATGATCTGCATGACACCCGAGGATCTTGAGACATACCGCCAAA
CCAAGGCCGGATACGAAGTGTCCCAGGATAACG-GG---GATGAGATT------
AATAAGCGACTGAGGACCAAGGTTAACCCGACCACGCATATGTACACTCATTGCGAAATCCATCCTAGTATG
CTTCTAGGTATYTGCGCGAGCATTATTCCGTTCCCCGATCATAATCAGGTA------------------------
TGTCACTTTCCTGGCGTGTCTCAGTA---

>H_chionostomum_STMA_14060

AAGCTGTTCCGCAATATCGTTCGTCGGCTCACCAACGAGATCTCGAACCACCTCAAACGCTGCATCGACACG
AACAAGCATTTCGAGATTGCCCTGGCTGCCAAGCCGGCCATCATCACGAACGGTATCAAGTACTCGCTCGCT
ACAGGAAACTGGGGCGACCAGAAGAAGGCCATGAGCTCGACCGCCGGCGTGTCGCAGGTCTTGAACAGAT
ACACGTTC-----TCCTCGAC-
GCTTTCCCATCTGAGAAGAACCAACACGCCCATCGGGCGAGACGGAAAGCTGGCGAAGCCCCGTCAGCTTC
ACAATACCCATTGGGGTCTGGTCTGCCCGGCCGAGACGCCCGAGGGTCAAGCCTGCGGTCTGGTCAAGAA
CTTATCCTTGATGTGTTCGATCAGCGTCGGTACTTCCACAGATCCTATCGTCGACTATATGATTACGAGGAAC
ATGGAGGTTTTGGAGGAGTACGAACCGCTGCGATACCCCGACGCCACCAAGATTTTCCTCAACGGCTCGTG
GATTGGCGTACACCAGAACCCCAAGGCCCTGGTCAGAGACGTCCAGAACCTGCGCCGAACCAACCAGATCC
CTGCCGAGGTGTCGCTGGTTCGCGACATACGCGACCGCGAGTTCAAGATATTTTCTGATGCCGGCCGCGTC

ATGCGCCCGTTGTTCGTTGTGCAGCAAGAGGAT--
ACCGAGGCCGGCATCAAGAAGGGAACGCTGGCTCTCACCAAGGAGATGATCCAGAGACTCGAGGACGATG
CTGAGATAGATCCCGACAGCGATGAGTACTTTGGCTGGCAAGGCTTGGTCAACGAAGGTGTCATCGATTAT
CTCGATGCCGAGGAGGAAGAGACGGCCATGATCTGCATGACGCCCGAGGACCTCGAGACCTACCGCCAGG
CGAAGGCCGGCTTCGACGTGTCCCAGGATAACG-GC---GACGAGATT------
AACAAGCGCCTGAGGACCAAGATCAACCCGACCACGCACATGTACACCCACTGCGAGATCCATCCCAGCAT
GCTTCTGGGTATCTGCGCGAGCATCATTCCGTTCCCGGATCATAACCAGGTA-----------------------
TGCTACCTTGCTATTGCGTCCTGGTATC--
TTTTTTATGCTAAC--

---CGTACCGCGTA---

>H_musceum_MUCL_53765

---C-
GCTTTCTCACTTGAGAAGAACCAACACGCCTATTGGGCGAGATGGAAAGTTGGCGAAGCCCCGGCAGCTTC
ACAATACCCATTGGGGTCTAGTCTGCCCGGCCGAGACGCCCGAGGGTCAGGCTTGCGGTCTGGTGAAGAA
CTTGTCGCTGATGTGTTCGATCAGTGTTGGTACCTCTACGGATCCTATCGTAGACTACATGATTACCAGAAAT
ATGGAGGTTCTCGAGGAGTACGAACCGCTGCGATACCCCGATGCTACTAAGATCTTCCTCAACGGCTCTTG
GATCGGCGTACATCAGAATCCCAAGGCTCTAGTGAGGGATGTTCAGAATTTACGTCGTACCAATCAGATCC
CGGCCGAGGTGTCGTTGGTCCGCGACATTCGCGATCGTGAATTCAAGATCTTTTCAGATGCCGGTCGTGTCA
TGCGCCCCATGTTCGTTGTACACCAAGAAGAC---
ACCGAGGATGGTATTAAGAAGGGAACGCTTGCTCTCACCAAGGACATGATCGCGAGGCTCGAGGCAGATC
TCGACGTAGATCCTGATAGTGAGGAGTACTTTGGCTGGGAAGGCCTGGTCAATGAAGGTGCCATCGATTAT
CTTGATGCCGAGGAGGAAGAAACGTGCATGATTTGCATGACGCCCGAGGAACTCGAGGGCTACCGCCAGC

GTAAGGCCGGATTCGAAGTTTCTCAGGATAACG-GG---GATGAGATT------
AATAAGCGACTCAAGACTAAGATCAATCCGACGACACATATGTATACTCATTGCGAGATCCATCCCAGTATG
CTTCTAGGTATTTGCGCAAGCATTATTCCGTTCCCGGATCATAATCAGGTA----------------------
TGCTACTCTA----TGCTTCTTGGT--

>H_isabellinum_MUCL_53308

GTCTCTCTTAGCGAAGCTGTTCCGGAATATTGTTCGTCGGCTCACTAACGAGATTACAAATCACCTTCGACG
CTGCATCGACACGAATAAGCATTTTGAAATTGCCCTTGCAGCCAAGCCCTCTATCATTTCGAATGGTCTCAA
GTACTCACTCGCCACAGGAAACTGGGGTGATCAGAAGAAAGCCATGAGCTCGACGGCCGGCGTGTCGCAA
GTCTTGAACCGATACACTTTC-----GCCTCGAC-
GCTTTCTCATTTGAGAAGAACCAACACACCTATTGGGCGAGATGGAAAGTTGGCGAAGCCTCGGCAGTTGC
ATAATACCCATTGGGGTCTGGTCTGTCCGGCTGAGACGCCTGAGGGTCAGGCCTGCGGTCTGGTGAAGAAC
TTGTCATTGATGTGTTCGATCAGTGTTGGCACGTCTACGGATCCTATCGTTGATTACATGATTACTAGAAATA
TGGAGGTGCTCGAGGAGTACGAGCCGTTGCGATATCCTGATGCTACCAAGATCTTCCTCAATGGCTCTTGG
ATCGGCGTGCATCATAACCCTAAGGCCCTGGTGAGAGACGTTCAGAACCTGCGCCGGACCAACCAGATCCC
GGCTGAGGTGTCACTAGTCCGTGACATACGTGACCGCGAATTCAAGATCTTTTCAGATGCTGGTCGCGTTAT
GCGCCCGGTGTTTGTCGTGGAGCAAGAGGAC---
ACGGAAGCTGGCGCTAAGAAAGGAACGTTAGCTCTCACAAAGGAGATGATCCAAAGACTCGAGGCGGATG
TCGAGATAGATCCCGAGAGTGAACAGTACTTTGGCTGGGAAGGTTTGGTCACTGCGGGTGCCATCGATTAT
CTCGACGCCGAGGAAGAAGAGACGGCCATGATCTGCATGACGCCTGAGGACCTTGAGACCTATCGCCAGA
CCAAGGCCGGATACGAAGTGTCCCAGGATAATGGGG---GATGAGGTT------
AATAAACGACTGAGGACTAAGGTTAACCCGACCACACACATGTACACTCACTGCGAGATCCACCCTAGCAT
GCTTCTAGGTATTTGCGCAAGCATTATTCCATTCCCGGATCATAATCAGGTA----------------------

TGTTACTCTGTTGGTGCATTTTGAGAC---
TTCTCTTGCTAAC---

--CG---

>H_perforatum_CBS_115281

---TACACTTTC-----GCCTCGAC-
GCTTTCTCATTTGAGAAGAACTAACACGCCCATCGGGCGAGATGGAAAGCTGGCAAAGCCTCGACAGCTTC
ACAATACCCATTGGGGTCTGGTCTGCCCCGCCGAAACGCCTGAGGGTCAAGCCTGCGGTCTGGTGAAGAAC
TTATCACTGATGTGTTCCATCAGCGTCGGCACATCTACGGATCCCATCGTAGATTACATGATTACGAGAAAT
ATGGAGGTACTCGAGGAGTACGAACCCTTACGATACCCCGACGCCACGAAAATCTTCCTCAACGGCTCTTG
GATCGGCGTGCACCAGAACCCTAAAGCTCTGGTGAGAGATGTTCAGAATCTGCGCCGGACCAATCAGATCC
CGGCCGAGGTGTCGTTGGTCCGCGACATACGTGATCGTGAATTCAAGATCTTTTCAGATGCTGGCCGCGTT
ATGCGCCCCATGTTCGTTGTTAACCAAGAGGAC---
ACGGAGGGTGGCGCTAAGAAGGGAACGCTAGCTCTCACCAAGGAGATGATTCAGAGGCTCGAAGCAGAT
GTCGAGATAGATCCTAACAGTCAGGAGTACTTTGGCTGGCAAGGCTTGGTCAACGAAGGTGCCATCGATTA
TCTTGATGCCGAGGAGGAAGAAACGGCCATGATCTGCATGACACCCGAAGACCTCGAGACCTACCGACAG
ACTAAGGCCGGATACCAAGTGTCGCAAGACAACG-GG---GATGAGGTT------
AACAAACGTCTGAGGACCAAAGTCAATCCGACCACTCACATGTACACTCACTGTGAGATCCACCCTAGCATG
CTTCTAGGTATCTGTGCCAGCATCATTCCGTTCCCGGATCACAACCAGGTA-------------------------
TGTTACTCTATTGATGCAGTTTGGTGC---
TTTTCTCGCTAAC---

--CGTGTTGTGTA---

>H_samuelsii_MUCL_51843

TATTGTTCGCCGGCTAACTACGGAGATTACGAATCACCTGAAGCGCTGCATCGAGTCGAACAAACATTTCGA
AATCGCCCTGGCTGCCAAACCAGCGATTATCACGAATGGTCTGAAATACTCGCTCGCCACCGGAAATTGGG
GCGACCAGAAGAAGGCGATGAGTTCGACAGCTGGCGTGTCTCAGGTCCTGAACCGATACACGTTC-----
GCTTCTAC-
GCTTTCCCACTTGAGAAGAACCAATACGCCTATCGGCCGAGACGGGAAGCTCGCGAAACCCCGGCAGCTTC
ACAATACCCATTGGGGTCTCGTCTGCCCGGCCGAAACACCTGAGGGTCAGGCCTGTGGTCTAGTGAAGAAC
TTATCGCTGATGTGTTCGATCAGCGTGGGCACGTCTACGGATCCTATAGTAGATTATATGATCACTAGGAAT
ATGGAGGTTCTGGAGGAATACGAACCCTTACGATACCCTGATGCTACCAAGATCTTCCTCAACGGCTCTTGG
ATCGGTGTACACCAGAACCCCAAGGCTCTAGTAAGAGACGTCCAGAATCTGCGCCGGACAAATCAGATTCC
GGCCGAGGTGTCGCTGATCCGTGACATACGCGATCGCGAATTCAAGATCTTTTCGGATGCTGGCCGCGTCA
TGCGCCCCTTGTTTGTTGTGAATCAAGAGGAT---
ACCGAGGCAGGCATTAAAAAGGGAACCTTAGCTCTCACTAAGGAGATGATTCAGAGACTTGAAGCAGATG
TCGACCTGGATCCTGAAAGCGAGGAATACTTTGGCTGGCAAGGCCTGGTCAATGAAGGTGTCATTGATTAC
CTCGATGCAGAAGAAGAAGAGACAGCCATGATCTGCATGACACCCGAGGACCTAGAGACCTATCGCCAGA
CCAAGCTCGGCTACAATGTGTCTCAGGATAACG-GG---GACGAGATT------
AATAAGCGACTGAGGACTAAGGTTAACCCGACTACCCATATGTATACCCACTGCGAGATCCATCCTAGCATG
CTCTTAGGTATATGCGCGAGCATTATCCCGTTCCCGGATCATAATCAGGTA------------------------
TGGCCCTTTCCTAGCACACCCTGCTA--
CTACTACTAAC--

--CTTAGC--

>H_munkii_MUCL_53315

AGCTGTTCCGCAATATCGTTCGTCGGCTTACTACGGAGATTACTAATCACCTGAAGCGATGCATCGAGTCGA
ACAAGCATTTCGAAATCGCCCTAGCTGCTAAACCGGCCATTATCACGAACGGCCTCAAGTACTCGCTCGCCA
CGGGAAATTGGGGCGATCAGAAGAAGGCCATGAGTTCGACCGCTGGTGTGTCTCAGGTCCTGAATCGATA
CACGTTC-----GCTTCTAC-
GCTTTCTCACTTGAGAAGAACCAACACGCCTATTGGTCGTGACGGTAAGCTCGCGAAACCCCGGCAGCTTC
ACAATACCCATTGGGGCCTGGTCTGCCCGGCCGAAACGCCCGAGGGTCAGGCCTGTGGTCTGGTGAAGAA
TTTGTCGTTGATGTGTTCGATCAGCGTGGGGACACCTACGGATCCTATAGTGGATTATATGATCACCAGGAA
TATGGAGGTTCTCGAGGAGTACGAACCCTTACGATACCCAGACGCTACTAAGATCTTCCTCAACGGCTCTTG
GATCGGTGTACACCAGAACCCCAAGGCTCTGGTAAGAGATGTCCAGAATTTGCGCCGGACAAATCAGATCC
CGGCCGAGGTATCGCTGATCCGCGACATACGCGATCGCGAATTCAAGATCTTTTCAGATGCGGGCCGGGTC
ATGCGACCCTTGTTTGTCGTGAATCAGGAGGAC---
ACCGAGGCTGGTATCAAGAAGGGTACCTTAGCTCTAACTAAGGAGATGATCCAGAGGCTTGAATCCGATGT
CGATCTGGATCCCGATAGCGAGGAGTACTTCGGCTGGCAGGGCTTGGTCAACGAAGGTGTCATCGATTATC
TCGACGCAGAGGAAGAAGAAACCGCTATGATCTGCATGACGCCCGAGGACCTAGAAACCTACCGCCAGAA
CAAACTCGGATACAACGTGTCCCAGGATAATG-GG---GATGAGATT------
AATAAGCGCCTGAGGACCAAGGTTAACCCGACTACGCACATGTACACCCACTGCGAGATCCATCCCAGCAT
GCTGCTGGGTATATGCGCGAGCATTATCCCGTTTCCGGATCACAATCAGGTA------------------------
TGCTCCCTTCCTAGCGTACCCTGTTA--
TCTACTATTAAC--

--CTTGCCATGTA--

--GTCCCCCGAA---------------

>H_hypomiltum_MUCL_51845

GCGCTGTATAGAATCCAATAAACATTTCGAGATCGCTCTAGCCGCTAAACCAGCTATCATCACGAACGGTCT
CAAATACTCACTCGCTACGGGAAACTGGGGCGACCAGAAGAAGGCCATGAGCTCGACAGCTGGTGTATCA
CAGGTCTTGAATCGATACACGTTT-----GCTTCCAC-
ACTTTCTCACTTGAGAAGAACCAACACACCCATTGGGCGTGACGGCAAGCTTGCGAAACCTCGACAGCTTCA
CAATACGCATTGGGGCCTAGTCTGTCCAGCAGAAACGCCTGAAGGCCAGGCCTGCGGTTTGGTGAAGAAC
CTGTCTCTGATGTGTTCCGTCAGTGTGGGGACAGCAACGGATCCTATAGTAGACTATATGATCACTAGGAAT
ATGGAAGTTCTTGAGGAGTACGAACCCATGCGATACCCTGACGCAACCAAGATTTTCCTCAATGGTTCCTGG
ATCGGTGTCCATCAGAACCCCAAGGCTCTAGTAAAGGACGTTCAGCATCTGCGCCGAACGAATCAGATCCC
GGCCGAGGTATCGCTAGTTCGCGACATACGTGATCGCGAGTTCAAGATCTTTTCAGATGCTGGTCGAGTCA
TGCGTCCTTTATTTGTTGTAAACCAAGAGGAT--
ACAGAAGCCGGCGCCAAAAAGGGTACCTTGGCTCTTACCAAGGATATGATTCAGAGGCTCGAGGCAGATG
TCGATCTTGATCCTGAGAGTGAGGAGTACTTTGGCTGGCAGGGCTTGGTCAGTGAAGGTGTCATCGACTAC
CTGGACGCCGAAGAAGAAGAAACAGCCATGATATGCATGACACCCGAGGACCTTGAGACCTACCGACAAG
CTAAACTCGGATACGATGTATCTCAGGACAATG-GA---GACGAGGTT------
AATAAACGACTGAAGACCAAGGTTAATCCGACAACGCACATGTACACGCACTGCGAGATCCATCCCAGCAT
GCTTCTCGGTATTTGTGCCAGTATTATCCCATTTCCAGATCATAACCAAGTA------------------------
TGTTAATTCCCTAAC---

>H_addis_MUCL_52797

CAAGCTATTCCGTAATATTGTTCGTCGGCTTACTGGTGAGATCACAAATCATCTCAAGCGATGCATCGAAGC
GAACAAGCATTTTGAGATTGCTCTGGCCGCTAAACCAGCTATTATCTCAAACGGTCTCAAGTACTCACTCGC
TACAGGAAACTGGGGTGATCAGAAGAAGGCCATGAGCTCGACAGCTGGTGTGTCGCAAGTCTTGAACCGA
TACACGTTC-----TCGTCGAC-
ACTTTCTCACTTGAGACGAACCAACACGCCTATCGGACGTGATGGAAAGCTTGCCAAACCTCGGCAGCTTCA
CAATACCCATTGGGGCTTGGTTTGTCCGGCAGAAACACCCGAGGGCCAGGCTTGCGGCCTGGTCAAGAACT
TGTCATTGATGTGTTCTATCAGTGTCGGTACCTCCACGGATCCTATCGTGGATTACATGATTACGAGAAACA
TGGAAGTTCTCGAGGAGTACGAACCCTTGCGATACCCCGATGCTACCAAGATCTTTCTCAACGGTTCCTGGA
TCGGCGTGCACCAAAACCCTAAGGCTCTAGTCAGAGATGTCCAGAACTTACGCCGAACGAATCAAATCCCA
GCCGAGGTGTCGTTAGTCCGAGATATACGTGATCGTGAATTCAAGATCTTTTCGGATGCCGGTCGAGTCAT
GCGTCCCTTGTTCGTCGTACATCAAGAGGAT---
ACTGATACTGGCGCCAAGAAAGGGACATTGGCTCTTACCAAGGAGATAATCCAGAGGCTTGAAGCGGATG
TAGATCTAGATCCCGACAGTGATGCGTACTTTGGCTGGCAGGGGTTGGTAAACGAAGGTGTCATCGACTAT
CTTGATGCCGAAGAAGAAGAGACAACCATGATCTGCATGACTCCTGAGGACCTTGAGACTTACCGCCAGGC
GAAGGCCGGTATGGAGGTAAACCAGGATAACG-GA---GACGAGGTT------
AATAAGCGTCTCCGAACCAAGGTTAATCCCACGACACACATGTACACTCACTGCGAGATTCATCCTAGCATG
CTTCTAGGTATTTGCGCGAGCATTATTCCGTTCCCGGATCATAATCAGGTA------------------------
TGTTGCTTCACCAATGCAAATTGGTA--
TTCATTGCTAAC--

--CGTGCCATGTA---

---GT-----------------------------

>H_cyclobalanopsidis_FCATAS2714

TAGTGCGTCGGCTTGTTCAAGAGATCACCACGCACCTTCGACGCTGCATCGAAAATAATAAGCACTTCGAGA
TCGCTCTAGCAGCCAAACCAGCGATTGTGACCAACGGTCTCAAGTACTCACTCGCCACAGGCAACTGGGGT
GATCAGAAGAAGGCGGCGAGTTCGACGGCCGGTGTGTCACAAGTCCTGAACCGATACACGTTC-----
GCTTCCAC-
TCTTTCTCATTTGAGACGAACGAACACTCCTATCGGAAGAGATGGAAAGCTCGCGAAACCCCGCCAGCTTCA
CAACACGCACTGGGGTCTAGTCTGCCCGGCCGAGACGCCCGAGGGACAGGCCTGTGGTTTGGTCAAGAAT
CTGTCGCTGATGTGCTCTATCAGTGTGGGCACGTCCACTGATCCAATCGTCGATTACATGATCACGAGAAAC
ATGGAAGTGCTCGAAGAGTATGAACCTTTGCGCTACCCCGATGCTACCAAGATCTTCTTAAACGGCTCCTGG
ATTGGCGTACACCAGGACCCCCAGGCGCTTGTGAAAGATGTGCAGCGTTTACGCCGCTCCGGCCAGATTCC
TCCTGAAGTGTCGCTAGTTCGAGACATTCGCGATCGCGAGTTCAAGATCTTCTCCGATGCTGGCCGTGTTAT
GCGTCCCTTGTTTGTTGTTCAGCAAGAGGGTGATAAGCCGTCGTCGTCGTCATCTGACGACAGTGAGGATG
AAGAAGAGAAGGAAGAGGATGAAGCTGACAAAGTCAAGGGAACTTTGGCGCTCACCAAGGAGATGATCC
AGAGGCTGGAAGCAGATAACGACCTCGATCCCGATAGCGAAGAGTACTTCGGTTGGCAAGGTTTGGTCGG
CGCTGGTGTCATCGACTACCTAGACGCTGAGGAAGAGGAGACGGCCATGATCTGCATGACACCCGAAGAT
CTGGAGATCTACCGTCGAACCAAGGCCGGTGACGAAGTCGACCAGGATAACG-GT---GACGAGATT------
AACAAGCGCCTGAAGACGAAGATCAACCCGACCACCCACATGTACACGCACTGTGAGATCCATCCTAGCAT
GCTGCTAGGTATCTGCGCGAGCATCATTCCG--

>H_eurasiaticum_MUCL_57720

GAAGGCCGCGAGCTCGACAGCCGGTGTATCACAAGTGCTGAACAGATACACATTC-----GCATCTAC-
CCTATCTCATTTGAGAAGAACGAACACGCCCATCGGCCGAGACGGCAAGCTTGCGAAGCCCCGACAGCTTC
ACAACACGCACTGGGGTCTAGTCTGCCCGGCCGAGACGCCCGAGGGACAGGCTTGCGGTTTGGTCAAGAA
TCTGTCGTTGATGTGCTCCATCAGTGTGGGCACTTCCACCGATCCTATCGTCGATTATATGATCACGAGAAAT
ATGGAGGTGCTCGAAGAATATGAACCGCTGCGTTACCCGGATGCCACAAAGATCTTCTTGAACGGTTCGTG
GATTGGTGTACACCAGGACCCCCAAGCGTTAGTTAAGGACGTGCAGCGTCTGCGCCGCTCCGGCCAGATTC
CTCCCGAAGTGTCACTAGTGAGGGACATCCGTGACCGTGAATTCAAGATCTTCTCCGATGCGGGTCGAGTC
ATGCGCCCCTTGTTTGTTGTCCAGCAAGAGGAT--
GAGGAAGGGACTGACGACGAGCCGGGCAAGGGCAAGGGAACCTTGGCTCTCACCAAGGACATGATCCAG
AGGCTGGAGGCAGACAACGAAGTCGACCCCAGCAGCGAAGAGTACTTTGGTTGGGAAGGCTTGGTTGGA
GCAGGTGTCATCGATTACCTAGATGCGGAAGAGGAGGAGACCGCCATGATTTGCATGACCCCCGAAGATCT
GGATATCTATCGCAGAACCAAGGCCGGCGACGAAGTCTATCAGGATAACG-GC---GAGGAGATT------
AACAAGCGCCTGAAGACGAAGATCAACCCGACCACTCACATGTACACCCACTGTGAGATTCATCCCAGCAT
GCTGCTAGGTATTTGTGCCAGCATCATTCCGTTCCCTGACCACAATCAGGTA------------------------
AGTGATTCACCTAA---

>H_fuscum_CBS_113049

CTTCGGCGCTGCATCGAGAACAACAAGCACTTCGAGATTGCTTTAGCAGCCAAGCCAGCCATCGTGACCAA
CGGTCTCAAGTATTCGCTCGCTACAGGCAACTGGGGTGACCAAAAGAAGGCGGCGAGCTCGACAGCCGGT
GTGTCGCAAGTGCTCAACAGATACACATTC-----GCGTCTAC-
TCTATCTCATTTGAGAAGAACGAACACGCCCATCGGCCGAGACGGCAAGCTTGCGAAACCCCGACAGCTTC
ACAACACGCATTGGGGTCTAGTCTGCCCGGCCGAGACGCCCGAGGGACAGGCTTGTGGTTTGGTCAAGAA
TCTGTCCTTGATGTGCTCCATCAGTGTGGGCACGTCCACCGATCCTATCGTCGATTATATGATCACGAGAAA
CATGGAGGTACTCGAAGAATATGAACCTTTGCGTTACCCGGATGCCACCAAGATCTTCTTGAACGGTTCGTG
GATCGGTGTACACCAGGATCCCCAAGCTCTAGTTAAGGACGTGCAGCGTTTGCGTCGCTCCGGCCAGATTC
CTCCTGAAGTGTCGCTGGTGAGGGACATCCGTGACCGTGAGTTCAAGATCTTCTCCGATGCTGGTCGAGTC
ATGCGCCCCTTGTTCGTTGTACAGCAAGAGGAT---
GAGGAAGGGACCGATGACGAACCGGGCAAGGGCAAGGGAACCTTGGCTCTCACCAAGGACATGATCCAG
AGGCTGGAGGCGGACAACGAAGTCGACCCTAGCAGCGAAGAGTACTTTGGTTGGGAAGGCTTGGTCGGA
GCGGGTGTCATCGATTACCTAGATGCGGAAGAGGAAGAGACCGCTATGATTTGCATGACCCCCGAAGACCT
GGACATCTACCGTAGGACCAAGGCCGGCGACGAAGTCTATCAGGATAACG-GA---GAGGAGATT------
AACAAGCGCCTGAAGACGAAGATCAACCCGACCACTCACATGTACACCCACTGTGAGATTCATCCCAGCAT
GCTGCTAGGTATTTGCGCCAGCATCATTCCGTTCCCGGATCACAACCAGGTA-----------------------
AGTGATTCGCCCCATGCATC--

>H_pseudofuscum_DSM112038

AGATCACCACGCATCTTCGGCGCTGCATCGAGAACAACAAGCACTTCGAGATTGCTCTAGCAGCCAAGCCG
GCGATCGTGACCAACGGTCTCAAATATTCGCTCGCTACAGGCAACTGGGGCGACCAAAAGAAGGCGGCGA
GCTCGACAGCCGGTGTGTCACAAGTACTTAACAGATACACGTTT-----GCGTCTAC-
TCTATCTCATTTGAGAAGAACGAACACGCCCATCGGCCGAGACGGCAAGCTTGCGAAGCCCCGACAGCTTC
ACAACACGCATTGGGGTCTCGTCTGCCCGGCCGAGACGCCCGAGGGACAGGCTTGCGGTTTGGTCAAGAA
TCTGTCCTTGATGTGCTCCATCAGTGTGGGCACGTCCACCGACCCTATCGTCGACTATATGATCACGAGAAA
CATGGAGGTGCTCGAAGAATATGAACCGTTGCGTTACCCGGACGCCACCAAGATCTTCTTGAACGGTTCGT
GGATTGGTGTACACCAGGATCCCCAAGCGCTAGTGAAGGACGTGCAGCGTTTGCGTCGCTCCGGCCAGATT
CCTCCTGAAGTGTCACTAGTGAGGGACATCCGTGACCGCGAGTTCAAGATCTTCTCCGATGCTGGTCGAGTC
ATGCGCCCCTTGTTTGTTGTTCAGCAAGAGGAC---------------------------------------
GAGGAAGGGACCGATGACGAGCCGGGCAAGGTCAAGGGAACCTTGGCTCTCACCAAGGACATGATCCAG
AGGCTGGAGGCGGACAACGAAGTCGACCCCAGCAGCGAAGAGTACTTTGGTTGGGAAGGCTTGGTCGGA
GCAGGTGTCATCGATTACCTGGATGCGGAAGAAGAAGAGACCGCTATGATTTGCATGACCCCCGAAGATCT
GGACATCTACCGCAGGACCAAGGCCGGCGACGAAGTCTATCAGGATAACG-GC---GAGGAGATT------
AACAAACGCCTGAAGACGAAGATCAACCCGACCACTCACATATACACCCACTGTGAGATTCATCCCAGCATG
CTGCTAGGTATTTGCGCCAGCATCATTCCGTTCCCGGATCATAACCAGGTA----------------------
AGCGATTCGCCCGACACATCT--

>H_fuscoides_MUCL_52670

GGCCTCTGTTGGCGAAGCTGTTCCGAAACATTGCGCGTCGGTTGGTTCAAGAGATCACCACGCATCTTCGG
CGCTGCATCGAGAACAACAAGCACTTCGAGATTGCTCTAGCAGCCAAGCCAGCGATCGTGACCAACGGTCT
CAAGTATTCGCTCGCTACAGGCAACTGGGGTGACCAAAAGAAGGCGGCGAGCTCGACAGCCGGTGTGTCA
CAAGTGCTTAACAGATACACGTTT-----GCGTCTAC-
TCTATCTCATTTGAGAAGAACGAACACGCCCATCGGCCGAGACGGCAAGCTTGCGAAGCCCCGACAGCTTC
ACAACACGCATTGGGGTCTAGTCTGCCCGGCCGAGACGCCCGAGGGACAGGCTTGCGGTTTGGTCAAGAA
TCTGTCGTTGATGTGCTCCATCAGTGTGGGCACGTCCACCGACCCTATCGTCGACTATATGATCACGAGAAA
CATGGAGGTACTCGAAGAATATGAACCGTTGCGTTACCCGGACGCCACCAAGATCTTCTTGAACGGTTCGT
GGATTGGTGTACACCAGGATCCCCAAGCGCTAGTGAAGGACGTGCAGCGTTTGCGTCGCTCCGGCCAGATT
CCTCCTGAAGTGTCGCTAGTGAGGGACATCCGTGACCGTGAGTTCAAGATCTTCTCCGATGCTGGTCGAGTC
ATGCGCCCCTTGTTCGTTGTTCAGCAAGAGGAT---
GAGGAAGGGACCGATGACGAGCCGGGCAAGGTCAAGGGAACCTTGGCTCTCACCAAGGACATGATTCAG
AGGCTAGAGGCAGACAACGAAGTCGACCCCAGCAGCGAAGAGTACTTTGGTTGGGAAGGCTTGGTCGGA
GCAGGTGTCATCGATTACCTAGATGCGGAAGAAGAGACCGCTATGATTTGCATGACCCCCGAAGATCT
GGACATCTACCGCAGGACCAAGGCCGGCGACGAAGTCTACCAGGATAACG-GT---GAGGAGATT------
AACAAGCGCCTGAAGACGAAGATCAACCCGACCACTCACATATACACCCACTGTGAGATTCATCCCAGCAT
GCTGCTAGGTATTTGCGCCAGCATCATCCCGTTCCCGGATCACAACCAGGTA-----------------------
AGCGATTCGCCCAACACATCTTGTAAG--
TATTTCCTGCTAAC--

---C-TGCTATGT---

>H_porphyreum_CBS_119022

--GCATCGAC-
TCTGTCTCATCTGAGAAGAACTAACACGCCTATCGGTAGAGACGGCAAGCTTGCGAAGCCCCGACAGCTTC
ACAACACGCATTGGGGTCTAGTCTGTCCAGCCGAGACGCCCGAAGGACAGGCCTGTGGTCTGGTCAAGAA
TCTGTCGTTGATGTGCTCTATCAGTGTGGGCACATCAACCGACCCCATCGTTGATTACATGATCACGCGAAA
CATGGAGGTGCTCGAAGAGTATGAACCACTACGTTACCCCGATGCCACAAAGATCTTCTTGAACGGCTCGT
GGATTGGTGTACACCAGGACCCGCAGGCTTTGGTCAAGGATGTGCAGCGTTTGCGCCGTACTGGCCAGATT
CCTCCTGAAGTGTCACTAGTTAGGGACATCCGTGACCGGGAGTTCAAGATCTTTTCCGATGCTGGTCGTGTC
ATGCGCCCCTTGTTTGTTGTTCAGCAAGAGGAC--
GAAGAGGGAACCGACGACGAACCCGGCAAGGGTAAGGGTACCTTGGCTCTCACCAAGGACATGATCCAGA
GGCTAGAGGCAGACAATGATGTCGATCCCAACAGCGAAGAGTACTTTGGTTGGGAAGGCTTGGTCGGAGC
AGGTGTCATCGATTATCTAGATGCGGAAGAAGAAGAGACCGCCATGATTTGCATGACCCCTGAGGATCTGG
AGATCTATCGCANGACCAAGGCCGGAGACGAAGTCTATCAGGATAATG-GC---GAAGAGATT------
AACAAGCGCCTGAAGACTAAGATCAACCCTACTACTCACATGTATACCCACTGCGAGATTCATCCTAGCATG
CTGTTAGGTATTTGCGCGTCGATCATTCCGTTCCCGGATCACAACCAGGTA------------------------
TGCGGTTGGCCCAATACATCAATTGAC---
TATTTATTGCTAA--

--

>H_vogesiacum_CBS_115273

AGCTGTTCCGAAGCATCGTGCGCCGCTTGGTCCAGGAGATCACGTCGTATCTCCGACGCTGCATCGAAACC

AACAAGCATTTCGAAATCGCTCTGGCCGCCAAACCCGCGATTGTCACAAACGGTCTCAAGTATTCCCTCGCC
ACCGGAAACTGGGGCGACCAGAAGAAAGCCATGAGCTCGACGGCCGGCGTGTCGCAGGTCCTGAACCGAT
ACACGTTC-----TCGTCGAC-
TCTTTCTCACTTGAGAAGAACGAACACGCCTATCGGCAGAGACGGCAAGCTTGCGAAGCCCCGACAACTGC
ACAACACGCACTGGGGTCTGGTCTGCCCGGCCGAGACGCCCGAGGGACAGGCCTGCGGTCTGGTAAAGAA
TCTGTCGTTGATGTGCTCCATCAGCGTGGGCACGTCGACCAATCCTATCATCGATTACATGATTACTAGAAA
TATGGAGGTTCTCGAGGAGTATGAGCCGACGCGCCACCCCGACGCTACCAAGATCTTCCTCAACGGCTCTT
GGATTGGTGTGCATCACAAACCCCAGAACCTAGTGCGAGATGTCCAGGCTTTGCGCCGCACCGGCCAGATC
CCCGCCGAGGTGTCGTTGGTTCGCGACATCCGTGATCGCGAATTCAAGATCTTTTCCGACGCCGGTCGAGTA
ATGCGCCCCTTGTTCGTCGTCGAGCAAGAGGTT--
GACGACAGCGGCATCAAGGACAAGGGAGTATTGGCTCTCACAAAGGACATGATCCAGAGATTGGAAGCCG
ATGTCGACATAGATCCCGAGAGCGAAGAGTACTTCGGCTGGCAAGGCCTGATCGACGCAGGTGTCGTCGA
TTATCTCGACGCGGAGGAGGAGGAGACGGCCATGATTTGCATGTCGCCGGAAGATCTCGACATCTACCGCC
GGACCAAGGCCGGCGAGGAGGTGTACCAGGACAATG-GG---GACGAGATT------
AACAAGCGCCTGAAGACCAAGATCAACCCCACGACGCATAGATATACCCACTGCGAGATTCACCCTAGCAT
GCTCTTGGGCATCTGCGCGAGCATCATTCCGTTCCCGGACCACAATCAGGTA----------------------
CGCGATGCGCCCGGCGCAACCTTCAAG--
TGTTCATTGCTA--

-

>H_crocopeplum_CBS_119004

--TCTCAACCGTTACACGTTC-----
GCCTCGAC-

CCTCTCGCATTTGAGGAGAACCAATACGCCTATTGGAAGAGACGGAAAGCTGGCCAAGCCTCGACAGCTGC
ACAACACTCATTGGGGCCTGGTCTGCCCGGCTGAAACCCCTGAAGGCCAAGCTTGTGGCCTTGTGAAGAAT
CTGTCGCTGATGTGCTCTATCAGCGTGGGTACTTCGACGGATCCTATTGTCGACTATATGATTACTAGGAAT
ATGGAAGTCCTAGAGGAGTACGAACCCATGAGATATCCTAACGCCACCAAGATCTTCCTGAACGGCTCGTG
GATCGGTGTACATCAAGACCCCAAATCTCTCGTCAGAGATGTTCAACAGCTGCGTCGGGCCAACCAGATCC
CATCCGAGGTCTCACTAGTCCGCGATATCCGTGATCGCGAGTTCAAGATCTTTTCGGATGCGGGCCGTGTCA
TGCGCCCCTTGTTTGTCGTACAGCAAGAGGAC---
GCTGGCGAGACCAAGGGCTGTTTGGCTCTCAACAAAGACATGATACAGAAACTCGAGGCAGACGTCGACA
TAGATCCCGAGAACGAAGAGTACTATGGCTGGCAGGGCCTAGTTAACGACGGCGTCATCGAGTACCTCGA
CGCAGAAGAGGAAGAGACAGCAATGATATGCATGACGCCTGAAGACCTGGAGAATTACCGGTTGACCAAG
GCCGGGTTTGATGTTGTTCAAGATAACG-GG---GATGAGATT------
AACAAACGCCTCAAAACCAAGGTCAACCCCACGACACACATGTATACTCACTGCGAGATTCATCCTAGCATG
CTCCTAGGCATCTGCGCCAGCATTATCCCCTTCCCGGATCACAACCAGGTA--------------------------
TTTCATTTCACTCACCTTT---
TGGTATTCCTGCTAAC--

---CTGCC---

>H_fendleri_MUCL_54792

CTATTCCGCANCATTGTTCGTCGTCTCACTCAAGAGATTACGAATCATCTTAGACGCTGTATTGAGACCAACA
GAAGATTCCAAATCGAGCTTGCTGCTAAGCCTGCCATCGTCACCAACGGACTGAAGTATTCACTCGCTACGG
GTAACTGGGGTGATCAGAAGAAGGCTATGAGTTCGACAGCTGGTGTATCTCAAGTTTTGAACCGTTACACA
TTC-----GCATCGAC-
CCTATCTCATTTAAGAAGAACCAACACTCCTATCGGAAGAGATGGTAAATTGGCCAAACCTCGACAACTTCA

CAATACTCATTGGGGTTTGGTCTGTCCGGCTGAAACCCCTGAAGGTCAAGCTTGTGGACTTGTGAAGAATTT
GTCACTTATGTGCTCCATCAGCGTGGGTACATCGACAGACCCTATCATCGACTACATGATTACCAGGAACAT
GGAAGTCCTTGAGGAATACGAACCCATGAGATATCCTAATGCTACCAAGATCTTCCTCAATGGTTCGTGGAT
CGGTGTACACCAAGATCCCAAGTCTCTTGTCAGGGATGTTCAGCAGCTACGTCGAGCCAACCAGATTCCCTA
CGAAGTTTCTCTTGTACGCGACATTCGTGATCGTGAGTTCAAGATTTTTTCGGATGCTGGTCGTGTCATGCG
TCCTTTGTTTGTCGTTCAACAAGAGGAT---TCCGATTCTGCCCA--

GAAAGGTTCTTTGGCCCTCACAAAGGACATGATACAGAGACTCGAGGCAGATGTTGACTTGGATCCTGAGA
GCGAGGATTATTTCGGATGGCAGGGTCTAGTCAACGAAGGTGTTATCGAATATCTCGATGCCGAAGAAGA
AGAGACTGCCATGATTTGCATGACACCTGAAGACTTGGAGAATTACCGATTGACCAAGGCTGGGGTTGATG
TTTACCAGGACAANG-GA---GATGAAATT------
AACAAACGTCTCAAGACCAAGGTCAACCCAACGACACATATGTATACTCATTGTGAGATTCATCCTAGTATG
TTGTTGGGTATTTGCGCCAGTATTATTCCATTCCCGGATCATAATCAGGTATGT----------------------
CCTAGTCCGTCCCGTCTGTCCTTTAACCCACTCA--
TCGCCCCTATTCTATCCATC--

--CACCTGATCCCCTGTCCCCCTTG----------------------------

ATCCCCCTGATCCAT--
--CCCCAACATTACCTCTTCA--
CTCCCAATATCATACTAACAAAG---

>P_hunteri_MUCL_52673

AACAGACGCTTCCAAATCGAGCTTGCTGCCAAGCCGGCCATAATCACCAATGGTTTGAAATATTCTCTAGCC
ACAGGCAACTGGGGCGACCAGAAGAAAGCCATGAGCTCCACGGCCGGCGTGTCGCAGGTCCTAAACAGAT
ATACGTTC-----GCCTCGAC-
CCTTTCCCATTTACGACGAACGAACACGCCCATCGGAAGGGACGGCAAGCTCGCGAAGCCGCGACAGCTAC
ACAACACTCATTGGGGTCTGGTATGTCCGGCCGAGACGCCCGAAGGCCAAGCCTGCGGGCTGGTCAAGAA

TCTGTCGCTTATGTGCTCCATCAGCGTGGGTACCTCAACGGATCCTATCGTAGATTATATGATCACCAGAAA
CATGGAGGTCTTGGAGGAATATGAGCCCATGAGATATCCTAACGCAACCAAGATCTTCCTCAACGGCTCCT
GGATCGGTGTGCACCAAGATCCCAAGTCTCTAGTTAGAGACGTTCAGCAGCTGCGCCGGGCCAACCAGATT
CCCTCCGAGGTATCTTTAGTTCGCGACATCCGAGACCGCGAGTTCAAGATTTTCTCAGACGCCGGCCGCGTC
ATGCGTCCCTTGTTTGTTGTACAGCAAGAGGAT--
GACCCGGACACCGGTGTCCCCAAGGGCCACCTGGCTCTCACGAAGACCCAGATTGCGAAGCTGGAGGCAA
GCATCGACGTAGAGGTCGACGCTCCCGGCTACTATGGCTGGCAAGGGTTAGTTAACGACGGTGTTATCGAG
TATCTCGATGCGGAGGAGGAGGAGACGGCTATGATATGCATGACGCCCGAAGACTTGGAAACATATCGCA
TGGCCAAGGCCGGCATTGATATGCCTCAGGACAACG-GG---GACGAGATC------
AACAAGCGCCTCAAGACCAAGGTTAACCCCACGACGCACATGTACACGCACTGCGAGATCCACCCGAGTAT
GCTTCTAGGTATTTGCGCTAGCATTATTCCCTTCCCAGACCATAATCAGGTA-----------------------
CGTAATTCTCCTAACCTCCCCTTCGG--

>P_nicaraguense_CBS_117739

AAGGCCAAGCCTGCGGGCTGGTCAAGAATCTGTCGCTTATGTGCTCCATCAGCGTGGGTACCTCAACGGAT
CCTATCGTAGATTATATGATCACCAGAAACATGGAGGTCTTGGAGGAATATGAGCCCATGAGATATCCTAA
CGCAACCAAGATCTTCCTCAACGGCTCCTGGATCGGTGTGCACCAAGATCCCAAGTCTCTAGTTAGAGACGT
TCAGCAGCTGCGCCGGGCCAACCAGATTCCCTCCGAGGTATCTTTAGTTCGCGACATCCGAGACCGCGAGT
TCAAGATTTTCTCAGACGCCGGCCGCGTCATGCGTCCCTTGTTTGTTGTACAGCAAGAGGAT------------------
----

GACCCGGACACCGGTGTCCCCAAGGGCCACCTGGCTCTCACGAAGACCCAGATTGCGAAGCTGGAGGCAA
GCATCGACGTAGAGGTCGACGCTCCCGGCTACTATGGCTGGCAAGGGTTAGTTAACGACGGTGTTATCGAG
TATCTCGATGCGGAGGAGGAGGAGACGGCTATGATATGCATGACGCCCGAAGACTTGGAAACATATCGCA
TGGCCAAGGCCGGCATTGATATGCCTCAGGACAACG-GG---GACGAGATC------
AACAAGCGCCTCAAGACCAAGGTTAACCCCACGACGCACATGTACACGCACTGCGAGATCCACCCGAGTAT
GCTTCTAGGTATTTGCGCTAGCATTATTCCCTTCCCGGACCATAATCAGGTA------------------------
CGTAATTCTCCTAACCTCCCCTTCGG--

>P_laminosus_MUCL_53305

CGGGCAACTGGGGCGACCAGAAGAAGGCCATGAGCTCCACGGCCGGCGTGTCGCAGGTCCTAAACCGATA
TACGTTC-----GCATCGAC-
TCTTTCTCATTTGCGACGAACGAACACGCCCATCGGAAGGGACGGCAAGCTTGCGAAGCCGCGACAGCTAC
ACAACACCCACTGGGGTCTGGTATGTCCGGCCGAGACGCCCGAAGGCCAGGCCTGCGGGCTGGTAAAGAA
TTTGTCGCTCATGTGCTCCATCAGCGTGGGTACCTCAACGGACCCGATCGTAGATTATATGATTACTAGGAA
TATGGAGGTCTTGGAGGAATATGAGCCGATGAGATATCCTAACGCCACCAAGATCTTCCTCAACGGCTCTT
GGATCGGCGTGCACCAAGATCCTAAGTCTCTGGTCAGGGACGTTCAGCAGCTGCGCCGGGCCAACCAGATC
CCCTCCGAGGTATCTTTAGTTCGAGACATCAGAGACCGCGAATTCAAGATTTTTTCGGATGCTGGCCGCGTC
ATGCGTCCCCTGTTCGTTGTACAGCAGGAGGAT---
GACCCCGTCACCGGCGTCCAAAAGGGCCACCTGGCTCTCACGAAGACCCAGATCGCGAAGCTGGAGGCAA
GCATCGACGTAGATGTCGATGCTCCTGGCTACTATGGTTGGCAAGGGTTGGTTAACGACGGTGTTATCGAG
TATCTCGATGCGGAAGAGGAGGAGACAGCTATGATATGCATGACGCCCGAAGACTTGGAAACCTATCGCAT

GACCAAGGCCGGCGTTGATTTGCCTCAGGACAACG-GG---GACGAGATT------
AACAAGCGCCTCAAGACCAAGGTCAACCCTACGACGCACATGTATACGCATTGCGAGATCCACCCGAGTAT
GCTTCTAGGTATTTGCGCTAGCATTATTCCCTTCCCAGACCATAATCAGGTA----------------------
CGTAATTCGCCTAGCCTTCCCTTCAGT--

>H_erythrostroma_MUCL_53759

AACATCGTTCGTCGTCTGGTCACCGAGATTACGCAGCACCTAAGACGCTGCATCGACCAGTCCAAGCGTTTC
CAGATCGAGCTTGCTGCTAAGCCTGCTATCGTCACGAATGGGCTGAAGTACTCCCTTGCCACAGGTAACTG
GGGTGACCAAAAGAAGGCAATGAGCTCGACCGCTGGTGTGTCCCAAGTTCTCAACCGTTACACGTTC-----
GCATCAAC-
CCTGTCACATTTGAGGCGAACGAACACTCCCATCGGACGAGACGGTAAACTGGCCAAGCCTCGGCAATTGC
ACAACACCCATTGGGGCCTAGTCTGCCCGGCCGAGACACCCGAAGGTCAGGCCTGCGGGCTGGTAAAGAA
TTTGTCGTTGATGTGCTCCATCAGCGTGGGCACATCGACAGATCCCATTGTCGACTATATGATAACCAGGAA
CATGGAAGTGCTTGAGGAGTACGAGCCGATGCGATACCCCAACGCGACCAAGATTTTCTTAAACGGATCTT
GGATCGGTGTCCATCAAGACCCCAAGTCACTCGTCCGGGATGTCCAGGCATTGCGTAGGAGCAACCAGATT
CCCGCCGAGGTGTCTTTGGTCCATGATATCCGTGATCGTGAATTCAAGATCTTCTCAGACGCCGGCCGTGTC
ATGCGCCCCTTGTTCGTCGTACAGCAAGAGGAT--AC------
GGAACAAGTGAAGAAGGGCTTTCTAGCCCTTAACAAAGATATGATACAACGGCTCGAGGCGGATTCCGAA
ACAGACCCTGACAGTGATGGCTATTATGGTTGGCAGGGTCTGGTCGACGATGGCGTCATCGAGTATCTCGA
CGCAGAGGAGGAAGAGACGGCCATGATATGCATGACTCCTGAAGACTTAGAGACATATCGAATGACAAAG
GCCGGCCAAGCTATTGCGGAAGATCACA-CA---GAGGAAACC------
AACAAGCGCCTCAAGACAAGGATCAATCCAACGACGCACATGTACACCCATTGCGAGATCCACCCCAGCAT

GCTTCTGGGCATATGCGCGAGTATTATTCCGTTCCCGGATCATAATCAGGTA---------------------
TGTTGTCTCTCTCCCTCTT---
GGTGTTCGATACTAAC--

--CTCATTTTCTA-----------------------------------

GTCGCCAGAATACG---

>H_howeanum_MUCL_47599

CATTTGAGAAGAACGAACACTCCTATTGGAAGAGACGGAAAGTTGGCGAAGCCCCGACAGCTTCACAACAC
GCACTGGGGTCTTGTCTGTCCAGCCGAGACGCCCGAAGGTCAGGCCTGTGGATTGGTCAAAAATCTGTCGT
TGATGTGCTCTATCAGTGTGGGTACATCGACCGACCCGATTGTAGACTATATGATCACTCGAAGCATGGATG
TTTTAGAGGAATATGAACCAAAGACCAATCCTAATGCTACGAAAATCTTCTTGAACGGCTCTTGGATTGGCA
CTCACACAGACCCCAAGGCTCTCGTCAGGGATATTCAGGAATTACGACGAGCTAACCAGATTCCATCTGAG
GTGTCATTGGTGCGCGACATTCGCGATCGCGAGTTCAAGATATTCTCTGATGCTGGTCGAGTTATGCGCCCA
CTATTTGTCGTTCAACGAGAGGAT---
GACCAGGAGAAGAGTATCGTCAAGGGGTCGTTGGCTCTCACAAAGGACATGATACAGAGGCTGGAGGCCG
ACAATGATCAAGATCCCGACAGTGAAGATTATTTTGGTTGGCAAGGACTAGTCAACGACGGTGCTATTGAA
TACCTCGATGCTGAGGAAGAGGAGACGGCGATGATTTGCATGACGCCGGAGGATCTCGATACGTTTCGTTT
GGCCAAGGCTGGTTATGATACGAACCAGGACAACG-GC---GACGAAATT------
AACAAACGGTTGAAAACCAAAGTCAACCCCACAACTCACATGTACACACATTGTGAGATCCATCCTAGCATG
CTCTTGGGAATCTGTGCCAGTATTATTCCCTTCCCAGATCACAATCAGGTA---------------------
TGCCACTCTTTCCCATCCCTTTTC---

--CCTATCTCGT--

>H_ticinense_CBS_115271

GGAATATTGTCCGTCGGTTGGTAACGGAGGTGTCACAGAATTTGAGACGATGCATCGACCAGAACAGACG
TTTCCAGATTGAGCTTGCAGCGAAGCCTGCCATCATCACGAATGGACTAAAGTATTCGCTCGCCACAGGAAA
TTGGGGTGATCAAAAGAAGGCGATGAGCTCGACTGCTGGTGTGTCTCAAGTTCTAAATCGATACACGTTC---
--GCTTCGAC-
CCTTTCTCATTTACGAAGAACGAACACCCCCATCGGAAGAGACGGAAAGTTGGCGAAGCCTCGCCAGCTTC
ACAACACGCATTGGGGTCTGGTCTGTCCAGCCGAGACGCCCGAGGGTCAGGCCTGTGGCCTGGTGAAGAA
TCTCTCGCTAATGTGCTCCATCAGCGTGGGTACTTCAACGGATCCTATCGTAGACTATATGATTACACGTAGC
ATGGACGTCTTGGAGGAATATGATCCAAAAACACGACCCAATGATACGAAAATCTTTCTGAATGGCTCCTG
GATTGGTACACACCAGGACCCTAAGGCCCTTGTTAGGGATATCCAGGAACTGCGACGATCCAACCAGATTC
CGTCCGAGGTATCCTTGGTCCGCGACATTCGTGACCGCGAATTCAAAATCTTCTCCGACGCTGGCCGCGTTA
TGCGCCCACTATTTGTCGTTCAACGAGAGGAT---
GACCCAGAGAAGCATATTGTCAAGGGATCATTAGCTCTTACAAAGGACATGATACAGAGGCTGGAAGCCG
ACGTTGATCAAGATCCCGAGAGTGAAGATTACTATGGCTGGCAAGGTCTAGTCAACGAAGGTGCCATTGAG
TATCTTGACGCTGAGGAGGAGGAAACGGCGATGATTTGCATGACACCGGAGGATCTGGAGACTTTCCGTTT
GACCAAGGCCGGCTATGAGGTGAGCCAGGACAACG-GC---GACGAGATC------
AACAAGCGGCTCAAGACCAAAGTCAATCCCACGACTCACATGTACACACACTGCGAAATTCATCCTAGCATG
CTCCTAGGAATCTGTGCCAGTATTATTCCATTCCCAGATCATAATCAGGTA-----------------------
CGCAGTCTACCCCTCCCTTCCATTCATCTCCGTACTA---

>D_rogersii_YMJ_92031201

GGCAAGAAGCGTCTGGACCTCGCGGGTCCGCTGCTCGCCAAGCTATTCCGTAATATTGTTCGCCGCTTAGTG
CAGGAGGTCAGCGGCCACCTCAAACGGTGTATCGATTCGAACCGGCGTTTCCATATCGAGCTTGCTGCTAA
GCCTTCCATCATCACGAACGGCCTGAAATACTCTCTGGCTACCGGCAACTGGGGCGACCAGAAGAAGGCGA
TGAGCTCGACCGCCGGTGTATCGCAAGTCCTTAATCGTTATACCTTC-----GCGTCGAC-
CCTGTCCCATCTGCGGAGAACCAATACGCCTATAGGACGCGACGGGAAGCTCGCGAAACCTCGGCAGCTTC
ACAACACCCACTGGGGGTTGGTTTGCCCAGCCGAAACACCAGAGGGCCAGGCCTGCGGGCTGGTCAAGAA
CTTGTCTTTGATGTGCTCCATCAGCGTCGGCACATCGACGGATCCTATCGTCGACTACATGATCACCCGAAA
TATGGAGGTTCTGGAGGAGTACGAGCCCATGCGATACCCCAACGCTACCAAGATCTTCCTCAACGGCTCTT
GGATCGGCGTACACCAAGATCCGAAGGCCTTGGTTAGAGACGTCCAGAACTTGCGCCGGGCCAATCAGAT
CCCGGCCGAGGTGTCCCTAATTCGCGATATTCGCGATCGCGAGTTCAAGATCTTTTCGGACGCCGGTCGGG
TCATGCGTCCCCTGTTCGTCGTCGAGCAGGAGGGCGAG---

AGGAAGGGGTCTTTGATTCTTACCAAGGACATGATCCACAGGCTAGAGGCGGACGTGGACCTATCTCCAGA
TAGCGATGACTATTTCGGCTGGCAGGGTCTGGTCAACGAGGGTGTCATCGAATTCCTAGACGCCGAGGAG
GAAGAGACGGCCATGATTTGCATGACGCCCGAGGATCTGGAAGCCTACCGTCAAGCCAAAGCCGGTTACG
CGCCGGTCGAGGACGACA-GC---GAGGAGATT------
AACCGGCGTCTCAAGACCAAGATGAACCCTACCACACACATGTACACACACTGCGAGATTCATCCGAGCAT
GTTGCTAGGTATCTGTGCTAGCATTATCCCGTTCCCCGACCACAACCAGGTTTGTCAAT-----------------
TCCCTTCAAAGTTGCCAGTCCTCTGTCCC---
GATAACTCTCTGCTAAC---

---CCTACCTCGCA--------------------------------------

GTCTCCTAGGAACAC---
---GTACCAATCGGCCA--

>D_rogersii_GMBC0204

GCAGGAGGTCAGCGGCCACCTCAAACGGTGTATCGATTCGAACCGGCGTTTCCATATCGAGCTTGCTGCTA
AGCCTTCCATCATCACGAACGGCCTGAAATACTCTCTGGCTACCGGCAACTGGGGCGACCAGAAGAAGGCG
ATGAGCTCGACCGCCGGTGTATCGCAAGTCCTTAATCGTTATACCTTC-----GCGTCGAC-
CCTGTCCCATCTGCGGAGAACCAATACGCCTATAGGACGCGACGGGAAGCTCGCGAAACCTCGGCAGCTTC
ACAACACCCACTGGGGGTTGGTTTGCCCAGCCGAAACACCAGAGGGCCAGGCCTGCGGGCTGGTCAAGAA
CTTGTCTTTGATGTGCTCCATCAGCGTCGGCACATCGACGGATCCTATCGTCGACTACATGATCACCCGAAA
TATGGAGGTTCTGGAGGAGTACGAGCCCATGCGATACCCCAACGCTACCAAGATCTTCCTCAACGGCTCTT
GGATCGGCGTACACCAAGATCCGAAGGCCTTGGTTAGAGACGTCCAGAACTTGCGCCGGGCCAATCAGAT
CCCGGCCGAGGTGTCCCTAATTCGCGATATTCGCGATCGCGAGTTCAAGATCTTTTCGGACGCCGGTCGGG
TCATGCGTCCCCTGTTCGTCGTCGAGCAGGAGGGCGAG--

AGGAAGGGGTCTTTGATTCTTACCAAGGACATGATCCACAGGCTAGAGGCGGACGTGGACCTATCTCCAGA
TAGCGATGACTATTTCGGCTGGCAGGGTCTGGTCAACGAGGGTGTCATCGAATTCCTAGACGCCGAGGAG
GAAGAGACGGCCATGATTTGCATGACGCCCGAGGATCTGGAAGCCTACCGTCAAGCCAAAGCCGGTTACG
CGCCGGTCGAGGACGACA-GC---GAGGAGATT------
AACCGGCGTCTCAAGACCAAGATGAACCCTACCACACACATGTACACACACTGCGAGATTCATCCGAGCAT
GTTGCTAGGTATCTGTGCTAGCATTATCCCGTTCCCCGACCACAACCAGGTTTGTCAAT-----------------
TCCCTTCAAAGTTGCCAGTCCTCTGTCCC---

>D_guizhouensis_GMBC0065

GTGCAGGAGGTCAGCGGCCACCTGAAACGGTGTATCGATTCGAACCGGCGTTTCCATATCGAGCTTGCTGC
TAAGCCTTCCATCATCACGAACGGCCTGAAATACTCTCTGGCTACCGGCAACTGGGGCGACCAGAAGAAGG
CGATGAGCTCGACCGCCGGTGTATCGCAAGTACTTAATCGCTATACCTTC-----GCCTCGAC-
CCTGTCCCATTTGCGGAGAACCAATACGCCTATAGGACGCGACGGGAAGCTCGCGAAACCTCGGCAGCTTC
ACAACACCCACTGGGGGTTGGTTTGCCCAGCCGAGACACCAGAGGGCCAGGCCTGCGGGCTGGTCAAGAA
CTTGTCTTTGATGTGCTCCATCAGCGTCGGCACATCAACAGATCCTATCGTCGACTACATGATCACCCGAAAT
ATGGAGGTTCTGGAGGAGTACGAGCCTATGCGATACCCCAACGCTACCAAGATCTTCCTCAACGGCTCTTG
GATCGGTGTACACCAAGATCCCAAGGCCTTGGTTAGAGACGTCCAGAACTTGCGCCGGGCCAATCAAATCC
CGGCCGAGGTATCCCTGATTCGCGATATTCGCGATCGCGAGTTCAAGATCTTTTCGGACGCCGGTCGGGTC
ATGCGTCCTCTGTTCGTCGTCGAGCAGGAGGGCGAG--

AGGAAGGGGTCTTTGATTCTTACCAAGGACATGATCCACAGGCTAGAGGCGGACGTCGACCTATCTCCGGA
TAGCGATGACTATTTCGGCTGGCAGGGTCTGGTCAACGAGGGTGTCATCGAATTCCTAGACGCCGAGGAG
GAAGAGACGGCCATGATTTGCATGACGCCCGAGGATCTGGAAGCCTACCGTCAAGCCAAGGCCGGTTACG
CGCCGGTCGAGGACGACA-GC---GAAGAGATT------
AACCGTCGTCTCAAGACCAAGATGAACCCTACCACACATATGTACACGCACTGCGAGATTCATCCGAGCATG
CTGCTAGGTATCTGCGCTAGCATTATCCCGTTCCCCGACCACAACCAGGTTTGTCAAT-----------------
TCCCTTCAAAGCTGCC--

>D_crateriformis_GMBC0205

GCATCGATTCGAACCGGCGTTTCCATATCGAGCTTGCCGCTAAGCCTTCCATCATCACGAATGGCCTGAAAT
ACTCTCTGGCTACCGGCAACTGGGGCGACCAGAAGAAGGCGATGAGCTCGACAGCCGGCGTATCGCAAGT
CCTCAACCGTTATACCTTC-----GCCTCGAC-
CCTGTCCCGTTTGCGGAGAACCAATACGCCTATTGGACGCGACGGAAAGCTCGCGAAACCTCGGCAGCTTC
ACAACACCCACTGGGGTTTGGTTTGCCCAGCCGAGACACCAGAGGGTCAGGCCTGCGGGCTGGTCAAGAA
CTTGTCGTTGATGTGCTCTATCAGCGTCGGTACATCGACGGATCCCATCGTCGACTACATGATCACCCGAAA
TATGGAGGTTCTGGAGGAGTACGAGCCTATGCGATACCCCAACGCCACTAAGATCTTCCTCAACGGCTCTTG
GATCGGCGTACACCAGGATCCCAAGGCCCTGGTTAGAGACGTCCAGAACTTGCGCCGGGACAATCAGATCC
CGGCCGAGGTGTCCCTGATTCGCGATATTCGCGATCGCGAGTTCAAGATCTTTTCGGACGCCGGTCGAGTC
ATGCGTCCCCTGTTCGTCGTCGAGCAGGAGGGCGAG---

AGGAAGGGGTCTTTGATTCTTACCAAGGACATGATCCACAGGCTAGAGGCGGACGTGGACCTATCTCCGGA
TGGCGATGACTATTTCGGCTGGCAGGGTCTGGTCAACGAGGGTGTCATCGAATTCCTAGACGCCGAGGAG
GAAGAGACGGCCATGATTTGCATGACGTCCGAGGATCTCGAAGCCTACCGTCAAGCCAAGGCTGGTTACGC
GCCGGTCGAGGACGATA-GC---GAGGAGATC------
AACCGTCGCCTTAAGACAAAGATGAACCCTACCACACATATGTACACGCACTGTGAGATTCATCCGAGCATG
TTGCTAGGTATCTGCGCTAGCATTATCCCATTCCCCGCCCA---

>D_comedens_YMJ_90071615

GGCAAGAAGCGTCTCGACCTCGCGGGTCCGCTGCTCGCCAAACTGTTCCGTAACATCGTTCGCCGCTTGGT
GCAGGAGGTCAGCGGCCACCTTAAGCGGTGCATCGATTCGAACCGGCGTTTCCACATCGAGCTCGCCGCCA
AGCCCTCTATCATCACGAACGGCCTGAAATACTCTCTGGCTACCGGCAACTGGGGTGACCAGAAGAAGGCG
ATGAGCTCGACCGCCGGCGTGTCGCAAGTGCTCAACCGTTATACCTTC-----GCCTCGAC-
CCTGTCGCATTTGCGGAGAACCAATACGCCTATAGGGCGCGATGGGAAGCTCGCGAAACCTCGGCAGCTTC
ACAACACCCACTGGGGTTTGGTTTGCCCAGCCGAAACACCAGAGGGCCAGGCTTGCGGGCTGGTCAAAAA
CTTGTCGTTGATGTGCTCCATCAGCGTCGGCACATCGACGGACCCCATTGTCGACTACATGATCACGCGAAA
TATGGAGGTCCTGGAGGAGTACGAGCCTATGCGATACCCCAACGCCACCAAGATCTTCCTCAACGGCTCTT
GGATCGGCGTACACCAGGATCCCAAGGCCTTGGTTAGGGACGTCCAGAACTTGCGCCGGGCCAATCAGAT
CCCGGCCGAGGTGTCTCTGATTCGCGATATTCGCGATCGCGAATTCAAGATCTTTTCGGACGCCGGTCGGGT
CATGCGTCCCCTGTTCGTCGTCGAGCAGGAGGGCGAG--

AGGAAGGGGTCTTTGATTCTTACCAAGGACATGATCCACAGACTGGAGGCAGACGTGGATATATCCCCGGA
CGCCGAGGATTATTTCGGCTGGCAGGGTCTCGTCAACGAGGGTGTCATCGAATTTCTCGACGCCGAGGAGG
AAGAGACAGCCATGATCTGCATGACGCCCGAGGATCTTGAAGCCTACCGTCAGGCCAAGGCCGGTTACGC
GCCGGTCGAGGATGACA-GC---GAGGAGATT------
AATCGTCGTCTGAAGACCAAGATGAACCCTACCACACATATGTACACCCACTGTGAGATCCATCCGAGCATG
TTACTAGGCATCTGCGCTAGCATCATCCCGTTCCCCGACCACAACCAGGTTTGTC----------------------------
GAGCTGCCAAGCCCTTCGTCTCGGT---
GACTCTCTGCTAAC---

--CCTGCCGCGCA--

---GTCTCCTAGGAACAC-

-----------------GTACCAGTCAGCCA---

>H_papillatum_ATCC_58729

--ATCGATATACTTTC-----
GCATCGAC-
TCTCTCCCATCTAAGGAGAACGAACACCCCCATCGGACGTGATGGGAAGCTTGCGAAACCCCGGCAGCTAC
ACAATACCCATTGGGGTCTAGTCTGTCCGGCGGAAACGCCTGAAGGCCAGGCCTGCGGGCTTGTAAAGAA
CTTATCGCTAATGTGCTCGATCAGTGTGGGTACGTCGACAGATCCCATCGTCGATTATATGATTACCCGAAA
CATGGAGGTTCTTGAAGAATACGAGCCTATGCGATATCCCAACGCTACCAAGATCTTCCTCAACGGTTCATG
GATCGGTGTGCACCAGGACCCCAAGGCTCTGGTCAGAGATGTCCAGAACCTTCGCCGGACCAATCAGATCC
CCGCTGAGGTGTCCTTAGTTCGGGACATCCGCGATCGTGAATTCAAGATCTTCTCGGATGCTGGTCGGGTTA
TGCGTCCCTTATTCGTCGTCGAACAGGAGGGTGAG---

AGGAAAGGGTCGTTAACCCTCACCAAGGAGATGATTCACAGGCTGGAGGCAGATGTAGACCTGCCGCAGG
ATAGTGAGGAGTACTTTGGCTGGCAAGGTCTAGTGAACGAGGGCGTCATCGAATTCCTAGATGCCGAAGA
AGAGGAGACAGCCATGATTTGTATGACGCCCGAAGACCTGGAAGCTTACCGTCAGGCCAAAGCCGGTTAT
GAGCCGGAGGAGAAGGAAG-CCCCTCAGGAGATT------
AACAGGCGACTGAAGACCAAGATGAATCCGACGACGCACATGTACACACACTGCGAAATCCATCCCAGTAT
GCTCTTGGGTATCTGCGCCAGCATTATCCCGTTCCCCGACCACAATCAGGTATGTGTCCCAACTC-------
CCAACCCAACCCAACCTGCCTTCCATTTAGCGTCGG---
----------CTGCTAAC--

--TCTGTCATGTA--------------------------------------

---G------------------------

>H_sp_MUCL_51392

TTGCAAACTCTTCCGCAACATAGTTCGTCGGCTAGTACAGGAGATTAGCGGTCATCTCAGGCGCTGTATTGA
TTCGAACAGACGCTTCCATATCGAGCTTGCTGCCAAGCCCGGCATCATCAGCAATGGTTTGRAATACTCCCT
AGCCACGGGCAATTGGGGCGACCAGAAGAAGGCGATGAGCTCAACCGCCGGTGTGTCGCAGGTCCTGAAT
CGATATACTTTC-----GCATCCAC-
GCTTTCCCATCTAAGAAGAACGAACACGCCCATCGGACGCGATGGGAAGCTTGCGAAACCCCGGCAACTGC
ACAACACCCATTGGGGCCTAGTCTGTCCGGCAGAAACGCCTGAAGGCCAGGCCTGCGGGCTTGTCAAGAA
CTTATCGCTAATGTGTTCGATCAGTGTGGGTACGTCGACAGATCCCATTGTCGATTATATGATTACCCGAAA
CATGGAGGTTCTTGAAGAATACGAGCCCATGCGGTATCCCAACGCTACCAAGATCTTCCTCAACGGATCATG
GATCGGTGTTCACCAGGACCCGAAGGCTCTGGTCAGAGACGTCCAGAACCTTCGCCGGACCAATCAGATCC
CCGCTGAGGTGTCCTTAGTCCGAGACATCCGTGACCGTGAATTCAAGATCTTTTCGGATGCCGGTCGTGTTA
TGCGTCCCTTATTTGTCGTCGAACAGGAGGGTGAG--
--
AGGAAAGGGTCGTTAATCCTCACCAAGGAGATGATTCACAGGCTGGAGGCAGATGTGGACCTACCTCAGG
ATAGTGAGGACTACTTTGGCTGGCAAGGTCTAGTGAACGAAGGCGTCATCGAATTTTTAGATGCTGAGGAA
GAAGAGACCGCCATGATTTGTATGACACCCGAAGACTTGGAAGCTTACCGTCAGGCCAAAGCCGGTTATGA
GCCGGAGAAAGAAAATA-CC---CAGGAGATT------
AACAGGCGACTCAAGACCAAGATGAATCCGACGACTCACATGTACACGCACTGCGAGATCCATCCAAGTAT
GCTCTTGGGTATCTGCGCTAGCATTATCCCATTCCCCGACCACAACCAGGTATGTGT-------------------
CTCAACCTAACCTGCCCTCTGTTTGGCATCGG---
--CTGCTAAC--

---TCTGTCATGTA--

---GTCCCT--AGAAAMC----------

--------GTACCATCGCT---

>H_chrysalidosporum_FCATAS2710

CGGAGTTTGTCTTGCTGGACCTC---
TTGGCCAGTTGTTCAGGAACATTGTCCGTCGATTCACGGGAGACATCACAACCCATCTCAGGCGCTGCATCG
ACTTGAATAAGCATTTCGAAATCGCACTTGCTGTCAAGCCGGCAATTATCACAAACGGACTGAAGTATTCGC
TCGCGACCGGAAATTGGGGTGATCAGAAGAAGGCGATGAGCTCGACTGCTGGTGTCTCTCAAGTCTTGAAC
CGATACACGTTC-----GCTTCGAC-
TCTCTCTCACTTGAGAAGAACCAACACGCCTATTGGAAGAGACGGCAAGCTTGCGAAGCCTCGACAGCTTC
ACAACACCCATTGGGGTCTAGTCTGTCCGGCTGAGACGCCGGAAGGACAGGCTTGTGGCCTAGTCAAGAAT
TTATCACTGATGTGCTCTGTCAGTGTGGGCACGTCGACCGAGCCTATCATTGATTATATGATAACGAGAAAT
ATGGAAGTTTTGGAAGAGTACGAACCTTTGCGTTACCCGGACGCTACTAAGATCTTTCTGAATGGTTCTTGG
ATCGGTATCCACCAAGATCCCAAGGCTCTCGTCGAGGATGTCCAAAACCTACGTCGCACGAACCAGATTCCC
GCCGAAGTATCCTTGTTCCGCGATATTCGAGATCGTGAATTCAAGATTTTCTCCGATGCCGGTCGAGTCATG
CGTCCCTTGTTCGTTGTGCAACAAGGAGAG--
AGCCCCGAGGATGGCATCAAAAAAGGAAAACTGGCCCTCACTAAAGAGATGATCCAGAGGCTGGAGGCAG
ACAACGACTTAGACCCCGATAGCGAAGAATACTTTGGATGGCAGGGCCTCGTCAATGAAGGAGCTATTGAT
TATTTGGATGCGGAAGAAGAGGAGACAGCCATGATCTGCATGACTCCAGAAGACCTGGATATCTATCGTCA
AGCCAAAGCCGGGCAAGACGTGTATCAGGATAACG-GT---GAAGAGGTC------
AACAAACGACTAAAGACCAAGATCAACCCTACAACCCACATGTACACCCACTGCGAAATCCACCCCAGCATG
CTGCTAGGTATTTGCGCGAGTATCATTCCATTCCCCGATC--

>H_lienhwacheense_MFLUCC_14_1231

aTGACCGACTACGACGAAGGTTACGAGTACGAGGAGGAGTATAGCGATGAGAGCTCGATCACGCCTGAAG
ATTGCTGGACGGTCATAGGCTCTTACTTCAAGAAGAAGGGCTTAGTTTCGCAACAAATCGATTCCTTCAATG
ATTTCCAGGAAACCACCATCCAGGAATTGATTGAAGAGTATTCGCAAATCAGCGTCGATCAGAACACCCCTG
TTT---

CTGAGGGTCAACCTGTCGCGCTACGCCGTTACGACCTGAAGCTTGGACATGTCACCATCGCGCGTCCTGTA
GCTATGGAAAGCGACAATACCTCAGGACCACTCTTGCCATACGAGTGTCGCGATCGAAATATGACGTACGC
GGCGCCGATCTATGTCAAAGTTGATAGTAAAGTGACTGCCTGTGTTGAACAGGATATTCCGCTCCACGAGA
TGGACGAGGAGCAGCAGGCTGAAATGGCAGAGACCGGGAAACATCCTACCCGTCTAGTATGGGAGGAAG
AGGAGAACGTTTTGGAGTCCCCTGCGACGGACAAGGGCAAGACCAGTGATCAGGTCTTCATTGGCAAGCT
ACCGATCATGGTGAAGTCGAAGGCGTGCCACTTGAGCACGGAGAGCACCGACGACCTCTTTCTACTGAACG
AGTGCCCTTACGATCAAGGTGGATATTTTATTATCAATGGCAGCGAAAAGGTTCTCATCGCTCAGGAGCGG
TCTGCGGCCAATATCGTTCAAGTCTTCAAGAAGGCCCAGCCGAGCCCGTATTCTTACACTGCCGAGATTCGA
AGTGCTCTAGAGAAGGGATCCCGTCTTATTTCCTCTCTGACGCTAAAGCTGTACTCAAAGGGCGATTCTTCG
CGAGGAGGTTACGGCGAGACAATTCACACCACTTTGCCCTTTGTCCGGGCCGATCTGCCGATCGCTATCGTT
TTCAGAGCTCTAGGTGTCGTCAGCGACGAGGAAATCCTCAATCATATCTGCTACGACCGCAACGATAGCCA
GATGCTCGAGAAGCTGCGTCCGTGTATTGAGGAGGCCTTCTGCATCCAGGATCGTGAGGTGGCCTTAGACT
ACATTGGAAAACGTGGGAGAGATTCCCACAG---
CGTGACACGAGAACGCCGTGTGAGGGCTGCGAAAGATATCCTGCAGAAAGAGATGCTTCCACACATTTCCC
AGACGGAAGGCTGCGAGACCAGGAAGGCTTTCTTCCTCGGTTATATGGTTCATAAGCTCTTGCAGTGCGCG
CTCGGCCGCCGTGATACCGACGATCGCGACCATTTTGGCAAGAAGCGTCTGGACCTTGCTGGGCCTCTCCTC
GCCAAATTGTTCAGGACTATTGTTCGCCGCCTGATGCTGGAAATTACCGGTCACCTCAAGCGGTGTATTGAA
TCGAACAAGCGCTTCCAGATCGAGCTTGCTGCGAAGCCGGCTATCATTACCAATGGGTTGAAGTACTCTCTC
GCTACAGGAAACTGGGGCGACCAGAAGAAGGCCATGAGTTCAACTGCGGGTGTGTCACAGGTACTGAATC
GATACACGTTC-----GCGTCGAC-
CCTTTCTCACTTGAGACGGACGAATACGCCTATTGGAAGAGATGGGAAGCTGGCGAAGCCCCGCCAATTGC
ACAATACACATTGGGGTCTCGTCTGTCCAGCCGAGACGCCCGAAGGTCAGGCTTGCGGTCTGGTGAAGAAT
TTGGCACTGATGTGCTCCGTAAGCGTGGGCACATCAACAGATCCCATCGTAGACTATATGATTACCAGGAAT
ATGGAGGTCCTAGAAGAATACGAGCCGATGAGATACCCTAATGCGACTAAAATATTCCTCAACGGTTCCTG
GATCGGAGTCCATCAGGATCCCCAGTCCCTGGTCAGGGATATCCAAAATCTGCGGCGGTCTGGCCAGATTC
CGGCTGAGGTGTCATTGGTTCGTGATATACGTGACCGCGAATTCAAGATTTTCTCAGATGCCGGTCGCGTCA
TGCGTCCCCTGTTCGTTGTACAACAAGAGGAC---
GGGCCAAACTCGACTAAGGGTACCCTAGCGCTCAATAAAGAAATGATACAAAGGTTAGAAGCAAGCGCAG
ATCTTGACCCAAATCATGAGGACTACTTCGGCTGGCAAGGTCTGGTCAACGAAGGTGTCATCGAATATCTC
GATGCGGAGGAAGAGGAGACGGCTATGATATGCATGACGCCCGAGGATCTGGAAACGTTCAGGATGGCC
AAAGCGGGACATAATATGTCTCAGGACATCG-GG---GATGAAATC------
AATAAGCGGTTAAAAACCAAGGTGAACCCTACGACGCACATGTATACACATTGCGAGATCCACCCAAGCAT
GCTCTTAGGCGTCTGCGCGAGCATTATACCCTTCCCAGACCATAATCAAGTAGGTGAACCCTAGCCCCTTGA
TTAGTATTTTGCTACTAACCTTGCCATAGTCACCTAGAAATAC----------------------------
ATATCAGTCAGCCATGGGTAAACAAGCCATGGGTTTATTCTTGACCAGTTTCGCACATCGTATGGATACGAT
GACGAATGTTCTCTTCTATCCGCAGAAACCACTCGGCACAACGCGGTCGATGGAGTTCTTGAAATTCAGAG
AACTTCCAGCTGGTCAGAACGCCATCGTAGCTATCCTGTGTTACTCGGGGTTCAACCAAGAAGATTCCATTA
TAATGAATCAGAGTAGTATAGACAGAGGTCTTTTCCGAAGTCTGTTCTTCCGATCCTATAACGACTGCGAGA
AACGAGTTGGGATAAATACGGTAGAGACGTTTGAGAAACCGTTCCGAGCGGACACGTTACGGCTCAAACA
AGGCACGTACGACAAGCTCGATGACGACGGTTTCGTGGCCCCCGGCTGCCGAGTGTCGGGCGAGGATATC
ATCATTGGAAAGACCTCCCCGATTAACCCAGATAACGAAGAGATGGGTCAGCGCACGAAGGTCCACGTCAA
ACGTGACGCGTCCACTCCCCTCCGTAGCACCGAAACCGGTATCGTTGATTCTGTTATTATCACGACGAACCC
AGACGGCCTGCGCTACGTGAAGGTACGAGTCCGGACGACGAAGATACCGCAGATCGGAGACAAGTTTGCC
TCCCGCCACGGGCAGAAGGGCACTATTGGTGTCACCTTCCGCCAAGAGGACATGCCTTTCACCTGCGAGGG

TATCGTTCCTGATATCATAATCAATCCGCACGCTATCCCCTCGCGTATGACGATTGCTCACTTAATCGAGTGT
CTGCTTAGCAAGGTCTCTACCCTTAAGGGAATGGAGGGAGACGCGACGCCGTTCACCGATGTCACTGTTGA
TTCAGTATCGAGCCTTCTCCGCGAGCACGGGTATCAGTCCCGCGGCTTCGAGGTCATGTACCATGGCCATAC
CGGACGCAAGCTCCGGGCTCAGGTCTTCTTCGGACCAACATACTACCAGCGTCTCAGACACATGGTGGACG
ACAAGATCCACGCACGAGCACGAGGCCCGGTGCAGATAATGACTCGTCAGCCCGTCGAGGGTCGCGCGAG
AGATGGAGGTCTCCGTTTCGGAGAAATGGAACGTGACTGCATGATTGCTCACGGTGCCGCTTCTTTCCTCAA
GGAGCGCCTCTTCGAGGTGTCGGATGCTTTCCGAGTACACGTCTGCGAGATCTGTGGTCTGATGACACCGA
TTGCGTAAGTTTTAGATCACCCTTTCCCCCTCTCCCACCCCTTTTTGCAGCAGCAGTAGCAGTAGCAGGCTTA
GTGCTAACATC----
ATGTAGTGTTCTAAGCAAAGGTTCGTTCGAGTGCCGACCGTGCAAGAATAAGACGAAGATCGCACAGGTG
CATATCCCGTATGCTGCCAAGCTCCTCTTCCAGGAGCTTCAGGCGATGAACATTGGGACTCGCCTGTTCACT
GATCGTGCCAGCGGCGGTATCCGTTAA

>H_rickii_MUCL_53309

CTGTTCCGGANTATCGTTCGACGGCTAGTCCAGGAGATTACGCAGCACCTACGACGTTGCATTGACCAGAA
TAGGCGGTTCCAGATCGAGCTTGCCGCGAAGCCGGCCATCATCACCAACGGCCTCAAGTACTCGCTCGCCA
CAGGCAACTGGGGCGATCAGAAGAAGGCCATGAGTTCGACCGCCGGTGTGTCCCAGGTGCTGAACCGATA
CACGTTC-----GCATCGAC-
ACTCTCGCATTTGCGACGAACCAACACCCCTATCGGACGTGACGGAAAGCTGGCGAAACCGCGACAGCTCC
ACAACACGCACTGGGGATTGGTGTGCCCGGCCGAGACGCCCGAAGGTCAGGCTTGTGGGTTGGTGAAGAA
TCTGTCCTTGATGTGTTCTATCAGCGTTGGGACATCGACCGACCCCATCGTAGACTACATGATCACTAGGAG
CATGGAAGTCTTGGAGGAGTACGAGCCGATGCGATACCCACACGCCACCAAGATCTTCCTGAACGGTTCCT
GGATCGGGGTGCATCAAGACCCGAAATCGCTGGTCCGAGATGTGCAGCAGCTCCGACGAGCTAACCAGAT
CCCATCCGAGGTGTCATTGCTCCACGATATACGGGATCGTGAGTTCAAGATCTTCTCGGATGCTGGTCGGGT
CATGCGACCCTTGTTCGTCGTGCAGCAAGACGAA--
GACCCCGAACGTCG---------
AGGAACGTTAGCACTTACCAAAGAGCATATTCAGCGATTGGAAACCGACAACGACTTGGATCCGGACAGTG
AAGAATACTTTGGTTGGCAAGGTTTGGTGAACGAAGGTGTTATTGAATACCTAGATGCGGAGGAAGAAGA
AACGGCGATGATCTGCATGACTCCGGAAGACCTCGAGACGTTCCGCTTAACGAAGGGCGGTTATGAAGTGT
CCCAAGACAACG-GT---GATGAAATC------
AACAAGAGGCTTAAGACGAAGATCAACCCTACGACACACACGTACACGCATTGCGAGATTCATCCCAGCAT
GTTGTTGGGTATTTGCGCCAGTATTATTCCTTTCCCAGACCACAACCAGGTA------------------------
TGTTCCATCATTCCCCCTCTCCCAGCCCTACTAA---

>Graphostroma_platystomum_CBS_270_87

AAGCGGAGTATCGAACAGAACCGGGGTTTCGCTATCGAGCTAGCTGTGAAGCCGACTATCATTACCAACGG
TCTGAAGTACTCGCTCGCCACCGGCAACTGGGGTGACCAGAAGAAAGCCATGAGCTCCACAGCTGGTGTTT
CGCAAGTGCTAAACCGATACACATTT-----TCCTCGAC-
GCTCTCTCACTTGAGACGTACAAACACTCCCGTAGGGAGAGATGGCAAGCTCGCCAAGCCACGGCAACTTC
ATAATACTCACTGGGGTCTTGTCTGTCCCGCCGAAACTCCCGAAGGCCAAGCTTGTGGCCTGGTGAAGAAC
CTGTCATTGATGTGCTCCATCAGCGTGGGCACATCGACAGAGCCCATTATCGATTACATGATTACCCGAAAC
ATGGAAGTGCTCGAGGAATACGAGCCCATGCGATATCCCCACGCCACCAAGATCTTCCTCAACGGCTCTTG
GATTGGTGTTCACCAGGACCCGAAGGCGCTCGTCAGGGATGTTCAGCAACTGCGCCGGAGCAATCAGATTC
CAGCAGAGGTGTCCCTAGTTCGCGACATCCGAGACCGCGAGTTTAAGATTTTCTCGGACGCTGGTCGCGTC
ATGCGACCCTTGTTTGTTGTCGAACAGGAGAGC---
GTGCCCGAGACGGGTGTCGAGAAGGGATCACTAGCTCTCAACAAGGACATGGTGAGACGACTTGAAATCG
ACCAAACGCTCCCTCCTGGAAGCGAAGAGTACTACGGCTGGCAAGGTTTGGTAAACGACGGTGTCATTGAA
TACCTTGATGCCGAGGAGGAAGAGACGGCTATGATATGCATGACGCCAGAAGATCTCGAGATCTATCGGA
GGACCAAGCTTGGTGAAGAGATTGTGAACGACAACG-GA---GACGATCTT------
AATAAGCGACTCAAGACAAAAATAAATCCAACCACGCACATGTACACGCATTGCGAAATTCATCCTAGTATG
CTTCTGGGTATCTGCGCCAGCATCATCCCCTTCCCTGATCACA---

>Natonodosa_speciosa_CLM_RV86

GATCACTTCGGCAAGAAGCGCCTGGATCTGGCCGGTCCCCTTCTCGGCAAGCTCTTCCGTAATATAGTGCGC
CGGATGACCCAAGAAGTCACCTCCAACCTGAAACGGTGCATCGAGCAGAACAAGACGTTCCAGATCGAATT
GGCTGTCAAGCCGGCCATCATCACCAACGGTCTCAAGTACTCTCTGGCCACGGGCAACTGGGGCGACCAGA
AGAAGGCCATGAGCTCTACGGCAGGTGTGTCTCAGGTGCTCAACCGATACACCTTC-----GCTTCTAC-
TCTCTCCCACTTGCGGAGAACCAACACGCCGGTGGGCAGAGACGGCAAGCTGGCGAAACCCCGTCAGCTAC
ACAACACGCATTGGGGCTTGGTCTGTCCTGCCGAGACGCCCGAAGGTCAGGCTTGTGGTCTGGTCAAGAAT
CTGTCACTCATGTGCTCTATCAGTGTAGGCACGTCGACCGAGCCTATCGAGGAGTACATGACCACCAGGAA
TATGGAAGTTCTCGAGGAGTACGAACCTAGTCGCTACCCGAACGCCACCAAGATCTTCTTGAACGGCTCCTG
GATCGGTGTGCACTCTGATCCGAAGGCACTGGTCAGAGATGTCCAGGAATTACGGCGAACGAACCAGATC
CCTGCTGAGGTATCCCTGGTCCGTGACATTCGCGACCGTGAATTCAAGATCTTCTCTGATGCGGGTCGGGTC
ATGCGGCCTCTGTTCGTGGTCGAGCAGGAGGAC---
AACCCGGAGACCGGCGTGGAGCAGGGTTCGCTGACATATACCCAAGAGATTGTCGAGAAGCTGAAGAACG
ACTTCAACGTGTACCCGGGACACGAAGAGTACTTTGGCTGGCAGGGTCTGGTGAACTCGGGTGTGATCGA
GTACCTAGATGCGGAAGAAGAGGAAACGGCGATGATCTGTATGACTCCGGAGGACCTGGAGATCTACCGC
GATTCCAAGAAGGGCATCGAAAAGGCCAACACCAATG-AA---GACGAACCT------
AATGCGCGTCTCAAGACGAAGATCAACCCCACCACTCACATGTATACGCACTGCGAGATCCATCCTAGTATG
CTGCTAGGTATCTGCGCCAGCATTATCCCCTTTCCGGATCATAACCAGGTATGCATTCT----------------
TGCCCCTTAGCCCGCGTTTTGACCCACACTGACATATTC--
GCAGTCTCCTCGTAAC--

---ACTTACCAGTCTGCCATGGGCAACAA--------------------

>X_arbuscula_CBS_126415

ATTGTTCGGCGGATGACCCAGGAGGTTCTGTCGCACCTGAAGCGGAGCATCGAGCAAGGCAAGCAATTCA
ATATTGCACTAGCTGTGAAGTCTAACATAATCACGAGTGGGTTGAAGTACTCACTCGCTACCGGCAACTGG
GGTGACCAGAAAAAGGCAATGAGCTCCACAGCCGGTGTTTCGCAAGTGTTGAATCGATACACATTC-----
GCCTCTAC-
CTTGTCACACTTGCGAAGAACAAATACCCCAGTCGGTAGAGATGGCAAGCTTGCCAAACCCCGACAACTTC
ACAATACCCACTGGGGGCTTGTCTGTCCAGCCGAGACCCCAGAAGGACAGGCATGTGGTTTGGTCAAAAAC
TTGTCGCTCATGTGCTCTATCAGCGTCGGTACATCAACGGATCCTATTATAGAATATATGATCCTTAGGAATA
TGGAAGTGCTAGAAGAGTATGATCCTGGTAGGTATCCCAACGCCACCAAGGTGTTTCTTAATGGTGCATGG
ATCGGCGTCCACCAGGATCCCAAGGCTCTAGTTAAGGATGTGCAACAATTGCGCCGAACAAACCAGATCCC
AGCTGAAGTATCCCTCATCCGGGATATTCGCGACCGTGAGTTCAAGATTTTCAGTGACGCCGGTCGCGTCAT
GCGCCCTCTGTTCGTAGTCGAGCAAGAAGAC--
GACGCTGAAAGGGGCATTGAGAAAAGCACGCTCGTTTTGACCAAAGATATGGTTCGGCGGCTTGAGGAAG
ACCAGAGCCTTCCACCCGGACACGAAGATTACTATGGATGGCAAAGTTTGGTTAATGCCGGTGTGATTGAA
TATATGGACGCTGAAGAAGAGGAGACGGCGATGATCTGCATGACCCCGGAAGACCTAGAGAGTTTCCGAT
GCAGCAAGTTGGGTCTAGCAGATCCTCACAACAACG-AT---
GATGTCTTTGCTCCCAACAAGCGGCTGAAGACGAGGATAAATCCGACCACACACATGTACACTCACTGTGA
AATTCATCCGAGCATGCTTCTAGGCATTTGTGCCAGTATCATTCCCTTCCCGGATCACAACCAAGTAAGTATC
AG---------------GATAACT--
ATGCTTATGCCAACTGC--

>X_hypoxylon_CBS_122620

TTGTCGCATTTGCGAAGAACGAATACTCCAGGTGGTAGAGACGGTAAGCTTGCGAAACCACGGCAGCTCCA
CAACAGCCACTGGGGTCTCGTCTGCCCGGCCGAGACCCCCGAAGGTCAGGCTTGTGGCCTAGTCAAAAACC
TGTCCCTCATGTGCTCCATCAGTGTTGGTACGTCCACGGAACCTATTATCGATTATATGATATCGCGTAATAT
GGAGGTTTTAGAGGAGTACGACCATCACAGATACCCTAATGCCACCAAGATCTTTCTCAATGGTGCATGGA
TCGGCGTCCACCAGGATCCTAAGTCTCTTGTGAGGGATGTGCAACAATTGCGCCGAACGAATCAGATTCCC
GCTGAAGTATCCTTGATTCGAGATATTCGTGACCGCGAATTCAAAATCTTCTCGGATGCCGGCCGTGTCATG
CGGCCCCTGTATGTGGTTGAGCAGGAGGAT--
GACGCTGAGAATGGTGTCGAGAAGGGCACACTGGTCTTAACAAAGGACATGGTTCGGAGACTTGAGATCG
ACCAGACCCTCCCACCCGGAAGTGACGAATACTTCGGATGGCATGGCCTGGTTCATGCTGGTGTCATCGAA
TACATGGATGCTGAAGAGGAGGAGACTGCGATGATTTGCATGACCCCCGAGGACTTGGAAAGTTTCCGAT
GCTCCAAGTTAGGTTTACCAGATCCTTTCAACAACG-AG---
GACACTTTCGCCCCAAACAAGCGGCTGAAGACGAGAATAAACCCGACAACCCATATGTACACTCACTGTGA
AATCCACCCGAGCATGCTTCTTGGCATTTGTGCCAGTATCATCCCCTTCCCCGATCATAATCAAGTAAGTGCC
AC----------------CGCAATCCGTATCGC---
CGCAATTATTGATCCCAGTTACA---

GTCACCCCGAAACAC--

---ATACCA---

>H_lenormandii_CBS_119003

--ACACGTTC-----
GCATCGACTCCTTTCACATTTGAGAAGAACCAACACACCTATTGGAAGAGATGGTAAGCTGGCGAAACCTC
GCCAGCTACACAATACCCATTGGGGTCTGGTCTGCCCAGCCGAGACTCCCGAAGGTCAGGCCTGTGGTTTG
GTGAAGAATCTGTCATTGATGTGCTCGATCAGTGTGGGTACCGCGACCGACCCCATTATAGAATACATGATT
ACTAGAAACATGGATGTTCTTGAGGAGTACGAGCCAATGCGCTATCCCAACGCAACCAAAATCTTCCTCAAC
GGCTCCTGGATTGGTGTACACCAGGATCCAAAATCTCTTGTCAAGGATGTTCAACAGCTGCGTCGCGCCAAC
CAGATTCCATCCGAGGTTTCGCTCATCCGCGACATCCGTGATCGCGAGTTCAAGATCTTTTCAGACGCGGGC
CGTGTCATGCGACCATTATTTGTTGTAAACCAAGAAAAT---
AATCCCGACAAGGGTGTGGAAAAGGGAACTCTGGTCCTTGACCAGGATATGCTCGGCAAACTCGAGCGCG
ATGTTGAGCTGGATCCAGATAGCGATGAGTACTACGGGTGGCAGGGCCTTGTCAATCAGGGCGTCATCGA
GTATCTGGATGCGGAGGAAGAAGAAACCGCGATGATCTGTATGACACCCGAAGACTTGGAAAATTACCGG
TTGATCAAGGCTGGATATGATATAGCTCAGGACAACG-GT---GACGAGATT------
AACAAGCGTCTCAAGACCAAGGTGAATCCTACGACACATATGTACACCCATTGCGAGATCCATCCCAGTATG
CTTCTGGGTATCTGCGCCAGTATCATTCCTTTCCCGGACCACAATCAAGTA------------------------TGCGAC-----
------CCCCTCTCATCCTTTCA--
GATCTTCTACTAACTGAA---

--TATGTA--

Table S6. Alignment of the *tub2* sequences used in the phylogenetic study.

>A_annulatum_CBS_140775

-----AAACGGCCCCTGA-----ACGCG-TC----------------AAAAA-------------CTC---CAAA---ACCCC-TTGA------T-
-----T-CCTGCCCCT-----CACGCAC---------AGA---AAA--
AAACAACACAA-----------------CGTCGCATTC------TATGT----TT-----------AACA---TCGCC-----
ATCACCATCAAG-TCG-----AG------------C---GAT------A--G------------------------TGATAT-------------
TCCACATCAGATAGCTAA-CCGTG---TT---TTTTCAT------------
CCAAATAGGTTCACCTTCAGACCGGCCAGTGCGTAAGTACTATAGC--------------TCC-TACGCCC----
GACGAAGAATC-----------------GCGACG----------------GAGT-ATAGCGG-----GGCTCAC--GA---A-TAT-----
TA-TAGGGTAACCAAATTGGTGCTGCTTTCT--
-GGCAAACCATCTCTGGCGAGCACGGCCTCGACAGCAATGGCGTGTAAGTACCT-----------------------
GAATCGTC-------------------AATTCGCAACGCC-------AAGAA------------------------AAAAAC--AAAC-------
TGAC-----------CGC-CAAT-
AAATAGCTACAATGGAACCTCGGAGCTCCAGCTCGAACGCATGAGCGTTTACTTTAACGAGGTACGCAAC-
CAGGG-----------------A----------A----ACA-----CA-TGGC--------------------CTGTTC-CC-A--------GGAGTAG-
TT-A-----CTAAT--CAC-CC-CAA---CATGC-
ACAGGCATCTGGTAACAAGTATGTTCCCCGAGCCGTCCTCGTCGACCTCGAGCCGGGTACCATGGACGCCG
TCCGTGCTGGGCCTTTCGGCCAACTTTTCCGACCCGACAACTTCGTTTTCGGCCAGTCTGGTGCCGGAAACA
ACTGGGCGAAGGGTCACTACACCGAGGGAGC-TGAGCTGGTTG-
ACCAGGTTCTTGATGTCGTTCGTCGTGAGGCCGAGGGCTGTGACTGCCTCCAGGGTTTCCAGATCACCCACT
CTCTCGGCGGTGGTACCGGTGCCGGTATGGGTACTCTGCTGATCTCCAAGATCCGCGAGGAGTTCCCCGAC
CGCATGATGGCCACCTTCTCCGTCGTTCCCTCTCCTAAGGTTTCCGACACCGTCGTCGAGCCTTACAACGCCA
CTCTCTCAGTCCACCAGTTGGTTGAGAACTCGGATGAGACGTTCTGTATCGACAACGAAGCTCTGTACGACA
TCTGCATGCGTACTCTCAAGTTATCCAACCCCTCTTACGGCGACTTGAACCACTTGGTTTCCGCCGTCATGTC
CGGTGTCACCACTTGCCTGCGTTTCCCCGGTCAGCTGAACTCTGACCTGCGCAAGCTTGCCGTGAACATGGT
TCCTTTCCCCCGTCTTCACTTCTTCATGGTCGGCTTTGCTCCCCTGACCAGCCGTGGCGCTTACTCTTTCCGCG
CCGTTACCGTTCCCGAGTTGACGCA-GCAGAT-
GTTCGATCCCAAGAACATGATGGCCGCGTCCGACTTCCGCAACGGCCGCTACCTAACGTGCTCCGCCATCTT
GTAAGATAAC-AC----CATTGTAT-----------TGATT--------------------------TTTAATGGG-CAA---
CATGCTAATTCG----AACCTTGTAGCCGTGGCAAGATCTCCA--

>A_truncatum_CBS_140778

--TGCCCCT-----
CACGCAC---------AGA---A--AAACACCACAA-----------------
GATCCTATTC------TCTGT----GT-----------AACA---TCAGC-----ATCAGCATCAAA-TCG-----AA------------C---
AAC------C--A------------------------TGATAT-------------TCCAGATCAGATAGCTAA-CCGTG---TT---TTTTCAC--
----------CTCGATAGGTTCACCTCCAGACCGGCCAGTGCGTAAGTACTACAGC--------------TAC-AACCCCC----
GACGAAGAATC-----------------GCGATG----------------CAAT-ATAGCGG-----GGCTCAC--GA---A-TAT-----CA-
TAGGGTAACCAAATCGGTGCTGCTTTCT--

GGCAAACCATCTCTGGCGAGCACGGTCTCGACAGCAATGGCGTGTAAGTATCT----------------------
GAATCGTC-------------------AATTCGCGACGCC-------AAGAA------------------------AAAAAC--AAAC-------
TGAC-----------CGC-CAAT-
AAACAGCTACAATGGAACCTCGGAGCTCCAGCTCGAACGCATGAGCGTTTACTTTAACGAGGTACGCAAC-
CAGGG-----------------A----------A----ACA-----CA-TGGT--------------------ATATCC-CG-A--------GGAGTAG-
TT-A-----CTAAT--CAC-CC-CAA---CATGC-
ACAGGCATCTGGTAACAAGTATGTTCCTCGAGCCGTCCTCGTCGACCTCGAGCCGGGCACCATGGACGCCG
TCCGTGCTGGCCCGTTCGGCCAACTTTTCCGACCCGACAACTTCGTCTTCGGCCAGTCTGGTGCCGGAAACA
ACTGGGCGAAGGGTCACTACACTGAGGGTGC-TGAGCTTGTTG-
ACAACGTTCTCGATGTCGTTCGTCGTGAGGCTGAGGGCTGTGACTGCCTTCAGGGTTTCCAGATCACCCACT
CTCTCGGTGGTGGTACCGGTGCCGGTATGGGTACTCTGCTGATCTCCAAGATCCGTGAAGAGTTCCCCGAC
CGTATGATGGCCACCTTCTCCGTCGTTCCTTCTCCTAAGGTTTCCGACACCGTCGTCGAGCCTTACAACGCCA
CTCTTTCAGTTCACCAGTTGGTTGAGAACTCGGACGAGACTTTCTGTATCGACAACGAAGCTCTGTACGACA
TCTGCATGCGTACTCTCAAGCTGTCCAACCCCTCTTACGGCGACTTGAACCACCTGGTTTCCGCCGTCATGTC
AGGTGTCACCACTTGCTTGCGTTTCCCTGGTCAGCTGAACTCTGACCTGCGCAAACTCGCCGTGAACATGGT
TCCTTTCCCTCGTCTTCACTTCTTCATGGTCGGCTTCGCTCCCTTGACCAGCCGTGGCGCTTACTCCTTCCGCG
CCGTTACCGTCCCCGAGTTGACGCA-ACAGAT-
GTTCGACCCCAAGAACATGATGGCCGCGTCCGACTTCCGCAACGGCCGCTACCTAACGTGCTCTGCCATCTT
GTAAGATAAC-GC----CTTTCTAT-----------TAATC--------------------------TTTGATAAG-CAAG--
TATGCTAATTC---

>J_cohaerens_CBS_119126

TACTGCTGACATGATCCTCCCCATCCCCATGCCCCTGA-----ACGCG-TCCT--------------GATCC-------------
TCC---AAAA--GCCCCC-TTGA------T------T-TCTGCCCCT-----CACGCAC-AGAAACAAAAA-----------------------
------------------------------AAA--CACCACCATA-------------------------TTC------CATCG----TT-----GTT---TACT---
TCAC------GCCAGCTTCAAATTTA-----CA------------C---AAT------C--G------------------------TGACAT------------
-TCTCGATCAATAAGCTAA-CCATA---TC-TTTTTTCAT------------
CCCAATAGGTTCACCTCCAGACCGGCCAATGCGTAAGTTTTATTACTTGGACT-----------AACTACA----
GACGAGATATC------------------GCGCTGGGATATAAGAATATAGGAAT-ATGGCGG-----GGCTAATATGA---
A-GAT-----GG-TAGGGTAACCAAATTGGTGCTGCTTTCT--
---------------GGCAAACCATCTCTGGCGAGCACGGCCTCGACAGCAATGGCGTGTAAGTATCT------------------
-----GAGTCGTC-------------------AATTCGCAAGGCC-------AAGGA---------------------------TATG-CAAC-------
TGACA----------AAC-CAAT-
AAACAGCTACAACGGAACCTCTGAGCTCCAGCTTGAGCGCATGAGCGTCTACTTCAACGAGGTACGCAAT-
CCAAG-----------------A----------A----ACC-----GATTCAT--------------------AGATGC----A-------AGGAGTAG-
TT-A-----CTAAT--CAC-CC-CAA---CATAC-
ACAGGCATCTGGTAACAAGTATGTTCCTCGCGCCGTCCTCGTCGACCTCGAGCCCGGCACCATGGACGCCG
TTCGCGCCGGTCCTTTCGGTCAGCTTTTCCGACCTGACAACTTCGTTTTCGGCCAGTCTGGTGCCGGAAACA
ACTGGGCCAAGGGTCATTACACCGAGGGTGC-TGAGCTTGTCG-
ACCAGGTTCTTGATGTCGTTCGTCGCGAGGCTGAGGGATGTGATTGCCTCCAGGGTTTCCAGATCACCCACT
CCCTCGGTGGTGGTACCGGTGCCGGTATGGGAACCTTGTTGATCTCCAAGATCCGCGAGGAGTTCCCCGAC
CGAATGATGGCTACCTTCTCCGTCGTTCCCTCTCCCAAGGTCTCCGACACCGTCGTCGAGCCCTACAACGCCA

CCCTGTCCGTCCATCAGCTGGTCGAGAACTCGGACGAGACCTTCTGCATTGACAACGAGGCTCTCTACGACA
TCTGCATGCGCACGCTGAAGCTGTCTAACCCTTCGTACGGTGACCTGAACCACCTGGTCTCCGCCGTCATGT
CTGGTGTCACCACCTGCTTGCGTTTCCCCGGCCAGCTGAACTCTGACCTGCGCAAACTCGCCGTGAACATGG
TTCCTTTCCCCCGTCTCCATTTCTTCATGGTCGGCTTTGCTCCCCTGACCAGCCGTGGCGCCTACTCTTTCCGT
GCCGTTACCGTTCCCGAGTTAACTCA-GCAGAT-
GTTTGACCCCAAGAACATGATGGCTGCTTCTGACTTCCGCAACGGTCGCTACCTGACGTGCTCTGCCATCTT
GTAAGATAAA-TA----CACGATACGTT-C--CTACCGGTC-------------------------CGTGATACC-TAA---
TTTGCTAACCCG---AAACTTTTTAGCCGTGGCAAGATCTCCATGAAGGAGGTCGAGGACCAGATGC-----------
----

>J_multiformis_CBS_119016

---TGATCCCCATCCTATCC--
CATCCCATCCCTACACCCAAATTTTATATTGCCCCTGA-----ACGCG-TCCC-------------GAAAC-------------
TCCAAGAAAA--AAACCC-TTGA------T------T-CCTGCCCCT-----CACGCAC-AGTAAAACACT--------------------
---------------------------------AAA--CACTGCCGTA------------------------TTT------CGCAA----TT-----GTT---
TACT---TCAC------ATCATCTTCAAA-CTA-----CG------------C---AAC------G--A------------------------TGACAT---
----------GCTCGATCAACATGCTAA-CCATA---TCTTTTTTTCAT------------
CTCGATAGGTTCACCTCCAGACCGGCCAATGCGTAAGTTTTACCGC--------------CTC-
GACCACGGACGGACGCAACGTC-----------------GCGCGG----------------GAAT-ATAGCGG-----
GGCTAACGTGA---A-GAT-----GG-TAGGGTAACCAAATTGGTGCTGCTTTCT--------------------------------------
----
GGCAAACCATCTCTGGCGAGCACGGCCTCGACAGCAATGGCGTGTAAGTATTT----------------------
TAGTCGTC-------------------AATTCGCAACGCC-------AAGGA---------------------------TATG-CAAC-------
TGAC-----------AGC-CAAT-
AAACAGCTACAATGGAACTTCGGAGCTCCAGCTTGAGCGCATGAGCGTCTACTTCAACGAGGTACGCAAT-
CCAAG-----------------A----------A----ACC-----CA-CGAT--------------------AGCATC-CA-A--------GGAGCAG-
TT-A-----CTAAT--CAC-CC-CAA---CATAC-
ACAGGCATCTGGTAACAAGTATGTTCCCCGCGCCGTCCTCGTCGATCTCGAGCCCGGCACCATGGACGCCG
TTCGTGCCGGTCCTTTCGGCCAGCTTTTCCGACCTGACAACTTCGTCTTCGGCCAGTCCGGTGCCGGAAACA
ACTGGGCCAAGGGTCATTACACTGAGGGTGC-TGAGCTTGTCG-
ACCAAGTTCTCGATGTCGTCCGTCGCGAGGCTGAGGGCTGTGACTGCCTCCAGGGTTTCCAGATCACCCACT
CCCTCGGTGGTGGTACCGGTGCCGGTATGGGAACCCTGTTGATCTCCAAGATCCGCGAGGAGTTCCCTGAC
CGAATGATGGCTACCTTCTCCGTCGTTCCCTCTCCAAAGGTCTCCGACACCGTCGTCGAGCCTTACAACGCCA
CCCTCTCCGTCCATCAGCTAGTCGAGAACTCCGATGAGACCTTCTGCATCGACAACGAGGCCCTCTACGACA
TCTGCATGCGCACGCTGAAGCTGTCTAACCCCTCGTACGGTGACCTGAACCACCTGGTCTCCGCTGTCATGT
CTGGTGTCACCACCTGCTTGCGTTTCCCCGGCCAGCTGAACTCTGACCTGCGCAAACTCGCCGTGAACATGG
TTCCCTTCCCCCGTCTCCATTTCTTCATGGTCGGCTTCGCTCCTCTGACCAGCCGTGGCGCTTACTCCTTCCGC
GCCGTCACCGTTCCCGAGTTGACTCA-GCAGAT-
GTTCGACCCCAAGAACATGATGGCTGCCTCCGACTTCCGCAACGGTCGCTACCTGACGTGCTCTGCCATCTT
GTAAGATGAT-AT----GTTCCATC-----------CGGTC-------------------------TATGATAAC-TAA---
TTTGCTAACTTG---AACTTTTCTAGCCGTGGCAAGATCTCCATGAAGGAGGTTGAGGACCAGATGCGCAAC--
----

>A_michelianum_CBS_119993

-----AAATTTTCCCTCA-----ATAAGATTCCCGT----------AATAAA-G--C------CG--------------------------------------
-ATTATCGCC-----TAAACCT--------TGAA--GAT--CTCCAGGAT----
----------------------TCC------CAACA----AC-----ATCCAGAGGG---TTAC------ACCAGCATCAAG-TTC-----CA----
--------C---AAC------G--A------------------------TGAGAGTTT-------------GTGAATCGAAGCTAA-CCGCA---TA---
TT-TCAT------------CTCAATAGGTTCACCTCCAGACCGGCCAATGCGTAAGTACTACGAT---CAAC--------------
--------GACGAGATATC-----------------GCGCTG----------------GAGT-ATAGTGG-----GGCTTACACGA---A-
TAT-----CG-TAGGGTAACCAAATTGGTGCTGCTTTCT--
------------GGCAAACCATCTCTGGCGAGCACGGTCTCGACAGCAATGGCGTGTAAGTGTTT---------------------
--GAGTTGCC-------------------AATTCGGAATGCC-------AAGAA---------------------------TAG--CAAC-------
TAAT-----------CAC-CAAT-
AAACAGCTACAACGGAACTTCCGAGCTCCAGCTCGAGCGCATGAGCGTTTACTTCAACGAGGTACGCAAC-
CAGGG-----------------G----------G----ACC-----TT-CAAC--------------------ACATGT-GG-A--------AGAGTAG-
TT-A-----CTAAT--CAC-CC-CAA---CATGC-
ACAGGCATCTGGTAACAAATACGTTCCTCGAGCCGTCCTCGTCGATCTCGAGCCCGGTACCATGGATGCCGT
CCGTGCTGGTCCTTTCGGCCAGCTCTTCCGACCTGACAACTTCGTCTTCGGTCAGTCCGGTGCCGGAAACAA
CTGGGCCAAGGGTCACTACACTGAGGGTGC-CGAGCTTGTCG-
ACAACGTTCTGGATGTCGTTCGTCGCGAGGCTGAGGGATGCGACTGCCTTCAGGGTTTCCAGATCACCCAC
TCTCTCGGTGGTGGTACCGGTGCCGGTATGGGTACTCTGTTGATCTCCAAGATCCGCGAAGAGTTCCCCGAC
CGCATGATGGCTACGTTCTCCGTCGTTCCCTCCCCTAAGGTTTCCGACACCGTTGTCGAGCCTTACAACGCCA
CTCTCTCCGTCCACCAGCTGGTCGAGAACTCCGATGAGACCTTCTGCATTGACAACGAGGCTCTCTACGACA
TCTGCATGCGTACGCTGAAGCTGTCTAACCCCTCGTACGGCGACCTGAACCATCTGGTCTCCGCCGTCATGT
CCGGTGTTACTACCTGCTTGCGTTTCCCTGGTCAGCTGAACTCTGATCTGCGCAAGCTCGCCGTGAACATGG
TTCCTTTCCCTCGTCTCCACTTCTTCATGGTCGGATTCGCTCCCCTGACCAGCCGTGGCGCTTACTCCTTCCGT
GCCGTCACCGTTCCCGAGTTGACTCA-GCAGAT-
GTTCGACCCCAAGAACATGATGGCTGCCTCCGACTTCCGCAACGGTCGCTACCTGACATGCTCTGCCATCTT-
----

>D_bambusicola_CBS_122872

-AACATGCGTGAGATTGTAAGTCATATCCAAAT-----------------------------------TCAAATGTTTACAC--------C--
TTTACCGAAGCCCAGC---------------ATCGCTAG-----AGAACCCCCCTGA-----ACGCG-TCTG--------------
AAAAA-------------CTC---GAAAACCCCCC-CTGA------GTT---CT-TCTGCCCCT-----CATGCGT-----------A-----
---CAA--CAACACCACAA------------------------TCT---------------AC-----
ATT--TTACA---CAGC------AACCATGTCAAAATTA-----TA------------C---GAC------A--A------------------------
CATGTT-------------GTAAAATATTCAAGCTAA-CCGCG---TT---TC-----------------
TTCAATAGGTTCACCTTCAGACTGGCCAATGTGTAAGTAACAACCA---TCAT-------CGA-AACGAAC----
TACGATATATATATACCTCTACACCTATTTCATA----------------AAAC-ATGGCGG-----GGCTCATACGA---A-
GAC-----GA-TAGGGTAACCAAATCGGTGCCGCTTTCT---
-------------GGCAAACCATCTCCAGCGAGCACGGTCTCGACAGCAATGGCGTGTATGTATTT---------------------
--GAATTGTC-------------------AATTTTCATCGTC-------AAGGA----------------------------TAT--CGAC-------TAAT-
----------CAC-CGAT-
AAATAGCTACAACGGTACTTCCGAGCTTCAGCTCGAGCGCATGAGCGTTTACTTCAACGAGGTACGAATT-
CACGC---AG------------A----------A----ATC-----AA-GGAT--------------------AGGTAA-AT-A--------GAATCGG-

CT-A-----CTAAT--CACCCT-CTA---CGCGT-
GCAGGCTTCCGGCAACAAGTATGTTCCTCGTGCCGTCCTCGTCGATCTCGAGCCCGGTACCATGGATGCTGT
CCGTGCTGGTCCCTTTGGTCAGCTCTTCCGACCCGACAACTTCGTTTTCGGTCAGTCCGGTGCTGGCAACAA
CTGGGCCAAGGGTCATTACACCGAGGGTGC-TGAGTTGGTTG-
ACAACGTTCTCGACGTCGTTCGCCGTGAGGCTGAAGGCTGCGACTGCCTCCAAGGTTTCCAGATCACCCACT
CCCTCGGTGGTGGTACTGGTGCCGGTATGGGTACCCTATTGATCTCCAAGATCCGCGAGGAGTTCCCCGAC
CGCATGATGGCTACTTTCTCCGTCATGCCCTCCCCTAAGGTCTCCGACACCGTCGTTGAGCCTTACAACGCCA
CCCTCTCTGTCCACCAGCTGGTCGAGAACTCCGACGAGACTTTCTGTATCGACAACGAGGCTCTGTACGACA
TCTGCATGCGTACTCTGAAGCTGTCCAACCCTTCCTACGGTGACCTGAACCACCTCGTCTCCGCCGTCATGTC
CGGTGTTACCACTTGCTTGCGTTTCCCCGGTCAGCTAAACTCTGACCTGCGCAAGCTCGCCGTGAACATGGT
TCCTTTCCCTCGTCTCCACTTCTTCATGGTCGGCTTCGCTCCCCTGACCAGCCGTGGCGCTCACTCCTTCCGTG
CCGTCACCGTTCCCGAGTTGACTCA-GCAGAT-
GTTCGACCCCAAGAACATGATGGCTGCTTCTGACTTCCGAAACGGTCGTTACCTGACGTGCTCTGCCATCTT
GTATGATATC-CT----CCCGTTTCTTTTT-----CTTTTTCTTTTT----------CTTTTTGTGGATGAATTCC-CAT---
TTTGCTAACTCG----
AATCGTGTAGCCGTGGCAAGGTCTCCATGAAGGAAGTTGAAGACCAGATGCGCAACGTTCAGAACAAGAA
CTCTTCGTACTTCGTCGAGTGGATTCCCAACAACATCCAGA

>D_childiae_CBS_122881

--ATGTATTC-----------------------
GAGTTGTC-------------------TACTCCTATTATTG------AAGAA--------------------------TAT--CAAC-------TAAT--
---------CGT-CAAT-
CAACAGTTACAACGGTACTTCCGAGCTCCAGCTCGAGCGCATGAGCGTCTACTTCAACGAGGTACGAATT-
TATAG-----------------A----------A----ATT-----AA-AGAT--------------------AAATAA-AT-G--------AAGATGA-TT-
G-----CTAAT--TGC-CT-CTA---CGCGT-
GCAGGCTTCCGGTAACAAGTATGTTCCTCGTGCCGTCCTCGTCGATCTCGAGCCCGGTACCATGGACGCCGT
CCGTGCTGGTCCCTTCGGTCAGCTCTTCCGACCCGACAACTTCGTCTTCGGTCAATCCGGTGCCGGAAACAA
CTGGGCCAAGGGTCATTACACTGAGGGTGC-CGAATTGGTTG-
ACCAAGTTCTCGATGTCGTTCGTCGTGAGGCTGAAGGCTGTGACTGCCTCCAGGGTTTCCAGATTACCCACT
CCCTCGGTGGTGGTACTGGTGCCGGTATGGGTACCCTATTGATCTCCAAGATCCGCGAGGAGTTTCCCGAC
CGCATGATGGCTACCTTCTCCGTTATGCCTTCCCCTAAGGTTTCCGATACCGTTGTCGAGCCTTATAACGCCA
CCCTCTCTGTCCACCAGCTGGTCGAGAACTCCGATGAGACCTTCTGTATCGACAACGAGGCTCTGTACGACA
TCTGCATGCGTACGCTGAAGCTGTCCAACCCCTCGTACGGTGACCTGAACCACCTGGTCTCTGCTGTCATGT
CCGGCGTTACTACTTGCTTGCGTTTCCCTGGTCAGCTAAACTCTGACCTGCGCAAGCTTGCCGTGAACATGG
TTCCTTTCCCTCGTCTCCACTTCTTCATGGTCGGCTTCGCCCCCCTGACCAGCCGTGGCGCTCACTCTTTCCGT
GCCGTCACCGTCCCTGAATTGACTCA-GCAGAT-
GTTCGACCCCAAGAACATGATGGCTGCTTCCGATTTCCGTAACGGTCGTTACCTGACGTGCTCAGCCATCTT
GTATGATTAT-CC----CCTTTAAA-----------ATTTT-------------------------ATACACTAC-TTG---

TTTGCTAACTTA----AATTCTCTAGCCGTGGCAAGGTCTCGATGAAGGAAGTCGAAGACCAGATG--------------
----

>D_petriniae_MUCL_49214

--AGCCCAGTT--------------
ATCAGTAG----AGAGAAACCCCTGA-----ACGCG-TCCG--------------AAAAAAAAA---------CCT---
CCAAACCCCCCC-CTTA------G------T-TCTGCCCCT-----CATACAC-----------A---------------------------------------
--------------CAA--CACTACCGCA-------------------------TCT---------------AC-----ATT--TCATA---TTGC------
AACTACATCAAA-TTG-----TA------------C---AGC------A--A------------------------CGTGTC--------------
GGAAAATTCAAAGCTAA-CCGCG---TT---TC-----------------
TTCAATAGGTTCATCTTCAGACCGGCCAATGTGTAAGTAACAGCGA---TGAT-------G--------------
GAAGAACCCAT----------------GGATATA----------------AAAT-ACAGCGG-----GGCTTACATGA---A-GAT-----
GA-TAGGGTAACCAAATTGGTGCTGCTTTCT--
--GGCAAACCATCTCCAGCGAGCACGGTCTCGACAGCAATGGCGTGTATGTATTC-----------------------
GAGTTATC-------------------TATTCCTATTACCGAGAATCAAGAA---------------------------TAT--CAAC-------
TAAT-----------CAT-CAAT-
CAACAGTTACAACGGTACTTCCGAGCTCCAGCTCGAGCGCATGAGCGTCTACTTCAACGAGGTAGGAATT-
TATAA-----------------G---------------AAT-----AG-GAAT--------------------AAATAA-AT-G--------GAGATAA-TT-
G-----CTAAT--TGT-CT-CCA---TGCGT-
GCAGGCTTCCGGCAACAAGTATGTTCCTCGTGCCGTCCTCGTCGATCTCGAGCCCGGTACCATGGACGCCGT
CCGTGCTGGTCCCTTCGGTCAGCTCTTCCGACCCGACAACTTCGTCTTCGGTCAATCCGGTGCCGGAAACAA
CTGGGCCAAGGGTCATTACACCGAGGGTGC-CGAGTTGGTTG-
ACCAGGTTCTCGATGTCGTCCGTCGTGAGGCTGAGGGTTGTGATTGCCTCCAGGGTTTCCAGATCACCCACT
CCCTCGGTGGTGGTACTGGTGCCGGTATGGGTACCCTGTTGATCTCCAAGATCCGCGAGGAGTTCCCCGAC
CGCATGATGGCCACCTTCTCCGTCATGCCCTCACCCAAGGTTTCCGACACCGTCGTCGAGCCTTACAACGCC
ACCCTCTCCGTCCACCAGCTGGTCGAGAACTCCGATGAGACCTTCTGTATCGACAACGAGGCTCTGTACGAC
ATCTGCATGCGTACGCTGAAGCTGTCCAACCCCTCGTACGGTGACCTGAACCACTTGGTCTCTGCTGTCATG
TCCGGCGTTACTACTTGCTTGCGTTTCCCTGGTCAGCTGAACTCTGACCTGCGCAAGCTTGCCGTGAACATG
GTTCCTTTCCCTCGTCTCCACTTCTTCATGGTCGGCTTCGCCCCCCTGACCAGCCGTGGCGCTCACTCTTTCCG
TGCCGTCACCGTCCCCGAGTTGACTCA-GCAGAT-
GTTCGACCCCAAGAACATGATGGCTGCTTCTGATTTCCGTAACGGTCGCTACCTGACGTGCTCAGCCATTTT
GTATGATGAT-AT----CCCTCGTAAA---------TATTT--------------------------ATACGTTAC-TTG---
TTTGCTAACTTG----
AATTCTCTAGCCGTGGCAAGGTCTCGATGAAGGAAGTCGAAGACCAGATGCGCAACGT-CAGAACAAG-----
----

>D_concentrica_CBS_113277

---GCCCAGT---------------
ATCACTA------GAGAAACCCCTGA-----ACGCG-TCCG--------------AAAAAAA-----------CTC---CAAA---
ACATC-CTTA------G------T-TCTACCCCT-----CATACAC-----------A---
-AAA--CGCTACCATA-------------------------TCT---------------AC-----ATT--TTATA---TTGC------
AACTACGTCAAA-TTG-----TA------------T---ACC------A--A------------------------CGTGTC--------------
GGGGAAATCAAAGCTAA-CCGCG---TT---TC-----------------
TTCAATAGGTTCATCTTCAGACTGGCCAATGTGTAAGTAACAGCGA---TCAT-------C--------------

GAAGAACCATG---------------GATATATA----------------AGAC-ACAGCGG-----GGCTCACATGA---A-GAT-----
GA-TAGGGTAACCAAATCGGTGCCGCTTTCT--
--GGCAAACCATCTCTAGCGAGCACGGTCTCGACAGCAATGGAGTGTATGTATTC-----------------------
GAATTGTT-------------------GATTCCCATCGAC-------GAGAA---------------------------TAT--CAAC-------TAAT--
---------CAT-CCAT-
CAACAGTTACAACGGTACTTCCGAGCTCCAGCTCGAGCGCATGAGCGTCTACTTCAACGAGGTATGAATT-
TGTAG-----------------G----------A----ACT-----AG-GGAT--------------------AAATAA-AC-G--------GAGACAA-
TT-G-----CTAAT--TGC-CT-CAA---CGCGT-
GCAGGCTTCTGGCAACAAGTATGTTCCTCGTGCCGTCCTCGTCGATCTCGAGCCCGGTACCATGGACGCCGT
CCGTGCTGGTCCCTTCGGTCAGCTCTTCCGACCCGACAACTTCGTTTTCGGTCAGTCCGGTGCCGGAAACAA
CTGGGCCAAGGGTCATTACACTGAGGGTGC-TGAGTTGGTTG-
ACCAAGTCCTCGATGTCGTTCGTCGTGAGGCTGAAGGCTGTGACTGCCTCCAGGGTTTCCAGATCACCCACT
CCCTCGGTGGTGGTACTGGTGCCGGTATGGGTACTCTGTTGATCTCCAAGATCCGCGAGGAGTTCCCCGAC
CGCATGATGGCCACCTTCTCCGTCATGCCCTCCCCTAAGGTTTCCGATACCGTTGTCGAGCCTTACAACGCCA
CCCTCTCCGTCCACCAGCTGGTCGAGAACTCCGATGAGACCTTCTGTATCGATAACGAGGCTCTGTACGACA
TCTGCATGCGCACGCTAAAGCTGTCCAACCCCTCGTACGGTGACCTGAACCACCTGGTCTCCGCCGTCATGT
CCGGCGTTACTACTTGCTTGCGTTTCCCTGGTCAGCTGAACTCTGACCTGCGCAAGCTTGCCGTGAACATGG
TTCCTTTCCCTCGTCTCCATTTCTTCATGGTTGGCTTCGCTCCCCTGACCAGCCGTGGCGCTCACTCTTTCCGT
GCCGTCACCGTCCCTGAGTTGACTCA-GCAGAT-
GTTCGACCCCAAGAACATGATGGCTGCTTCTGACTTCCGTAACGGTCGCTACCTGACGTGCTCAGCCATCTT
GTATGATATT-CC----CTTTAA-------------ATTTC--------------------------ATACATTGC-TTG---TTTGCTAACTTG-
---AATTCTCTAGCCGTGGCAAGGTCTCAATGAAGGAAGTTGAAGACCAGATGCGCAACGT-CAGAAC---------
----

>D_dennisii_CBS_114741

---GCCCAGT---------------
ATCACCA------GAGAAACCCCTGA-----ACGCG-TCCG--------------AAAAAAAAAAACT---CCCCC---CAAA---
ACACC-CTTA------G------T-TCTACCCCT-----CATACAC-----------A---
--AAA--CGCTACCATA-------------------------TCT---------------AC-----ATT--TTATA---TTGC------
AACTACGTCAAA-TTG-----TA------------T---ACC------A--A------------------------CGTGTC--------------
GGAAAAATCAAAGCTAA-CCGCG---TT---TC-----------------
TTCAATAGGTTCATCTTCAAACTGGCCAATGTGTAAGTAACAGCGA---TCAT-------C--------------
GAATAACTATG---------------GATATATA----------------AAAC-ACAGCGG-----GGCTCACATGG---A-GAT-----
GA-TAGGGTAACCAAATCGGTGCCGCTTTCT--
--GGCAAACCATCTCCAGCGAGCACGGTCTCGACAGCAATGGAGTGTATGTATTC-----------------------
GAATTGTT-------------------GATTCCTATGGAC-------AAAAA---------------------------TAT--CAAT-------TAAT--
---------CAT-CCAT-
CAACAGTTACAACGGTACTTCCGAGCTCCAGCTCGAGCGCATGAGCGTCTACTTCAACGAGGTATGAATT-
TGTAG-----------------G----------A----ACT-----AG-GGTT--------------------AAATAA-AT-G--------GAGACAA-
TG-G-----CTAAT--TGC-TT-CAA---CGCGT-
GCAGGCTTCTGGCAACAAGTATGTTCCCCGTGCCGTCCTCGTCGATCTCGAGCCCGGTACCATGGACGCCGT
CCGTGCTGGTCCCTTTGGTCAGCTCTTCCGGCCCGACAACTTCGTTTTCGGTCAGTCCGGTGCCGGAAACAA
CTGGGCCAAGGGTCATTACACTGAGGGTGC-TGAGTTGGTTG-
ACCAAGTCCTCGATGTCGTTCGTCGTGAGGCTGAAGGCTGTGACTGCCTCCAGGGTTTCCAGATCACCCACT

CCCTCGGTGGTGGTACTGGTGCCGGTATGGGTACTCTGTTGATCTCCAAGATCCGCGAGGAGTTCCCCGAT
CGCATGATGGCCACCTTCTCCGTCATGCCCTCCCCTAAGGTTTCCGATACCGTTGTCGAGCCTTACAACGCCA
CCCTCTCCGTCCACCAGCTGGTCGAGAACTCCGATGAGACCTTCTGTATCGATAACGAGGCTCTGTACGACA
TCTGCATGCGCACGCTAAAGCTGTCCAACCCCTCGTACGGTGACCTGAACCACCTGGTCTCCGCCGTCATGT
CCGGCGTTACTACTTGCTTGCGTTTCCCTGGTCAGCTGAACTCTGACCTGCGCAAGCTTGCCGTGAACATGG
TTCCTTTCCCTCGTCTCCATTTCTTCATGGTTGGCTTCGCTCCCCTGACCAGCCGTGGCGCTCACTCTTTCCGT
GCCGTCACCGTCCCTGAGTTGACTCA-GCAGAT-
GTTCGACCCCAAGAATATGATGGCTGCTTCTGACTTCCGTAACGGTCGCTACCTGACGTGCTCAGCCATCTT
GTAAGATACC-CC----CTTTAA------------ATTTT------------------------ATACGTTTA-TTG---
TTTGCTAATTCA----
AGTTCTCTAGCCGTGGAAAGGTTTCAATGAAGGAAGTTGAAGACCAGATGCGCAACGT-CAGAAC----------
----

>D_vernicosa_CBS_119316

------TT---TT----------------GTCAATAGGTTCATCTTCAGACTGGCCAATGTGTAAGTAAAAGCGA---TCAT----
---C-------------GAAGAACCATG----------------GATATA---------------AAAT-ACAGCGG-----
GGCTCACATGA---A-GAT-----GA-TAGGGTAACCAAATCGGTGCCGCTTTCT-----------------------------------
----
GGCAAACCATCTCCAGCGAGCACGGTCTCGACAGCAATGGCGTGTATGTATTC---------------------
GAGTTGCC------------------CATTCCTGTCACC-------AAGAA-------------------------TAT--CAAC-------TAAT--
---------CAT-CAAT-
CAACAGTTACAACGGTACTTCCGAGCTCCAGCTCGAGCGCATGAGCGTCTACTTCAACGAGGTACGAATT-
TATAG----------------G----------A-ACCACT-----AG-GGAT------------------AAATAA-AT-G--------GAGAGAA-
TT-G-----CTAAT--TGC-CT-CTA---CGCCT-
GCAGGCTTCCGGCAACAAGTATGTTCCTCGTGCCGTCCTCGTCGATCTCGAGCCCGGTACCATGGACGCCGT
CCGTGCTGGTCCCTTCGGTCAGCTCTTCCGACCCGACAACTTCGTCTTCGGTCAATCCGGTGCCGGAAACAA
CTGGGCCAAGGGTCATTACACTGAGGGTGC-CGAGCTGGTTG-
ACCAAGTTCTCGATGTCGTTCGTCGTGAGGCTGAAGGCTGTGACTGCCTTCAGGGTTTCCAGATCACTCACT
CCCTCGGTGGTGGTACTGGTGCCGGTATGGGTACCCTATTGATCTCCAAGATCCGCGAGGAGTTCCCCGAC
CGCATGATGGCCACCTTCTCCGTCATGCCCTCTCCTAAGGTTTCTGACACCGTTGTCGAGCCTTACAACGCCA
CCCTCTCCGTCCACCAGCTGGTCGAGAACTCCGACGAGACCTTCTGTATCGACAACGAGGCTCTGTACGACA
TCTGCATGCGCACGCTGAAGCTGTCCAACCCTTCGTACGGTGACCTGAACCACCTGGTCTCTGCTGTCATGT
CCGGCGTTACTACTTGCTTGCGTTTCCCTGGCCAGCTGAACTCTGACCTGCGCAAGCTTGCCGTAAACATGG
TTCCTTTCCCTCGTCTCCACTTCTTCATGGTCGGCTTCGCTCCCCTGACCAGCCGTGGTGCTCACTCTTTCCGT
GCCGTCACCGTCCCTGAGTTGACTCA-GCAGAT-GTTC---

>D_eschscholtzii_MUCL_45435

--GAAGCCCAGC--------------
-ATTGTTAGAG--AAAAACCCCCCTGA-----ACGCG-TCCG--------------AAAAAA------------CTC---
GAAAACCACCCC-CTGA------G------T-TCTGCCCCT-----CATACAT-----------A---------------------------------------
---------------CAA--CGCCACCACA-------------------------TCT---------------AC-----ATC-TTCATA---GCTC------
AACCACGTCAAA-TCG-----TA------------TA--AGC------A--G-------------------------CGTGTTGGA----------A--
ATACATATAGGCTAA-CCGCG---TT---TCT----------------
TTCAATAGGTTCACCTTCAGACTGGCCAATGTGTAAGTAACAGCAA---TCAC-------GAA-AAGGACC------
CGCGATAAT-----------------ATAGTA----------------GTAC-ATGGCGG-----GGCTCATACGA---A-GTT-----GA-
TAGGGTAACCAAATCGGTGCCGCTTTCT--
GGCAAACCATCTCCAGCGAGCACGGTCTCGACAGCAATGGCGTGTATGTATTT-----------------------
GAATTGTC-------------------AATTGCCATCGCC-------AAGGA---------------------------TAT--CGAC-------TGAC-
----------CATAAAAT-
TAATAGCTACAACGGTACTTCTGAGCTTCAGCTCGAGCGCATGAGCGTCTACTTCAACGAGGTACGAATT-
CACAGC----------------A----------A-ATGATA-----AG-GGAT--------------------AGGTGG-GT-G--------
GGATCAG-TT-A-----CTAAT--TGT-CT-CTA---CGCAT-
GCAGGCTTCCGGCAACAAGTATGTTCCTCGTGCCGTCCTCGTCGATCTCGAGCCCGGTACCATGGACGCCGT
CCGTGCTGGTCCCTTCGGTCAGCTCTTCCGACCCGACAACTTCGTTTTCGGTCAGTCCGGTGCTGGCAACAA
CTGGGCCAAGGGTCATTACACTGAGGGTGC-TGAGCTGGTCG-
ACAACGTTCTCGATGTCGTTCGTCGTGAGGCTGAAGGTTGTGACTGCCTTCAGGGTTTCCAGATCACCCACT
CTCTCGGTGGTGGTACCGGTGCTGGTATGGGTACTCTGTTGATCTCCAAGATCCGCGAGGAATTCCCCGACC
GTATGATGGCTACCTTCTCCGTCATGCCCTCCCCCAAGGTTTCCGACACCGTCGTTGAGCCTTACAACGCCAC
CCTCTCGGTCCACCAGCTGGTCGAGAACTCCGATGAGACTTTCTGTATCGACAACGAGGCTCTGTACGATAT
CTGCATGCGTACTCTGAAGCTGTCCAACCCCTCCTACGGTGACCTGAACCACTTGGTTTCCGCGGTCATGTCC
GGTGTTACCACTTGCTTGCGTTTCCCTGGTCAGCTGAACTCTGACCTGCGCAAGCTGGCCGTGAACATGGTT
CCTTTCCCTCGACTCCACTTCTTCATGGTCGGCTTCGCTCCCCTGACCAGCCGTGGCGCTCACTCTTTCCGTGC
CGTCACCGTTCCTGAGTTGACTCA-GCAGAT-
GTTCGACCCCAAGAACATGATGGCTGCTTCCGACTTCCGCAACGGTCGTTACCTGACGTGCTCTGCCATCTT
GTATGATACT-TC----CCCGAAT--------------TTT--------------------------TTTTGTTAC-TAT---GTTGCTGACTTG-
--AAAATGTGTAGCCGTGGCAAGGTCTCCATGAAGGAAGTTGAAGACCAGATGCGGAACG--------------------
----

>D_placentiformis_MUCL_47603

--CTTACCCCGAAGCCCAGC--------
-------ATCGCTAGAGAAAAAAAACCCCCTGA-----ACGCG-TCCG--------------AAAAA-------------CTC---
GAAAAACCACCC-CTGA------G------T-TCTGCCCCT-----CATACAT-----------A---------------------------------------
---------------CAA--CGCCACCACA-------------------------TCT---------------AC-----ATT-TTCACA---GTTC------
AACCACGTCAAA-TTG-----TA------------TA--AAC------A--G-------------------------CATGTTGGA----------
AATAAACATATAAGCTAA-CCGCG---TC---TCT----------------
TTCAATAGGTTCACCTTCAGACTGGCCAATGTGTAAGTAACAGCAA---TCAC-------GAA-AAGGACC------
CGCGATAAT-----------------ATAGTA----------------GTAC-ATGGCGG-----GGCTCATACGA---A-GAT-----GA-
TAGGGTAACCAAATCGGTGCCGCTTTCT--
GGCAAACCATCTCCAGCGAGCACGGTCTCGACAGCAATGGCGTGTATGTATTT-----------------------
GAATTGTC-------------------AATTGCCATCGTC-------ATGGA---------------------------TAT--CGAC-------TGAC--
---------CATGAAAT-

TAATAGCTACAACGGTACTTCTGAGCTTCAGCTCGAGCGCATGAGCGTCTACTTCAACGAGGTACGAATT-
CACAGCCCAG------------A----------A-AT-GAT-----AA-GAAT--------------------AGGTAA-AT-G--------
GAATCAG-GT-A-----CTAAT--TGT-CT-CTA---CGCAT-
GCAGGCTTCAGGCAACAAGTATGTTCCTCGTGCCGTCCTCGTCGATCTCGAGCCCGGTACCATGGACGCCGT
CCGTGCTGGTCCCTTCGGTCAGCTCTTCCGACCCGACAACTTCGTTTTCGGTCAGTCCGGTGCTGGCAACAA
CTGGGCCAAGGGTCATTACACCGAGGGTGC-TGAGTTGGTTG-
ACAACGTTCTCGATGTCGTTCGTCGTGAGGCTGAAGGCTGTGACTGCCTTCAGGGTTTCCAGATCACCCACT
CTCTCGGTGGTGGTACCGGTGCCGGTATGGGTACTCTGTTGATCTCCAAGATCCGCGAGGAGTTCCCCGAC
CGCATGATGGCTACCTTCTCCGTCATGCCCTCCCCCAAGGTTTCCGACACCGTCGTTGAGCCTTACAACGCCA
CCCTCTCGGTCCACCAACTGGTCGAGAACTCCGATGAGACCTTCTGTATCGACAACGAGGCTCTGTACGACA
TCTGCATGCGTACTCTGAAGCTGTCCAACCCCTCCTACGGTGACCTGAACCACTTGGTTTCCGCAGTCATGTC
CGGTGTTACCACTTGCTTGCGTTTCCCTGGTCAGCTGAACTCTGACCTGCGCAAGCTGGCCGTGAACATGGT
TCCTTTCCCGCGTCTCCACTTCTTCATGGTCGGCTTCGCTCCCCTGACCAGCCGTGGCGCTCACTCTTTCCGTG
CCGTCACCGTTCCTGAGTTGACTCA-GCAGAT-
GTTCGACCCCAAGAACATGATGGCTGCTTCCGACTTCCGCAACGGTCGTTACCTGACGTGCTCTGCCATCTT
GTATGATTCC-TA----CCCGAGA------------TTTTT-------------------------ATTCATTAC-TGT---
GTTGCTAACTTG----AAATGTGTAGCCGTGGCAAGGTCTCCATGAAGGAAGTTGAAGACCAGAT--------------
----

>Ruwenzoria_pseudoannulata_MUCL_51394

------------------------GCG-TCCG--------------AAAAA-------------CTC---GAAA---CCCCC-CTGA------A------T-
TCTGCCCCT-----CATGCCT-----------A--CAA--CGCCACCACA------
-------------------TCT---------------AC-----ATT--GTATA-------------------------CTG------------------------------------
-----------------------CATGTC--------------GGAAATATCAAAGCTAA-CCGCG---TT---CTT----------------
TTTAATAGGTTCATCTCCAGACTGGCCAATGTGTAAGTAATAGCGA---TGAC-------GGA-AGAAACC----
CACGGGATATA-------------------------------------AAAAC-ACAGCGG-----GGCTCATACGA---A-GTT-----GA-
TAGGGTAACCAAATCGGTGCCGCTTTCT--
GGCAAACCATCTCCAGCGAACACGGCCTCGACAGCAATGGCGTGTATGTATTT----------------------
CAATTGAC-------------------AATTTCAATTGTT-------AAGAA---------------------------TGT--CAAC-------TGAC--
---------CAC-CAAT-
CAATAGCTACAATGGTACTTCCGAGCTTCAGCTTGAGCGCATGAGCGTCTACTTCAACGAGGTACGAATT-
AATAG-----------------A----------G----CTT-----AC-GGAT-------------------AAATAA-AT-G--------
GAACCAACTT-A-----CTAAT--TGC-CT-CTA---CGTGT-
GTAGGCTACCGGCAACAAGTATGTTCCTCGTGCCGTCCTCGTCGATCTCGAGCCCGGTACCATGGACGCCGT
CCGTGCTGGTCCTTTCGGTCAGCTCTTCCGACCCGACAACTTCGTCTTTGGTCAGTCCGGTGCTGGAAACAA
CTGGGCCAAGGGTCATTACACTGAGGGTGC-TGAGTTGGTTG-
ACAACGTTCTCGATGTCGTTCGTCGTGAGGCTGAGGGCTGTGACTGTCTCCAGGGTTTCCAAATCACCCACT
CCCTCGGTGGTGGTACTGGTGCCGGTATGGGTACCTTGTTGATCTCCAAGATCCGCGAAGAGTTCCCCGAC
CGCATGATGGCTACCTTCTCCGTCATGCCTTCCCCCAAGGTCTCCGACACCGTCGTCGAGCCTTACAACGCTA
CCCTCTCCGTCCACCAGCTGGTCGAGAACTCCGACGAGACCTTCTGTATCGACAACGAGGCTCTGTACGACA
TCTGTATGCGTACTCTTAAGCTTTCCAACCCCTCCTACGGTGACCTGAACTACCTGGTCTCCGCCGTCATGTC
TGGCGTTACCACTTGCTTGCGTTTCCCCGGTCAACTGAACTCTGACCTGCGCAAGCTCGCCGTGAACATGGT
TCCTTTTCCTCGTCTGCACTTCTTCATGGTCGGCTTCGCTCCCCTGACCAGCCGTGGCGCCCACTCTTTCCGTG

CTGTCACCGTTCCCGAGTTGACTCA-GCAGAT-
GTTCGACCCCAAGAACATGATGGCTGCTTCTGACTTCCGTAACGGCCGTTACCTGACGTGCTCTGCCATCTT
GTATGTTATT-CT----TCTACTTTTT---------TTATT-------------------------ATAGATTAT-TAG---
TTTGCTAACTTC----AATCGTCTAGCCGTGGCAGGGTCTCCA---

>Thamnomyces_dendroidea_CBS_123578

--TGAA-------------T-TCTGCCCCT-----
CACGCAT---------ACAA----------------------CACGCATAC-------------------AACAA--CGCCGCCACA---------------
----------TCA---------------AC-----AACTTACACG---CTGC------AACCACGTCAAA-TTTAATTTTA------------T---
TAC------A--G------------------------CTTGTT------------GAAAAAATAGTCGAGCTAA-CCGCG---TT---TTC--------
--------TTCAATAGGTTCACCTTCAAACTGGCCAATGTGTAAGTAGAGGTAG---CAAC-----------GATGAGG----
GAAAGGACCGC-----------------GATATAT---------------AGCA-ATAGCGG-----GGCTCATACGA---A-GAT-----
GA-TAGGGTAACCAAATCGGTGCTGCTTTCT--
--GGCAAACCATCTCCAGCGAGCACGGCCTCGACAGCAATGGCGTGTATGTATTT-----------------------
GAATTGTT------------------AAATTCATATCGCC-------GAGGA---------------------------TAT--CAAC-------TGAC-
----------CAA-CGAT-
GGATAGCTACAACGGTACTTCCGAGCTTCAGCTCGAGCGCATGAGCGTCTACTTCAACGAGGTACGAATT-
TCCAG-----------------A----------T----ACC-----AA-GGATTACTACCTAG-----GTAAAATAAAT-AT-A--------
GAATCAG-TT-G-----TTAAT--CGC-TT-CTA---CGCGT-
GCAGGCTTCCGGCAACAAGTATGTTCCTCGTGCCGTCCTCGTCGATCTCGAGCCCGGTACTATGGACGCTGT
CCGTGCTGGTCCCTTTGGCCAGCTCTTCCGACCCGACAACTTCGTTTTTGGCCAGTCCGGTGCTGGAAACAA
CTGGGCCAAGGGTCACTATACCGAGGGTGC-TGAATTGGTTG-
ACAACGTTCTCGACGTCGTTCGTCGCGAGGCTGAGGGCTGTGACTGCCTCCAGGGTTTCCAGATCACCCACT
CCCTCGGTGGTGGTACTGGTGCCGGTATGGGTACCCTGCTGATCTCCAAGATCCGTGAGGAGTTCCCCGAC
CGTATGATGGCTACCTTCTCCGTCATGCCCTCCCCCAAGGTGTCCGACACCGTCGTTGAGCCTTACAACGCC
ACCCTCTCCGTCCACCAGCTGGTCGAGAACTCGGACGAGACCTTCTGTATCGACAACGAGGCTCTGTACGAC
ATCTGCATGCGTACCCTGAAGCTGTCTAACCCCTCGTACGGTGACCTGAACCACCTGGTCTCCGCCGTCATG
TCCGGTGTTACCACTTGCTTGCGTTTCCCCGGTCAGCTAAACTCGGACCTGCGCAAGCTTGCCGTGAACATG
GTTCCTTTCCCTCGTCTCCACTTCTTCATGGTCGGCTTCGCTCCCCTGACCAGCCGTGGCGCTCACTCTTTCCG
TGCCGTCACCGTTCCCGAGTTGACTCA-GCAGAT-
GTTCGACCCCAAGAACATGATGGCTGCTTCTGACTTCCGCAACGGTCGTTACCTGACGTGCTCTGCCATCTT
GTACGATATT-CC----CCCTTTCCCCGAG-----ATTCTC-------------------------ATGCGCTAT-TGT---
TTTGCTAACTTG----
AACCGCGTAGCCGTGGCAAGGTCTCCATGAAGGAAGTTGAAGACCAGATGCGCAACGT-CAGAACAAGA---
----

>Rhopalostroma_angolense_CBS_126414

---CCC----------CCCCC-CTGA------G------T-TCTGCCCCT-
----CATGCAC---------GCAA--CGCCATCACA---------------------
---TCG---------------AC-----ATT--TTACG---CTGC------AACCGAATCAGA-TGTCGG--CAAA-------TGTT---
GAC------A--AA----------------------TATTGCGAAC----------GTGAATTTTTAAGCTAA-CCGCG---TT---TTC-----

-----------TCCAATAGGTTCACCTTCAGACCGGCCAATGTGTAAGTAGCAGCGA---CGAC----------------------
GGGAAGGACC-----------------GCGATGTATA----------TAGTGA-ATGGCGG-----GGCTCATACCA---A-GGT----
-GA-TAGGGTAACCAAATCGGTGCTGCTTTCT--
---GGCAAACCATCTCCAGCGAGCACGGCCTCGACAGCAATGGCGTGTACGTATCC----------------------
GAGCGGCC-------------------AGTCCTTATCGTC-------GAGGA--------------------------CGT--CAAC-------
TGACG----------GAC-AAAT-
CAATAGCTACAACGGTACTTCCGAGCTTCAGCTCGAGCGCATGAGCGTCTACTTCAACGAGGTACGGACT-
TACTTGCAG-------------A----------T----ACC-----AA-AAAT--------------------AGGCAA-AT-A--------GAACTAG-
CT-ACTAATCTAAT--CGC-CT-CGA---CGCGT-
ACAGGCTTCCGGCAACAAGTATGTTCCCCGTGCCGTCCTCGTCGATCTCGAGCCCGGTACCATGGACGCCGT
CCGTGCTGGTCCCTTCGGCCAGCTCTTCCGACCCGACAACTTCGTTTTTGGTCAGTCCGGTGCTGGCAACAA
CTGGGCCAAGGGTCACTACACCGAGGGTGC-CGAGCTGGTTG-
ACAACGTTCTTGACGTCGTTCGTCGCGAGGCTGAAGGCTGCGACTGCCTCCAGGGTTTCCAGATCACCCACT
CCCTCGGTGGTGGTACCGGTGCCGGTATGGGTACCCTGCTCATCTCCAAGATCCGCGAGGAGTTCCCCGAC
CGTATGATGGCCACCTTCTCCGTCATGCCCTCCCCCAAGGTCTCCGACACGGTCGTTGAGCCTTACAATGCA
ACCCTCTCCGTCCACCAGCTGGTCGAGAACTCGGATGAGACCTTCTGTATCGACAACGAGGCGCTGTACGA
CATCTGCATGCGTACCCTGAAGCTGTCTAACCCCTCCTACGGAGACCTGAACCACCTGGTCTCCGCCGTCAT
GTCCGGTGTTACCACTTGCTTGCGTTTCCCTGGTCAGCTAAACTCTGACCTGCGCAAGCTCGCCGTGAACAT
GGTTCCTTTCCCTCGTCTCCACTTCTTCATGGTCGGCTTCGCTCCCCTGACCAGCCGCGGCGCTTACACTTTCC
GTGCCGTCACCGTTCCCGAATTGACGCA-ACAGAT-
GTTCGACCCCAAGAACATGATGGCTGCTTCCGACTTCCGTAACGGTCGTTACCTGACGTGCTCTGCCATCTT
GTATGATATC-CT----CCCCCCT------------TCCCCCCCTTT-TTTTTAAAATTTTTTTGTATGCATGAC-TAG---
CTTGCTAACCTG----GACTGTGTAGCCGTGGCAAGGT--

>P_hunteri_MUCL_52673

-------GAAACCCCTGA-----ACGCG-TCGA-------------GAAAGT---------GA-------------------------------------
ATGCCCCTC-----CACGCAT--------CACA--GAG--CTGAACCACAT-
---------------------CATCC------TATGG----TT-----CTC---TGGA---ACAC------ACTACCACGAAAAATAAA---CA--
----------C---GAC------T--A-----------------------TTTGAC---------------GAATATTTGGAGCTAA-CCATA---TC---
TCTT-GT------------CTTTATAGGTTCACCTTCAGACCGGCCAATGCGTAAGTACCATAAT---CGCC---------------
-------AACGAGACATA-----------------GCACTG----------------GGAC-GTAGCGG-----GGCTCATACGA---A-
GAT-----CG-TAGGGTAACCAAATCGGTGCTGCTTTCT---
------------GGCAAACCATCTCGAGCGAGCACGGCCTCGACAGCAATGGCGTGTACGTATTTC-------------------
---GAGTCGTC-------------------AACACCTACCGCG-------AAGAG--------------------------GTC--CAAC-------
TAAT-----------CAC-CAAT-
AAACAGCTACAACGGCACCTCCGAGCTCCAGCTCGAGCGCATGAGCGTCTACTTCAACGAGGTACGGATT-
CGCGA-----------------A----------A----GCA-----G-------------------------AAATGG-AT-G--------GAGCTAG-
TTAA-----CTGAT--CGT-AT-CAC---CGCAT-
GCAGGCCTCCGGCAACAAGTATGTTCCTCGCGCTGTCCTCGTCGATCTCGAGCCCGGCACCATGGACGCCG
TCCGTGCCGGTCCCTTCGGTCAGCTCTTCCGACCGGACAACTTCGTCTTCGGTCAGTCCGGTGCTGGCAACA
ACTGGGCCAAGGGTCACTACACTGAGGGTGC-TGAGCTGGTTG-
ACAACGTCCTCGACGTCGTCCGCCGTGAGGCCGAAGGCTGCGACTGCCTCCAGGGCTTCCAGATCACCCAC

TCCCTCGGTGGTGGTACCGGTGCCGGTATGGGTACCCTGTTGATCTCCAAGATCCGCGAGGAGTTCCCCGA
CCGCATGATGGCCACCTTCTCCGTCATGCCTTCGCCCAAGGTCTCCGACACGGTCGTCGAGCCCTACAACGC
CACTCTCTCCGTCCACCAGCTGGTCGAGAACTCTGACGAGACCTTCTGTATCGACAACGAGGCTCTGTACGA
TATCTGCATGCGTACGCTGAAGTTGTCCAACCCCTCGTACGGCGATCTGAACCACCTGGTCTCCGCCGTCAT
GTCTGGCGTCACCACTTGTCTGCGTTTCCCCGGCCAGCTGAACTCTGACCTGCGCAAGCTCGCCGTGAACAT
GGTTCCTTTCCCCCGTCTCCACTTCTTCATGGTCGGCTTCGCCCCTCTGACCAGCCGCGGCGCTCACTCCTTCC
GCGCCGTCACCGTTCCCGAGTTGACTCA-GCAGAT-
GTTCGACCCCAAGAACATGATGGCTGCCTCCGACTTCCGCAACGGTCGCTACCTGACGTGCTCTGCCATCTT
GTAAGATATC-CC----CCTAAAT------------CACTT-------------------------TGTTAGTAT-CGG---
CCTGCTGACCTA----AATCCCGTAGCCGTGGCAAGGTCTCCA---

>P_nicaraguense_CBS_117739

GGGACTATCCTTAGAGAAACCCCTGA-----ACGCG-TCGA--------------GAAAAGT---------GA--------------------
------------------ATGCCCCTC-----CACGCAT--------CACA---GAA--
CTGAACCACAT----------------------CATCC------TATGG----TT-----CTC---TGGA---ACAC------
ACTACCACGAAAAATAAA---CA------------C---GAC------T--A-----------------------TTTGAC---------------
GAATATTTGGAGCTAA-CCATC---TC---TCTT-GT-----------
CTTTATAGGTTCACCTTCAGACCGGCCAATGCGTAAGTACCATAAT---CGCC----------------------
AACGAGACATA-----------------GCACTG----------------GGAC-GTAGCGG-----GGCTCATACGA---A-GAT-----
CG-TAGGGTAACCAAATCGGTGCTGCTTTCT---
--GGCAAACCATCTCGAGCGAGCACGGCCTCGACAGCAATGGCGTGTACGTATTTC----------------------
GAGTCGTC-------------------AACCTCTACCGCG-------AAGAA--------------------------GTC--CAAC-------TAAT-
----------CAC-CAAT-
AAACAGCTACAACGGCACCTCCGAGCTCCAGCTCGAGCGCATGAGCGTCTACTTCAACGAGGTACGGATT-
CGCGA-----------------A----------A----GCA-----G-------------------------AAATAG-AT-G--------GAGCTAG-
TTAA-----CTGAC--CGT-AT-CGC---CGCAT-
GCAGGCCTCTGGCAACAAGTATGTTCCTCGCGCTGTCCTCGTCGATCTCGAGCCCGGCACCATGGACGCCGT
CCGTGCCGGTCCCTTCGGCCAGCTCTTCCGACCGGACAACTTCGTCTTCGGTCAGTCCGGTGCTGGCAACAA
CTGGGCCAAGGGTCACTACACTGAGGGTGC-TGAGCTGGTTG-
ACAACGTCCTCGACGTCGTCCGCCGTGAGGCCGAAGGCTGCGACTGCCTCCAGGGCTTCCAGATCACCCAC
TCCCTCGGTGGTGGTACCGGTGCCGGTATGGGTACCCTGTTGATCTCCAAGATCCGCGAGGAGTTCCCCGA
CCGCATGATGGCCACCTTCTCCGTCATGCCTTCGCCCAAGGTCTCCGACACGGTCGTCGAGCCCTACAACGC
CACTCTCTCCGTCCACCAGCTGGTCGAGAACTCTGACGAGACCTTCTGTATCGACAACGAGGCTCTGTACGA
TATCTGCATGCGTACGCTGAAGTTGTCCAACCCCTCGTACGGCGATCTGAACCACCTGGTCTCCGCCGTCAT
GTCCGGCGTCACCACTTGTCTGCGTTTCCCCGGCCAGCTGAACTCTGACCTGCGCAAGCTCGCCGTGAACAT
GGTTCCTTTCCCCCGTCTCCACTTCTTCATGGTCGGCTTCGCCCCTCTGACCAGCCGCGGCGCTCACTCCTTCC
GCGCCGTCACCGTTCCCGAGTTGACTCA-GCAGAT-
GTTCGACCCCAAGAACATGATGGCTGCCTCTGACTTCCGCAACGGTCGCTACCTGACGTGCTCTGCCATCTT
GTAAGATAGC-CC----CCTAAAT------------CACTT-------------------------TGTTAGTAT-CGG---
CCTGCTGACCTA----AATCCCGTAGCCGTGGCAAGGTCTCCATGAAGGAAGTGGAAGACCAGATGCG--------
----

>P_laminosus_MUCL_53305

--ATAGTGAA-------------CTC---CAAA---ACCCC-TGAT------------A-
TCTGCCCCTC----CACGCAT--------CACA--GAA--CTCAACCACA---
----------------------TCC------CGTGG----TT-----CTC---CAGA---ACAC------ACTACCACGAAAAATGAA---CA----
--------C---GAC------T--A-----------------------TTTGAC---------------GAATATTTGGAGCTAA-CCATA---TC---
TCTT-CT------------CTTTATAGGTTCACCTTCAGACCGGCCAATGCGTAAGTACCATAAT---CGCC---------------
-------GACGAGACATA-----------------GCACTG----------------GAAT-ATAGCGG-----GGCTCATGCGA---A-
GAT-----CG-TAGGGTAACCAAATCGGTGCTGCTTTCT--
------------GGCAAACCATCTCGAGCGAGCACGGTCTCGACAGCAATGGCGTGTATGTATTTC-------------------
--GAGTCGTC-------------------AACATCGACCGCG-------AAGAA--------------------------CTC--GAAC-------
TAAT-----------CGC-CGAT-
AAACAGCTACAATGGCACCTCCGAGCTCCAGCTCGAGCGCATGAGCGTCTACTTCAACGAGGTACGGATT-
CGCGA-----------------A----------A----GCAAAAATAG-ATGG--------------------AAATAG-AT-G--------
GAACTAG-TTAA-----CTGAT--CGT-AT-CAC---CGCAT-
GCAGGCCTCCGGTAACAAGTATGTTCCTCGCGCCGTCCTCGTCGATCTCGAGCCCGGCACCATGGACGCCG
TCCGCGCTGGTCCCTTCGGTCAGCTCTTCCGACCGGATAACTTCGTTTTCGGTCAGTCCGGTGCCGGCAACA
ACTGGGCCAAGGGTCACTACACCGAGGGTGC-CGAGCTGGTTG-
ACAACGTCCTCGACGTCGTTCGCCGTGAGGCCGAAGGCTGCGACTGCCTCCAGGGCTTCCAGATCACCCAC
TCCCTCGGTGGTGGTACCGGTGCTGGTATGGGTACCCTGTTGATCTCCAAGATCCGCGAGGAGTTCCCCGA
CCGCATGATGGCTACCTTCTCCGTCATGCCTTCCCCCAAGGTCTCCGACACCGTCGTCGAGCCTTACAACGCC
ACTCTCTCCGTCCACCAGCTGGTCGAGAACTCTGACGAGACGTTCTGTATCGACAACGAGGCTCTGTACGAT
ATCTGCATGCGTACGCTGAAGCTGTCCAACCCCTCGTACGGCGATCTGAACCACCTGGTCTCCGCCGTCATG
TCCGGCGTCACCACTTGTCTGCGTTTCCCCGGCCAGCTGAACTCTGATCTGCGCAAGCTCGCCGTGAACATG
GTTCCCTTCCCCCGTCTCCACTTCTTCATGGTCGGCTTCGCCCCTCTGACCAGCCGCGGCGCTCACTCCTTCCG
CGCCGTCACCGTTCCCGAGTTGACTCA-GCAGAT-
GTTCGACCCCAAGAACATGATGGCTGCCTCCGACTTCCGCAACGGTCGCTACCTGACGTGCTCGGCCATCTT
GTAAGATACC-CC----CCCAGAA------------TTTTT-------------------------TATTGGTAT-CAG---
CCTGCTGACCTA----AA--

>H_hinnuleum_MUCL_3621

CCCGAACCGGGTACCCAATTGCCCCTGA-----ACGCG-TCCA-------------AAAAC------------CCT---CCAA---
ACCCC-TGAT------T------T-TCTGCCCCT-----CACATGC---------ACA---
---CAA--CATTACAGCA-------------------------TCA------TACAG----CT-----CTT---CTCG---CTAT------
CATGGCATCAAA-CTG-----GAG---------------AATAAATGAA--A-----------------------TGAGATGA-----------
GAATAGAATTACAGCTAA-CAG---------ATCATTTT------------
CTCGATAGGTTCACCTCCAGACCGGCCAATGCGTAAGTACAACAAC--------------CAC-GACCGCG----
GACGAAGCAAC-----------------GCGCTGGG-------------AATC-TTAGAGG-----GGCTCACAC-----A-ACC-----
TG-CAGGGTAACCAAATCGGTGCTGCTTTCT--
--GGCAAACCATCTCTGGCGAGCACGGCCTCGACAGCAATGGCGTGTAAGTAGAG----------------------
TAGCTGCC-------------------AATTTCTGAGGCC-------AAGAG--------------------------TGA--GAAC-------TGAC-
----------CAC---AA-
CAATAGCTACAACGGAACCTCTGAGCTCCAGCTCGAACGCATGAGCGTCTACTTCAACGAGGTATGCAAGG

AATCA-----------------A----------A----ACT-----GG-GGAT-------------------GACGGT-GC-C--------AGATCAG-GT-A-----CTAAT--CGC-CC-TAC---TCTAC-GCAGGCTTCCGGTAACAAGTACGTCCCTCGTGCCGTCCTCGTCGATCTCGAGCCCGGTACCATGGACGCCGTCCGTGCTGGCCCCTTCGGCCAGCTCTTCCGACCTGACAACTTCGTTTTCGGCCAGTCTGGTGCCGGAAACAACTGGGCCAAGGGTCATTACACTGAGGGTGC-TGAGCTTGTTG-ACCAGGTCCTTGATGTCGTTCGCCGTGAGGCTGAGGGCTGCGATTGCCTTCAGGGTTTCCAGATCACCCACTCTCTCGGCGGTGGTACTGGTGCCGGTATGGGTACTCTCCTGATCTCCAAGATCCGTGAGGAGTTCCCTGACCGCATGATGGCCACCTTCTCCGTCGTTCCTTCGCCTAAGGTCTCCGACACTGTCGTTGAGCCCTACAACGCCACCCTCTCCGTCCACCAGCTGGTCGAGAACTCCGACGAGACCTTCTGTATCGACAACGAGGCCCTGTACGACATCTGCATGCGCACCCTGAAGCTATCCAACCCCTCGTACGGTGACCTGAACCACCTGGTCTCCGCCGTCATGTCCGGTGTCACCACCTGCTTGCGCTTCCCCGGCCAGCTGAACTCCGACCTACGCAAGCTCGCCGTGAACATGGTTCCTTTCCCTCGTCTCCACTTCTTCATGGTCGGCTTCGCTCCCCTGACCAGCCGTGGCGCGCACTCCTTCCGCGCCGTCACTGTTCCCGAGTTGACTCA-GCAGAT-GTTCGACCCCAAGAACATGATGGCTGCCTCCGACTTCCGCAACGGTCGTTATCTGACCTGCTCCGCCATCTTGTAAGATGCT-CC----TCGTAAA------------TTGCG-------------------------TGATATTAC-TTG---TTGGCTAACATG----TGCTCTCTAGCCGTGGTAAGGTCTCCATGAAGGAGGTCGAGGATCAGATGCGCA---

>H_investiens_CBS_118183

---ACCCTGTCCCCAATTGCCCCTGA-----ACGCG-TCCA-------------AAAAAA------------TCT---CCAA---ACCCC-TGAT------T------T-CCTGCCCCT-----CATGCAC---------ACA--AAA--CAGTACAGCT-------------------------TCA------TGCAG----CT-----CTT---TTCG---CAAC------GATTCAATCAAA-CCG-----AA------------C---AAC------G--A-----------------------TGAGAT----------------GAAAATCGCAGCTAA-CAA---------ATGTTTTT------------CTCAATAGGTTCACCTCCAGACCGGCCAATGCGTAAGTACAACGAT--------------CAC-AACCGCG----ACCGAGGTAAC-----------------GCGCTGG---------------AAAT-ATAGAGG-----GGCTTACACAA-----ACT-----AA-CAGGGTAACCAAATTGGTGCTGCTTTCT---GGCAAACCATCTCTGGCGAGCACGGTCTCGACAGCAATGGCGTGTAAGTATAT-----------------------GAGTTGTC-------------------AATTCCCGATGCC-------AAGAA---------------------------TAG--CACC-------TAAT-----------GAC-CAAT-AATTAGCTACAACGGAACCTCCGAGCTCCAGCTCGAGCGCATGAGCGTCTACTTCAACGAGGTATGCAAG-AATCG-----------------A----------A----GCC-----AG-GGAT-------------------AGAGAT-TCAA--------AGGTCGG-CT-A-----CTAAT--CAA-CC-TAA---CCTAC-ACAGGCTTCCGGCAACAAGTATGTCCCTCGCGCCGTCCTCGTCGATCTCGAGCCCGGTACCATGGACGCTGTCCGTGCTGGTCCCTTCGGCCAGCTTTTCCGACCTGACAACTTCGTCTTCGGCCAGTCTGGTGCCGGAAACAACTGGGCCAAGGGTCACTACACTGAGGGTGC-TGAGCTTGTTG-ACAACGTCCTTGATGTCGTTCGTCGTGAGGCTGAGGGCTGCGACTGCCTCCAGGGTTTCCAGATCACCCACTCCCTCGGTGGTGGTACTGGTGCCGGTATGGGTACTCTCTTGATCTCCAAGATCCGTGAGGAGTTCCCCGACCGCATGATGGCCACCTTCTCCGTTGTGCCTTCCCCTAAGGTTTCCGACACTGTCGTCGAACCCTACAACGCCACCCTCTCGGTCCACCAGCTGGTCGAGAACTCTGACGAGACCTTCTGCATTGACAACGAGGCTCTGTACGACATCTGCATGCGTACCCTGAAGCTATCCAACCCCTCGTACGGTGACCTGAACCACCTGGTCTCTGCTGTCATGTCCGGTGTCACCACTTGCTTGCGTTTCCCCGGTCAGCTGAACTCTGATCTGCGCAAGCTCGCCGTGAACATGGTTCCTTTCCCTCGTCTCCACTTCTTCATGGTCGGCTTCGCTCCTCTGACCAGCCGTGGCGCTCACTCCTTCCGTGC

CGTTACCGTTCCCGAGTTGACTCA-GCAGAT-
GTTCGATCCCAAGAACATGATGGCTGCTTCTGACTTCCGTAACGGTCGTTACCTCACTTGCTCTGCCATCTTG
TAAGATACC-CA----GCCTAAA------------CCCTCC------------------------ATAAATCTC-TGA---
TTCACTAACTCT----
ATTTATCTAGCCGTGGCAAGGTTTCCATGAAGGAGGTTGAGGACCAAATGCGCAACGTCCAGAA-------------
----

>H_lateripigmentum_MUCL_53304

--ACGCAC--
-------ACG---TAA--AACCACCACAG-----------------------CTCC------
TGCGG----CT-----GTT---TTCG---ATAC------GATGTCAACAAA-CCA-----AAG-----------CA--GAC--GCNCA--
A-----------------------TGACAT----------------AGGAATCAAAGCTAA-CAG---------ATGTTTGT------------
CTCAATAGGTTCACCTCCAGACCGGCCAATGCGTAAGTACAATGAT--------------CAC-GACCGCA----
GACGAAGAAAC-----------------GCGCTGG---------------AATT-ATAGAGG-----GGCTCACAC-----A-AGC-----
TG-CAGGGTAACCAAATCGGTGCTGCTTTCT---
--GGCAAACCATCTCTGGCGAGCACGGTCTCGACAGCAATGGCGTGTAAGTACCT-----------------------
GAGTTGTC-------------------AATTCCTGATGCC-------AAGAA---------------------------CAG--GAGC-------TAAC-
----------CAC-CAAT-
ATTTAGCTACAACGGAACCTCTGAGCTGCAGCTCGAGCGCATGAGCGTCTACTTCAATGAGGTATGCATGT
AATCG-----------------A----------A---ATC-----AA-GGAT--------------------GGCCAT-GC-T--------AGGACAG-
TC-A-----CTAAT--CAC-CC-TAA---CCTAC-
ACAGGCTTCCGGCAACAAGTATGTCCCTCGCGCCGTCCTCGTCGATCTCGAGCCCGGTACCATGGATGCCGT
CCGTGCTGGCCCCTTCGGCCAGCTTTTCCGACCTGACAACTTCGTCTTCGGTCAATCCGGTGCCGGAAACAA
CTGGGCCAAGGGTCACTACACTGAGGGTGC-TGAGCTGGTTG-
ACAACGTCCTTGACGTCGTTCGTCGTGAGGCTGAGGGTTGCGACTGCCTCCAGGGTTTCCAGATCACCCACT
CCCTCGGTGGTGGTACCGGTGCCGGTATGGGTACCCTCTTGATCTCCAAGATCCGCGAGGAGTTCCCCGAT
CGCATGATGGCCACCTTCTCCGTCGTTCCTTCCCCTAAGGTCTCCGACACCGTCGTCGAGCCCTACAACGCCA
CCCTGTCTGTCCACCAGCTGGTCGAGAACTCGGACGAGACCTTCTGCATTGACAACGAGGCTCTGTACGAC
ATCTGCATGCGTACCCTGAAGCTATCCAACCCCTCGTATGGTGACCTGAACCACCTGGTCTCTGCCGTCATGT
CCGGTGTTACCACCTGTTTGCGATTCCCCGGCCAGCTGAACTCCGACCTACGCAAGCTCGCCGTGAACATGG
TTCCTTTCCCTCGTCTTCACTTCTTCATGGTCGGCTTCGCTCCTCTGACCAGCCGTGGCGCTCACTCCTTCCGT
GCCGTCACCGTTCCCGAGTTGACTCA-GCAGAT-GTTCGACCCCAAGAACATGATGGCTGCCTCTGACTTCC--
----

>H_pulicicidum_CBS_122622

------------CCCTGA-----ACGCG-TCCC--------------AAAAAAT-----------ACT---CCAA---ACCCC-TGAT------T-----
-T-TCTGCCCCT-----CACGCAC--ACACAAAACA---AAC--
CACTGCAGCA-------------------------TCA------TGCGG----TG-----GAC---AGGA---CTGC------
GATGTCATCAAA-CTG-----GA------------C---AAC------G--A-----------------------CGAGGA----------------
AAGAATCACAGCTAA-CAG---------ATGTTTGT------------
TTCAATAGGTTCACCTCCAGACCGGCCAATGCGTAAGTAAACAACCAA-CGGT-------CACGAACCACG----

GGCGAAGCTAC-----------------GCGCTGG---------------AAAC-ATAGAGG-----GGCTTACAC-----A-AAC-----
TG-CAGGGTAACCAAATCGGTGCTGCTTTCT--
--GGCAAACCATCTCTGGCGAGCACGGCCTCGACAGCAATGGCGTGTAAGTACAT-----------------------
GAGCTGTC-------------------AATTCCTGATGCC-------AAGAG----------------------------TAG--GAAC-------TAAC-
----------CAC-CGAT-
AATTAGCTACAACGGAACCTCTGAGCTCCAGCTCGAGCGCATGAGCGTTTACTTCAACGAGGTATGCAGG-
AGTCGGTTGGG------GGTTGA----------A----GCC-----TC-GAAG--------------------AAAGCC-TC-A--------
AGGACAG-CT-A-----CTAAC--TAC-TC-TAA---CCTCT-
GCAGGCTTCCGGCAACAAGTACGTGCCTCGCGCCGTCCTCGTCGATCTCGAGCCCGGTACCATGGATGCCG
TCCGTGCTGGTCCCTTCGGCCAGCTCTTCCGACCTGACAACTTCGTCTTCGGCCAGTCTGGTGCCGGAAACA
ACTGGGCCAAGGGTCACTACACTGAGGGTGC-TGAGCTTGTTG-
ACAACGTCCTTGATGTTGTCCGTCGTGAGGCTGAGGGCTGCGACTGCCTCCAGGGTTTCCAGATCACCCACT
CCCTCGGTGGTGGTACCGGTGCCGGTATGGGTACTCTCCTGATCTCCAAGATCCGTGAGGAGTTCCCCGAC
CGCATGATGGCCACCTTCTCCGTCGTGCCTTCTCCTAAGGTCTCTGACACCGTCGTCGAGCCCTACAACGCCA
CCCTCTCGGTCCACCAGCTGGTCGAGAACTCCGACGAGACCTTCTGCATTGACAACGAGGCTCTGTACGACA
TCTGCATGCGTACCCTGAAGCTATCCAACCCCTCGTACGGTGACCTGAACCACCTGGTCTCCGCTGTCATGTC
CGGTGTCACCACCTGCTTGCGCTTCCCTGGCCAGCTGAACTCTGACCTCCGCAAGCTTGCCGTGAACATGGT
TCCTTTCCCTCGTCTCCACTTCTTCATGGTCGGCTTCGCCCCTCTGACCAGCCGTGGCGCTCACTCCTTCCGCG
CTGTCACCGTTCCCGAGTTGACTCA-GCAGAT-
GTTCGACCCCAAGAACATGATGGCTGCTTCCGACTTCCGTAACGGCCGCTACCTGACTTGCTCTGCCATCTT
GTAAGATATC-CT----TCGCGAA------------TTGTC--------------------------CGCTATTGC-TAGT--
TTTGCTAACTTCT--
GTGTTTTCCAGCCGTGGCAAGGTCTCCATGAAGGAGGTTGAGGACCAGATGCGCAACGTCCAGAACAAGA
A--

>H_olivaceopigmentum_DSM_107924

--------------CTGA-----ACGCG-TCCT-----------TCAAAAAAA------------CTC---CAAA---ACCCC-TGAT------T-----
-T-TCTGCCCCT-----CACGCAA------CACACA--CAA--
CGGCACAGCA-------------------------TCC------CTCGA----TT-----CTC---CCTG---ACAG------CCCATCATCAAC-
TCA-----TG------------C---AGG------A--A------------------------TGAGAT----------------GGTAAATCAAGCTAA-
CCGCG---TT---ATTACTT------------GCCTATAGGTTCACCTTCAGACCGGCCAGTGCGTAAGTACTACGAT-----
---------GAC-AACCACC----GACGAAACGTC-----------------GCGATA----------------GAAC-TTGGAGG-----
GGCTCACATGA---A-TAT-----CA-TAGGGTAACCAAATTGGTGCTGCTTTCT--

GGCAGACCATCTCTGGCGAGCACGGTCTCGACAGCAATGGCGTGTATGTATAT------------------------
GAGTTGTC-------------------AATTTCTGATGCC-------AAGAA----------------------------TCC--CAAC-------TGAC--
---------CAC-TGAT-
ACATAGCTACAACGGAACCTCTGAGCTCCAGCTCGAGCGCATGAGCGTCTACTTCAACGAGGTACGCAGC-
AATCG-----------------A----------A----GCC-----AG-GAAG--------------------AGTCAT-CT-A--------GGAGTAG-
CC-A-----CTAAT------CA-TAA---TATGC-
ATAGGCTTCCGGCAACAAGTATGTTCCTCGTGCCGTTCTCGTCGATCTCGAGCCCGGTACCATGGATGCCGT
CCGCGCTGGTCCTTTCGGCCAGCTTTTCCGACCTGACAACTTCGTCTTCGGCCAGTCGGGTGCCGGCAACAA
CTGGGCCAAGGGTCACTACACTGAGGGTGC-TGAGCTTGTTG-

ACCAGGTCCTTGACGTTGTTCGTCGTGAGGCTGAGGGCTGCGACTGCCTCCAGGGTTTCCAGATCACCCACT
CTCTCGGTGGTGGTACCGGTGCTGGTATGGGTACTCTTCTGATCTCCAAGATCCGTGAGGAGTTCCCCGACC
GCATGATGGCTACCTTCTCCGTCGTGCCCTCCCCCAAGGTCTCCGACACCGTCGTCGAGCCTTACAACGCCA
CCCTCTCCGTCCACCAGCTGGTCGAGAACTCGGATGAGACCTTCTGCATTGACAACGAGGCTCTGTACGACA
TCTGCATGCGCACCCTGAAGCTATCCAACCCCTCGTACGGTGACCTGAACCACCTGGTCTCGGCTGTCATGT
CTGGTGTCACCACTTGCTTGCGTTTCCCCGGCCAGCTGAACTCTGACCTTCGCAAGCTCGCCGTGAACATGG
TTCCCTTCCCTCGTCTCCACTTCTTCATGGTCGGCTTCGCCCCTCTGACCAGCCGTGGCGCGCACTCCTTCCGC
GCCGTCACCGTTCCCGAGCTCACTCA-GCAGAT-
GTTCGACCCCAAGAACATGATGGCTGCCTCTGACTTCCGCAACGGTCGCTACCTGACCTGCTCTGCCATCTT
GTAAGATGAT-CC----TCTTGA-------------CTATT-------------------------TATGAATGC-TAT---
GTTACTAACTTG----GTTCCTCTAGCCGTGGTAAGGTCTCCA--

>H_lechatii_MUCL_54609

CTGATAAACGAAAACCCCCTGA-----ACGCG-TCCC--------------AAAAA-------------CTC---CAAA--ACCCCC-
TGAT------C------T-TCTGCCCCT-----CATGTAC---------ACA--AAC-
-CGCCACTACAACG-------------------------------TGCAT----CT-----------CGTG---TTAT------ATCATCACCAGA-
TTA-----TA------------C---AGC------A--A------------------------TAGCTTGAAA---------
AAAAAACATCTAAACTAA-CCGCG-----------TTTT------------
CTCAATAGGTTCATCTTCAGACTGGCCAATGTGTAAGTGAACAAG-------------------GACGATC----
GATAAACCGTG-----------------ATCCAAC---------------AAGT-ATAGCGG-----GGCTCACGAAC---A-ACG-----
TA-TAGGGTAACCAAATTGGTGCCGCCTTCT--
-GGCAAACCATCTCCAGCGAGCACGGCCTCGACAGCAATGGCGTGTATGTATTT----------------------
GAGTCGTC-------------------AATTGCTACTACC-------AAAGA---------------------------AGT--CAAC-------TAAC-
----------CGC-CAAT-
AAATAGCTACAATGGAACCTCCGAGCTCCAGCTTGAGCGCATGAGCGTCTACTTCAACGAGGTACGAGTT-
TATAA-----------------G----------A----GCT-----GG-TTGC--------------------GTGTAA-AT-A--------TAACCAA-TG-
A---AGCTAAT--CGC-CT-CTA---
CGTATAATAGGCTTCTGGCAACAAGTATGTCCCTCGCGCCGTTCTCGTCGATCTCGAGCCCGGTACCATGGA
TGCCGTCCGTGCTGGTCCCTTTGGTCAGCTTTTCCGACCCGACAACTTTGTCTTCGGTCAGTCCGGTGCTGGC
AACAACTGGGCCAAGGGTCACTACACTGAGGGTGC-CGAGTTAGTCG-
ACAACGTCCTCGATGTCGTTCGTCGTGAGGCTGAAGGCTGTGACTGCCTCCAGGGTTTCCAGATCACCCACT
CTCTCGGTGGTGGTACTGGTGCTGGTATGGGTACTCTGTTGATCTCCAAGATCCGCGAAGAGTTCCCCGACC
GCATGATGGCTACCTTCTCCGTCATGCCCTCGCCTAAGGTCTCTGATACCGTCGTCGAGCCCTATAACGCCAC
CCTCTCCGTCCACCAGCTCGTCGAGAACTCCGACGAGACCTTCTGTATCGACAACGAGGCCCTTTACGATAT
CTGCATGCGTACTCTCAAGTTATCTAACCCTTCGTATGGCGATCTTAACCACCTCGTCTCCGCCGTCATGTCC
GGCGTTACCACTTGCTTGCGTTTCCCCGGTCAGCTAAACTCTGACCTTCGCAAGCTCGCCGTGAACATGGTT
CCTTTCCCTCGTCTACACTTCTTCATGGTTGGCTTCGCACCTCTTACCAGCCGTGGCGCTCACTCTTTCCGCGC
TGTTACCGTTCCCGAGTTGACCCA-GCAGAT-
GTTCGACCCCAAGAACATGATGGCTGCTTCCGACTTCCGTAACGGTCGTTACCTGACTTGCTCTGCCATCTTG
TAAGATGAT-AC----CGCTCCTAA----------TTATT-------------------------GCTCGTCGTCAAG---
TTTGCTAACCTC----GTTTTCCCAGCCGTGGCAAGGTCTCGATGAAGGAAGTCGAAGACCAGATGCGGAAC--
----

>H_hainanense_FCATAS2712

--TCATGCGTGAGATTGTAAGTTGATAAC---------------------------------------TTTATATTTACT----------------
CTTG---------------AATCCTCAGCCGAATACAGCTAGAGAACCCCTGA-----ACGCG-TTCG--------------AAAAC-
------------TCC---AAACCCCCCCCC-CTGA------T------T-CCTGCCCCT-----CATGCAC---------ACA-----------------
------------------------------------ATG--TGCCACCACA-------------------------TCT------CATCA----CA-----TTC---
TTTA---ATTC------GTTGTTATCAGA-CTG-----CA------------T---AGC------ATAGCA---GCTGTACCATT-------
AAATCGTGGCG---------AACAATACCAGAAGCTAA-CATTG---CC---TTTTTCT------------
CTCGATAGGTTCACCTCCAGACCGGCCAATGCGTAAGTGCCACGAA---AACC----------------------
AATTCAACATG-----------------GTACTG----------------GAAC-ATAGCGG-----GGCTTACACGA---G-GAT-----
TA-TAGGGTAACCAAATTGGTGCTGCCTTCT--
-GGCAAACCATCTCCAGCGAGCACGGCCTCGACAGCAACGGCGTGTACGTATCT-----------------------
GAGTCGTC-------------------GATTAGCAACATT-------GAGAA---------------------------TCC--TGAC-------TGAC-
----------AAT-CAAT-
AAATAGCTACAATGGAACCTCGGAGCTCCAGCTCGAGCGCATGAGCGTTTACTTCAACGAGGTAGGGCTA-
CGTAG-----------------A----------A----ATG-----AA-GAAC--------------------GATAGT-TTTA-------CGGATCTG-
TT-G-----CTAAT--CAA-CC-CGA---CCCAC-
GCAGGCCTCTGGTAACAAGTATGTTCCTCGCGCTGTCCTCGTCGATCTCGAGCCCGGCACCATGGACGCCGT
CCGTGCTGGTCCCTTCGGCCAGCTCTTCCGACCCGACAACTTCGTTTTTGGCCAGTCTGGCGCCGGAAACAA
CTGGGCCAAGGGTCACTACACCGAGGGTGC-TGAGCTCGTCG-
ACAACGTCCTTGATGTTGTTCGTCGCGAAGCTGAGGGCTGCGACTGCCTTCAGGGTTTCCAGATCACTCACT
CTCTCGGTGGTGGTACCGGTGCCGGTATGGGTACTCTTCTGATCTCCAAGATCCGCGAAGAGTTCCCCGACC
GCATGATGGCTACTTTCTCCGTCATGCCCTCCCCTAAGGTCTCTGACACCGTCGTCGAGCCCTACAACGCCAC
CCTCTCCGTCCACCAGCTGGTCGAGAACTCGGACGAGACCTTCTGCATTGACAACGAGGCTCTCTACGACAT
CTGCATGCGCACCCTGAAGTTGTCCAACCCTTCGTACGGTGACCTGAACCACTTGGTCTCTGCCGTCATGTCC
GGCGTCACCACTTGCCTGCGTTTCCCCGGTCAGCTAAACTCTGATCTGCGCAAGCTCGCCGTGAACATGGTT
CCCTTCCCTCGTCTTCACTTTTTCATGGTTGGCTTCGCTCCCCTGACGAGCCGTGGCGCCTACACCTTCCGTGC
CGTCACCGTCCCTGAGTTGACTCA-GCAGAT-
GTTCGACCCCAAGAACATGATGGCTGCTTCCGATTTCCGCAACGGTCGCTACCTGACGTGCTCTGCCATCTT
GTATGATACC-CC----TTATATT------------ATATCAT------------------------TTCTGTTAC-AGA---
TTTGCTAACTTG----
AGTTTCCTAGCCGTGGCAAGGTCTCCGTCAAGGAGGTTGAGGACCAGATGCGCAACGTCCAGAACAAGAG
TCGCCAGTT--------------------------------

>H_lividipigmentum_BCRC_34077

-
AACATGCGTGAGATTGTAAGTGATATATTTCCCTACCTAACACGCTACACACTTCCCTGTGTACCTGTCTACA
TCGACT----------------CCTGAATCGACCT---------------GGGCACCGCTACAGAAAACCCCTGA-----ACGCG-
TCTG--------------AAAAC-------------TGC---AAAAACACATCC-TTGA------T------A-TCTGCCCCT-----
CATGCAT---------TCA---ATG--
TGCCACCACATCCCATCCCATCCCATCCCATCCCATCT------CGTCG----TC-----CTC---TCTC---ATTC------
GCCTCTGTTCAGCCTG-----CA------------C---GCC------A--T------------------------CATGAC--------------
CAAGACATCGAAGCTAA-CCATA---TC--TCTTTTCT------------
CTCCGTAGGTTCATCTTCAGACCGGCCAATGCGTAAGTGCCACGAA---TACC----------------------
GACGGAACGGA-----------------GTATTG----------------GGAC-ACAGCGG-----GGCTTACATA------GGT-----

CG-CAGGGTAACCAAATTGGTGCTGCTTTCT---
--GGCAGACCATCTCCAGCGAGCACGGTCTCGACAGCAATGGCGTGTACGTATTC----------------------
GAGAAGTT------------------CAATCCGCGTTGCC-------AGGGA--------------------------TGT--CGAC-------
TGAC-----------GGC-CAAT-
AAACAGCTACAACGGAACCTCGGAGCTCCAGCTCGAGCGCATGAGCGTCTACTTCAACGAGGTATGGATC-
TGCGA-----------------G----------A----GCTG----GG-GGGT--------------------GGATAT-GT-A--------GAATCAT-
TT-G-----CTGAT--CAT-CT-CGA---CCTGC-
GCAGGCTTCCGGTAACAAGTATGTCCCTCGCGCCGTCCTCGTCGATCTCGAGCCCGGCACCATGGACGCCG
TCCGCGCTGGCCCCTTCGGTCAGCTTTTCCGACCCGACAACTTCGTTTTCGGCCAGTCGGGTGCCGGCAACA
ACTGGGCCAAGGGTCACTACACTGAGGGTGC-CGAGCTCGTCG-
ACAACGTCCTCGACGTCGTCCGCCGTGAGGCTGAGGGCTGCGACTGCCTCCAGGGCTTCCAGATCACCCAC
TCGCTCGGTGGTGGTACCGGTGCTGGTATGGGTACCCTGCTGATCTCCAAGATCCGCGAGGAGTTCCCCGA
CCGCATGATGGCCACCTTCTCCGTCATGCCCTCCCCAAGGTCTCCGACACCGTTGTCGAGCCCTACAACGC
CACCCTCTCGGTCCACCAGCTGGTCGAGAACTCGGACGAGACCTTCTGCATTGACAACGAGGCTCTGTACG
ACATCTGCATGCGCACCCTGAAGTTGTCCAACCCCTCGTACGGTGACCTGAACCACCTGGTCTCTGCCGTCA
TGTCCGGCGTCACTACCTGCCTGCGTTTCCCCGGTCAGCTGAACTCTGACCTGCGCAAGCTCGCCGTGAACA
TGGTTCCCTTCCCTCGTCTACACTTCTTCATGGTCGGCTTCGCCCCCCTGACCAGCCGTGGCGCCTACACCTT
CCGAGCCGTCACCGTCCCCGAGCTGACCCA-GCAGAT-
GTTCGACCCCAAGAACATGATGGCTGCCTCCGACTTCCGCAACGGTCGCTACCTGACGTGCTCTGCCATCTT
GTATGATAGC-CC----CGTCTGT------------CTTTT--------------------------CTCTGTGAA-CAT---
GATGCTAACCTA----
AAGTAATCAGCCGTGGCAAGGTCTCCGTCAAGGAGGTTGAGGACCAGATGCGCAACGTCCAGAACAAGAA
CTCGACCTACTTCGTTGAGTGGATTCCCAACAACATCCAGA

>H_lividipigmentum_STMA_14045

---TCGACT----------------CCTGAATCGACCC-
GGG--------GCACCGCTACAGGAAACCAAACCCCTGA-----ACGCG-TCTG-------------AAAAC------------TGC-
--AAAA--ACATCC-TTGA------T------A-TCTGCCCCT-----CATGCAT---------CCA-------------------------------------
----------------ATG--TGCAACCACATGCCATGCCATGTCATCCTATCCCATGT------CATTA----TC-----CCC---
TCTT---ATTC------GCCTCTATTCAGCCTG-----CG-----------T---GCC------A--T------------------------CATGAT---
-----------CAAGACATCAAAGCTAA-CCGCG---TC---TCTTTT-------------
CTCTATAGGTTCATCTTCAGACCGGCCAATGCGTAAGTGCCACGAA---TACC----------------------
GACGAAACGCG-----------------GTACTG----------------GGAG-GCAGCGG-----GGCTCACATG------GGT-----
TG-CAGGGTAACCAAATCGGTGCCGCTTTCT---
--GGCAGACCATCTCCAGCGAGCACGGTCTCGACAGCAATGGCGTGTATGT-TCT----------------------
GAGAAGTC-------------------AATCCGCGGTACC-------AGGGA--------------------------TGT--CGGC-------
TAAC-----------GAC-CAAT-
AAACAGCTACAACGGAACCTCGGAGCTCCAACTCGAGCGCATGAGCGTCTACTTCAACGAGGTATGGATC-
TACRA-----------------A----------G----CTG-----GA-GTGT--------------------GGACAT-GT-A--------GAATGAT-
TT-G-----CTGAT--CGC-CCACGA---CCTAC-
GCAGGCTTCCGGCAACAAGTATGTTCCTCGCGCCGTTCTCGTCGATCTCGAGCCCGGCACCATGGATGCCGT
CCGTGCTGGCCCCTTCGGCCAGCTTTTCCGACCGGACAACTTCGTTTTCGGCCAGTCGGGTGCCGGCAACAA
CTGGGCCAAGGGTCACTACACTGAGGGTGC-TGAGCTCGTCG-
ACAATGTTCTCGACGTTGTCCGCCGCGAGGCCGAGGGCTGCGACTGCCTCCAGGGCTTCCAGATCACCCAC

TCGCTCGGTGGTGGTACCGGTGCCGGTATGGGTACCCTGCTGATCTCCAAGATCCGCGAAGAGTTCCCCGA
CCGCATGATGGCCACCTTCTCCGTCATGCCCTCCCCCAAGGTCTCCGACACCGTCGTCGAGCCTTACAACGC
CACCCTCTCTGTCCACCAGCTGGTCGAGAACTCGGACGAGACCTTCTGCATTGACAACGAGGCTCTGTACGA
CATCTGCATGCGCACCCTGAAGTTGTCCAACCCCTCGTACGGTGACCTGAACCACCTGGTCTCTGCCGTCAT
GTCCGGCGTCACAACCTGCCTGCGTTTCCCCGGTCAGCTGAACTCYGACCTGCGCAAGCTCGCCGTGAACAT
GGTTCCCTTCCCTCGTCTCCACTTCTTCATGGTCGGCTTCGCTCCCCTGACCAGCCGTGGCGCCTACACCTTCC
GAGCCGTCACCGTTCCCGAGCTGACCCA-GCAGAT-
GTTCGACCCCAAGAACATGATGGCTGCCTCTGACTTCCGCAACGGTCGCTACCTGACGTGCTCCGCCATCTT
GTATGATACC-CT----CCTCTACTTATCT-----GTTTTC-------------------------CGCTATGAA-TAT---
GCTGCTGACCTA----
AAGTAATTAGCCGTGGCAAGGTCTCCGTCAAGGAGGTTGAGGACCAGATGCGCAACGTCCAGAACAAGAT-
----

>H_barbarensis_STMA_14081

---CCAA---GCCCC-TTGA------T------T-TCTGCCCCT-
----CACGCCC---------ACA---AAT--CACCACAATAA-------------------
---CAATC------CAGCA----CT-----CTC---TACG---CTATG-----CTTATTGCGAAC-TTG-----CA-----------C---
AAC------T--T-----------------------CGTGAT---------------GGAATTTTGAAACTAACTTGTG---TC---TTTTCAT---
---------GTCGATAGGTTCACCTTCAGACCGGCCAGTGCGTAAGTACTATCTA----------------C-GATTCCC----
GACGAAACATG-----------------GCGCGAG---------------AGAC-ATAGTGGG----GGCTCACATAA---A-TAT-----
TA-TAGGGTAACCAAATTGGTGCTGCTTTCT---
-GGCAAACTATCTCTGGCGAGCATGGTCTCGACAGCGATGGTGTGTACGTATAG-----------------------
CAATGGTC-------------------AATTCAGAGGCTT-------CGGAA-------------------------GTAT--GAAC-----------T--
--------GAC-CAAT-
CAACAGCTACAACGGTACCTCTGAGCTCCAGCTTGAGCGCATGAGCGTCTACTTCAACGAGGTATGTAGC-
TTCAG-----------------A----------A----ATC-----CA-ACAT--------------------GGATAC-AC-A--------AGATCGG-CT-
G-----CTAAT--CAT-CCCTGA---TATCT-
ACAGGGTTCTGGCAACAAGTATGTTCCTCGCGCTGTTCTCGTCGATCTCGAGCCCGGCACCATGGATGCTGT
CCGTGCTGGTCCTTTCGGCCAGCTTTTCCGACCTGACAACTTCGTCTTTGGCCAGTCCGGTGCCGGCAACAA
CTGGGCCAAGGGTCACTACACTGAGGGTGC-TGAGCTTGTTG-
ACCAGGTCCTCGATGTCGTTCGTCGTGAGGCTGAAGGCTGTGATTGCCTTCAGGGTTTCCAGATCACCCACT
CGCTCGGTGGTGGTACCGGTGCCGGTATGGGTACTCTGTTGATCTCCAAGATCCGTGAAGAGTTCCCCGAC
CGAATGATGGCCACCTTCTCCGTCGTTCCCTCCCCCAAGGTCTCCGATACCGTTGTCGAGCCTTACAACGCCA
CCCTCTCCGTCCACCAGCTGGTCGAGAACTCTGACGAGACTTTCTGTATCGACAACGAGGCGCTGTACGACA
TCTGCATGCGTACCCTGAAGCTGTCCAACCCCTCTTACGGTGACCTGAACCACCTCGTCTCCGCCGTCATGTC
CGGTGTCACCACCTGCCTGCGTTTCCCTGGTCAGCTGAACTCTGACCTGCGCAAGCTCGCCGTGAACATGGT
TCCTTTCCCTCGTCTCCACTTCTTCATGGTTGGATTCGCTCCTCTGACCAGCCGCGGCGCGTACTCTTTCCGTG
CCGTCACCGTCCCCGAGTTGACCCA-GCAGAT-
GTTCGACCCCAAGAACATGATGGCTGCTTCCGACTTCCGCAATGGTCGCTACCTGACGTGCTCTGCCATCTT
GTAGGATACC-AT----CCCTGAATC----------CCTTT-------------------------TTTTCCTGC-TAA---
TGTGCTAACTTGA--A--

>H_submonticulosa_CBS_115280

---A-----------------------------------
GCCGACTCGGCTACAGCATTGAGAGATACCCCTGA-----ACGCG-TCCG--------------AAAAAAA-----------
ACT---CCAA---ACCCC-TTGA------T------T-TCTGCCCCT-----CACACAC-------ACACA------------------------------
-----------------------AAT--CACCACAATAA----------------------CAATC------CAGAC----CT-----CTC---TACG---
ATAC------GCTATTGCGAAA-TTC-----TA------------C---AAC------G--C------------------------TTTGAT-------------
--GAGAATTTGAAGCTAA-CTACG---TA---TTTTCAT------------
GTCAACAGGTTCACCTTCAGACCGGCCAGTGCGTAAGTACTATCTA----------------C-GATTCCC----
GACAAAACATG-----------------GCGCGAG---------------AAAC-ATAGTGGG----GGCTCACATAT---A-TAT-----
CC-TAGGGTAACCAAATTGGTGCTGCTTTCT--
-GGCAAACTATCTCTGGCGAGCATGGCCTCGATAGCGATGGTGTGTACGTATAT----------------------
CAATGGTC-------------------AATTTAGAGCCTC-------AAAAATA-----------------------------TATC-------GACT---
--------GAC-CAAT-
AAACAGCTATAACGGTACCTCGGAGCTCCAGCTCGAGCGCATGAGTGTCTACTTCAACGAGGTATGTAGC-
TCCAG-----------------A----------A----ATC-----CA-GTAT--------------------GGATAT-AT-A--------AAATCAC-CT-
A-----CTAAT--TAT-CCCGGG---CATCT-
ACAGGGTTCTGGCAACAAGTATGTTCCTCGCGCCGTTCTCGTCGATCTCGAGCCCGGCACCATGGACGCCGT
CCGTGCTGGTCCTTTCGGCCAGCTTTTCCGACCTGACAACTTCGTCTTTGGCCAGTCCGGTGCCGGCAACAA
CTGGGCCAAGGGTCACTACACTGAAGGTGC-TGAGCTTGTCG-
ACCAAGTCCTCGATGTCGTTCGTCGTGAGGCTGAAGGCTGCGACTGCCTTCAGGGCTTCCAGATCACCCACT
CGCTTGGTGGTGGTACCGGTGCCGGTATGGGTACTCTGTTGATCTCCAAGATCCGTGAAGAGTTCCCCGAC
CGAATGATGGCCACCTTCTCCGTCGTTCCCTCCCCCAAGGTCTCCGACACCGTCGTCGAGCCTTACAACGCC
ACCCTCTCCGTCCACCAGCTGGTCGAGAACTCTGACGAGACCTTCTGTATCGACAACGAGGCTCTGTACGAC
ATCTGCATGCGTACTCTGAAGCTGTCCAACCCCTCTTACGGTGACCTGAACCACCTAGTCTCCGCCGTCATGT
CCGGCGTTACCACCTGCTTGCGTTTCCCCGGTCAGCTGAACTCTGACCTGCGCAAGCTCGCCGTGAACATGG
TTCCTTTCCCTCGTCTCCACTTCTTCATGGTTGGATTCGCTCCTCTGACCAGCCGTGGCGCGTACTCTTTCCGT
GCCGTCACCGTTCCCGAGTTGACCCA-GCAGAT-
GTTCGACCCCAAGAACATGATGGCTGCCTCTGACTTCCGCAACGGTCGCTACCTGACGTGCTCTGCCATCTT
GTAGGATACC-CT----CTCCGAA------------TTCTT--------------------------TTTGCTACT-AAT---
TTTACTAACTTG----ATATTTCCAGCCGTGGCAAGGTCTCCATGAAGGAGGTTGAGGACC----------------------
----

>H_monticulosa_MUCL_54604

----------ACCCCTGA-----ACGCG-TCCG--------------AAAAA-------------ACT---CCAA---GCCCC-TTGA------T----
--T-TTTGCCCCT-----CACGCAC---------ACA--AAA--
CACCACAAGAG-----------------------CAATC------TAGAT----AT-----GTC---TACG----------
ATTGCTACCATGGAA-TCG-----TA------------CA-TAAC---------A-------------------------AGTATT---------------
GGAAATTTGAAACTAA-CCATG---TC---TTTTCAT------------
CTCGATAGGTTCACCTCCAGACCGGCCAGTGCGTAAGTACTACCTA----------------C-GATTACC----
GACGAAACATC-----------------GCGAAAA---------------ACAT-AGTGGGA-----GGCTCACATAA---A-TAT-----
TG-TAGGGTAACCAAATTGGTGCTGCTTTCT--
-GGCAAACTATCTCTGGCGAGCACGGCCTCGACAGCGATGGTGTGTACGTATAT----------------------
CAATGGTC-------------------AATTCAAAGCTCC-------GAAA---------------------------------TAAC-------AACT-----
------GAC-CAAT-

AAACAGCTACAACGGTACCTCTGAGCTCCAGCTCGAGCGCATGAGCGTCTACTTCAACGAGGTATGTAGC-
AACAG-----------------A----------A----ATC-----CC-GTAT--------------------AGATAT-AC-A--------AGATCGG-CT-
A-----CTAAT--CAC-CCCTGA---TATGT-
ACAGGGTTCTGGCAACAAGTATGTTCCTCGCGCTGTTCTCGTCGATCTCGAGCCCGGTACCATGGATGCCGT
CCGTGCTGGTCCCTTCGGCCAGCTTTTCCGACCTGACAACTTCGTCTTTGGCCAGTCCGGTGCCGGCAACAA
CTGGGCCAAGGGTCACTACACTGAGGGTGC-TGAGCTTGTTG-
ACAACGTTCTCGATGTCGTTCGTCGTGAGGCTGAAGGCTGCGACTGCCTTCAGGGTTTCCAGATTACCCACT
CGCTCGGTGGTGGTACCGGTGCCGGTATGGGTACTCTGCTGATCTCCAAGATCCGTGAAGAGTTCCCCGAC
CGAATGATGGCCACCTTCTCCGTCGTTCCCTCCCCCAAGGTCTCTGACACCGTTGTCGAGCCTTACAACGCTA
CCCTTTCCGTCCACCAGCTTGTCGAGAACTCCGACGAGACCTTCTGTATCGACAACGAGGCGCTGTACGATA
TCTGCATGCGTACCCTGAAGCTATCCAACCCCTCGTATGGTGACCTAAACCACCTGGTCTCCGCCGTCATGTC
CGGTGTCACCACCTGCTTGCGTTTCCCTGGTCAGTTGAACTCTGACCTGCGCAAGCTTGCTGTGAACATGGT
TCCTTTCCCCCGTCTCCACTTCTTCATGGTTGGCTTCGCTCCTCTGACCAGCCGTGGCGCGTACTCTTTCCGTG
CCGTCACCGTTCCCGAGTTGACCCA-GCAGAT-
GTTCGACCCCAAGAACATGATGGCTGCTTCTGACTTCCGTAACGGTCGTTACCTGACGTGCTCTGCCATCTT
GTAAGATGCC-TC----TTTCGAACCC--

>H_crocopeplum_CBS_119004

ATACGAACCCGAACACCAAATTTTGGCCCCCTGA-----ACGCG-TCCG--------------AAATC-------------CTG---
CGAC-GCCCCCC-TGAT------T------T-TGTGCCCCT-----CACGCAC---------AAA--------------------------------------
----------------CCA--CTATACATAAA----------------------CATCA------TACAG----CT-----ATC---TATA---TCAA----
--GCTGTTTTTAAT-TTA-----CA------------C---AAC------G--A------------------------CATGAT---------------
GGAGAATTGAAGCTAA-CTATG---TC---TCTTTAT------------
CATTATAGGTTCACCTTCAGACCGGCCAGTGCGTAAGTATAACGACAA---------------------------
CACAAAAGATC-----------------GCGATG----------------GAAC-ATAGCAG-----GGCTCACACGA---T-ACT-----
AA-TAGGGTAACCAAATTGGTGCTGCTTTCT--
-GGCAAACCATCTCTGGCGAGCACGGCCTCGACAGCAATGGCGTGTACGTATCT-----------------------
AATTCGGC-------------------AATTCATAAGAC--------AAGAG---------------------------TGG--AAAC-------TGAC-
----------CGC-CAAT-
CAACAGCTACAACGGAACTTCCGAGCTCCAGCTTGAGCGCATGAGCGTCTACTTCAACGAGGTACGCCCT-
CATCG-----------------A----------A---ACT-----GA-----------------------TTAAACGC----G--------AAAGACT-TG-G-
----CTAAT--CAC------T---ACTAT-
ATAGGCTTCTGGCAACAAGTATGTTCCTCGCGCTGTCCTCGTCGATCTCGAGCCTGGTACCATGGATGCCGT
CCGTGCTGGTCCCTTCGGTCAGCTCTTCCGACCTGACAACTTCGTCTTCGGCCAGTCTGGTGCCGGCAACAA
CTGGGCCAAGGGTCACTACACCGAGGGTGC-CGAGCTCGTTG-
ACCAGGTTCTCGATGTCGTCCGTCGTGAAGCTGAAGGCTGCGACTGCCTTCAGGGCTTCCAGATCACCCACT
CCCTCGGTGGTGGTACTGGTGCTGGTATGGGTACCCTGTTGATCTCCAAGATCCGCGAGGAGTTCCCTGAC
CGCATGATGGCCACCTTCTCTGTCGTTCCCTCGCCCAAAGTTTCCGACACTGTCGTCGAGCCTTACAACGCCA
CCCTCTCCGTCCACCAGCTGGTCGAGAACTCGGACGAGACCTTCTGCATTGACAACGAGGCTCTGTACGACA
TCTGCATGCGTACCCTTAAGCTATCCAACCCCTCGTACGGCGACCTGAACTACCTGGTATCCGCTGTCATGTC
TGGTGTCACCACTTGCCTGCGTTTCCCCGGTCAGCTGAACTCGGACCTGCGCAAGCTTGCCGTCAACATGGT
TCCCTTCCCTCGTCTGCACTTCTTCATGGTCGGCTTCGCTCCCTTGACCAGCCGTGGTGCTCACTCGTTCCGTG

CTGTCACCGTCCCCGAGTTGACTCA-GCAGAT-
GTTCGACCCCAAGAACATGATGGCTGCCTCTGATTTCCGCAACGGCCGCTACCTGACTTGCTCTGCCATCTT
GTAAGATACA-TT----TCCCTAC------------TGGTT-------------------------CTGTCACGT-TTA---
TTTGCTAACCTG----
TCTCTTCCAGCCGCGGCAAGGTCTCGATGAAGGAGGTTGAGGACCAGATGCGCAATGTCCAGA-------------
----

>H_fendleri_MUCL_54792

--AATTTTGGCCCCCTGA-----ACGCG-TCCC--------------AAATC-------------CTC---CAAT---GCCCC-TGAT------
T------T-CCTGCCCCT-----CACGCAC---------ACA---AAAA-
AAACATCACA--------------------CAACACTT------GATAG-----------CTA---TATA---TTGA------
GCTGCTATTTAA-TTA-----CA------------C---AAT------G--A-----------------------CATGAT--------------
AAGGAATTGAAGCTAA-CCATC---TT---TTTTTGTC-----------
CACTATAGGTTCACCTCCAGACCGGCCAGTGCGTAAGTAACACGAC---CATC-----------------------
AACGAATATT-----------------GCGATG----------------AGAC-ATTGCGG-----GGCTCACACGA---A-GTA-----AA-
TAGGGTAACCAAATTGGTGCTGCCTTCT---
GGCAAACCATCTCTGGCGAGCACGGCCTCGACAGCAATGGCGTGTACGTAAATC---------------------
AATTCGCC-------------------AATTCCTATCGAG-------AGGAT--------------------------AAC--CAAC-------TAAT--
---------GGT-CAAT-
AAACAGCTACAACGGTACTTCCGAGCTCCAGCTCGAGCGCATGAGCGTCTACTTCAACGAGGTATGCACA-
GATACCGA--------------AG---------A----ATT-----GG-GAAA--------------------AGATGC-AA-A--------
AGATTGG-TC-GGTTA-TTAAT--CAT-CA-TA----------
ACAGGCTTCCGGCAACAAGTATGTTCCTCGCGCCGTCCTCGTCGATCTCGAGCCCGGTACCATGGATGCCGT
CCGTGCTGGTCCCTTCGGTCAGCTCTTCCGACCCGACAACTTCGTCTTCGGTCAGTCTGGTGCCGGAAACAA
CTGGGCCAAGGGTCACTACACTGAGGGTGC-TGAGCTTGTTG-
ACAACGTCCTCGATGTCGTTCGTCGTGAGGCTGAGGGTTGTGATTGCCTTCAGGGTTTCCAGATCACCCACT
CCCTCGGTGGTGGCACTGGTGCTGGTATGGGTACTTTGTTGATCTCCAAGATCCGTGAGGAGTTCCCCGAC
CGAATGATGGCCACCTTTTCCGTTGTTCCCTCTCCCAAGGTCTCCGACACCGTCGTCGAGCCTTACAACGCCA
CCCTCTCGGTCCACCAGCTGGTCGAGAACTCGGACGAGACCTTCTGCATTGACAACGAGGCTCTGTACGAC
ATCTGCATGCGTACCCTCAAGCTGTCTAACCCCTCCTATGGCGACCTGAACCACCTCGTCTCCGCTGTCATGT
CTGGTGTCACCACTTGCCTGCGTTTCCCCGGTCAGCTGAACTCTGACCTGCGCAAGCTTGCCGTCAACATGG
TTCCCTTCCCTCGTCTGCACTTCTTCATGGTTGGCTTCGCTCCTTTGACCAGCCGTGGCGCCCACTCTTTCCGC
GCCGTCACCGTCCCCGAGTTGACCCA-GCAGAT-
GTTCGACCCCAAGAACATGATGGCTGCTTCTGACTTCCGCAACGGTCGCTACCTGACGTGCTCTGCCATCTT
GTGAGATATT-TA----TTCCAGC------------TAACC--------------------------CGTTTTCGC-ATA---
TTTGCTAACCCG----TATTCTGTAGCCGTGGCAAGGTCTCCAT--

>H_erythrostroma_MUCL_53759

------------CCCTGA-----ACGCG-TCCG-------------CAAATC-----------CTG---CAAC--GCCTCC-TGAT------T-----
-T-CGTGCCCCT-----CACGCAC---------ACA---AAA--
CACCAAAAAAA-------------------TAATATCC------CACAG----CA-----AGC---TATG----------

CGAACGATCATCGAA-CCA-----CACA-------TATC---AAC------G--A-----------------------TGAGAT---------------
-GGAAATTGATGCTAA-CTGTA---TC---TT----------------
TCATATAGGTTCACCTTCAGACCGGCCAGTGCGTAAGTAGATATCTC----------------GAGCAA-----
GAACCAAGATA----------------GCGATGA--------------AACG-TTTGCGG-----GGCTCACACAA---T-GCT-----
TG-TAGGGTAACCAAATTGGTGCTGCTTTCT--
-GGCAGACCATCTCTGGCGAGCACGGCCTCGACAGCAATGGCGTGTACGTATAT----------------------
AATTCGCC------------------AATTCAGTCTT---------GAAGC-------------------------TCG--TAAC-------TAAC----
-------GAC-CAAT-
AAATAGCTACAACGGAACTTCTGAGCTCCAGCTCGAGCGCATGAGCGTCTACTTCAACGAGGTACGAACC-
CGTAG----------------A----------A----CCC-----GC-GAAT------------------AGATAACGA-A--------GGAGACT-
CT-G-----CTAAT--TAC----CAA---CAAAC-
ACAGGCTTCTGGCAACAAGTATGTTCCTCGCGCTGTCCTCGTCGATCTCGAGCCCGGTACCATGGACGCCGT
CCGCGCTGGTCCCTTTGGTCAGCTCTTCCGACCCGACAACTTCGTCTTCGGCCAGTCTGGTGCCGGAAACAA
CTGGGCCAAGGGTCACTACACTGAGGGTGC-TGAGCTTGTCG-
ACAACGTCCTCGACGTCGTCCGTCGCGAGGCTGAGGGATGCGACTGCCTTCAGGGCTTCCAGATCACTCAC
TCTCTCGGTGGTGGTACCGGTGCTGGTATGGGTACTCTGCTTATCTCCAAGATCCGCGAGGAGTTCCCCGAC
CGCATGATGGCTACTTTCTCCGTCGTCCCCTCGCCCAAGGTCTCTGACACCGTCGTCGAGCCTTACAACGCCA
CTCTCTCCGTCCACCAGCTGGTCGAGAACTCGGACGAGACCTTCTGCATCGACAACGAGGCTCTGTACGATA
TCTGCATGCGTACTCTCAAGCTATCCAACCCCTCGTATGGTGATCTGAATCACCTGGTCTCTGCTGTCATGTC
TGGCGTCACCACTTGCCTGCGCTTCCCCGGTCAGCTCAACTCTGACCTGCGCAAGCTTGCCGTCAACATGGT
TCCCTTCCCTCGTCTGCACTTCTTCATGGTCGG-
TTTGCCCCCTTGACCAGCCGTGGTGCTCACTCTTTCCGTGCCGTCACTGTTCCTGAGTTGACTCA-GCAGAT-
GTTCGACCCCAAGAACATGATGGCTGCTTCTGACTTCCGCAACGGTCGCTACCTGACCTGCTCTGCCATCTT
GTAAGATAAT-CC----TTTCTACC-----------ATTTT-------------------------GATTCGAGT-CTA---
ATTGCTCACTTG-----TGTTCCCAGCCGTGGCAAGGTCTCCATGAAG---

>H_ferrugineum_CBS_141259

-AACATGCGTGAGATTGTAAGTCATCATCATCT--------------------------TATTCCCACTAATGTTTACCT--------
GCTGCATCCCG---------------ATTCCCGAGCACCGCCAAAATTGTGACCCCCTGA-----ACGCG-TTCG------------
--AAATT-------------CTC---CAAC--GGCCCC-TGAT------T------T-CGTGCCCCT-----CACGCAC---------AAA------
---ACA--TACAGCAAAAT-------------------GAACATGC------CACAAG---
CT-----GCT---CACG---TCGA------ACTGGGATGGAA-TTA-----CA------------C---AAC------G--A------------------
-----GATGAT----------------GGAAATGTATGCTAA-TCATA---TC---TTTTTCT-------------
CATATAGGTTCACCTCCAGACCGGCCAGTGCGTAAGTACACATCC---------------AC-GACCACC----
GATATAATACG-----------------GCGATA--------------AAAGAC-ATGGCGG-----GGCTCACACAA---T-ATT-----
CG-TAGGGTAACCAAATCGGTGCTGCTTTCT--
--GGCAGACCATCTCTGGCGAGCACGGCCTCGACAGCAATGGCGTGTACGTATAT----------------------
TATTCGCC-------------------AATGCCTCGATTT-------AACAT-------------------------CTGT--CATC-------TAAC---
--------GGC-GAAA-
TAACAGTTACAACGGCACTTCCGAGCTCCAGCTCGAGCGCATGAGCGTCTACTTCAACGAGGTATGCACT-
TACAA-----------------G----------A----TAA-----AG-AAAA--------------AAATAAGTCGC-GA-T--------
GGATACT-TT-G-----CTAAT--CAT-CA-CAA---AACAT-
GCAGGCTTCCGGCAACAAGTATGTTCCCCGCGCTGTCCTCGTCGATCTCGAACCCGGCACCATGGATGCTGT

CCGTTCTGGTCCCTTCGGTCAGCTCTTCCGACCCGACAACTTCGTCTTCGGCCAGTCTGGTGCCGGAAACAA
CTGGGCCAAGGGTCACTACACTGAGGGTGC-TGAGCTCGTCG-
ACCAAGTTCTCGATGTCGTTCGTCGCGAGGCTGAGGGCTGCGACTGCCTTCAGGGCTTCCAGATCACCCACT
CCCTCGGTGGTGGTACCGGTGCCGGTATGGGTACTCTCCTGATCTCCAAGATCCGCGAGGAGTTCCCCGAC
CGCATGATGGCTACCTTCTCCGTCGTTCCCTCTCCCAAGGTCTCCGACACCGTCGTTGAGCCTTACAACGCCA
CCCTCTCCGTCCACCAGCTGGTCGAGAACTCCGACGAGACCTTCTGCATTGACAACGAGGCTCTGTACGACA
TCTGCATGCGTACCCTCAAGCTATCCAACCCCTCGTACGGTGACCTGAACCACCTTGTCTCTGCCGTCATGTC
CGGTGTTACCACCTGCCTGCGCTTCCCCGGTCAGCTCAACTCTGACCTGCGCAAGCTTGCCGTCAACATGGT
TCCCTTCCCTCGTCTGCACTTCTTCATGGTCGGATTCGCTCCCCTGACCAGCCGTGGTGCTCACTCCTTCCGC
GCTGTCACTGTTCCCGAGTTGACTCA-GCAGAT-
GTTCGACCCCAAGAACATGATGGCTGCTTCTGACTTCCGCAACGGTCGCTACCTGACCTGCTCTGCCATCTT
GTAAGATACC-TT----TATACGTCGCT--------AGCTT-------------------------TGCTCGTCT-TCG---
TTTACTAACTCA-----
TGTTTCTAGCCGTGGCAAGGTCTCCATGAAGGAGGTTGAGGACCAGATGCGCAACGTCCAGAACAAGAAC
TCGTCGTACTTCGTCGAGTGGATTCCCAACAACATCCAGA

>H_lenormandii_CBS_119003

GAACCCAAGCCACCTCCAAATCTTGACCCCCTGA-----ACGCG-TCTG--------------AAATC-------------CTC--
CCAAC---GCCCC-TGAT------T------T-CCTGCCCCT-----CACGCAC---------AAA------------------------------------
----------------AAAATCATCACGCAA-----------------------CACCA------AACAG----CT----CATG---TTTA---CTAA--
----GCTGTTATCGAG-CTG-----CAA---------------TAC------G--A------------------------TGGAAT--------------
TGAATTATTGAAGCTAA-CCATG---TT---TTTTCAT------------
CATTATAGGTTCATCTCCAGACCGGCCAGTGCGTAAGTAACCACATG--CAAC-----------GATGTCC-----
AACGAATATC-----------------TCGATAG-------------AAAAAC-ATTGCGG-----GGCTCACACGA-----AGC-----
TA-TAGGGTAACCAAATCGGTGCTGCCTTCT--
-GGCAAACCATCTCTGGCGAGCACGGCCTCGACAGCAATGGCGTGTATGTATTT----------------------
CATGCGGC-------------------AATTCATCAAAC---------------------------------------GGC--CAAC-------TAAC------
-----CAC-CAAT-
AATTAGCTACAATGGAACTTCCGAGCTCCAGCTTGAGCGCATGAGCGTTTACTTCAACGAGGTATGCAGC-
GACGA-----------------A----------A----TCT-----TG-GAAT--------------------AGATGA-TG-A--------
AGATTGGTTT-G-----CTAAT--TAT-CA-CACC-ACATAT-
GTAGGCTTCTGGCAACAAGTATGTTCCTCGCGCCGTCCTCGTCGATCTCGAGCCCGGTACCATGGATGCCGT
CCGTGCTGGTCCCTTCGGTCAGCTTTTCCGACCCGACAACTTCGTCTTCGGCCAGTCTGGTGCCGGCAACAA
CTGGGCCAAGGGTCATTACACTGAAGGTGC-TGAGCTTGTTG-
ACCAGGTCCTTGACGTCGTTCGTCGTGAAGCTGAGGGCTGCGACTGCCTCCAGGGTTTCCAGATCACTCACT
CTCTCGGTGGTGGTACTGGTGCCGGTATGGGTACCCTGTTGATCTCCAAGATCCGTGAGGAGTTCCCTGAC
CGCATGATGGCCACCTTCTCCGTTGTTCCCTCCCCCAAGGTCTCCGACACCGTCGTTGAGCCTTACAACGCCA
CCCTCTCCGTCCACCAGCTGGTCGAGAACTCCGACGAGACCTTCTGCATTGACAACGAGGCTCTGTACGATA
TCTGCATGCGTACTCTCAAGCTATCCAACCCCTCGTACGGTGACCTGAACTACCTGGTTTCCGCCGTCATGTC
GGGTGTCACCACTTGCCTGCGTTTCCCCGGTCAGCTAAACTCTGACTTGCGCAAGCTTGCCGTCAACATGGT
TCCCTTCCCTCGTCTGCACTTCTTCATGGTCGGCTTCGCTCCCCTGACCAGCCGTGGTGCTTACTCTTTCCGTG
CCGTCACTGTTCCTGAGTTGACTCA-GCAGAT-
GTTCGACCCCAAGAACATGATGGCCGCCTCTGACTTCCGCAATGGTCGCTACCTAACATGCTCTGCCATCTT

GTAAGCTTAC-GT----TTAAATTC--GTCTTATAC-TCA---TTCGCTAACTCA--
----ATCTATAGCCGTGGTAAGGTTTCCATGAAGGAGGTCGAGGACCAGATGCGCAACGTCCAGAACAAGA--
----

>H_chrysalidosporum_FCATAS2710

AAAGCTAA-CAGCA---TC---TCTTCGTC----------
GCTCCATAGGTTCACCTCCAGACCGGCCAATGCGTAAGAAATATCCCC-----------------------------
CGACGATCAAT------------------GCGATCCCAGA----ACTGCGGGGGG-GCTTTGA-----GGCTCACGAAT---A-
TTTGATGGTA-TAGGGTAACCAAATCGGTGCTGCTTTCT--
-----------------GGCAAACCATCTCTGGCGAGCACGGTCTCGACAGCAATGGCGTGTATGTTTTTTT---------------
------CATTCTCT--------------------TATTCTCCATACC-------CAGGT---------------------------TTAT--ATAG-------
TGGCATCTATACTGACTC-GATG-
AAACAGCTACAACGGAACGTCTGAGCTCCAGCTGGAGCGCATGAGCGTTTACTTCAACGAGGTACGCTAT-
CTTAG------------------A----------A---AAA-----GA-ATAT---------------------CCATGC-GAAA--------AGTCCCG-
TT-A-----CTAAT--CAC-CC-CAA---CATGC-
ACAGGCGTCTGGTAACAAGTACGTGCCCCGAGCCGTCCTCGTCGATCTCGAGCCCGGTACCATGGACGCCG
TCCGCGCTGGTCCCTTCGGTCAGCTCTTCCGCCCCGACAACTTCGTCTTCGGCCAGTCTGGTGCCGGAAACA
ACTGGGCCAAGGGTCACTACACCGAAGGTGC-TGAGCTGGTCG-
ACCACGTCCTCGACGTCGTCCGTCGTGAGGCTGAGGGCTGTGACTGCCTTCAGGGCTTCCAGATCACCCACT
CCCTCGGTGGTGGTACCGGTGCCGGTATGGGTACTTTGTTGATCTCCAAGATCCGCGAGGAGTTCCCCGAC
CGCATGATGGCTACTTTCTCCGTCGTCCCCTCCCCCAAGGTCTCCGACACCGTTGTCGAGCCTTACAACGCTA
CCCTCTCCGTCCACCAGCTGGTCGAGAACTCGGACGAGACCTTCTGCATTGACAACGAGGCTCTGTACGACA
TCTGCATGCGTACCCTTAAGCTGTCTAACCCCTCCTACGGTGACCTGAACCACCTCGTCTCCGCCGTCATGTC
TGGCGTCACCACCTGCTTGCGATTCCCCGGTCAGCTGAACTCTGACCTGCGCAAGCTCGCCGTCAACATGGT
TCCCTTCCCCCGTCTGCACTTCTTCATGGTCGGCTTCGCTCCCCTGACCAGCCGCGGCGCCCACTCTTTCCGC
GCCGTCACCGTTCCCGAGTTGACCCA-GCAGAT-
GTTCGACCCCAAGAACATGATGGCTGCTTCTGACTTCCGCAACGGTCGTTACCTAACGTGCTCTGCCATCTT
GTAAGTTATT-TA----TATACTT-------------TGATTTCCT-------------------GCCATTGCTACAC-AAG---
TTTGCTAACTCGA--TTCTTTTCTAGCCGTGGCAAGGTCTCCATGAAGGAAGTCGAGGACCAGATGCGC-------
----

>H_cyclobalanopsidis_FCATAS2714

-------AAAACCCCTGA-----ACGCG-TTCC---------------AAAAAT-------------CTC---CAAC--GCCCCC-TGAT------
TTGC-----TATGCCCCTC----CACGCAC---------ACA--
AACAATTCGACGACAT-----------------------------------CGCAAA---CT-----ACT---CAAG---TTGT--------
TGATATTTGG--------------------------------------A----------------------------TATTATTATT----------
TTGAATATTAAAACTAA-C-------------CGTTTTT-------------
CTCGATAGGTTCACCTTCAGACCGGCCAATGCGTAAGAATGATATCC-----------------------------
CGACGACCAGC------------------GCGGTAG----------------AAAG-ATGGCGG-----GGCTCAC--GA---A-GAT-----

CA-TAGGGTAACCAAATCGGTGCTGCTTTCT--
-
GGCAAACCATCTCTGGCGAGCACGGCCTCGACAGCAATGGCGTGTATGTTTTTCTGTTATATTATATTGACT
ATATCATTGGCC-------------------AATCCTGTTACAAGAC----AAATA--------------------------TAT--
GAATATCA--ATAAC-----------TGA-CAAA-
CAACAGCTACAATGGCACCTCGGAACTCCAGCTGGAGCGCATGAGCGTCTACTTCAACGAGGTACGCTAT-
CTTGA-----------------A----------A----ATG-----CT----------------------TCCCTCGC-GCAA--------AGAACCG-TT-
A-----CTAAT--CAC-CC-CAA---CATGC-
ACAGGCGTCTGGTAACAAGTACGTGCCTCGTGCCGTCCTCGTCGACCTCGAGCCCGGCACCATGGACGCCG
TCCGTGCTGGCCCCTTTGGTCAGCTCTTCCGACCCGACAACTTCGTCTTTGGTCAGTCCGGTGCCGGAAACA
ACTGGGCCAAGGGTCACTACACCGAGGGTGC-CGAGCTGGTCG-
ACCAGGTTCTCGACGTCGTCCGTCGTGAGGCCGAGGGCTGCGACTGTCTCCAGGGCTTCCAGATCACCCAC
TCCCTCGGTGGTGGTACCGGTGCCGGTATGGGAACCTTGTTGATCTCCAAGATCCGCGAGGAGTTCCCCGA
CCGCATGATGGCTACCTTCTCCGTTGTGCCCTCTCCCAAGGTCTCTGACACCGTTGTCGAGCCTTACAACGCC
ACCCTCTCGGTCCACCAGCTGGTCGAGAACTCGGACGAGACCTTCTGCATCGACAACGAGGCTCTCTACGA
CATCTGCATGCGTACCCTCAAGCTGTCCAACCCCTCGTACGGCGACCTGAACCACCTGGTCTCCGCCGTCAT
GTCGGGCGTCACCACCTGCCTGCGATTCCCCGGCCAGCTGAACTCGGACCTGCGCAAGCTCGCCGTCAACA
TGGTTCCCTTCCCGCGTCTGCACTTCTTCATGGTCGGCTTCGCCCCCCTGACCAGCCGCGGTGCTCACTCCTT
CCGCGCCGTCACCGTGCCCGAGTTGACCCA-GCAGAT-
GTTCGACCCCAAGAACATGATGGCTGCCTCTGACTTCCGCAACGGCCGCTACCTGACGTGCTCTGCCATCTT
GTGAGTGCTC-AT----ATATCACTATTAA------------------------------------CCTCTACGT-AGC---
ATTGCTAATAAA----
GACATCACAGCCGTGGCAAGGTCTCCATGAAGGAGGTCGAGGACCAGATGCGCAACGTC----------------------
----

>H_eurasiaticum_MUCL_57720

ATA---TCGC---------------AAA-CTA--T-TCAA---------------GAT------G--A------------------------TAT-------------
TATTGGAATATGAAAAGCTAA-CCGTG---TC--TTCTTCAT------------
CTTCATAGGTTCACCTTCAGACCGGCCAATGCGTAAGAATGATAT----------------------CCCG----
ACGAAAACAAG-----------------GCGATAG--------------AAAAG-ATTGCGG-----GGCTCACACGA---A-TAT-----
CA-TAGGGTAACCAAATCGGTGCTGCTTTCT--
-GGCAAACCATCTCTGGCGAGCACGGTCTCGACAGCAATGGCGTGTATGTGTTTTG---------------
TTGTTTTATTGGTC-------------------AATTCCTTAAATGGAG----ATGAA--------------------------TATC-AAAT---
----TGACA--------AAAA--TCTA-
AAATAGGTACAATGGAACCTCGGAACTCCAGTTGGAGCGCATGAGCGTCTACTTCAACGAGGTACGCTAT-
CTTGA-----------------A----------A----AC-AA---CA-----------------------GAAAATGC-GCAA--------AGACCCG-
TT-A-----CTAAT--CAC-CC-CAA---TATGC-
ACAGGCGTCTGGTAACAAGTACGTGCCTCGTGCCGTCCTCGTCGATCTCGAGCCCGGTACCATGGATGCTG
TCCGTGCTGGTCCCTTTGGTCAACTCTTCCGACCCGACAACTTCGTCTTTGGTCAATCCGGTGCCGGAAACAA
CTGGGCGAAGGGTCATTACACCGAAGGTGC-TGAGCTGGTTG-
ACCAGGTTCTCGACGTCGTTCGTCGTGAGGCTGAGGGCTGCGATTGTCTTCAGGGTTTCCAGATCACCCACT

CGCTCGGTGGTGGTACTGGTGCCGGTATGGGTACCTTGTTGATCTCCAAGATTCGCGAGGAGTTCCCCGAC
CGCATGATGGCTACCTTCTCCGTCGTGCCCTCCCCTAAGGTCTCCGACACCGTCGTCGAGCCTTACAACGCTA
CCCTCTCCGTCCATCAGCTGGTCGAGAATTCGGACGAGACCTTCTGCATCGACAACGAGGCTCTGTACGACA
TCTGCATGCGTACCCTTAAGCTATCCAACCCCTCGTACGGTGACCTGAACCACCTGGTCTCTGCTGTCATGTC
CGGTGTTACCACTTGCTTGCGATTCCCCGGTCAGCTGAACTCTGATCTGCGCAAGCTGGCTGTCAACATGGT
TCCTTTCCCGCGTCTGCACTTCTTCATGGTCGGCTTTGCGCCCCTGACCAGCCGTGGTGCTTACACCTTCCGC
GCCGTCACCGTTCCCGAGTTGACGCA-GCAGAT-
GTTCGACCCCAAGAACATGATGGCTGCTTCTGACTTCCGCAACGGTCGTTACCTGACATGCTCTGCCATCTT
GTAAGTGACT-AT----C---

>H_fuscum_CBS_113049

----------------GTAAGTC------------------------------------ATATTCTTATTTTGTTTACAG-----------
CTTACCCGAG------------CCGAGGGCCTAGACCAGCAGGAAAAAACCCCCTGA-----ACGCG-TCCC-------------
-AAAT-------------CTC---CGAC--ATCCCC-TGAT------TTC----T-TCTACCCCTC----CACGCAC---------ACA------
---AACAATTCAACGACAA-------------------CCGCATCG------TGCCA----
TT-----CTC---TACA---TCGC---------------AAA-CTG--T-TCAA---------------TAT------G--A------------------------
TATGAT-----------ATTGGAATATTAAAAGCTAA-CCGTG---TC--TTCTTCCT------------
CTTAATAGGTCCACCTCCAGACCGGCCAATGCGTAAGATTGATAT----------------------CCCC----
GACGAAACAAG-----------------GCGATAG---------------AAAG-ATGGCGG-----GGCTCACACGA---A-TAT-----
CA-TAGGGTAACCAAATCGGTGCTGCTTTCT---
-GGCAAACCATCTCTGGCGAGCACGGTCTCGACAGCAATGGCGTGTACGTGTTGTG---------------
TTGTTTTATTGGTC-------------------AATTCTTCTCTAAGAG----ACAAA---------------------------CATCAAAAT---
----TGAC-----------AA--TCTA-
AAATAGGTACAACGGAACTTCCGAGCTTCAGCTGGAGCGCATGAGCGTCTACTTCAACGAGGTACGCTAT-
CTTGA-----------------A----------A---AAA-----CA-----------------------TGAGATGC-GCAA--------AGACCGTTTT-
A-----CTAAT--CAC-CC-CAA---CATGC-
ACAGGCGTCTGGTAACAAGTACGTGCCTCGTGCCGTCCTCGTCGATCTCGAGCCCGGTACCATGGATGCTG
TCCGTGCTGGTCCCTTCGGTCAACTCTTCCGACCCGACAACTTCGTCTTTGGTCAATCCGGTGCCGGAAACA
ACTGGGCAAAGGGTCATTACACCGAGGGTGC-TGAGCTGGTTG-
ACCAGGTTCTCGATGTCGTTCGTCGTGAGGCTGAGGGCTGCGATTGCCTTCAGGGTTTCCAGATCACCCACT
CGCTCGGTGGTGGTACTGGTGCCGGTATGGGTACCTTGTTGATTTCCAAGATTCGCGAGGAGTTCCCCGAC
CGCATGATGGCTACCTTCTCCGTCGTACCCTCCCCCAAGGTCTCCGACACCGTCGTCGAGCCTTACAACGCTA
CCCTCTCCGTCCATCAGCTGGTCGAGAACTCGGATGAGACCTTCTGCATCGACAACGAGGCTCTGTACGATA
TCTGTATGCGTACTCTGAAGCTATCCAACCCCTCGTACGGTGACCTGAACCACCTGGTCTCCGCCGTCATGTC
TGGTGTTACTACCTGCTTGCGATTCCCCGGTCAGCTGAACTCTGATCTGCGCAAGCTGGCCGTCAACATGGT
TCCTTTCCCGCGTCTGCACTTCTTCATGGTCGGCTTCGCACCCCTGACCAGCCGTGGTGCTTACACCTTCCGC
GCCGTCACCGTTCCCGAGTTGACTCA-GCAGAT-
GTTCGACCCCAAGAACATGATGGCTGCCTCTGACTTCCGCAACGGTCGTTACCTGACATGCTCTGCCATCTT
GTAAGTGACC-TA----TCTTTAT------------TAATA----------------------------TCTGCATAT-
TGAGTAAGTACTAATAAC----GACATTATAGCCGTGGCAAGGTCTCCATGAAGGAAGTTGAGGACCAGA-----
----

>H_pseudofuscum_DSM112038

-----------------------CGCG-TCCC--------------AAAT--------------CTC---CAAC--ATCCCC-TGAT------TTC----T-
TCTACCCCTC----CACGCAC---------ACA---
AACAATTCAACGACAA-------------------CCGCATCG------TGTCA----TT-----CTC---TACA---CGGC--------------
AAA-CTG--T-TCAA---------------GAT------A--A-----------------------
TATGAGAATATGAGAATATGGGAATATCAAAAGCTAA-CCGTG---TC--TTCTTCAT------------
CTTAATAGGTCCACCTTCAGACCGGCCAATGCGTAAGATTGCTATC---------------------CCCC----
GACGAAACAAG-----------------GCGATAG---------------AAAG-AGGGCGG-----GGCTCACACGA---A-TAT-----
CA-TAGGGTAACCAAATTGGTGCTGCTTTCT--
-GGCAAACCATCTCTGGCGAGCACGGTCTCGACAGCAATGGCGTGTACGTCTTCTG--------------
TTATCTTATTGGTC-------------------AATTCTGTAAGAG-------ACGAA--------------------------TATC-AAAT-----
--TGAC-----------AA--TCTG-
AAATAGGTACAACGGAACTTCGGAGCTTCAGCTGGAGCGCATGAGCGTCTACTTCAACGAGGTACGCTAT-
CTTGA-----------------A----------A----AGA-----CA----------------------TGAGATGC-GCAA--------AGACGGT-TT-
A-----CTAAT--CAC-CC-CAA---CATGC-
ACAGGCGTCTGGTAACAAGTACGTGCCTCGTGCCGTCCTCGTCGATCTCGAGCCCGGTACCATGGATGCTG
TCCGTGCCGGTCCCTTCGGTCAACTCTTCCGACCCGACAACTTCGTCTTCGGTCAATCCGGTGCCGGAAACA
ACTGGGCCAAGGGTCATTACACCGAGGGTGC-TGAGCTGGTCG-
ACCAGGTTCTCGATGTCGTTCGACGTGAGGCCGAGGGCTGCGATTGCCTTCAGGGCTTCCAGATCACCCAC
TCGCTCGGTGGTGGTACTGGTGCCGGTATGGGTACCTTGTTGATCTCCAAGATTCGCGAGGAGTTCCCCGA
CCGCATGATGGCCACCTTCTCCGTCGTGCCCTCCCCCAAGGTCTCCGACACCGTCGTCGAGCCTTACAACGC
TACCCTCTCAGTCCATCAGCTGGTCGAGAACTCGGACGAGACCTTCTGCATCGACAACGAGGCTCTGTACGA
CATCTGCATGCGTACCCTGAAGCTATCCAACCCCTCGTACGGTGACCTGAACCACCTGGTCTCCGCCGTCAT
GTCCGGTGTTACCACCTGCTTGCGATTCCCCGGTCAGCTGAACTCGGACCTGCGCAAGCTGGCCGTCAACAT
GGTTCCCTTCCCGCGTCTGCACTTCTTCATGGTCGGCTTCGCGCCCCTGACCAGCCGTGGTGCTTACACCTTC
CGCGCCGTCACCGTTCCCGAGTTGACTCA-GCAGAT-
GTTCGACCCCAAGAACATGATGGCTGCTTCTGACTTCCGCAACGGTCGTTACCTGACATGCTCTGCCATCTT
GTAAGTGACC-GA----TATATTACTT---------TTATA--------------------------TCTACGTAT-
CGAGTAAGTACTAATAACG--ATATATTATAGCCGTGGCAAGGTCTCCATGAAGGAAG--------------------------
----

>H_fuscoides_MUCL_52670

CAGACCAGCAAGAAAACAGGAAAAAACCCCTGA-----ACGCG-TCCA--------------AAAT--------------CTC---
CAAC-ATCCCCTGTGAT------TCC----T-TCTGCCCCTC----CACGCAA--------CACA----------------------------------
-------------------AACAATTCAACGACAA-------------------CCGCATTT------TGCAA----------------------------------
-------------CWG--T-TCAA---------------GAT------A--A-----------------------TATGGA---------------
AATAGTAAAGAGCTAA-CCGTG---TC--TTCTTCAT------------
CGTAATAGGTCCACCTTCAGACCGGCCAATGCGTAAGATTGCTAT----------------------CCCC----
GACGAAACAAG-----------------GCGATAG---------------AAAG-AGGGCGG-----GGCTCACACGA---A-TAT-----
CA-TAGGGTAACCAAATCGGTGCTGCTTTCT--
-GGCAAACCATCTCTGGCGAGCACGGTCTCGACAGCAATGGCGTGTACGTCTTCT-------------------
CTTTATTGGTC-------------------AATTCTTGAAGAG-------ACGAA--------------------------CATC-AAAT-------
TGAC-----------AA--TCTG-

AAATAGGTACAACGGAACTTCGGAGCTTCAGCTGGAGCGCATGAGCGTCTACTTCAACGAGGTACGCTAT-
CTTGA-----------------A----------A----ACA-----CA-CGATGAAAACACTGAAAACACACGAGATGC-GCAA--------
AGACGGT-TT-A-----CTAAT--CAC-CC-CAA---CATGC-
ACAGGCGTCTGGTAACAAGTACGTGCCTCGTGCCGTCCTCGTCGATCTCGAGCCCGGTACCATGGATGCTG
TCCGTGCCGGTCCCTTCGGTCAACTCTTCCGCCCCGACAACTTCGTCTTCGGTCAATCCGGTGCCGGAAACA
ACTGGGCCAAGGGTCATTACACCGAGGGTGC-TGAGCTGGTGG-
ACCAGGTTCTCGACGTCGTTCGTCGTGAGGCCGAGGGTTGCGATTGCCTTCAGGGCTTCCAGATCACCCACT
CGCTCGGTGGTGGTACTGGTGCCGGTATGGGTACCTTGTTGATCTCCAAGATTCGCGAGGAGTTCCCCGAC
CGCATGATGGCCACCTTCTCCGTCGTACCCTCCCCCAAGGTCTCCGACACCGTCGTCGAGCCTTACAATGCTA
CCCTCTCGGTCCATCAGCTGGTCGAGAACTCGGACGAGACCTTCTGCATCGACAACGAGGCTCTGTACGAC
ATCTGCATGCGTACCCTCAAGCTATCCAACCCCTCGTACGGTGACCTGAACCACCTGGTCTCCGCCGTCATGT
CCGGTGTTACCACCTGCTTGCGATTCCCCGGTCAGCTGAACTCGGACCTGCGCAAGCTGGCCGTCAACATG
GTTCCTTTCCCGCGTCTGCACTTCTTCATGGTCGGCTTCGCGCCCCTGACCAGCCGTGGTGCTTACACCTTCC
GCGCCGTCACCGTTCCCGAGTTGAYTCA-GCAGAT-
GTTCGACCCCAAGAACATGATGGCTGCTTCTGACTTCCGCAACGGTCGATACCTGACATGCTCTGCCATCTT
GTAAGTCACC-TA----TCTATTTCGT---------TAATA--------------------------TCTACGCAT-
CGAGCAAGTACTAATAACG--
ATATATAATAGCCGTGGCAAGGTCTCCATGAAGGAAGTTGAGGACCAGATGCGCAACGTCAGAACAAG----
----

>H_porphyreum_CBS_119022

-------------------------GAGGGA---
CCATTGGATCAGAGGAAAAGAGGGAAACCCCCTGA-----ACGCG-TCCA--------------AAAAT-------------CTC--
-CAAC--GCCCCCCTGAT------GTTTGATT-TCTGCCCCTC----CACGCAC---------ACA------------------------------
----------------------AAC-ACCCAACGACA--------------------TCGCATCG------TGCAA----TT-----CCC---TACA---
TCGC---------------AAA-CTA--C-TCGA---------------GAC------G--G------------------------TG-------------------
AAATTTTGAAAGCTAA-CCGTG---TC---TCTTCAT------------
CTCGATAGGTTCACCTTCAGACCGGCCAATGCGTAAGAATTCTTATCC--------------------------
CGACGACCAAC-----------------GCGATA----------------GAAG-ATTGCGG-----GGCTCACACGA---A-TGT-----
CA-TAGGGTAACCAAATCGGTGCTGCTTTCT---
-GGCAAACCATCTCTGGCGAGCACGGCCTCGACAGCAATGGCGTGTATGTGTTGTG------------------
TTCCATTGGTC-------------------AATTCTTGGGAG--------ACGAA---------------------------TATA-AAAT-------
TGACA--------GAACC-GTTT-
GAATAGGTACAACGGAACCTCCGAGCTCCAGCTGGAGCGCATGAGCGTCTACTTCAACGAGGTACGCTAT-
CTTGA-----------------A----------A----ACA-----AA-------------T----------AAATGC-GCAA--------AGACCCG-TT-
A-----CTAAT--CAC-CC-CAA---CATGC-
ACAGGCGTCTGGTAACAAGTACGTGCCTCGTGCCGTCCTCGTCGATCTCGAGCCCGGTACCATGGACGCTG
TCCGTGCTGGCCCCTTCGGTCAGCTCTTCCGACCCGACAACTTCGTCTTCGGCCAATCCGGTGCCGGAAACA
ACTGGGCGAAGGGTCACTACACCGAGGGTGC-TGAGCTGGTTG-
ACCAGGTCCTTGATGTCGTCCGTCGTGAGGCTGAGGGCTGCGACTGCCTTCAGGGCTTCCAGATCACCCACT
CTCTCGGTGGTGGTACTGGTGCCGGTATGGGTACCCTGCTGATCTCCAAGATCCGCGAGGAGTTCCCCGAC
CGCATGATGGCCACCTTCTCCGTCGTCCCCTCCCCCAAGGTCTCCGACACCGTCGTCGAGCCTTACAACGCC
ACCCTCTCCGTCCACCAGCTGGTCGAGAACTCTGACGAGACCTTCTGCATCGACAACGAGGCCCTGTACGAC
ATCTGCATGCGTACCCTGAAGCTATCCAACCCCTCGTACGGTGACCTGAACCACCTGGTCTCCGCTGTCATG

TCTGGTGTTACCACCTGCTTGCGATTCCCCGGTCAGCTGAACTCGGACTTGCGCAAGCTGGCCGTCAACATG
GTTCCCTTCCCGCGTCTGCACTTCTTCATGGTCGGCTTCGCTCCCCTGACCAGCCGTGGTGCTCACTCCTTCC
GCGCCGTCACCGTTCCCGAGTTGACTCA-GCAGAT-
GTTCGACCCCAAGAACATGATGGCCGCCTCTGACTTCCGCAACGGTCGTTACCTGACGTGCTCTGCCATCTT
GTAAGTGCCT-AT----CTCGATCAA----------TGTTT-------------------------GATGCGTAC-CGA---
TCTGCTAACGCC-----
GATATTCAGCCGCGGCAAGGTCTCCATGAAGGAAGTTGAGGACCAGATGCGCAACGTCAGAACA-----------
----

>H_vogesiacum_CBS_115273

--CC---CAAC--GCCCCT-TGAT-------------T-
CCTGCCCCTC----CACGCAC---------ATA---AAC--ACCAAACAAAC-
-----------------ATCAGATGT------CTAAA----TT-----CTC---TGCA---TCGC---------------GAT-CCA-----CA---------
---CA--CAT------A--------------------------CGATATT---------------GGGAAATTGAAGCTAA-TCATG---TC---
TCTTCAT------------CTGAATAGGTTCACCTTCAGACCGGCCAATGCGTAAGAACCACAACC--------------------
-------CGACGATCAGC-----------------ACGATG----------------GAGG-ATGGCTT-----AACTCACACGA---A-TCT--
---TA-TAGGGCAACCAAATTGGTGCTGCTTTCT---
----GGCAAACCATCTCTGGCGAGCACGGTCTCGACAGCAATGGCGTGTACGTGTCTTG----TCTTGTC----
TTGTCTTATTGGTC-------------------AATTCCGCAGAGA-------GGCAT--------------------------CTC--GAAC-----
--TGAC-----------ACC-CTAT-
CAACAGGTACAACGGAACCTCTGAGCTCCAGCTCGAGCGCATGAGCGTCTACTTCAACGAGGTACGATAT-
CTTGA-----------------A----------A----AAT-----CA---------------------ACAAGATGC-GC-A--------AGACCCG-TT-
A-----CTAAT--CA--CC-TAA---CATGC-
ACAGGCATCTGGTAACAAGTACGTGCCCCGTGCCGTCCTCGTCGATCTCGAGCCCGGTACCATGGACGCCG
TCCGTGCTGGTCCCTTCGGCCAGCTCTTCCGACCCGACAACTTCGTCTTCGGCCAGTCCGGTGCCGGAAACA
ACTGGGCCAAGGGTCACTACACCGAGGGTGC-TGAGCTGGTCG-
ACCAGGTTCTCGACGTCGTCCGTCGCGAGGCCGAGGGCTGCGACTGCCTCCAGGGTTTCCAGATCACCCAC
TCTCTCGGCGGTGGCACTGGTGCCGGTATGGGCACCCTGCTGATCTCCAAGATCCGCGAGGAGTTCCCCGA
CCGCATGATGGCCACCTTCTCCGTCGTCCCCTCCCCCAAGGTCTCCGACACCGTCGTCGAGCCTTACAACGCC
ACCCTCTCCGTCCACCAGCTGGTCGAGAACTCGGACGAGACCTTCTGCATCGACAACGAGGCTCTCTACGAC
ATCTGCATGCGCACTCTCAAGCTGTCCAACCCCTCGTACGGCGACCTGAACCACCTGGTCTCCGCCGTCATG
TCCGGCGTCACCACCTGCCTGCGATTCCCCGGTCAGCTGAACTCTGACCTGCGCAAGCTCGCCGTCAACATG
GTGCCCTTCCCGCGTCTGCACTTCTTCATGGTCGGCTTCGCGCCCCTGACCAGCCGCGGTGCCTACACCTTCC
GCGCCGTCACCGTTCCCGAGTTGACTCA-GCAGAT-
GTTCGACCCCAAGAACATGATGGCCGCCTCTGACTTCCGCAACGGTCGTTACCTGACGTGCTCGGCCATCTT
GTAAGACACC-CA----CCCTACT------------CCGTA-------------------------TGATGCCGC-TCA---
TCTGCTAACCTG----ACTCTTCCAGCCG---

>H_carneum_MUCL_54177

GAGAAGGAGAGGAACCCCTGA-----ACGCG-TCCA---------AAAGAAAAAAGAAGAAGAAAACACCC---
TAAT-TTCCCCC-TAAT------TTCATGCTCTTTGCCCCTCCCTCTATGTAC---------AAA--

CACCATAAACACCATAAACACCATAAACACCACAATCACCATCACCACAACAAC--TACCACAACCACCAG----
---AAACACGACAGGATAT------ATCAA----CT-----CCT---TGCA---TGGC---------------GGA-ATG-----------------
--C---TGC------A--G-----------------------AATTCC---------------CGAAAATGAAAGCTGA-CCGTA---TC---TC----
-------------ATCAATAGGTTCACCTCCAGACCGGCCAATGTGTAAGAATTACCTA---CACC-------CAT-
AACCATC----TACCTACGAAC-----------------GCGATAGG------------AAGAAT-ATGGCGG-----
GGCTCACATGA---G-TTT-----TATTAGGGTAACCAAATTGGTGCCGCTTTCT---------------------------------------
----

GGCAAACCATTTCTGGCGAGCACGGTCTCGACAGCAATGGCGTGTACGTATTTT---------------------
TGTTTATC-------------------AATTCCTTACGAC-------GAAGGTAT---------------------GGTTAG--AAAC-------
TAAT-----------GAT-CTGT-
CAACAGCTACAACGGAACCTCCGAGCTCCAACTGGAGCGCATGAGCGTCTACTTCAACGAGGTATGCCTT-
GATCA-----------------A----------G----AGC-----AG-GAAT--------------------ATTTGC-GCAAGGACC--
CAGACTCC-TT-A-----CTGAT--TAT-TA-TGA---CATGC-
GCAGGCTTCCGGCAACAAGTACGTGCCTCGTGCCGTCTTGGTCGATCTCGAGCCCGGTACCATGGACGCTG
TTCGTGCTGGTCCTTTTGGTCAGCTCTTCCGACCCGACAACTTCGTTTTCGGTCAGTCCGGTGCCGGCAACAA
CTGGGCCAAGGGTCATTACACGGAGGGTGC-CGAGCTGGTCG-
ACGCCGTCTTGGATGTCGTTCGTCGCGAGGCTGAGGGATGCGACTGCCTTCAGGGCTTCCAGATTACCCATT
CGCTCGGTGGTGGTACCGGTGCCGGTATGGGTACCTTGCTGATCTCCAAGATTCGCGAGGAGTTCCCCGAC
CGCATGATGGCCACCTTCTCCGTCGTTCCCTCTCCCAAGGTCTCCGACACCGTCGTCGAGCCTTACAACGCCA
CCCTCTCCGTCCATCAGCTGGTCGAGAACTCGGACGAGACTTTCTGCATTGACAACGAGGCTCTATACGACA
TCTGCATGCGTACCCTGAAGCTATCCAACCCTTCCTATGGTGACCTGAACCACTTGGTCTCGGCCGTCATGTC
GGGTGTCACCACCTGTCTGCGATTCCCTGGCCAGCTGAACTCTGACCTCCGCAAGCTGGCCGTCAACATGGT
GCCCTTCCCTCGTCTGCACTTCTTCATGGTTGGCTTCGCTCCTCTGACCAGCCGCGGTGCTTACTCTTTCCGTG
CCGTCACCGTTCCCGAGTTGACGCA-GCAGAT-
GTTTGACCCGAAGAACATGATGGCCGCCTCGGACTTCCGCAACGGTCGCTACCTGACGTGCTCTGCCATCTT
GTAAGATACC-TAAAACCCCCAAG------------CCGTC--------------------------CACGAGTGC-TTG---
TTTGCTGACCCT----AATACTCTAGCCGTGGCAAGATCTCGATGAAGGAGGTCGAGGACCAGA----------------
----

>H_cercidicola_CBS_119009

--TGA--------------------------------CCCAGCCGAGT-----------
-------------ACGAGAAGAACCCCTGA-----ACGCG-TCCC--------------GAAAA-------------CCC---CAAC--
TTCCCC-TGATTTCCTGTTCCTGTT-CCTGCCCCTC----CACCCAC---------AAA--------------------------------------
--------------CAC--CACAGACAACA--------------GAATCCGAGAT--------ATCAA----AT-----CCC---TACA---
TCGC---------------AAA-CCA-------------------C---CAC------A--G-----------------------GATTCT---------------
GGAAAACGAAAGCTAA-CCATA---TA---TCTTTAT------------
CGGAATAGGTTCACCTTCAGACCGGCCAATGCGTAAGAACTACCTA---CACC-------TAC-AACCAGC-----
GAT-ACCAAC------------------GCGGAT--------------AGAAAG-ATGGTGG-----GGCTCACATGA---A-TTT-----
TA-CAGGGTAACCAAATTGGTGCTGCTTTCT---
-GGCAAACCATCTCTGGCGAGCACGGTCTCGACAGCAATGGCGTGTACGTATTC----------------------
CATTGGTC-------------------AATTCCCATCGAC-------GAGAC--------------------------TGA--AACT-------AATA--
---------AAT-TTGT-
AAATAGCTACAACGGAACCTCTGAGCTCCAGTTGGAGCGCATGAGCGTCTACTTCAACGAGGTATGCCAC-
AATCA-----------------G----------G----AA------AG-AAAT--------------------CCGTGC-GC-G--------AGACATG-

GT-A-----CTAAT--TAC-AC-CGA---AATTT-
GCAGGCTTCCGGCAACAAGTATGTACCCCGTGCCGTCCTCGTCGATCTCGAGCCCGGTACCATGGATGCCG
TCCGTGCTGGTCCCTTCGGTCAGCTCTTCCGACCCGACAACTTCGTTTTCGGTCAGTCTGGTGCTGGCAACAA
CTGGGCCAAGGGTCACTACACTGAGGGTGC-CGAGCTCGTTG-
ACCAGGTTTTGGATGTCGTTCGTCGTGAGGCTGAGGGCTGCGACTGCCTTCAGGGTTTCCAGATTACCCACT
CGCTCGGTGGTGGTACCGGTGCCGGTATGGGTACCTTGTTGATCTCCAAGATCCGCGAGGAGTTCCCTGAC
CGCATGATGGCTACTTTCTCCGTCGTTCCCTCTCCTAAGGTCTCTGATACCGTTGTCGAGCCTTACAACGCCA
CCCTCTCCGTCCACCAGCTGGTCGAGAACTCGGACGAGACCTTCTGCATTGATAACGAGGCCCTGTACGACA
TCTGCATGCGTACCCTGAAGCTATCCAACCCCTCGTACGGTGACCTGAACCACCTGGTTTCCGCCGTCATGTC
TGGTGTCACCACCTGCTTGCGATTCCCTGGCCAGCTGAACTCTGACCTCCGAAAGTTGGCTGTCAACATGGT
GCCCTTCCCTCGTCTGCACTTCTTCATGGTCGGCTTCGCTCCCTTGACCAGCCGTGGTGCTTACTCTTTCCGTG
CTGTCACCGTTCCCGAGTTGACTCA-GCAAAT-
GTTCGACCCCAAGAACATGATGGCTGCCTCTGACTTCCGTAACGGTCGTTACCTGACGTGCTCTGCCATCTT
GTAAGATGCC-TG----TTTCAAG------------TTGTC--------------------------GATTAGTGC-TTA---
TTTGCTGACCTT------
CACTCTAGCCGTGGCAAGATCTCCATGAAGGAAGTCGAGGACCAGATGCGTAACGTCCAGAACAAG--------
----

>H_petriniae_CBS_114746

----------------GTAAGT-------------------------------------CTTGGTCTTTTATGTTTA-----------
CTGCTGACCCGAGCCGAGT-----------------------ACGAGAAGAACCCCTGA-----ACGCG-TCCC-------------
GAAAA-------------CCC---CAGC--TTCCCC-TGAT------TTCCTGTT-CCTGCCCCTC----CACGCAC---------AAA---
---CAC--CACAAACACAG---------------AACCCGAGAT--------ATCAA--
--AT-----CCC---TACG---TCGC---------------AAA-CTA-------------------C---CAC------A--G------------------------
GATT-----------------GGAAAATGAAAGCTAA-CCATA---TA---TCTTTAT------------
CGGAATAGGTTCACCTTCAGACCGGCCAATGTGTAAGAACTATCTA---CACC-------TAC-AACCAGC--------
GATCAAC-----------------GCGAAT--------------AGCAAG-ATGGTGG-----GGCTCACATGA---A-TTT-----TA-
TAGGGTAACCAAATTGGTGCTGCTTTCT---
GGCAGACCATCTCTGGCGAGCACGGCCTCGACAGCAATGGCGTGTACGTATTC-----------------------
CATTGGTC-------------------AATTCCTCCGACG-------AGA-----------------------------CCA--AAAC-------TAAT----
-------AAT-TTGT-
CAACAGCTACAACGGAACCTCTGAGCTCCAGCTGGAGCGCATGAGCGTCTACTTCAACGAGGTATGTCAC-
AATCA-----------------A----------G----AGC-----AG-GAAC-------------------CCGTGC-GC-G--------AGACCTG-
TT-A-----CTAAC------CC-CGA---CTTGC-
GCAGGCTTCCGGCAACAAGTATGTACCTCGTGCCGTCCTCGTCGATCTCGAGCCCGGTACCATGGATGCCGT
CCGTGCTGGTCCCTTCGGTCAGCTCTTCCGACCCGACAACTTCGTTTTCGGTCAGTCCGGTGCTGGCAACAA
CTGGGCCAAGGGTCATTACACTGAGGGTGC-CGAGCTCGTTG-
ACCAGGTTTTGGATGTCGTTCGTCGTGAGGCTGAGGGCTGCGACTGCCTTCAGGGTTTCCAGATTACCCACT
CGCTTGGTGGTGGTACCGGTGCCGGTATGGGTACCTTGTTGATCTCTAAGATCCGCGAGGAGTTCCCCGAC
CGCATGATGGCTACTTTCTCCGTCGTTCCCTCCCCCAAGGTTTCTGACACCGTTGTCGAGCCTTACAACGCCA
CCCTCTCTGTCCACCAGCTGGTCGAGAACTCGGACGAGACCTTCTGCATTGATAACGAGGCTCTGTACGACA
TCTGCATGCGTACCCTGAAGCTATCCAACCCCTCGTACGGTGACCTGAACCACCTGGTCTCCGCCGTCATGT
CCGGTGTTACCACCTGCTTGCGATTCCCTGGCCAGCTGAACTCTGACCTCCGAAAGTTGGCTGTCAACATGG
TGCCCTTCCCTCGTCTGCACTTCTTCATGGTCGGCTTCGCTCCCCTGACCAGCCGCGGTGCTTACTCCTTCCGT

GCTGTCACCGTTCCCGAGTTGACTCA-GCAGAT-
GTTCGACCCCAAGAACATGATGGCCGCCTCCGACTTCCGCAACGGTCGCTACCTGACGTGCTCTGCCATCTT
GTAAGATACC-TG----TTCCGAT------------TTGTC-------------------------GATTAGTGC-TT----TTTGCTAAC-----
----

>H_isabellinum_MUCL_53308

-----GGAGGACCCCTGA-----ACGCG-TCCC--------------ATAAA-------------CCC---CAAC-TTTCCCC-TGAT------
TTCATCCT-CCTGCCCCTC----CACTCAC---------AAA--CAC--
CACAAATCCAA--------------AACGAAGATAT--------TCCAG----TC-----TCC---TACA---TCGC---------------AGA-
CTT-------------------C---AGC------A--G------------------------AGTTTT---------------GAGAAACGAAAGCTAA-
CCTTA---TC---TCGTC--------------TCAAATAGGTTCACCTCCAGACCGGCCAATGCGTAAGAATTGCCTC---
CACCTCCAC-ATAC-AACCATC-----GAATATCGAC-----------------GCGATAGGA-----------
AAAAATAATGGCGG-----GGCTC--ACGA---A-TAT-----TG-TAGGGTAACCAAATCGGTGCTGCTTTCT---------
----
GGCAAACCATCTCTGGCGAGCACGGTCTCGACAGCAATGGCGTGTACGTATTT-----------------------
CATTGGTC-------------------AATTCTTGGGATG-------GGAAT---------------------------TGG--GAAT-------TAAT-
----------GGG-TTAT-
CTACAGCTACAACGGAACCTCTGAGCTCCAGCTGGAGCGCATGAGCGTCTACTTCAACGAGGTACGCCGA-
AATCA-----------------A----------C----AGC-----AA-AAAT--------------------ACATGC-GC-A--------AGGACCG-
TT-A-----CTAAT-------T-CAA---CATGC-
GCAGGCTTCCGGTAACAAGTACGTTCCCCGTGCCGTCCTCGTCGATCTCGAGCCCGGTACCATGGACGCCGT
CCGTGCTGGTCCCTTCGGTCAGCTCTTCCGACCTGACAACTTCGTCTTTGGTCAATCCGGTGCCGGCAACAA
CTGGGCCAAGGGTCACTACACTGAGGGTGC-TGAGCTTGTCG-
ACAACGTTTTGGATGTCGTTCGTCGTGAGGCTGAGGGCTGCGATTGCCTTCAGGGTTTCCAGATTACCCACT
CGCTCGGTGGTGGTACCGGTGCCGGTATGGGTACCTTGTTGATCTCCAAGATCCGCGAGGAGTTCCCCGAC
CGAATGATGGCTACTTTCTCCGTCGTTCCCTCCCCCAAGGTCTCCGACACCGTCGTCGAGCCCTACAACGCTA
CTCTTTCCGTCCACCAGCTGGTCGAGAACTCGGACGAGACCTTCTGCATTGATAACGAGGCTCTGTACGACA
TCTGCATGCGTACCCTGAAGCTATCCAACCCCTCGTACGGTGACCTGAACCACCTGGTCTCCGCCGTCATGT
CGGGTGTTACCACTTGCTTGCGTTTCCCTGGTCAGCTGAACTCTGATCTCCGCAAGTTGGCTGTCAACATGG
TGCCATTCCCTCGTCTGCACTTCTTCATGGTCGGCTTCGCTCCCCTGACCAGCCGCGGTGCTTACTCTTTCCGT
GCCGTCACCGTCCCCGAGTTGACGCA-ACAGAT-
GTTCGACCCCAAGAACATGATGGCCGCTTCCGACTTCCGCAACGGTCGCTACCTGACATGTTCTGCCATCTT
GTAAGATA---TA----CCCTAAA------------TCTCC-------------------------AATAAGTGC-TTA---
TTTGCTAACCCT----GAATTTCTAGCCGTGGCAAGATCTCCATGAAGGAGGTTGAGGACCAGATGCGCAAC--
----

>H_musceum_MUCL_53765

-------GGAACCCCTGA-----ACGCG-TCCC--------------GAAAA-------------CCC---CAAC--TTCCCC-TGAT------
TTCATGCT-CCTACCCCTC----CAAGCAC---------AAA--CAC--
CACAAACCCAA---------------AACGAGATAT--------CTCAG----TG-----CCC---AGCA---TCGC--------------AGA-
CTA-------------------C---ATC------G--G-----------------------ATTATT---------------TGAAGCTAAAAGCTAA-
CCTTA---TT---TCTTCGT------------CTCAATAGGTTCACCTCCAGACCGGCCAATGCGTAAGAACCACCTA---

TACC-------TAC-GACCATC-----GATCGTCAAC-----------------GCGATAGGG-----------AAAAAT-ATAGCGG----
-GGCTC--ACGA---A-TTT-----TA-TAGGGTAACCAAATTGGTGCCGCTTTCT--

GGCAAACCATCTCTGGCGAGCACGGTCTCGACAGCAATGGCGTGTATGTGCTT----------------------
GATTGGAC-------------------AATTCTTCATATG-------GGGAT--------------------------CGA--AAAT-------TAAT--
---------AAT-TTCT-
GAATAGCTACAACGGAACCTCTGAGCTTCAGTTGGAGCGCATGAGCGTCTACTTCAACGAGGTACGTCGG-
AATTA-----------------A----------G----ATA-----AA-AGCT--------------------ACATGC-GC-A--------AGAATTG-TC-
A-----CTAAT--TAT----TGA---TATGC-
GCAGGCTTCCGGCAACAAGTATGTCCCCCGTGCCGTCCTCGTCGATCTCGAGCCCGGCACCATGGATGCCG
TCCGTGCTGGTCCTTTCGGTCAGCTCTTCCGACCCGACAACTTCGTCTTTGGTCAATCCGGTGCTGGCAACAA
CTGGGCCAAGGGTCACTACACTGAGGGTGC-TGAGCTTGTTG-
ACAACGTTTTGGATGTCGTTCGTCGTGAGGCTGAGGGCTGCGACTGCCTTCAGGGTTTCCAGATTACCCACT
CGCTCGGTGGTGGTACCGGTGCCGGTATGGGTACCTTGTTGATCTCCAAGATCCGCGAGGAGTTCCCCGAC
CGAATGATGGCCACCTTCTCCGTCGTTCCCTCCCCCAAGGTCTCCGACACCGTCGTCGAGCCCTACAACGCC
ACCCTGTCCGTTCACCAGCTGGTCGAGAACTCGGACGAGACCTTCTGCATTGATAACGAGGCTCTGTACGAT
ATCTGCATGCGTACCCTTAAGCTATCCAACCCCTCGTATGGTGACCTGAACCACCTGGTCTCCGCCGTTATGT
CGGGTGTCACCACTTGCTTGCGATTCCCTGGTCAGCTAAACTCTGACCTGCGCAAGTTGGCCGTCAACATGG
TGCCCTTCCCTCGTCTGCACTTCTTCATGGTTGGCTTCGCTCCTCTGACCAGCCGTGGTGCTTACTCTTTCCGT
GCTGTCACCGTTCCCGAGTTGACACA-GCAGAT-
GTTCGACCCCAAGAACATGATGGCTGCCTCTGACTTCCGTAACGGTCGCTACCTGACGTGCTCTGCCATCTT
GTAAGAGACC-TG----CTTTAAA------------GTTTT-------------------------GATAAGTGC-TTA---
TTTGCTAACTCC----TAATCTTTAGCCGTGGCAAGATCTCCA---

>H_perforatum_CBS_115281

--GAGCCGAGT---------------
----ACGAAGGAAGAGGAACCCCTGA-----ACGCG-TCCC-------------GAAAA------------CCC---CAAT--
TTCCCC-TGAT------TTCATGCT-CCTACCCCTC----CACGCAC---------AAA--
----------CAC--CACAAACACAA-------------AAACGAGATAT--------CTCCA----TT-----CCC---TGCA---TCGC----
-----------AGA-CTT-------------------C---AGC------A--G-----------------------AGTTTT-------------
GGGGAAATGAAAGCTAA-CCTTA---TA---TCTTCGT------------
CTCAATAGGTTCACCTCCAGACCGGCCAATGCGTAAGAATTACCTA---CCCC-------TAC-AACTACC-----
GACTATCAAC-----------------GCGATAGG------------GAAAACGATAGCGG-----GGCTCACGAAT---A-TTA-----
TA-TAGGGTAACCAAATTGGTGCTGCTTTCT---
-GGCAAACCATCTCTGGCGAGCACGGTCTCGACAGCAATGGCGTGTACGTATTT----------------------
CATTGGTC-------------------AATCCTTCGGATG-------GGAGT--------------------------TGG--ATAT-------TAAT--
---------GGT-TTAT-
CAACAGCTACAACGGAACCTCTGAGCTACAGCTGGAGCGCATGAGCGTCTACTTCAACGAGGTACGTCGA-
AATCA-----------------A----------G----AAC-----AA-GAAT-------------------ACATGC-GC-A--------AAGCCCG-
TT-A-----CTAAT--TAT-CT-TGA---CTTGT-
GCAGGCTTCCGGCAACAAGTATGTTCCCCGTGCCGTCCTCGTCGATCTCGAGCCCGGTACCATGGACGCCGT
CCGTGCTGGTCCCTTCGGTCAACTCTTCCGACCCGACAACTTCGTCTTTGGTCAATCCGGTGCCGGCAACAA
CTGGGCCAAGGGTCACTACACTGAGGGTGC-TGAGCTGGTCG-

ACCAGGTTTTGGATGTTGTTCGTCGTGAGGCTGAGGGCTGTGACTGCCTCCAGGGTTTCCAGATTACCCACT
CGCTCGGTGGTGGTACCGGTGCCGGTATGGGTACCTTGTTGATCTCCAAGATCCGCGAGGAGTTCCCCGAC
CGAATGATGGCCACCTTCTCCGTCGTTCCCTCCCCCAAGGTCTCTGACACCGTTGTCGAGCCTTACAACGCTA
CCCTTTCCGTCCACCAGCTGGTCGAGAACTCGGACGAGACCTTCTGCATTGATAACGAGGCTCTATACGATA
TCTGCATGCGTACCCTGAAGCTATCCAACCCCTCGTACGGTGACCTGAACCACCTGGTCTCTGCTGTCATGTC
GGGTGTCACCACTTGCCTGCGATTCCCCGGTCAGCTGAACTCTGACCTCCGCAAGTTGGCTGTCAACATGGT
GCCCTTCCCCCGTCTGCACTTCTTCATGGTCGGCTTCGCTCCTCTGACCAGCCGCGGCGCTTACTCCTTCCGT
GCTGTCACCGTTCCCGAGTTGACGCA-GCAGAT-
GTTCGACCCCAAGAACATGATGGCTGCCTCTGACTTCCGCAACGGTCGTTACCTGACATGCTCTGCCATCTT
GTAAGATACTATG----CTCCAAA------------TCGTC-------------------------AATAAATGT-TTA---
TTTGCTAACCCT----
TAATCTCTAGCCGTGGCAAGATCTCCATGAAGGAGGTTGAGGACCAGATGCGCAACGTCCA-----------------
----

>H_sporistriatatunicum_UCH9542

----------GAGATTGTAAGTCTT----------------------------------ACTACTTTTTAATGTTTACTG--------
CTGACCCTCCGAGCCGAGT-------------------AACGAAGGAGAGGAACCCCTGA-----ACGCG-TCCG------------
-GAAAA-------------CCC---CAAC--TTCCCC-TGAT------TTCATGCT-CCTACCCCTC----CACCCAC---------AAA--
---CAC--CAAAAAC----------------------AGATAT--------CTCCA----TC-
----CCC---TGCA---ACGC---------------AGA-CAT------------------C---AGC------A--G-----------------------
AGTTCC--------------AGAAAATTGAAAGCTAA-CCTTA---TC---TCTTCGT------------
CTCAATAGGTTCACCTCCAGACCGGCCAATGCGTAAGAACTATACC---CACC-------TAT-AACCATC-----
GACCATCAAT-----------------GCGATAGG------------GAAAATAATGGCGG-----GGCTC--ACGA---A-TTT-----
TA-TAGGGTAACCAAATTGGTGCTGCTTTCT--
-GGCAAACCATCTCTGGCGAGCACGGTCTCGACAGCAATGGCGTGTACGTATTT----------------------
CATTGGTC-------------------AATTGTTCGAATT-------GGAAT---------------------------TGG--AAAT-------TAAT--
---------GAT-TTAT-
CATTAGATACAACGGAACCTCAGAGCTCCAGCTTGAGCGCATGAGCGTCTACTTCAACGAGGTAGGTCAA-
AATCA-----------------A----------C----AAG-----AA-GAAT--------------------ATTTGC-GC-A--------GGGTCAG-
TC-A-----CTAAT-------T-CAA---CATGC-
GCAGGCTTCCGGCAACAAGTATGTTCCCCGTGCCGTCCTCGTCGATCTTGAGCCCGGTACCATGGACGCCGT
CCGTGCTGGTCCCTTCGGTCAGCTCTTCCGACCCGACAACTTCGTCTTTGGTCAATCCGGTGCTGGCAACAA
CTGGGCCAAGGGTCACTACACTGAGGGTGC-TGAGCTTGTTG-
ACCAGGTTTTGGATGTCGTTCGTCGTGAGGCTGAGGGCTGCGACTGCCTTCAGGGTTTCCAGATTACCCACT
CGCTTGGTGGTGGTACCGGTGCCGGTATGGGTACCTTGTTGATCTCCAAGATCCGCGAGGAGTTCCCCGAC
CGAATGATGGCCACTTTCTCCGTCGTTCCCTCCCCCAAGGTCTCCGACACCGTTGTCGAGCCTTACAACGCTA
CCCTCTCCGTCCACCAGCTGGTCGAGAACTCGGACGAGACCTTCTGCATTGATAACGAGGCTCTGTACGATA
TCTGCATGCGTACCCTGAAGCTATCCAACCCCTCGTACGGTGACCTGAACCACCTGGTCTCCGCCGTCATGT
CGGGTGTTACCACTTGCTTGCGATTCCCTGGTCAGCTGAACTCTGACCTCCGCAAGTTGGCTGTCAACATGG
TGCCCTTCCCTCGTCTGCACTTCTTCATGGTCGGCTTCGCTCCCCTGACCAGCCGTGGCGCTTACTCCTTCCGT
GCTGTCACCGTTCCCGAGTTGACGCA-ACAGAT-
GTTCGACCCCAAGAACATGATGGCTGCCTCCGATTTCCGCAACGGTCGCTACCTGACGTGCTCTGCCATCTT
GTAAGATACC-CA----CCTCACG------------TTATT-------------------------GATAGGCCC-TCA---
TTTGCTAACGTT------

TAATCCAGCCGTGGCAAGATCTCCATGAAGGAAGTCGAGGACCAGATGCGCAACGTCCAGAACAAGAACT
CGTCGTACTTCGTTGAGTGGATT----------------

>H_gibriacense_MUCL_52698

---CCGAGTA-------------
CGAGAGTACGAAAGAAACAACCCCTGA-----ACGCG-TCCC-------------GAAAA------------CCC---CAAC--
TTCCCC-TGAT------TTCATGCT-CCTACCCCTC----CACGCAC---------AAA---
----------CGC--CACAAACACAA---------------AACGAGATAT--------CTCAA----TC-----CCC---TACA---TCGC-----
----------AGG-CCA------------------C---AAC------A--G-----------------------AGTTCT---------------
AGAAACTGAAAGCTAA-CCTTA---TC---TCTTCTTC----------
TCAATATAGGTTCACCTCCAGACCGGCCAATGCGTAAGAACTACCTT---TACC-----------TACCACC-----
GACCATCAGC-----------------GCGATAGG------------AAAAAT-ATGGCGG-----GGCTCACACGA---A-TAT-----
TA-TAGGGTAACCAAATTGGTGCTGCTTTCT---
-GGCAAACCATCTCTGGCGAGCACGGTCTCGACAGCAATGGCGTGTATGTATCT----------------------
CATTGGTC------------------AATTCATCA-ACG-------GGAAT--------------------------TGG--AAAC-------TAAT--
---------AGT-TGGT-
CAACAGCTACAACGGAACCTCGGAGCTCCAGCTCGAGCGCATGAGCGTCTACTTCAACGAGGTATGTCG--
AATCG-----------------A----------G---AGC-----AA-GAAT--------------------ACATGC-GC-A--------
AGACTCGCTT-G-----CTAAT--AAC-TT-CCA---CGTGC-
GCAGGCTTCCGGCAACAAGTATGTTCCCCGTGCCGTCCTCGTCGATCTCGAGCCCGGTACCATGGACGCTGT
CCGCGCTGGTCCTTTCGGTCAGCTCTTCCGACCTGACAACTTCGTCTTCGGTCAATCCGGTGCCGGCAACAA
CTGGGCCAAGGGTCACTACACCGAGGGTGC-TGAGCTTGTTG-
ACCAAGTTTTGGATGTCGTTCGTCGTGAGGCTGAGGGCTGCGACTGCCTTCAGGGTTTCCAGATTACCCACT
CGCTCGGTGGTGGTACCGGTGCCGGTATGGGTACCTTGTTGATCTCCAAGATCCGCGAGGAGTTCCCCGAC
CGCATGATGGCCACCTTCTCTGTCGTTCCCTCCCCCAAGGTCTCTGATACCGTTGTCGAGCCCTACAACGCCA
CCCTCTCCGTCCACCAGCTGGTCGAGAACTCGGACGAGACCTTCTGCATTGATAACGAGGCTCTCTACGATA
TCTGCATGCGTACCCTAAAGCTATCCAACCCCTCGTACGGCGACCTGAACCACCTGGTCTCCGCCGTCATGT
CGGGTGTTACCACTTGCTTGCGATTCCCCGGTCAGCTAAACTCTGACCTCCGCAAGTTGGCTGTCAACATGG
TGCCCTTCCCTCGTCTGCACTTCTTCATGGTTGGCTTCGCTCCCCTGACCAGCCGTGGTGCTTACTCTTTCCGC
GCWGTCACCGTTCCCGAGTTGACGCA-GCAGAT-
GTTCGACCCCAAGAACATGATGGCYGCCTCCGATTTCCGCAACGGTCGCTACCTGACGTGCTCTGCCATCTT
GTAAGATACA-TA----CCCCGAG------------TTATC-------------------------GAGAAGTGC-TTG---
TTTGCYAACCTM----
TAATGTCTAGCCGTGGCAAGATCTCCATGAAGGAAGTCGAGGACCAAATGCGCAACGTCCAGAACAAG-----
----

>H_chionostomum_STMA_14060

-----AGAGAACCCCTGA-----ACGCG-TCCC--------------GAAAAAT-----------CCC---CAAC--TTCCCC-TGAT-----
-TTCATACT-CCTACCCCTC----CACGCAC---------AAA---CAC--
CACAAGCCCCA--------------AAACGAGATGT--------TCCGA----TT-----CCC---AACA---CCGC--------------
AAA-ATA------------------C---CAC------A--G-----------------------GATTCG--------------
AGAAATTAGAAGCTAA-CCATA---TC---TCTTCAT-----------
CGTAATAGGTTCACCTCCAGACCGGCCAATGCGTAAGAACCACCTA---CACC-------CTC-AACTACC-----

GTCGATCGAC-----------------GCGATAG-------------GAAAAC-ATGGCGG-----GGCTCACACGG---A-TTG-----
TA-TAGGGTAACCAAATTGGTGCTGCTTTCT---
-GGCAGACCATCTCTGGCGAGCACGGCCTCGACAGCAATGGCGTGTATGTATTCGT----------------
GTTCCATTGGTC-------------------AATTCCTCCGGCG-------GGACT--------------------------CCA--AAGC-------
TAAT-----------TGT-TCGT-
CAACAGCTACAACGGTACTTCTGAGCTCCAGCTGGAGCGCATGAGCGTCTACTTCAACGAGGTATGTCAC-
GATCG-----------------A----------A----AGC-----AA-AAAT--------------------ACCCAGCGC-A--------TGATCCG-
CT-A-----CTAAT--TTC-TT-CGA---CGTGT-
GCAGGCTTCCGGCAACAAGTACGTTCCCCGTGCCGTCCTCGTCGATCTCGAGCCCGGTACCATGGACGCTGT
CCGTGCCGGTCCTTTCGGTCAGCTCTTCCGACCCGACAACTTCGTCTTTGGTCAATCCGGTGCCGGCAACAA
CTGGGCCAAGGGTCACTACACGGAGGGTGC-CGAGCTTGTCG-
ACCAGGTTCTGGATGTCGTCCGTCGCGAGGCTGAGGGCTGCGACTGCCTCCAGGGTTTCCAGATCACCCAC
TCACTCGGTGGCGGTACCGGTGCCGGTATGGGTACCTTGTTGATCTCCAAGATCCGCGAGGAGTTCCCCGA
CCGCATGATGGCCACTTTCTCCGTCGTTCCCTCCCCCAAGGTCTCCGACACGGTCGTCGAGCCCTACAACGC
CACCCTCTCGGTCCACCAGCTGGTTGAGAACTCGGACGAGACGTTCTGTATTGATAACGAGGCCCTATACG
ACATCTGCATGCGTACCCTCAAGCTATCCAACCCCTCGTACGGCGACCTGAACCACCTGGTCTCGGCCGTCA
TGTCGGGCGTCACCACTTGCTTGCGATTCCCCGGCCAGCTGAACTCTGACCTGCGCAAGTTGGCTGTCAACA
TGGTGCCCTTCCCTCGTCTGCACTTCTTCATGGTCGGCTTCGCTCCCCTGACCAGCCGCGGTGCTTACTCTTTC
CGTGCCGTCACCGTTCCCGAGTTGACGCA-GCAGAT-
GTTCGACCCCAAGAACATGATGGCTGCCTCCGACTTCCGCAACGGTCGCTACCTGACGTGCTCTGCCATCTT
GTAGGATGCC-CG----CTCCTCATGA---------TTGCC--------------------------AATACGTGT-TTC---
CTTGCTGACCCT-----AAATTCTAGCCGTGGCAAGATCTCCATGAAGGAGGTTGAGGACCAGATGCG----------
----

>H_ochraceum_MUCL_54625

------------------CG-TCCCCCA-TCCC--------------CGAAA-------------CCC---CAAC--TTCCCC-TGAT------
TTCGTGTT-CCTACCCCTC----CACGCAC---------AAA---CAT--
CACAAACACGA--------------AACGAGA------------TTCGA----TT-----CCC---TACA---TTGC---------------AGA-
ACG-------------------C---TGG------A--G------------------------AATTCT---------------AAAAATCAAATGCTAA-
CCATA---TA---TTTTCAT------------CTCGATAGGTTCACCTCCAGACCGGCCAATGCGTAAGATCTACCTA---
TCCC-------TAC-AACTACC-----GACCATCGAC-----------------GCGATAGA------------AAGAAT-ATAGCGG-----
GGCTCACATGA---A-TTT-----TA-CAGGGTAACCAAATTGGTGCCGCTTTCT--

GGCAGACCATCTCTGGCGAGCACGGCCTCGACAGCAATGGCGTGTACGTGTTT-----------------------
AATTGGGC-------------------AATTCTCAACAAG-------GGAAT---------------------------TAA--GAAC-------TAAT-
----------AAT----T-
CGATAGCTACAACGGAACCTCTGAGCTCCAACTGGAGCGCATGAGCGTCTACTTCAACGAGGTACGTAAT-
AAACACC---------------A--CGCAACC-A----ACA-----AG-AAGT--------------------GCATGC-GA-A--------
AGACTCG-TT-A-----TTAAT--TACATT-TGA---CGTGC-
ACAGGCTTCCGGCAACAAGTATGTTCCCCGTGCTGTCCTTGTCGATCTTGAGCCTGGTACCATGGACGCCGT
CCGTGCTGGTCCCTTCGGTCAGCTCTTCCGACCCGACAACTTCGTCTTCGGTCAATCCGGTGCTGGCAACAA
CTGGGCCAAAGGTCACTACACTGAAGGTGC-TGAGCTGGTTG-
ACAACGTCTTGGATGTTGTCCGTCGCGAGGCTGAGGGCTGCGACTGCCTCCAGGGTTTCCAAATCACCCACT

CGCTCGGTGGTGGTACTGGTGCCGGTATGGGTACCTTGTTGATCTCCAAGATCCGCGAGGAGTTCCCTGAC
CGTATGATGGCCACTTTCTCCGTCGTTCCCTCCCCTAAGGTCTCCGACACCGTTGTCGAGCCTTACAACGCCA
CTCTCTCCGTCCACCAGCTGGTCGAGAACTCGGACGAGACCTTCTGCATTGATAACGAGGCTCTCTACGATA
TCTGCATGCGTACCCTAAAGCTATCCAACCCCTCGTATGGTGACCTGAACCACCTGGTCTCCGCCGTCATGTC
GGGTGTTACCACTTGCTTGCGATTCCCTGGCCAGCTGAACTCTGACCTCCGCAAGTTGGCTGTCAACATGGT
GCCCTTCCCTCGTCTGCACTTCTTCATGGTCGGCTTCGCCCCCCTGACCAGCCGTGGTGCCTACTCCTTCCGT
GCCGTCACCGTTCCCGAGTTGACGCA-GCAGAT-
GTTCGACCCCAAGAACATGATGGCTGCTTCTGACTTCCGAAATGGTCGCTACCTGACGTGCTCTGCCATCTT
GTAAGTGGCT-TAG---GTCCAAA------------TTGTC-------------------------GATTAGTAC-TTG---
TTTACTAACACT----AAATTAATAGCCGTGGCAAGATCTCCATGAAGGAGGT-------------------------------------
----

>H_guilanense_MUCL_57726

-------GGAACCCCTGA-----ACGCG-TCCC--------------AAA---------------CCC---CAACTTTTCCCC-TGAT------
TTCCTGTT-CCTACCCCTCC--ACACGCAC---------AAA--CAC--
CACAAGCACGA--------------AACGGCGATATTTCGA-TGCCCAA----TTCTCTACTC---TATA---TCGC------------
--GAAA-CCA-------------------C---CAC------G--ATA---------------------TTATCTT---------------
GGAATATGAAAGCTAA-CCATA---TA---TCTTCAT------------
TTCAATAGGTTCACCTTCAGACCGGCCAATGCGTAAGAACTACCTA---CACCT------AAC-AACCACC-A-
AAAACCATCGAC-----------------GCGAT---------------AAGAAT-ATGGCGG-----GGCTCACACGA---A-TTA-----
TT-TAGGGTAACCAAATCGGAGCTGCTTTCT--
-GGCAAACCATCTCTGGCGAGCACGGTCTCGACAGCAATGGCGTGTACGTATAT----------------------
GATTGGGC-------------------AATTCCCTCGAAA-------GGAGT---------------------------ACA--AAAT-------TAAT-
----------AGT-TTAT-
CAATAGCTACAACGGAACCTCTGAGCTCCAACTGGAGCGAATGAGCGTCTACTTCAACGAGGTATGCCAT-
AATTA-----------------A----------G----CCT-----AG-AAAT--------------------ACATGC-GC-A--------AAACCCT-TT-
A-----CTAAT--TAC-AT-CGA---TTCGC-
GCAGGCTTCCGGAAACAAGTATGTGCCTCGTGCCGTCCTCGTCGATCTCGAGCCCGGCACCATGGATGCCG
TTCGTGCTGGTCCCTTCGGTCAGCTCTTCCGACCCGACAACTTTGTCTTCGGTCAGTCCGGTGCCGGCAACA
ACTGGGCGAAGGGTCACTACACTGAGGGTGC-CGAGCTCGTTG-
ACCAGGTTTTGGATGTCGTTCGTCGTGAGGCTGAGGGCTGCGATTGCCTTCAGGGTTTCCAGATCACCCACT
CGCTCGGAGGTGGTACTGGTGCCGGTATGGGTACCCTGTTGATCTCCAAGATCCGTGAGGAGTTCCCCGAC
CGCATGATGGCCACTTTCTCCGTTGTGCCTTCCCCCAAGGTCTCTGACACCGTCGTCGAGCCTTACAACGCCA
CTCTTTCTGTCCACCAGCTGGTCGAGAACTCTGACGAGACCTTCTGCATTGACAACGAGGCTCTGTACGACA
TCTGCATGCGTACCCTCAAGCTATCCAACCCCTCGTATGGTGATCTGAACCATCTGGTCTCCGCTGTCATGTC
CGGTGTCACCACCTGTCTGCGATTCCCTGGCCAGCTGAACTCTGATCTCCGCAAGTTGGCCGTGAACATGGT
GCCCTTCCCTCGTCTGCACTTCTTCATGGTTGGCTTTGCTCCCCTGACCAGCCGCGGTGCTTACTCTTTCCGTG
CCGTCACCGTTCCCGAGTTGACTCA-GCAGAT-
GTTCGACCCCAAGAACATGATGGCTGCTTCTGACTTCCGCAACGGTCGTTACCTGACGTGCTCCGCCATCTT
GTAAGAAACC-TG----CTCCGAG------------TCGTT-------------------------AATG-----------------------------
AATGCT--

>H_texense_DSM_107933

-----GAGGAACCCCTGA-----ACGCG-TCCC-------------AAA--------------CCC---CAACTTTTCCCC-TGAT------
TTCCTGTT-CCTACCCCTCC--ACACGCAC---------AAA--CAC--
CACAAGCACGA-------------AACGGCGATATTTCGA-TGCCCAA----TT-----CTC---TATA---TCGC-------------
GAAA-CCA------------------C---CAC------G--ATAT----------------TGTTTATCTT--------------
GGAATAGGAAAGCTAA-CCATA---TA---TCTTCAT-----------
TTCAATAGGTTCACCTTCAGACCGGCCAATGCGTAAGAACTACCCA---CACCT------AAC-AACCACCAA-
AAAACCATCGAC----------------GCGAT--------------AAGAAT-ATGGCGG-----GGCTCACACGA---A-TTA-----
TT-TAGGGTAACCAAATCGGTGCTGCTTTCT---
-GGCAAACCATCTCTGGCGAGCACGGTCTCGACAGCAATGGCGTGTACGTATAT----------------------
GGTTGGGC------------------AATTCCCTCGAAA-------GGAGT-------------------------ACA--AAAT-------TAAT-
----------AGT-TTGT-
CAACAGCTACAACGGAACCTCTGAGCTCCAGCTGGAGCGAATGAGTGTCTACTTTAACGAGGTACGCCAT-
AATCA----------------A----------G----ACT-----AG-GAAT-------------------ACATGC-GC-A--------AATCCCT-TT-
A-----CTAAT--TAC-AT-CGA---TTTGC-
GCAGGCTTCCGGAAACAAGTATGTGCCCCGTGCCGTCCTCGTCGATCTCGAGCCCGGCACCATGGATGCCG
TCCGTGCTGGTCCTTTCGGTCAGCTCTTCCGACCCGACAACTTTGTCTTCGGTCAGTCCGGTGCCGGCAACA
ACTGGGCCAAGGGTCACTACACTGAGGGTGC-CGAGCTCGTTG-
ACCAGGTTTTGGATGTCGTTCGTCGTGAGGCTGAGGGCTGCGACTGCCTTCAGGGTTTCCAGATCACCCACT
CGCTCGGTGGTGGTACTGGTGCCGGTATGGGTACCCTGTTGATCTCCAAGATCCGCGAGGAGTTCCCCGAC
CGCATGATGGCTACTTTCTCCGTCGTGCCTTCCCCCAAGGTCTCTGACACCGTCGTCGAGCCTTACAACGCCA
CTCTCTCTGTCCACCAGCTGGTCGAGAACTCCGACGAGACCTTCTGCATTGACAACGAGGCTCTGTACGACA
TCTGCATGCGTACCCTCAAGCTATCCAACCCCTCGTATGGTGACCTGAACCACCTGGTCTCGGCCGTTATGTC
TGGTGTCACCACCTGCTTGCGATTCCCAGGCCAGCTGAACTCTGATCTCCGCAAGTTGGCTGTGAACATGGT
GCCCTTCCCTCGTCTGCACTTCTTCATGGTTGGCTTCGCTCCCCTGACCAGCCGCGGTGCTTACTCTTTCCGTG
CCGTCACCGTTCCCGAGTTGACTCA-GCAGAT-
GTTCGACCCCAAGAACATGATGGCTGCCTCTGACTTCCGCAACGGTCGTTACTTGACGTGCTCTGCCATCTT
GTAAGATACC-TG----CTCCAAG-----------TTATT-------------------------AATATT----TCG---TATGCTGACCCT-
----GAATTCTAGCCGTGGCAAGATCTCCATGAAGGAGGTT--

>H_canariense_MUCL_47224

---TGCTGACCTGAGCCGAGTA------
----CGAGACGGGAGACGGGAGAAGAACCCCTGA-----ACGCG-TCCC-------------AAAAC------------CCC---
CCAACTTTCCCC-TGAT------TTCCTGTT-CCTACCCCTCC--ACACGCAC---------AAA------------------------------
---------------------CAC--CACAAGCACGA-------------AACGACGATATTTCCATTTCCCGA----TT-----CCC---
TATA---TCGCGAAC---------CGAAA-TCA------------------C---CAT------G--C----------------------AATCTT------
---------GGAATATGAAAGCTAA-CCATA---TA---TCTTGAT-----------
TTGAATAGGTTCACCTTCAGACCGGCCAATGCGTAAGAACTACCTA---CTCCTACACATAAC-AACCGCC----
GAATCATCGAC----------------GCGATAGT-----------AAGAAC-ATAGCGG-----GGCTCACAT-----A-TTT-----
AT-TAGGGTAACCAAATTGGTGCTGCTTTCT---
-GGCAGACCATCTCTGGCGAGCACGGTCTCGACAGCAATGGCGTGTACGTATTT----------------------
GGCTGGGC------------------AATTTATTCAACG-------AGAGT-------------------------ACA--AAAT-------TAAT-
----------AGT-TTGT-

CAACAGCTACAACGGAACCTCTGAGCTCCAGCTGGAGCGCATGAGCGTTTACTTCAACGAGGTAGGTCAT-
AATCA-----------------G----------G----ACC-----AG-GAAT--------------------GCATGC-GC-A--------AGAGTCG-
TT-A-----CTAAT--TAC-TT-TCA---TACGC-
GCAGGCCTCTGGAAACAAGTATGTGCCCCGTGCCGTTCTCGTCGATCTCGAGCCCGGTACCATGGATGCCG
TCCGTGCTGGTCCCTTTGGTCAGCTCTTCCGACCCGACAACTTCGTCTTCGGTCAGTCCGGTGCTGGCAACA
ACTGGGCCAAGGGTCACTACACCGAGGGTGC-CGAGCTCGTTG-
ACCAGGTTTTGGATGTCGTTCGTCGCGAGGCCGAGGGCTGCGACTGCCTTCAAGGTTTCCAGATCACCCACT
CGCTCGGTGGTGGTACCGGTGCCGGTATGGGTACCTTGTTGATCTCCAAGATCCGCGAGGAGTTTCCCGAC
CGCATGATGGCCACTTTCTCCGTCGTGCCCTCCCCCAAGGTCTCCGACACCGTCGTCGAGCCTTACAACGCC
ACCCTCTCCGTCCACCAGCTGGTCGAGAACTCGGACGAGACCTTCTGCATTGACAATGAGGCTCTGTACGAC
ATCTGCATGCGTACCCTCAAGCTGTCCAACCCCTCGTATGGTGACCTGAACCACCTGGTCTCGGCCGTCATG
TCTGGTGTCACCACCTGCCTGCGATTCCCTGGTCAGCTGAACTCGGATCTCCGCAAGTTAGCCGTCAACATG
GTGCCCTTCCCTCGTCTGCACTTCTTCATGGTCGGCTTTGCTCCCCTGACCAGCCGCGGTGCTTACTCTTTCCG
TGCCGTCACCGTTCCCGAATTGACCCA-GCAGAT-
GTTCGACCCCAAGAACATGATGGCTGCTTCCGACTTCCGTAACGGTCGTTACCTGACGTGCTCTGCCATCTT
GTAAGATACC-CG----TTCCGAG------------TCGTC--------------------------GATAAGYGTTTTA---
TATGCTAACCCT-----
GAATTCTAGCCGTGGCAAGATCTCCATGAAAGAGGTCGAGGACCAGATGCGCAACGTCCAGAACAAG------
----

>H_laschii_MUCL_52796

---CTGCTGACCACAGCCGAGT-------
-------------ACGAGACGGGAGAACCCCTGA-----ACGCG-TTCC--------------CAAAC-------------CCC---
CAATTTCCCCCC-TGAT------TTCCTGCT-CCTACCCCTCC--ACACGCAC---------AAA-------------------------------
----------------------CAC--CACAAGCACGA--------------AACGACGATATTC--A-TTCCCGA----TT-----CTC---
TGTA---TCGC--------------GAAA-CCA-------------------C---CTT------G--A------------------------GAACTT----------
-----GAAAAATGAAAGCTAA-CCATA---TA---TCTTCAT------------
CTGGATAGGTTCACCTTCAGACCGGCCAATGCGTAAGAACTACCTA---CATGC--ATCTAAT-AACCACC-----
CATCATCAAC-----------------GCGATAGG------------AAGAAC-ATAGCGG-----GGCTCACATGG---A-TTT---
TTAT-TAGGGTAACCAAATTGGTGCTGCTTTCT---
----GGCAAACCATCTCTGGCGAGCACGGTCTCGACAGCAATGGCGTGTACGTATCT-----------------------
GGCTGGGC-------------------AATCCCCTCAACA-------GGAGT---------------------------GCA--AAAT-------TAAT-
----------ATT-TCGT-
CAACAGCTACAACGGAACCTCTGAGCTCCAACTGGAGCGCATGAGCGTCTACTTCAACGAGGTACGCCAC-
AATCA-----------------A----------G----ATC-----TG-GAAT--------------------ACATGC-GCGA--------AGACTCG-
TT-A-----CTGAT--TAC-TT-GGA---TATGC-
GTAGGCTTCCGGCAACAAGTACGTACCCCGTGCTGTTCTCGTCGATCTCGAGCCCGGTACCATGGATGCCGT
CCGTGCTGGTCCTTTCGGTCAGCTCTTCCGACCCGACAACTTCGTCTTCGGTCAGTCGGGTGCTGGCAACAA
CTGGGCCAAGGGTCACTACACTGAGGGTGC-CGAGCTCGTTG-
ACCAGGTTTTGGATGTCGTTCGTCGCGAGGCCGAGGGCTGCGACTGCCTTCAGGGTTTCCAGATCACCCAC
TCGCTCGGTGGTGGTACCGGTGCCGGTATGGGTACCTTGTTGATCTCCAAGATCCGCGAGGAGTTCCCCGA
CCGCATGATGGCTACTTTCTCCGTCGTGCCCTCCCCCAAGGTCTCTGACACGGTCGTCGAGCCTTACAACGC
CACCCTCTCGGTCCACCAGCTGGTCGAGAACTCGGACGAGACCTTCTGCATTGACAACGAGGCTCTGTACG
ACATCTGCATGCGTACCCTGAAGCTATCCAACCCCTCGTATGGTGACCTGAACCACCTAGTCTCGGCCGTCA

TGTCCGGTGTCACCACCTGCTTGCGATTCCCCGGCCAGCTGAACTCTGATCTCCGCAAGTTGGCTGTCAACA
TGGTGCCCTTCCCTCGTCTGCACTTCTTCATGGTCGGCTTCGCTCCTCTGACCAGCCGCGGTGCTTACTCCTTC
CGTGCTGTCACCGTTCCCGAGTTGACTCA-GCAGAT-
GTTCGACCCCAAGAACATGATGGCTGCTTCTGACTTCCGCAACGGTCGTTACCTGACGTGCTCTGCCATCTT
GTAAGATACC-CG----CCCCAAG------------TCAGT-------------------------AACAAGTGCTTTA---
TTYGCTGACCCT-----
GAACTCTAGCCGTGGCAAGATCTCCATGAAGGAGGTCGAGGACCAGATGCGCAACGTCCAGAACAAGA----
----

>H_pilgerianum_STMA_13455

---CCC---CAAC--TTCCCC-TGAT------T--------
TCCCCCCCTC----CACGCAC---------AAA---CAC--CACAACCACAG--
--------------ACCGAGAT--------CTCAA----TT-----CCC---TACA---TTGC--------------AGA-TTA------------------
CCTATAT------A--G------------------------GATTTT---------------GGAAGACAAAAGCTAA-CCATA---TC---
TTTTCCA------------TATAATAGGTTCACCTCCAGACTGGCCAATGTGTAAGTACTACCTA---CACC-------TAC-
AGCTGCC-----GACCAGCGAC-----------------GCGATAGG------------AAGAAT-ATGGCAG-----
GGCTCACATATT-TA-TTT-----TA-TAGGGTAACCAAATTGGTGCTGCTTTCT--------------------------------------
----
GGCAAACCATCTCTGGCGAGCACGGTCTCGACAGCAATGGCGTGTATGTACATTT--------------------
CATTGGTC------------------AATTGTGCAGG---------GAGGA-------------------------TCA--AAAC-------TAAC--
---------AGT-TGGG-
TTATAGCTACAACGGAACCTCTGAGCTCCAGCTCGAGCGCATGAGCGTCTACTTCAACGAGGTATGCCAA-
AAAAA-----------------AATAATAGCGGA----GGC-----AA-GAAT--------------------ACATGC-GC-A--------
GGACCCG-TA-A-----CTAAT--TAC-CT-TGA---CGTTT-
ACAGGCTTCCGGCAACAAGTATGTTCCCCGTGCCGTCCTCGTCGATCTCGAGCCCGGTACCATGGATGCCGT
CCGTGCTGGTCCTTTCGGTCAACTCTTCCGACCCGACAACTTTGTTTTCGGTCAATCCGGTGCCGGCAACAAC
TGGGCCAAGGGTCATTACACTGAGGGTGC-CGAGCTTGTCG-
ACCAGGTTTTGGATGTCGTTCGTCGTGAGGCTGAGGGCTGCGATTGCCTCCAGGGTTTCCAGATTACCCACT
CGCTCGGTGGTGGTACCGGTGCCGGTATGGGTACCTTGTTGATCTCCAAGATCCGCGAGGAGTTTCCCGAC
CGCATGATGGCCACCTTCTCCGTTGTTCCCTCCCCCAAGGTCTCCGATACCGTTGTCGAGCCTTACAACGCCA
CTCTCTCCGTCCACCAGCTGGTCGAGAACTCAGACGAGACCTTCTGTATCGATAACGAGGCTCTGTACGACA
TCTGCATGCGTACCCTGAAGCTATCCAACCCTTCGTACGGTGACCTGAACCACTTGGTCTCCGCCGTCATGTC
GGGTGTCACCACTTGCTTGCGATTCCCTGGTCAGCTGAACTCTGACCTCCGCAAGTTGGCTGTCAACATGGT
GCCCTTCCCTCGTCTGCACTTCTTCATGGTCGGCTTCGCTCCCCTGACCAGCCGTGGTGCTTACTCCTTCCGT
GCTGTCACCGTCCCCGAGTTGACCCA-GCAGAT-
GTTCGACCCCAAGAACATGATGGCTGCCTCTGACTTCCGCAACGGTCGCTACCTAACATGCTCTGCCATCTT
GTAAGCTTCC-TCA---CTTCCCA------------TTGTC-------------------------GATTAATAT-TTG---
TTTTCTGACCTA-----GAATTCTAGCCGTGGCAAGATCTCCATGAAGGAAGTTGAGGACCAGATGCGCAAC---
----

>H_wuzhishanense_FCATAS2708

----------------------CAGA-TTA------------------C---TGC------A--A-----------------------ACTTCC--------------
AGACGACGAAAGCTAA-CCATA---TT---TCTTAA------------
AATAATAGGTTCACCTCCAGACTGGCCAATGCGTAAGATTTACCTA---CACC-------TGC-AGCGGAC-----
GATCATCGAC----------------GCGATAGG------------AAGAAT-ATAGTCG-----GGCTCAC-------A-TAT-----TA-
TAGGGTAACCAAATTGGTGCCGCTTTCT---
GGCAGACCATCTCTGGCGAGCACGGTCTGGACAGCAATGGCGTGTATGTTGTTG---------------------
CATCGGGC------------------AATTGCTTAA----------TGGAT--------------------------TGA--AGAC-------TAAT----
-------TAT------
TCATAGCTACAACGGAACCTCCGAGCTCCAGCTGGAGCGCATGAGCGTCTACTTCAACGAGGTACGTCTA-
ATCAA----------------A-----AACAGG----AGC-----AA-GAAT-------------------ACATGC-GC-A--------
AGACTCA-TT-A-----CTAAT--TAC-CT-GGA---CGTGC-
GCAGGCTTCCGGCAACAAATACGTCCCCCGTGCCGTCCTTGTCGATCTCGAGCCCGGTACCATGGATGCCGT
CCGTGCTGGTCCTTTCGGTCAACTCTTCCGCCCAGACAACTTCGTCTTTGGTCAATCCGGTGCCGGCAACAAC
TGGGCCAAGGGTCACTACACTGAGGGTGC-CGAACTTGTCG-
ACCAGGTTCTGGATGTCGTCCGTCGTGAGGCTGAAGGCTGCGACTGCCTGCAGGGTTTCCAGATCACCCAC
TCGCTCGGTGGTGGTACCGGTGCCGGTATGGGTACCTTGTTGATCTCCAAGATCCGCGAGGAGTTCCCCGA
CCGCATGATGGCTACTTTCTCCGTCGTTCCCTCCCCCAAGGTCTCCGACACCGTCGTCGAGCCCTACAACGCC
ACCCTCTCCGTCCACCAGCTGGTCGAGAACTCGGACGAGACCTTCTGCATTGATAACGAGGCTCTGTACGAC
ATCTGCATGCGCACCCTGAAGCTATCCAACCCTTCGTACGGTGACCTGAACCACCTCGTCTCCGCCGTCATGT
CGGGTGTCACCACTTGTTTGCGATTCCCCGGTCAGCTGAACTCTGACCTCCGCAAGTTGGCCGTCAACATGG
TGCCGTTCCCTCGTCTGCACTTCTTCATGGTCGGCTTCGCTCCCCTGACCAGCCGTGGTGCTTACTCTTTCCGT
GCCGTCACCGTCCCCGAGTTGACCCA-GCAGAT-
GTTCGACCCCAAGAACATGATGGCTGCTTCTGACTTCCGCAACGGTCGCTACCTGACGTGCTCTGCCATCTT
GTAAGTTCTC-CC----ATTCAG------------TTGTC-------------------------GATCAATTT-TTG---
TTTTCTAACCCG-----
AAAAACTAGCCGTGGCAAGATCTCCATGAAGGAAGTTGAGGACCAGATGCGCAACGTCCAGAACAAGAAC
TTCTCTTC------------------------------

>H_rubiginosum_MUCL_52887

TAACATGCGTGAGATTGTAAGTCTT----------------------------------ACCCTCTTTAATGTTTA-----------
CTGCTGGCCCGAGCCGAGT-------------------ACGAGATGGGAGAACCCCTGA-----ACGCG-TCCC-------------
AAAAC------------CCC---CAAT--TTCCCC-TGAT------TTCCTGTT-CCTACCCCTC----CACGCAC--------AAAA---
---CAC--CAGAGGCACGA-------------AACGACGATATCTCCA-
TTGCCAA----TT-----GCG---TATA---TCGC-------------GAAA-CCA------------------C---CAC------A--A----------
-------------GACCTT---------------GGAATACAAAAGCTAA-CCATA---CA---TCTTTAT------------
TTCGATAGGTTCATCTTCAGACCGGCCAATGCGTAAGAATTACCTA---TACCT------AAG-AACCACC-----
GATCAACAAC----------------GCGATGG-------------AAAGAT-ATGGCGG-----GGCTCACATGA---A-TTT-----
TGTTAGGGTAACCAAATTGGTGCTGCTTTCT---
--GGCAAACCATCTCTGGCGAGCACGGTCTCGACAGCAATGGCGTGTATGTGTTT----------------------
GACCAGCG------------------AATTCCCCCGACG-------AGAGT--------------------------TCA--AAAT-------TAAT-
----------ATT-TTGT-
CAACAGCTACAACGGAACCTCTGAGCTCCAGCTGGAGCGCATGAGCGTCTACTTCAACGAGGTACGCCAC-
AATCA----------------A----------A----TCC-----GA-CAAT-------------------CCATGC-GCAA--------GGACTCG-

TT-A-----CTAAT--TAC-TT-CGA---TATGC-
GCAGGCTTCCGGAAACAAGTATGTGCCCCGTGCCGTCCTCGTCGATCTCGAGCCTGGTACCATGGATGCCG
TCCGCGCTGGTCCCTTCGGTCAGCTCTTCCGACCCGACAACTTCGTTTTCGGTCAGTCCGGTGCTGGCAACA
ACTGGGCCAAGGGTCACTACACTGAGGGTGC-CGAACTCGTTG-
ACCAGGTTTTGGATGTCGTTCGTCGTGAGGCTGAGGGCTGCGACTGCCTTCAGGGTTTCCAGATCACTCACT
CGCTCGGTGGTGGTACCGGTGCCGGTATGGGTACCTTGTTGATCTCCNAGATCCGCGAGGANTTCCCCGAC
CGCATGATGGCTACTTTCTCCGTCGTCCCCTCCCC--

>H_hypomiltum_MUCL_51845

CGAGCCAAAATAAGACGAACAAACCCCTGA-----ACGCG-TTCC-----------CGAAAAAA-------------CCC---
CAAC-TTTCCCC-TGATT-----TTCTTGCT-TTTGCCCCTC----CACCCAC---------AAA---------------------------------
--------------------CAC--AAACATCACGA--------------TGGAAGAAT----------CGAGG----TG-----TCG---TGTC---
TCGC---------------AGT-TTT-----TA-T----------C---TAC----------------------------------AGAATC---------------
AGAAAATGGAAGCTAA-TCATA---TC---TCTTCGT------------
CTTAATAGGTTCACCTTCAGACCGGCCAATGCGTAAGAACTACCTA---TATT-------TCC-AAC--------
TGATTATCAAC-----------------GCGATAGCA-----------AGGAAT-ATTGCGG-----GGCTCACATGG---A-TTT---
TTAT-TAGGGTAACCAAATTGGTGCTGCTTTCT---
----GGCAAACCATCTCTGGCGAGCACGGTCTCGACAGCAATGGCGTGTATGTGTTT------------------------
ATTATTC-------------------AATTCCTATAACGATAA---CAAGT---------------------------TAA--AAGC-------TAAT-
----------GGT-TGGC-
CAATAGTTACAACGGAACCTCTGAGCTCCAGCTGGAGCGCATGAGCGTCTACTTCAACGAGGTAAGCTAC-
GATAG-----------------A----------A----GCT-----AC-AAAT--------------------ACGTGT-CC-A--------AATCTAG-
GC-A-----CTAATC--------CTG---CTTGC-
ATAGGCTTCCGGTAACAAGTATGTTCCCCGTGCCGTTCTCGTCGATCTCGAGCCCGGTACCATGGATGCCGT
CCGTGCTGGTCCCTTCGGTCAGCTCTTCCGACCCGACAACTTCGTCTTTGGTCAGTCCGGTGCTGGCAACAA
CTGGGCCAAGGGTCACTACACTGAAGGTGC-TGAGCTTGTTG-
ACAATGTTTTGGATGTCGTTCGTCGTGAGGCTGAGGGCTGTGACTGCCTTCAGGGTTTCCAGATCACCCACT
CTCTCGGTGGTGGTACTGGTGCCGGTATGGGTACCCTGTTGATCTCCAAGATCCGCGAAGAGTTCCCTGACC
GCATGATGGCTACCTTCTCCGTTGTTCCTTCTCCCAAGGTTTCGGATACCGTTGTTGAGCCTTACAACGCCAC
TCTCTCCGTCCACCAGCTGGTCGAGAACTCAGACGAGACCTTCTGCATTGACAACGAAGCTCTATACGACAT
CTGCATGCGTACCCTGAAGCTATCCAACCCCTCGTACGGTGACCTGAACCACCTGGTTTCCGCTGTCATGTCT
GGTGTCACTACCTGCTTGCGTTTCCCCGGTCAGCTAAACTCTGACCTCCGCAAGCTGGCTGTCAACATGGTG
CCTTTCCCTCGTCTGCACTTCTTCATGGTCGGCTTCGCGCCCCTGACCAGCCGTGGTGCTTACTCTTTCCGTGC
CGTCACCGTTCCTGAGTTGACTCA-GCAGAT-
GTTCGATCCCAAGAACATGATGGCTGCTTCTGACTTCCGTAACGGTCGTTACCTGACATGCTCTGCTATCTTG
TGAGATAAC-CC----TTCCTAA------------TTATT--------------------------GACTAATAT-TTG---TTTACTAACGTA-
-----AATTCTAGCCGTGGCAAGATCTCCATGAAGGAAGTTGAGGACCAGATGCGCAACGTC--------------------
----

>H_munkii_MUCL_53315

-----------------------TAGCG------------------GCAAGATA-----------CCC---CAAC-TTTCCCC-TGAT------
TTTCTGCT-TCTACCCCTC----CACACAT---------AAA---CAC--
CACGAACACCA--------------GACATGAAT----------CGAGA----TA-----CCA---CATC---TCAT--------------AG--
TTG--------T----------T---CGT-----------------------------------CTATAT-------------ACAGAGAATGAAAGCTAA-
TCATA---TC---TATTCAT------------CTTAATAGGTTCACCTTCANACCGGCCAATGCGTAAGAACTACCTA---
GAAC-------TCC-AACCGTC-----GACTATCGAC-----------------GCGATAG---------------GAAT-ATTGCGG-----
GGCTCACATGG---ATTTT-----TG-TAGGGTAACCAAATTGGTGCTGCTTTCT---

GGCAGACCATCTCTGGCGAGCACGGTCTCGACAGCAATGGCGTGTACGTGTTA------------------------
CATTGGTC-------------------AATTCTCGTGACG-------AGTAC---------------------------A-----AAAC-------TAATA--
--------AGC-TCAT-
TAATAGTTACAACGGAACCTCTGAGCTCCAGCTAGAGCGCATGAGCGTCTACTTCAACGAGGTATGCCAC-
AACGA-----------------A----------G----CCC-----AG-AAAT--------------------GTATGC-GC-A--------AGTCCCG-
TC-A-----CTAAT--TAT-CC-CTA---CGTGC-
GCAGGCTTCCGGCAACAAGTATGTTCCCCGTGCCGTCCTTGTCGATCTTGAGCCCGGTACCATGGATGCCGT
CCGTGCTGGTCCCTTCGGTCAGCTCTTCCGACCCGACAACTTCGTCTTTGGTCAGTCCGGTGCTGGCAACAA
CTGGGCCAAGGGTCACTACACGGAAGGTGC-CGAACTCGTTG-
ACAATGTCTTGGATGTCGTTCGTCGTGAGGCTGAGGGCTGCGACTGCCTTCAGGGCTTCCAGATCACTCACT
CGCTCGGTGGTGGTACCGGTGCCGGTATGGGTACTTTGTTGATCTCCAAGATCCGCGAGGAGTTCCCTGAC
CGCATGATGGCCACCTTCTCCGTTGTCCCTTCCCCCAAGGTCTCGGATACCGTTGTCGAGCCTTACAACGCCA
CCCTCTCCGTGCACCAGCTGGTTGAGAACTCGGATGAGACTTTCTGCATTGACAACGAGGCTCTGTACGACA
TCTGCATGCGTACTCTGAAGCTATCTAACCCCTCGTATGGTGACCTGAACCACTTGGTCTCTGCCGTCATGTC
TGGCGTTACCACCTGCTTGCGATTCCCTGGTCAGCTGAACTCTGACCTCCGTAAGCTCGCTGTCAACATGGT
GCCCTTCCCCCGTCTGCACTTCTTCATGGTCGGTTTCGCGCCCCTGACCAGCCGTGGCGCTTACTCGTTCCGT
GCTGTCACCGTTCCCGAGTTGACTCA-GCAAAT-
GTTCGACCCCAAGAACATGATGGCTGCCTCTGACTTCCGCAACGGTCGTTACCTGACGTGCTCGGCCATCTT
GTAAGACTTT-CCTA--CTCTAAA------------TAGTC--------------------------GATTCGTACTTTA---
TTTGCTAACCTA-------GATCTAGCCGTGGCAAGATCT---

>H_samuelsii_MUCL_51843

GCTGAGCACAAGATGACAACCCCTGA-----ACGCG-TCCC--------------GAAAACC-----------CCC---
CAACTTTCCCCT-TGAT------TTCCTGCT-ACTGCCCCTC----CACCTAC---------AAA------------------------------
--------------------CAC--CACAAACACCACG------------AATACCGCAAACCTGA-ATCGAGA----TA-----TTA---
CATC---TCGC--------------------------CACT----------C---TCC------G--T------------------------CTCTAT--------------
-AGAAAATGAAAGCTAA-TCGTA---TC---TCTTCAT------------
CTCGATAGGTTCACCTTCAGACCGGCCAATGCGTAAGAAATACCCTA--TACA-------TCT-AACTATC-----
GACTATTGAC-----------------GCGATAG---------------GAAT-ATTGCGG-----GGCTTACAATG--GACTTT-----
TG-TAGGGTAACCAAATTGGTGCCGCTTTCT---
-GGCAGACCATCTCTGGCGAGCACGGTCTCGACAGCAATGGCGTGTACGTGCTT------------------------
CATCGGTC-------------------AATTCGCAAGATG-------AGTA----------------------------CAA--AAAC-------TGAC--

---------AGC-TCGT-
TACTAGCTACAACGGAACCTCTGAGCTCCAGCTGGAACGCATGAGCGTCTACTTCAACGAGGTACGCCAC-
AGTAG-----------------A----------G----TCCACCCAAG-GAAT--------------------ACATGC-GC-G--------
AGTCCTG-TC-A-----CTAATTATAT-TT-CTT---CGTGC-
GTAGGCTTCCGGCAACAAGTATGTTCCCCGTGCCGTCCTCGTCGATCTCGAGCCTGGTACCATGGATGCCGT
CCGTGCTGGCCCCTTCGGACAGCTCTTCCGACCGGACAACTTCGTCTTTGGTCAGTCCGGTGCTGGCAACAA
CTGGGCCAAGGGTCACTACACTGAGGGTGC-CGAGCTCGTTG-
ACAATGTCTTGGATGTTGTTCGTCGCGAGGCTGAAGGCTGCGACTGCCTTCAGGGTTTTCAGATCACCCACT
CGCTCGGTGGCGGTACTGGTGCCGGTATGGGTACCTTGCTGATCTCCAAGATCCGCGAGGAGTTCCCTGAC
CGCATGATGGCCACCTTCTCCGTTGTTCCTTCCCCCAAGGTCTCGGATACCGTTGTAGAGCCTTACAACGCCA
CCCTCTCCGTCCACCAGCTGGTCGAGAACTCGGACGAGACTTTCTGCATTGACAACGAGGCTTTGTACGACA
TCTGCATGCGTACCTTGAAGCTATCCAACCCCTCGTATGGTGATCTGAACCACCTAGTCTCCGCCGTCATGTC
TGGCGTCACCACCTGCTTGCGATTCCCTGGTCAGCTGAACTCTGACCTCCGTAAGCTGGCTGTCAACATGGT
GCCCTTCCCTCGTCTGCACTTCTTCATGGTTGGCTTCGCGCCCCTGACCAGCCGTGGTGCTTACTCGTTCCGT
GCCGTCACCGTTCCCGAGTTGACTCA-GCAGAT-
GTTCGACCCCAAGAACATGATGGCTGCCTCTGACTTCCGCAATGGTCGCTACCTGACGTGCTCCGCCATCTT
GTAAGACTGC-CCG---CCCTAAG------------TAGTC--------------------------GATTCGTGCTTTG---
CTTGCTGACCTT------
AATTCTAGCCGTGGTAAGATCTCCATGAAGGAGGTTGAGGACCAGATGCGCAACGTCCAGAAC--------------
----

>H_addis_MUCL_52797

--------------CTGA-----ACGCG-TCCC--------------GAAAAAC-----------CCC---CAAT---TTCTC-CTGA------
TTCCTGCT-CCTACCCCTC----CACACAC---------AGA---
TATACCACAAGCAGGA---AAAA---AAAA------
GATGTCATTGAG-TTT-----TA------------C---CGT------ATCGCA----------------------TATTATCAG-----------
AGGAAATGAAAAGCTAA-TCGTA---TC---TCTTCGTC-----------
TTAAATAGGTTCACCTTCAGACCGGCCAATGTGTAAGAATCAACCTAA-AACC----------------------
TACAACGAAGA-----------------ACGATAG---------------GAAG-ATAGCGG-----GGCTCACACGA---T-TTT-----
AA-TAGGGTAACCAAATTGGTGCTGCTTTCT--
-GGCAGACCATTTCTGGTGAGCACGGCCTTGACAGCAATGGAGTGTATGTAATT---------------------------
GGTC-------------------AACCCTATTGAAT-------AGGGTA--------------------------CGG--GAGT-------TAAT-------
--------TGTT-
TGTCAGGTATAACGGAACCTCCGAGCTTCAGCTGGAGCGCATGAGCGTCTACTTCAACGAGGTATGTTAC-
AATGA-----------------A----------G----AGCAA---GA-AAAA--------------------AGATGC-GC-A--------AGATCAA-
TC-A-----CTAAT--TAC-TT-GAA---TACGC-
GCAGGCTTCCGGTAACAAGTATGTTCCTCGTGCCGTTCTTGTCGATCTCGAACCCGGTACCATGGATGCCGT
TCGTGCTGGTCCCTTTGGTCAGCTCTTCCGACCCGACAACTTCGTTTTCGGTCAATCCGGTGCCGGAAACAA
CTGGGCCAAGGGTCACTACACCGAGGGTGC-TGAGTTGGTCG-
ACCAGGTCCTTGACGTCGTTCGTCGTGAGGCTGAGGGTTGCGATTGCCTCCAGGGTTTCCAGATTACCCACT
CGCTCGGTGGTGGTACCGGTGCCGGTATGGGTACCTTGTTGATCTCCAAGATTCGCGAGGAGTTCCCCGAC
CGTATGATGGCTACTTTCTCCGTCGTTCCTTCCCCCAAGGTTTCCGATACCGTTGTCGAGCCTTACAACGCTA
CCCTCTCGGTTCACCAGCTGGTCGAAAACTCGGACCAGACCTTCTGCATTGATAACGAGGCTTTGTACGACA

TCTGCATGCGTACCCTTAAGCTATCCAACCCTTCGTACGGTGACCTGAACCACCTGGTCTCGGCCGTCATGTC
GGGTGTTACCACTTGCTTGCGATTCCCTGGTCAGCTAAACTCTGATCTCCGCAAGTTGGCTGTTAACATGGT
GCCTTTCCCTCGTCTGCACTTCTTCATGGTCGGCTTTGCTCCTCTGACTAGCCGTGGTGCTTACTCTTTCCGTG
CTGTTACCGTTCCCGAGTTGACTCA-GCAAAT-
GTTCGACCCCAAGAACATGATGGCTGCTTCCGACTTCCGTAACGGTCGTTACCTAACGTGCTCTGCCATCTT
GTAAGTTTAT-TG----TATTTTCTAA---TAGTAATTG-TCC---CTCGCTAACTTT-
-----TACTCTAGCCGTGGCAAGATCTCAATGAAGGAGGTCGAGGACCAA--

>D_rogersii_YMJ_92031201

-AACATGCGTGAGATTGTAAGTCATGATG---------------------------------CAGCCCCCTTTGCCCCTC------------
GCCTCCCTCCAAGCCCAGTC-CAAG-------ACCAAGAAAAAAAAAAACCGAGTACCGAA-----ACGCG------------
-------------A-----------CG-----------CCCCT-CCAC--------------ACACGCTCTGT----CACGCAT---------ACA---------
--TGA--CACGACCACCA--------------------CCGCGTCT------TGTGGT--CCT--
---CTC---GGCG---GCGT------TGAGCTGTCAACAACC-----CA-----------A---CAC------A--A-----------------------
-CAGGATGAA--------------AATTGCAAAGCTAACCCGTG---TC---TCGTTCT------------
CTTGATAGGTTCACCTCCAGACCGGCCAATGCGTAAGTGCCACAAC---CAACA-----G--------------
GAACCCCCATC-----------------GCGCTA----------------GAAT-CTGGCAG-----CGCTCACACAG---G-ATT-----
CG-CAGGGCAACCAAATTGGTGCTGCTTTCT--
--GGCAGACCATCTCGGGCGAGCACGGTCTCGACAGCAATGGCGTGTACGTATATCT---------------------
AGACCCGGCG----------------ATGTGTCGACACGGC-------AGGGA----------------------------CGG--
TAATGCTGACAGACG-----------GGT-CGGT-
GGACAGTTACAATGGCACCTCGGAGCTCCAGCTTGAGCGCATGAGCGTCTACTTCAACGAGGTACGTGGT-
TGCGG------------------C----------C----ATG-----T--GGAG---------------------GGATGC-CA-
TCGGACGGAGGACGAG-GA-C-----GCTAA--CGT-GC-CCC---GGGTG-
ACAGGCCTCTGGTAACAAGTACGTGCCTCGCGCCGTCCTTGTCGATCTCGAGCCCGGCACCATGGATGCCG
TCCGTGCTGGCCCCTTCGGCCAGCTCTTCCGCCCCGACAACTTCGTCTTCGGCCAGTCGGGTGCTGGCAACA
ACTGGGCCAAGGGCCACTACACCGAGGGCGC-CGAGCTCGTCG-
ACCAGGTCCTCGACGTCGTCCGCCGCGAAGCTGAGGGCTGCGACTGCCTTCAGGGCTTCCAGATCACCCAC
TCTCTCGGCGGTGGTACCGGTGCCGGTATGGGCACCCTGCTGATCTCCAAGATCCGCGAGGAATTCCCAGA
CCGCATGATGGCCACCTTCTCCGTCGTCCCCTCCCCCAAGGTCTCCGACACCGTCGTCGAGCCTTACAACGCC
ACCCTCTCCGTCCACCAGCTGGTCGAGAACTCTGACGAGACTTTCTGTATCGATAACGAGGCCCTGTACGAC
ATTTGCATGCGCACTCTGAAGCTGTCGAACCCCTCGTACGGTGATCTCAACCACCTCGTCTCTGCCGTCATGT
CCGGTGTCACCACTTGCCTGCGCTTCCCCGGTCAGCTAAACTCTGACCTGCGCAAGCTTGCCGTGAACATGG
TGCCCTTCCCTCGTCTACACTTCTTCATGGTCGGCTTCGCACCCCTGACCAGTCGCGGTGCCCACTCTTTCCG
CGCTGTCACCGTTCCCGAGTTGACCCA-GCAGAT-
GTTTGACCCCAAGAACATGATGGCTGCTTCGGATTTCCGCAACGGTCGATATCTCACGTGCTCTGCCATCTT
GTAAGCCTCG-TG----TTCACTC------------TCCTTCCTCTA-ACCCC--CCCCCCC-CCCCCAAACAAAC-CCA---
ACTGCTAACCCT---
AATCAAATTAGCCGTGGCAAGGTCTCGATGAAGGAGGTCGAGGACCAGATGCGCAATGTTCAGAACAAGA
ACTCGTCCTACTTCGTTGAGTGGATTCCCAACAACATCCAGA

>D_rogersii_GMBC0204

---A-----------CG-----------CCCCT-CCAC-------------
ACACGCTCTGT----CACGCAT---------ACA---TGA--
CACGACCACCA-------------------CCGCGTCT------TGTGGT--CCT-----CTC---GGCG---GCGT------
TGAGCTGTCAACAACC-----CA------------A---CAC------A--A------------------------CAGGATGGA--------------
AATTGCAAAGCTAACCCGTG---TC---TCGTTCT------------
CTTGATAGGTTCACCTCCAGACCGGCCAATGCGTAAGTGCCACAAC---CAACA-----G---------------
GAACTCCCATC-----------------GCGCTA----------------GAAT-CTGGCAG-----CGCTCACACGG---G-ATT-----
CG-CAGGGCAACCAAATTGGTGCTGCTTTCT---
--GGCAGACCATCTCGGGCGAGCACGGTCTCGACAGCAATGGCGTGTACGTATATCT--------------------
AGACCTGGCG----------------ATGTGTCGACACGGC-------AGGGA---------------------------CGG--
TAATGCTGACAGACG-----------GGT-CGGT-
GGACAGTTACAATGGCACCTCGGAGCTCCAGCTTGAGCGCATGAGCGTCTACTTCAACGAGGTACGTGGT-
TGCGG-----------------C----------C----ATG-----TG-GGAG--------------------GGATGC-CA-
TCGGACGGAGGACGAG-GA-C-----GCTAA--CGT-GC-CCC---GGGTG-
ACAGGCCTCTGGTAACAAGTACGTGCCTCGCGCCGTCCTTGTCGATCTCGAGCCCGGCACCATGGATGCCG
TCCGTGCTGGCCCCTTCGGCCAGCTCTTCCGCCCCGACAACTTCGTCTTCGGCCAGTCGGGTGCTGGCAACA
ACTGGGCCAAGGGCCACTACACCGAGGGCGC-CGAGCTCGTCG-
ACCAGGTCCTCGACGTCGTCCGCCGCGAGGCTGAGGGCTGCGACTGCCTTCAGGGCTTCCAGATCACCCAC
TCTCTCGGCGGTGGTACCGGTGCCGGTATGGGCACCCTGCTGATCTCCAAGATCCGCGAGGAATTCCCAGA
CCGCATGATGGCCACCTTCTCCGTCGTCCCCTCCCCCAAGGTCTCCGACACCGTCGTCGAGCCTTACAACGCC
ACCCTCTCCGTCCACCAGCTGGTCGAGAACTCTGACGAGACTTTCTGTATCGATAACGAGGCCCTGTACGAC
ATTTGCATGCGCACT---

>D_guizhouensis_GMBC0065

-------------TGTGTTGACACTAT-------GGGGA---------------------------CGG--TAACGCTGACAGACG-----------
GGT-CGGT-
GGACAGTTACAATGGCACCTCGGAGCTCCAGCTTGAGCGCATGAGCGTCTACTTTAACGAGGTACGTGGT-
TGCGG-----------------C----------G----ATG-----T--GGAG--------------------GGATGC-CA-
TCGGACGAAGGACGAG-GA-C-----GCTAA--CGT-GC-CCC---GGGTG-
ACAGGCCTCTGGTAACAAGTACGTGCCTCGCGCCGTCCTTGTCGATCTCGAGCCCGGCACCATGGACGCCG
TCCGTGCTGGCCCCTTCGGCCAGCTCTTCCGCCCCGACAACTTCGTCTTCGGCCAGTCGGGTGCCGGCAACA
ACTGGGCCAAGGGTCACTACACGGAGGGCGC-CGAGCTCGTCG-

ACCAGGTCCTTGACGTCGTCCGCCGCGAGGCTGAGGGCTGCGACTGCCTTCAGGGCTTCCAGATCACCCAC
TCTCTCGGCGGCGGTACCGGTGCCGGTATGGGCACCCTGCTGATCTCCAAGATCCGCGAGGAGTTCCCAGA
CCGCATGATGGCTACCTTCTCCGTCGTCCCCTCCCCCAAGGTCTCCGACACCGTCGTCGAGCCTTACAACGCC
ACCCTCTCCGTCCACCAGCTGGTCGAGAACTCTGACGAGACTTTCTGTATCGATAACGAGGCCCTGTATGAC
ATTTGCATGCGCACTCTGAAGCTGTCGAACCCCTCGTATGGTGATCTCAACCACCTCGTCTCTGCCGTCATGT
CCGGTGTCACCACTTGCCTGCGCTTCCCCGGTCAGCTAAACTCTGACCTGCGCAAGCTTGCCGTGAACATGG
TGCCCTTCCCTCGTCTACACTTCTTCATGGTCGGCTTCGCACCCCTGACCAGCCGCGGTGCCCACTCTTTCCG
CGCTGTCACCGTTCCCGAGTTGACCCA-GCAGAT-
GTTTGACCCCAAGAACATGATGGCTGCTTCGGATTTCCGCAACGGTCGATATCTAACGTGCTCTGCCATCTT
GTAAGCCTTG-TG----TTCACTC------------TCCTTCCTCTC-ACCCC--------------CCAACAAAC-CCA---
ACTGCTAACCCT---AATCAAACTAGCCGTGGCAAGGTCTCGATGAAGGAGGTCGAGGACCA------------------
----

>D_crateriformis_GMBC0205

------------ATGTATCAACACTGC-------GGGAA---------------------------CGG--TAACGCTGATAGATG-----------
GGT-TGGT-
GGATAGTTACAATGGCACCTCGGAGCTCCAGCTCGAGCGCATGAGCGTCTACTTCAATGAGGTAAGTGGT-
TGCGG-----------------C----------G----ATG-----T--GGAC--------------------GGATGC-CA-TCAGACGA------AG-
GA-C-----GCTAA--CGT-GC-CGC---CGATG-
ATAGGCCTCTGGTAACAAGTACGTGCCTCGCGCCGTTCTTGTCGATCTCGAGCCCGGCACCATGGACGCCGT
CCGTGCTGGCCCCTTCGGCCAACTCTTCCGCCCCGACAACTTCGTCTTCGGCCAGTCGGGTGCCGGCAACAA
CTGGGCCAAGGGCCACTACACGGAGGGCGC-TGAGCTCGTCG-
ACCAGGTCCTCGACGTCGTCCGCCGCGAGGCTGAGGGCTGCGACTGCCTCCAGGGCTTCCAGATCACCCAC
TCTCTCGGCGGTGGTACCGGTGCCGGTATGGGTACCCTGCTGATCTCCAAGATCCGTGAGGAGTTCCCAGA
CCGCATGATGGCTACCTTCTCCGTCGTCCCCTCCCCCAAGGTCTCCGACACCGTCGTCGAGCCTTACAATGCT
ACCCTCTCCGTCCACCAGCTGGTCGAGAACTCTGACGAGACTTTCTGTATCGATAACGAGGCCCTGTACGAC
ATTTGCATGCGCACTCTGAAGCTGTCGAACCCCTCGTATGGTGATCTCAACCACCTCGTCTCCGCCGTCATGT
CTGGTGTCACCACTTGCCTGCGCTTCCCCGGTCAGCTAAACTCTGATCTGCGCAAGCTTGCCGTGAACATGG
TGCCCTTCCCTCGTCTACACTTCTTCATGGTCGGCTTCGCACCCCTGACCAGCCGCGGCGCCCACTCTTTCCG
CGCCGTCACCGTTCCCGAGTTGACCCA-GCAGAT-
GTTTGACCCCAAGAACATGATGGCTGCCTCGGATTTCCGAAACGGTCGATATCTAACGTGCTCTGCCATCTT
GTAAGCCTTA-TG----TTCACTC------------ACCTTCCTCTC-ACCCC--------------CCAACAAAC-CCA---
ACTGCTAACCCC---GATCAAACTAGCCGTGGCAAG---

>D_comedens_YMJ_90071615

-AACATGCGTGAGATTGTAAGTCATGACAGAA---------------------------GAGCCCCTTTTGCCTCTC-----------
GACTCCCTCCTCGCCCTGCC-CGAC-----------------ACCCCCTCATCCCCCAGC-----AAGTA-CCCAGA------------
--------------------C--GCGAC--GCCCCT-CCAC--------------ACACGCTCTGT----CACGCAC---------ACA------------
--TGA--CACGAGCACCA-------------------CCCCATCC------CGTGG----CC-----
CTC---GACC---GCGT------CGGGCTGTCAACAACCCG-----------------A---CCC------G--A-----------------------
CAGGATGGA--------------AAATGCAAAGCTAACCCGTG---TC---TCGCTCT------------
CTCGATAGGTTCACCTCCAGACCGGCCAATGCGTAAGTGCCTCCCAAC-CGGCA-----G-----------A--
ACACCCCCCATC-----------------GCGCTA----------------GACT-CTGGCGT-----CGCTCACACGA---G-ATT-----
CG-CAGGGCAACCAAATTGGTGCTGCTTTCT---
--GGCAGACCATCTCGGGCGAGCACGGTCTCGACAGCAATGGCGTGTACGTATATCC--------------------
AAGCCCGGCG----------------ACGGGTCGACGCTGC-------GGGGA--------------------------CGG--
TAACGCTGACGGACT-----------GGC-CGGC-
GGATAGCTACAATGGCACCTCGGAGCTCCAGCTCGAGCGTATGAGCGTCTACTTTAACGAGGTATGTCGT-
TGCGG-----------------C----------C----GCG-----T--AGAT--------------------GGATGC-CA-TCACACGAGAGAT----
---------GTTGA--CGT-GC-CGC---CCTTG-
ACAGGCCTCTGGTAACAAGTACGTGCCTCGCGCCGTGCTGGTCGATCTCGAGCCTGGCACCATGGACGCCG
TCCGTGCTGGCCCCTTCGGTCAGCTCTTCCGGCCCGACAACTTCGTCTTCGGCCAGTCGGGCGCCGGCAACA
ACTGGGCCAAGGGTCACTACACGGAGGGCGC-TGAGCTCGTCG-
ACCAGGTCCTCGACGTCGTCCGCCGCGAGGCTGAGGGCTGCGACTGCCTCCAGGGCTTCCAGATCACCCAC
TCCCTCGGCGGTGGTACCGGTGCCGGTATGGGTACCCTGCTGATTTCCAAGATCCGCGAGGAGTTCCCGGA
CCGCATGATGGCCACCTTCTCCGTCGTCCCCTCCCCCAAGGTCTCCGACACCGTTGTCGAGCCTTACAATGCT
ACCCTCTCCGTCCACCAGCTGGTCGAGAACTCTGACGAGACTTTCTGTATCGATAACGAGGCCCTGTACGAC
ATTTGCATGCGCACTTTGAAGCTGTCGAACCCCTCGTATGGCGATCTCAACCACCTCGTCTCCGCTGTTATGT
CCGGCGTCACTACCTGCCTGCGCTTCCCCGGTCAGCTGAACTCTGACTTGCGCAAGCTTGCCGTGAACATGG
TGCCCTTCCCTCGTCTGCACTTCTTCATGGTCGGGTTTGCACCCCTGACCAGCCGCGGTGCTCACTCTTTCCG
CGCCGTCACCGTTCCCGAGTTGACCCA-GCAGAT-
GTTCGACCCCAAGAACATGATGGCTGCTTCGGATTTCCGCAACGGTCGATATCTGACGTGTTCTGCCATCTT
GTAAGCCTCG-TG----TTCACTC------------TCCTTCCTCTCAAACCC-------------CCTGTGGGC-CCG---
GGTGCTAACCTC----
AATCAACTAGCCGTGGCAAGGTCTCGATGAAGGAGGTCGAGGACCAGATGCGCAACGTTCAGAACAAGAA
CTCGTCCTACTTCGTCGAATGGATTCCCAACAACATCCAGA

>H_papillatum_ATCC_58729

TCTCACCCGGCCCGGGTACAACCAGACCCCCTGA-----ACGCG-TCCG-------------AAAAGA--------------------
--------------------T------T-GATGCCCCTC----CACACGC---------ATA--
CACACCAAA--CAATAGCGCA-------------------------TCC------TGGAG----TT-----CTC---TACA---TCCA------
TCCACCGTCAAC-CTC-----TA------------C---CAC------A--A-----------------------CACGAT---------------
GGAAAACTGAAGCTAA-CCGTG---TC---TCTTCAT------------
CTCTACAGGTCCATCTCCAGACCGGCCAATGCGTAAGTACCACAAC---CACC----------------------
GAACAGCTATC-----------------GCGCTG----------------GGAC-ATTGCGG-----GGCTCACATGA-----AAT-----
CG-CAGGGTAACCAAATTGGTGCTGCCTTCTGGTATGTGC---
CTACTCGCCTATCTCGAGGGCATAGAGGCGGCTGTCAACAT--GAATATTAACCT---
CGCTCCAGGCAAACCATCTCCGGTGAGCATGGTCTCGACAGCAATGGCGTGTAAGTATCTT------------------

---TCGACCCC-------------------CGATTCAGCCCAT-------GGGAA---------------------------TGA--TAAT-------
TGAT-----------CAT-CTGT-
AAATAGCTACAACGGAACCTCCGAGCTCCAGCTCGAGCGCATGAGCGTCTACTTCAACGAGGTATGCGGG-
CATGG-----------------T----------A----ATGG-------GAAT--------------------AGATGC-AA-G--------GGATGAG-
GT-A-----CTAAT----C----TAT---TAATA-
ATAGGCTTCCGGCAACAAGTATGTTCCTCGCGCTGTCCTCGTCGATCTTGAGCCCGGTACCATGGATGCCGT
CCGTGCTGGTCCCTTCGGCCAGCTCTTCCGACCTGACAACTTCGTCTTCGGCCAGTCCGGTGCTGGAAACAA
CTGGGCCAAGGGTCACTACACCGAGGGCGC-CGAGCTTGTCG-
ACAACGTTCTCGATGTCGTCCGTCGCGAGGCTGAGGGTTGCGATTGCCTCCAGGGTTTCCAGATCACCCACT
CTCTCGGTGGTGGTACCGGTGCCGGTATGGGTACCCTGCTGATCTCTAAGATCCGTGAGGAGTTCCCCGAC
CGCATGATGGCTACTTTCTCCGTCGTGCCCTCCCCCAAGGTTTCCGACACCGTCGTCGAGCCTTACAATGCCA
CCCTCTCCGTCCACCAGCTGGTCGAGAACTCTGACGAGACTTTCTGCATTGACAACGAGGCCCTTTACGACA
TCTGCATGCGCACTCTGAAGTTGTCCAACCCCTCGTACGGCGATCTCAACCACCTGGTCTCCGCTGTCATGTC
CGGCGTCACTACTTGCCTACGTTTCCCGGGCCAGCTGAACTCTGACCTGCGCAAGCTCGCCGTGAACATGGT
GCCTTTCCCCCGTCTACACTTCTTCATGGTCGGATTCGCTCCTCTGACCAGCCGTGGTGCTCACTCCTTCCGC
GCTGTCACTGTTCCCGAGTTGACTCA-GCAGAT-
GTTCGACCCCAAGAACATGATGGCTGCCTCTGATTTCCGCAATGGCCGCTACCTCACATGCTCTGCCATCTTG
TAAGCTTTC-CC----CCCCTTTCTTTTT--GA-GTCTCT-------------------------ATCCTTGAC-GAT---
CTTGCTGACTTA----
ATTAATCTAGCCGTGGCAAGGTTTCCATGAAGGAGGTTGAGGACCAGATGCGCAATGTCAGAAC------------
----

>H_sp_MUCL_51392

AATTGAAGCTAA-CCGTG---TC---TCTTCAT------------
CGCTATAGGTTCACCTCCAGACCGGCCAATGCGTAAGTACCATAAC---CACC----------------------
AAACAACTATC-----------------GCGCTG----------------GGAC-ATCGCGG-----GGCTCACATAA-----AAT-----CG-
CAGGGTAACCAAATTGGTGCCGCCTTCTGGTATGTGCCTGCTACTCGTCTAACTCAAGGACGTAGAGACGA
CTGTCAACAT--AAATATTAACCT---
CGCTCCAGGCAAACCATCTCTGGCGAGCACGGTCTCGACAGCAATGGCGTGTATGTATTT--------------------
--TTCGCCTC-------------------CTGTCCAGCCCAT-------AAGAG--------------------------CAG--CAAC-------
TGAT-----------CAT-CCGT-
AAATAGCTACAATGGAACCTCCGAGCTCCAGCTCGAGCGCATGAGCGTCTACTTCAACGAGGTATGCGGA-
CACAG-----------------G----------A----ATGG-------GAAT--------------------AGATGC-AG-G--------GGATGAG-
GT-G-----CTAAT--TCT-CC-TAT---TAATA-
ATAGGCTTCCGGCAACAAGTACGTTCCTCGCGCCGTTCTCGTCGATCTTGAGCCCGGTACCATGGATGCCGT
CCGTGCTGGTCCCTTCGGCCAGCTCTTCCGACCTGACAACTTCGTCTTCGGCCAGTCCGGTGCTGGCAACAA
CTGGGCCAAGGGTCACTACACCGAGGGTGC-CGAGCTTGTCG-
ATAACGTTCTCGATGTCGTCCGTCGCGAGGCTGAGGGCTGCGATTGCCTCCAGGGTTTCCAGATCACCCACT
CCCTCGGTGGTGGTACCGGTGCCGGTATGGGTACTCTGCTGATCTCCAAGATCCGTGAGGAGTTCCCTGAC
CGCATGATGGCTACTTTCTCCGTCGTCCCCTCCCCCAAGGTTTCCGACACCGTTGTCGAGCCTTACAATGCCA

CTCTCTCTGTCCACCAGCTGGTCGAGAACTCTGACGAGACTTTCTGCATTGACAACGAGGCCCTGTACGACA
TCTGCATGCGCACTCTGAAGCTGTCCAACCCCTCATATGGCGATCTCAACCACCTGGTCTCTGCTGTCATGTC
CGGTGTCACTACTTGTCTGCGTTTCCCGGGCCAGCTGAACTCCGACCTGCGCAAGCTTGCCGTGAACATGGT
GCCCTTCCCCCGTCTGCACTTCTTCATGGTCGGATTCGCTCCTCTGACCAGCCGTGGCGCCCACTCCTTCCGC
GCCGTTACTGTTCCCGAGTTGACTCA-GCAGAT-
GTTCGACCCCAAGAACATGATGGCTGCCTCTGATTTCCGTAATGGCCGCTACCTCACATGCTCTGCCATCTTG
TAAGTTTTT-TC----TCCATTCTCTTTG--AATCTCTCT--------------------------ATCCTCGAC-AGT---
CTTACTGACTTA----ATTAATCTAG---

>H_baruense_UCH9545

------------GGATGTAAGT------------------AC------------------AATTTTCGCTTATGTTTACCAAGGAACCC---------
----------------ATGGTCCCTTTTTGGGGGGAATAAAAAACCCCTGA-----ACGCG-AATC--------------TAACA-----
--------TAC---AAAT---ACCCC-CTGA------T------T-TTCACCCCT-----CACGCAC---------ACAATCGTCA----------
AAACCCCGAAAAAA---------------ACATAGCAAG--CAACACAGCA-------------------------TCCT-----CATAG----
TC--------A-TGATA---TTGCTATGAGACTATCGTCAAA-TCC-----CT------------CA--AACACGT--A--A------------
------------CATTAT--------------GGAGAAGTGGAAGCTAA-TATCA---TT-ATTTTTTAT------------
CTTAATAGGTTCATCTTCAGACCGGCCAATGCGTAAGTAGTAGCCAG--CTACA-----CCAT-ATCCAAC----
AAACAACGAAA-----------------TCGACAC-------------GAGAAT-ATGGTGG-----TACTAAC--TA---A-GAT-----
CG-TAGGGTAACCAAATTGGTGCTGCTTTCT---
-GGCAAACCATCTCTAGCGAGCACGGTCTTGACAGCAATGGAGTGTATGTATTTC----------------------
TATTCGCCATCCTGATTTTTACTTGATTATTTCTTATGTTTGTTT-TGATGAT-----------------------ATTCGG--
TAAC-------TAAC-----------AGC-CAAT-
CAATAGTTACAATGGAACTTCTGAGCTCCAGCTCGAGCGTATGAGCGTCTACTTTAACGAGGTACACAGT-
CTTAG-----------------A----------A---ACC-----CC-AATT--------------------AGAAGA-GC-A--------AGAATCG-TT-
A-----CTAAT--CAC-CC-TAA---CATGC-
ACAGGCATCTGGTAACAAGTATGTCCCCCGCGCCGTCCTCGTCGATCTCGAACCCGGTACCATGGATGCCGT
CCGCGCTGGTCCTTTCGGTCAACTCTTCCGTCCCGACAACTTCGTCTTCGGCCAATCCGGTGCCGGAAACAA
CTGGGCCAAGGGTCATTACACTGAAGGTGC-TGAGCTTGTCG-
ACAACGTTCTCGATGTCGTTCGTCGTGAGGCTGAGGGCTGTGACTGCCTCCAGGGTTTCCAAATTACCCACT
CTCTTGGTGGTGGTACCGGTGCTGGTATGGGTACCTTGTTGATCTCCAAGATCCGTGAAGAGTTCCCTGACC
GTATGATGTCCACTTACTCCGTCGTTCCTTCTCCCAAGGTCTCCGATACTGTTGTTGAGCCTTACAATGCTACT
CTCTCCGTTCACCAGCTGGTCGAGAACTCCGACGAGACCTTCTGTATTGATAACGAGGCCTTGTACGATATT
TGCATGCGCACTCTCAAGCTATCTAACCCTTCTTATGGTGACCTGAACCACCTTGTCTCCGCCGTCATGTCTG
GCGTTACCACTTGCTTGCGTTTCCCTGGTCAGCTGAACTCTGATCTACGCAAGCTTGCCGTCAACATGGTTCC
TTTCCCTCGTCTCCATTTCTTCATGGTTGGCTTCGCTCCTCTAACCAGCCGTGGTGCCTATACCTTCCGTGCTG
TCACCGTTCCCGAGTTGACTCA-GCAAAT-
GTTCGACCCCAAGAACATGATGGCTGCTTCTGACTTCCGTAATGGTCGTTACCTGACGTGCTCTGCCATCTTG
TAAGATATC-CC----TTTCCAAC-----------TCACT--------------------------TTTTTGTAT-ATA---
TCTGCTAACTACCCCCCTTTTTTTAGCCGTGGTAAGGTCTCCATGAAGGAGGTTGAGGACCAAATGCGCAAC
GTCCAGAACAAGAACTCGTCCTACTTCGTTGAGTGGATTCCCA------------

>H_fragiforme_MUCL_51264

ATATTCCCCCTCAAAAAAAGAACAGCCACCCCTGA-----ACGCG-TC------------------------------------CAAC-
--GTCCC-CTGA------T------T-TTCGCCCCT-----CACGCAC---------AAA--
------ATA--AACCACCTCA-------------------------TTC------TACAA----TC-----GCC---GATG---TTGA------
ACTGTTGTCAAA-ACA-----CG------------C---AAC------A--ACAACAATAATAACAACAAATTGAAAATTAT------
---------GCAGAGTTGAAGCTAA-TCGCG---TC-TTTTTTTAT------------
CTCAATAGGTTCATCTTCAGACCGGCCAGTGCGTAAGTAACCACCAC--TACC-----------GATCAAC----
AAATATCGAAT-----------------TTCAGTG-------------AGGAAC-TTGGCGG-----GGCTCACAACA---G-TAT-----
TG-TAGGGTAACCAAATCGGTGCTGCTTTCT---
-GGCAAACTATCTCCAGCGAGCACGGTCTTGACAGCAATGGCGTGTACGTATTTT---------------------
AATTCGCC-------------------AATTCCCCAAAAA-------CGCGATATA------------CACATGTGTTCTCT--GTAC-----
--TGAC-----------CGC-CAAT-
AAATAGCTACAATGGCACTTCAGAGCTCCAGCTCGAGCGCATGAGCGTCTACTTTAATGAGGTACACAGC-
CGAAG-----------------A----------G----CCC-----CC-AATG--------------------ATGTGG-TT-A--------GGAATAG-
TT-A-----CTAAT--CAC-CC-CAA---CATGC-
ACAGGCATCTGGTAACAAGTATGTCCCCCGCGCCGTCCTCGTCGATCTCGAACCCGGTACCATGGACGCCGT
CCGTGCTGGTCCCTTCGGCCAACTCTTCCGACCCGACAACTTCGTTTTCGGCCAATCCGGTGCCGGAAACAA
CTGGGCCAAGGGTCATTACACTGAGGGTGC-TGAGCTAGTCG-
ACCAGGTTCTCGATGTCGTTCGTCGTGAGGCTGAGGGCTGTGACTGCCTTCAGGGTTTCCAGATCACCCACT
CCCTCGGTGGTGGTACCGGTGCCGGTATGGGTACTTTGTTGATCTCCAAGATCCGCGAGGAGTTCCCCGAC
CGCATGATGTCCACCTACTCCGTTGTTCCCTCTCCCAAGGTCTCTGACACCGTCGTTGAACCTTACAACGCCA
CTCTCTCCGTCCATCAGCTGGTCGAGAACTCAGACGAGACCTTCTGCATTGATAACGAGGCCTTGTACGACA
TTTGCATGCGCACTCTCAAGTTGTCCAACCCTTCGTATGGCGACCTGAACCACCTGGTCTCTGCCGTCATGTC
CGGTGTTACTACTTGCCTGCGTTTCCCCGGCCAGTTGAACTCTGACCTACGCAAGCTAGCCGTCAACATGGT
TCCCTTCCCTCGTCTGCATTTCTTCATGGTCGGCTTCGCTCCTCTTACCAGCCGTGGTGCCTACACTTTCCGTG
CTGTCACCGTTCCCGAGTTGACTCA-GCAGAT-
GTTCGACCCAAAGAACATGATGGCCGCCTCTGACTTCCGCAACGGTCGTTACTTGACGTGCTCTGCCATCTT
GTACGTTTAC-CC----CCCAAACCCC--

>H_howeanum_MUCL_47599

TGATTCAGCCCCCAAGCACCAAGCACCTCCCCCCCAAAAAAGAACAGCCACCCCTGA-----ACGCG-TC-------
------------------------------CAAC---ATCCC-CTGA------T------T-TTCGCCCCT-----CACGCAC---------AAA-------
--ATA--AACCACATCA------------------------TTC------TACAA----TC-----
CCC---TATG---TTGA------ACTGTTGTCAAA-TCA-----CGCAACAACAAAAAC---AAC------A--
ACAACAACAACAACAACAAATTGAATATTAT--------------GGAGAGTTGAAGCTAA-TCGCG---TC----
TTTTAT------------CTAAACAGGTTCATCTTCAGACCGGCCAGTGCGTAAGTAACCACCAC--TACC-----------
GATCAAC----AAATACCGAGC-----------------TTCAGTA-------------AGGAAT-ATAGCGG-----
GGCTCACAACA---G-TAT-----TG-TAGGGTAACCAAATCGGTGCTGCTTTCT--------------------------------------
----

GGCAAACTATCTCCAGCGAGCACGGTCTCGACAGCAATGGCGTGTACGTATTTT---------------------
AATTCGCC-------------------AATTCCAGAAGAG-------GAAAAAAAC------------GCGATATGTTACGT--GTGC--
-----TGAC-----------CGC-CAAT-

AAATAGCTACAATGGCACTTCAGAGCTCCAGCTCGAGCGCATGAGCGTCTACTTTAACGAGGTACACAGC-
CGAAG-----------------A----------G----CCC-----CC-AATG--------------------ATATGG-TT-A--------GGAATAG-
TT-A-----CTAAT--CAC-CC-CAA---CATGC-
ACAGGCATCTGGTAACAAGTATGTCCCCCGCGCCGTCCTCGTCGATCTCGAACCCGGTACCATGGACGCCGT
CCGTGCTGGTCCCTTCGGCCAACTCTTCCGACCCGACAACTTCGTTTTCGGCCAATCCGGTGCCGGCAACAA
CTGGGCCAAGGGTCATTACACTGAGGGTGC-TGAGCTTGTCG-
ACCAGGTTCTCGATGTCGTTCGTCGTGAGGCTGAGGGCTGTGACTGCCTTCAGGGTTTCCAGATCACCCACT
CCCTCGGTGGTGGTACCGGTGCTGGTATGGGTACCTTGTTGATCTCCAAGATCCGCGAGGAGTTCCCTGAC
CGCATGATGTCCACCTACTCCGTTGTTCCCTCCCCCAAGGTCTCCGACACCGTCGTTGAACCTTACAACGCCA
CTCTCTCCGTCCACCAGCTGGTCGAGAACTCAGATGAGACCTTCTGCATTGACAACGAGGCCTTGTACGACA
TTTGCATGCGCACTCTAAAGTTGTCTAACCCTTCGTACGGTGACCTGAACCACCTGGTCTCTGCCGTCATGTC
TGGTGTTACTACTTGCCTACGTTTCCCCGGCCAGTTGAACTCTGACCTACGCAAGCTAGCCGTCAACATGGTT
CCCTTCCCTCGTCTCCATTTCTTCATGGTCGGCTTCGCTCCTCTTACCAGCCGTGGTGCCTACACCTTCCGTGC
TGTCACCGTTCCCGAGTTGACTCA-GCAGAT-
GTTCGACCCCAAGAACATGATGGCCGCCTCTGACTTCCGCAACGGTCGTTACTTGACGTGCTCTGCCATCTT
GTACGTTTAT-TC----CCCAAACCCCTAACCAATTAGATT--------------------------GGTCCCAAT-TCG---
GTTGCTAACTATT--
ACTGCTTCTAGCCGTGGCAAGGTCTCCATGAAGGAGGTTGAGGACCAGATGCGCAACGTCAGAA------------
----

>H_ticinense_CBS_115271

-AACATGCGTGAGATTGTAAGTCGATATAATAT---------A----------TTCCATTTCTCCTTCATCGTGTTTACTCA-
---------TGATTCCGCCCCCAA-------CTATCTATCTCCCCAACTTAAAAAAAAAAACCCTGA-----ACGCG-TC-------
----------------------------------CAAC---GTCCC-CTGA------T------T-TTCGCCCCT-----CACGCAC---------AAA------
--ATC---AAC---CTTC-
--TTGT------GCTATCGTCAAG-TCA-----CG------------C---AAC------A--A------------------------CGTTAT----------
-----AGAAAGATGAAGCTAA-TCGCG---TC---TTTTTTA------------
TCGTATAGGTTCACCTTCAGACCGGCCAGTGCGTAAGTAATCACCGC--TACC-----------GATCAAC----
AAACATCGAAT-----------------CTCAGCG-------------TGGAAC-ATGGCGG-----GGCTCACAGCA---A-TAT-----
TG-TAGGGTAACCAAATCGGTGCTGCTTTCT---
-GGCAAACCATCTCCAGCGAGCACGGTCTCGACAGCAACGGCGTGTATGTATTTT----------------------
TATTCGTC-------------------AATTTAGCGCAGT-------GATTT---------------------------CCC--CTGC-------TAAC---
--------CGC-CAAT-
AAACAGCTACAATGGCACTTCGGAGCTCCAGCTCGAGCGCATGAGCGTCTACTTTAACGAGGTACACAGC-
CGAAG-----------------A----------G----CCC-----CT-GATG-------------------ATATCG-TT-A--------GGAATAG-
TT-A-----CTAAT--CAC-CC-CAA---CATGC-
ACAGGCATCTGGTAACAAGTATGTCCCCCGCGCCGTCCTCGTCGATCTCGAACCCGGAACCATGGACGCCG
TCCGTGCTGGTCCCTTTGGCCAGCTCTTCCGACCCGACAACTTCGTTTTCGGCCAATCCGGTGCCGGCAACA
ACTGGGCCAAGGGTCACTATACGGAGGGTGC-TGAGCTTGTCG-
ACCAAGTTTTGGATGTTGTTCGTCGTGAGGCAGAAGGCTGTGACTGCCTCCAGGGTTTCCAGATCACCCACT
CCCTTGGTGGTGGTACCGGTGCCGGTATGGGTACTCTGTTGATCTCCAAGATCCGTGAGGAGTTCCCCGAC
CGCATGATGTCCACATACTCCGTCGTTCCCTCTCCTAAGGTCTCCGACACCGTCGTTGAACCTTACAATGCCA
CTCTCTCCGTCCACCAGCTGGTCGAGAATTCGGACGAGACCTTCTGCATTGACAACGAGGCCCTATACGACA
TCTGCATGCGTACTCTTAAGCTGTCGAACCCTTCATACGGTGACCTGAACCACCTGGTCTCTGCCGTCATGTC

TGGCGTCACCACTTGTCTGCGTTTCCCCGGCCAACTGAACTCTGACCTGCGCAAGCTTGCCGTCAACATGGT
TCCTTTCCCTCGTCTTCACTTCTTCATGGTCGGCTTCGCTCCCCTGACCAGCCGTGGTGCCTACACCTTCCGTG
CTGTTACTGTCCCCGAGTTGACTCA-GCAGAT-
GTTCGACCCCAAGAACATGATGGCCGCCTCTGACTTCCGTAACGGCCGTTACCTGACATGCTCTGCCATCTT
GTAAGATTAC-CT----CTCGTCCGTTT---CGGT-TTGTTTT----------------------ATTTCGTGCC-TTG---
TTCGCTAATTCTT--
TTTGCTTTTAGCCGCGGCAAGGTCTCCATGAAGGAGGTTGAGGACCAGATGCGCAATGTCCAGAACAAGAA
CTCGTCATACTTCGTCGAGTGGATTCCCAACAACATCCAGA

>H_rickii_MUCL_53309

--TCAAT--
------AAG---
------------ATGG------GCTATCGCAAAT-CAA-----GG------------C---AAT------A--A-----------------------
CAACAT----------------AGGAATTGGAGCTAA-TCATG---TC---TGTTTAC-----------
ATCTCTAGGTTCACCTTCAGACCGGCCAGTGCGTAAGTAAACACTAC----------------GAACGAT----
GGATCACGAAT-----------------TCGAGTA-------------AGGAGT-ATGGCGG-----GGCTCAC--TA---A-GAT-----
GA-TAGGGTAACCAAATCGGTGCTGCTTTCT--
--GGCAGACCATCTCCAGCGAGCACGGTCTCGACAGCAATGGCGTGTAGGTGCTCT--------------------
CATTCGTC-------------------AATTCCTTGCTAT-------AACATTGC-------------CGTGATGTTCGGT--GGGC------
-TAAC-----------AGC-CAAT-
GAACAGCTACAACGGGACCTCGGAGCTCCAGCTCGAGCGCATGAGCGTCTACTTTAACGAGGTACACAGT-
CAAAG-----------------A----------A----ACC-----AA-CCAT----------------GTTTTCTAGT-TT-A--------GGAGTGG-
TT-A-----CTAAT--CAC-CC-CAA---CATGC-
ACAGGCATCGGGTAACAAGTACGTCCCCCGCGCCGTCCTCGTCGATCTCGAACCCGGTACCATGGACGCCG
TCCGCGCTGGCCCCTTCGGTCAGCTCTTCCGACCCGACAACTTCGTCTTCGGCCAATCCGGTGCCGGCAACA
ACTGGGCCAAGGGTCATTACACTGAGGGCGC-TGAGCTCGTTG-
ACAACGTTCTTGACGTCGTCCGCCGCGAGGCTGAGGGCTGCGACTGCCTCCAGGGCTTCCAGATTACCCAC
TCTCTCGGTGGTGGTACCGGTGCCGGTATGGGTACCCTGCTGATCTCCAAGATCCGCGAGGAGTTCCCTGA
CCGCATGATGTCTACCTACTCCGTCGTTCCCTCCCCCAAGGTCTCTGACACCGTCGTTGAGCCCTACAATGCC
ACCCTCTCCGTCCACCAGCTGGTCGAGAACTCGGACGAGACCTTCTGCATTGACAACGAGGCCTTGTACGAC
ATCTGCATGCGTACCCTCAAGCTATCCAACCCCTCGTACGGTGACCTGAACCACCTGGTCTCCGCCGTCATGT
CCGGTGTTACCACCTGCTTGCGTTTCCCTGGCCAGCTGAACTCTGATCTGCGCAAGCTTGCCGTCAACATGG
TTCCTTTCCCTCGTCTGCACTTCTTCATGGTTGGATTTGCTCCTCTGACCAGCCGTGGTGCCTACACCTTCCGT
GCTGTCACCGTCCCCGAGTTGACCCA-GCAGAT-
GTTCGATCCCAAGAACATGATGGCCGCTTCTGACTTCCGTAACGGTCGCTACTTGACGTGCTCTGCCATCTT
GTAAGATATT-AT----CTCCGTGA-----------TCGTTGA-----------------------ATTTTCTGGT-GGG---
CTTGCTAACTGT----ATATTCCTAGCCGTGGCAAGGTCTCGA--

>H_subticinense_MUCL_53752

--CTCAAAT-------------CCN---NAAC---ACCCC-CTGA------T------T-
TTCACCNCT-----CACGCAC---------AAA---------------------------AAT---------------TTACTAGAGA--

CACCACAACA-------------------------TCC------TGATGA---------ATTT---CATGG-TTTGG------
CACATCGCCAAA-CCA-----CGA---------------AGC------A--G-----------------------CAGTTT------------
GGAAAAAGTGGAAGCTAA-CCACC---TC-TATTTTGAT------------
ATCAATAGGTTCACCTTCAGACCGGCCAGTGCGTAAGTAAACACCAC--AATCGA----CCAC-AATCGAT----
CAACAACGAAC-----------------TCGAGGA-------------AGGAAC-TCGGCGG-----GGCTCAC--CA---A-GGT-----
GA-TAGGGTAACCAAATTGGTGCTGCTTTCT--
--GGCAGACCATCTCCAGCGAGCACGGTCTCGACAGCAATGGCGTGTACGTATTTT---------------------
TATTCGCC-------------------AATTTATGATAAA-------CAGCA-----------------------TTCGGT--GAGC-------
TAAC-----------GAC-CAAT-
GAACAGCTACAATGGAACTTCGGAGCTCCAGCTTGAGCGCATGAGCGTCTACTTCAACGAGGTACACAGT-
CAAGG-----------------A----------A----GCC-----CA-GCAT----------------AGTCCCTTTT-TC-A--------GGAGTGG-
TT-A-----CTAAT--CAC-CC-CAA---CATGC-
ACAGGCATCCGGTAACAAGTACGTCCCCCGCGCCGTCCTCGTCGATCTCGAACCCGGTACCATGGACGCCG
TTCGCGCTGGTCCCTTCGGTCAGCTCTTCCGACCCGACAACTTCGTCTTTGGTCAATCCGGTGCCGGAAACA
ACTGGGCCAAGGGTCACTACACTGAGGGTGC-TGAGCTTGTCG-
ACAACGTTCTCGATGTCGTCCGCCGTGAGGCTGAGGGCTGTGACTGTCTCCAGGGTTTCCAGATTACCCACT
CTCTCGGTGGTGGTACCGGTGCCGGTATGGGTACCCTGCTGATCTCCAAGATCCGCGAGGAGTTCCCCGAC
CGCATGATGTCCACCTACTCCGTCGTTCCCTCTCCCAAGGTCTCCGACACCGTCGTTGAGCCTTACAATGCCA
CTCTCTCCGTCCACCAGCTGGTCGAGAACTCGGACGAGACCTTCTGCATTGACAACGAGGCCTTGTACGACA
TCTGCATGCGCACTCTCAAGCTATCCAACCCCTCGTACGGTGACCTGAACCACCTGGTCTCTGCCGTCATGTC
CGGTGTCACCACTTGCTTGCGTTTCCCTGGCCAGCTGAACTCTGACCTGCGCAAGCTTGCCGTCAACATGGT
TCCCTTCCCCCGTCTGCACTTCTTCATGGTCGCGTTCGCTCCTCTGACCAGTCGCGGTGCTTACACCTTCCGTG
CCGTCACCGTTCCCGAGTTGACTCACGCAGATNGTTCGACCCCAAGAACATGATGGCTGCTTCTGACTTCCG
TAACGGTCGCTACCTGACGTGCTCTGCCACCTTGTAAGAT---

>H_invadens_MUCL_51475

CACAAAAAAAAGCCCCCCCTGAACCCAACGCG-TCCA---------CAAGGGAAAAGAG------------CCC---CAAA--
TCCCCT-TGAT------T------T-ACTGCCCCT-----CATACAC---------TCA--
----AGA--CATGATTATA-------------------------TCC------CCCAAG---TT-----ATC---AACA---TTAC---
GATGATATCCTCGAA-TAG-----AA------------C---TGT------A--A-----------------------TACGAT------------
GGAAGATAAGAAAGCTGA-CCATA---------TTTCAT------------
CTCAATAGGTTCACCTCCAGACCGGCCAATGTGTAAGTAACAACAG------------------GACTACC----
GACGAGATATC-----------------GCGTCT----------------GGTT-ATAGCGAGAGGGTGCTCACACGA---A-GAT----
-AA-TAGGGTAACCAAATTGGTGCTGCTTTCT---
--GGCAGACCATCTCCAGCGAGCACGGTCTCGACAGCAATGGCGTGTACGTATTC----------------------
GACTCGTC-------------------AATTCATCGTGCC-------AAGAG-------------------------CAAC--AAAC-------TAAC-
----------GAC-
CAATAAAACAGCTACAATGGAACCTCTGAGCTACAGCTCGAGCGTATGAGCGTCTACTTTAACGAGGTACG
GGGC-ACATA-----------------AC-------------------------CCAT--------------------ATACGG-AA-G--------
AGATCAG-TT-A-----CTAAT--CAC-CC-CAA---CATGC-
ACAGGCATCTGGTAACAAATACGTTCCTCGCGCCGTCCTCGTCGATCTCGAACCCGGTACCATGGACGCCGT

TCGCGCTGGTCCCTTTGGTCAACTTTTCCGTCCCGACAACTTCGTTTTCGGTCAGTCCGGTGCTGGCAACAAC
TGGGCCAAGGGTCACTACACTGAGGGTGC-CGAGTTGGTCG-
ACCAGGTTCTCGATGTCGTTCGTCGCGAGGCTGAGGCTTGCGACTGCCTCCAGGGTTTCCAGATTACTCACT
CCCTCGGTGGTGGTACTGGTGCTGGTATGGGTACCCTGCTGATCTCCAAGATCCGCGAAGAGTTCCCAGAC
CGCATGATGGCCACTTTCTCGGTCGTCCCCTCTCCTAAGGTCTCTGACACTGTCGTTGAGCCTTATAACGCTA
CCTTATCCGTCCACCAGCTGGTCGAAAACTCTGACGAGACCTTTTGCATTGATAACGAGGCTCTCTACGATA
TCTGCATGCGTACTCTCAAGTTATCTAACCCTTCGTATGGTGACCTGAACTACCTGGTCTCTGCTGTCATGTC
CGGCGTCACCACTTGCTTACGATTCCCTGGTCAGCTGAACTCCGACCTGCGCAAGCTCGCCGTGAACATGGT
TCCGTTCCCTCGTCTCCACTTCTTCATGGTTGGATTCGCTCCTCTGACCAGCCGTGGCGCTCACTCTTTCCGTG
CCGTCACCGTTCCCGAATTAACTCA-GCAGAT-
GTTTGACCCCAAGAACATGATGGCCGCCTCTGATTTCCGTAATGGTCGCTACCTAACGTGCTCTGCCATCTTG
TAAGACACT-CT----TTTTTAAA-----------ATGCG-------------------------TATTGCTGT-TGA---
TATACTAATTTG---
ATTCTTTTTAGCCGTGGCAAGGTTACTGGTAAGGAGGTTGAGGACCAGATGCGCAACGTCC-------------------
----

>H_macrocarpum_CBS_119012

--TCCGAACCGAGTA-
CTACCATTTACCACTACACATACACAAAAAGCCCCCTGAACCCAACGCG-TCCA----------AGGAAAAGAG-----
-------CCC---CAAA--CCCCCT-TGGT------T------T-ACTGCCCCT-----CATACAC---------TCA-------------------------
-----------------------------AAA--CATGATTATAT-----------------------ATCC------CCCAAG---TT-----ATC---AACA-
--TTAC---GATGTTATCCTCAAA-TAA-----AA------------C---AAG------A--A------------------------TACGAT--------
-----GGAAGACAAGAAAGCTAA-CCATA---TT---ACTTCAT------------
CTCAATAGGTTCACCTCCAGACCGGCCAATGTGTAAGTAACAACAA------------------GACTACC----
GACGAAATATC-----------------GCGTCT----------------ATTT-ATAGCGAGAGG-GACTTACACGA---A-GAT-----
AA-TAGGGTAACCAAATTGGTGCTGCTTTCT---
-GGCAGACCATCTCCAGCGAGCACGGTCTCGACAGCAATGGCGTGTATGTATTC-----------------------
GACTCGTC-------------------AATTCATCGTGCC-------AAGAG--------------------------CAAC--AAAC-------TAAC-
----------CAC-
CAATAAAACAGCTACAATGGAACGTCCGAGCTACAGCTCGAGCGTATGAGCGTCTACTTTAACGAGGTACG
GGGC-ACATA-----------------AC-------------------------CCAT--------------------ATACGG-AA-G--------
AGATCAG-TT-A-----CTAAT--CAC-CC-CAA---CATGC-
ACAGGCATCTGGTAACAAATACGTTCCTCGCGCCGTCCTCGTCGATCTCGAACCCGGTACCATGGACGCCGT
TCGCGCCGGTCCCTTCGGTCAACTTTTCCGTCCCGACAACTTCGTCTTCGGTCAGTCCGGTGCTGGCAACAAC
TGGGCCAAGGGTCACTACACTGAGGGTGC-CGAGTTGGTCG-
ACCAGGTTCTCGATGTCGTTCGTCGCGAGGCTGAGGCTTGCGATTGCCTCCAGGGTTTCCAGATTACTCACT
CCCTCGGTGGTGGTACTGGTGCTGGTATGGGTACCCTGCTGATCTCCAAGATCCGCGAAGAGTTCCCAGAC
CGTATGATGGCCACTTTCTCGGTTGTCCCCTCTCCTAAGGTCTCCGACACTGTCGTTGAGCCTTATAACGCTA
CCTTATCCGTCCACCAGCTGGTCGAAAACTCTGACGAGACCTTTTGCATTGATAACGAGGCTCTCTACGATA
TCTGCATGCGTACTCTCAAGTTATCTAACCCTTCATWTGGTGACCTGAACTACCTGGTCTCTGCTGTCATGTC
CGGCGTCACCACTTGCTTACGATTCCCTGGTCAGCTGAACTCCGACCTGCGCAAGCTCGCCGTGAACATGGT
TCCGTTCCCTCGTCTCCACTTCTTCATGGTTGGATTCGCTCCTCTAACCAGCCGTGGCGCTCACTCTTTCCGTG
CCGTCACCGTTCCCGAATTAACTCA-GCAGAT-
GTTTGACCCCAAGAACATGATGGCCGCCTCTGACTTCCGTAATGGTCGCTACCTAACGTGCTCTGCCATCTT

GTAAGATACC-CT----TTTCTACA-----------ATACG--------------------------TGTTACTAT-TGA---
TATACTAATTTC---
ATTCTTTTTAGCCGTGGCAAGGTTACTGGTAAGGAGGTTGAGGACCAGATGCGCAACGTCCAGAAC---------
----

>H_trugodes_MUCL_54794

--GTTTACT---------GCTTTATCCCCAACCGAGTG-
CCA----------CCACCCCCGAAAAGGAGCCCCCTTGA-----ACGCG-TCCCAGGAAGAAA-AAAAAAAGAG--------
----CTC---CAAA---CCCCC-TTGA------T------T-TCTGCCCCT-----CACGCAC---------ACG---------------------------
--------------------------AAA--CACGATTGTACAC-------------------------------CCATA----TT-----ATT---TACA---
CCAT------GATACCCTCAAA-TCGTG---TA------------T---GAT------A--T------------------------TTCGAT-----------
----CAAGGATAATAGCTAA-CCATATAATT---TTTTTAT------------
CTAAATAGGTTCACCTCCAGACCGGCCAATGCGTGAGTACAACCAT---AACC----------------------
GATAAAATATC-----------------ATCTCT----------------GGGT-ATAGCGGG----GGAACTCATAA---G-TAT-----
TA-TAGGGTAACCAAATTGGTGCCGCCTTCT---
-GGCAAACCATCTCTGGCGAGCACGGTCTCGACAGCAATGGCGTGTACGTATTC-----------------------
GACTCGCC-------------------AATTCAATATGCC-------AAGGA---------------------------TGC--GAAC-------TAAT-
----------CAG-CAAT-
AAACAGCTACAACGGAACTTCCGAGCTCCAGCTCGAGCGCATGAGCGTCTACTTTAACGAGGTACGGGGC-
TCAAT-----------------G------------------------C-CTAT--------------------ACACGG-AA-G--------GGATCAG-TT-A--
---CTAAT--CAC-CC-CAA---CATGC-
ACAGGCATCTGGTAACAAATACGTTCCTCGCGCCGTCCTCGTCGATCTCGAACCCGGTACCATGGACGCCGT
TCGCGCCGGTCCTTTCGGACAACTTTTCCGCCCCGACAACTTCGTCTTCGGTCAGTCCGGTGCTGGAAACAA
CTGGGCCAAGGGTCACTACACGGAAGGTGC-TGAGCTAGTCG-
ACAACGTTCTTGACGTCGTTCGTCGTGAGGCCGAGGCTTGCGACTGCCTCCAGGGTTTCCAGATCACCCACT
CTCTCGGTGGTGGTACTGGTGCTGGTATGGGTACTCTATTGATCTCCAAGATCCGCGAAGAGTTCCCCGACC
GCATGATGGCTACTTTCTCGGTCGTTCCCTCTCCTAAGGTTTCCGACACCGTCGTTGAGCCTTACAATGCTAC
TCTCTCCGTCCACCAGCTGGTCGAGAACTCCGACGAGACCTTCTGCATTGACAACGAGGCTCTCTACGACAT
CTGCATGCGTACTCTTAAGCTATCCAACCCCTCGTACGGTGACCTGAACCACCTGGTTTCCGCTGTCATGTCC
GGTGTTACTACTTGCTTGCGATTCCCTGGTCAGCTAAACTCAGATCTACGCAAGCTCGCTGTAAACATGGTT
CCGTTCCCTCGTCTCCACTTCTTCATGGTTGGATTCGCTCCTCTAACCAGCCGTGGTGCTTACACTTTCCGTGC
TGTCACCGTTCCTGAGTTGACTCA-GCAAAT-
GTTCGACCCCAAGAACATGATGGCTGCTTCTGACTTCCGTAACGGTCGCTACCTAACGTGCTCTGCCATCTT
GTAAGATACT-CT----CTTCCCGAA----------GCATT--------------------------TGTTACTAC----CC-
ATTACTAACATA-----TTTTTCTAGCCGTGGCAAGGTCACTGGTAAGGAGGTTGAGGACCAGATGCGC---------
----

>H_aveirense_MUM_19_40

--CGAGTC-----
CCAATCTGTGTGCCACCGCCCAACGAGGCCCCTGA-----ACGCG-TCCG--------------AGGAAA------------CAT-
--CCAA---TTCCC-CTGA------T------G-TCTGCCCCT-----CACGCAC---------ACG-------------------------------------
----------------AAACGCACGACAGCTATC---------------TCAGCAGTCCTCA--TTCCAC----CC-----TTC---GCCG---
CCAT------GTCGCTGTCTGA-TTTTAC-TTA------------C---TAC----TTG--A------------------------TGCAACGTGG-
-------AAGTGGAAGTGAAGGCTAA-TCCGA---TA---TTTTCTC------------

ATATATAGGTTCACCTCCAGACCGGCCAGTGCGTAAGTACAACCAG---CAGC-------CTC-GATC-------
GACGAGACGTC-----------------GCGATGGA--------------AAAC-ATAGC------CAGCTCACACGA--TA-GCA-----
AA-TAGGGTAACCAAATTGGTGCTGCTTTCT---
-GGCAGACCATTTCTGGCGAGCACGGTCTCGACAGCAACGGTGTGTACGTATTA-----------------------
GGGTCGCC-------------------AATTTCGATTGTC-------CATCA--------------------------CAGC--CAGC-------TAAC-
----------GAC-CAAT-
AAACAGGTACAATGGAACCTCGGAGCTCCAGCTCGAGCGCATGAGCGTCTACTTCAACGAGGTACGCGGT-
CGAAG-----------------A----------A----GCC-----AC-----------------------C-CGTTTT-GT-G--------AGATCGG-TT-
A-----TTAAT--CAC-CC-TAA---CATAC-
ACAGGCTTCTGGTAACAAGTATGTTCCCCGCGCCGTCCTCGTCGATCTCGAGCCCGGTACCATGGACGCCGT
CCGTGCCGGCCCCTTCGGTCAGCTCTTCCGTCCCGACAACTTCGTCTTCGGTCAGTCCGGTGCCGGCAACAA
CTGGGCCAAGGGTCACTACACCGAGGGT--

>H_griseobrunneum_CBS_331_73

ATCCATGTGCCACCACCCAACGAGGCCCCTGA-----ACGCG-TCCG--------------AGAGAG------------ATG---
CCAA---TTCCC-CTGA------T------A-TCTACCCCT-----CACGTAC---------ACC--
-------------AAA--CACGACAGCTATC---------------ATCACAATCGACT--GTCTAG----CC-----TTC---GCAG---
TCAT------GTCGCCGTCTGA-TTT-----TA------------C---TAC----TTG--A------------------------CGAAACGTAG----
----AATTCGAAATGGAAGCTAA-CTCAA---TA---TTTTC--------------
ATGCATAGGTTCACCTCCAGACCGGCCAGTGCGTAAGTACAACCAA---CAAC-------CTC-GATC-------
GAGGAAACGTC-----------------GCGATGGGA-----------GAAAAC-ATGGC------CAGCTCACACGA---T-ATC----
-AA-CAGGGTAACCAAATTGGTGCTGCTTTCT--
---GGCAGACCATCTCTGGCGAGCACGGTCTCGACAGCAACGGTGTGTACGTATCA-----------------------
AGATCGCC-------------------CGCTCCGATTGTC-------AAGAA-------------------------TCAAC--AAAT-------
TAAC-----------GAC-CAAT-
CAACAGCTACAATGGCACCTCCGAGCTCCAGCTCGAGCGCATGAGCGTCTACTTCAACGAGGTACACAGT-
CAAAG-----------------A----------A----GCC-----TC-----------------------C-CCTTTT-GC-G--------AGATCGG-TT-A-
----TTAAT--CAC-CC-TAA---CATAC-
ACAGGCTTCTGGTAACAAGTATGTTCCCCGCGCCGTCCTCGTCGATCTCGAGCCCGGTACCATGGACGCCGT
CCGTGCCGGTCCCTTCGGTCAGCTCTTCCGCCCCGACAACTTCGTCTTCGGTCAGTCCGGTGCCGGCAACAA
CTGGGCCAAGGGTCACTACACGGAGGGTGC-TGAGCTTGTCG-
ACAACGTTCTCGATGTCGTCCGTCGCGAGGCTGAGGGCTGCGACTGCCTCCAGGGCTTCCAGATCACCCAC
TCCCTCGGTGGTGGTACCGGTGCCGGTATGGGTACCTTGTTGATCTCCAAGATCCGCGAGGAGTTCCCCGA
CCGCATGATGGCCACCTTCTCCGTCGTGCCCTCTCCCAAGGTCTCCGACACCGTCGTCGAGCCCTACAACGC
CACCCTCTCCGTGCACCAGCTCGTCGAGAACTCCGACGAGACCTTCTGTATCGACAACGAGGCCCTGTACGA
CATCTGCATGCGTACCCTGAAGTTGTCCAACCCCTCGTACGGTGACCTGAACCACCTAGTCTCTGCCGTCAT
GTCCGGTGTCACCACCTGTCTGCGCTTCCCCGGCCAGCTGAACTCGGACCTGCGCAAGCTCGCTGTGAACAT

GGTTCCTTTCCCTCGTCTCCACTTCTTCATGGTCGGCTTCGCTCCCCTGACCAGCCGTGGTGCCTACTCCTTCC
GCGCCGTCACCGTTCCCGAGTTGACTCA-GCAGAT-
GTTCGACCCCAAGAACATGATGGCTGCCTCTGACTTCCGCAACGGTCGCTACCTGACGTGCTCTGCCATCTT
GTAAGATTCC-CT----ACCGACAAAATAC-----CCGACA-------------------------TATAGTGAT-TGA---
TGTGCTAACCCA----
ACTTCTCCAGCCGTGGCAAGGTCTCCATGAAGGAGGTTGAGGACCAGATGCGCAACGTCAG-----------------
----

>H_haematostroma_MUCL_53301

--------ACCCCCTGCA-----ACGCG-TCTT-------------GGTTT-------------CTC-----------CCCCT-TGGA------T------
T-TGCACCCCT-----CGCCCATCACCTGCACGCA---AAC--
CATCAACGCA------------------------TTC------AACGGT--------------------------------TCCAACCTTATA-CCG-----
-----------------AAC--------------------------------TATCAC-----------------GCGAATCGAAGCTAA-TCATG---TC---
TCTTCCT-----------ATCTATAGGTTCACCTTCAAACCGGCCAGTGCGTAAGTGCGAGGCCCT-CGAC-----------
GACACCC----GATGATTTGTA----------------GCAGGA-------------GAAGGT-TCCACAG-----
AACTCATACAA--GA-GAT-----TA-TAGGGTAACCAAATCGGTGCTGCTTTCT-------------------------------------
----
GGCAAACCATCTCTGGCGAGCATGGTCTCGACAGCAATGGCGTGTACGTATTT----------------------
TATTCGAC-------------------AATTCCAATCTTG-------AGAAT-----------------------CACCCG--TAAC-------
TAAC-----------AAC-CAAT-
AAACAGCTACAACGGAACCTCTGAGCTCCAGCTCGAGCGCATGAGCGTCTACTTCAACGAGGTACATACC-
TATCATTG-------------A---------G----ATC-----CG-TAAC------------------GGATAC-GC-GCG-----
ATGAATAG-TC-A-----CTGAC--TGT-AC-TGA---
CACGCAACAGGCTTCCGGTAACAAGTATGTTCCCCGTGCCGTTCTCGTCGATCTCGAGCCCGGTACCATGGA
CGCCGTCCGTGCTGGTCCCTTCGGTCAGCTCTTCCGTCCCGACAACTTCGTCTTCGGCCAATCTGGTGCCGG
CAACAACTGGGCCAAGGGTCACTACACTGAGGGTGC-TGAGCTTGTTG-
ACAACGTCCTCGACGTTGTCCGTCGTGAGGCTGAGGGCTGTGATTGCCTCCAGGGTTTCCAGATCACCCACT
CACTCGGTGGTGGTACCGGTGCCGGTATGGGTACCTTGTTGATCTCCAAGATTCGCGAGGAGTTCCCCGAC
CGCATGATGGCAACCTTCTCCGTCGTTCCCTCCCCCAAAGTTTCCGACACCGTCGTCGAGCCTTACAATGCCA
CTCTCTCCATCCACCAGCTGGTTGAGAACTCGGACGAGACCTTCTGCATTGATAACGAGGCCCTCTACGACA
TCTGCATGCGCACGCTTAAGTTGTCCAACCCGTCTTACGGCGACCTGAACCACCTGGTCTCCGCCGTCATGT
CTGGCGTTACCACTNGCTTGCGATTCCCCGGCCAGTTGAACTCCGATCTGCGCAAGCTTGCCGTCAACATGG
TTCCCTTCCCTCGTCTCCACTTCTTCATGGTCGGCTTTGCTCCCCTGACCAGCCGCGGCGCCTACTCCTTCCGC
GCCGTTACCGTCCCCGAGTTGACCCA-GCAGAT-
GTTCGACCCCAAGAACATGATGGCTGCTTCCGACTTCCGCAGCGGTCGATACCTGACATGCT-
TGCCATCTTGTAAGATATT-CA----CCCCATT--

>H_lienhwacheense_MFLUCC_14_1231

-ATTATTATTATCATTATCA-------------------------------------TCGTCATTATCATTATTATTTTA-----------------
--------GTGACTACACCCTACCTTGATCTCCTCCCCCCCCCTTGA-----ACGCG-TCGTCT-----------CTAAC------
-------TCC---CAAC---ACCTATTCAT------TGCTATTT-ACTACCCCT-----CATATAC---------ACG------------------
------------------------------TAA--AATCATCCTGTTC--------------ATCTTCATCT------CGTCGATGTTT-----

TTA---TTTA---CCTC------GTCATCATTAAA-TCG----CTA----------------TATCCTTTCA--------------------------
ATCACT---------------TGGAATCTCAAGCTAA-TCGCG---TGTTTTTTTAAT------------
CCATATAGGTTCACCTTCAGACCGGCCAATGCGTAAGTTATCTCCAC--CACG---------------------
AAGCAAACATG-----------------GTTATG----------------ATCT-ATGGCGG---CGAGCTCACATCA---A-CAT-----
GA-TAGGGTAACCAAATTGGTGCCGCCTTCT---
--GGCAAACCATCTCCAGCGAGCATGGTCTCGACAGCAATGGAGTGTATGTATCT-----------------------
AATTCGAC-------------------AATCATCGTCGCC-------AAGGA---------------------------TATA-GAAC-------
TGAC-----------CAC-TGAT-
AAACAGGTACAATGGAACTTCCGAGCTCCAGCTTGAGCGCATGAGCGTCTACTTCAACGAGGTACGAATC-
CGAAGCCGCGTCTGTAGAATAGA----------A----ATTGGGGTGATGCAT--------------------GTACGC-GT-A------
--GGATCGG-TTAA-----CTAAC--CTC-TT-CCA---CCTTC-
GCAGGCCTCTGGCAACAAGTATGTCCCTCGCGCCGTTCTCGTCGATCTCGAGCCCGGTACTATGGACGCTGT
CCGTGCTGGTCCCTTCGGTCAGCTCTTCCGACCCGACAACTTCGTCTTTGGTCAGTCCGGTGCCGGAAACAA
CTGGGCCAAGGGTCACTACACCGAGGGTGC-TGAGCTCGTCG-
ATCAGGTCCTTGACGTCGTCCGCCGCGAGGCTGAAGGCTGTGACTGCCTCCAGGGCTTCCAGATCACCCAC
TCTCTCGGTGGTGGTACCGGTGCCGGTATGGGTACCCTGCTTATCTCCAAGATTCGCGAGGAGTTCCCCGAC
CGCATGATGGCTACTTTCTCCGTCATGCCCTCCCCCAAGGTCTCTGACACTGTCGTCGAGCCCTACAACGCTA
CCCTCTCCGTTCACCAGCTGGTCGAGAACTCGGACGAGACCTTCTGCATTGACAACGAGGCTCTCTACGACA
TCTGCATGCGTACCCTGAAGTTGTCCAACCCCTCGTATGGCGACCTGAACCACTTGGTCTCCGCTGTCATGTC
CGGTGTCACCACCTGTCTGCGTTTCCCTGGTCAGCTGAACTCCGACCTCCGCAAGCTCGCCGTGAACATGGT
TCCCTTCCCTCGTCTCCACTTCTTCATGGTCGGCTTTGCTCCCTTGACTAGCCGTGGTGCTCACTCTTTCCGCG
CCGTGACCGTGCCCGAGTTGACCCA-GCAGAT-
GTTCGACCCGAAGAACATGATGGCTGCCTCCGACTTCCGCAACGGTCGATACCTGACGTGTTCTGCGATCTT
GTATGATACC-TT----GCCCCCCCCTCGT------CTCTA-------------------------TCTCGCTAC-TGA---
TCTGCTAACATG----ATGTCTCTAGCCGCGGCAAGGTCTCCA---

>Rostrohypoxylon_terebratum_CBS_119137

--AAAGTCATCGTATGCTTC------------------------------------
TGCTGACCCGGCCCACCACCGAGACGACCCCTGA-----GCGCG-TCCA--------------AGAA--------------CTC---
CAAA---ACCCC-TTGA------T------T-TCTGCCCCT-----CCCGCAC---------ACA--------------------------------------
--------------AAA--CACCACAACA------------------------TTG------CATGG----TG-----TTC---TGTG---TTAC------
ACCAGCATCAAA-TTT-----CG------------C---AGT------A--------------------------TTTATTCTA-------------
TGAATCGAAGCTAA-CCGCG-------CATTTCAT------------
TTCAATAGGTTCACCTCCAGACCGGCCAATGCGTAAGTGCTACGAT--------------TAC-GACTACC----
GACAAGATATT-----------------TCGCTG----------------GGAT-ATAGCGG-----GGCTCACACGA---A-GAT------
C-TAGGGTAACCAAATTGGTGCTGCTTTCT--
GGCAAACCATCTCTGGCGAGCACGGTCTCGACAGCAATGGCGTGTAAGTGTTT----------------------
TAGTTGTC-------------------AATTCGAAATGCC-------AAGAA---------------------------TAT--CAAC-------TAAT--
---------TAC-GAAT-
AAACAGCTACAACGGAACTTCCGAGCTCCAGCTCGAGCGCATGAGCGTTTACTTCAACGAGGTACGCAAC-
CAGGG-----------------A----------A----AAC-----AA-ACTTA-------------------ACAAAC-AG-A--------AGAATAG-
TT-A-----CTAAT--CAC-CC-TAA---CATGC-
ACAGGCATCTGGTAACAAGTATGTTCCCCGAGCCGTCCTCGTCGATCTCGAGCCCGGTACGATGGATGCCG

TCCGCGCTGGTCCTTTCGGTCAGCTCTTCCGTCCCGACAACTTCGTCTTCGGCCAGTCTGGTGCCGGAAACA
ACTGGGCCAGGGGTCACTACACTGAGGGTGCGTGAGCTAGTGACACACGGTCTCTGATGTCGTCGTCGTG
GAGGCTGAAGGTTGCGACTGCCTTCCAGGTTTCCAGATCCACCACTCTCTTGGTGGTGGTACCGGTGGCGG
TATGGGTACTCTTGTGATCTTCCAGATTCGTGAAGAG-
TCCCCGACCGCATGATGGCCACCTTTTCCGTCGTTCCCTCTCCCAAGGTTTCCGACACCGGTGGTGAGCCTTA
CAACGCCACTCTTTCCGTTCACCAGTTGGTTGAGAACTCGGACGAGACCTTTTGCATTGACAACGAGGCTTT
GTACGACATTCGCATGCGTACCCTGAAGCTATCTAACCCCTCGTACGGCGACCTGAAACACCTGGTCTCCGC
CGTCATGTCCGGTGTCACCACCTGTTTGCGGTTCCCCGGTCAGCTGAACTTTGACCTTCGCCAGCTCGCCGT
GAACAAGGTTCCTTTCCCCCGTCTTCAATTCTTCATGGTTGGCTTCGCACCCCTGACCAGCCGTGGCGCCTAC
TCCTTTCGCGCCGTCACCGTACCCGAGTTGACCCA-GCAGAT-
GTTTGACCCCAAGAACCTGATGGCCGCTTTTGACCTCCGCAACGGTCGTTACCTGACGTGGTTTGCCATTTT
GTAAGCTTAC-CT----TTTCTATT-----------CGATT-------------------------TGTTATCGA-GGA---
GATGCTAACCCC-----
CATTTCTAGCCGTGGCAAGATCTCCATGAAGGAGGTTGAGGACCAGATGCGCAACGTCCAGAACAAGAACT
CGTCGACCAG-----------------------------

>X_arbuscula_CBS_126415

---CCTCCCTCTGTTTACTTTTCAACC--------------------------
CAAGCTCCGCCAGAATTCTGCTACTTTCCGCCCGAG----ACGCG-TTAC-------------ATGTCT------------CTC---
TAAT--AGCTTC-TCATC-----TCCC-----ATCATCTCT-----CATCTGC---CTTAGCATG--------------------------------
--------------------ATA--AATTACTAGA--------------------CAATATTT---------------CT-----GTC---
ATCAGAGGCAT----GGAGCTTGATTGAACATTCTC-CCG--------TTATCTGGCAC------A--A----------------------
-TATTGG---------------GTGAAACCTAAACTAA-CCGCG----------CTCCT------------
CTCTACAGGTCCACCTCCAGACCGGCCAATGCGTAAGTCGCTTTAC---GACC-------TTC-GACG-----------------
------------------ATAATACC------------TTGAAA-CAGCCGA-----AACTTACATAG---A-ATG-----AT-
CAGGGTAACCAAATTGGTGCTGCTTTCT---
GGCAGCAAATCTCCGGCGAGCACGGTCTCGATGGCAGTGGCGTGTATGTGTAC----------------------
CTATGCCC--------------TTCGGGGTCATGGACTAT-------AAGGA------------------------CACAA--TGAC-------
TGAT-----------AGT-TATG-
GAATAGTTACCAGGGAACCTCTGACCTCCAGCTGGAGCGTATGAGGGTTTACTTCAACGAGGTAGGCTCA-
AATTC-----------------C----------G----AGC-----TA-CATC--------------------GATATC-AT-A--------CAAGTAA-TG-
GT----CTAAC------AT-GGG---TTTGT-
TCAGGGCTCGGGCAACAAGTACGTTCCTCGCGCTGTCCTCGTCGATTTAGAGCCTGGTACCATGGACGCTGT
CCGTGCTGGTCCCTTCGGTCAGCTCTTCCGACCCGACAACATCGTCTTCGGTCAGTCTGGTGCTGGCAACAA
CTGGGCCAAGGGTCACTACACTGAGGGTGC-TGAGCTTGTTG-
ACAACGTTCTTGACGTTGTCCGTCGTGAGGCTGAGGGCTGTGACTGCCTCCAGGGTTTCCAGATCACCCACT
CGCTCGGTGGTGGTACCGGTGCCGGTATGGGTACGCTGCTCATCTCCAAGATCCGTGAGGAGTTCCCCGAC
CGCATGATGGCTACCTTCTCCGTCATGCCCTCCCCCAAGGTATCGGACACCGTCGTCGAACCTTACAACGCC
ACTCTCTCCGTCCACCAGCTGGTCGAGAACTCCGACGAGACCTTCTGCATTGACAACGAGGCTCTCTACGAC
ATCTGCATGCGCACCCTGAAGCTATCCAACCCTTCGTACGGTGACCTGAACCACCTTGTCTCCGCTGTCATGT
CTGGCGTCACCACTTGCCTTCGTTTCCCTGGACAACTTAACTCTGACCTGCGCAAGTTGGCCGTCAACATGGT
GCCCTTCCCTCGTCTGCACTTCTTCATGGTCGGCTTCGCCCCTTTGACCAGCCGTGGTGCCCACTCTTTCCGT
GCCGTCACGGTTCCTGAGTTGACCCA-GCAAAT-
GTTCGACCCCAAGAACATGATGGCCGCCGCTGACTTCCGCAACGGTCGCTACCTGACATGCTCTGCCATCTT

GTGAGTACTC-TT----ACTTCGAGCTT-------------------------------------CTAACATGT-TTG---
ATTACTAATCTGCTACCCATCACTAGCCGTGGCAAGGTCTCTATGAAGGAGGTTGAGGACCAGATGCGAAA
TGTGCAGAAC---

>X_hypoxylon_CBS_122620

TCCGTCCATTATTCGGCTGCCTTATACCGAA-----ACGCG-TCCC-------------AAAT-------------CTA---CAAT---
GCCCT-TGAT------C--------TTAGCTTCTC----CACATAT---------ACA---
---CAT--CAGCATCGTTCGC------------AGTCAAACTATCT------CGTAG----CT-----GCC---AATA---ACAC------
AGCTTCCTTGAACCTA----CTAGA------TTGTCT--AGT----GAAATACC---------------------TATTAT------------
ATGAGAACTACCTGCTAA-CCATG-------CTTTTCCC-----------
TTTTGCAGGTTCACCTCCAAACCGGCCAATGCGTAAGTCGCCCCCC---GATC-------CTC-GATA----------------
-----------------ACGATGTC-----------TAGAAC-CTCCCGA-----GGCTCACACTA-----TAC-----GA-
CAGGGTAACCAAATTGGTGCTGCTTTCT--
GGCAACAAATTTCCGGCGAGCACGGTCTCGATGGCAGTGGCGTGTATGTCTATTA-------------------
GATCTATG------------------AACTACGACAACC-------ACGAC---------------------TGAATGGT--CGAC-------
TAAC-----------ACT-TGTG-
GGCCAGTTACAACGGAACCTCTGAGCTCCAGCTGGAGCGCATGAGCGTTTACTTCAATGAGGTAGAAAGC-
CCCAT----------------C---------A----AGT-----CG-CGTT-------------------CGTCTC-GT---------GCAACAA-CC-
ATT---TTGATG-----CT-GAT---ATTTT-
CTAGGGTGCTAATAACAAATATGTTCCTCGCGCCGTCCTCGTCGACTTGGAGCCCGGTACCATGGATGCTGT
CCGTTCTGGTCCCTTTGGTCAGCTCTTCCGACCCGACAACTTCATCTTCGGCCAGTCTGGTGCTGGCAACAAC
TGGGCCAAGGGTCACTACACAGAGGGTGC-TGAGCTCGTTG-
ACGCCGTTCTTGATGTCGTTCGTCGCGAGGCTGAGGGCTGCGATTGCCTCCAGGGTTTCCAGATCACCCACT
CGCTCGGTGGTGGTACCGGTGCTGGTATGGGTACTCTGCTGATCTCCAAGATTCGCGAGGAATTCCCTGAC
CGCATGATGGCTACCTTCTCCGTCATGCCCTCTCCCAAGGTCTCAGATACCGTCGTCGAGCCTTACAACGCTA
CCCTCTCCGTCCACCAGTTGGTTGAGAACTCCGATGAGACCTTCTGTATTGACAACGAGGCTCTGTACGATA
TCTGCATGCGCACCTTGAAGCTATCCAACCCCTCATATGGTGATTTGAACCACCTTGTCTCTGCCGTCATGTC
TGGCGTAACCACCTGCCTGCGTTTCCCCGGTCAGCTTAACTCTGATCTGCGCAAACTAGCCGTCAACATGGT
GCCCTTCCCTCGTCTACACTTCTTTATGGTCGGGTTTGCCCCTCTCACTAGCCGTGGTGCCCACTCTTTCCGTG
CTGTCACGGTTCCCGAGCTGACCCA-GCAAAT-
GTTCGACCCCAAGAACATGATGGCTGCCGCTGACTTCCGCAACGGTCGTTACCTCACATGCTCTGCTATCTT
GTAAGAATTT-GC----CCTTTTATCTT-------------------------------------TTAACACGG-CCA---
ATTGCTAACTTATC-CTTTTTACCAGCCGTGGCAAGGTTTCCA--

>Graphostroma_platystomum_CBS_270_87

---CCC--
CCCCTCCAATTCCCATAACTAGAAAGATCCCCGA-----ACGCG-TCCT-------------AAAAGCCC---------CCC---
CAAAACGCCCCC-TGGCT-----T------T-GCTACCCCTCC-AACAAGCTC-------TCGCA-----------------------------
--------------------AGC--AATCATCATG------------------------TCA------TGTAA-----------------------ACAC------
ACCCTCGTGGAT-TCG---
AACATGGCAAGCTAA-CCGCG---TC---
TTTTTTCTTTTTTTTCGTGTTTTATAGGTTCACCTCCAGACCGGCCAATGCGTAAGTACTTCTGT---CCTC-------

AAC-GACGACGCC--GACTAAGT--------------------GCGCTTGC--------------GAAC-ATGGCGAG----
GGCTCACATATCTCATATT-----CC-TAGGGTAACCAAATCGGTGCTGCTTTCTGGTGTGTAG---
CTTACTACATCAACAAACACCCT--------
CCGCCGACATGGAAATATTAACTCGCGCATTCTAGGCAAACCATCTCTGGCGAGCACGGTCTCGACAGCAA
TGGCGTGTACGTATCT-----------------------ACCCTCCT-------------------CTTTTCCTCCTTT-------
ACATACCCTACTTTTATGCGACTGTGGCATCGGAA--AGGC-------TGAC-----------CGG-TTCT-
CTATAGTTACAACGGCACGTCCGAGCTCCAGCTGGAGCGGATGAGCGTGTACTTCAACGAGGTAATGTGG-
TCGGA------------------T---------GTGGCACC--------TGGT--------------------AGACTC---------------------TT-
GTGCCCCTAAT--TAA-CC-TGAATTGGTGT-
GCAGGCCTCTGGTAACAAGTATGTTCCTCGCGCCGTCCTCGTCGATCTCGAGCCTGGCACCATGGATGCCGT
CCGCGCTGGTCCTTTCGGCCAGCTCTTCCGTCCCGACAACTTCGTCTTCGGTCAGTCTGGTGCTGGCAACAA
CTGGGCCAAGGGTCATTACACTGAGGGTGC-CGAGCTGGTTG-
ACCAGGTTTTGGATGTTGTTCGTCGTGAGGCTGAGGGCTGCGACTGCCTCCAGGGCTTCCAGATCACCCACT
CCCTCGG--

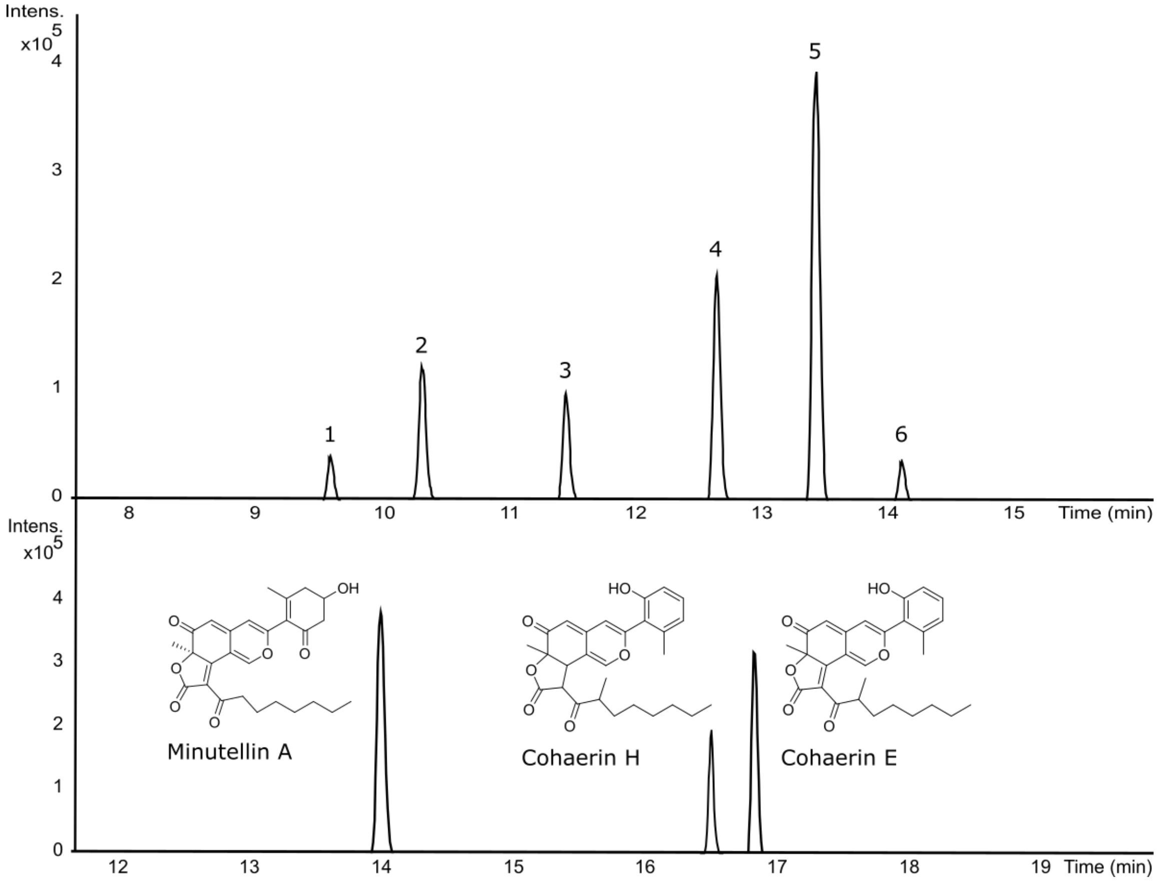

Figure S2. Extracted ion chromatogram of the stromatal metabolites **1**–**6** from the *Parahypoxylon* spp. and standards of minutellin A and cohaerin E.

Table S7. Dereplicated metabolites (1–6) from the stromatal extracts of the *Parahypoxylon* spp and in-house standards.

**Compound**	***m/z***	**rt**	**CCS**	**Formula**	**Annotation***
1	413.15950 $[M+H]^+$	9.61	204.1	$C_{23}H_{24}O_7$	Level 2
2	411.14418 $[M+H]^+$	10.37	204.1	$C_{23}H_{22}O_7$	Level 2
3	427.13902 $[M+H]^+$	11.49	194.2	$C_{23}H_{20}O_8$	Level 2
4	395.14902 $[M+H]^+$	12.67	196.8	$C_{23}H_{22}O_6$	Level 2
5	393.13320 $[M+H]^+$	13.50	197.5	$C_{23}H_{20}O_6$	Level 2
6	392.14921 $[M+H]^+$	14.14	193.4	$C_{23}H_{21}NO_5$	Level 2
Minutellin A	467.20641 [M+H]+	14.02	221.7	$C_{27}H_{30}O_7$	Level 0
Cohaerin E	463.21159 [M+H]+	16.85	217.4	$C_{28}H_{30}O_6$	Level 0
Cohaerin H	465.22654 [M+H]+	16.60	228.1	$C_{28}H_{32}O_6$	Level 0

*Level 0 corresponds to data obtained from in-house standards; level 1 to annotations by comparison with standards; level 2 to putative annotations (i.e., MS/MS library search); level 3 to a compound class assignment. rt = retention time in min; CCS = collisional cross section in $Å^2$.

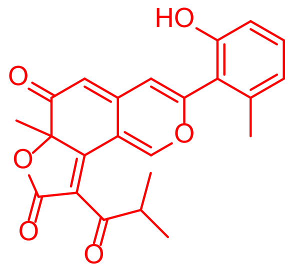

Chemical Formula: $C_{23}H_{20}O_6$
Molecular Weight: 392.41

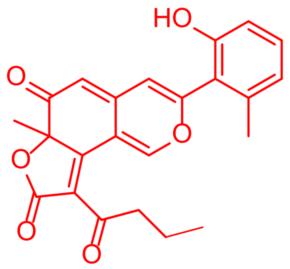

Chemical Formula: $C_{23}H_{20}O_6$
Molecular Weight: 392.41

Putative azaphilones from *Parahypoxylon* spp.

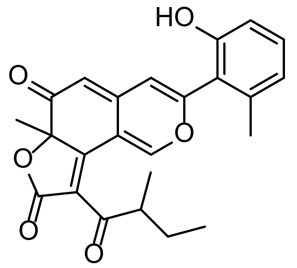

Chemical Formula: $C_{24}H_{22}O_6$
Molecular Weight: 406.43

Multiformin type azaphilones

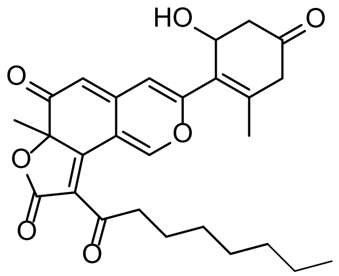

Chemical Formula: $C_{27}H_{30}O_7$
Molecular Weight: 466.53

Minutellin type azaphilones

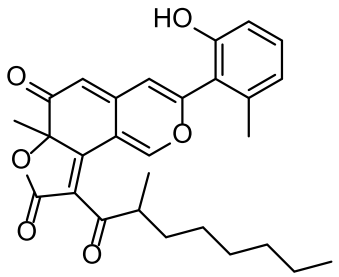

Chemical Formula: $C_{28}H_{30}O_6$
Molecular Weight: 462.54

Cohaerin type azaphilones

Figure S3. Observed different carbon skeletons for the known cohaerin type azaphilones in the Hypoxylaceae.
